# The ongoing COVID-19 epidemic in Minas Gerais, Brazil: insights from epidemiological data and SARS-CoV-2 whole genome sequencing

**DOI:** 10.1080/22221751.2020.1803146

**Published:** 2020-08-11

**Authors:** Joilson Xavier, Marta Giovanetti, Talita Adelino, Vagner Fonseca, Alana Vitor Barbosa da Costa, Adriana Aparecida Ribeiro, Katlin Nascimento Felicio, Clara Guerra Duarte, Marcos Vinicius Ferreira Silva, Álvaro Salgado, Mauricio Teixeira Lima, Ronaldo de Jesus, Allison Fabri, Cristiane Franco Soares Zoboli, Thales Gutemberg Souza Santos, Felipe Iani, Massimo Ciccozzi, Ana Maria Bispo de Filippis, Marilda Agudo Mendonça Teixeira de Siqueira, André Luiz de Abreu, Vasco de Azevedo, Dario Brock Ramalho, Carlos F. Campelo de Albuquerque, Tulio de Oliveira, Edward C. Holmes, José Lourenço, Luiz Carlos Junior Alcantara, Marluce Aparecida Assunção Oliveira

**Affiliations:** a Laboratório de Genética Celular e Molecular, Universidade Federal de Minas Gerais, Belo Horizonte, Minas Gerais, Brazil; b Laboratório de Flavivírus, Instituto Oswaldo Cruz, Fundação Oswaldo Cruz, Rio de Janeiro, Brazil; c Laboratório Central de Saúde Pública, Fundação Ezequiel Dias, Belo Horizonte, Brazil; d KwaZulu-Natal Research Innovation and Sequencing Platform (KRISP), College of Health Sciences, University of KwaZuluNatal, Durban 4001, South Africa; e Laboratório de Vírus Respiratórios e Sarampo, Instituto Oswaldo Cruz, Fundação Oswaldo Cruz, Rio de Janeiro, Brazil; f Coordenação Geral dos Laboratórios de Saúde Pública/Secretaria de Vigilância em Saúde, Ministério da Saúde, Brasília, Distrito Federal, Brazil; g Secretaria de Estado de Saúde de Minas Gerais, Belo Horizonte, Brazil; h Organização Pan-Americana da Saúde/Organização Mundial da Saúde, Brasília-DF, Brazil; i Marie Bashir Institute for Infectious Diseases and Biosecurity, School of Life and Environmental Sciences and School of Medical Sciences, University of Sydney, Sydney, NSW, Australia; j Department of Zoology, University of Oxford, Oxford OX1 3PS, UK; k University Campus Bio-Medico of Rome, Rome, Italy

**Keywords:** SARS-CoV-2, genomic surveillance, Minas Gerais, southeast Brazil, pandemic, sequencing, genomic epidemiology

## Abstract

The recent emergence of a coronavirus (SARS-CoV-2), first identified in the Chinese city of Wuhan in December 2019, has had major public health and economic consequences. Although 61,888 confirmed cases were reported in Brazil by 28 April 2020, little is known about the SARS-CoV-2 epidemic in this country. To better understand the recent epidemic in the second most populous state in southeast Brazil - Minas Gerais (MG) - we sequenced 40 complete SARS-CoV-2 genomes from MG cases and examined epidemiological data from three Brazilian states. Both the genome analyses and the geographical distribution of reported cases indicate for multiple independent introductions into MG. Epidemiological estimates of the reproductive number (R) using different data sources and theoretical assumptions suggest the potential for sustained virus transmission despite a reduction in R from the first reported case to the end of April 2020. The estimated date of SARS-CoV-2 introduction into Brazil was consistent with epidemiological data from the first case of a returned traveller from Lombardy, Italy. These findings highlight the nature of the COVID-19 epidemic in MG and reinforce the need for real-time and continued genomic surveillance strategies to better understand and prepare for the epidemic spread of emerging viral pathogens..

## Introduction

The World Health Organization (WHO) office in China was informed about a cluster of new cases of pneumonia of unknown aetiology in the city of Wuhan (Hubei province), in late December 2019 [1]. Shortly afterwards, a new type of coronavirus, now termed SARS-CoV-2, was isolated and identified by Chinese authorities, with its genetic sequence shared with the international community on 10 January 2020 [2–5]. Phylogenetic analysis revealed that SARS-CoV-2 was similar to other (epidemic) betacoronaviruses, such as severe acute respiratory syndrome coronavirus (SARS-CoV) and Middle East respiratory syndrome coronavirus (MERS-CoV) [4,5], and was clearly related to coronaviruses isolated from bats and Malayan pangolins (*Manis javanica*) indicative of a zoonotic origin [2,5–7].

To date, more than 10.7 million cases of the disease caused by SARS-CoV-2, termed COVID-19, have been reported globally [8,9]. On 11 March 2020, the WHO declared a pandemic, prompting a dramatic increase in international concern and response [10]. On 26 February 2020, the first confirmed case of COVID-19 was reported in São Paulo (SP) state, Brazil [11]. Two months later (28 April 2020), 61,888 cases and 4,205 deaths attributed to COVID-19 had been reported in Brazil [12]. Initial phylogenetic analysis using the first two SARS-CoV-2 complete genomes isolated in São Paulo from travellers returning from Italy revealed two independent introductions into the country relative to the data set available at that time [13].

The state of Minas Gerais (MG) is the second largest Brazilian state in terms of population size, estimated at approximately 21 million people [14]. Due to its large population size and strong connections to active neighbouring states such as São Paulo and Rio de Janeiro, the state of MG is likely to be highly affected by the COVID-19 pandemic.

Genetic analyses and surveillance allow the characterization of circulating viral lineages, the inference of introduction events and the reconstruction of transmission patterns [15]. Together with epidemiological data they constitute powerful tools to assist public health initiatives and preparedness. Herein, we present a summary of epidemiological data and the generation and analysis of 40 new SARS-CoV-2 genome sequences isolated from clinical samples of confirmed cases from MG. Our aim was to provide a preliminary epidemiological overview of SARS-CoV-2 circulation and introduction events into Minas Gerais.

## Materials and methods

### Ethics statement

Anonymised samples processed in this study were sent to the Central Public Health Laboratory/Octávio Magalhães Institute (IOM) of the Ezequiel Dias Foundation (FUNED), which belongs to public laboratories network from the *Brazilian Ministry of Health* (BMoH). They were previously obtained by the local health services for the routine diagnosis and epidemiological surveillance of SARS-CoV-2. The availability of these samples for research purposes during outbreaks of international concern is allowed under the terms of the 510/2016 Resolution of the National Ethical Committee for Research – Brazilian Ministry of Health (CONEP - Comissão Nacional de Ética em Pesquisa, Ministério da Saúde): this authorizes the use of clinical samples collected in the Brazilian Central Public Health Laboratories to accelerate knowledge building and contribute to surveillance and outbreak response. The study protocol was reviewed and approved by Research Ethics Committee of the Universidade Federal de Minas Gerais with approval No. 32912820.6.1001.5149.

### Sample collection and RT-qPCR diagnosis

Samples used in this study were from residual anonymised clinical samples, with no or minimal risk to patients, provided for research and surveillance purposes as described above. *Swab* samples collected from COVID-19 suspected cases were collected throughout the state of MG and sent to IOM-FUNED facilities. At the IOM-FUNED facilities they were submitted to total RNA extraction with an automated protocol on the QIAsymphony platform using the DSP Virus/Pathogen Kit (Qiagen), following the manufacturer's recommendations. *The molecular diagnosis was performed on a 7500 Real-Time PCR System (Thermofisher Scientific), using a RT-qPCR singleplex kit for the SARS-CoV-2 envelope and RNA-dependent RNA polymerase genes developed by Bio-Manguinhos/Fiocruz (Rio de Janeiro, Brazil) and provided by the Brazilian Ministry of Health, following the manufacturer's recommendations*. We selected 48 samples with RT-qPCR positive results, collected up to 3 April 2020 from patients residing in different municipalities of the state of MG and presenting with symptoms such as fever, cough, headache, dyspnea, *sore throat and/or vomiting. Samples were selected based on cycle threshold (Ct) values *≤* 32. Associated epidemiological data, such as symptoms, travel history and municipality of residency, were collected from medical records accompanying the collected samples provided by IOM/FUNED*.

### cDNA synthesis and sequencing multiplex PCR

For cDNA synthesis, the SuperScript IV Reverse Transcriptase kit (Invitrogen) was used following the manufacturer's instructions. The generated cDNA was subjected to multiplex PCR sequencing using Q5 High Fidelity Hot-Start DNA Polymerase (New England Biolabs) and a set of specific primers designed by the ARTIC Network (https://github.com/artic-network/artic-ncov2019/tree/master/primer_schemes/nCoV-2019/V1) for sequencing the complete SARS-CoV-2 genome [16]. PCR conditions have been previously reported in [16]. All experiments were performed in biosafety level-2 cabinet.

### Whole genome sequencing

Amplified PCR products were purified using the 1x AMPure XP Beads (Beckman Coulter) following previously a published protocol [17]. Purified PCR products were quantified using the Qubit® dsDNA HS Assay Kits (Invitrogen), following the manufacturer's instructions. Of the 48 samples, only 40 contained sufficient DNA (≥ *2ng/µL*) to proceed to library preparation. Sequencing libraries were prepared using the Oxford Nanopore Ligation Sequencing Kit (SQK-LSK109) following previously a published protocol [17]. Before pooling all samples, each sample was barcoded using the Native Barcoding Expansion kits (NBD104 and EXP-NBD114). After barcoding adaptor ligation, sequencing libraries were loaded on a flow cell (FLO-MIN106) for subsequent MinION sequencing, programmed to run for six hours. Reads were *basecalled* using Guppy and barcode demultiplexing was performed using qcat. Consensus sequences were generated by *de novo* assembling using Genome Detective and Coronavirus Typing Tool [18,19].

### Phylogenetic analysis

Public SARS-CoV-2 complete genome sequences available up to 15 April 2020 were retrieved from the GISAID database (https://www.gisaid.org/). Sequences were aligned using MAFFT (FF-NS-2 algorithm) employing default parameters [20]. The alignment was manually curated to remove artefacts at the terminal regions and within the alignment using Aliview [21]. Phylogenetic analysis of these sequences was performed using the maximum likelihood method implemented in IQ-TREE (version 1.6.10), employing the best-fit model of nucleotide substitution according to the Bayesian Information Criterion (BIC) as indicated by the Model Finder application implemented in IQ-TREE [22]. The statistical robustness of individual nodes was determined using 1000 bootstrap replicates.

Lineage assessment was conducted using the Phylogenetic Assignment of Named Global Outbreak LINeages tool available at https://github.com/hCoV-2019/pangolin [23]. Four data sets of complete or near-complete SARS-CoV-2 genome sequences were generated. Data set 1 (*n* = 3,102) comprised the data reported in this study (*n* = 40) plus publicly available SARS-CoV-2 sequences (*n* = 3,062) from GISAID. Subsequently, to investigate the evolutionary behaviour of SARS-CoV-2 within the three different SARS-CoV-2 lineages (A, B and B.1), Bayesian molecular clock analysis was conducted on three smaller subsets of data for each of the three lineages identified in the ML phylogeny and containing isolates from MG (data set 2 *for* subset A *n* = 100; data set 3 *for* subset B *n* = 84; data set 4 *for* subset B.1 *n* = 169). ML trees from these three data subsets were inspected in TempEst v1.5.3 for the presence of a temporal (i.e. molecular clock) signal [24]. Linear regression of root-to-tip genetic distances against sampling dates indicated that the SARS-CoV-2 sequences evolve in a relatively-strong clock-like manner (r = 0·43; r = 0·47; r = 0·40 from subset A; B and B.1, respectively) (Supplementary Figure S18). For more detailed Bayesian time-scaled phylogenetic analysis we employed the BEAST 1.10.4 program [25]. For this analysis we employed the strict molecular clock model, the HKY+Γ4 codon partitioned (CP)1 + 2,3 nucleotide substitution model and the exponential growth coalescent model [26]. We computed MCMC (Markov chain Monte Carlo) triplicate runs of 100 million states each, sampling every 10.000 steps for each data set. Convergence of MCMC chains was checked using Tracer v.1.7.1 [27]. Maximum clade credibility *trees* were summarized from the MCMC samples using TreeAnnotator after discarding 10% as burn-in.

### Epidemiological data assembly

Data used in the epidemiological analysis were retrieved from https://github.com/wcota/covid19br [28].

## Results

**Figure 1.**
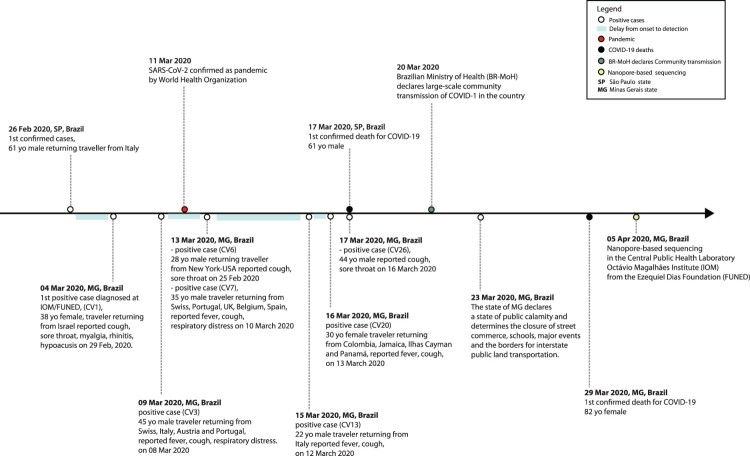
Timeline of key events following the first confirmed case of COVID-19 in Brazil. Events below the line occurred in Minas Gerais (MG) state, while national events are presented above the line. Codes in parentheses refer to the identification code (CV#) of the isolates from the cases described in this study.

**Figure 2.**
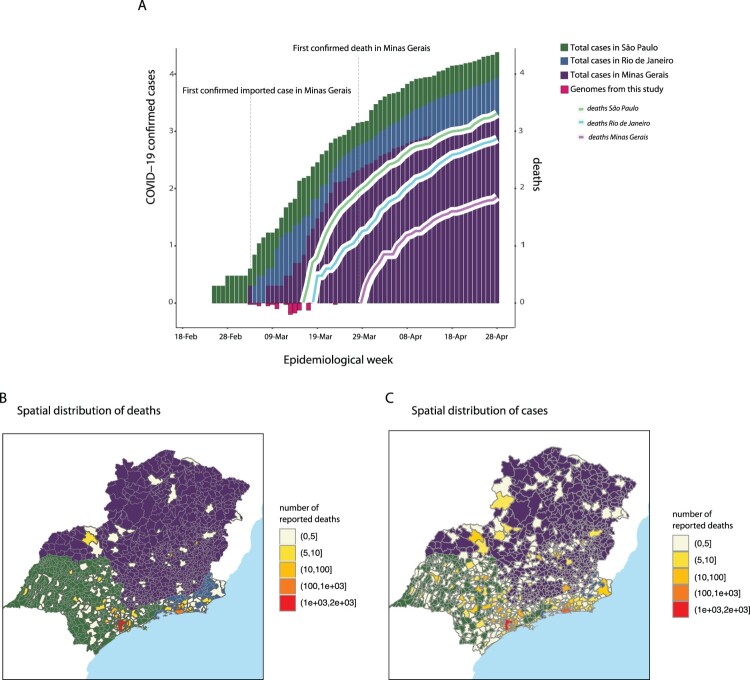
SARS-CoV-2 epidemic curve and spatial distribution of cases and deaths reported in the states of Minas Gerais (MG), São Paulo (SP) and Rio de Janeiro (RJ), Brazil. Panel A: Daily confirmed cases of COVID-19 in the state of MG. The X-axis represents the days from the first case in Brazil until 28 April 2020, while the Y-axis (left-hand side) represents the number of cases. The right-hand side of the Y-axis represents the number of deaths related to COVID-19. Y-axis numbers are represented as log10. Panels B and C: Maps with location (municipality) of (B) deaths and case (C) events, coloured by total number of reports. Different background colours highlight the boundaries of the three states: green for SP, purple for MG, blue for RJ.

After the WHO declared the outbreak of SARS-CoV-2 a Public Health Emergency of International Concern (PHEIC) on 30 January 2020, the Brazilian government declared a Public Health Emergency of National Importance on 3 February 2020, enabling the introduction of measures to prevent and control virus spread [29]. Twenty-three days later, the first confirmed case in Brazil was reported in the city of São Paulo, related to a traveller returning from Lombardy, Italy (Figure 1) [11]. By the 28th April 2020, more than 61,888 COVID-19 cases were confirmed in Brazil, 1,578 of which were from MG (Figure 2A) [30]. Over this period, MG registered 71 COVID-19-related deaths and the capital city, Belo Horizonte, with an estimated population of 2.5 million people, reported 555 cases [30,31]. Figure 2A shows MG's epidemic (reported cases) curve compared to those of two other neighbouring states, São Paulo (SP) and Rio de Janeiro (RJ). Temporal trends reveal a growth in the number of cases in MG, although with SP accounting for the largest number of cases and deaths (24,041 cases; 2,049 deaths) by COVID-19 up to 28th April 2020.

### Epidemiological analyses using cases and mortality time series data from three Brazilian states

Without access to the total number of tests in time and in each state, we obtained a crude estimate of the case fatality ratio (CFR) for MG, SP and RJ as the ratio between the number of reported deaths and cases [32]. Accordingly, CFR was found to increase with time in all states (Supplementary Figure S2), with means from date of first reported case up to the 28 April in each state as 2.67% for MG, 5.39% for RJ and 6.0% for SP. Although all such comparisons should be made with caution, the CFR in SP and RJ was consistently higher than reported in other localities; for example, 2.6% (95% CI 0·89-6·7) for the Diamond Princess cruise ship [33], and 3.67% (95% CI 3.56-3.80) and 1.2% (95% CI 0.3-2.7) and 1.4% (95% CI 0.9-2.1) for different Chinese regions [33–35].

We used the mortality time series (MTS) from MG, SP and RJ to project the (unobserved total) cumulative number of infections, making two main simplifying assumptions: first, that the infection fatality ratio (IFR) of SARS-CoV-2 would be similar in the Brazilian states to that reported elsewhere; and second, that the number of cumulative deaths in each state were well reported. We utilised the IFR estimated by Verity and colleagues (0.66%, CI 95% 0.39-1.33% [34]), for its general use in the modelling literature [36]. The cumulative number of infections in time was taken to be $I(t)=\frac{D(t)}{\frac{\mathrm{IFR}}{100}}$, where D(t) is the cumulative number of deaths. From I(t) we further obtain the observation rate θ of reported cases from $\theta (t)=\frac{c(t)}{I(t)}$ where c(t) is the number of reported cases in time. We found that the observation rate decreased in time for all states, a likely outcome of epidemic growth superseding tracing and testing efforts as the epidemic progressed (Supplementary Figure S1). By 28 April 2020, the last time point analysed, RJ and SP had similar observation rates at 7.6% and 7.74% (respectively), while in MG, where the epidemic started later, the observation rate was 15.3% (i.e. 1 reported case in 7 infections).

To compare transmission potential, we used reported cases (CTS) and mortality time series (MTS) from MG, SP and RJ states to estimate the (effective) reproduction number, R_._ For this, we performed maximum likelihood estimation of the (CTS and MTS) epidemic growth *r* using a phenomenological model, and two theoretical formulations on how *r* relates to R - one based on the SEIR epidemiological framework by Wallinga and colleagues [37], and another on the distribution of the serial interval [36] (see Supplementary Material for details). For all states R was found to decrease in time since the first reported case (Supplementary Figures S5 and S10). When considering the entire period from first reported case to the 28th of April, estimation methods estimated similar R values for each state (Supplementary Figures S6 and S11). For example, when using the CTS and serial interval formulation, R was 1.91 (CI 95% 1.2-3.1) for SP, 1.88 (CI 95% 1.27-2.8) for RJ and 1.82 (CI 95% 1.2-3.25) for MG.

Using geographic information from reported data in each state (Figure 2C), we found cases and deaths to be disproportionally reported in capital cities for the RJ and SP states but not for MG (Supplementary Figures S13-14). Typically, incidence (cases, deaths) are normalized per 100K individuals, taking into account the total population size of each state. Because of the very different spatial dispersion of cases and deaths in MG when compared to SP and RJ, we also calculated the effective population size; that is, the sum of the population sizes of all municipalities with reports. When using reported cases, we found that the effective population sizes were ∼100%, ∼100% and 64% of the total population sizes of RJ, SP and MG, respectively. When using reported deaths, the effective population sizes were ∼95%, ∼92%, and 35% of the total population sizes of RJ, SP and MG, respectively. Overall these numbers suggest that in MG cases and deaths have been reported only in a subset of the overall population, while in the other states SARS-CoV-2 appears widely dispersed. Incidence of reported cases per 100K using the effective population size was ∼60 in SP, ∼51 in RJ and ∼7.85 in MG (Supplementary Figure S7), while incidence of deaths per 100K was ∼5.56 in SP, ∼4.69 in RJ and ∼0.94 in MG (Supplementary Figure S12).

### Phylogenetic analyses of 40 new SARS-CoV-2 sequences from Minas Gerais

In MG, samples from (clinically) suspected cases were screened at the Central Public Health Laboratory/Octávio Magalhães Institute (IOM) of the Ezequiel Dias Foundation (FUNED), which belongs to the public laboratories network of the Brazilian Ministry of Health (MoH). By 3rd April 2020, IOM/FUNED had performed 3,303 RT-qPCR tests for SARS-CoV-2 on swab samples from suspected cases. We used Nanopore sequencing to generate complete genomes from 40 COVID-19 patients in 15 different municipalities in MG (Table 1).

**Table 1.** Information on the 40 sequenced samples from Minas Gerais state.

Project-ID	Lab ID	Sample type	Ct value	Onset date	Collection date	Age	Sex	State	Municipality	Travel information
CV1	47/20	SWAB	20.54	29/02/20	04/03/20	38	F	MG	Ipatinga	Israel
CV2	115/20	SWAB	24.41	06/03/20	08/03/20	44	F	MG	Sete Lagoas	Portugal, Spain
CV3	135/20	SWAB	27.77	08/03/20	09/03/20	45	M	MG	Belo Horizonte	Italy, Switzerland, Austria, Portugal
CV4	242/20	SWAB	21.92	NA	09/03/20	65	M	MG	Juiz De Fora	USA
CV5	252/20	SWAB	29.93	12/03/20	12/03/20	32	M	MG	Belo Horizonte	..
CV6	298/20	SWAB	18.69	13/03/20	13/03/20	28	M	MG	Belo Horizonte	USA
CV7	352/20	SWAB	26.96	10/03/20	13/03/20	35	M	MG	Belo Horizonte	Switzerland, Portugal, England, Belgium, Spain
CV8	399/20	SWAB	22.61	06/03/20	13/03/20	34	M	MG	Belo Horizonte	..
CV9	428/20	SWAB	30.19	06/03/20	13/03/20	33	F	MG	Belo Horizonte	Sao Paulo (Brazil)
CV11	607/20	SWAB	27.92	10/03/20	16/03/20	40	F	MG	Mariana	Germany, Hungary, Czech Republic
CV12	615/20	SWAB	24.9	10/03/20	11/03/20	37	F	MG	Juiz De Fora	USA
CV13	660/20	SWAB	25.69	15/03/20	15/03/20	22	M	MG	Belo Horizonte	Italy
CV16	791/20	SWAB	20.64	15/03/20	16/03/20	52	M	MG	Belo Horizonte	..
CV17	809/20	SWAB	26.95	09/03/20	11/03/20	61	M	MG	Sete Lagoas	Portugal, Spain
CV18	833/20	SWAB	24.54	11/03/20	16/03/20	25	F	MG	Belo Horizonte	..
CV19	836/20	SWAB	22.04	11/03/20	16/03/20	22	M	MG	Belo Horizonte	Rio de Janeiro (Brazil)
CV20	838/20	SWAB	23.52	13/03/20	16/03/20	30	F	MG	Belo Horizonte	Colombia, Jamaica, Cayman Islands, Panama
CV21	842/20	SWAB	16.67	05/03/20	16/03/20	56	F	MG	Bom Despacho	-
CV22	895/20	SWAB	27.92	15/03/20	16/03/20	20	F	MG	Mariana	Germany
CV24	1028/20	SWAB	25.15	13/03/20	16/03/20	22	F	MG	Uberlândia	..
CV26	1078/20	SWAB	18.76	16/03/20	17/03/20	44	M	MG	Belo Horizonte	..
CV27	1166/20	SWAB	22.99	11/03/20	17/03/20	60	F	MG	Boa Esperança	..
CV28	1142/20	SWAB	22.21	13/03/20	17/03/20	46	F	MG	São João Del Rei	USA
CV31	1274/20	SWAB	17.38	16/03/20	17/03/20	35	M	MG	Betim	..
CV32	1290/20	SWAB	16.41	17/03/20	17/03/20	27	M	MG	Betim	..
CV33	1420/20	SWAB	18.79	14/03/20	17/03/20	35	M	MG	Sabara	..
CV34	1467/20	SWAB	22.31	16/03/20	18/03/20	48	F	MG	Belo Horizonte	..
CV35	1500/20	SWAB	24.06	07/03/20	18/03/20	75	M	MG	Poços De Caldas	Chile, Peru
CV36	1504/20	SWAB	24.07	18/03/20	18/03/20	50	F	MG	Muriae	..
CV40	1834/20	SWAB	23.97	18/03/20	19/03/20	29	M	MG	Belo Horizonte	..
CV41	1892/20	SWAB	22.84	16/03/20	18/03/20	20	F	MG	Serra Do Salitre	..
CV42	2119/20	SWAB	18.78	18/03/20	20/03/20	67	M	MG	São João Del Rei	..
CV43	2159/20	SWAB	24.81	14/03/20	17/03/20	19	F	MG	Patrocinio	..
CV44	2196/20	SWAB	23.47	17/03/20	18/03/20	19	F	MG	Patrocinio	..
CV45	2241/20	SWAB	22.85	14/03/20	20/03/20	58	M	MG	Muriae	Sao Paulo (Brazil)
CV46	2271/20	SWAB	22.9	19/03/20	20/03/20	35	M	MG	Belo Horizonte	..
CV47	2288/20	SWAB	22.4	17/03/20	19/03/20	35	M	MG	Belo Horizonte	..
CV48	2693/20	SWAB	22.43	19/03/20	20/03/20	74	M	MG	Varginha	..
CV49	2801/20	SWAB	20.95	16/03/20	22/03/20	30	M	MG	Belo Horizonte	..
CV50	5068/20	SWAB	31.86	20/03/20	26/03/20	44	M	MG	Mariana	..

Project-ID=sample identifier; Onset date= Symptoms onset date; F=Female; M=Male; MG=State of Minas Gerais; NA=Not Available.

Of the 40 samples, 17 (42.5%) were from the state's capital (Belo Horizonte), while the other municipalities were represented by one or a maximum of three samples. These samples were from 17 females and 23 males, with collection dates ranging from 4 March 2020 to 26 March 2020 (Table 1). The median age of the patients was 35 years (ranging from 19-79 years old). The first sample that tested positive by RT-qPCR at IOM/FUNED was collected on 4th March 2020 (Table 1 and Figure 1). Selected samples had cycle threshold (Ct) values that ranged from 16.41 to 31.86 (median=22.945). We found no demographic variables (age, gender) to be statistically correlated with sample Ct (Supplementary Figure S17). The new sequences had a median genome coverage of 82.5% related to the reference genome NC_045512.3 (S1 Table). All sequences generated in this study have been submitted to the GISAID following WHO guidelines [38].

Of the 17 (42.5%, n=40) sequenced cases with available travel history information, 14 (82.35%, n=17) reported international travel while three reported domestic travel (Table 1). Two among the later visited the city of São Paulo and one the city of Rio de Janeiro. Of the international travel-related cases, seven (50%) were linked to travel to European countries (Portugal, Spain, Italy, Switzerland, Austria, England, Belgium, Germany, Czech Republic, and Hungary), while six reported travel to countries in the Americas (USA, Colombia, Jamaica, Cayman Islands, Panama, Chile, and Peru). One case reported travel to Israel.

**Figure 3.**
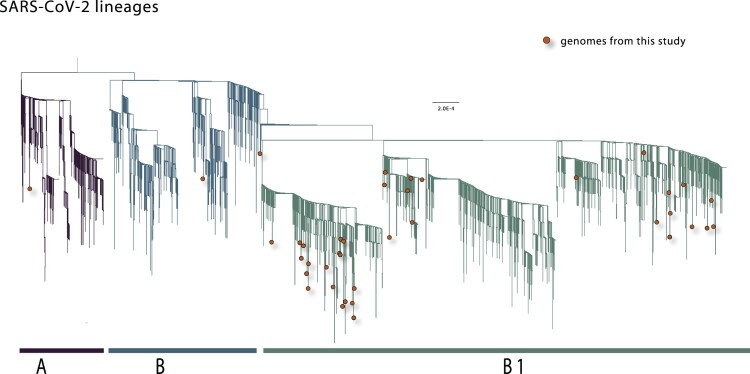
Phylogenetic analysis of the SARS-CoV-2 isolated in the state of MG, Brazil. Maximum likelihood phylogeny inferred using 40 genome sequences from SARS-CoV-2 generated in this study (marked with red circles) and 3062 sequences already deposited on the GISAID database. The tree is rooted between lineages A and B. Vertical branch lengths give the number of nucleotide substitutions per site.

To explore the epidemiological history of the virus in MG, we performed a maximum likelihood (ML) phylogenetic analysis on the 40 new sequences combined with another 3,062 sequences deposited in GISAID up to 15 April 2020. We also made use of a publicly available tool for the automated lineage assignment within SARS-CoV-2 (https://github.com/hCoV-2019/pangolin) [23]. Our estimated phylogeny identified two major clades branching at the root of the tree (Figure 3). These two clades were named lineages A and B, following a recently proposed SARS-CoV-2 lineage nomenclature [39]: lineage A can be defined by the Wuhan/WH04/2020 sequence, while lineage B is represented by Wuhan-Hu-1. Lineage B can be further divided into a number of component sublineages, and our phylogenetic analysis revealed that the majority (n=37, 92.5%) of MG sequences belonged to lineage B.1 which contains sequences from a variety of countries including Australia, China, Canada, Malaysia, and the USA [40] (Figure 3, Table 1). Of these 37 B.1 sequences, 11 were isolates from cases that reported travel to European countries (CV2, CV3, CV11, CV13, CV17) or the Americas (CV4, CV6, CV12, CV20, CV28, CV35), in addition to the isolate CV1 from a traveller who returned from Israel. Aside from sublineage B.1, two sequences were assigned to lineage B in our ML phylogeny (isolates CV22 and CV36, with the former reporting travel to Germany), while one sequence could be assigned to lineage A (isolate CV7) who reported travel to European countries (full results from the Pangolin COVID-19 lineage assigner are presented in Supplementary Table S2).

**Figure 4.**
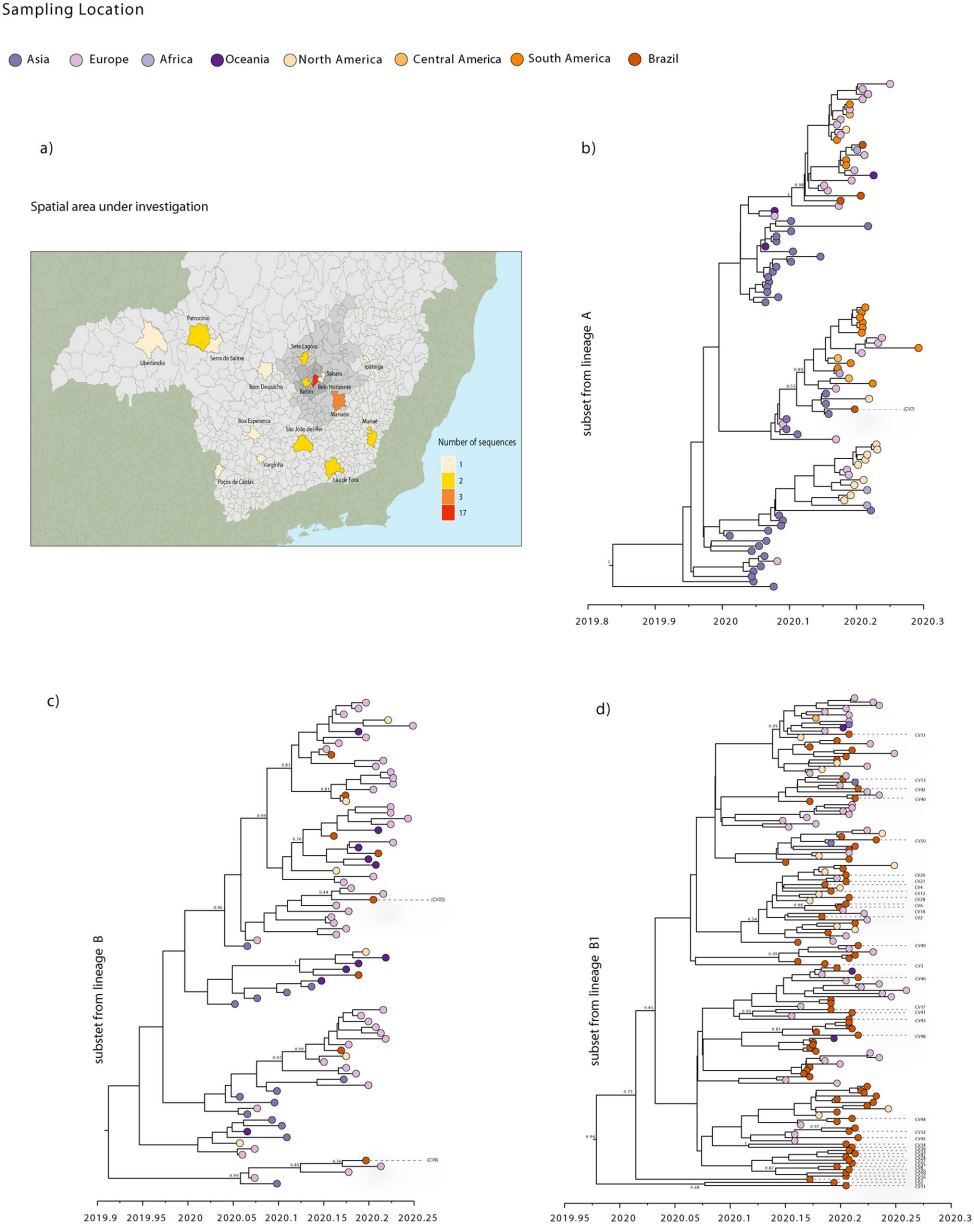
Bayesian analysis of the SARS-CoV-2 isolated in the state of MG, Brazil. (a) Map of the MG state showing the number of SARS-CoV-2 new sequences by patient´s municipality. (b) Molecular clock phylogeny of a subset of lineage A viruses, including one new sequence from MG. (c) Molecular clock phylogeny of a subset of lineage B viruses, including two new sequences from MG. (d) Molecular clock phylogeny of the subset from sublineage B.1, including 37 new sequences from MG. In all cases, the numbers along branches represent posterior probabilities while colours represent different sampling locations.

To assess the evolution of these lineages in more detail and in time, we performed Bayesian time-measured phylogenetic analysis using a molecular clock model. We analysed three subsets of data (A, B, B.1) extracted from each lineage from the ML tree that included Brazilian sequences. Our maximum clade credibility (MCC) trees showed that most of MG's sequences were interspersed with viruses sampled from other countries (Figure 4b, c, d). This pattern, similar to that observed elsewhere [40–42], is also in accordance with our ML tree and with the epidemiological data, indicating that the appearance of these viruses were linked to travel exposure rather than community transmission, and reinforcing the idea that multiple independent introductions from overseas occurred in MG.

In contrast, some other SARS-CoV-2 sequences from MG grouped together, forming clusters that included sequences from Brazil and other countries: these are apparent in the data subset B.1 phylogeny (Figure 4d). However, these clusters have very low posterior probability support, likely due to the low genetic diversity of SARS-CoV-2 genomes [43–45]. Nonetheless, four clusters, each comprising only two MG sequences, showed posterior probabilities >80%. One of these clusters (Figure 4d), with a posterior probability of 100%, was formed by isolates CV34 and CV36 that arose from local contacts with a confirmed and a suspected COVID-19 case, respectively.

From the time-scaled phylogenies, we estimated the mean time of the most recent common ancestor (tMRCA) of the SARS-CoV-2 epidemic in Brazil to range from 27 January to 22 February 2020 (95% HPD interval; mean date of 10 February 2020). This is consistent with the start of reported cases in Brazil and with the epidemiological data from the first case confirmed in SP, regarding a traveller returning from Lombardy, Italy, on 21 February 2020 [11,13].

## Discussion

We provide a preliminary characterization of the ongoing COVID-19 epidemic in MG, the second largest state in Brazil with respect to population size, where 1,578 cases were reported up to 28 April 2020. We report genomic data obtained by sequencing 40 SARS-CoV-2 infection cases confirmed by RT-qPCR in MG. We also collected epidemic data of case and death time series to estimate key epidemiological metrics such as the case fatality ratio, the observation rate and the effective reproduction number, R. Combined epidemiological and genomic data strongly suggest that the epidemic of SARS-CoV-2 infection in MG was associated with multiple independent importations from returned travellers.

Epidemiological analysis using case and death time series revealed that in MG, where case incidence was ∼7.85, the distribution of cases and deaths was more spatially uniform (i.e. less focused on Belo Horizonte), while in SP and RJ states it was more centralized around capital cities, although with a wide variance. The estimated observation rate was 15.3% in MG, indicating that 1 case in 7 was reported in that state by 28 April 2020. Despite the different approaches used, the R values estimated for MG were similar to those estimated previously [46]. As all estimates of R were > 1, we conclude that virus transmission is not controlled and that the epidemic in MG will continue to grow unless stricter non-pharmaceutical interventions are implemented.

Our time-measured phylogeny revealed that MG's isolates are scattered throughout the tree, although some clusters suggestive of local transmission were also observed. This dispersed distribution has been observed in other studies such as from China [42] and New York [47], and is consistent with the self-declared travel history of patients to different countries in the Americas and Europe. From our phylogeny we inferred that multiple independent importations of SARS-CoV-2 infection from returned travellers had occurred in MG. This is noteworthy because it has been argued that several introductions are necessary for an outbreak to establish in a new location [48]. The mean time of the most recent common ancestor of all Brazilian strains analysed in this study was estimated to be from 27 January to 22 February 2020, and range that includes the first COVID-19 confirmed case reported in 21 February 2020 [49]. These results illustrate that by combining genomic data and available epidemiological information we can generate more reliable phylogenetic inferences.

Despite the grouping of some MG sequences, because of the small sample size data which covers only about 30 days of the MG epidemic we cannot infer a close relationship between these sequences with certainty at this stage. In addition, the low genetic diversity of sequences available limits conclusions about SARS-CoV-2 directionality and spread based on genetic data alone. As noted elsewhere [43], such phylogenetic results should be approached with caution and considered as hypothesis-generating with respect to the transmission events of SARS-CoV-2 in a local setting.

In conclusion, at the end of April 2020, the COVID-19 epidemic in the state of MG was expanding (R>1) and highly geographically dispersed, with many cases and deaths reported away from the capital city. Genomic data and other epidemiological information from travel-related cases allowed us to identify several independent introductions in MG, helping to explain the geographical patchiness of reported cases and deaths. These initial insights based on the restricted data reveal that transmission is likely to continue in the near future and suggest there is considerable room to improve reporting. Increasing COVID-19 testing and SARS-CoV-2 genomic sequencing will undoubtedly help to understand how the virus is spreading in Brazil and thus greatly assist epidemic control.

## Supplementary Material

Supplementary_Table_S3_final.docx

Supplementary_Material.docx

## Data Availability

SARS-CoV-2 genome sequences generated in this study have been deposited in the GISAID platform (https://www.gisaid.org/), accession numbers IDs EPI_ISL_429664 to EPI_ISL_429703.
