# Supplementary material for: The ongoing COVID-19 epidemic in Minas Gerais, Brazil: insights from epidemiological data and SARS-CoV-2 whole genome sequencing
Source: Emerg Microbes Infect. 2020 Aug 11;9(1):1824–34. doi: 10.1080/22221751.2020.1803146 (PMC7473129; doi:10.1080/22221751.2020.1803146)
Supplement: Supplementary_Table_S3_final.docx [file TEMI_A_1803146_SM3076.docx]

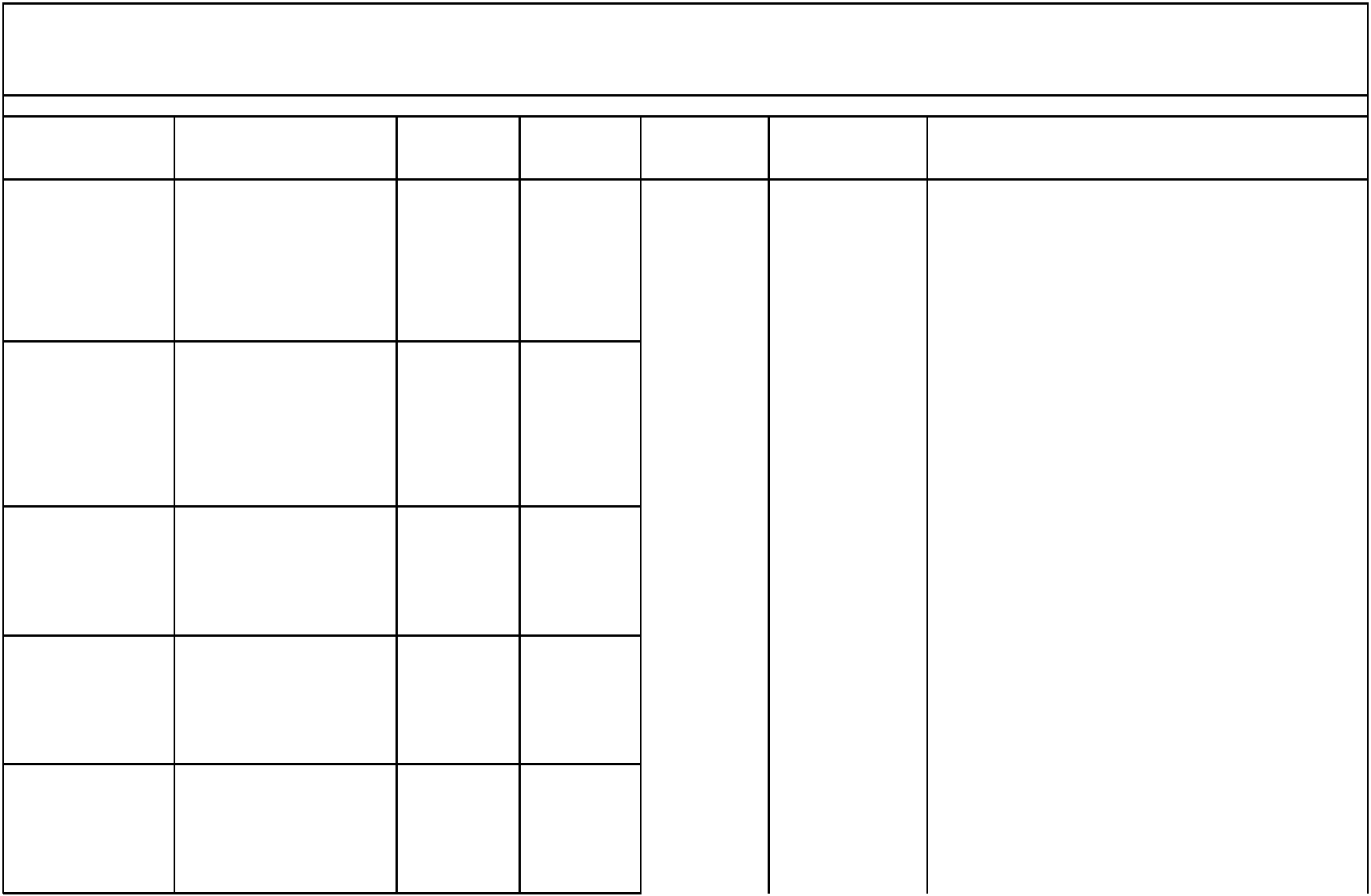


**We gratefully acknowledge the following Authors from the Originating laboratories responsible for obtaining the specimens and the Submitting laboratories where genetic sequence data were generated and shared via the GISAID Initiative, on which this research is based.**

**All submitters of data may be contacted directly via www.gisaid.org**

**Accession ID** **Virus name** **Location** **Collection date Originating lab** **Submitting lab** **Authors**

EPI_ISL_412964

EPI_ISL_413016

EPI_ISL_414014

EPI_ISL_414015

EPI_ISL_414016

hCoV-19/Brazil/SPBR-01/2020

hCoV-19/Brazil/SPBR-02/2020

hCoV-19/Brazil/SPBR-03/2020

hCoV-19/Brazil/SPBR-06/2020

hCoV-19/Brazil/SPBR-05/2020

South America / Brazil / Sao Paulo / Sao Paulo

South America / Brazil / Sao Paulo / Sao Paulo

South America / Brazil / Sao Paulo

South America / Brazil / Sao Paulo / Sao Paulo

South America / Brazil / Sao Paulo / Sao Paulo

2020-02-25

2020-02-28

2020-03-02

2020-02-29

2020-02-29

|  |  |  | Jaqueline Goes de Jesus, Claudio Tavares Sacchi, Daniela |  |
| --- | --- | --- | --- | --- |
|  |  | Instituto Adolfo Lutz | Bernardes Borges da Silva, Ingra Morales Claro, Flávia |  |
| Hospital Israelita |  | Interdisciplinary | Cristina da Silva Sales, Claudia Regina Gonçalves, Joshua |  |
| Albert Einstein |  | Procedures Center | Quick, Maria do Carmo, Sampaio Tavares Timenetsky, |  |
|  |  | Strategic Laboratory | Nicholas James Loman, Andrew Rambaut, Ester Cerdeira |  |
|  |  |  | Sabino, Nuno Rodrigues Faria |  |
|  |  |  |  |  |
|  |  |  | Jaqueline Goes de Jesus, Claudio Tavares Sacchi, Fabiana |  |
|  |  | Instituto Adolfo Lutz, | Cristina Pereira dos Santos, Ingra Morales Claro, Flávia |  |
| Hospital Israelita |  | Interdisciplinary | Cristina da Silva Sales, Claudia Regina Gonçalves, Joshua |  |
| Albert Einstein |  | Procedures Center, | Quick, Maria do Carmo Sampaio Tavares Timenetsky, |  |
|  |  | Strategic Laboratory | Nicholas James Loman, Andrew Rambaut, Ester Cerdeira |  |
|  |  |  | Sabino, Nuno Rodrigues Faria |  |
|  |  |  |  |  |
|  |  | Instituto Adolfo Lutz, | Claudio Tavares Sacchi, Claudia Regina Gonçalves, Katia |  |
| Hospital Israelita |  | Interdiciplinary | Correia dos Santos, Carlos Henrique Camargo, Maria do |  |
| Albert Einstein |  | Procedures Center, | Carmo Sampaito Tavares Timenetsky, Terezinha Maria de |  |
|  |  | Strategic Laboratory | Paiva, Ester Cerdeira Sabino |  |
|  |  |  |  |  |
| Hospital São |  | Instituto Adolfo Lutz, | Claudio Tavares Sacchi, Claudia Regina Gonçalves, |  |
|  |  |  | SimoneGuadagnucci Morillo, Carlos Henrique Camargo, |  |
| Joaquim |  | Interdiciplinary |  |  |
|  |  |  | Maria do Carmo Sampaito Tavares Timenetsky, Fabiana |  |
| Beneficencia |  | Procedures Center, |  |  |
|  |  |  | Cristina Pereira dos Santos Terezinha Maria de Paiva, Ester |  |
| Portuguesa |  | Strategic Laboratory |  |  |
|  |  |  | Cerdeira Sabino |  |
|  |  |  |  |  |
|  |  |  |  |  |
| Hospital São |  | Instituto Adolfo Lutz, | Claudio Tavares Sacchi, Claudia Regina Gonçalves, Audrey |  |
| Joaquim |  | Interdiciplinary | Cilli, Carlos Henrique Camargo, Maria do Carmo Sampaito |  |
| Beneficencia |  | Procedures Center, | Tavares Timenetsky, Daniela Bernardes Borges da Silva, |  |
| Portuguesa |  | Strategic Laboratory | Terezinha Maria de Paiva, Ester Cerdeira Sabino |  |
|  |  |  |  |  |

EPI_ISL_414017

EPI_ISL_414045

EPI_ISL_415105

EPI_ISL_415128

EPI_ISL_416028

EPI_ISL_416029

EPI_ISL_416031

hCoV-19/Brazil/SPBR-04/2020

hCoV-19/Brazil/RJ-314/2020

hCoV-19/Brazil/BA-312/2020

hCoV-19/Brazil/ES-225/2020

hCoV-19/Brazil/SPBR-07/2020

hCoV-19/Brazil/SPBR-08/2020

hCoV-19/Brazil/SPBR-09/2020

South America / Brazil / Sao Paulo

South America / Brazil / Rio de Janeiro / Rio de Janeiro

South America / Brazil / Bahia / Feira de Santana

South America / Brazil / Espirito Santo / Vila Velha

South America / Brazil / Sao Paulo / Sao Paulo

South America / Brazil / Sao Paulo / Sao Paulo

South America / Brazil / Sao Paulo / Sao Paulo

2020-03-04

2020-03-04

2020-03-04

2020-02-29

2020-03-03

2020-03-04

2020-03-04

Hospital São Joaquim Beneficencia Portuguesa


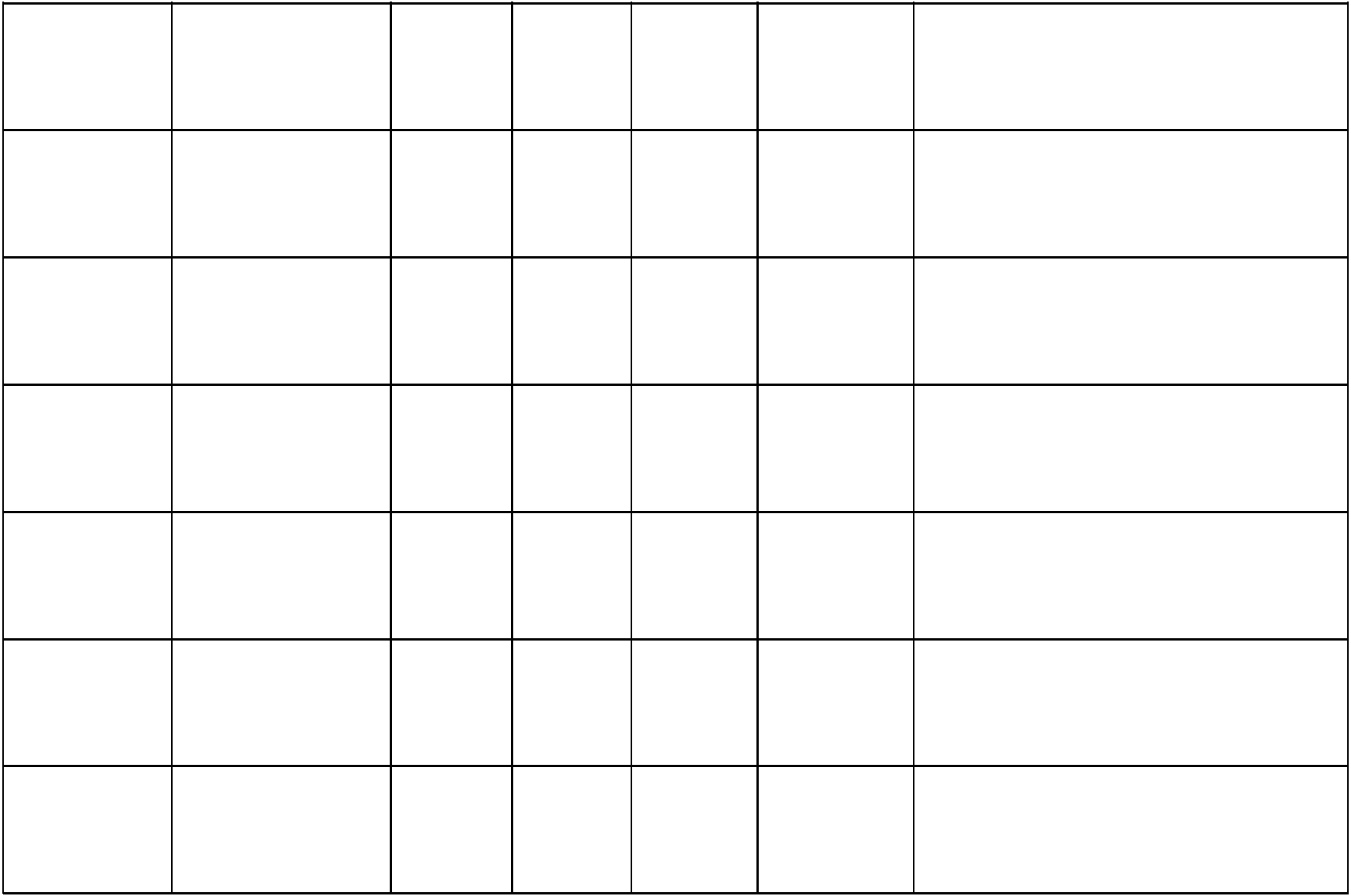


LACEN RJ - Laboratório Central de Saúde Pública Noel Nutels

Laboratório

Central de

Saúde Pública

Professor

Gonçalo Moniz

– LACEN/BA

LACEN/ES -

Laboratório Central de Saúde Pública do Espírito Santo

National

Influenza Center

- Instituto Adolfo Lutz

Laboiratório

Fleury

National

Influenza Center

- Instituto Adolfo Lutz

Instituto Adolfo Lutz, Interdiciplinary Procedures Center, Strategic Laboratory

Instituto Oswaldo

Cruz FIOCRUZ -

Laboratory of

Respiratory Viruses

and Measles (LVRS)

Instituto Oswaldo

Cruz FIOCRUZ -

Laboratory of

Respiratory Viruses

and Measles (LVRS)

Instituto Oswaldo

Cruz FIOCRUZ -

Laboratory of

Respiratory Viruses

and Measles (LVRS)

Instituto Adolfo Lutz, Interdiciplinary Procedures Center, Strategic Laboratory

Instituto Adolfo Lutz, Interdiciplinary Procedures Center, Strategic Laboratory

Instituto Adolfo Lutz, Interdiciplinary Procedures Center, Strategic Laboratory

Claudio Tavares Sacchi, Claudia Regina Gonçalves, Fabiana

Cristina Pereira dos Santos, Carlos Henrique Camargo, Maria do Carmo Sampaito Tavares Timenetsky, Daniela Bernardes Borges da Silva, Terezinha Maria de Paiva, Ester Cerdeira Sabino

Paola Resende, Alisson Fabri, Joilson Xavier, Sunando Roy, Fernando Motta, Aline Mattos, Milene Miranda, Cristiana Garcia, Braulia Caetano, Maria Ogrzewalska, Jonathan Lopes, Luciana Appolinario, Maria Nóbrega, Marilda Siqueira

Paola Resende, Allison Fabri, Joilson Xavier, Sunando Roy, Fernando Motta, Aline Mattos, Milene Miranda, Cristiana Garcia, Braulia Caetano, Maria Ogrzewalska, Jonathan Lopes, Luciana Appolinario, Maria Nóbrega, Marilda Siqueira

Paola Resende, Allison Fabri, Joilson Xavier, Sunando Roy, Fernando Motta, Aline Mattos, Milene Miranda, Cristiana Garcia, Braulia Caetano, Maria Ogrzewalska, Jonathan Lopes, Luciana Appolinario, Maria Nóbrega, Marilda Siqueira

Claudio Tavares Sacchi, Claudia Regina Gonçalves, Carlos Henrique Camargo, Fabiana Cristina Pereira dos Santos, Daniela Bernardes Borges da Silva, Simone Guadagnucci Morillo, Adriano Abbud, Adriana Bugno, Maria do Carmo Sampaio Tavares Timenetsky, Terezinha Maria de Paiva

Claudio Tavares Sacchi, Claudia Regina Gonçalves, Carlos Henrique Camargo, Fabiana Cristina Pereira dos Santos, Daniela Bernardes Borges da Silva, Simone Guadagnucci Morillo, Adriano Abbud, Adriana Bugno, Maria do Carmo Sampaio Tavares Timenetsky, Terezinha Maria de Paiva

Claudio Tavares Sacchi, Claudia Regina Gonçalves, Carlos Henrique Camargo, Fabiana Cristina Pereira dos Santos, Daniela Bernardes Borges da Silva, Simone Guadagnucci Morillo, Adriano Abbud, Adriana Bugno, Maria do Carmo Sampaio Tavares Timenetsky, Terezinha Maria de Paiva

EPI_ISL_416032

EPI_ISL_416033

EPI_ISL_416034

hCoV-19/Brazil/SPBR-10/2020

hCoV-19/Brazil/SPBR-11/2020

hCoV-19/Brazil/SPBR-12/2020

South America /

Brazil / Distrito

Federal /

Brasilia

South America / Brazil / Sao Paulo / Sao Paulo

South America / Brazil / Sao Paulo / Sao Paulo

|  | National |  | Instituto Adolfo Lutz, |  |
| --- | --- | --- | --- | --- |
| 2020-03-04 | Influenza Center |  | Interdiciplinary |  |
|  | - Instituto Adolfo |  | Procedures Center, |  |
|  |  |  |  |  |
|  | Lutz |  | Strategic Laboratory |  |
|  |  |  |  |  |
|  | Hospital Israelita |  | Instituto Adolfo Lutz, |  |
| 2020-03-03 |  |  | Interdiciplinary |  |
|  | Albert Einstein |  | Procedures Center, |  |
|  |  |  |  |  |
|  |  |  | Strategic Laboratory |  |
|  |  |  |  |  |
|  | Hospital Israelita |  | Instituto Adolfo Lutz, |  |
| 2020-03-04 |  |  | Interdiciplinary |  |
|  | Albert Einstein |  | Procedures Center, |  |
|  |  |  |  |  |


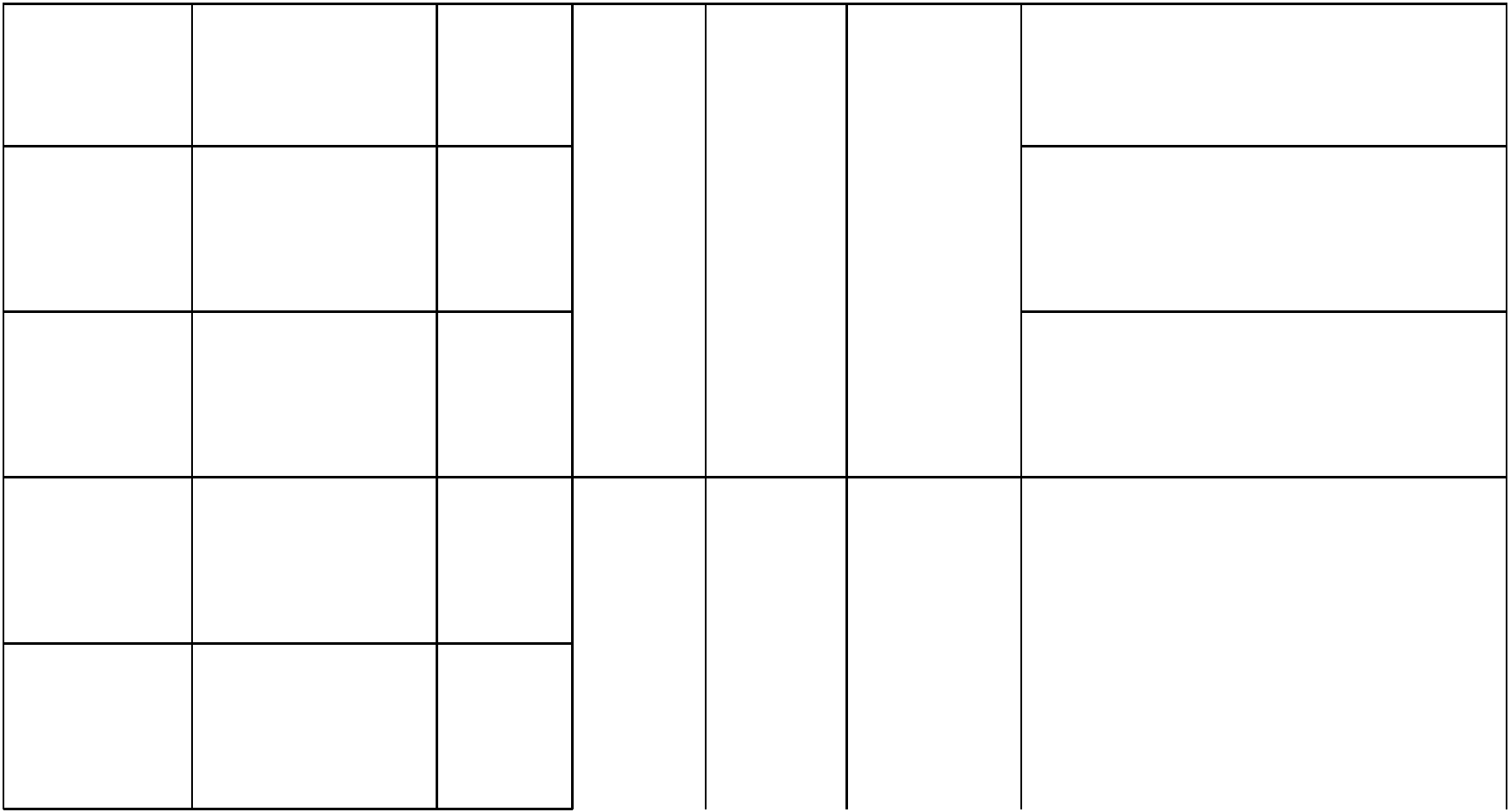


Strategic Laboratory

Claudio Tavares Sacchi, Claudia Regina Gonçalves, Carlos Henrique Camargo, Fabiana Cristina Pereira dos Santos, Daniela Bernardes Borges da Silva, Simone Guadagnucci Morillo, Adriano Abbud, Adriana Bugno, Maria do Carmo Sampaio Tavares Timenetsky, Terezinha Maria de Paiva

Claudio Tavares Sacchi, Claudia Regina Gonçalves, Carlos Henrique Camargo, Erica Valessa Ramos Gomes, Fabiana Cristina Pereira dos Santos, Daniela Bernardes Borges da Silva, Simone Guadagnucci Morillo, Adriano Abbud, Adriana Bugno, Maria do Carmo Sampaio Tavares Timenetsky, Terezinha Maria de Paiva

Claudio Tavares Sacchi, Claudia Regina Gonçalves, Carlos Henrique Camargo, Erica Valessa Ramos Gomes, Fabiana Cristina Pereira dos Santos, Daniela Bernardes Borges da Silva, Simone Guadagnucci Morillo, Adriano Abbud, Adriana Bugno, Maria do Carmo Sampaio Tavares Timenetsky, Terezinha Maria de Paiva

EPI_ISL_416035

EPI_ISL_416036

hCoV-19/Brazil/SPBR-13/2020

hCoV-19/Brazil/SPBR-14/2020

South America / Brazil / Sao Paulo / Sao Paulo

South America / Brazil / Sao Paulo / Sao Paulo

|  |  |  |  | Claudio Tavares Sacchi, Claudia Regina Gonçalves, Carlos |  |
| --- | --- | --- | --- | --- | --- |
|  | National |  | Instituto Adolfo Lutz, | Henrique Camargo, Erica Valessa Ramos Gomes, Fabiana |  |
| 2020-03-05 | Influenza Center |  | Interdiciplinary | Cristina Pereira dos Santos, Daniela Bernardes Borges da |  |
|  | - Instituto Adolfo |  | Procedures Center, | Silva, Simone Guadagnucci Morillo, Adriano Abbud, Adriana |  |
|  |  |  |  |  |  |
|  | Lutz |  | Strategic Laboratory | Bugno, Maria do Carmo Sampaio Tavares Timenetsky, |  |
|  |  |  |  | Terezinha Maria de Paiva |  |
|  |  |  |  |  |  |
|  |  |  |  | Claudio Tavares Sacchi, Claudia Regina Gonçalves, Carlos |  |
|  | National |  | Instituto Adolfo Lutz, | Henrique Camargo, Erica Valessa Ramos Gomes, Fabiana |  |
| 2020-03-05 | Influenza Center |  | Interdiciplinary | Cristina Pereira dos Santos, Daniela Bernardes Borges da |  |
|  | - Instituto Adolfo |  | Procedures Center, | Silva, Simone Guadagnucci Morillo, Adriano Abbud, Adriana |  |
|  |  |  |  |  |  |
|  | Lutz |  | Strategic Laboratory | Bugno, Maria do Carmo Sampaio Tavares Timenetsky, |  |
|  |  |  |  | Terezinha Maria de Paiva |  |
|  |  |  |  |  |  |

|  |  |  |  | South America / |  |
| --- | --- | --- | --- | --- | --- |
| EPI_ISL_417034 |  | hCoV-19/Brazil/AMBR- |  | Brazil / |  |
|  | 02/2020 | |  | Amazonas |  |
|  |  |  |  |  |  |
|  |  |  |  | State / Manaus |  |
|  |  |  |  |  |  |

South America /

EPI_ISL_417925 hCoV-19/Brazil/MG0101/2020 Brazil / Minas

Gerais

South America /

EPI_ISL_417926 hCoV-19/Brazil/MG0102/2020 Brazil / Minas

Gerais

2020-03-16

2020-03-17

2020-03-18

Laboratorio de Ecologia de Doencas


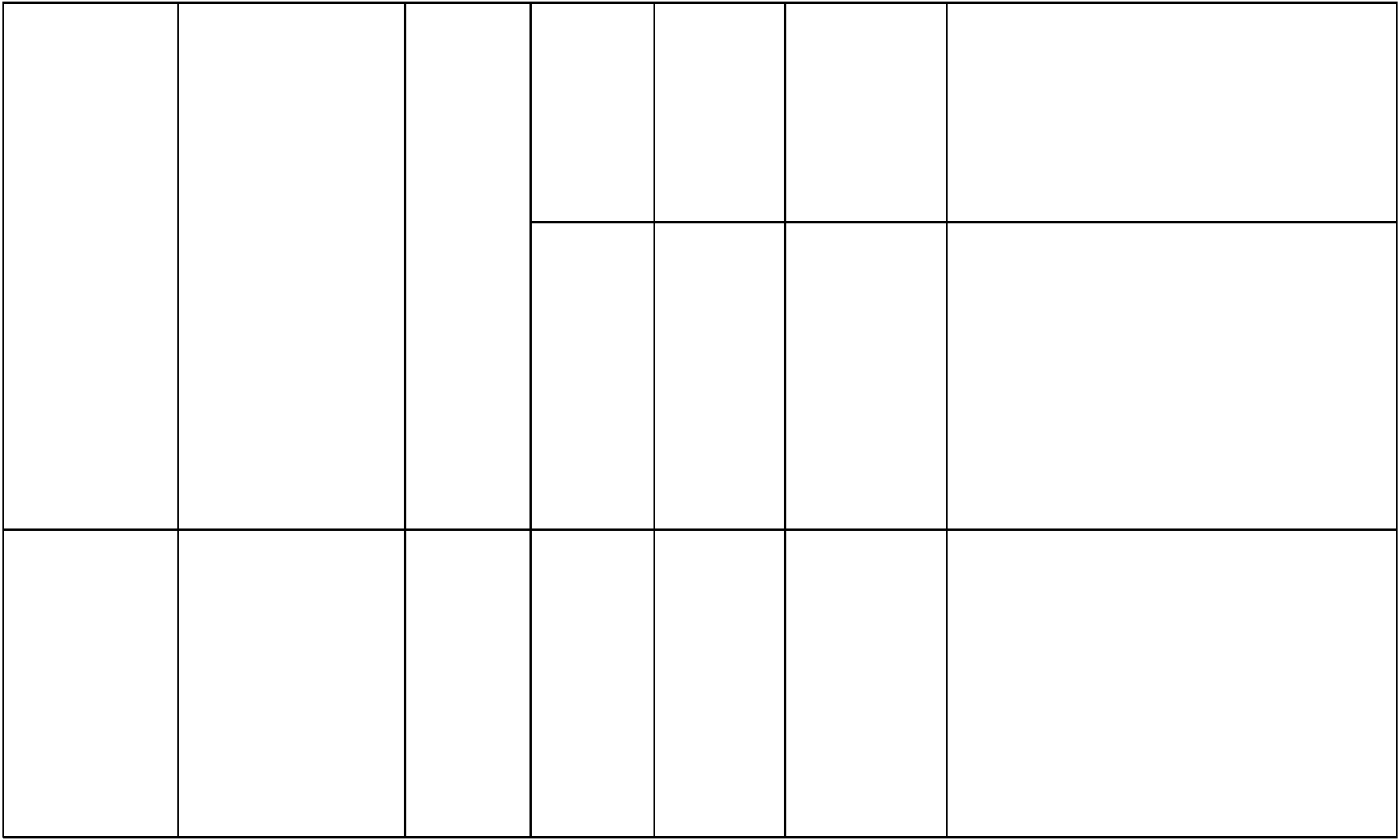


Transmissiveis

na Amazonia,

Instituto

Leonidas e

Maria Deane -

Fiocruz

Amazonia

Laboratório Simili

Laboratório Simili

Laboratorio de Ecologia de Doencas

Transmissiveis na Amazonia, Instituto Leonidas e Maria Deane - Fiocruz Amazonia

Bioinformatics Laboratory / LNCC

Bioinformatics Laboratory / LNCC

Valdinete Nascimento, André Corado, Fernanda Nascimento, Ágatha Costa, Debora Duarte, Luciana Gonçalves, Michele Jesus, Sérgio Luz, Felipe Naveca

Filipe Romero, Ana Paula Guimarães, Mariane Talon, Luiz Gonzaga Paula de Almeida, Ronaldo da Silva, Francisco Junior, Diana Mariani, Lídia Boullosa, Alexandra Gerber, Jaqueline Goes de Jesus, Ingra Morales Claro, Ester Cerdeira Sabino, Nuno Rodrigues Faria, Terezinha Marta Pereira, Pinto Castiñeiras, Isabela de Carvalho Leitão, Rafael de Mello Galliez, Cássia Cristina Alves Gonçalves, Érica Ramos dos Santos Nascimento, Richard Araújo Maia, Mauro Teixeira,Cristiano Xavier Lima, Orlando Ferreira Jr., Rodrigo Brindeiro, Luciana Jesus Costa e André Felipe Santos, Laboratorio Hermes Pardini, Laboratorio Simile, Amilcar Tanuri, Renato Santana Aguiar e Ana Tereza Vasconcelos

Filipe Romero, Ana Paula Guimarães, Mariane Talon, Luiz Gonzaga Paula de Almeida, Ronaldo da Silva Francisco Junior, Diana Mariani, Lídia Boullosa, Alexandra Gerber, Jaqueline Goes de Jesus, Ingra Morales Claro, Ester Cerdeira Sabino, Nuno Rodrigues Faria, Terezinha Marta Pereira, Pinto Castiñeiras, Isabela de Carvalho Leitão, Rafael de Mello Galliez, Cássia Cristina Alves Gonçalves, Érica Ramos dos Santos Nascimento, Richard Araújo Maia, Mauro Teixeira,Cristiano Xavier Lima, Orlando Ferreira Jr., Rodrigo Brindeiro, Luciana Jesus Costa e André Felipe Santos, Laboratorio Hermes Pardini, Laboratorio Simile, Amilcar Tanuri, Renato Santana Aguiar e Ana Tereza Vasconcelos

EPI_ISL_417928

EPI_ISL_417929

EPI_ISL_417930

hCoV-19/Brazil/SP0104/2020

hCoV-19/Brazil/SP0105/2020

hCoV-19/Brazil/GO0106/2020

South America / Brazil / São Paulo

South America / Brazil / São Paulo

South America / Brazil / Goiais

|  |  |  |  | Filipe Romero, Ana Paula Guimarães, Mariane Talon, Luiz |  |
| --- | --- | --- | --- | --- | --- |
|  |  |  |  | Gonzaga Paula de Almeida, Ronaldo da Silva Francisco |  |
|  |  |  |  | Junior, Diana Mariani, Lídia Boullosa, Alexandra Gerber, |  |
|  |  |  |  | Jaqueline Goes de Jesus, Ingra Morales Claro, Ester |  |
|  |  |  |  | Cerdeira Sabino, Nuno Rodrigues Faria, Terezinha Marta |  |
| 2020-03-13 | Laboratório |  | Bioinformatics | Pereira, Pinto Castiñeiras, Isabela de Carvalho Leitão, Rafael |  |
|  | Hermes Pardini |  | Laboratory - LNCC | de Mello Galliez, Cássia Cristina Alves Gonçalves, Érica |  |
|  |  |  |  |  |  |
|  |  |  |  | Ramos dos Santos Nascimento, Richard Araújo Maia, Mauro |  |
|  |  |  |  | Teixeira,Cristiano Xavier Lima, Orlando Ferreira Jr., Rodrigo |  |
|  |  |  |  | Brindeiro, Luciana Jesus Costa e André Felipe Santos, |  |
|  |  |  |  | Laboratorio Hermes Pardini, Laboratorio Simile, Amilcar |  |
|  |  |  |  | Tanuri, Renato Santana Aguiar e Ana Tereza Vasconcelos |  |
|  |  |  |  |  |  |
|  |  |  |  | Filipe Romero, Ana Paula Guimarães, Mariane Talon, Luiz |  |
|  |  |  |  | Gonzaga Paula de Almeida, Ronaldo da Silva Francisco |  |
|  |  |  |  | Junior, Diana Mariani, Lídia Boullosa, Alexandra Gerber, |  |
|  |  |  |  | Jaqueline Goes de Jesus, Ingra Morales Claro, Ester |  |
|  |  |  |  | Cerdeira Sabino, Nuno Rodrigues Faria, Terezinha Marta |  |
| 2020-03-13 | Laboratório |  | Bioinformatics | Pereira, Pinto Castiñeiras, Isabela de Carvalho Leitão, Rafael |  |
|  | Hermes Pardini |  | Laboratory - LNCC | de Mello Galliez, Cássia Cristina Alves Gonçalves, Érica |  |
|  |  |  |  |  |  |
|  |  |  |  | Ramos dos Santos Nascimento, Richard Araújo Maia, Mauro |  |
|  |  |  |  | Teixeira,Cristiano Xavier Lima, Orlando Ferreira Jr., Rodrigo |  |
|  |  |  |  | Brindeiro, Luciana Jesus Costa e André Felipe Santos, |  |
|  |  |  |  | Laboratorio Hermes Pardini, Laboratorio Simile, Amilcar |  |
|  |  |  |  | Tanuri, Renato Santana Aguiar e Ana Tereza Vasconcelos |  |
|  |  |  |  |  |  |
|  |  |  |  | Filipe Romero, Ana Paula Guimarães, Mariane Talon, Luiz |  |
|  |  |  |  | Gonzaga Paula de Almeida, Ronaldo da Silva Francisco |  |
|  |  |  |  | Junior, Diana Mariani, Lídia Boullosa, Alexandra Gerber, |  |
|  |  |  |  | Jaqueline Goes de Jesus, Ingra Morales Claro, Ester |  |
|  | Laboratório |  | Bioinformatics | Cerdeira Sabino, Nuno Rodrigues Faria, Terezinha Marta |  |
| 2020-03-13 |  |  |  | Pereira, Pinto Castiñeiras, Isabela de Carvalho Leitão, Rafael |  |
|  | Hermes Pardini |  | Laboratory - LNCC | de Mello Galliez, Cássia Cristina Alves Gonçalves, Érica |  |
|  |  |  |  |  |  |


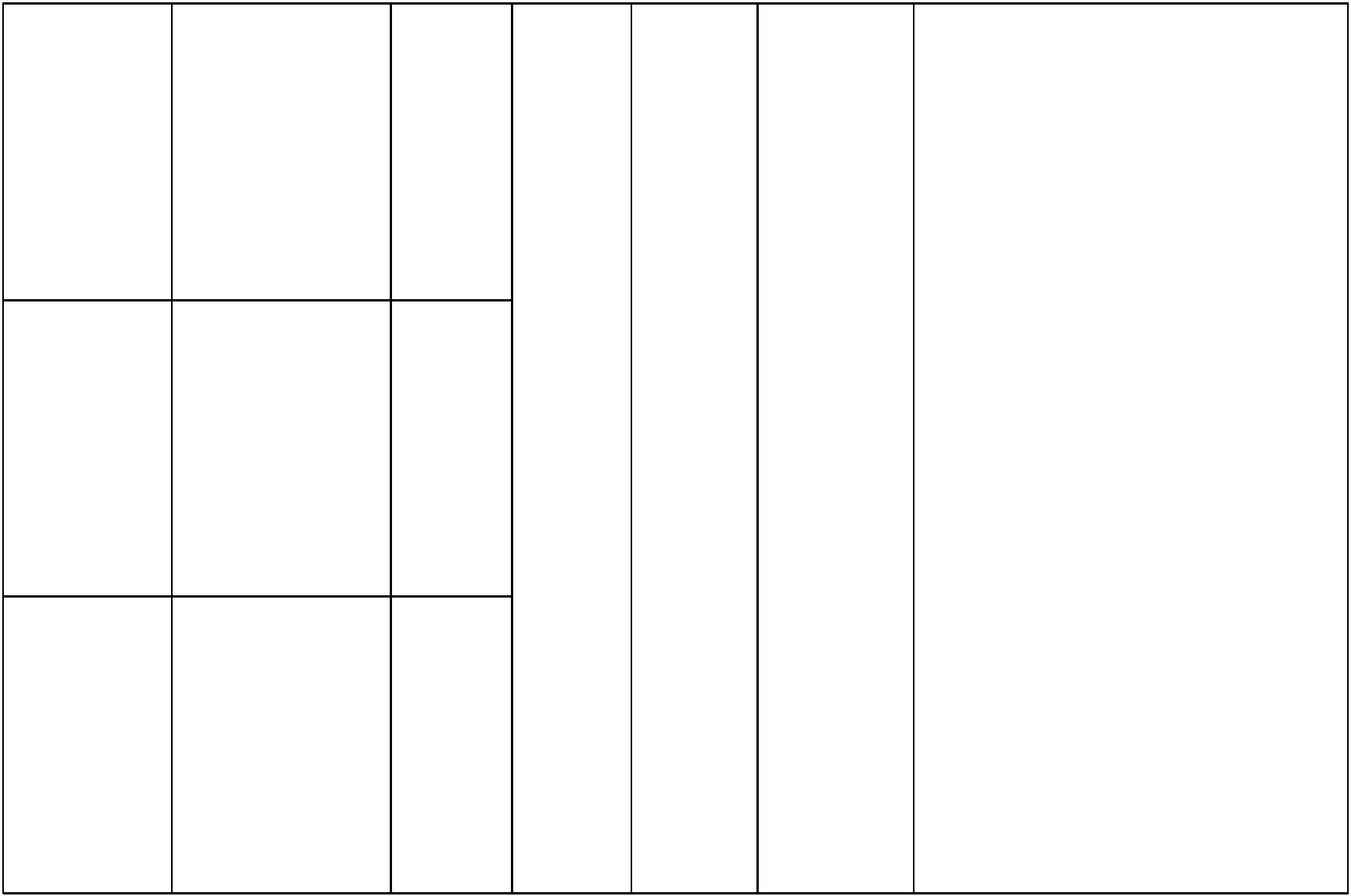


Ramos dos Santos Nascimento, Richard Araújo Maia, Mauro

Teixeira,Cristiano Xavier Lima, Orlando Ferreira Jr., Rodrigo

Brindeiro, Luciana Jesus Costa e André Felipe Santos,

Laboratorio Hermes Pardini, Laboratorio Simile, Amilcar

Tanuri, Renato Santana Aguiar e Ana Tereza Vasconcelos


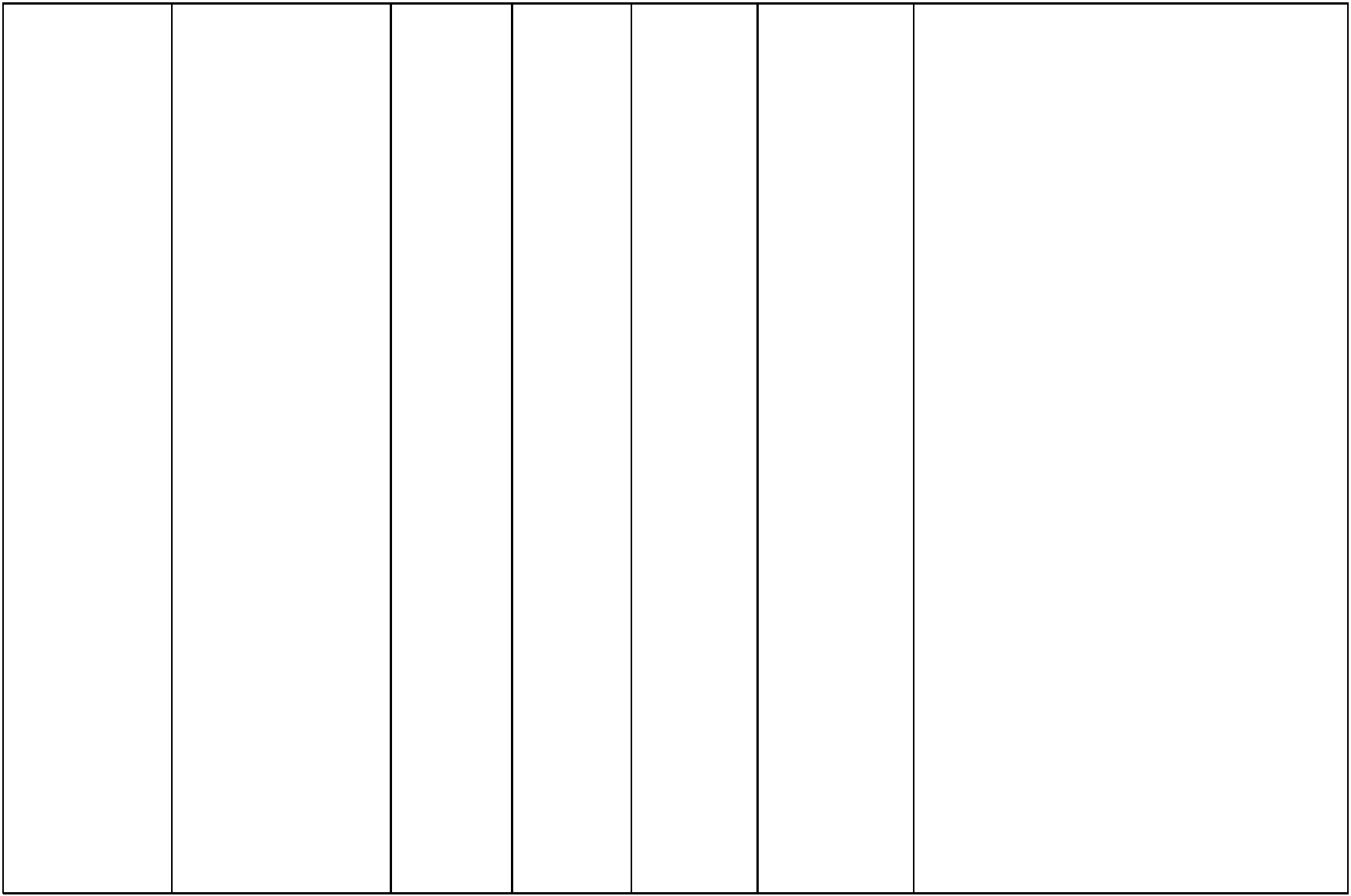


|  |  |  |  |  |  |  |  |  |  | Filipe Romero, Ana Paula Guimarães, Mariane Talon, Luiz |  |
| --- | --- | --- | --- | --- | --- | --- | --- | --- | --- | --- | --- |
|  |  |  |  |  |  |  |  |  |  | Gonzaga Paula de Almeida, Ronaldo da Silva Francisco |  |
|  |  |  |  |  |  |  |  |  |  | Junior, Diana Mariani, Lídia Boullosa, Alexandra Gerber, |  |
|  |  |  |  |  |  |  |  |  |  | Jaqueline Goes de Jesus, Ingra Morales Claro, Ester |  |
|  |  |  |  | South America / |  |  |  |  |  | Cerdeira Sabino, Nuno Rodrigues Faria, Terezinha Marta |  |
| EPI_ISL_417934 |  | hCoV-19/Brazil/RS0107/2020 |  |  | 2020-03-14 | | Laboratório |  | Bioinformatics | Pereira, Pinto Castiñeiras, Isabela de Carvalho Leitão, Rafael |  |
|  |  |  |  | Brazil / Rio |  |  |  |  |  |  |  |
|  |  |  |  |  |  |  | Hermes Pardini |  | Laboratory - LNCC | de Mello Galliez, Cássia Cristina Alves Gonçalves, Érica |  |
|  |  |  |  | Grande do Sul |  |  |  |  |  |  |  |
|  |  |  |  |  |  |  |  |  |  | Ramos dos Santos Nascimento, Richard Araújo Maia, Mauro |  |
|  |  |  |  |  |  |  |  |  |  |  |  |
|  |  |  |  |  |  |  |  |  |  | Teixeira,Cristiano Xavier Lima, Orlando Ferreira Jr., Rodrigo |  |
|  |  |  |  |  |  |  |  |  |  | Brindeiro, Luciana Jesus Costa e André Felipe Santos, |  |
|  |  |  |  |  |  |  |  |  |  | Laboratorio Hermes Pardini, Laboratorio Simile, Amilcar |  |
|  |  |  |  |  |  |  |  |  |  | Tanuri, Renato Santana Aguiar e Ana Tereza Vasconcelos |  |
|  |  |  |  |  |  |  |  |  |  |  |  |
|  |  |  |  |  |  |  |  |  |  | Filipe Romero, Ana Paula Guimarães, Mariane Talon, Luiz |  |
|  |  |  |  |  |  |  |  |  |  | Gonzaga Paula de Almeida, Ronaldo da Silva Francisco |  |
|  |  |  |  |  |  |  |  |  |  | Junior, Diana Mariani, Lídia Boullosa, Alexandra Gerber, |  |
|  |  |  |  |  |  |  |  |  |  | Jaqueline Goes de Jesus, Ingra Morales Claro, Ester |  |
|  |  |  |  | South America / |  |  |  |  |  | Cerdeira Sabino, Nuno Rodrigues Faria, Terezinha Marta |  |
| EPI_ISL_417936 |  | hCoV-19/Brazil/MG0108/2020 |  |  | 2020-03-15 | | Laboratório |  | Bioinformatics | Pereira, Pinto Castiñeiras, Isabela de Carvalho Leitão, Rafael |  |
|  |  |  |  | Brazil / Minas |  |  |  |  |  |  |  |
|  |  |  |  |  |  |  | Hermes Pardini |  | Laboratory - LNCC | de Mello Galliez, Cássia Cristina Alves Gonçalves, Érica |  |
|  |  |  |  | Gerais |  |  |  |  |  |  |  |
|  |  |  |  |  |  |  |  |  |  | Ramos dos Santos Nascimento, Richard Araújo Maia, Mauro |  |
|  |  |  |  |  |  |  |  |  |  |  |  |
|  |  |  |  |  |  |  |  |  |  | Teixeira,Cristiano Xavier Lima, Orlando Ferreira Jr., Rodrigo |  |
|  |  |  |  |  |  |  |  |  |  | Brindeiro, Luciana Jesus Costa e André Felipe Santos, |  |
|  |  |  |  |  |  |  |  |  |  | Laboratorio Hermes Pardini, Laboratorio Simile, Amilcar |  |
|  |  |  |  |  |  |  |  |  |  | Tanuri, Renato Santana Aguiar e Ana Tereza Vasconcelos |  |
|  |  |  |  |  |  |  |  |  |  |  |  |
|  |  |  |  |  |  |  |  |  |  | Filipe Romero, Ana Paula Guimarães, Mariane Talon, Luiz |  |
|  |  |  |  |  |  |  |  |  |  | Gonzaga Paula de Almeida, Ronaldo da Silva Francisco |  |
|  |  |  |  |  |  |  |  |  |  | Junior, Diana Mariani, Lídia Boullosa,Alexandra Gerber, |  |
|  |  |  |  |  |  |  |  |  |  | Jaqueline Goes de Jesus, Ingra Morales Claro, Ester |  |
|  |  |  |  | South America / |  |  | Laboratório |  | Bioinformatics | Cerdeira Sabino, Nuno Rodrigues Faria, Terezinha Marta |  |
| EPI_ISL_417940 |  | hCoV-19/Brazil/MG0109/2020 |  |  | 2020-03-16 | |  |  |  | Pereira, Pinto Castiñeiras, Isabela de Carvalho Leitão, Rafael |  |
|  |  |  |  | Brazil / Minas |  |  |  |  |  |  |  |
|  |  |  |  |  |  |  | Hermes Pardini |  | Laboratory - LNCC | de Mello Galliez, Cássia Alves Gonçalves, Érica Ramos dos |  |
|  |  |  |  |  |  |  |  |  |  |  |  |

Gerais

Teixeira,Cristiano Xavier Lima, Orlando Ferreira Jr., Rodrigo

Brindeiro, Luciana Jesus Costa e André Felipe Santos,

Laboratorio Hermes Pardini, Laboratorio Simile, Amilcar

Tanuri, Renato Santana Aguiar e Ana Tereza Vasconcelos


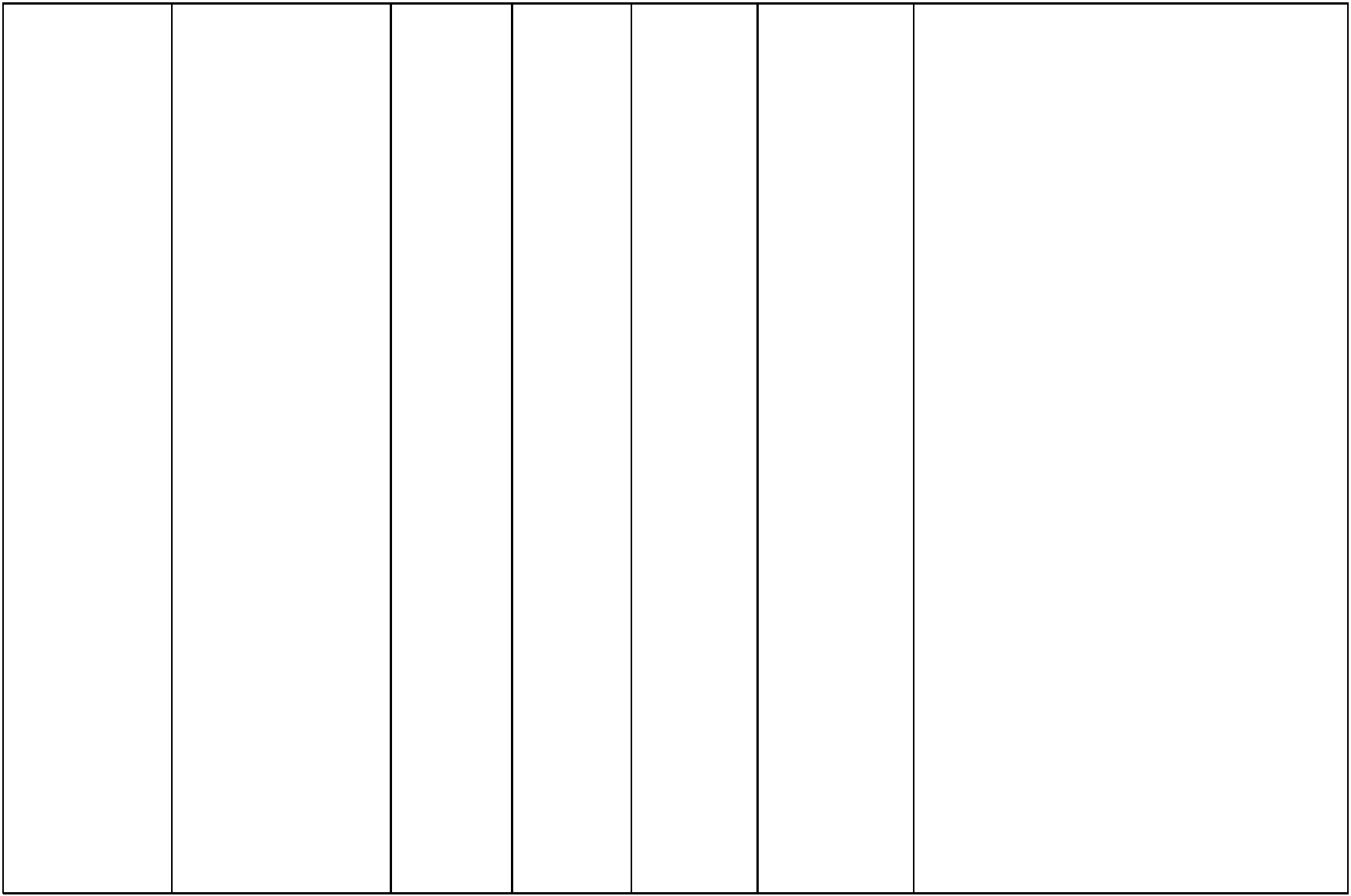


|  |  |  |  |  |  |  |  |  |  | Filipe Romero, Ana Paula Guimarães, Mariane Talon, Luiz |  |
| --- | --- | --- | --- | --- | --- | --- | --- | --- | --- | --- | --- |
|  |  |  |  |  |  |  |  |  |  | Gonzaga Paula de Almeida, Ronaldo da Silva Francisco |  |
|  |  |  |  |  |  |  |  |  |  | Junior, Diana Mariani, Lídia Boullosa,Alexandra Gerber, |  |
|  |  |  |  |  |  |  |  |  |  | Jaqueline Goes de Jesus, Ingra Morales Claro, Ester |  |
|  |  |  |  | South America / |  |  |  |  |  | Cerdeira Sabino, Nuno Rodrigues Faria, Terezinha Marta |  |
| EPI_ISL_417943 |  | hCoV-19/Brazil/SP0110/2020 |  |  | 2020-03-17 | | Laboratório |  | Bioinformatics | Pereira, Pinto Castiñeiras, Isabela de Carvalho Leitão, Rafael |  |
|  |  |  |  | Brazil / São |  |  |  |  |  |  |  |
|  |  |  |  |  |  |  | Hermes Pardini |  | Laboratory | de Mello Galliez, Cássia Alves Gonçalves, Érica Ramos dos |  |
|  |  |  |  | Paulo |  |  |  |  |  |  |  |
|  |  |  |  |  |  |  |  |  |  | Santos Nascimento, Richard Araújo Maia, Mauro |  |
|  |  |  |  |  |  |  |  |  |  |  |  |
|  |  |  |  |  |  |  |  |  |  | Teixeira,Cristiano Xavier Lima, Orlando Ferreira Jr., Rodrigo |  |
|  |  |  |  |  |  |  |  |  |  | Brindeiro, Luciana Jesus Costa e André Felipe Santos, |  |
|  |  |  |  |  |  |  |  |  |  | Laboratorio Hermes Pardini, Laboratorio Simile, Amilcar |  |
|  |  |  |  |  |  |  |  |  |  | Tanuri, Renato Santana Aguiar e Ana Tereza Vasconcelos |  |
|  |  |  |  |  |  |  |  |  |  |  |  |
|  |  |  |  |  |  |  |  |  |  | Filipe Romero, Ana Paula Guimarães, Mariane Talon, Luiz |  |
|  |  |  |  |  |  |  |  |  |  | Gonzaga Paula de Almeida, Ronaldo da Silva Francisco |  |
|  |  |  |  |  |  |  |  |  |  | Junior, Diana Mariani, Lídia Boullosa,Alexandra Gerber, |  |
|  |  |  |  |  |  |  |  |  |  | Jaqueline Goes de Jesus, Ingra Morales Claro, Ester |  |
|  |  |  |  | South America / |  |  |  |  |  | Cerdeira Sabino, Nuno Rodrigues Faria, Terezinha Marta |  |
| EPI_ISL_417945 |  | hCoV-19/Brazil/SP0111/2020 |  |  | 2020-03-17 | | Laboratório |  | Bioinformatics | Pereira, Pinto Castiñeiras, Isabela de Carvalho Leitão, Rafael |  |
|  |  |  |  | Brazil / São |  |  |  |  |  |  |  |
|  |  |  |  |  |  |  | Hermes Pardini |  | Laboratory - LNCC | de Mello Galliez, Cássia Alves Gonçalves, Érica Ramos dos |  |
|  |  |  |  | Paulo |  |  |  |  |  |  |  |
|  |  |  |  |  |  |  |  |  |  | Santos Nascimento, Richard Araújo Maia, Mauro |  |
|  |  |  |  |  |  |  |  |  |  |  |  |
|  |  |  |  |  |  |  |  |  |  | Teixeira,Cristiano Xavier Lima, Orlando Ferreira Jr., Rodrigo |  |
|  |  |  |  |  |  |  |  |  |  | Brindeiro, Luciana Jesus Costa e André Felipe Santos, |  |
|  |  |  |  |  |  |  |  |  |  | Laboratorio Hermes Pardini, Laboratorio Simile, Amilcar |  |
|  |  |  |  |  |  |  |  |  |  | Tanuri, Renato Santana Aguiar e Ana Tereza Vasconcelos |  |
|  |  |  |  |  |  |  |  |  |  |  |  |
|  |  |  |  |  |  |  |  |  |  | Filipe Romero, Ana Paula Guimarães, Mariane Talon, Luiz |  |
|  |  |  |  |  |  |  |  |  |  | Gonzaga Paula de Almeida, Ronaldo da Silva Francisco |  |
|  |  |  |  |  |  |  |  |  |  | Junior, Diana Mariani, Lídia Boullosa,Alexandra Gerber, |  |
|  |  |  |  |  |  |  |  |  |  | Jaqueline Goes de Jesus, Ingra Morales Claro, Ester |  |
|  |  |  |  | South America / |  |  | Laboratório |  | Bioinformatics | Cerdeira Sabino, Nuno Rodrigues Faria, Terezinha Marta |  |
| EPI_ISL_417949 |  | hCoV-19/Brazil/MG0112/2020 |  |  | 2020-03-17 | |  |  |  | Pereira, Pinto Castiñeiras, Isabela de Carvalho Leitão, Rafael |  |
|  |  |  |  | Brazil / Minas |  |  |  |  |  |  |  |
|  |  |  |  |  |  |  | Hermes Pardini |  | Laboratory - LNCC | de Mello Galliez, Cássia Alves Gonçalves, Érica Ramos dos |  |
|  |  |  |  |  |  |  |  |  |  |  |  |

Gerais

Teixeira,Cristiano Xavier Lima, Orlando Ferreira Jr., Rodrigo

Brindeiro, Luciana Jesus Costa e André Felipe Santos,

Laboratorio Hermes Pardini, Laboratorio Simile, Amilcar

Tanuri, Renato Santana Aguiar e Ana Tereza Vasconcelos


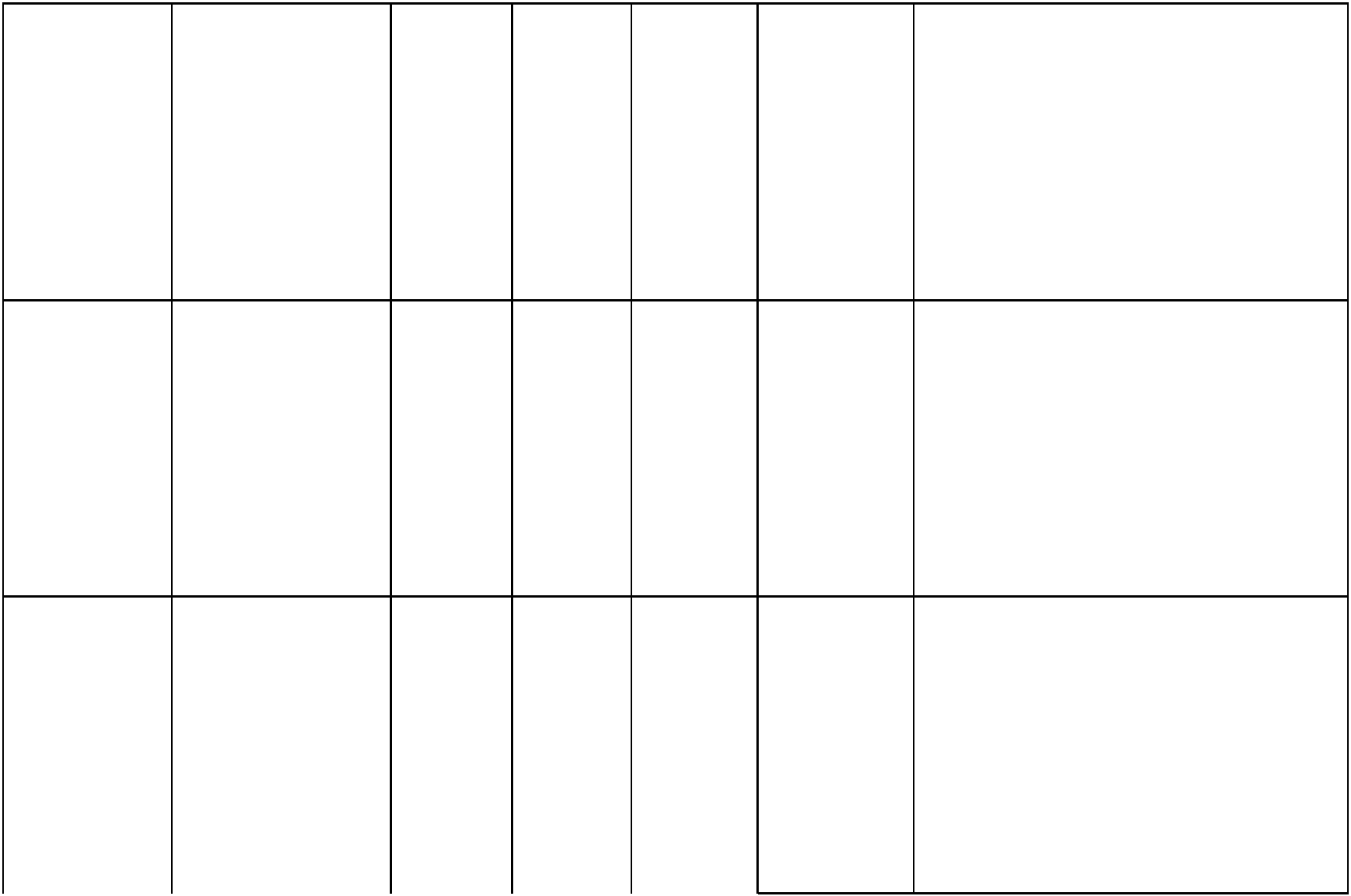


|  | South America / |  | Universidade |
| --- | --- | --- | --- |
| EPI_ISL_417951 | hCoV-19/Brazil/RJ0114/2020 Brazil / Rio de | 2020-03-17 | Federal do Rio |
|  | Janeiro |  | de Janeiro |

|  | South America / |  | Universidade |
| --- | --- | --- | --- |
| EPI_ISL_417953 | hCoV-19/Brazil/RJ0115/2020 Brazil / Rio de | 2020-03-18 | Federal do Rio |
|  | Janeiro |  | de Janeiro |

|  |  |  |  | South America / |  |  | Universidade |
| --- | --- | --- | --- | --- | --- | --- | --- |
| EPI_ISL_417982 |  | hCoV-19/Brazil/RJ0116/2020 Brazil / Rio de | | | 2020-03-19 | | Federal do Rio |
|  |  |  |  | Janeiro |  |  | de Janeiro |
|  |  |  |  |  |  |  |  |

Bioinformatics

Laboratory - LNCC

Bioinformatics

Laboratory - LNCC

Bioinformatics

Laboratory - LNCC

Filipe Romero, Ana Paula Guimarães, Mariane Talon, Luiz Gonzaga Paula de Almeida, Ronaldo da Silva Francisco Junior, Diana Mariani, Lídia Boullosa,Alexandra Gerber, Jaqueline Goes de Jesus, Ingra Morales Claro, Ester Cerdeira Sabino, Nuno Rodrigues Faria, Terezinha Marta Pereira, Pinto Castiñeiras, Isabela de Carvalho Leitão, Rafael de Mello Galliez, Cássia Alves Gonçalves, Érica Ramos dos Santos Nascimento, Richard Araújo Maia, Mauro Teixeira,Cristiano Xavier Lima, Orlando Ferreira Jr., Rodrigo Brindeiro, Luciana Jesus Costa e André Felipe Santos, Laboratorio Hermes Pardini, Laboratorio Simile, Amilcar Tanuri, Renato Santana Aguiar e Ana Tereza Vasconcelos

Filipe Romero, Ana Paula Guimarães, Mariane Talon, Luiz Gonzaga Paula de Almeida, Ronaldo da Silva Francisco Junior, Diana Mariani, Lídia Boullosa,Alexandra Gerber, Jaqueline Goes de Jesus, Ingra Morales Claro, Ester Cerdeira Sabino, Nuno Rodrigues Faria, Terezinha Marta Pereira, Pinto Castiñeiras, Isabela de Carvalho Leitão, Rafael de Mello Galliez, Cássia Alves Gonçalves, Érica Ramos dos Santos Nascimento, Richard Araújo Maia, Mauro Teixeira,Cristiano Xavier Lima, Orlando Ferreira Jr., Rodrigo Brindeiro, Luciana Jesus Costa e André Felipe Santos, Laboratorio Hermes Pardini, Laboratorio Simile, Amilcar Tanuri, Renato Santana Aguiar e Ana Tereza Vasconcelos

Filipe Romero, Ana Paula Guimarães, Mariane Talon, Luiz Gonzaga Paula de Almeida, Ronaldo da Silva Francisco Junior, Diana Mariani, Lídia Boullosa,Alexandra Gerber, Jaqueline Goes de Jesus, Ingra Morales Claro, Ester Cerdeira Sabino, Nuno Rodrigues Faria, Terezinha Marta Pereira, Pinto Castiñeiras, Isabela de Carvalho Leitão, Rafael de Mello Galliez, Cássia Alves Gonçalves, Érica Ramos dos Santos Nascimento, Richard Araújo Maia, Mauro Teixeira,Cristiano Xavier Lima, Orlando Ferreira Jr., Rodrigo Brindeiro, Luciana Jesus Costa e André Felipe Santos, Laboratorio Hermes Pardini, Laboratorio Simile, Amilcar Tanuri, Renato Santana Aguiar e Ana Tereza Vasconcelos


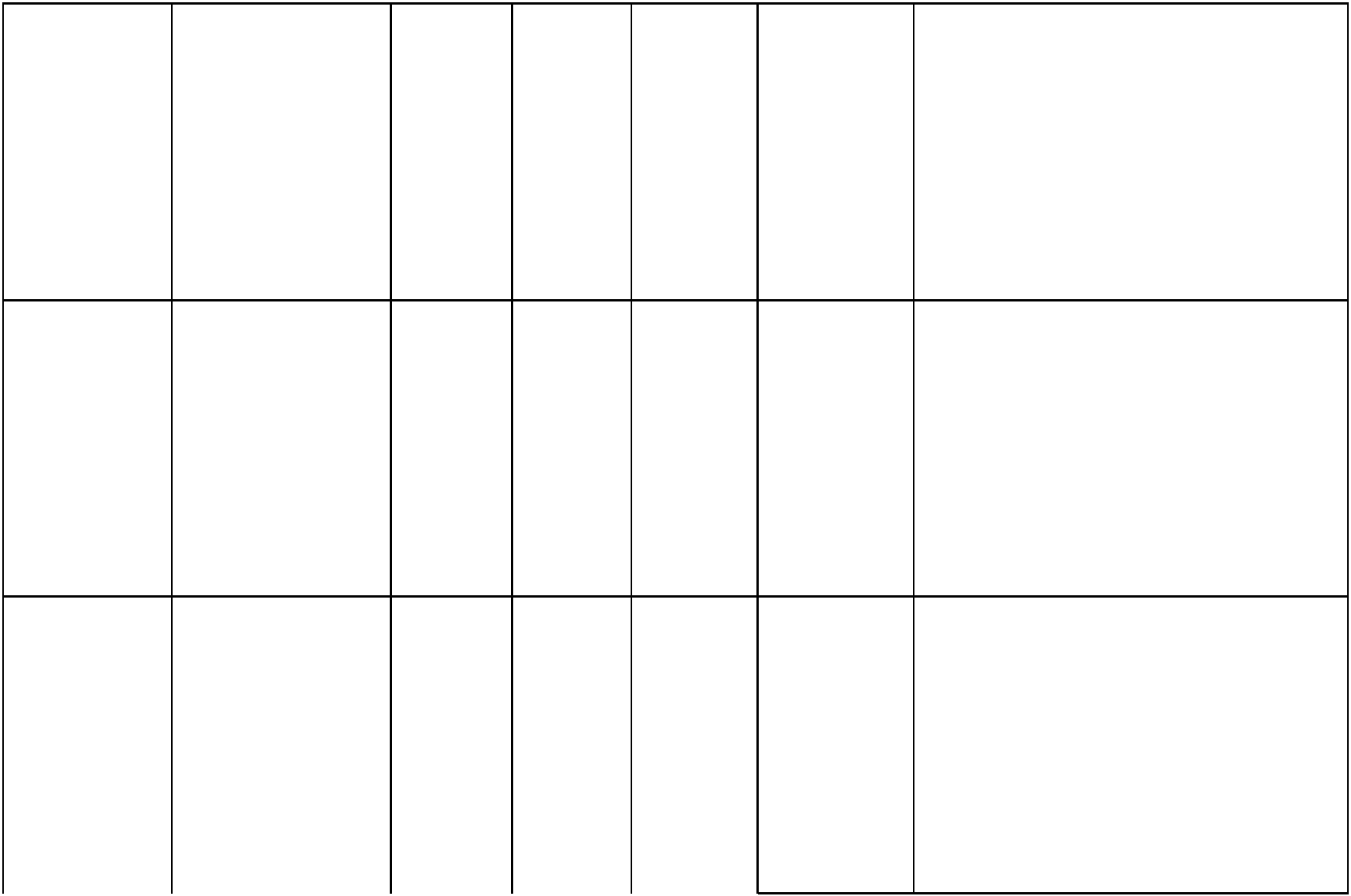


|  | South America / |  | Universidade |
| --- | --- | --- | --- |
| EPI_ISL_417983 | hCoV-19/Brazil/RJ0117/2020 Brazil / Rio de | 2020-03-19 | Federal do Rio |
|  | Janeiro |  | de Janeiro |

|  | South America / |  | Universidade |
| --- | --- | --- | --- |
| EPI_ISL_417984 | hCoV-19/Brazil/RJ0118/2020 Brazil / Rio de | 2020-03-19 | Federal do Rio |
|  | Janeiro |  | de Janeiro |

|  |  |  |  | South America / |  |  | Universidade |
| --- | --- | --- | --- | --- | --- | --- | --- |
| EPI_ISL_417985 |  | hCoV-19/Brazil/RJ0119/2020 Brazil / Rio de | | | 2020-03-19 | | Federal do Rio |
|  |  |  |  | Janeiro |  |  | de Janeiro |
|  |  |  |  |  |  |  |  |

Bioinformatics

Laboratory - LNCC

Bioinformatics

Laboratory - LNCC

Bioinformatics

Laboratory - LNCC

Filipe Romero, Ana Paula Guimarães, Mariane Talon, Luiz Gonzaga Paula de Almeida, Ronaldo da Silva Francisco Junior, Diana Mariani, Lídia Boullosa,Alexandra Gerber, Jaqueline Goes de Jesus, Ingra Morales Claro, Ester Cerdeira Sabino, Nuno Rodrigues Faria, Terezinha Marta Pereira, Pinto Castiñeiras, Isabela de Carvalho Leitão, Rafael de Mello Galliez, Cássia Alves Gonçalves, Érica Ramos dos Santos Nascimento, Richard Araújo Maia, Mauro Teixeira,Cristiano Xavier Lima, Orlando Ferreira Jr., Rodrigo Brindeiro, Luciana Jesus Costa e André Felipe Santos, Laboratorio Hermes Pardini, Laboratorio Simile, Amilcar Tanuri, Renato Santana Aguiar e Ana Tereza Vasconcelos

Filipe Romero, Ana Paula Guimarães, Mariane Talon, Luiz Gonzaga Paula de Almeida, Ronaldo da Silva Francisco Junior, Diana Mariani, Lídia Boullosa,Alexandra Gerber, Jaqueline Goes de Jesus, Ingra Morales Claro, Ester Cerdeira Sabino, Nuno Rodrigues Faria, Terezinha Marta Pereira, Pinto Castiñeiras, Isabela de Carvalho Leitão, Rafael de Mello Galliez, Cássia Alves Gonçalves, Érica Ramos dos Santos Nascimento, Richard Araújo Maia, Mauro Teixeira,Cristiano Xavier Lima, Orlando Ferreira Jr., Rodrigo Brindeiro, Luciana Jesus Costa e André Felipe Santos, Laboratorio Hermes Pardini, Laboratorio Simile, Amilcar Tanuri, Renato Santana Aguiar e Ana Tereza Vasconcelos

Filipe Romero, Ana Paula Guimarães, Mariane Talon, Luiz Gonzaga Paula de Almeida, Ronaldo da Silva Francisco Junior, Diana Mariani, Lídia Boullosa,Alexandra Gerber, Jaqueline Goes de Jesus, Ingra Morales Claro, Ester Cerdeira Sabino, Nuno Rodrigues Faria, Terezinha Marta Pereira, Pinto Castiñeiras, Isabela de Carvalho Leitão, Rafael de Mello Galliez, Cássia Alves Gonçalves, Érica Ramos dos Santos Nascimento, Richard Araújo Maia, Mauro Teixeira,Cristiano Xavier Lima, Orlando Ferreira Jr., Rodrigo Brindeiro, Luciana Jesus Costa e André Felipe Santos, Laboratorio Hermes Pardini, Laboratorio Simile, Amilcar Tanuri, Renato Santana Aguiar e Ana Tereza Vasconcelos

EPI_ISL_418959

EPI_ISL_426580

EPI_ISL_427292

EPI_ISL_427293

EPI_ISL_427294

|  |  |  |  |  |  |  |  | Filipe Romero, Ana Paula Guimarães, Mariane Talon, Luiz |  |
| --- | --- | --- | --- | --- | --- | --- | --- | --- | --- |
|  |  |  |  |  |  |  |  | Gonzaga Paula de Almeida, Ronaldo da Silva Francisco |  |
|  |  |  |  |  |  |  |  | Junior, Diana Mariani, Lídia Boullosa, Alexandra Gerber, |  |
|  |  |  |  |  |  |  |  | Jaqueline Goes de Jesus, Ingra Morales Claro, Ester |  |
|  |  | South America / |  |  | Universidade |  |  | Cerdeira Sabino, Nuno Rodrigues Faria, Terezinha Marta |  |
| hCoV-19/Brazil/RJ0113/2020 |  |  | 2020-03-17 | | Federal do Rio |  | Bioinformatics | Pereira, Pinto Castiñeiras, Isabela de Carvalho Leitão, Rafael |  |
|  |  | Brazil / Rio de |  |  |  |  |  |  |  |
|  |  |  |  |  | de Janeiro - |  | Laboratory - LNCC | de Mello Galliez, Cássia Cristina Alves Gonçalves, Érica |  |
|  |  | Janeiro |  |  |  |  |  |  |  |
|  |  |  |  |  | UFRJ |  |  | Ramos dos Santos Nascimento, Richard Araújo Maia, Mauro |  |
|  |  |  |  |  |  |  |  |  |  |
|  |  |  |  |  |  |  |  | Teixeira,Cristiano Xavier Lima, Orlando Ferreira Jr., Rodrigo |  |
|  |  |  |  |  |  |  |  | Brindeiro, Luciana Jesus Costa e André Felipe Santos, |  |
|  |  |  |  |  |  |  |  | Laboratorio Hermes Pardini, Laboratorio Simile, Amilcar |  |
|  |  |  |  |  |  |  |  | Tanuri, Renato Santana Aguiar, e Ana Tereza Vasconcelos |  |
|  |  |  |  |  |  |  |  |  |  |
|  |  | South America / |  |  |  |  |  | Fernando L Melo,Gustavo Barra, Ticiane H Santa-Rita, |  |
| hCoV-19/Brazil/DFBR- |  | Brazil / Distrito | 2020-03-13 | | Instituto Sabin |  | Laboratory of |  |  |
|  |  |  |  |  |  |  |  | Pedro G Mesquita, Ikaro A Andrade, Tatsuya Nagata, |  |
| 0001/2020 |  | Federal / |  |  |  |  | Virology |  |  |
|  |  |  |  |  |  |  |  | Bergmann M Ribeiro |  |
|  |  | Brasilia |  |  |  |  |  |  |  |
|  |  |  |  |  |  |  |  |  |  |
|  |  |  |  |  |  |  |  |  |  |
|  |  |  |  |  | LACEN-AL - |  | Instituto Oswaldo | Paola Resende, Fernando Motta, Luciana Appolinario, |  |
|  |  | South America / |  |  |  |  | Cruz FIOCRUZ - |  |  |
| hCoV-19/Brazil/AL-837/2020 |  |  | 2020-03-18 | | Laboratorio |  |  | Sunando Roy, Aline Mattos, Milene Miranda, Cristiana |  |
|  |  | Brazil / Alagoas |  |  |  |  | Laboratory of |  |  |
|  |  |  |  |  | Central de |  |  | Garcia, Braulia Caetano, Maria Ogrzewalska, Priscila Born, |  |
|  |  | / Maceio |  |  |  |  | Respiratory Viruses |  |  |
|  |  |  |  |  | Alagoas |  |  | Jonathan Lopes, Marilda Siqueira |  |
|  |  |  |  |  |  |  | and Measles (LVRS) |  |  |
|  |  |  |  |  |  |  |  |  |  |
|  |  |  |  |  |  |  |  |  |  |
|  |  |  |  |  | LACEN-BA - |  | Instituto Oswaldo |  |  |
|  |  | South America / |  |  | Laboratório |  |  | Paola Resende, Fernando Motta, Luciana Appolinario, |  |
|  |  |  |  |  |  |  | Cruz FIOCRUZ - |  |  |
| hCoV-19/Brazil/BA-510/2020 |  | Brazil / Bahia / | 2020-03-06 | | Central de |  |  | Sunando Roy, Aline Mattos, Milene Miranda, Cristiana |  |
|  |  |  |  |  |  |  | Laboratory of |  |  |
|  |  | Feira de |  |  | Saúde Pública |  |  | Garcia, Braulia Caetano, Maria Ogrzewalska, Priscila Born, |  |
|  |  |  |  |  |  |  | Respiratory Viruses |  |  |
|  |  | Santana |  |  | Professor |  |  | Jonathan Lopes, Marilda Siqueira |  |
|  |  |  |  |  |  |  | and Measles (LVRS) |  |  |
|  |  |  |  |  | Gonçalo Moniz |  |  |  |  |
|  |  |  |  |  |  |  |  |  |  |
|  |  |  |  |  |  |  |  |  |  |
|  |  |  |  |  | Instituto |  |  |  |  |
|  |  | South America / |  |  | Oswaldo Cruz |  | Instituto Oswaldo | Paola Resende, Fernando Motta, Luciana Appolinario, |  |
|  |  |  |  |  | FIOCRUZ - |  | Cruz FIOCRUZ - |  |  |
|  |  | Brazil / Distrito |  |  |  |  |  | Sunando Roy, Aline Mattos, Milene Miranda, Cristiana |  |
| hCoV-19/Brazil/DF-615i/2020 |  |  | 2020-03-13 | | Laboratory of |  | Laboratory of |  |  |
|  |  | Federal / |  |  |  |  |  | Garcia, Braulia Caetano, Maria Ogrzewalska, Priscila Born, |  |
|  |  |  |  |  | Respiratory |  | Respiratory Viruses |  |  |
|  |  | Brasilia |  |  |  |  |  | Jonathan Lopes, Marilda Siqueira |  |
|  |  |  |  |  | Viruses and |  | and Measles (LVRS) |  |  |
|  |  |  |  |  |  |  |  |  |  |


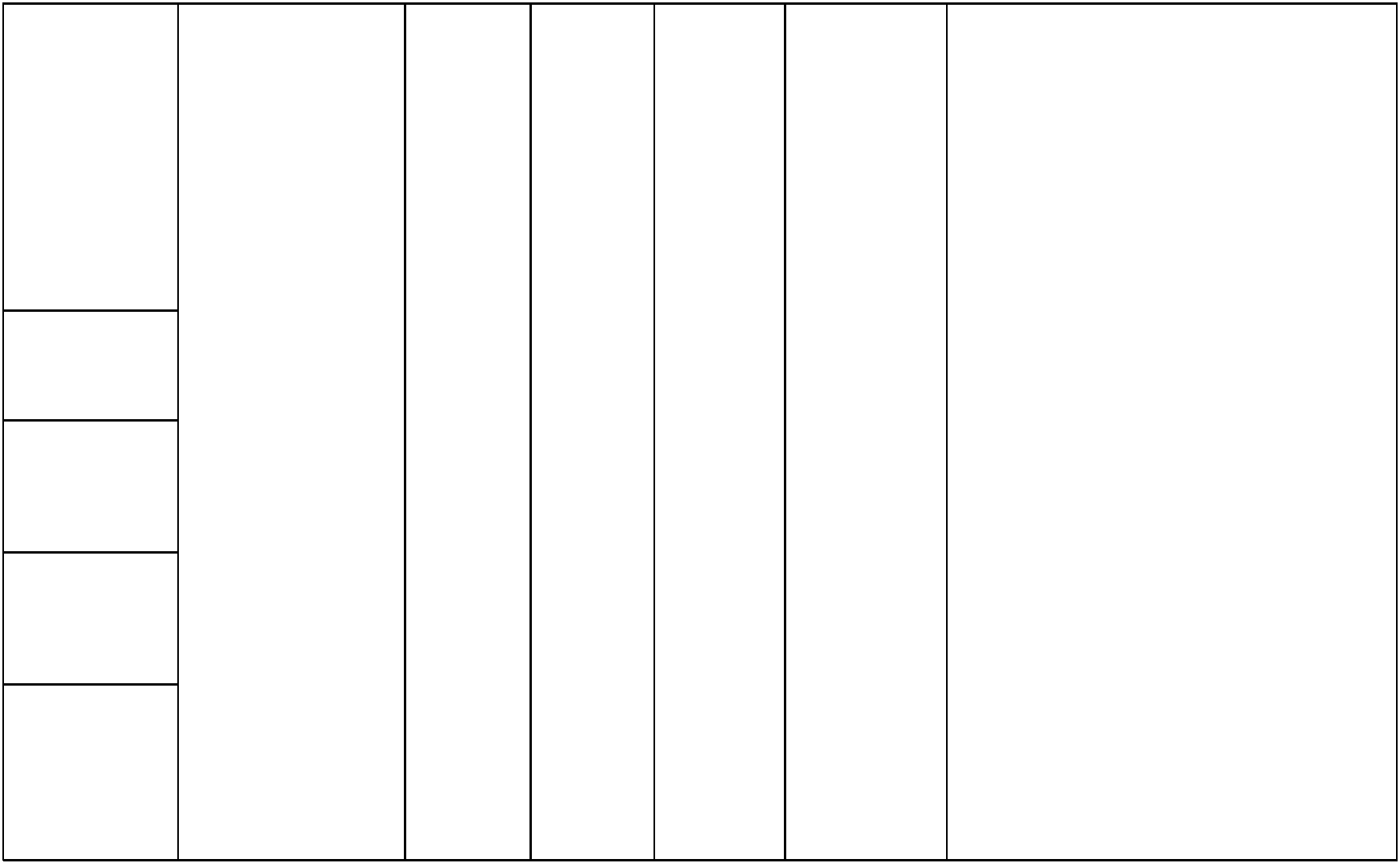


Measles (LVRS)

EPI_ISL_427295

EPI_ISL_427296

EPI_ISL_427297

EPI_ISL_427298

EPI_ISL_427299

hCoV-19/Brazil/DF-619i/2020

hCoV-19/Brazil/DF-861/2020

hCoV-19/Brazil/DF-862/2020

hCoV-19/Brazil/DF-891/2020

hCoV-19/Brazil/RJ-352/2020

South America /

Brazil / Distrito

Federal /

Brasilia

South America /

Brazil / Distrito

Federal /

Brasilia

South America /

Brazil / Distrito

Federal /

Brasilia

South America /

Brazil / Distrito

Federal /

Brasilia

South America / Brazil / Rio de Janeiro / Niteroi

|  | Instituto |  |  |  |
| --- | --- | --- | --- | --- |
|  | Oswaldo Cruz |  | Instituto Oswaldo |  |
|  | FIOCRUZ - |  | Cruz FIOCRUZ - |  |
| 2020-03-13 | Laboratory of |  | Laboratory of |  |
|  | Respiratory |  | Respiratory Viruses |  |
|  | Viruses and |  | and Measles (LVRS) |  |
|  | Measles (LVRS) |  |  |  |
|  |  |  |  |  |
|  | Instituto |  |  |  |
|  | Oswaldo Cruz |  | Instituto Oswaldo |  |
|  | FIOCRUZ - |  | Cruz FIOCRUZ - |  |
| 2020-03-23 | Laboratory of |  | Laboratory of |  |
|  | Respiratory |  | Respiratory Viruses |  |
|  | Viruses and |  | and Measles (LVRS) |  |
|  | Measles (LVRS) |  |  |  |
|  |  |  |  |  |
|  | Instituto |  |  |  |
|  | Oswaldo Cruz |  | Instituto Oswaldo |  |
| 2020-03-23 | FIOCRUZ - |  | Cruz FIOCRUZ - |  |
|  | Laboratory of |  | Laboratory of |  |
|  | Respiratory |  | Respiratory Viruses |  |
|  | Viruses and |  | and Measles (LVRS) |  |
|  | Measles (LVRS) |  |  |  |
|  |  |  |  |  |
|  | Instituto |  |  |  |
|  | Oswaldo Cruz |  | Instituto Oswaldo |  |
|  | FIOCRUZ - |  | Cruz FIOCRUZ - |  |
| 2020-03-22 | Laboratory of |  | Laboratory of |  |
|  | Respiratory |  | Respiratory Viruses |  |
|  | Viruses and |  | and Measles (LVRS) |  |
|  | Measles (LVRS) |  |  |  |
|  |  |  |  |  |
|  | Instituto |  |  |  |
|  | Oswaldo Cruz |  | Instituto Oswaldo |  |
|  | FIOCRUZ - |  | Cruz FIOCRUZ - |  |
| 2020-03-05 | Laboratory of |  | Laboratory of |  |
|  | Respiratory |  | Respiratory Viruses |  |
|  | Viruses and |  | and Measles (LVRS) |  |
|  | Measles (LVRS) |  |  |  |
|  |  |  |  |  |


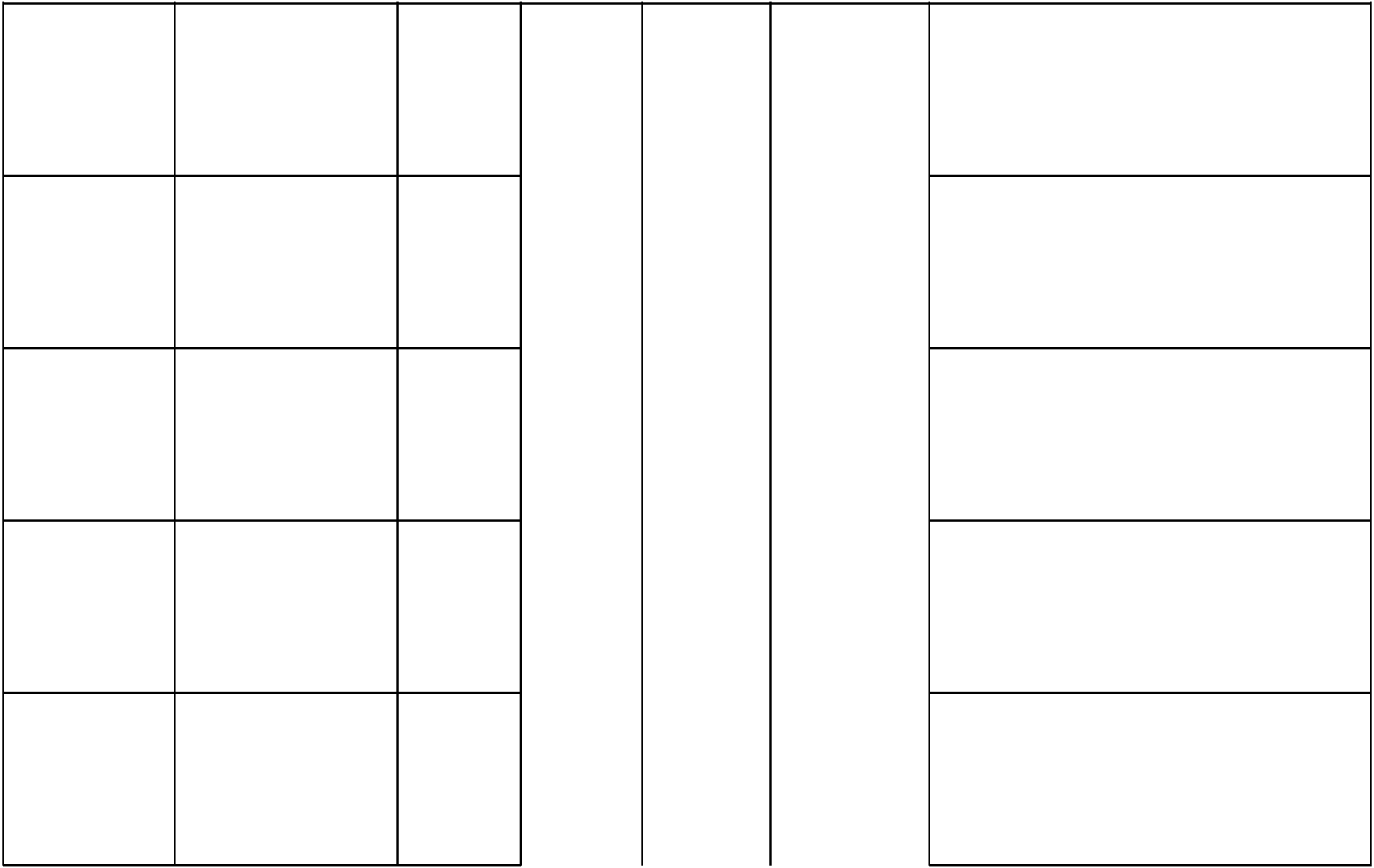


Paola Resende, Fernando Motta, Luciana Appolinario, Sunando Roy, Aline Mattos, Milene Miranda, Cristiana Garcia, Braulia Caetano, Maria Ogrzewalska, Priscila Born, Jonathan Lopes, Marilda Siqueira

Paola Resende, Fernando Motta, Luciana Appolinario, Sunando Roy, Aline Mattos, Milene Miranda, Cristiana Garcia, Braulia Caetano, Maria Ogrzewalska, Priscila Born, Jonathan Lopes, Marilda Siqueira

Paola Resende, Fernando Motta, Luciana Appolinario, Sunando Roy, Aline Mattos, Milene Miranda, Cristiana Garcia, Braulia Caetano, Maria Ogrzewalska, Priscila Born, Jonathan Lopes, Marilda Siqueira

Paola Resende, Fernando Motta, Luciana Appolinario, Sunando Roy, Aline Mattos, Milene Miranda, Cristiana Garcia, Braulia Caetano, Maria Ogrzewalska, Priscila Born, Jonathan Lopes, Marilda Siqueira

Paola Resende, Fernando Motta, Luciana Appolinario, Sunando Roy, Aline Mattos, Milene Miranda, Cristiana Garcia, Braulia Caetano, Maria Ogrzewalska, Priscila Born, Jonathan Lopes, Marilda Siqueira

EPI_ISL_427300

EPI_ISL_427301

EPI_ISL_427302

EPI_ISL_427303

EPI_ISL_427304

hCoV-19/Brazil/RJ-477/2020

hCoV-19/Brazil/RJ-477i/2020

hCoV-19/Brazil/RJ-763/2020

hCoV-19/Brazil/RJ-818/2020

hCoV-19/Brazil/RJ-872/2020

South America / Brazil / Rio de Janeiro / Rio de Janeiro

South America / Brazil / Rio de Janeiro / Rio de Janeiro

South America / Brazil / Rio de Janeiro / Rio de Janeiro

South America / Brazil / Rio de Janeiro / Rio de Janeiro

South America / Brazil / Rio de Janeiro / Rio de Janeiro

|  | Instituto |  |  |  |
| --- | --- | --- | --- | --- |
|  | Oswaldo Cruz |  | Instituto Oswaldo |  |
|  | FIOCRUZ - |  | Cruz FIOCRUZ - |  |
| 2020-03-11 | Laboratory of |  | Laboratory of |  |
|  | Respiratory |  | Respiratory Viruses |  |
|  | Viruses and |  | and Measles (LVRS) |  |
|  | Measles (LVRS) |  |  |  |
|  |  |  |  |  |
|  | Instituto |  |  |  |
|  | Oswaldo Cruz |  | Instituto Oswaldo |  |
|  | FIOCRUZ - |  | Cruz FIOCRUZ - |  |
| 2020-03-11 | Laboratory of |  | Laboratory of |  |
|  | Respiratory |  | Respiratory Viruses |  |
|  | Viruses and |  | and Measles (LVRS) |  |
|  | Measles (LVRS) |  |  |  |
|  |  |  |  |  |
|  | Instituto |  |  |  |
|  | Oswaldo Cruz |  | Instituto Oswaldo |  |
| 2020-03-20 | FIOCRUZ - |  | Cruz FIOCRUZ - |  |
|  | Laboratory of |  | Laboratory of |  |
|  | Respiratory |  | Respiratory Viruses |  |
|  | Viruses and |  | and Measles (LVRS) |  |
|  | Measles (LVRS) |  |  |  |
|  |  |  |  |  |
|  | Instituto |  |  |  |
|  | Oswaldo Cruz |  | Instituto Oswaldo |  |
|  | FIOCRUZ - |  | Cruz FIOCRUZ - |  |
| 2020-03-25 | Laboratory of |  | Laboratory of |  |
|  | Respiratory |  | Respiratory Viruses |  |
|  | Viruses and |  | and Measles (LVRS) |  |
|  | Measles (LVRS) |  |  |  |
|  |  |  |  |  |
|  | Instituto |  |  |  |
|  | Oswaldo Cruz |  | Instituto Oswaldo |  |
|  | FIOCRUZ - |  | Cruz FIOCRUZ - |  |
| 2020-03-26 | Laboratory of |  | Laboratory of |  |
|  | Respiratory |  | Respiratory Viruses |  |
|  | Viruses and |  | and Measles (LVRS) |  |
|  | Measles (LVRS) |  |  |  |
|  |  |  |  |  |


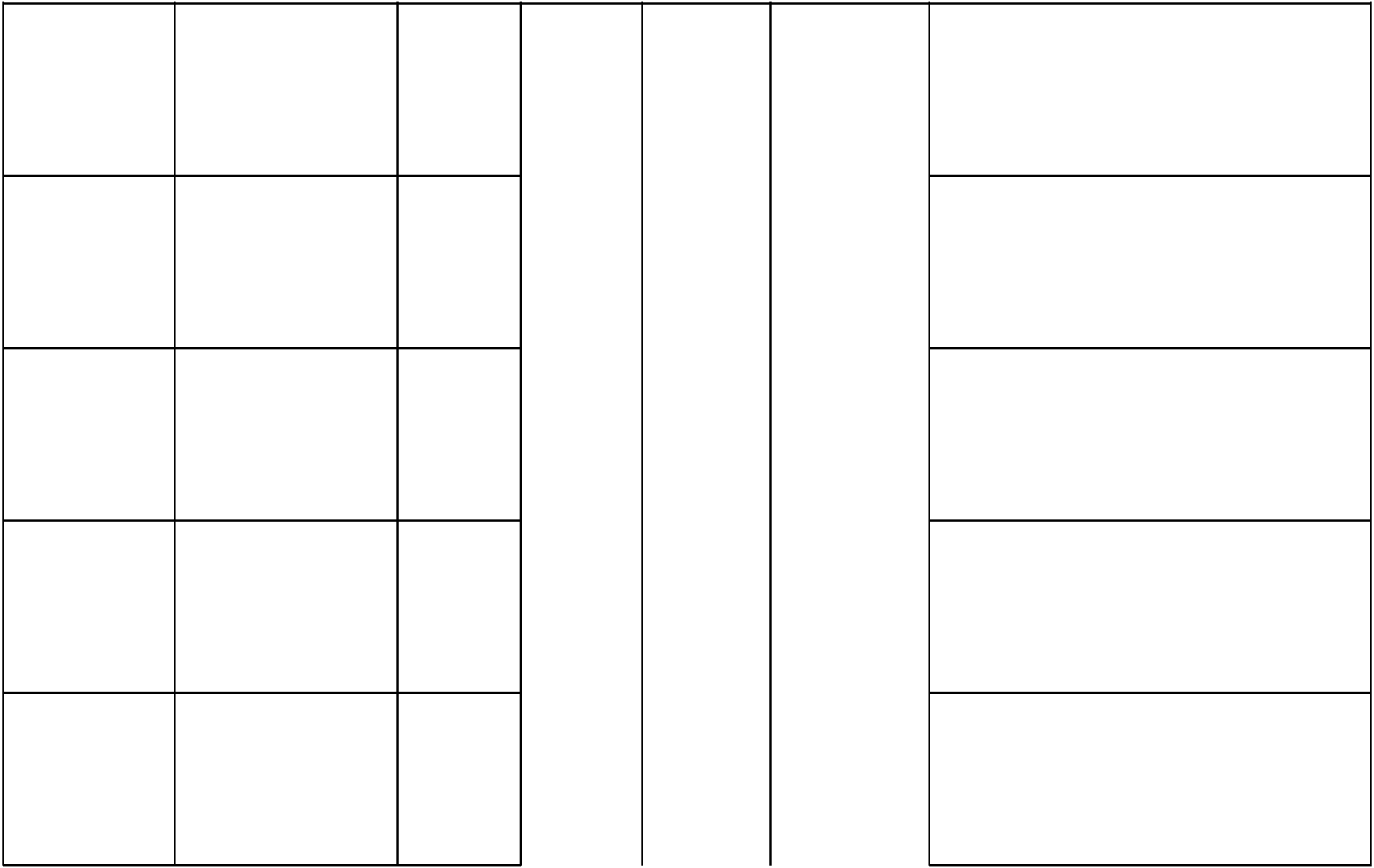


Paola Resende, Fernando Motta, Luciana Appolinario, Sunando Roy, Aline Mattos, Milene Miranda, Cristiana Garcia, Braulia Caetano, Maria Ogrzewalska, Priscila Born, Jonathan Lopes, Marilda Siqueira

Paola Resende, Fernando Motta, Luciana Appolinario, Sunando Roy, Aline Mattos, Milene Miranda, Cristiana Garcia, Braulia Caetano, Maria Ogrzewalska, Priscila Born, Jonathan Lopes, Marilda Siqueira

Paola Resende, Fernando Motta, Luciana Appolinario, Sunando Roy, Aline Mattos, Milene Miranda, Cristiana Garcia, Braulia Caetano, Maria Ogrzewalska, Priscila Born, Jonathan Lopes, Marilda Siqueira

Paola Resende, Fernando Motta, Luciana Appolinario, Sunando Roy, Aline Mattos, Milene Miranda, Cristiana Garcia, Braulia Caetano, Maria Ogrzewalska, Priscila Born, Jonathan Lopes, Marilda Siqueira

Paola Resende, Fernando Motta, Luciana Appolinario, Sunando Roy, Aline Mattos, Milene Miranda, Cristiana Garcia, Braulia Caetano, Maria Ogrzewalska, Priscila Born, Jonathan Lopes, Marilda Siqueira

EPI_ISL_427305

EPI_ISL_427306

EPI_ISL_406531

EPI_ISL_406533

EPI_ISL_406534

hCoV-19/Brazil/SC-766/2020

hCoV-19/Brazil/SC-769/2020

hCoV-

19/Guangdong/20SF174/2020

hCoV-

19/Guangzhou/20SF206/2020

hCoV-

19/Foshan/20SF207/2020

South America / Brazil / Santa Catarina / Joinville

South America / Brazil / Santa Catarina / Florianopolis

Asia / China / Guangdong / Zhuhai

Asia / China /

Guangdong /

Guangzhou

Asia / China / Guangdong / Foshan

2020-03-10

2020-03-10

2020-01-22

2020-01-22

2020-01-22

LACEN-SC - Laboratorio Central de Santa Catarina


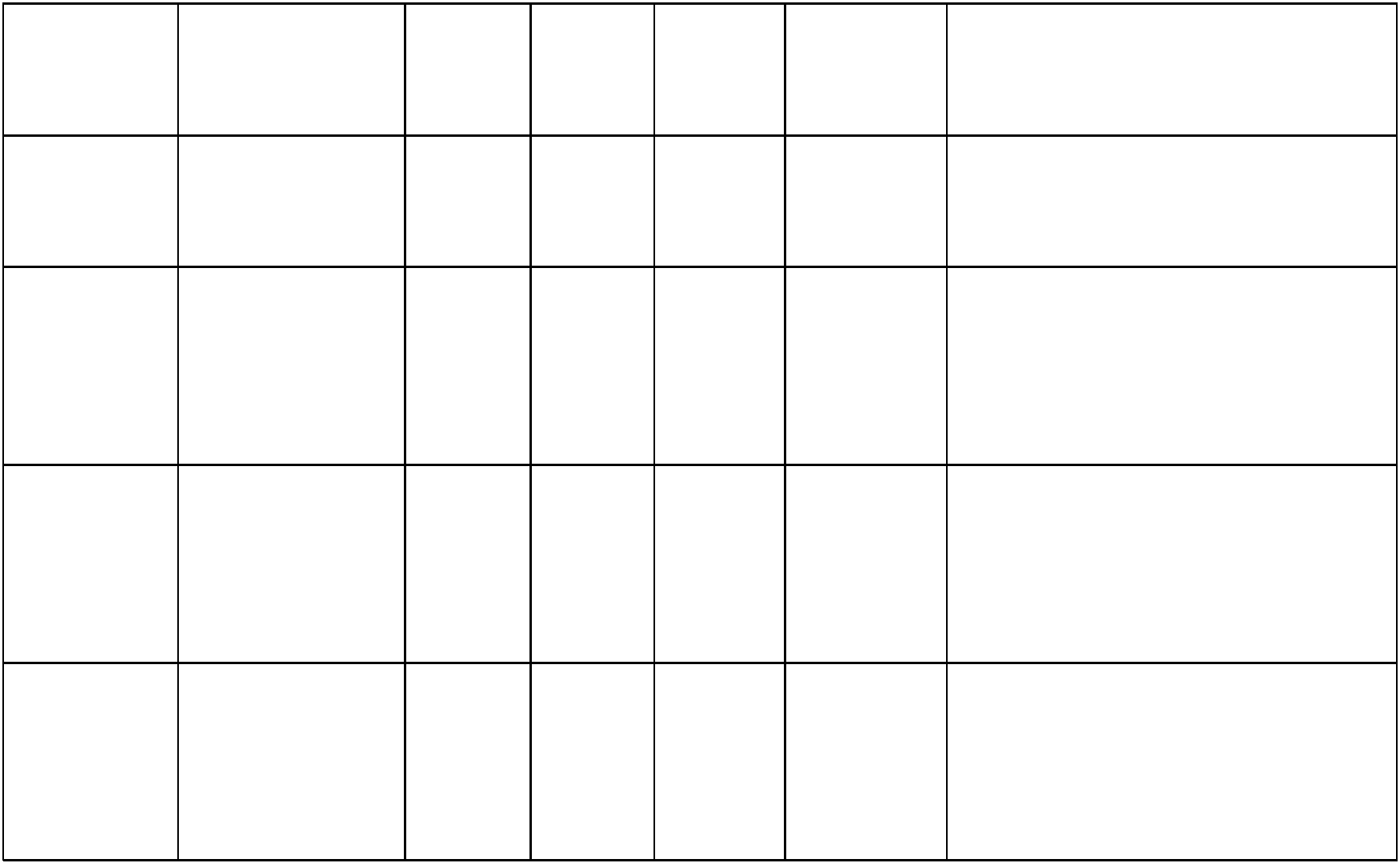


LACEN-SC - Laboratorio Central de Santa Catarina

Guangdong

Provincial Center for Diseases Contorl and Prevention; Guangdong Provinical Public Health

Guangdong

Provincial Center for Diseases Contorl and Prevention; Guangdong Provinical Public Health

Guangdong

Provincial Center for Diseases Control and Prevention; Guangdong Provincial Public Health

Instituto Oswaldo

Cruz FIOCRUZ -

Laboratory of

Respiratory Viruses

and Measles (LVRS)

Instituto Oswaldo

Cruz FIOCRUZ -

Laboratory of

Respiratory Viruses

and Measles (LVRS)

Guangdong

Provincial Center for Disease Control and Prevention

Guangdong Provincial Center for Diseases Control and Prevention

Guangdong Provincial Center for Diseases Control and Prevention

Paola Resende, Fernando Motta, Luciana Appolinario, Sunando Roy, Aline Mattos, Milene Miranda, Cristiana Garcia, Braulia Caetano, Maria Ogrzewalska, Priscila Born, Jonathan Lopes, Marilda Siqueira

Paola Resende, Fernando Motta, Luciana Appolinario, Sunando Roy, Aline Mattos, Milene Miranda, Cristiana Garcia, Braulia Caetano, Maria Ogrzewalska, Priscila Born, Jonathan Lopes, Marilda Siqueira

Min Kang, Jie Wu, Jing Lu, Tao Liu, Baisheng Li, Shujiang Mei, Feng Ruan, Lifeng Lin, Changwen Ke, Haojie Zhong, Yingtao Zhang, Lirong Zou, Xuguang Chen, Qi Zhu, Jianpeng Xiao, Jianxiang Geng, Zhe Liu, Jianxiong Hu, Weilin Zeng, Xing Li, Yuhuang Liao, Xiujuan Tang, Songjian Xiao, Ying Wang, Yingchao Song, Xue Zhuang, Lijun Liang, Guanhao He, Huihong Deng, Tie Song, Jianfeng He, Wenjun Ma

Min Kang, Jie Wu, Jing Lu, Tao Liu, Baisheng Li, Shujiang Mei, Feng Ruan, Lifeng Lin, Changwen Ke, Haojie Zhong, Yingtao Zhang, Lirong Zou, Xuguang Chen, Qi Zhu, Jianpeng Xiao, Jianxiang Geng, Zhe Liu, Jianxiong Hu, Weilin Zeng, Xing Li, Yuhuang Liao, Xiujuan Tang, Songjian Xiao, Ying Wang, Yingchao Song, Xue Zhuang, Lijun Liang, Guanhao He, Huihong Deng, Tie Song, Jianfeng He, Wenjun Ma

Min Kang, Jie Wu, Jing Lu, Tao Liu, Baisheng Li, Shujiang Mei, Feng Ruan, Lifeng Lin, Changwen Ke, Haojie Zhong, Yingtao Zhang, Lirong Zou, Xuguang Chen, Qi Zhu, Jianpeng Xiao, Jianxiang Geng, Zhe Liu, Jianxiong Hu, Weilin Zeng, Xing Li, Yuhuang Liao, Xiujuan Tang, Songjian Xiao, Ying Wang, Yingchao Song, Xue Zhuang, Lijun Liang, Guanhao He, Huihong Deng, Tie Song, Jianfeng He, Wenjun Ma

EPI_ISL_406535

EPI_ISL_406536

EPI_ISL_406538

EPI_ISL_406593

hCoV-

19/Foshan/20SF210/2020

hCoV-

19/Foshan/20SF211/2020

hCoV-

19/Guangdong/20SF201/2020

hCoV-19/Shenzhen/SZTH-

002/2020

Asia / China / Guangdong / Foshan

Asia / China / Guangdong / Foshan

Asia / China / Guangdong

Asia / China / Guandong / Shenzhen

2020-01-22

2020-01-22

2020-01-23

2020-01-13

Guangdong


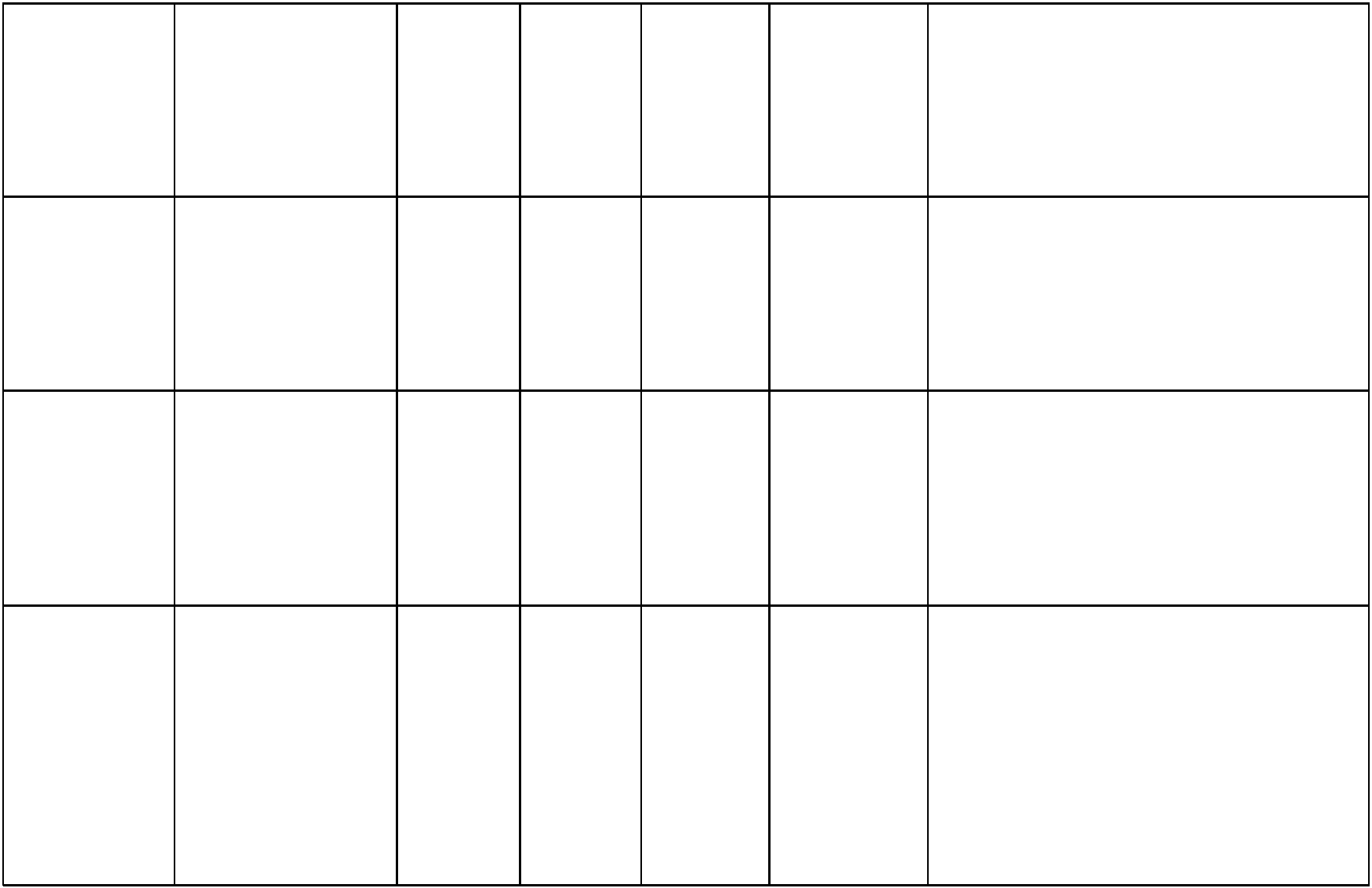


Provincial Center for Diseases Control and Prevention; Guangdong Provincial Public Health

Guangdong

Provincial Center for Diseases Control and Prevention; Guangdong Provincial Public Health

Guangdong

Provincial Center for Diseases Control and Prevention;Guan gdong Provincial Institute of Public Health

Shenzhen Key Laboratory of Pathogen and Immunity, National Clinical Research Center for Infectious Disease, Shenzhen Third People's Hospital

Guangdong Provincial Center for Diseases Control and Prevention

Guangdong Provincial Center for Diseases Control and Prevention

Guangdong Provincial Center for Diseases Control and Prevention

Shenzhen Key Laboratory of Pathogen and

Immunity, National Clinical Research Center for Infectious Disease, Shenzhen Third People's Hospital

Min Kang, Jie Wu, Jing Lu, Tao Liu, Baisheng Li, Shujiang Mei, Feng Ruan, Lifeng Lin, Changwen Ke, Haojie Zhong, Yingtao Zhang, Lirong Zou, Xuguang Chen, Qi Zhu, Jianpeng Xiao, Jianxiang Geng, Zhe Liu, Jianxiong Hu, Weilin Zeng, Xing Li, Yuhuang Liao, Xiujuan Tang, Songjian Xiao, Ying Wang, Yingchao Song, Xue Zhuang, Lijun Liang, Guanhao He, Huihong Deng, Tie Song, Jianfeng He, Wenjun Ma

Min Kang, Jie Wu, Jing Lu, Tao Liu, Baisheng Li, Shujiang Mei, Feng Ruan, Lifeng Lin, Changwen Ke, Haojie Zhong, Yingtao Zhang, Lirong Zou, Xuguang Chen, Qi Zhu, Jianpeng Xiao, Jianxiang Geng, Zhe Liu, Jianxiong Hu, Weilin Zeng, Xing Li, Yuhuang Liao, Xiujuan Tang, Songjian Xiao, Ying Wang, Yingchao Song, Xue Zhuang, Lijun Liang, Guanhao He, Huihong Deng, Tie Song, Jianfeng He, Wenjun Ma

Min Kang, Jie Wu, Jing Lu, Tao Liu, Baisheng Li, Shujiang Mei, Feng Ruan, Lifeng Lin, Changwen Ke, Haojie Zhong, Yingtao Zhang, Lirong Zou, Xuguang Chen, Qi Zhu, Jianpeng Xiao, Jianxiang Geng, Zhe Liu, Jianxiong Hu, Weilin Zeng, Xing Li, Yuhuang Liao, Xiujuan Tang, Songjian Xiao, Ying Wang, Yingchao Song, Xue Zhuang, Lijun Liang, Guanhao He, Huihong Deng, Tie Song, Jianfeng He, Wenjun Ma

Yang Yang, Chenguang Shen, Li Xing, Zhixiang Xu, Haixia Zheng, Yingxia Liu

EPI_ISL_406594

EPI_ISL_406596

EPI_ISL_406597

EPI_ISL_407893

hCoV-19/Shenzhen/SZTH-

003/2020

hCoV-

19/France/IDF0372/2020

hCoV-

19/France/IDF0373/2020

hCoV-

19/Australia/NSW01/2020

Asia / China / Guandong / Shenzhen

Europe /

France / Ile-de-

France / Paris

Europe /

France / Ile-de-

France / Paris

Oceania / Australia / New South Wales / Sydney

|  | Shenzhen Key |  |  |  |  |
| --- | --- | --- | --- | --- | --- |
|  | Laboratory of |  | Shenzhen Key |  |  |
|  | Pathogen and |  | Laboratory of |  |  |
|  | Immunity, |  | Pathogen and |  |  |
|  | National Clinical |  | Immunity, National | Yang Yang, Chenguang Shen, Li Xing, Zhixiang Xu, Haixia |  |
| 2020-01-16 | Research |  | Clinical Research |  |  |
|  |  |  |  | Zheng, Yingxia Liu |  |
|  | Center for |  | Center for Infectious |  |  |
|  |  |  |  |  |  |
|  | Infectious |  | Disease, Shenzhen |  |  |
|  | Disease, |  | Third People's |  |  |
|  | Shenzhen Third |  | Hospital |  |  |
|  | People's Hospital |  |  |  |  |
|  |  |  |  |  |  |
|  | Department of |  |  |  |  |
|  | Infectious and |  | National Reference | Mélanie Albert, Marion Barbet, Sylvie Behillil, Méline Bizard, |  |
|  | Tropical |  | Center for Viruses of |  |  |
| 2020-01-23 |  |  |  | Angela Brisebarre, Flora Donati, Vincent Enouf, Maud |  |
|  | Diseases, |  | Respiratory |  |  |
|  |  |  |  | Vanpeene, Sylvie van der Werf, Yazdan Yazdanpanah, |  |
|  | Bichat Claude |  | Infections, Institut |  |  |
|  |  |  |  | Xavier Lescure. |  |
|  | Bernard |  | Pasteur, Paris |  |  |
|  |  |  |  |  |  |
|  | Hospital, Paris |  |  |  |  |
|  |  |  |  |  |  |
|  | Department of |  |  |  |  |
|  | Infectious and |  | National Reference | Mélanie Albert, Marion Barbet, Sylvie Behillil, Méline Bizard, |  |
|  | Tropical |  | Center for Viruses of |  |  |
| 2020-01-23 |  |  |  | Angela Brisebarre, Flora Donati, Vincent Enouf, Maud |  |
|  | Diseases, |  | Respiratory |  |  |
|  |  |  |  | Vanpeene, Sylvie van der Werf, Yazdan Yazdanpanah, |  |
|  | Bichat Claude |  | Infections, Institut |  |  |
|  |  |  |  | Xavier Lescure. |  |
|  | Bernard |  | Pasteur, Paris |  |  |
|  |  |  |  |  |  |
|  | Hospital, Paris |  |  |  |  |
|  |  |  |  |  |  |
|  | Centre for |  | NSW Health |  |  |
|  |  |  | Pathology - Institute |  |  |
|  | Infectious |  |  | Eden J-S, Carter I, Rahman H, Holmes EC, Rockett R, |  |
|  |  |  | of Clinical Pathology |  |  |
|  | Diseases and |  |  |  |  |
| 2020-01-24 |  |  | and Medical | O’Sullivan MV, Sintchenko V, Chen SC, Maddocks S, Kok J |  |
|  | Microbiology |  |  |  |  |
|  |  |  | Research; | and Dwyer DE for the 2019-nCoV Study Group |  |
|  | Laboratory |  |  |  |  |
|  |  |  | Westmead Hospital; |  |  |
|  | Services |  |  |  |  |
|  |  |  | University of Sydney |  |  |
|  |  |  |  |  |  |
|  |  |  |  |  |  |


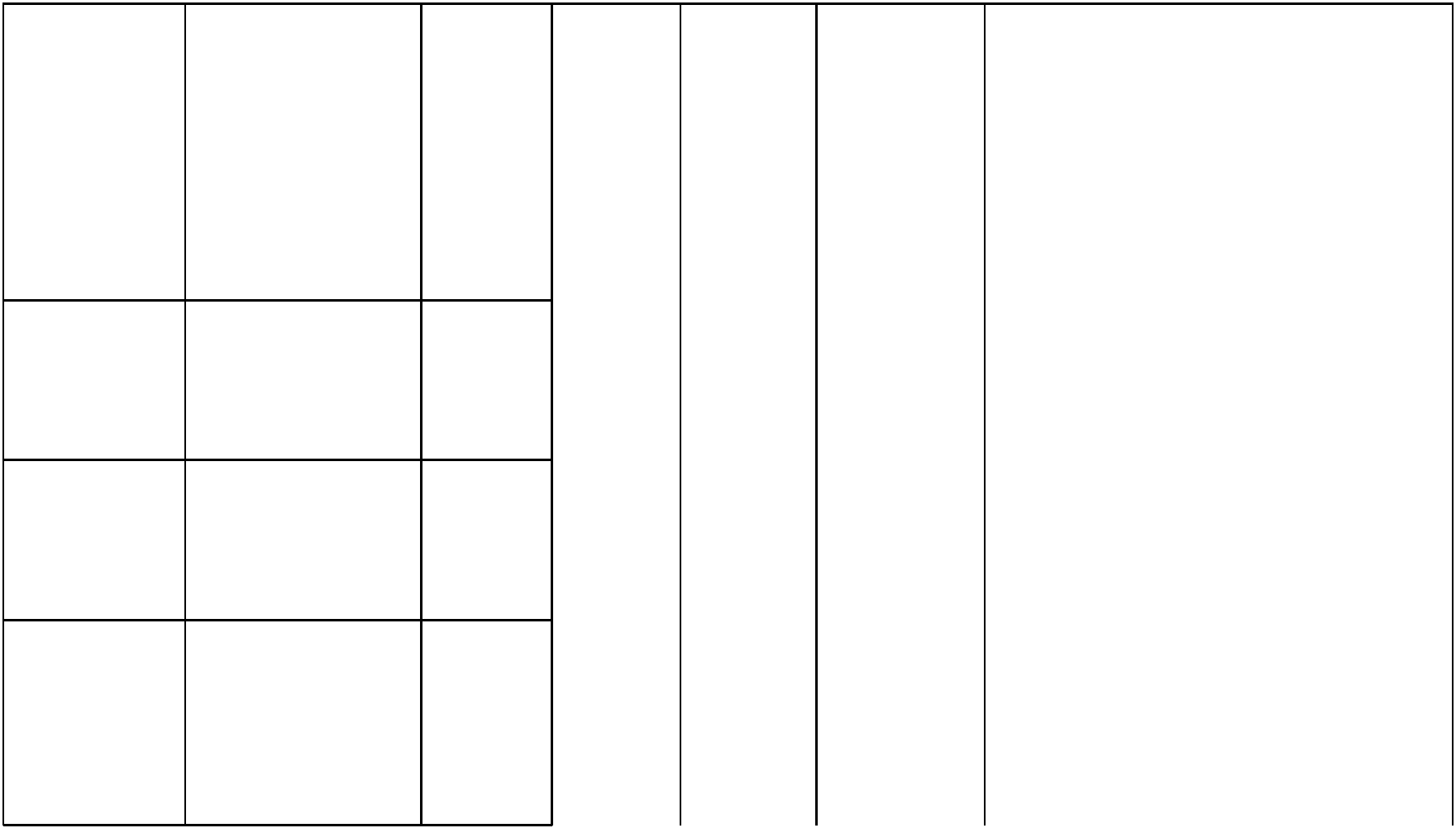


EPI_ISL_407894

EPI_ISL_407896

EPI_ISL_410531

EPI_ISL_410532

EPI_ISL_410535

EPI_ISL_410536

hCoV-

19/Australia/QLD01/2020

hCoV-

19/Australia/QLD02/2020

hCoV-19/Japan/NA-20-05-

1/2020

hCoV-19/Japan/OS-20-07-

1/2020

hCoV-19/Singapore/4/2020

hCoV-19/Singapore/5/2020

Oceania / Australia / Queensland / Gold Coast

Oceania / Australia / Queensland / Gold Coast

Asia / Japan /

Nara

Asia / Japan /

Osaka

Asia / Singapore

Asia / Singapore

2020-01-28

2020-01-30

2020-01-25

2020-01-23

2020-02-03

2020-02-06

|  |  |  | Ben Huang, Alyssa Pyke, Amanda De Jong, Andrew Van |  |
| --- | --- | --- | --- | --- |
|  |  |  | Den Hurk, Carmel Taylor, David Warrilow, Doris Genge, |  |
| Pathology |  | Public Health | Elisabeth Gamez, Glen Hewitson, Ian Maxwell Mackay, Inga |  |
|  |  |  | Sultana, Jamie McMahon, Jean Barcelon, Judy Northill, |  |
| Queensland |  | Virology Laboratory |  |  |
|  |  |  | Mitchell Finger, Natalie Simpson, Neelima Nair, Peter |  |
|  |  |  |  |  |
|  |  |  | Burtonclay, Peter Moore, Sarah Wheatley, Sean Moody, |  |
|  |  |  | Sonja Hall-Mendelin, Timothy Gardam, and Frederick Moore. |  |
|  |  |  |  |  |
|  |  |  | Ben Huang, Alyssa Pyke, Amanda De Jong, Andrew Van |  |
|  |  |  | Den Hurk, Carmel Taylor, David Warrilow, Doris Genge, |  |
| Pathology |  | Public Health | Elisabeth Gamez, Glen Hewitson, Ian Maxwell Mackay, Inga |  |
|  |  |  | Sultana, Jamie McMahon, Jean Barcelon, Judy Northill, |  |
| Queensland |  | Virology Laboratory |  |  |
|  |  |  | Mitchell Finger, Natalie Simpson, Neelima Nair, Peter |  |
|  |  |  |  |  |
|  |  |  | Burtonclay, Peter Moore, Sarah Wheatley, Sean Moody, |  |
|  |  |  | Sonja Hall-Mendelin, Timothy Gardam, and Frederick Moore. |  |
|  |  |  |  |  |
| Dept. of |  |  |  |  |
| Pathology, |  | Pathogen Genomics | Tsuyoshi Sekizuka, Harutaka Katano, Shutoku Matsuyama, |  |
| National |  | Center, National | Naganori Nao, Kazuya Shirato, Motoi Suzuki, Hideki |  |
| Institute of |  | Institute of Infectious | Hasegawa, Takaji Wakita, Makoto Takeda, Tadaki Suzuki, |  |
| Infectious |  | Diseases | Makoto Kuroda |  |
| Diseases |  |  |  |  |
|  |  |  |  |  |
| Dept. of |  | Pathogen Genomics | Tsuyoshi Sekizuka, Harutaka Katano, Shutoku Matsuyama, |  |
| Pathology, |  |  |  |  |
| National |  | Center, National | Naganori Nao, Kazuya Shirato, Motoi Suzuki, Hideki |  |
| Institute of |  | Institute of Infectious | Hasegawa, Takaji Wakita, Makoto Takeda, Tadaki Suzuki, |  |
| Infectious |  | Diseases | Makoto Kuroda |  |
| Diseases |  |  |  |  |
|  |  |  |  |  |
| National Centre |  | Programme in | Danielle E Anderson, Martin Linster, Yan Zhuang, Jayanthi |  |
|  |  | Emerging Infectious |  |  |
| for Infectious |  |  | Jayakumar, David CB Lye, Yee Sin Leo, Barnaby E Young, |  |
|  |  | Diseases, Duke- |  |  |
| Diseases |  |  | Yvonne CF Su, Gavin JD Smith |  |
|  |  | NUS Medical School |  |  |
|  |  |  |  |  |
|  |  |  |  |  |
| Singapore |  |  |  |  |
| General |  | Programme in |  |  |
| Hospital, |  |  | Danielle E Anderson, Martin Linster, Yan Zhuang, Jayanthi |  |
|  |  | Emerging Infectious |  |  |
| Molecular |  |  | Jayakumar, Kian Sing Chan, Lynette LE Oon, Shirin |  |
|  |  | Diseases, Duke- |  |  |
| Laboratory, |  |  | Kalimuddin, Jenny GH Low, Yvonne CF Su, Gavin JD Smith |  |
|  |  | NUS Medical School |  |  |
| Division of |  |  |  |  |
|  |  |  |  |  |
| Pathology |  |  |  |  |
|  |  |  |  |  |


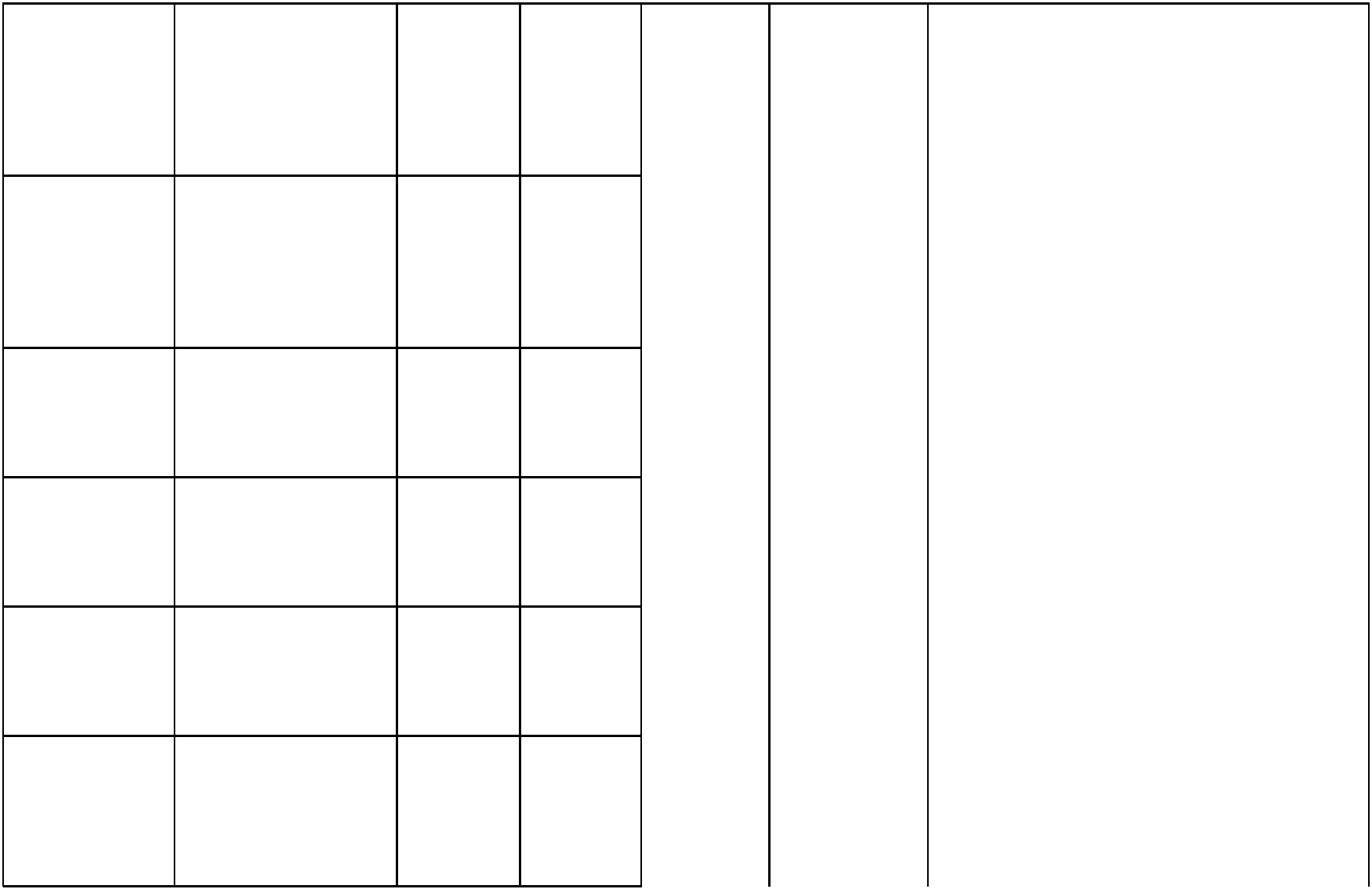


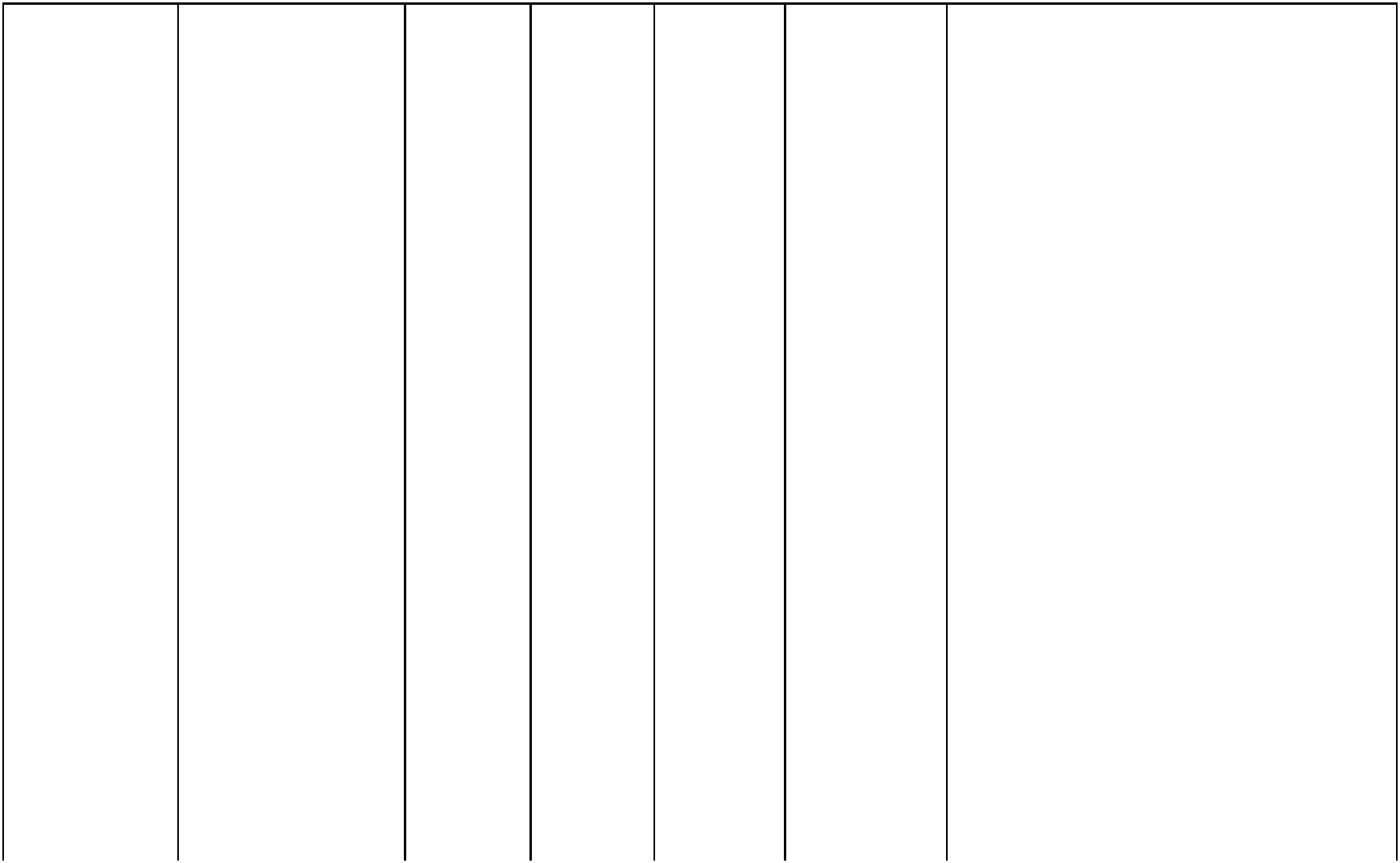


|  |  |  |  |  |  |  | Singapore |  |  |  |  |
| --- | --- | --- | --- | --- | --- | --- | --- | --- | --- | --- | --- |
|  |  |  |  |  |  |  | General |  | Programme in |  |  |
|  |  |  |  |  |  |  | Hospital, |  |  | Danielle E Anderson, Martin Linster, Yan Zhuang, Jayanthi |  |
| EPI_ISL_410537 |  | hCoV-19/Singapore/6/2020 |  | Asia / Singapore | 2020-02-09 | |  |  | Emerging Infectious |  |  |
|  |  |  |  |  |  |  | Molecular |  |  | Jayakumar, Kian Sing Chan, Lynette LE Oon, Shirin |  |
|  |  |  |  |  |  |  |  |  | Diseases, Duke- |  |  |
|  |  |  |  |  |  |  | Laboratory, |  |  | Kalimuddin, Jenny GH Low, Yvonne CF Su, Gavin JD Smith |  |
|  |  |  |  |  |  |  |  |  | NUS Medical School |  |  |
|  |  |  |  |  |  |  | Division of |  |  |  |  |
|  |  |  |  |  |  |  |  |  |  |  |  |
|  |  |  |  |  |  |  | Pathology |  |  |  |  |
|  |  |  |  |  |  |  |  |  |  |  |  |
|  |  |  |  |  |  |  |  |  | Virology Unit, Institut |  |  |
|  |  |  |  |  |  |  |  |  | Pasteur du |  |  |
|  |  |  |  |  |  |  |  |  | Cambodge |  |  |
|  |  |  |  |  |  |  |  |  | (Sequencing done |  |  |
|  |  |  |  |  |  |  |  |  | by: Jessica E |  |  |
|  |  |  |  |  |  |  |  |  | Manning/Jennifer A |  |  |
|  |  |  |  | Asia / |  |  | Virology Unit, |  | Bohl at Malaria and | Erik A Karlsson, Jennifer A Bohl, Vida Ahyong, Veasna |  |
| EPI_ISL_411902 |  | hCoV-19/Cambodia/0012/2020 |  | Cambodia / | 2020-01-27 | | Institut Pasteur |  | Vector Research |  |  |
|  |  |  |  |  |  |  |  |  |  | Duong, Philippe Dussart, Jessica E Manning. |  |
|  |  |  |  | Sihanoukville |  |  | du Cambodge. |  | Research |  |  |
|  |  |  |  |  |  |  |  |  |  |  |  |
|  |  |  |  |  |  |  |  |  | Laboratory, National |  |  |
|  |  |  |  |  |  |  |  |  | Institute of Allergy |  |  |
|  |  |  |  |  |  |  |  |  | and Infectious |  |  |
|  |  |  |  |  |  |  |  |  | Diseases and Vida |  |  |
|  |  |  |  |  |  |  |  |  | Ahyong from Chan- |  |  |
|  |  |  |  |  |  |  |  |  | Zuckerberg Biohub) |  |  |
|  |  |  |  |  |  |  |  |  |  |  |  |
|  |  |  |  |  |  |  |  |  | Department of |  |  |
|  |  | hCoV-19/Taiwan/CGMH-CGU- |  | Asia / Taiwan / |  |  | Laboratory |  | Laboratory Medicine, | Kuo-Chien Tsao, Yu-Nong Gong, Shu-Li Yang, Yi-Chun Li, |  |
| EPI_ISL_411915 |  |  |  |  | 2020-01-25 | |  |  | Lin-Kou Chang Gung |  |  |
|  | 01/2020 | |  | Taoyuan |  |  | Medicine |  |  | Chung-Guei Huang, Yhu-Chering Huang, Shin-Ru Shih |  |
|  |  |  |  |  |  |  |  |  | Memorial Hospital, |  |  |
|  |  |  |  |  |  |  |  |  |  |  |  |
|  |  |  |  |  |  |  |  |  | Taoyuan, Taiwan. |  |  |
|  |  |  |  |  |  |  |  |  |  |  |  |
|  |  |  |  | Asia / Taiwan / |  |  | Taiwan Centers |  | Taiwan Centers for |  |  |
| EPI_ISL_411926 |  | hCoV-19/Taiwan/3/2020 |  |  | 2020-01-24 | | for Disease |  |  | Ji-Rong Yang, Yu-Chi-Lin, Jung-Jung Mu, Ming-Tsan-Liu |  |
|  |  |  |  | Taipei |  |  |  |  | Disease Control |  |  |
|  |  |  |  |  |  |  | Control |  |  |  |  |
|  |  |  |  |  |  |  |  |  |  |  |  |
|  |  |  |  |  |  |  |  |  |  |  |  |
|  |  |  |  | Asia / Taiwan / |  |  | Taiwan Centers |  | Taiwan Centers for |  |  |
| EPI_ISL_411927 |  | hCoV-19/Taiwan/4/2020 |  |  | 2020-01-28 | | for Disease |  |  | Ji-Rong Yang, Yu-Chi-Lin, Jung-Jung Mu, Ming-Tsan-Liu |  |
|  |  |  |  | Taipei |  |  |  |  | Disease Control |  |  |
|  |  |  |  |  |  |  | Control |  |  |  |  |
|  |  |  |  |  |  |  |  |  |  |  |  |
|  |  |  |  |  |  |  |  |  |  |  |  |

Asia / China /

EPI_ISL_411950 hCoV-19/Jiangsu/JS01/2020 _Jiangsu_

| EPI_ISL_411951 |  | hCoV-19/Sweden/01/2020 |  | Europe / |  |
| --- | --- | --- | --- | --- | --- |
|  |  |  |  | Sweden |  |
|  |  |  |  |  |  |
|  |  |  |  |  |  |

Asia / China /

EPI_ISL_411952 hCoV-19/Jiangsu/JS02/2020 _Jiangsu_

| EPI_ISL_411953 | hCoV-19/Jiangsu/JS03/2020 | Asia / China / |  |
| --- | --- | --- | --- |
|  |  | Jiangsu |  |
|  |  |  |  |

2020-01-23

2020-02-07

2020-01-24

2020-01-24

NHC Key laboratory of Enteric Pathogenic Microbiology,


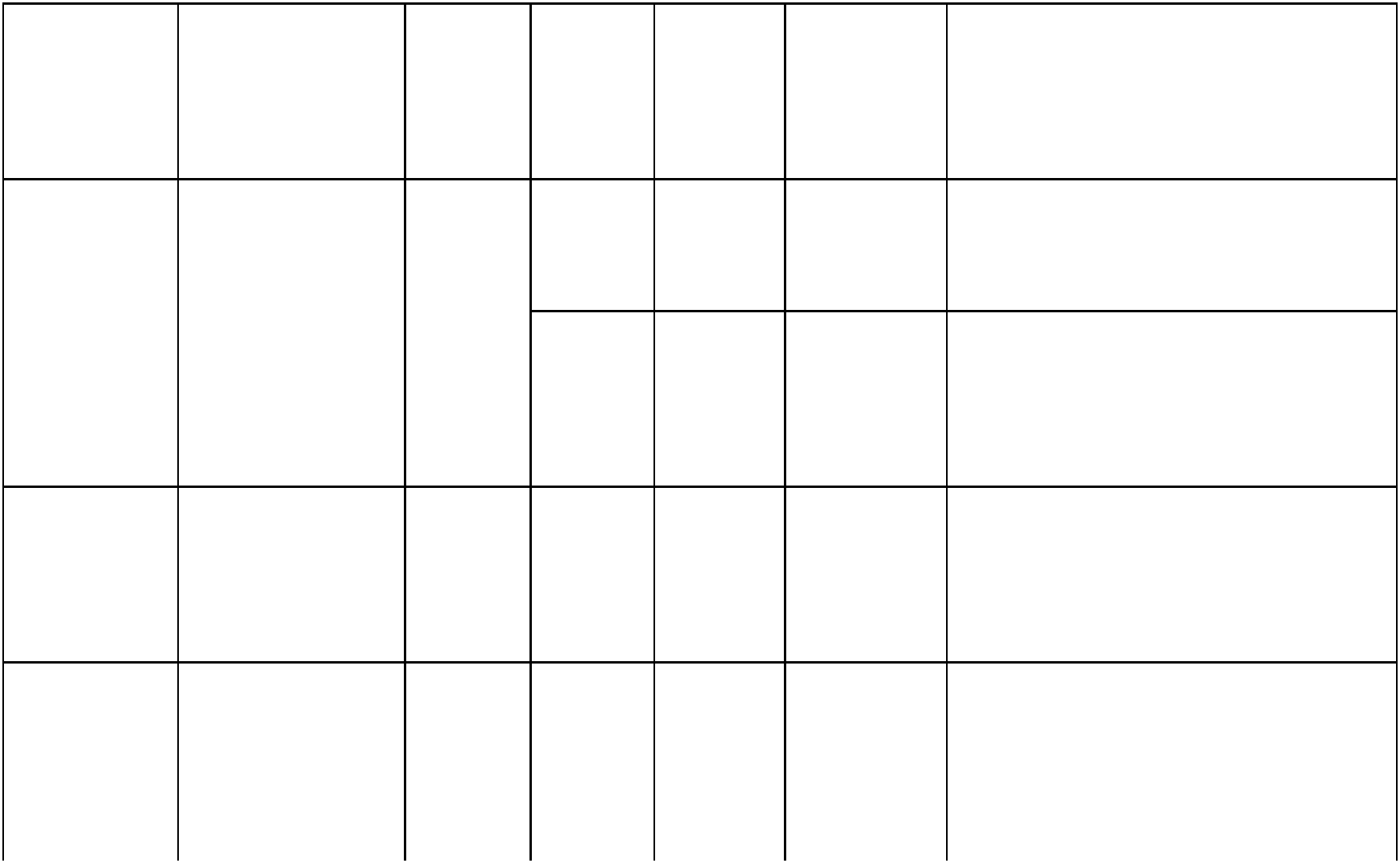


Institute of Pathogenic Microbiology

unknown

NHC Key laboratory of Enteric Pathogenic Microbiology,

Institute of Pathogenic Microbiology

NHC Key laboratory of Enteric Pathogenic Microbiology,

Institute of Pathogenic Microbiology

Jiangsu Provincial Center for Disease Control & Prevention

Unit for Laboratory Development and Technology Transfer, Public Health Agency of Sweden

Jiangsu Provincial Center for Disease Control & Prevention

Jiangsu Provincial Center for Disease Control & Prevention

Lunbiao Cui,Kangchen Zhao,Xiaojuan Zhu,Yiyue Ge,Tao Wu,Bin Wu,Yin Chen,Fengcai Zhu,Baoli Zhu,Ming Wu

Bengner,M., Palmerus,M., Lindsjo,O., Lind Karlberg,M., Monteil,V., Appelberg,S., Brave,A., Muradrasoli,S. and Tegmark-Wisell,K.

Kangchen Zhao, Xiaojuan Zhu, Lunbiao Cui, Tao Wu, Yiyue Ge, Bin Wu, Yin Chen, Fengcai Zhu, Baoli Zhu, Ming Wu

Kangchen Zhao, Xiaojuan Zhu, Lunbiao Cui, Tao Wu, Yiyue Ge, Bin Wu, Yin Chen, Fengcai Zhu, Baoli Zhu, Ming Wu

|  |  |  |  |  |  |  |  |  | Pathogen Discovery, |  |  |
| --- | --- | --- | --- | --- | --- | --- | --- | --- | --- | --- | --- |
|  |  |  |  |  |  |  |  |  | Respiratory Viruses | Krista Queen, Anna Uehara, Jing Zhang, Yan Li, Ying Tao, |  |
|  |  |  |  | North America / |  |  | California |  | Branch, Division of | Clinton R. Paden, Haibin Wang, Shifaq Kamili, Xiaoyan Lu, |  |
| EPI_ISL_411954 |  | hCoV-19/USA/CA7/2020 |  |  | 2020-02-06 | | Department of |  | Viral Diseases, | Brian Lynch, Senthil Kumar K. Sakthivel, Brett L. Whitaker, |  |
|  |  |  |  | USA / California |  |  |  |  |  |  |  |
|  |  |  |  |  |  |  | Public Health |  | Centers for Dieases | Lijuan Wang, Janna' R. Murray, Susan I. Gerber, Stephen |  |
|  |  |  |  |  |  |  |  |  |  |  |  |
|  |  |  |  |  |  |  |  |  | Control and | Lindstrom, Suxiang Tong |  |
|  |  |  |  |  |  |  |  |  | Prevention |  |  |
|  |  |  |  |  |  |  |  |  |  |  |  |

EPI_ISL_411955

EPI_ISL_411956

EPI_ISL_411957

EPI_ISL_413518

EPI_ISL_413519

EPI_ISL_413520

EPI_ISL_413521

EPI_ISL_413523

hCoV-19/USA/CA8/2020

hCoV-19/USA/TX1/2020

hCoV-19/China/WH-09/2020

hCoV-19/Beijing/105/2020

hCoV-19/Beijing/231/2020

hCoV-19/Beijing/233/2020

hCoV-19/Beijing/235/2020

hCoV-19/India/1-31/2020

North America / USA / California

North America / USA / Texas

Asia / China

Asia / China /

Beijing

Asia / China /

Beijing

Asia / China /

Beijing

Asia / China /

Beijing

Asia / India /

Kerala

2020-02-10

2020-02-11

2020-01-08

2020-01-26

2020-01-28

2020-01-28

2020-01-28

2020-01-31

California Department of Public Health


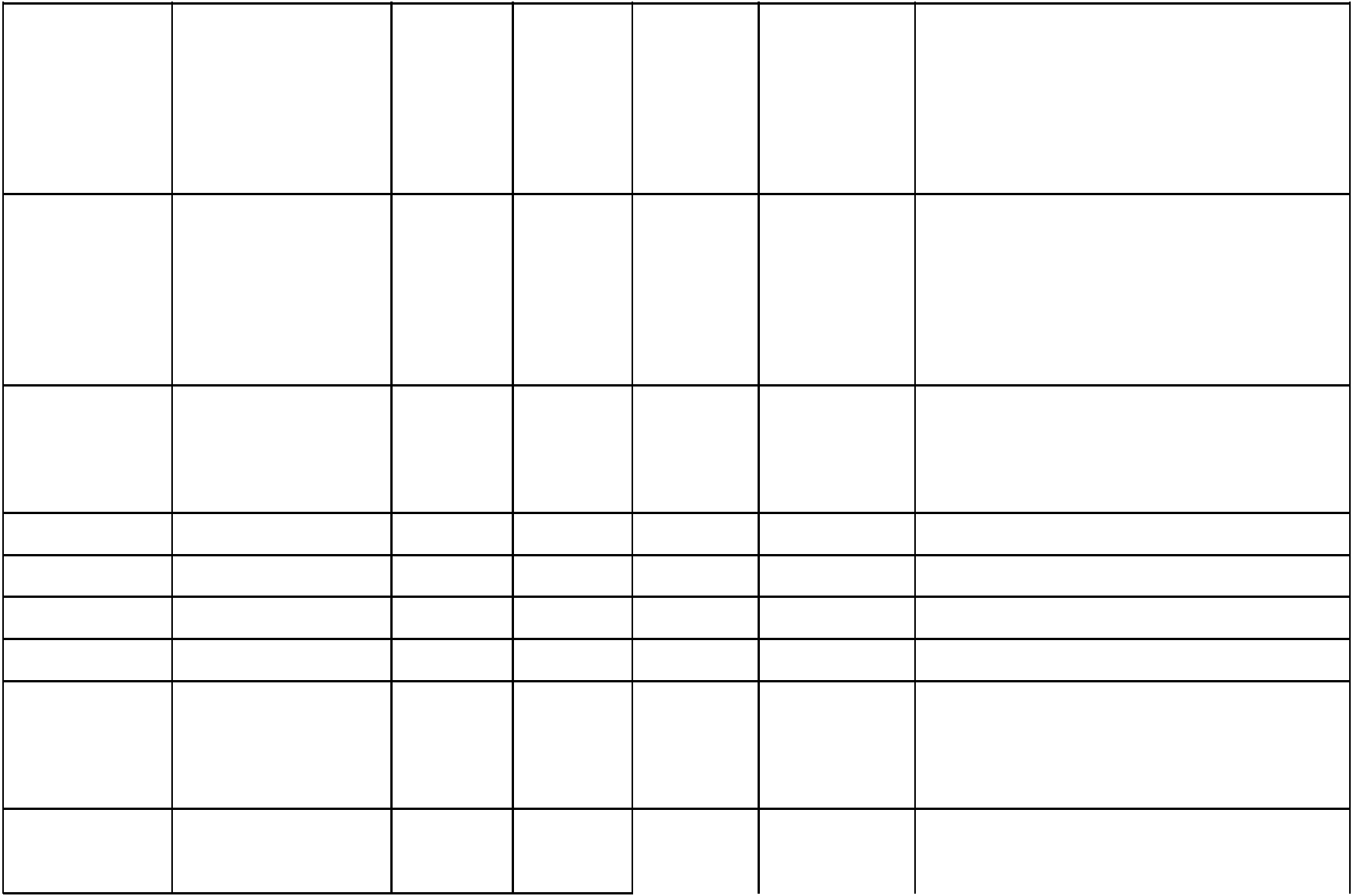


Texas

Department of

State Health

Services

unknown

unknown

unknown

unknown

unknown

Indian Council

of Medical

Research-

National

Institute of

Virology

Pathogen Discovery, Respiratory Viruses Branch, Division of Viral Diseases, Centers for Dieases Control and Prevention

Pathogen Discovery, Respiratory Viruses Branch, Division of Viral Diseases, Centers for Dieases Control and Prevention

Key Laboratory of

Human Diseases,

Comparative

Medicine, Institute of

Laboratory Animal

Science

Infectious Disease

Control Center

Infectious Disease

Control Center

Infectious Disease

Control Center

Infectious Disease

Control Center

National Influenza Center, Indian Council of Medical Research-National Institute of Virology

Krista Queen, Anna Uehara, Jing Zhang, Yan Li, Ying Tao, Clinton R. Paden, Haibin Wang, Shifaq Kamili, Xiaoyan Lu, Brian Lynch, Senthil Kumar K. Sakthivel, Brett L. Whitaker, Lijuan Wang, Janna' R. Murray, Susan I. Gerber, Stephen Lindstrom, Suxiang Tong

Krista Queen, Anna Uehara, Jing Zhang, Yan Li, Ying Tao, Clinton R. Paden, Haibin Wang, Shifaq Kamili, Xiaoyan Lu, Brian Lynch, Senthil Kumar K. Sakthivel, Brett L. Whitaker, Lijuan Wang, Janna' R. Murray, Susan I. Gerber, Stephen Lindstrom, Suxiang Tong

Linlin,B., Lili,R., Shuran,G., Jiangning,L., Feifei,Q., Qi,L., Fengdi,L., Jing,X., Wei,D., Pin,Y., Yanfeng,X., Yajin,Q., Hong,G., Qiang,W., Mingya,L., Guanpeng,W., Shunyi,W., Zhiqi,S., Li,G., Lan,C., Conghui,W., Ying,W., Xinming,W., Yan,X., Qi,J. and Chuan,Q.

Li,J., Li,L., Li,Z., Qiu,S., Song,H., Li,P. and Li,P.

Li,J., Li,L., Li,Z., Qiu,S., Song,H., Li,P. and Li,P.

Li,J., Li,L., Li,Z., Qiu,S., Song,H., Li,P. and Li,P.

Li,J., Li,L., Li,Z., Qiu,S., Song,H., Li,P. and Li,P.

Potdar V, Yadav PD, Choudhary ML, Shete-Aich A

EPI_ISL_413555

hCoV-19/Wales/PHW1/2020

Europe / United Kingdom / Wales

2020-02-27

| Wales Specialist |  | Public Health Wales | Catherine Moore, Cen Sabu, Joanne Watkins, Sally Corden, |
| --- | --- | --- | --- |
| Virology Centre |  | Microbiology Cardiff | Tom Connor |
|  |  |  |  |

EPI_ISL_413556

EPI_ISL_413557

EPI_ISL_413558

EPI_ISL_413559

EPI_ISL_413560

EPI_ISL_413561

EPI_ISL_413566

EPI_ISL_413571

hCoV-19/Wales/PHW2/2020

hCoV-19/USA/CA-CDPH-

UC1/2020

hCoV-19/USA/CA-CDPH-

UC2/2020

hCoV-19/USA/CA-CDPH-

UC3/2020

hCoV-19/USA/WA-S3/2020

hCoV-19/USA/CA-CDPH-

UC4/2020

hCoV-

19/Netherlands/Blaricum_1364

780/2020

hCoV-

19/Netherlands/Eindhoven_13

63782/2020

Europe / United Kingdom / Wales

North America / USA / California

- Sonoma County

North America /

USA / California

/ Solano County

North America /

USA / California

/ Solano County

North America /

USA /

Washington

North America /

USA / California

/ Solano County

Europe / Netherlands / Blaricum

Europe / Netherlands / Eindhoven

2020-03-04

2020-02-28

2020-02-27

2020-02-27

2020-02-28

2020-02-27

2020-03-02

2020-03-02

Wales Specialist Virology Centre


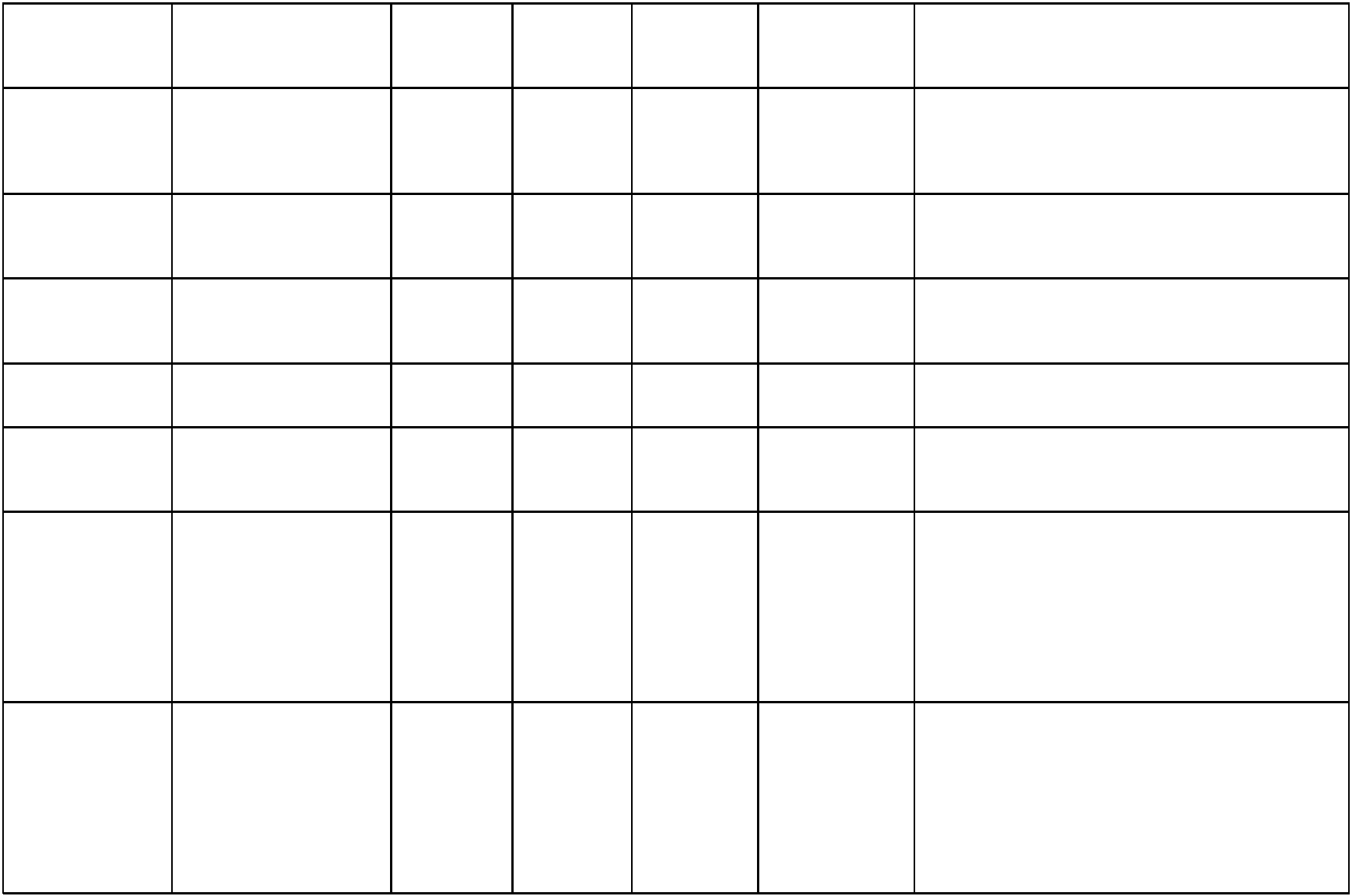


California Department of Public Health

California Department of Public Health

California Department of Public Health

Seattle Flu Study

California Department of Public Health

MHC Gooi & Vechtstreek

MHC Brabant

Zuidoost

Public Health Wales Microbiology Cardiff

Chiu Laboratory, University of California, San Francisco

Chiu Laboratory,

University of

California, San

Francisco

Chiu Laboratory, University of California, San Francisco

Seattle Flu Study

Chiu Laboratory,

University of

California, San

Francisco

Erasmus Medical

Center

Erasmus Medical

Center

Catherine Moore, Tim Jones, Joanne Watkins, Sally Corden, Tom Connor

Xianding Deng, Scot Federman, Chao-Yang Pan, Hugo Guevara,Wei Gu, Debra A. Wadford, and Charles Y. Chiu

Xianding Deng, Scot Federman, Chao-Yang Pan, Hugo Guevara,Wei Gu, Debra A. Wadford, and Charles Y. Chiu

Xianding Deng, Scot Federman, Chao-Yang Pan, Hugo Guevara,Wei Gu, Debra A. Wadford, and Charles Y. Chiu

Chu et al

Xianding Deng, Scot Federman, Chao-Yang Pan, Hugo Guevara,Wei Gu, Debra A. Wadford, and Charles Y. Chiu

David Nieuwenhuijse, Bas Oude Munnink, Reina Sikkema, Claudia Schapendonk, Irina Chestakova, Anne van der Linden, Mark Pronk, Pascal Lexmond, Corien Swaan, Manon Haverkate, Madelief Mollers, Mart Stein, Sandra Kengne Kamga Mobou, Jeroen van Kampen, Jolanda Voermans, Aura Timen, Corine GeurtsvanKessel, Annemiek van der Eijk, Richard Molenkamp, Marion Koopmans, on behalf of the Dutch national COVID-19 response team.

David Nieuwenhuijse, Bas Oude Munnink, Reina Sikkema, Claudia Schapendonk, Irina Chestakova, Anne van der Linden, Mark Pronk, Pascal Lexmond, Corien Swaan, Manon Haverkate, Madelief Mollers, Mart Stein, Sandra Kengne Kamga Mobou, Jeroen van Kampen, Jolanda Voermans, Aura Timen, Corine GeurtsvanKessel, Annemiek van der Eijk, Richard Molenkamp, Marion Koopmans, on behalf of the Dutch national COVID-19 response team.

|  | hCoV- | Europe / |  |
| --- | --- | --- | --- |
| EPI_ISL_413572 | 19/Netherlands/Haarlem_1363 | Netherlands / | 2020-03-01 |
|  | 688/2020 | Haarlem |  |

| EPI_ISL_413574 | hCoV- | Europe / | 2020-02-29 |  |
| --- | --- | --- | --- | --- |
|  | 19/Netherlands/Helmond_136 | Netherlands / |  |  |
|  | 3548/2020 | Helmond |  |  |

|  | hCoV- | Europe / |  |
| --- | --- | --- | --- |
| EPI_ISL_413579 | 19/Netherlands/Nootdorp_136 | Netherlands / | 2020-03-03 |
|  | 4222/2020 | Nootdorp |  |

| EPI_ISL_413584 |  | hCoV- |  | Europe / | 2020-03-03 | |  |
| --- | --- | --- | --- | --- | --- | --- | --- |
|  |  | 19/Netherlands/Rotterdam_13 |  | Netherlands / |  |  |  |
|  | 64740/2020 | |  | Rotterdam |  |  |  |
|  |  |  |  |  |  |  |  |

|  |  |  | David Nieuwenhuijse, Bas Oude Munnink, Reina Sikkema, |  |
| --- | --- | --- | --- | --- |
|  |  |  | Claudia Schapendonk, Irina Chestakova, Anne van der |  |
| MHC |  |  | Linden, Mark Pronk, Pascal Lexmond, Corien Swaan, Manon |  |
|  |  | Erasmus Medical | Haverkate, Madelief Mollers, Mart Stein, Sandra Kengne |  |
| Kennemerland |  | Center | Kamga Mobou, Jeroen van Kampen, Jolanda Voermans, |  |
|  |  |  | Aura Timen, Corine GeurtsvanKessel, Annemiek van der |  |
|  |  |  | Eijk, Richard Molenkamp, Marion Koopmans, on behalf of the |  |
|  |  |  | Dutch national COVID-19 response team. |  |
|  |  |  |  |  |
|  |  |  | David Nieuwenhuijse, Bas Oude Munnink, Reina Sikkema, |  |
|  |  |  | Claudia Schapendonk, Irina Chestakova, Anne van der |  |
|  |  |  | Linden, Mark Pronk, Pascal Lexmond, Corien Swaan, Manon |  |
| MHC West- |  | Erasmus Medical | Haverkate, Madelief Mollers, Mart Stein, Sandra Kengne |  |
| Brabant |  | Center | Kamga Mobou, Jeroen van Kampen, Jolanda Voermans, |  |
|  |  |  | Aura Timen, Corine GeurtsvanKessel, Annemiek van der |  |
|  |  |  | Eijk, Richard Molenkamp, Marion Koopmans, on behalf of the |  |
|  |  |  | Dutch national COVID-19 response team. |  |
|  |  |  |  |  |
|  |  |  | David Nieuwenhuijse, Bas Oude Munnink, Reina Sikkema, |  |
|  |  |  | Claudia Schapendonk, Irina Chestakova, Anne van der |  |
| MHC |  | Erasmus Medical | Linden, Mark Pronk, Pascal Lexmond, Corien Swaan, Manon |  |
|  |  |  | Haverkate, Madelief Mollers, Mart Stein, Sandra Kengne |  |
| Haaglanden |  | Center | Kamga Mobou, Jeroen van Kampen, Jolanda Voermans, |  |
|  |  |  | Aura Timen, Corine GeurtsvanKessel, Annemiek van der |  |
|  |  |  | Eijk, Richard Molenkamp, Marion Koopmans, on behalf of the |  |
|  |  |  | Dutch national COVID-19 response team. |  |
|  |  |  |  |  |
|  |  |  | David Nieuwenhuijse, Bas Oude Munnink, Reina Sikkema, |  |
|  |  |  | Claudia Schapendonk, Irina Chestakova, Anne van der |  |
|  |  |  | Linden, Mark Pronk, Pascal Lexmond, Corien Swaan, Manon |  |
| unknown |  | Erasmus Medical | Haverkate, Madelief Mollers, Mart Stein, Sandra Kengne |  |
|  |  | Center | Kamga Mobou, Jeroen van Kampen, Jolanda Voermans, |  |
|  |  |  |  |  |
|  |  |  | Aura Timen, Corine GeurtsvanKessel, Annemiek van der |  |
|  |  |  | Eijk, Richard Molenkamp, Marion Koopmans, on behalf of the |  |
|  |  |  | Dutch national COVID-19 response team. |  |
|  |  |  |  |  |


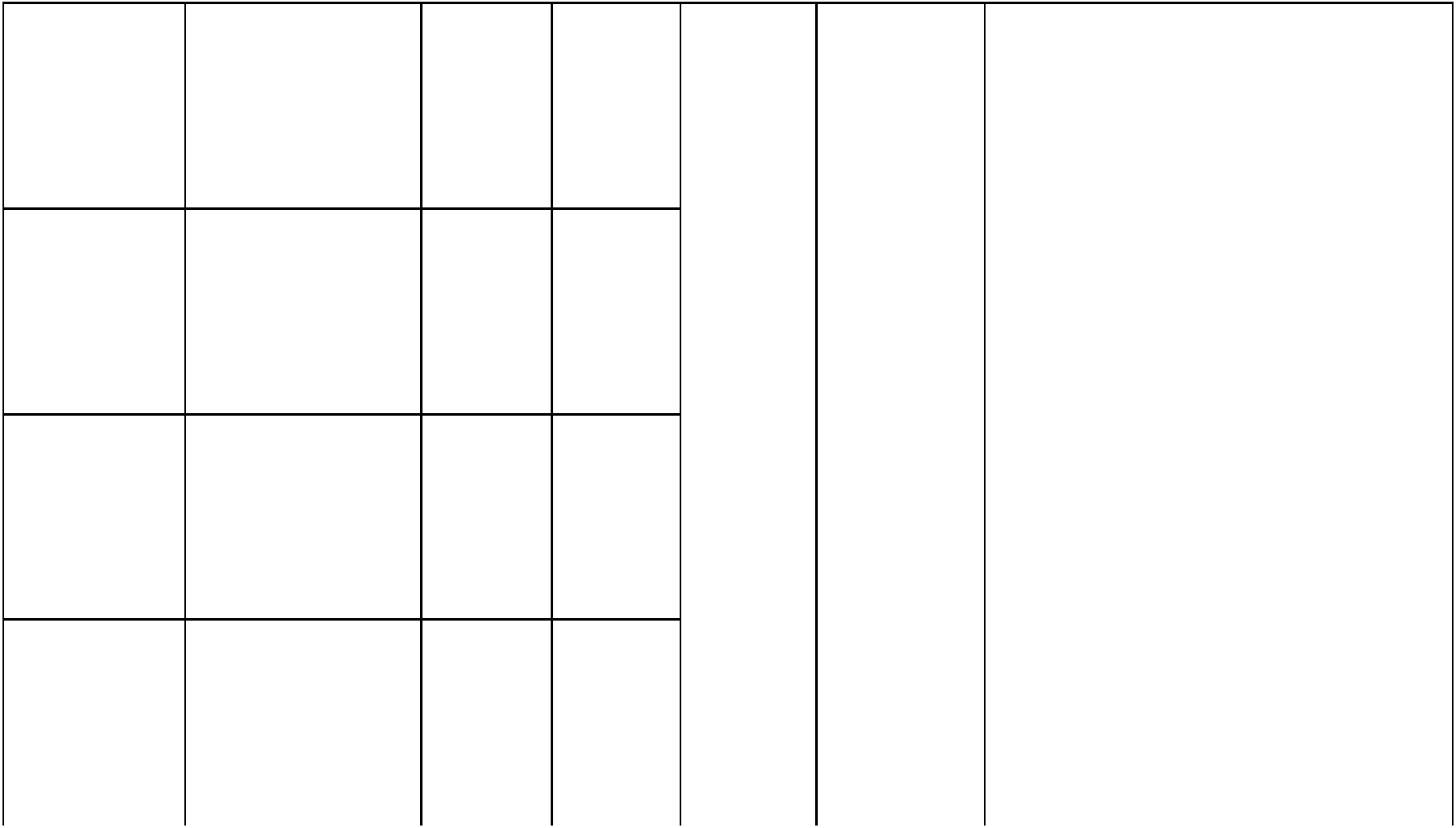


|  | hCoV- | Europe / |  |
| --- | --- | --- | --- |
| EPI_ISL_413587 | 19/Netherlands/Tilburg_13642 | Netherlands / | 2020-03-03 |
|  | 86/2020 | Tilburg |  |

| EPI_ISL_413588 | hCoV- | Europe / | 2020-03-01 |  |
| --- | --- | --- | --- | --- |
|  | 19/Netherlands/Utrecht_13635 | Netherlands / |  |  |
|  | 64/2020 | Utrecht |  |  |

|  | hCoV- | Europe / |  |
| --- | --- | --- | --- |
| EPI_ISL_413589 | 19/Netherlands/Utrecht_13636 | Netherlands / | 2020-03-01 |
|  | 28/2020 | Utrecht |  |

| EPI_ISL_413591 |  | hCoV- |  | Europe / | 2020-03-02 | |  |
| --- | --- | --- | --- | --- | --- | --- | --- |
|  |  | 19/Netherlands/Zeewolde_136 |  | Netherlands / |  |  |  |
|  | 5080/2020 | |  | Zeewolde |  |  |  |
|  |  |  |  |  |  |  |  |

Foundation Elisabeth-Tweesteden Ziekenhuis


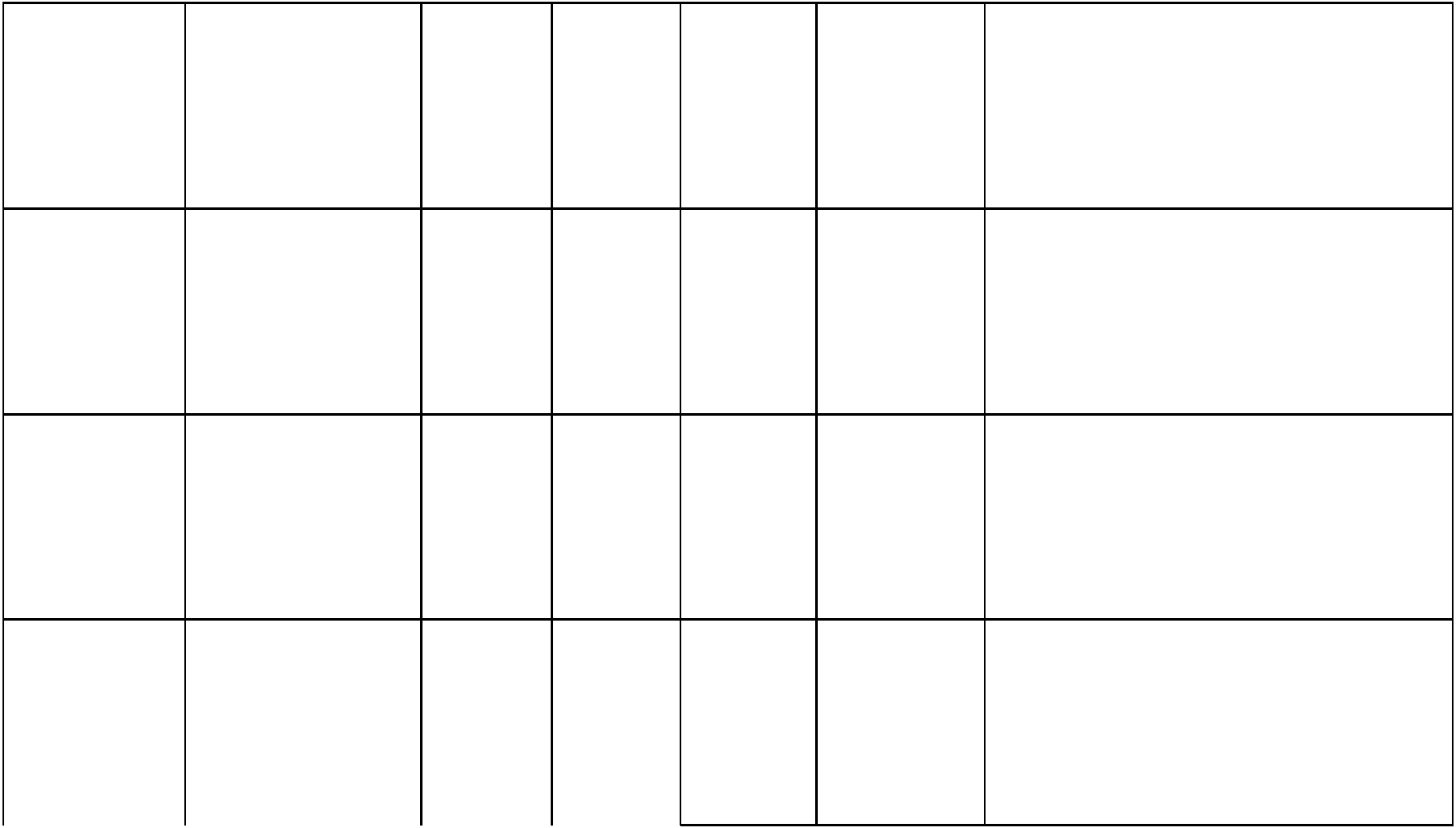


MHC Utrecht

MHC Utrecht

MHC Flevoland

Erasmus Medical

Center

Erasmus Medical

Center

Erasmus Medical

Center

Erasmus Medical

Center

David Nieuwenhuijse, Bas Oude Munnink, Reina Sikkema, Claudia Schapendonk, Irina Chestakova, Anne van der Linden, Mark Pronk, Pascal Lexmond, Corien Swaan, Manon Haverkate, Madelief Mollers, Mart Stein, Sandra Kengne Kamga Mobou, Jeroen van Kampen, Jolanda Voermans, Aura Timen, Corine GeurtsvanKessel, Annemiek van der Eijk, Richard Molenkamp, Marion Koopmans, on behalf of the Dutch national COVID-19 response team.

David Nieuwenhuijse, Bas Oude Munnink, Reina Sikkema, Claudia Schapendonk, Irina Chestakova, Anne van der Linden, Mark Pronk, Pascal Lexmond, Corien Swaan, Manon Haverkate, Madelief Mollers, Mart Stein, Sandra Kengne Kamga Mobou, Jeroen van Kampen, Jolanda Voermans, Aura Timen, Corine GeurtsvanKessel, Annemiek van der Eijk, Richard Molenkamp, Marion Koopmans, on behalf of the Dutch national COVID-19 response team.

David Nieuwenhuijse, Bas Oude Munnink, Reina Sikkema, Claudia Schapendonk, Irina Chestakova, Anne van der Linden, Mark Pronk, Pascal Lexmond, Corien Swaan, Manon Haverkate, Madelief Mollers, Mart Stein, Sandra Kengne Kamga Mobou, Jeroen van Kampen, Jolanda Voermans, Aura Timen, Corine GeurtsvanKessel, Annemiek van der Eijk, Richard Molenkamp, Marion Koopmans, on behalf of the Dutch national COVID-19 response team.

David Nieuwenhuijse, Bas Oude Munnink, Reina Sikkema, Claudia Schapendonk, Irina Chestakova, Anne van der Linden, Mark Pronk, Pascal Lexmond, Corien Swaan, Manon Haverkate, Madelief Mollers, Mart Stein, Sandra Kengne Kamga Mobou, Jeroen van Kampen, Jolanda Voermans, Aura Timen, Corine GeurtsvanKessel, Annemiek van der Eijk, Richard Molenkamp, Marion Koopmans, on behalf of the Dutch national COVID-19 response team.

EPI_ISL_413592

EPI_ISL_413593

EPI_ISL_413594

EPI_ISL_413595

EPI_ISL_413596

|  |  |  |  |  | Department of |  | Microbial Genomics |  |  |
| --- | --- | --- | --- | --- | --- | --- | --- | --- | --- |
|  |  | Asia / Taiwan / |  |  | Laboratory |  | Core Lab, National | Shiou-Hwei Yeh, You-Yu Lin, Ya-Yun Lai, Chiao-Ling Li, |  |
| hCoV-19/Taiwan/NTU03/2020 |  |  | 2020-03-02 | | Medicine, |  | Taiwan University |  |  |
|  |  | Taipei |  |  | National Taiwan |  | Centers of Genomic | Shan-Chwen Chang, Pei-Jer Chen, Sui-Yuan Chang |  |
|  |  |  |  |  |  |  |  |  |  |
|  |  |  |  |  | University |  | and Precision |  |  |
|  |  |  |  |  | Hospital |  | Medicine |  |  |
|  |  |  |  |  |  |  |  |  |  |
|  |  |  |  |  |  |  |  | David Nieuwenhuijse, Bas Oude Munnink, Reina Sikkema, |  |
|  |  |  |  |  |  |  |  | Claudia Schapendonk, Irina Chestakova, Anne van der |  |
| hCoV- |  | Europe / | 2020-02-29 | | Laboratoire |  | Erasmus Medical | Linden, Mark Pronk, Pascal Lexmond, T. Abdelrahman, G. |  |
|  |  |  |  |  | National de |  |  | Fournier, J. Mossong, T. Nguyen, Jeroen van Kampen, |  |
| 19/Luxembourg/Lux1/2020 |  | Luxembourg |  |  |  |  | Center |  |  |
|  |  |  |  |  | Santé |  |  | Jolanda Voermans, Corine GeurtsvanKessel, Annemiek van |  |
|  |  |  |  |  |  |  |  |  |  |
|  |  |  |  |  |  |  |  | der Eijk, Richard Molenkamp, Marion Koopmans, on behalf |  |
|  |  |  |  |  |  |  |  | of the Dutch national COVID-19 response team. |  |
|  |  |  |  |  |  |  |  |  |  |
|  |  |  |  |  | Centre for |  | NSW Health |  |  |
|  |  |  |  |  |  |  | Pathology - Institute |  |  |
|  |  |  |  |  | Infectious |  |  | Rockett R, Eden J-S, Lam C, Gray K, Timms, V, Gall, M, |  |
|  |  | Oceania / |  |  |  |  | of Clinical Pathology |  |  |
| hCoV- |  |  | 2020-02-28 | | Diseases and |  |  | Alicia, A, Carter I, Rahman H, Holmes EC, , O’Sullivan MV, |  |
|  |  | Australia / NSW |  |  |  |  | and Medical |  |  |
| 19/Australia/NSW08/2020 |  |  |  |  | Microbiology |  |  | Sintchenko V, Chen SC, Maddocks S, Kok J and Dwyer DE |  |
|  |  | / Sydney |  |  |  |  | Research; |  |  |
|  |  |  |  |  | Laboratory |  |  | for the 2019-nCoV Study Group* |  |
|  |  |  |  |  |  |  | Westmead Hospital; |  |  |
|  |  |  |  |  | Services |  |  |  |  |
|  |  |  |  |  |  |  | University of Sydney |  |  |
|  |  |  |  |  |  |  |  |  |  |
|  |  |  |  |  |  |  |  |  |  |
|  |  |  |  |  | Centre for |  | NSW Health |  |  |
|  |  |  |  |  |  |  | Pathology - Institute |  |  |
|  |  | Oceania / |  |  | Infectious |  |  | Rockett R, Eden J-S, Lam C, Gray K, Timms, V, Gall, M, |  |
|  |  |  |  |  |  |  | of Clinical Pathology |  |  |
| hCoV- |  |  |  |  | Diseases and |  |  | Carter I, Rahman H, Holmes EC, O’Sullivan MV, Sintchenko |  |
|  |  | Australia / NSW | 2020-02-28 | |  |  | and Medical |  |  |
| 19/Australia/NSW09/2020 |  |  |  |  | Microbiology |  |  | V, Chen SC, Maddocks S, Kok J and Dwyer DE for the 2019- |  |
|  |  | / Sydney |  |  |  |  | Research; |  |  |
|  |  |  |  |  | Laboratory |  |  | nCoV Study Group* |  |
|  |  |  |  |  |  |  | Westmead Hospital; |  |  |
|  |  |  |  |  | Services |  |  |  |  |
|  |  |  |  |  |  |  | University of Sydney |  |  |
|  |  |  |  |  |  |  |  |  |  |
|  |  |  |  |  |  |  |  |  |  |
|  |  |  |  |  |  |  | NSW Health |  |  |
|  |  |  |  |  | Centre for |  | Pathology - Institute | Rockett R, Eden J-S, Lam C, Gray K, Timms, V, Gall, M, |  |
|  |  | Oceania / |  |  | Infectious |  | of Clinical Pathology |  |  |
| hCoV- |  |  | 2020-02-28 | |  |  |  | Carter I, Rahman H, Holmes EC, O’Sullivan MV, Sintchenko |  |
|  |  | Australia / NSW |  |  | Diseases and |  | and Medical |  |  |
| 19/Australia/NSW10/2020 |  |  |  |  |  |  |  | V, Chen SC, Maddocks S, Kok J and Dwyer DE for the 2019- |  |
|  |  | / Sydney |  |  | Microbiology - |  | Research; |  |  |
|  |  |  |  |  |  |  |  | nCoV Study Group* |  |
|  |  |  |  |  | Public Health |  | Westmead Hospital; |  |  |
|  |  |  |  |  |  |  |  |  |  |


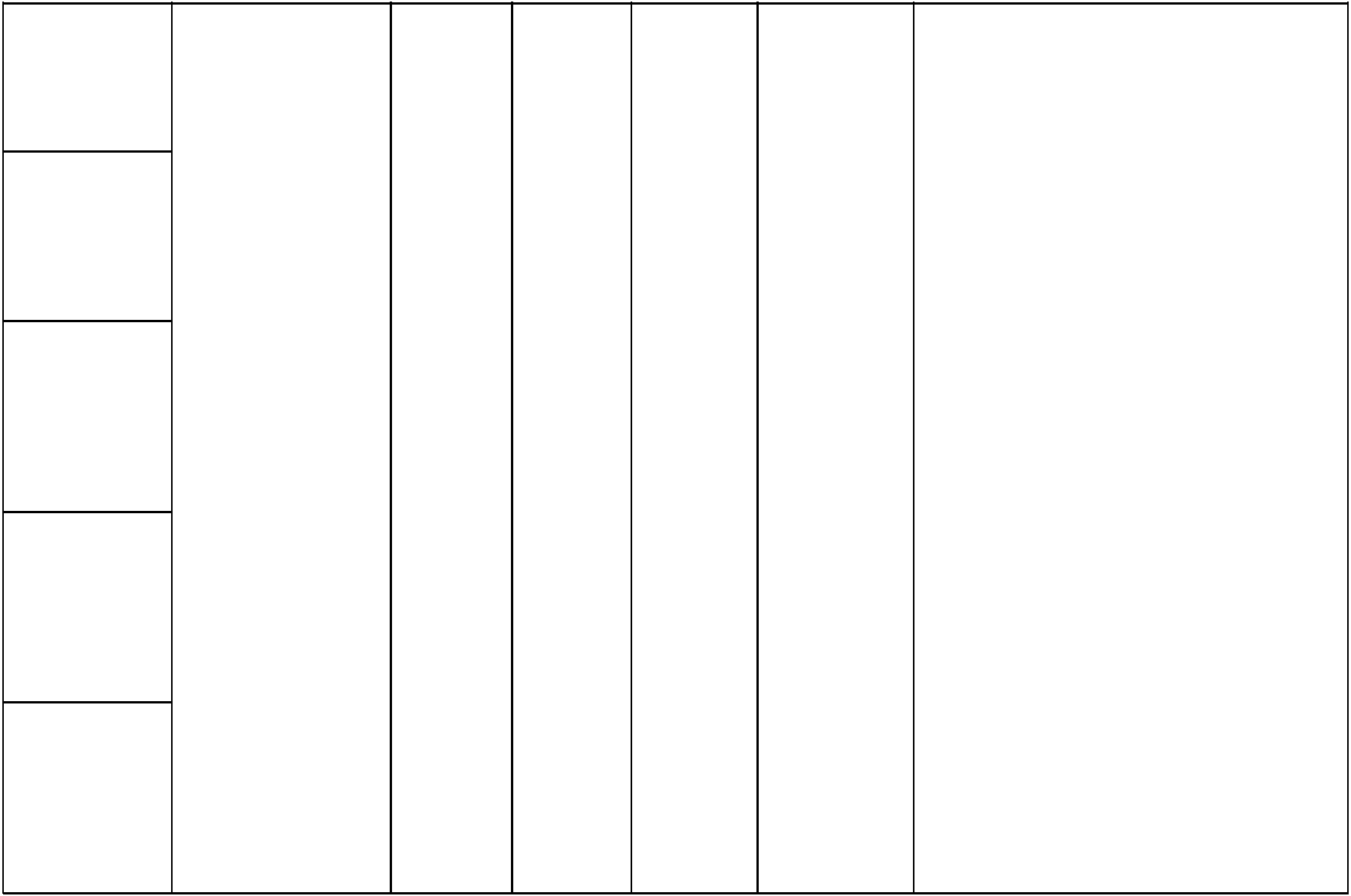


University of Sydney

EPI_ISL_413597

EPI_ISL_413598

EPI_ISL_413599

EPI_ISL_413600

hCoV-

19/Australia/NSW11/2020

hCoV-

19/Australia/NSW12/2020

hCoV-

19/Australia/NSW13/2020

hCoV-

19/Australia/NSW14/2020

|  |  |  |  |  | NSW Health |  |
| --- | --- | --- | --- | --- | --- | --- |
| Oceania / |  |  | Centre for |  | Pathology - Institute |  |
|  |  |  | Infectious |  | of Clinical Pathology |  |
| Australia / NSW | 2020-03-02 | | Diseases and |  | and Medical |  |
| / Sydney |  |  | Microbiology- |  | Research; |  |
|  |  |  | Public Health |  | Westmead Hospital; |  |
|  |  |  |  |  | University of Sydney |  |
|  |  |  |  |  |  |  |
|  |  |  | Centre for |  | NSW Health |  |
|  |  |  |  |  | Pathology - Institute |  |
| Oceania / | 2020-03-04 | | Infectious |  | of Clinical Pathology |  |
| Australia / NSW |  |  | Diseases and |  | and Medical |  |
| / Sydney |  |  | Microbiology - |  | Research; |  |
|  |  |  | Public Health |  | Westmead Hospital; |  |
|  |  |  |  |  | University of Sydney |  |
|  |  |  |  |  |  |  |
|  |  |  |  |  | NSW Health |  |
| Oceania / |  |  | Centre for |  | Pathology - Institute |  |
|  |  |  | Infectious |  | of Clinical Pathology |  |
| Australia / NSW | 2020-03-04 | | Diseases and |  | and Medical |  |
| / Sydney |  |  | Microbiology - |  | Research; |  |
|  |  |  | Public Health |  | Westmead Hospital; |  |
|  |  |  |  |  | University of Sydney |  |
|  |  |  |  |  |  |  |
|  |  |  | Centre for |  | NSW Health |  |
|  |  |  |  |  | Pathology - Institute |  |
| Oceania / | 2020-03-03 | | Infectious |  | of Clinical Pathology |  |
| Australia / NSW |  |  | Diseases and |  | and Medical |  |
| / Sydney |  |  | Microbiology - |  | Research; |  |
|  |  |  | Public Health |  | Westmead Hospital; |  |
|  |  |  |  |  | University of Sydney |  |
|  |  |  |  |  |  |  |


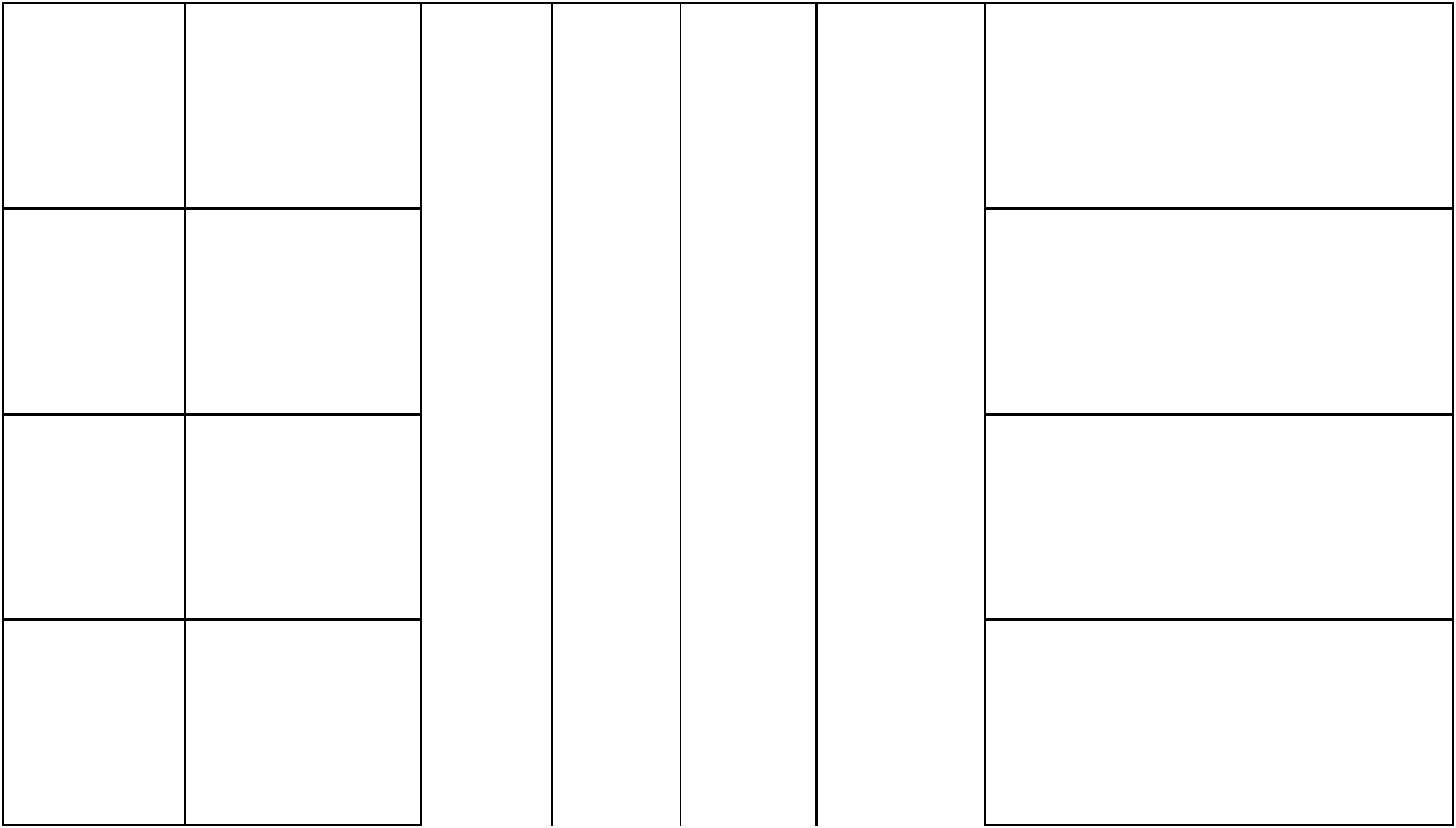


Lam C, Eden J-S, Rockett R, Gray K, Timms, V, Gall, M, Carter I, Rahman H, Holmes EC, O’Sullivan MV, Sintchenko V, Chen SC, Maddocks S, Kok J and Dwyer DE for the 2019-nCoV Study Group*

Gray K, Eden J-S, Lam C, Rockett R, Timms, V, Gall, M, Carter I, Rahman H, Holmes EC, O’Sullivan MV, Sintchenko V, Chen SC, Maddocks S, Kok J and Dwyer DE for the 2019-nCoV Study Group*

Timms, V, Eden J-S, Lam C, Gray K, Rockett R, Gall, M, Carter I, Rahman H, Holmes EC, O’Sullivan MV, Sintchenko V, Chen SC, Maddocks S, Kok J and Dwyer DE for the 2019-nCoV Study Group*

Gall, M, Eden J-S, Lam C, Gray K, Timms, V, Rockett R, Carter I, Rahman H, Holmes EC, O’Sullivan MV, Sintchenko V, Chen SC, Maddocks S, Kok J and Dwyer DE for the 2019-nCoV Study Group*

EPI_ISL_414005

EPI_ISL_414006

EPI_ISL_414007

EPI_ISL_414008

EPI_ISL_414009

EPI_ISL_414010

hCoV-

19/England/200940527/2020

hCoV-

19/England/200990724/2020

hCoV-

19/England/200990725/2020

hCoV-

19/England/200960041/2020

hCoV-

19/England/200960515/2020

hCoV-

19/England/200981386/2020

Europe / United Kingdom / England

Europe / United Kingdom / England

Europe / United Kingdom / England

Europe / United Kingdom / England

Europe / United Kingdom / England

Europe / United Kingdom / England

2020-02-25

2020-02-28

2020-02-28

2020-02-27

2020-02-25

2020-02-26

Respiratory Virus Unit, Microbiology Services Colindale, Public Health England


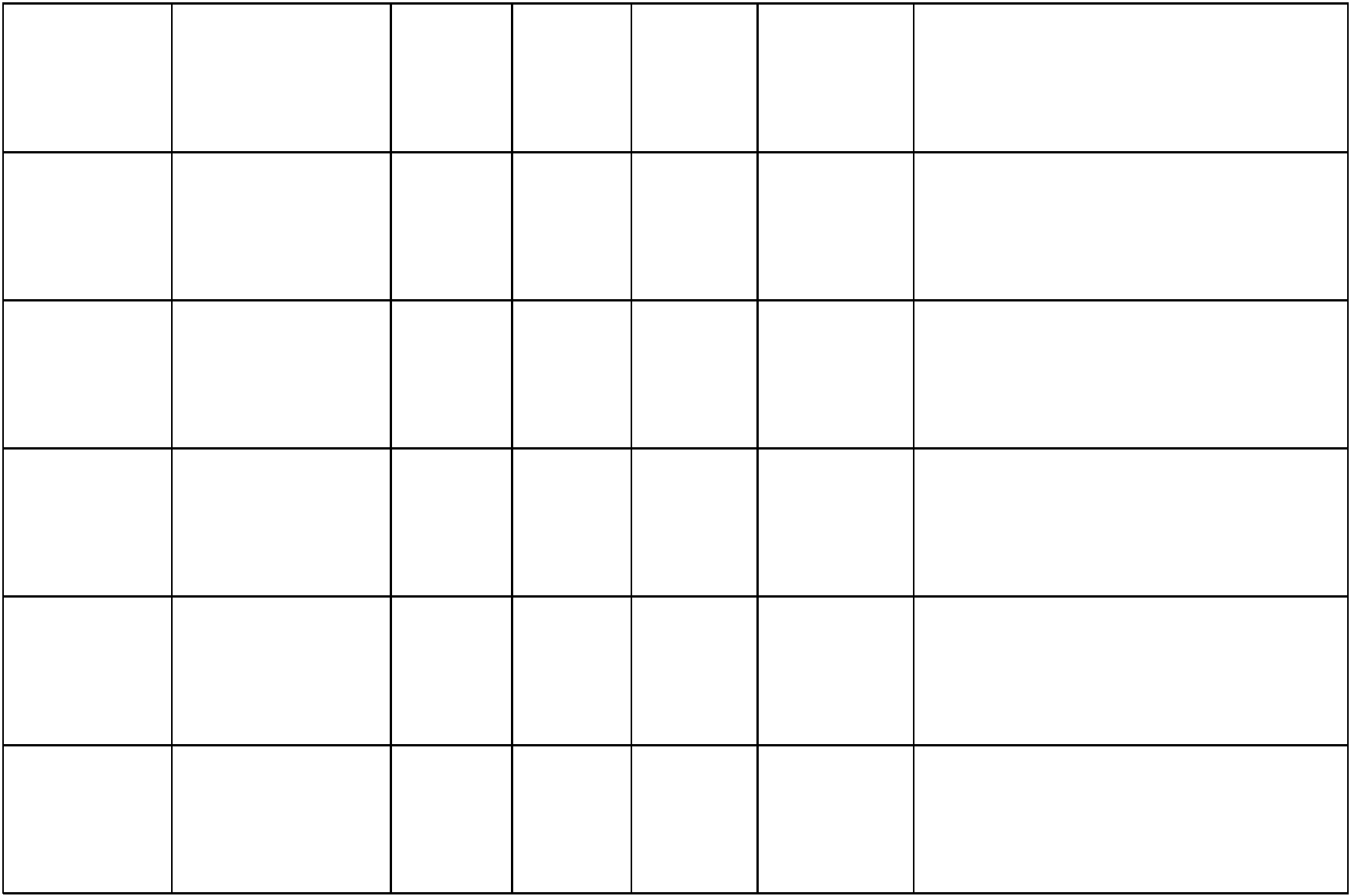


Respiratory Virus Unit, Microbiology Services Colindale, Public Health England

Respiratory Virus Unit, Microbiology Services Colindale, Public Health England

Respiratory Virus Unit, Microbiology Services Colindale, Public Health England

Respiratory Virus Unit, Microbiology Services Colindale, Public Health England

Respiratory Virus Unit, Microbiology Services Colindale, Public Health England

Respiratory Virus Unit, Microbiology Services Colindale, Public Health England

Respiratory Virus Unit, Microbiology Services Colindale, Public Health England

Respiratory Virus Unit, Microbiology Services Colindale, Public Health England

Respiratory Virus Unit, Microbiology Services Colindale, Public Health England

Respiratory Virus Unit, Microbiology Services Colindale, Public Health England

Respiratory Virus Unit, Microbiology Services Colindale, Public Health England

Monica Galiano, Shahjahan Miah, Angie Lackenby, Omolola Akinbami, Tiina Talts, Leena Bhaw, Richard Myers, Steven Platt, Kirstin Edwards, Jonathan Hubb, Joanna Ellis, Maria Zambon

Monica Galiano, Shahjahan Miah, Angie Lackenby, Omolola Akinbami, Tiina Talts, Leena Bhaw, Richard Myers, Steven Platt, Kirstin Edwards, Jonathan Hubb, Joanna Ellis, Maria Zambon

Monica Galiano, Shahjahan Miah, Angie Lackenby, Omolola Akinbami, Tiina Talts, Leena Bhaw, Richard Myers, Steven Platt, Kirstin Edwards, Jonathan Hubb, Joanna Ellis, Maria Zambon

Monica Galiano, Shahjahan Miah, Angie Lackenby, Omolola Akinbami, Tiina Talts, Leena Bhaw, Richard Myers, Steven Platt, Kirstin Edwards, Jonathan Hubb, Joanna Ellis, Maria Zambon

Monica Galiano, Shahjahan Miah, Angie Lackenby, Omolola Akinbami, Tiina Talts, Leena Bhaw, Richard Myers, Steven Platt, Kirstin Edwards, Jonathan Hubb, Joanna Ellis, Maria Zambon

Monica Galiano, Shahjahan Miah, Angie Lackenby, Omolola Akinbami, Tiina Talts, Leena Bhaw, Richard Myers, Steven Platt, Kirstin Edwards, Jonathan Hubb, Joanna Ellis, Maria Zambon

EPI_ISL_414011

EPI_ISL_414012

EPI_ISL_414019

EPI_ISL_414020

EPI_ISL_414021

EPI_ISL_414022

EPI_ISL_414023

EPI_ISL_415470

hCoV-

19/England/200990006/2020

hCoV-

19/England/200990723/2020

hCoV-

19/Switzerland/GE3121/2020

hCoV-

19/Switzerland/GE5373/2020

hCoV-

19/Switzerland/BL0902/2020

hCoV-

19/Switzerland/GE9586/2020

hCoV-

19/Switzerland/VD5615/2020

hCoV-

19/Netherlands/NA_14/2020

Europe / United Kingdom / England

Europe / United Kingdom / England

Europe / Switzerland / Geneva

Europe / Switzerland / Geneva

Europe / Switzerland / Basel

Europe / Switzerland / Geneva

Europe / Switzerland / Vaud

Europe /

Netherlands

2020-02-26

2020-02-27

2020-02-27

2020-02-27

2020-02-27

2020-02-27

2020-03-01

2020-03-10

Respiratory Virus Unit, Microbiology Services Colindale, Public Health England


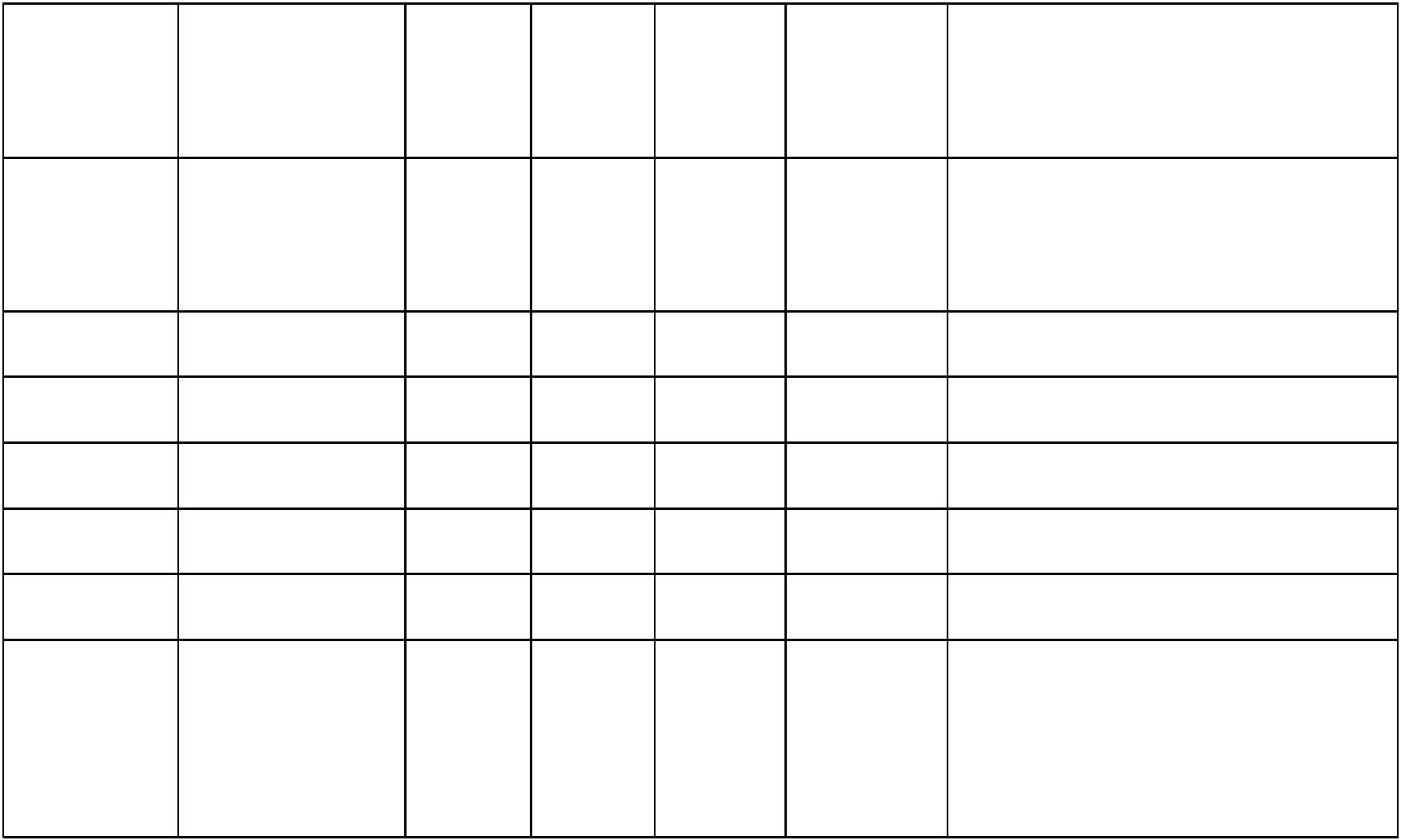


Respiratory Virus Unit, Microbiology Services Colindale, Public Health England

Laboratoire de Virologie, HUG

Laboratoire de Virologie, HUG

Laboratoire de Virologie, HUG

Laboratoire de Virologie, HUG

Laboratoire de Virologie, HUG

Dutch COVID-

1. response team

Respiratory Virus Unit, Microbiology Services Colindale, Public Health England

Respiratory Virus Unit, Microbiology Services Colindale, Public Health England

Swiss National Reference Centre for Influenza

Swiss National Reference Centre for Influenza

Swiss National Reference Centre for Influenza

Swiss National Reference Centre for Influenza

Swiss National Reference Centre for Influenza

Erasmus Medical

Center

Monica Galiano, Shahjahan Miah, Angie Lackenby, Omolola Akinbami, Tiina Talts, Leena Bhaw, Richard Myers, Steven Platt, Kirstin Edwards, Jonathan Hubb, Joanna Ellis, Maria Zambon

Monica Galiano, Shahjahan Miah, Angie Lackenby, Omolola Akinbami, Tiina Talts, Leena Bhaw, Richard Myers, Steven Platt, Kirstin Edwards, Jonathan Hubb, Joanna Ellis, Maria Zambon

LAUBSCHER Florian et al.

LAUBSCHER Florian et al.

LAUBSCHER Florian et al.

LAUBSCHER Florian et al.

LAUBSCHER Florian et al.

David Nieuwenhuijse, Bas Oude Munnink, Reina Sikkema, Claudia Schapendonk, Irina Chestakova, Anne van der Linden, Mark Pronk, Pascal Lexmond, Corien Swaan, Manon Haverkate, Madelief Mollers, Mart Stein, Sandra Kengne Kamga Mobou, Jeroen van Kampen, Jolanda Voermans, Aura Timen, Corine GeurtsvanKessel, Annemiek van der Eijk, Richard Molenkamp, Marion Koopmans, on behalf of the Dutch national COVID-19 response team.

EPI_ISL_415471

EPI_ISL_415472

EPI_ISL_415473

EPI_ISL_415474

hCoV-

19/Netherlands/NA_15/2020

hCoV-

19/Netherlands/NA_16/2020

hCoV-

19/Netherlands/NA_17/2020

hCoV-

19/Netherlands/NA_18/2020

Europe /

Netherlands

Europe /

Netherlands

Europe /

Netherlands

Europe /

Netherlands

2020-03-11

2020-03-11

2020-03-09

2020-03-09

Dutch COVID-


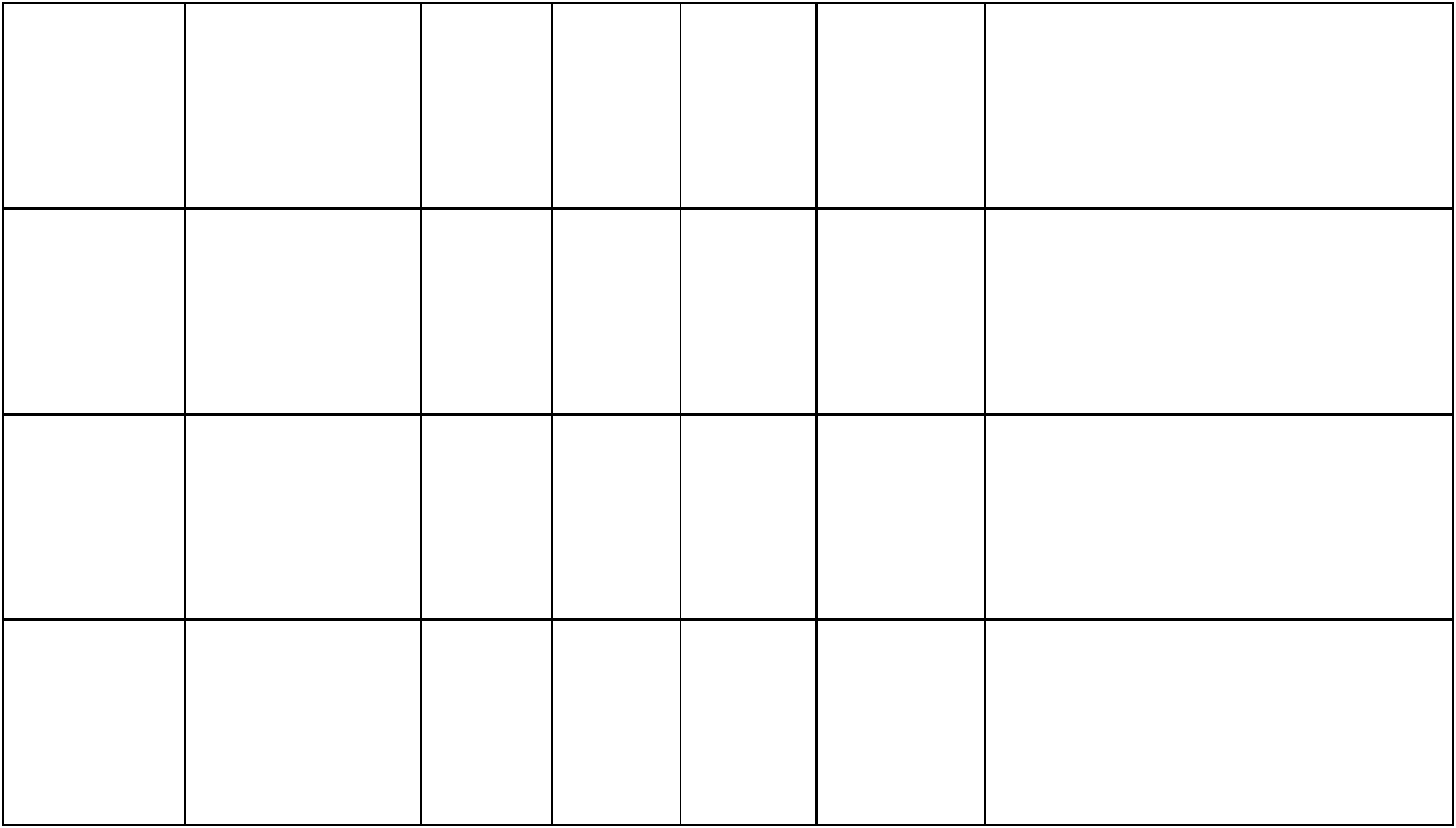


1. response team

Dutch COVID-

1. response team

Dutch COVID-

1. response team

Dutch COVID-

1. response team

Erasmus Medical

Center

Erasmus Medical

Center

Erasmus Medical

Center

Erasmus Medical

Center

David Nieuwenhuijse, Bas Oude Munnink, Reina Sikkema, Claudia Schapendonk, Irina Chestakova, Anne van der Linden, Mark Pronk, Pascal Lexmond, Corien Swaan, Manon Haverkate, Madelief Mollers, Mart Stein, Sandra Kengne Kamga Mobou, Jeroen van Kampen, Jolanda Voermans, Aura Timen, Corine GeurtsvanKessel, Annemiek van der Eijk, Richard Molenkamp, Marion Koopmans, on behalf of the Dutch national COVID-19 response team.

David Nieuwenhuijse, Bas Oude Munnink, Reina Sikkema, Claudia Schapendonk, Irina Chestakova, Anne van der Linden, Mark Pronk, Pascal Lexmond, Corien Swaan, Manon Haverkate, Madelief Mollers, Mart Stein, Sandra Kengne Kamga Mobou, Jeroen van Kampen, Jolanda Voermans, Aura Timen, Corine GeurtsvanKessel, Annemiek van der Eijk, Richard Molenkamp, Marion Koopmans, on behalf of the Dutch national COVID-19 response team.

David Nieuwenhuijse, Bas Oude Munnink, Reina Sikkema, Claudia Schapendonk, Irina Chestakova, Anne van der Linden, Mark Pronk, Pascal Lexmond, Corien Swaan, Manon Haverkate, Madelief Mollers, Mart Stein, Sandra Kengne Kamga Mobou, Jeroen van Kampen, Jolanda Voermans, Aura Timen, Corine GeurtsvanKessel, Annemiek van der Eijk, Richard Molenkamp, Marion Koopmans, on behalf of the Dutch national COVID-19 response team.

David Nieuwenhuijse, Bas Oude Munnink, Reina Sikkema, Claudia Schapendonk, Irina Chestakova, Anne van der Linden, Mark Pronk, Pascal Lexmond, Corien Swaan, Manon Haverkate, Madelief Mollers, Mart Stein, Sandra Kengne Kamga Mobou, Jeroen van Kampen, Jolanda Voermans, Aura Timen, Corine GeurtsvanKessel, Annemiek van der Eijk, Richard Molenkamp, Marion Koopmans, on behalf of the Dutch national COVID-19 response team.

EPI_ISL_415475

EPI_ISL_415476

EPI_ISL_415478

EPI_ISL_415480

hCoV-

19/Netherlands/NA_19/2020

hCoV-

19/Netherlands/NA_2/2020

hCoV-

19/Netherlands/NA_21/2020

hCoV-

19/Netherlands/NA_23/2020

Europe /

Netherlands

Europe /

Netherlands

Europe /

Netherlands

Europe /

Netherlands

2020-03-12

2020-03-10

2020-03-08

2020-03-09

Dutch COVID-


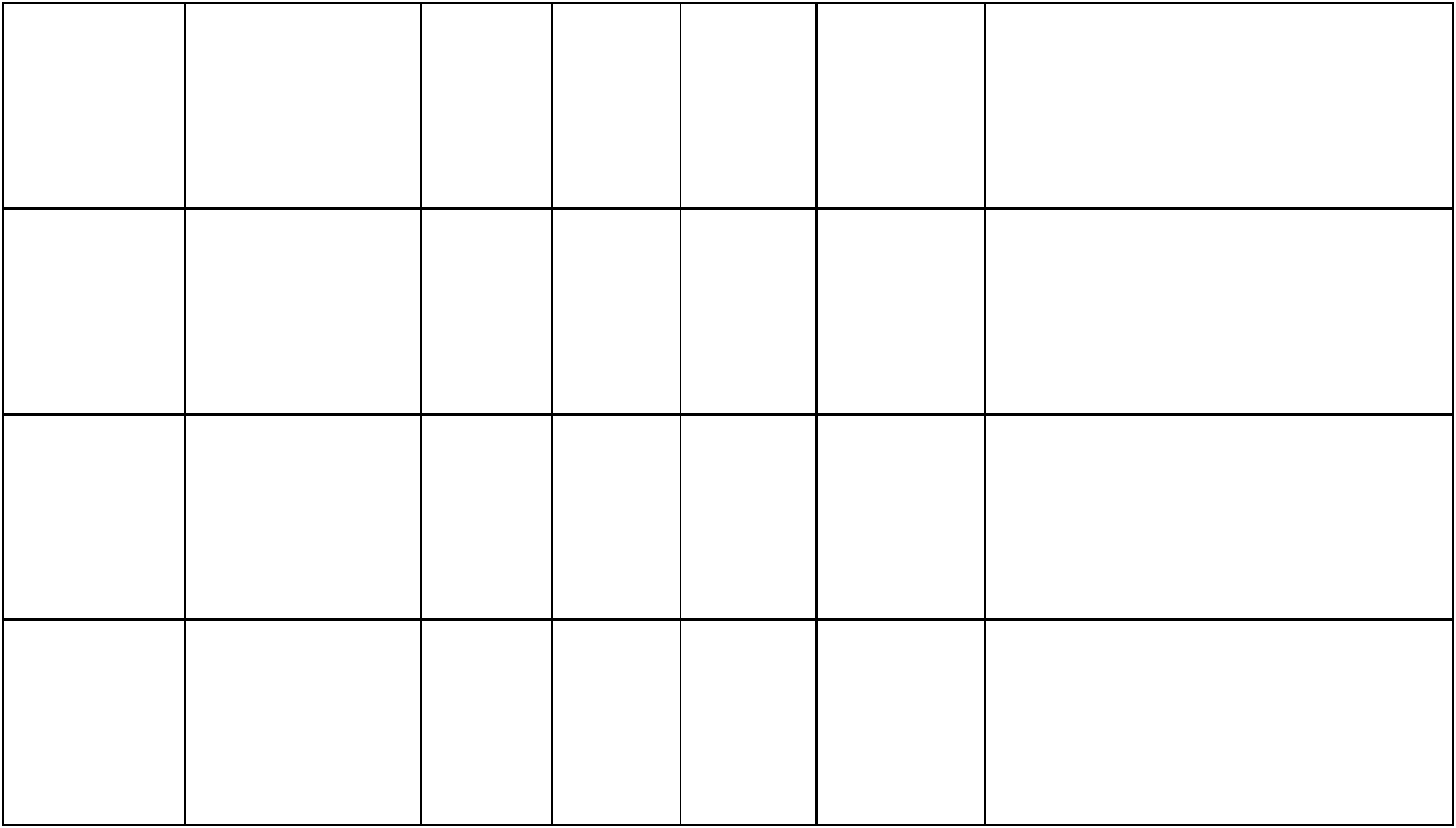


1. response team

Dutch COVID-

1. response team

Dutch COVID-

1. response team

Dutch COVID-

1. response team

Erasmus Medical

Center

Erasmus Medical

Center

Erasmus Medical

Center

Erasmus Medical

Center

David Nieuwenhuijse, Bas Oude Munnink, Reina Sikkema, Claudia Schapendonk, Irina Chestakova, Anne van der Linden, Mark Pronk, Pascal Lexmond, Corien Swaan, Manon Haverkate, Madelief Mollers, Mart Stein, Sandra Kengne Kamga Mobou, Jeroen van Kampen, Jolanda Voermans, Aura Timen, Corine GeurtsvanKessel, Annemiek van der Eijk, Richard Molenkamp, Marion Koopmans, on behalf of the Dutch national COVID-19 response team.

David Nieuwenhuijse, Bas Oude Munnink, Reina Sikkema, Claudia Schapendonk, Irina Chestakova, Anne van der Linden, Mark Pronk, Pascal Lexmond, Corien Swaan, Manon Haverkate, Madelief Mollers, Mart Stein, Sandra Kengne Kamga Mobou, Jeroen van Kampen, Jolanda Voermans, Aura Timen, Corine GeurtsvanKessel, Annemiek van der Eijk, Richard Molenkamp, Marion Koopmans, on behalf of the Dutch national COVID-19 response team.

David Nieuwenhuijse, Bas Oude Munnink, Reina Sikkema, Claudia Schapendonk, Irina Chestakova, Anne van der Linden, Mark Pronk, Pascal Lexmond, Corien Swaan, Manon Haverkate, Madelief Mollers, Mart Stein, Sandra Kengne Kamga Mobou, Jeroen van Kampen, Jolanda Voermans, Aura Timen, Corine GeurtsvanKessel, Annemiek van der Eijk, Richard Molenkamp, Marion Koopmans, on behalf of the Dutch national COVID-19 response team.

David Nieuwenhuijse, Bas Oude Munnink, Reina Sikkema, Claudia Schapendonk, Irina Chestakova, Anne van der Linden, Mark Pronk, Pascal Lexmond, Corien Swaan, Manon Haverkate, Madelief Mollers, Mart Stein, Sandra Kengne Kamga Mobou, Jeroen van Kampen, Jolanda Voermans, Aura Timen, Corine GeurtsvanKessel, Annemiek van der Eijk, Richard Molenkamp, Marion Koopmans, on behalf of the Dutch national COVID-19 response team.

EPI_ISL_415481

EPI_ISL_415482

EPI_ISL_415483

EPI_ISL_415484

hCoV-

19/Netherlands/NA_24/2020

hCoV-

19/Netherlands/NA_25/2020

hCoV-

19/Netherlands/NA_26/2020

hCoV-

19/Netherlands/NA_27/2020

Europe /

Netherlands

Europe /

Netherlands

Europe /

Netherlands

Europe /

Netherlands

2020-03-08

2020-03-09

2020-03-09

2020-03-13

Dutch COVID-


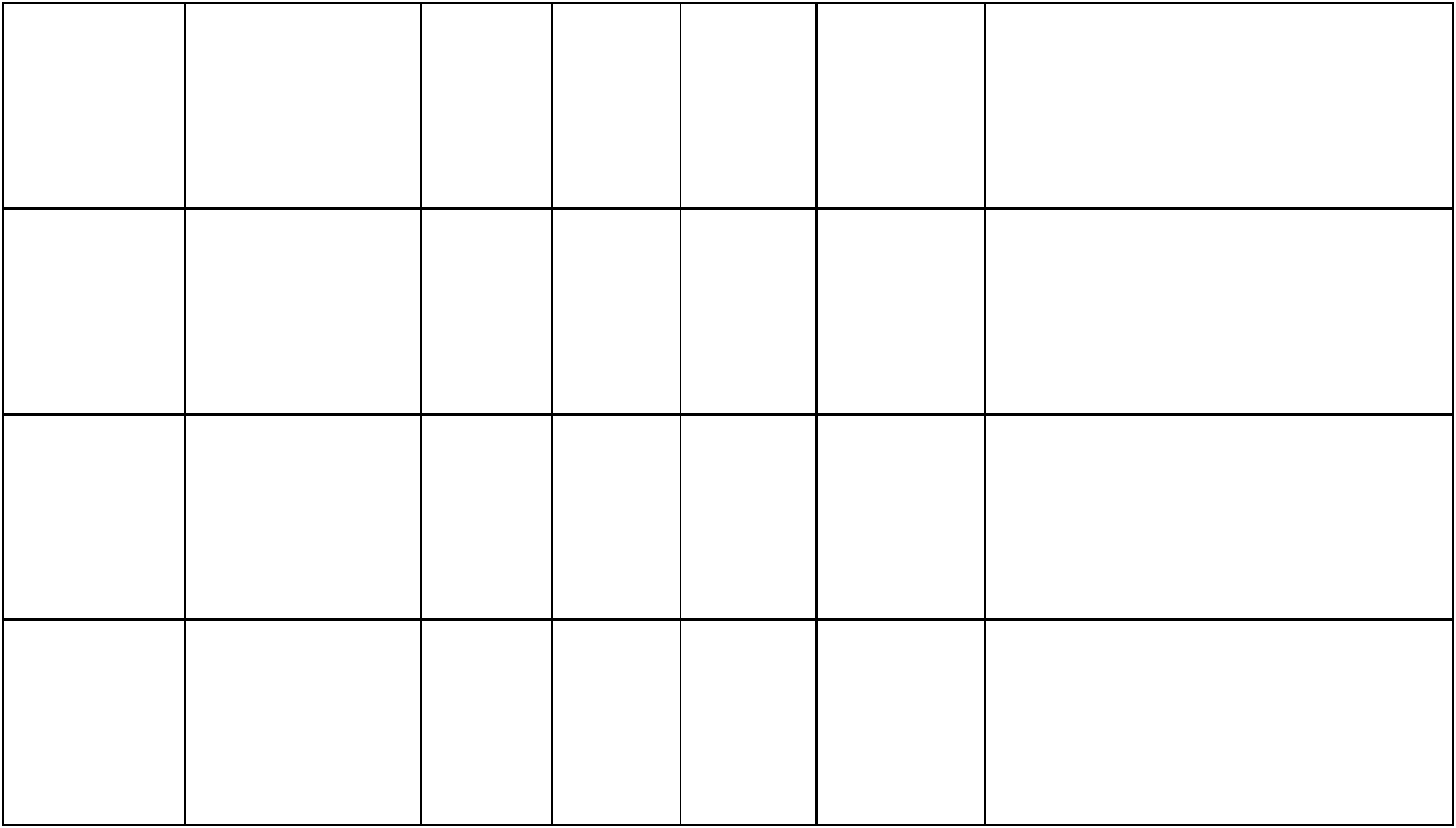


1. response team

Dutch COVID-

1. response team

Dutch COVID-

1. response team

Dutch COVID-

1. response team

Erasmus Medical

Center

Erasmus Medical

Center

Erasmus Medical

Center

Erasmus Medical

Center

David Nieuwenhuijse, Bas Oude Munnink, Reina Sikkema, Claudia Schapendonk, Irina Chestakova, Anne van der Linden, Mark Pronk, Pascal Lexmond, Corien Swaan, Manon Haverkate, Madelief Mollers, Mart Stein, Sandra Kengne Kamga Mobou, Jeroen van Kampen, Jolanda Voermans, Aura Timen, Corine GeurtsvanKessel, Annemiek van der Eijk, Richard Molenkamp, Marion Koopmans, on behalf of the Dutch national COVID-19 response team.

David Nieuwenhuijse, Bas Oude Munnink, Reina Sikkema, Claudia Schapendonk, Irina Chestakova, Anne van der Linden, Mark Pronk, Pascal Lexmond, Corien Swaan, Manon Haverkate, Madelief Mollers, Mart Stein, Sandra Kengne Kamga Mobou, Jeroen van Kampen, Jolanda Voermans, Aura Timen, Corine GeurtsvanKessel, Annemiek van der Eijk, Richard Molenkamp, Marion Koopmans, on behalf of the Dutch national COVID-19 response team.

David Nieuwenhuijse, Bas Oude Munnink, Reina Sikkema, Claudia Schapendonk, Irina Chestakova, Anne van der Linden, Mark Pronk, Pascal Lexmond, Corien Swaan, Manon Haverkate, Madelief Mollers, Mart Stein, Sandra Kengne Kamga Mobou, Jeroen van Kampen, Jolanda Voermans, Aura Timen, Corine GeurtsvanKessel, Annemiek van der Eijk, Richard Molenkamp, Marion Koopmans, on behalf of the Dutch national COVID-19 response team.

David Nieuwenhuijse, Bas Oude Munnink, Reina Sikkema, Claudia Schapendonk, Irina Chestakova, Anne van der Linden, Mark Pronk, Pascal Lexmond, Corien Swaan, Manon Haverkate, Madelief Mollers, Mart Stein, Sandra Kengne Kamga Mobou, Jeroen van Kampen, Jolanda Voermans, Aura Timen, Corine GeurtsvanKessel, Annemiek van der Eijk, Richard Molenkamp, Marion Koopmans, on behalf of the Dutch national COVID-19 response team.

EPI_ISL_415485

EPI_ISL_415486

EPI_ISL_415487

EPI_ISL_415488

hCoV-

19/Netherlands/NA_28/2020

hCoV-

19/Netherlands/NA_29/2020

hCoV-

19/Netherlands/NA_30/2020

hCoV-

19/Netherlands/NA_31/2020

Europe /

Netherlands

Europe /

Netherlands

Europe /

Netherlands

Europe /

Netherlands

2020-03-12

2020-03-13

2020-03-13

2020-03-13

Dutch COVID-


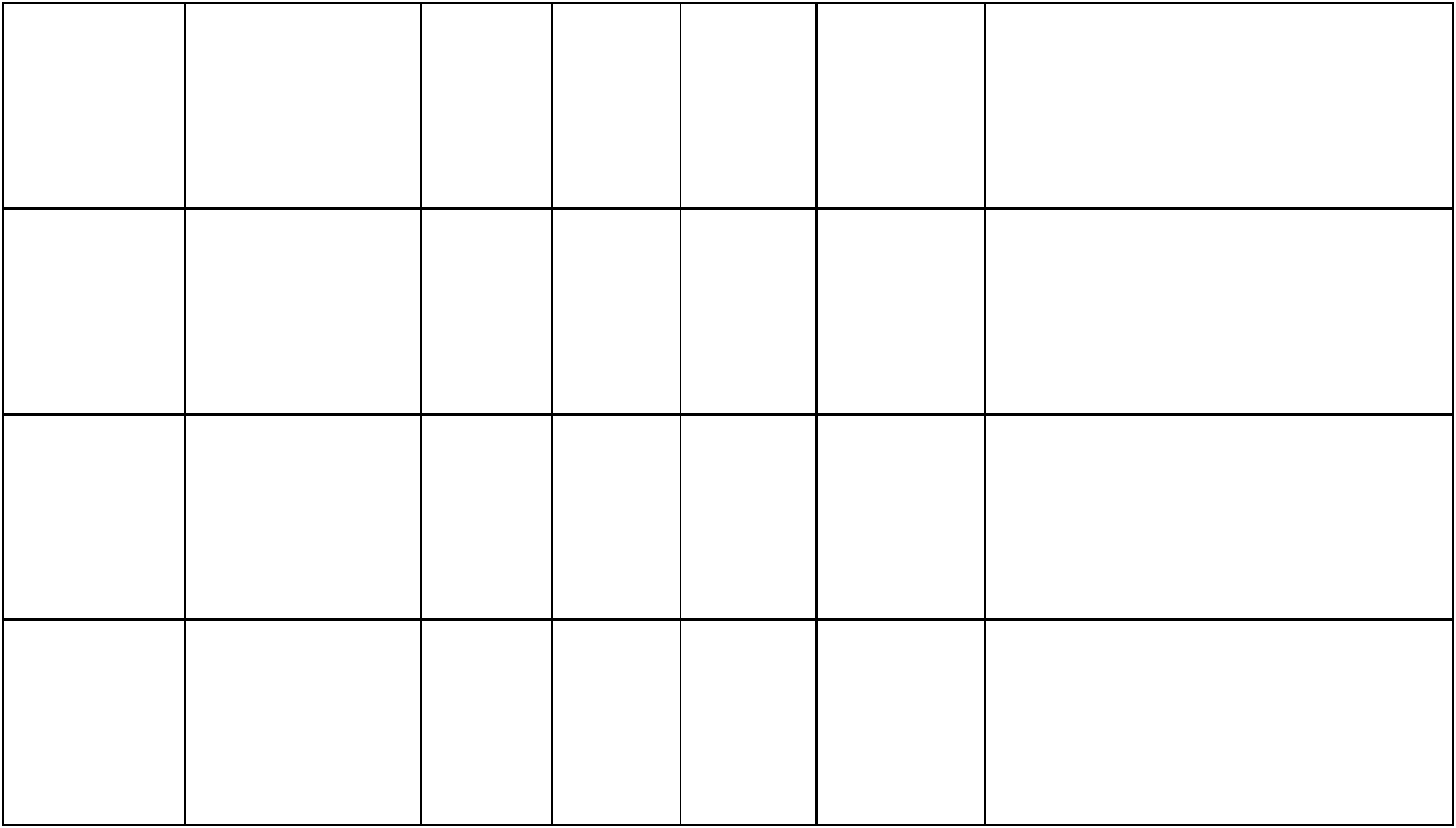


1. response team

Dutch COVID-

1. response team

Dutch COVID-

1. response team

Dutch COVID-

1. response team

Erasmus Medical

Center

Erasmus Medical

Center

Erasmus Medical

Center

Erasmus Medical

Center

David Nieuwenhuijse, Bas Oude Munnink, Reina Sikkema, Claudia Schapendonk, Irina Chestakova, Anne van der Linden, Mark Pronk, Pascal Lexmond, Corien Swaan, Manon Haverkate, Madelief Mollers, Mart Stein, Sandra Kengne Kamga Mobou, Jeroen van Kampen, Jolanda Voermans, Aura Timen, Corine GeurtsvanKessel, Annemiek van der Eijk, Richard Molenkamp, Marion Koopmans, on behalf of the Dutch national COVID-19 response team.

David Nieuwenhuijse, Bas Oude Munnink, Reina Sikkema, Claudia Schapendonk, Irina Chestakova, Anne van der Linden, Mark Pronk, Pascal Lexmond, Corien Swaan, Manon Haverkate, Madelief Mollers, Mart Stein, Sandra Kengne Kamga Mobou, Jeroen van Kampen, Jolanda Voermans, Aura Timen, Corine GeurtsvanKessel, Annemiek van der Eijk, Richard Molenkamp, Marion Koopmans, on behalf of the Dutch national COVID-19 response team.

David Nieuwenhuijse, Bas Oude Munnink, Reina Sikkema, Claudia Schapendonk, Irina Chestakova, Anne van der Linden, Mark Pronk, Pascal Lexmond, Corien Swaan, Manon Haverkate, Madelief Mollers, Mart Stein, Sandra Kengne Kamga Mobou, Jeroen van Kampen, Jolanda Voermans, Aura Timen, Corine GeurtsvanKessel, Annemiek van der Eijk, Richard Molenkamp, Marion Koopmans, on behalf of the Dutch national COVID-19 response team.

David Nieuwenhuijse, Bas Oude Munnink, Reina Sikkema, Claudia Schapendonk, Irina Chestakova, Anne van der Linden, Mark Pronk, Pascal Lexmond, Corien Swaan, Manon Haverkate, Madelief Mollers, Mart Stein, Sandra Kengne Kamga Mobou, Jeroen van Kampen, Jolanda Voermans, Aura Timen, Corine GeurtsvanKessel, Annemiek van der Eijk, Richard Molenkamp, Marion Koopmans, on behalf of the Dutch national COVID-19 response team.

EPI_ISL_415491

EPI_ISL_415492

EPI_ISL_415495

EPI_ISL_415496

hCoV-

19/Netherlands/NA_34/2020

hCoV-

19/Netherlands/NA_35/2020

hCoV-

19/Netherlands/NA_6/2020

hCoV-

19/Netherlands/NA_7/2020

Europe /

Netherlands

Europe /

Netherlands

Europe /

Netherlands

Europe /

Netherlands

2020-03-07

2020-03-10

2020-03-10

2020-03-09

Dutch COVID-


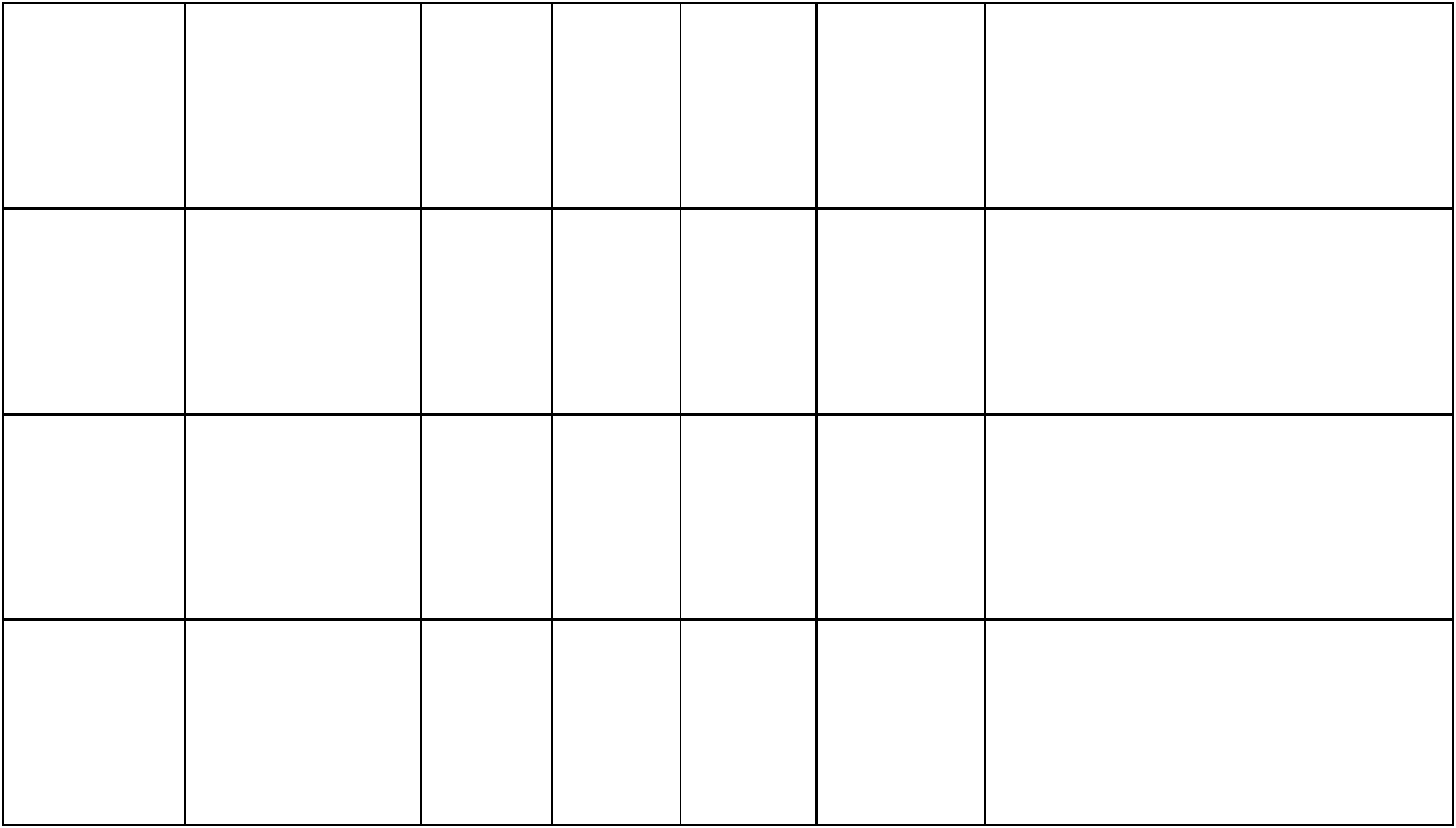


1. response team

Dutch COVID-

1. response team

Dutch COVID-

1. response team

Dutch COVID-

1. response team

Erasmus Medical

Center

Erasmus Medical

Center

Erasmus Medical

Center

Erasmus Medical

Center

David Nieuwenhuijse, Bas Oude Munnink, Reina Sikkema, Claudia Schapendonk, Irina Chestakova, Anne van der Linden, Mark Pronk, Pascal Lexmond, Corien Swaan, Manon Haverkate, Madelief Mollers, Mart Stein, Sandra Kengne Kamga Mobou, Jeroen van Kampen, Jolanda Voermans, Aura Timen, Corine GeurtsvanKessel, Annemiek van der Eijk, Richard Molenkamp, Marion Koopmans, on behalf of the Dutch national COVID-19 response team.

David Nieuwenhuijse, Bas Oude Munnink, Reina Sikkema, Claudia Schapendonk, Irina Chestakova, Anne van der Linden, Mark Pronk, Pascal Lexmond, Corien Swaan, Manon Haverkate, Madelief Mollers, Mart Stein, Sandra Kengne Kamga Mobou, Jeroen van Kampen, Jolanda Voermans, Aura Timen, Corine GeurtsvanKessel, Annemiek van der Eijk, Richard Molenkamp, Marion Koopmans, on behalf of the Dutch national COVID-19 response team.

David Nieuwenhuijse, Bas Oude Munnink, Reina Sikkema, Claudia Schapendonk, Irina Chestakova, Anne van der Linden, Mark Pronk, Pascal Lexmond, Corien Swaan, Manon Haverkate, Madelief Mollers, Mart Stein, Sandra Kengne Kamga Mobou, Jeroen van Kampen, Jolanda Voermans, Aura Timen, Corine GeurtsvanKessel, Annemiek van der Eijk, Richard Molenkamp, Marion Koopmans, on behalf of the Dutch national COVID-19 response team.

David Nieuwenhuijse, Bas Oude Munnink, Reina Sikkema, Claudia Schapendonk, Irina Chestakova, Anne van der Linden, Mark Pronk, Pascal Lexmond, Corien Swaan, Manon Haverkate, Madelief Mollers, Mart Stein, Sandra Kengne Kamga Mobou, Jeroen van Kampen, Jolanda Voermans, Aura Timen, Corine GeurtsvanKessel, Annemiek van der Eijk, Richard Molenkamp, Marion Koopmans, on behalf of the Dutch national COVID-19 response team.

EPI_ISL_415497

EPI_ISL_415498

EPI_ISL_416636

EPI_ISL_416641

EPI_ISL_416642

EPI_ISL_416658

EPI_ISL_416660

EPI_ISL_416661

EPI_ISL_416662

EPI_ISL_416663

hCoV-

19/Netherlands/NA_8/2020

hCoV-

19/Netherlands/NA_9/2020

hCoV-19/USA/WA-

UW98/2020

hCoV-19/USA/WA-

UW103/2020

hCoV-19/USA/WA-

UW104/2020

hCoV-19/USA/WA-

UW120/2020

hCoV-19/USA/WA-

UW122/2020

hCoV-19/USA/WA-

UW123/2020

hCoV-19/USA/WA-

UW124/2020

hCoV-19/USA/WA-

UW125/2020

Europe /

Netherlands

Europe /

Netherlands

North America /

USA /

Washington

North America /

USA /

Washington

North America /

USA /

Washington

North America /

USA /

Washington

North America /

USA

North America /

USA /

Washington

North America /

USA /

Washington

North America /

USA /

Washington

|  |  |  |  | David Nieuwenhuijse, Bas Oude Munnink, Reina Sikkema, |  |
| --- | --- | --- | --- | --- | --- |
|  |  |  |  | Claudia Schapendonk, Irina Chestakova, Anne van der |  |
|  | Dutch COVID- |  |  | Linden, Mark Pronk, Pascal Lexmond, Corien Swaan, Manon |  |
|  |  |  | Erasmus Medical | Haverkate, Madelief Mollers, Mart Stein, Sandra Kengne |  |
| 2020-03-09 | 19 response |  |  |  |  |
|  |  |  | Center | Kamga Mobou, Jeroen van Kampen, Jolanda Voermans, |  |
|  | team |  |  |  |  |
|  |  |  |  | Aura Timen, Corine GeurtsvanKessel, Annemiek van der |  |
|  |  |  |  |  |  |
|  |  |  |  | Eijk, Richard Molenkamp, Marion Koopmans, on behalf of the |  |
|  |  |  |  | Dutch national COVID-19 response team. |  |
|  |  |  |  |  |  |
|  |  |  |  | David Nieuwenhuijse, Bas Oude Munnink, Reina Sikkema, |  |
|  |  |  |  | Claudia Schapendonk, Irina Chestakova, Anne van der |  |
|  | Dutch COVID- |  |  | Linden, Mark Pronk, Pascal Lexmond, Corien Swaan, Manon |  |
| 2020-03-09 |  |  | Erasmus Medical | Haverkate, Madelief Mollers, Mart Stein, Sandra Kengne |  |
|  | 19 response |  |  |  |  |
|  |  |  | Center | Kamga Mobou, Jeroen van Kampen, Jolanda Voermans, |  |
|  | team |  |  |  |  |
|  |  |  |  | Aura Timen, Corine GeurtsvanKessel, Annemiek van der |  |
|  |  |  |  |  |  |
|  |  |  |  | Eijk, Richard Molenkamp, Marion Koopmans, on behalf of the |  |
|  |  |  |  | Dutch national COVID-19 response team. |  |
|  |  |  |  |  |  |
| 2020-03-12 | UW Virology Lab |  | UW Virology Lab | Pavitra Roychoudhury, Hong Xie, Keith Jerome, Alexander |  |
|  |  |  |  | Greninger |  |
|  |  |  |  |  |  |
|  |  |  |  |  |  |
| 2020-03-11 | UW Virology Lab |  | UW Virology Lab | Pavitra Roychoudhury, Hong Xie, Keith Jerome, Alexander |  |
|  |  |  |  | Greninger |  |
|  |  |  |  |  |  |
|  |  |  |  |  |  |
| 2020-03-11 | UW Virology Lab |  | UW Virology Lab | Pavitra Roychoudhury, Hong Xie, Keith Jerome, Alexander |  |
|  |  |  |  | Greninger |  |
|  |  |  |  |  |  |
|  |  |  |  |  |  |
| 2020-03-11 | UW Virology Lab |  | UW Virology Lab | Pavitra Roychoudhury, Hong Xie, Keith Jerome, Alexander |  |
|  |  |  |  | Greninger |  |
|  |  |  |  |  |  |
|  |  |  |  |  |  |
| 2020-03-10 | UW Virology Lab |  | UW Virology Lab | Pavitra Roychoudhury, Hong Xie, Keith Jerome, Alexander |  |
|  |  |  |  | Greninger |  |
|  |  |  |  |  |  |
| 2020-03-12 | UW Virology Lab |  | UW Virology Lab | Pavitra Roychoudhury, Hong Xie, Keith Jerome, Alexander |  |
|  |  |  |  | Greninger |  |
|  |  |  |  |  |  |
|  |  |  |  |  |  |
| 2020-03-12 | UW Virology Lab |  | UW Virology Lab | Pavitra Roychoudhury, Hong Xie, Keith Jerome, Alexander |  |
|  |  |  |  | Greninger |  |
|  |  |  |  |  |  |
|  |  |  |  |  |  |
| 2020-03-12 | UW Virology Lab |  | UW Virology Lab | Pavitra Roychoudhury, Hong Xie, Keith Jerome, Alexander |  |
|  |  |  |  | Greninger |  |
|  |  |  |  |  |  |
|  |  |  |  |  |  |


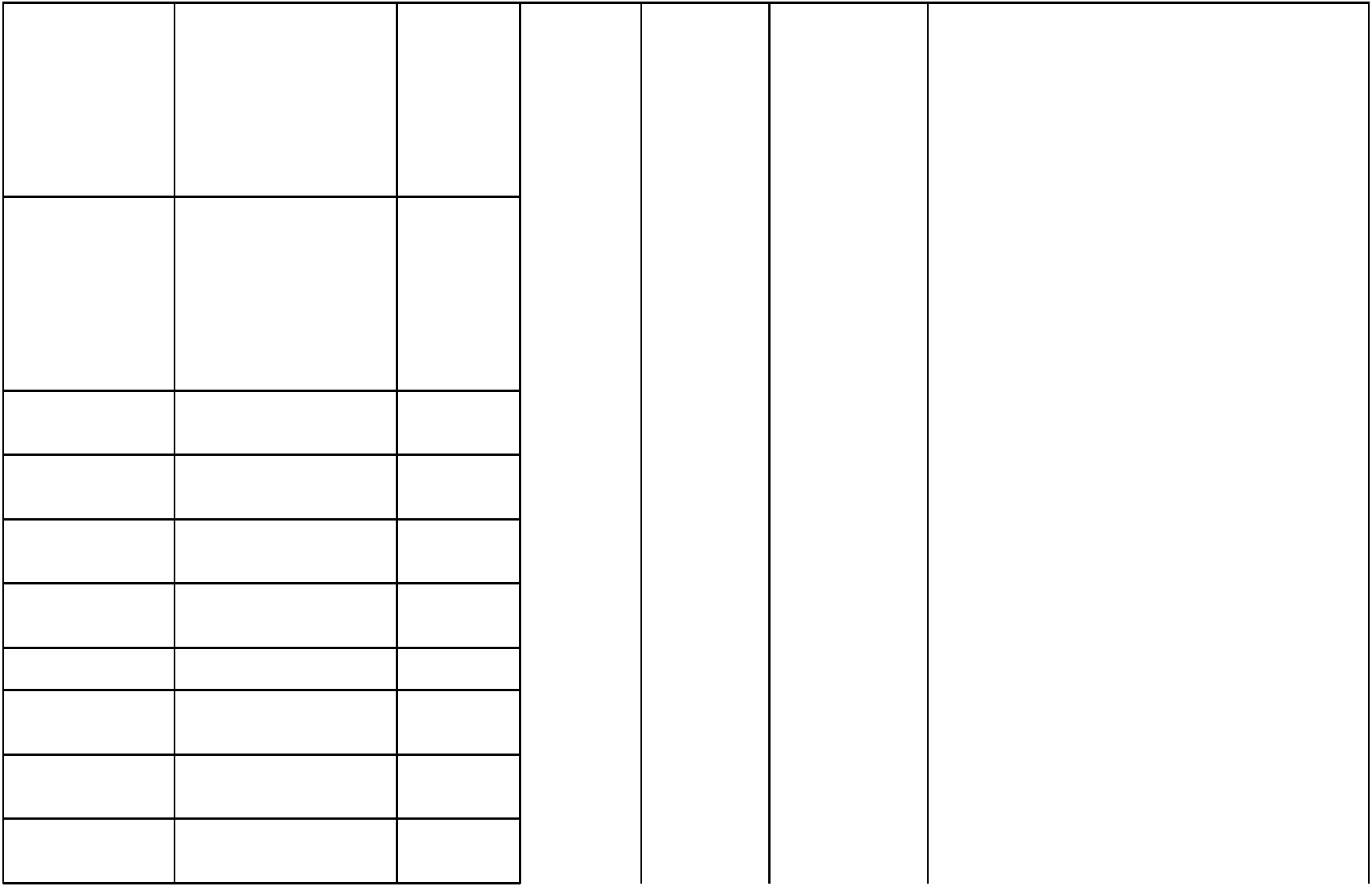


EPI_ISL_416673

EPI_ISL_416684

EPI_ISL_416690

EPI_ISL_416691

EPI_ISL_416692

EPI_ISL_416696

EPI_ISL_416697

EPI_ISL_416698

EPI_ISL_416723

hCoV-19/USA/OR-

UW135/2020

hCoV-19/USA/WA-

UW146/2020

hCoV-19/USA/WA-

UW152/2020

hCoV-19/USA/WA-

UW153/2020

hCoV-19/USA/WA-

UW154/2020

hCoV-19/USA/CT-

UW158/2020

hCoV-19/USA/CT-

UW159/2020

hCoV-19/USA/WA-

UW160/2020

hCoV-19/USA/WA-

UW185/2020

North America / USA / Oregon

North America /

USA /

Washington

North America /

USA /

Washington

North America /

USA /

Washington

North America /

USA /

Washington

North America /

USA /

Connecticut

North America /

USA /

Connecticut

North America /

USA /

Washington

North America /

USA /

Washington

| 2020-03-11 | UW Virology Lab |  | UW Virology Lab |
| --- | --- | --- | --- |
|  |  |  |  |
| 2020-03-14 | UW Virology Lab |  | UW Virology Lab |
|  |  |  |  |
| 2020-03-13 | UW Virology Lab |  | UW Virology Lab |
|  |  |  |  |
| 2020-03-13 | UW Virology Lab |  | UW Virology Lab |
|  |  |  |  |
| 2020-03-14 | UW Virology Lab |  | UW Virology Lab |
|  |  |  |  |
| 2020-03-13 | UW Virology Lab |  | UW Virology Lab |
|  |  |  |  |
| 2020-03-13 | UW Virology Lab |  | UW Virology Lab |
|  |  |  |  |
| 2020-03-13 | UW Virology Lab |  | UW Virology Lab |
|  |  |  |  |
| 2020-03-14 | UW Virology Lab |  | UW Virology Lab |
|  |  |  |  |


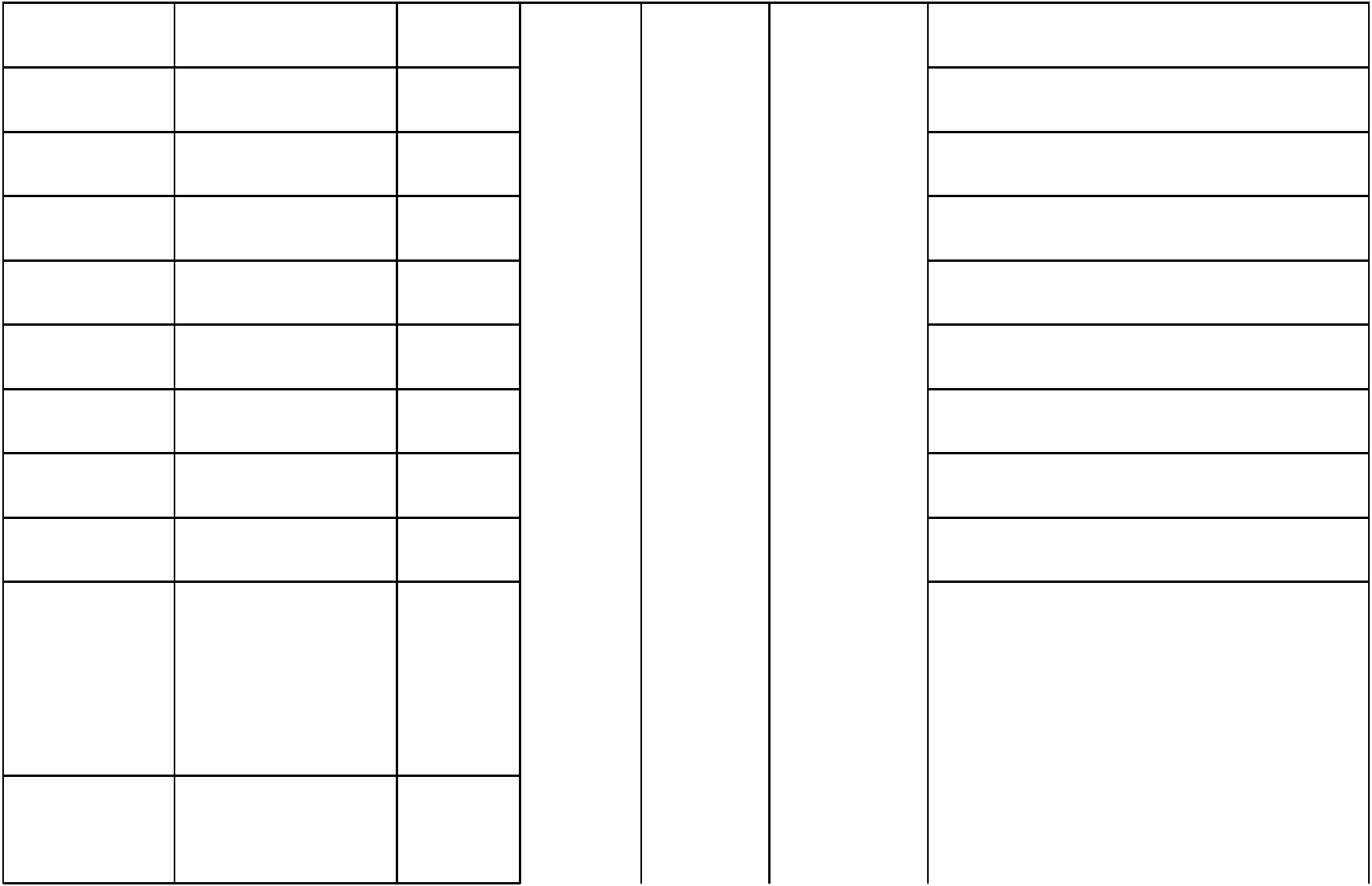


Pavitra Roychoudhury, Hong Xie, Keith Jerome, Alexander Greninger

Pavitra Roychoudhury, Hong Xie, Keith Jerome, Alexander Greninger

Pavitra Roychoudhury, Hong Xie, Keith Jerome, Alexander Greninger

Pavitra Roychoudhury, Hong Xie, Keith Jerome, Alexander Greninger

Pavitra Roychoudhury, Hong Xie, Keith Jerome, Alexander Greninger

Pavitra Roychoudhury, Hong Xie, Keith Jerome, Alexander Greninger

Pavitra Roychoudhury, Hong Xie, Keith Jerome, Alexander Greninger

Pavitra Roychoudhury, Hong Xie, Keith Jerome, Alexander Greninger

Pavitra Roychoudhury, Hong Xie, Keith Jerome, Alexander Greninger

EPI_ISL_416731

EPI_ISL_416750

hCoV-19/England/SHEF-

BFCC0/2020

hCoV-

19/France/Lyon_683/2020

Europe /

England /

Northamtonshire

Europe /

France / ARA

|  | Virology |  | Department of |  |  |
| --- | --- | --- | --- | --- | --- |
|  |  |  | Infection, Immunity |  |  |
|  | Department, |  |  |  |  |
|  |  |  | and Cardiovascular | Thushan de Silva, Matthew Parker, Adri Angyal, Rebecca |  |
|  | Sheffield |  |  |  |  |
| 2020-03-03 |  |  | Disease, The Florey | Brown, Matthew Wyles, Mehmet Yavuz, Mohammad Raza, |  |
|  | Teaching |  |  |  |  |
|  |  |  | Institute, The | Cariad Evans |  |
|  | Hospitals NHS |  |  |  |  |
|  |  |  | Medical School, |  |  |
|  | Foundation Trust |  |  |  |  |
|  |  |  | University of Sheffield |  |  |
|  |  |  |  |  |  |
|  | Institut des |  | CNR Virus des | Bal, Antonin; Destras, Gregory; Gaymard, Alexandre; |  |
|  | Agents |  |  |  |  |
|  |  |  | Infections | Bouscambert-Duchamp, Maude; Cheynet, Valérie; Brengel- |  |
| 2020-03-06 | Infectieux (IAI) |  |  |  |  |
|  |  |  | Respiratoires - | Pesce, Karen; Morfin-Sherpa, Florence; Valette, Martine; |  |
|  | Hospices Civils |  |  |  |  |
|  |  |  | France SUD | Josset, Laurence; Lina, Bruno. |  |
|  | de Lyon |  |  |  |  |
|  |  |  |  |  |  |
|  |  |  |  |  |  |

EPI_ISL_417093

EPI_ISL_417095

EPI_ISL_417096

EPI_ISL_417097

EPI_ISL_417098

EPI_ISL_417099

EPI_ISL_417100

EPI_ISL_417101

EPI_ISL_417107

hCoV-19/USA/WA-S40/2020

hCoV-19/USA/WA-S42/2020

hCoV-19/USA/WA-S43/2020

hCoV-19/USA/WA-S44/2020

hCoV-19/USA/WA-S45/2020

hCoV-19/USA/WA-S46/2020

hCoV-19/USA/WA-S47/2020

hCoV-19/USA/WA-S48/2020

hCoV-19/USA/WA-S54/2020

North America /

USA /

Washington /

Snohomish

County

North America /

USA /

Washington /

King County

North America /

USA /

Washington

North America /

USA /

Washington

North America /

USA /

Washington

North America /

USA /

Washington

North America /

USA /

Washington /

Snohomish

County

North America /

USA /

Washington /

Snohomish

County

North America /

USA /

Washington /

King County

2020-02-28

2020-02-28

2020-02-27

2020-02-28

2020-02-29

2020-02-29

2020-02-29

2020-02-29

2020-03-05

Washington


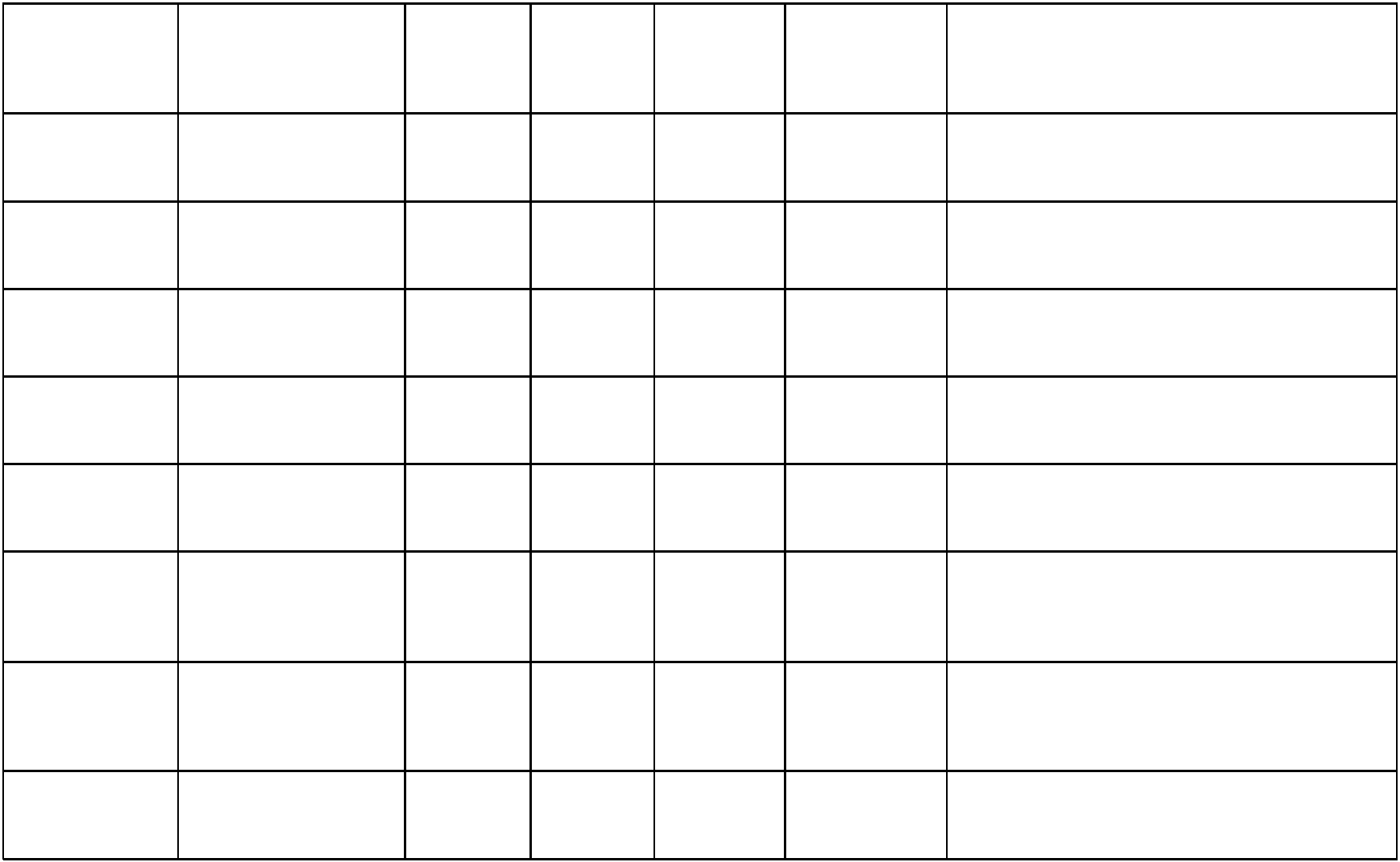


State

Department of

Health

Washington

State

Department of

Health

Washington

State

Department of

Health

Washington

State

Department of

Health

Washington

State

Department of

Health

Washington

State

Department of

Health

Washington

State

Department of

Health

Washington

State

Department of

Health

Washington

State

Department of

Health

Seattle Flu Study

Seattle Flu Study

Seattle Flu Study

Seattle Flu Study

Seattle Flu Study

Seattle Flu Study

Seattle Flu Study

Seattle Flu Study

Seattle Flu Study

Chu etl al

Chu etl al

Chu etl al

Chu etl al

Chu etl al

Chu etl al

Chu etl al

Chu etl al

Chu etl al

EPI_ISL_417108

EPI_ISL_417120

EPI_ISL_417134

EPI_ISL_417135

EPI_ISL_417137

EPI_ISL_417139

EPI_ISL_417142

EPI_ISL_417143

EPI_ISL_417144

hCoV-19/USA/WA-S55/2020

hCoV-19/USA/WA-S67/2020

hCoV-19/USA/WA-S81/2020

hCoV-19/USA/WA-S82/2020

hCoV-19/USA/WA-S84/2020

hCoV-19/USA/WA-S86/2020

hCoV-19/USA/WA-S89/2020

hCoV-19/USA/WA-S90/2020

hCoV-19/USA/WA-S91/2020

North America /

USA /

Washington /

King County

North America /

USA /

Washington /

King County

North America /

USA /

Washington /

King County

North America /

USA /

Washington /

King County

North America /

USA /

Washington /

King County

North America /

USA /

Washington /

King County

North America /

USA /

Washington /

Umatilla County

North America /

USA /

Washington /

Umatilla County

North America /

USA /

Washington /

Snohomish

County

2020-02-29

2020-03-04

2020-02-26

2020-02-22

2020-02-21

2020-03-01

2020-02-29

2020-02-29

2020-03-02

Washington


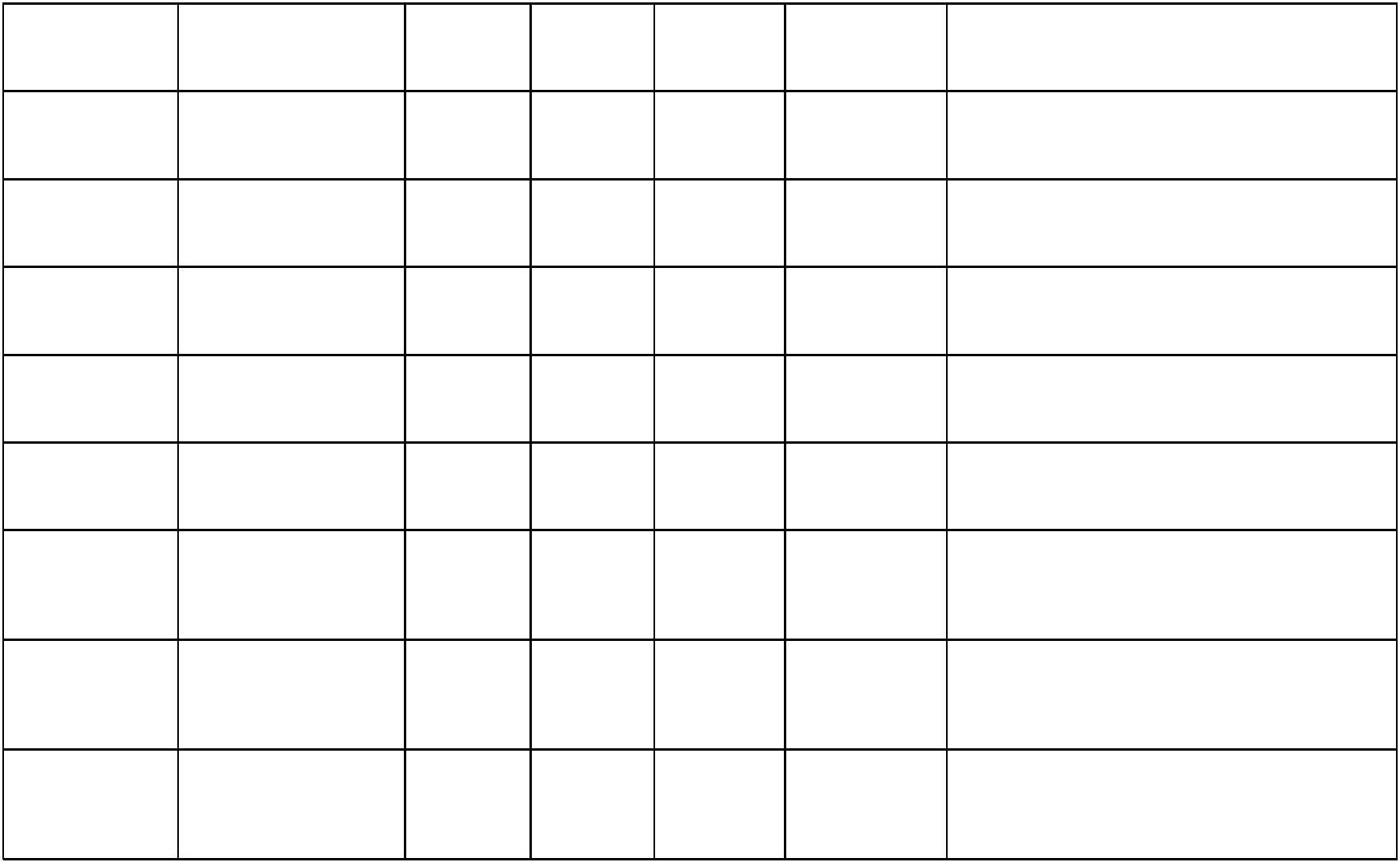


State

Department of

Health

Washington

State

Department of

Health

Washington

State

Department of

Health

Washington

State

Department of

Health

Washington

State

Department of

Health

Washington

State

Department of

Health

Washington

State

Department of

Health

Washington

State

Department of

Health

Washington

State

Department of

Health

Seattle Flu Study

Seattle Flu Study

Seattle Flu Study

Seattle Flu Study

Seattle Flu Study

Seattle Flu Study

Seattle Flu Study

Seattle Flu Study

Seattle Flu Study

Chu etl al

Chu etl al

Chu etl al

Chu etl al

Chu etl al

Chu etl al

Chu etl al

Chu etl al

Chu etl al

EPI_ISL_417145

EPI_ISL_417146

EPI_ISL_417147

EPI_ISL_417148

EPI_ISL_417149

EPI_ISL_417150

EPI_ISL_417151

EPI_ISL_417152

EPI_ISL_417153

EPI_ISL_417154

hCoV-19/USA/WA-S92/2020

hCoV-19/USA/WA-S93/2020

hCoV-19/USA/WA-S94/2020

hCoV-19/USA/WA-S95/2020

hCoV-19/USA/WA-S96/2020

hCoV-19/USA/WA-S97/2020

hCoV-19/USA/WA-S98/2020

hCoV-19/USA/WA-S99/2020

hCoV-19/USA/WA-S100/2020

hCoV-19/USA/WA-S101/2020

North America /

USA /

Washington /

King County

North America /

USA /

Washington /

King County

North America /

USA /

Washington /

Snohomish

County

North America /

USA /

Washington /

King County

North America /

USA /

Washington /

King County

North America /

USA /

Washington /

King County

North America /

USA /

Washington /

King County

North America /

USA /

Washington /

King County

North America /

USA /

Washington /

King County

North America /

USA /

Washington /

King County

2020-02-29

2020-02-29

2020-02-28

2020-02-28

2020-02-28

2020-02-28

2020-02-29

2020-02-28

2020-02-29

2020-02-28

Washington


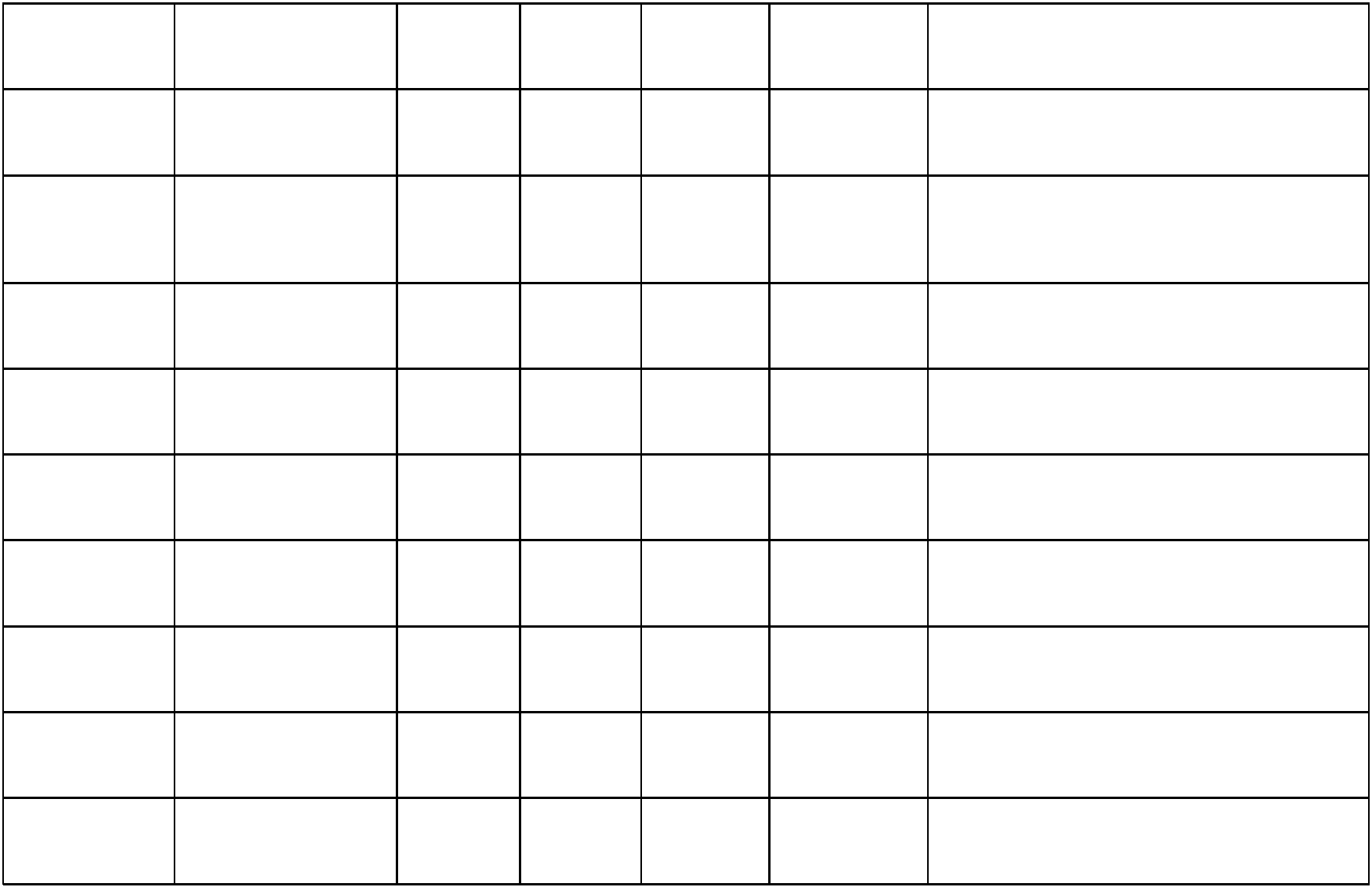


State

Department of

Health

Washington

State

Department of

Health

Washington

State

Department of

Health

Washington

State

Department of

Health

Washington

State

Department of

Health

Washington

State

Department of

Health

Washington

State

Department of

Health

Washington

State

Department of

Health

Washington

State

Department of

Health

Washington

State

Department of

Health

Seattle Flu Study

Seattle Flu Study

Seattle Flu Study

Seattle Flu Study

Seattle Flu Study

Seattle Flu Study

Seattle Flu Study

Seattle Flu Study

Seattle Flu Study

Seattle Flu Study

Chu etl al

Chu etl al

Chu etl al

Chu etl al

Chu etl al

Chu etl al

Chu etl al

Chu etl al

Chu etl al

Chu etl al

EPI_ISL_417155

EPI_ISL_417156

EPI_ISL_417158

EPI_ISL_417159

EPI_ISL_417160

EPI_ISL_417161

EPI_ISL_417162

EPI_ISL_417168

EPI_ISL_417169

EPI_ISL_417172

hCoV-19/USA/WA-S102/2020

hCoV-19/USA/WA-S103/2020

hCoV-19/USA/WA-S105/2020

hCoV-19/USA/WA-S106/2020

hCoV-19/USA/WA-S107/2020

hCoV-19/USA/WA-S108/2020

hCoV-19/USA/WA-S109/2020

hCoV-19/USA/WA-S115/2020

hCoV-19/USA/WA-S116/2020

hCoV-19/USA/WA-S119/2020

North America /

USA /

Washington /

King County

North America /

USA /

Washington /

King County

North America /

USA /

Washington /

King County

North America /

USA /

Washington /

King County

North America /

USA /

Washington /

King County

North America /

USA /

Washington /

King County

North America /

USA /

Washington /

Snohomish

County

North America /

USA /

Washington /

King County

North America /

USA /

Washington

North America /

USA /

Washington /

King County

2020-02-28

2020-02-28

2020-02-28

2020-02-29

2020-02-29

2020-02-29

2020-03-01

2020-02-29

2020-03-02

2020-02-29

Washington


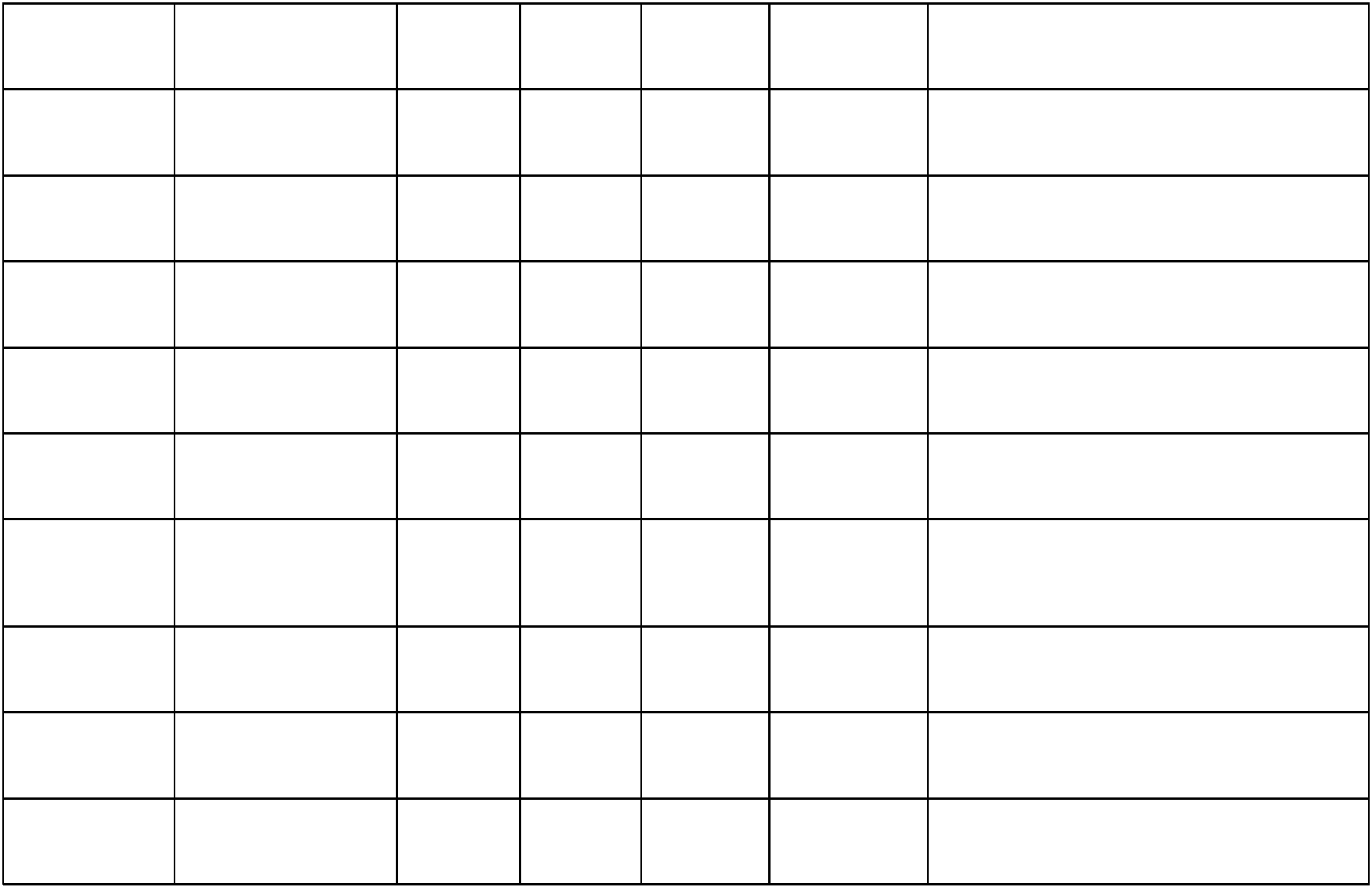


State

Department of

Health

Washington

State

Department of

Health

Washington

State

Department of

Health

Washington

State

Department of

Health

Washington

State

Department of

Health

Washington

State

Department of

Health

Washington

State

Department of

Health

Washington

State

Department of

Health

Washington

State

Department of

Health

Washington

State

Department of

Health

Seattle Flu Study

Seattle Flu Study

Seattle Flu Study

Seattle Flu Study

Seattle Flu Study

Seattle Flu Study

Seattle Flu Study

Seattle Flu Study

Seattle Flu Study

Seattle Flu Study

Chu etl al

Chu etl al

Chu etl al

Chu etl al

Chu etl al

Chu etl al

Chu etl al

Chu etl al

Chu etl al

Chu etl al

EPI_ISL_417192

EPI_ISL_417194

EPI_ISL_417196

EPI_ISL_417198

EPI_ISL_417213

EPI_ISL_417254

EPI_ISL_417921

hCoV-19/USA/MN25-

MDH25/2020

hCoV-19/USA/MN26-

MDH26/2020

hCoV-19/USA/MN29-

MDH29/2020

hCoV-19/USA/MN30-

MDH30/2020

hCoV-

19/England/20102068502/202

0

hCoV-

19/England/20108006802/202

0

hCoV-19/Italy/INMI3/2020

North America /

USA /

Minnesota

North America /

USA /

Minnesota

North America /

USA /

Minnesota

North America /

USA /

Minnesota

Europe / United Kingdom / England

Europe / United Kingdom / England

Europe / Italy /

Rome

2020-03-12

2020-03-12

2020-03-11

2020-03-12

2020-03-01

2020-03-04

2020-03-01

Minnesota


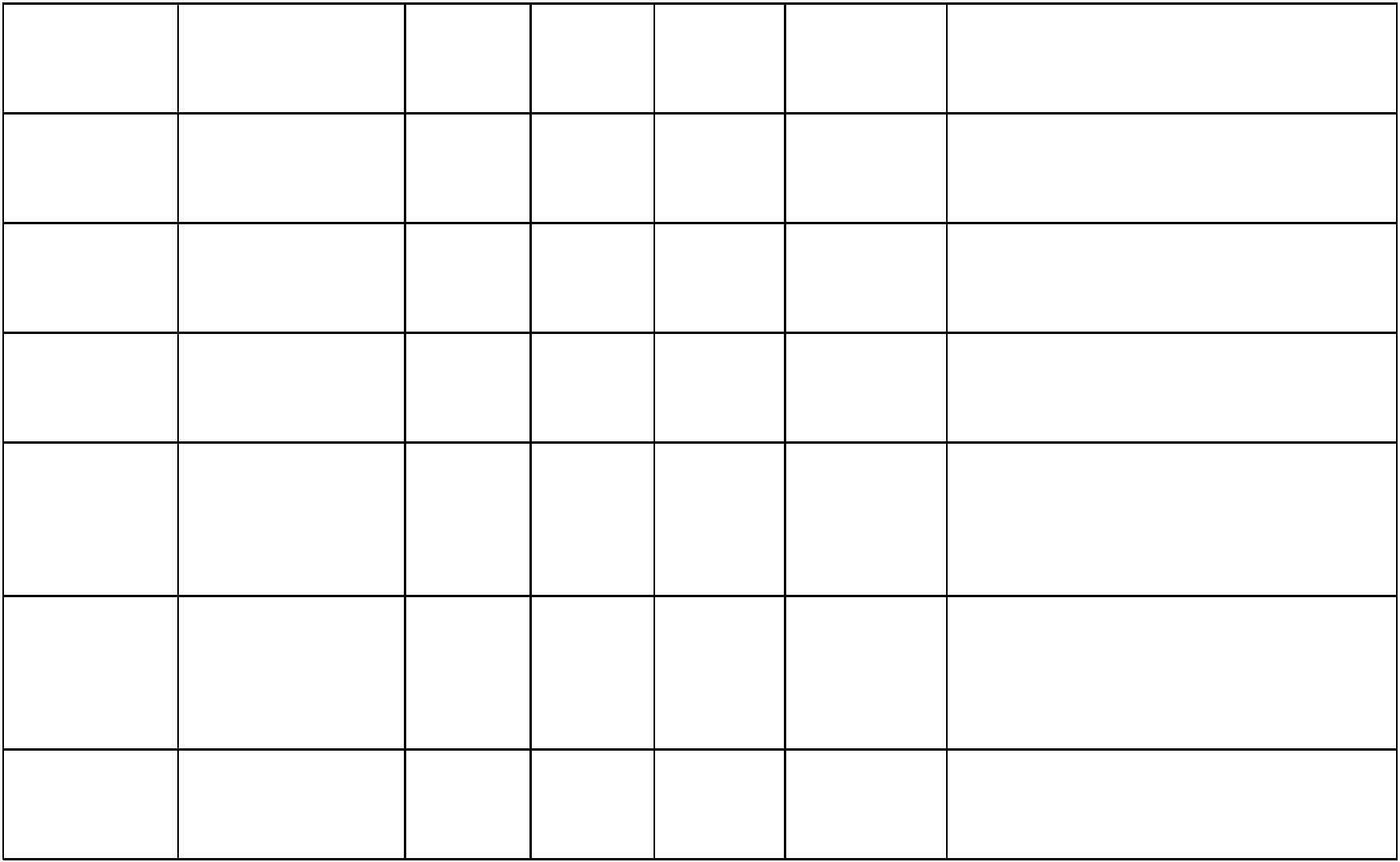


Department of

Health, Public

Health

Laboratory

Minnesota

Department of

Health, Public

Health

Laboratory

Minnesota

Department of

Health, Public

Health

Laboratory

Minnesota

Department of

Health, Public

Health

Laboratory

Respiratory Virus Unit, Microbiology Services Colindale, Public Health England

Respiratory Virus Unit, Microbiology Services Colindale, Public Health England

INMI Lazzaro Spallanzani IRCCS

Minnesota

Department of

Health, Public Health

Laboratory

Minnesota

Department of

Health, Public Health

Laboratory

Minnesota

Department of

Health, Public Health

Laboratory

Minnesota

Department of

Health, Public Health

Laboratory

Respiratory Virus Unit, Microbiology Services Colindale, Public Health England

Respiratory Virus Unit, Microbiology Services Colindale, Public Health England

Laboratory of Virology, INMI Lazzaro Spallanzani IRCCS

Matt Plumb, Jake Garfin and Xiong Wang

Matt Plumb, Jake Garfin and Xiong Wang

Matt Plumb, Jake Garfin and Xiong Wang

Matt Plumb, Jake Garfin and Xiong Wang

Monica Galiano, Shahjahan Miah, Angie Lackenby, Omolola Akinbami, Tiina Talts, Leena Bhaw, Richard Myers, Steven Platt, Kirstin Edwards, Jonathan Hubb, Joanna Ellis, Maria Zambon

Monica Galiano, Shahjahan Miah, Angie Lackenby, Omolola Akinbami, Tiina Talts, Leena Bhaw, Richard Myers, Steven Platt, Kirstin Edwards, Jonathan Hubb, Joanna Ellis, Maria Zambon

Martina Rueca, Barbara Bartolini, Francesco Messina,

Cesare E. M. Gruber, Emanuela Giombini, Maria R.

Capobianchi, Fabrizio Carletti, Francesca Colavita, Concetta Castilletti, Eleonora Lalle, Daniele Lapa, Giuseppe Ippolito.

EPI_ISL_417922

EPI_ISL_417923

EPI_ISL_417924

hCoV-19/Italy/INMI4/2020

hCoV-19/Italy/INMI5/2020

hCoV-

19/Colombia/Antioquia79256/2

020

Europe / Italy /

Rome

Europe / Italy /

Rome

South America / Colombia / Antioquia

2020-02-28

2020-03-04

2020-03-11

INMI Lazzaro Spallanzani IRCCS


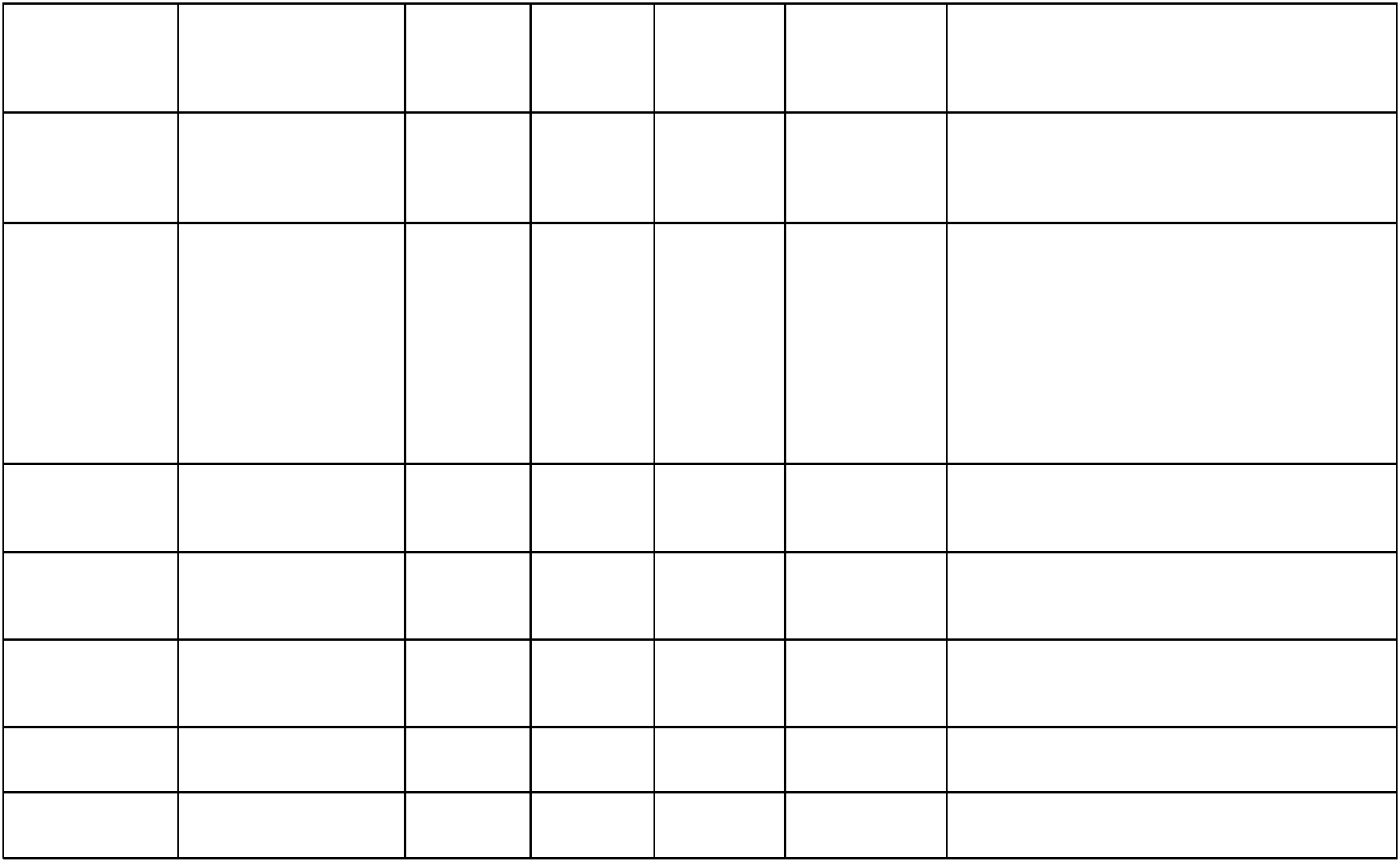


INMI Lazzaro Spallanzani IRCCS

Secretaría de Salud Medellín

Laboratory of Virology, INMI Lazzaro Spallanzani IRCCS

Laboratory of Virology, INMI Lazzaro Spallanzani IRCCS

Instituto Nacional de Salud, Universidad Cooperativa de Colombia, Instituto Alexander von Humboldt, Imperial College-London, London School of Hygiene & Tropical Medicine

Cesare E. M. Gruber, Martina Rueca, Barbara Bartolini,

Francesco Messina, Emanuela Giombini, Maria R.

Capobianchi, Fabrizio Carletti, Francesca Colavita, Concetta Castilletti, Eleonora Lalle, Daniele Lapa, Giuseppe Ippolito.

Francesco Messina, Barbara Bartolini, Martina Rueca,

Cesare E. M. Gruber, Emanuela Giombini, Maria R.

Capobianchi, Fabrizio Carletti, Francesca Colavita, Concetta Castilletti, Eleonora Lalle, Daniele Lapa, Giuseppe Ippolito.

Marcela Mercado-Reyes, Katherine Laiton-Donato, Diego A. Álvarez-Díaz, Carlos Franco-Muñoz, Jose A. Usme-Ciro, Gloria Puerto, Nicolás D. Franco-Sierra, Mailyn A. Gonzalez,

Zulma M. Cucunubá, Christian Julian Villabona‐Arenas, Liz

Villabona-Arenas, Sussy Echeverria-Londoño, Astrid C. Flórez, Sergio Gomez Rangel, Luz Dary Rodriguez, Juliana Barbosa, Erika Ospitia, Diana Marcela Walteros-Acero, Martha Lucia Ospina Martinez

EPI_ISL_417954

EPI_ISL_417956

EPI_ISL_417957

EPI_ISL_417958

EPI_ISL_417960

hCoV-

19/Spain/Madrid_H3_10/2020

hCoV-

19/Spain/Madrid_H5_34/2020

hCoV-

19/Spain/Madrid_H7_36/2020

hCoV-19/USA/UT-00010/2020

hCoV-19/USA/UT-00012/2020

Europe / Spain

/ Madrid

Europe / Spain

/ Madrid

Europe / Spain

/ Madrid

North America /

USA / Utah

North America /

USA / Utah

2020-03-12

2020-03-11

2020-03-12

2020-03-10

2020-03-12

Hospital Universitario 12 de Octubre

Hospital Universitario 12 de Octubre

Hospital Universitario 12 de Octubre

Utah Public

Health

Laboratory

Utah Public Health Laboratory

Hospital

Universitario La Paz

Hospital

Universitario La Paz

Hospital

Universitario La Paz

Utah Public Health

Laboratory

Utah Public Health

Laboratory

Elias Dahdouh, Sara González, Fernando Lázaro, Esther Viedma, Natalia Stella, Julio García, Juan Carlos Galán, Rafael Cantón, Mª Dolores Folgueira, Rafael Delgado, Jesús Mingorance

Elias Dahdouh, Sara González, Fernando Lázaro, Esther Viedma, Natalia Stella, Julio García, Juan Carlos Galán, Rafael Cantón, Mª Dolores Folgueira, Rafael Delgado, Jesús Mingorance

Elias Dahdouh, Sara González, Fernando Lázaro, Esther Viedma, Natalia Stella, Julio García, Juan Carlos Galán, Rafael Cantón, Mª Dolores Folgueira, Rafael Delgado, Jesús Mingorance

Erin Young, Kelly Oakeson

Erin Young, Kelly Oakeson

EPI_ISL_417963

EPI_ISL_417964

EPI_ISL_417966

EPI_ISL_417967

EPI_ISL_417972

EPI_ISL_417975

EPI_ISL_417978

EPI_ISL_417979

EPI_ISL_417980

EPI_ISL_417981

hCoV-

19/Spain/Madrid_H10_39/202

0

hCoV-19/USA/UT-00014/2020

hCoV-19/USA/UT-00016/2020

hCoV-

19/Spain/Madrid_H11_40/202

0

hCoV-

19/Spain/Madrid_LP12_21/20

20

hCoV-

19/Spain/Madrid_LP14_3/202

0

hCoV-

19/Spain/Madrid_LP15_4/202

0

hCoV-

19/Spain/Madrid_R2_15/2020

hCoV-

19/Spain/Madrid_R5_8/2020

hCoV-

19/Spain/Madrid_R10_33/202

0

Europe / Spain

/ Madrid

North America /

USA / Utah

North America /

USA / Utah

Europe / Spain

/ Madrid

Europe / Spain

/ Madrid

Europe / Spain

/ Madrid

Europe / Spain

/ Madrid

Europe / Spain

/ Madrid

Europe / Spain

/ Madrid

Europe / Spain

/ Madrid

2020-03-12

2020-03-13

2020-03-13

2020-03-12

2020-03-09

2020-03-09

2020-03-09

2020-03-03

2020-03-03

2020-03-02

Hospital Universitario 12 de Octubre


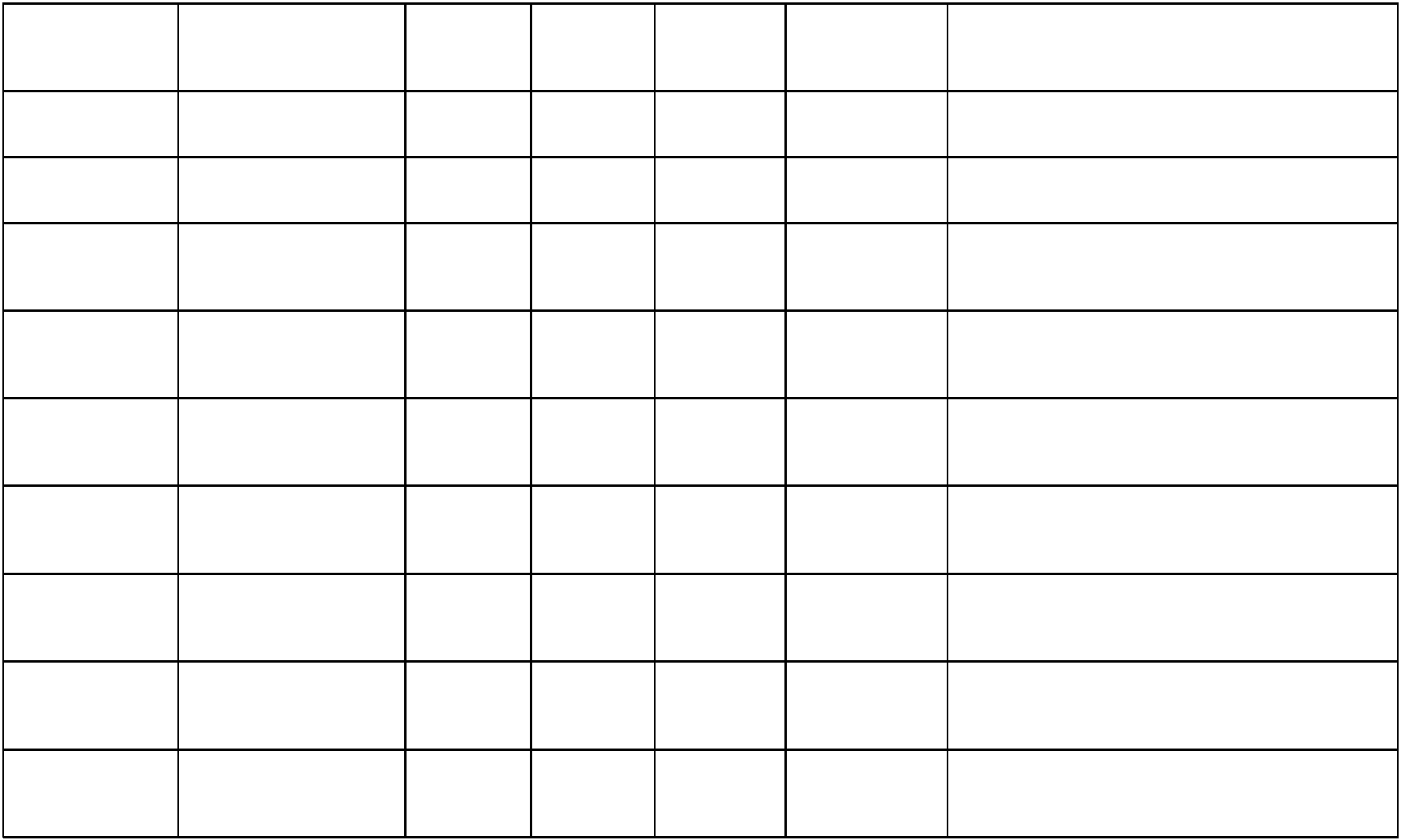


Utah Public Health Laboratory

Utah Public Health Laboratory

Hospital Universitario 12 de Octubre

Hospital Universitario La Paz

Hospital Universitario La Paz

Hospital Universitario La Paz

Hospital

Universitario

Ramón y Cajal

Hospital

Universitario

Ramón y Cajal

Hospital

Universitario

Ramón y Cajal

Hospital

Universitario La Paz

Utah Public Health

Laboratory

Utah Public Health

Laboratory

Hospital

Universitario La Paz

Hospital

Universitario La Paz

Hospital

Universitario La Paz

Hospital

Universitario La Paz

Hospital

Universitario La Paz

Hospital

Universitario La Paz

Hospital

Universitario La Paz

Elias Dahdouh, Sara González, Fernando Lázaro, Esther Viedma, Natalia Stella, Julio García, Juan Carlos Galán, Rafael Cantón, Mª Dolores Folgueira, Rafael Delgado, Jesús Mingorance

Erin Young, Kelly Oakeson

Erin Young, Kelly Oakeson

Elias Dahdouh, Sara González, Fernando Lázaro, Esther Viedma, Natalia Stella, Julio García, Juan Carlos Galán, Rafael Cantón, Mª Dolores Folgueira, Rafael Delgado, Jesús Mingorance

Elias Dahdouh, Sara González, Fernando Lázaro, Esther Viedma, Natalia Stella, Julio García, Juan Carlos Galán, Rafael Cantón, Mª Dolores Folgueira, Rafael Delgado, Jesús Mingorance

Elias Dahdouh, Sara González, Fernando Lázaro, Esther Viedma, Natalia Stella, Julio García, Juan Carlos Galán, Rafael Cantón, Mª Dolores Folgueira, Rafael Delgado, Jesús Mingorance

Elias Dahdouh, Sara González, Fernando Lázaro, Esther Viedma, Natalia Stella, Julio García, Juan Carlos Galán, Rafael Cantón, Mª Dolores Folgueira, Rafael Delgado, Jesús Mingorance

Elias Dahdouh, Sara González, Fernando Lázaro, Esther Viedma, Natalia Stella, Julio García, Juan Carlos Galán, Rafael Cantón, Mª Dolores Folgueira, Rafael Delgado, Jesús Mingorance

Elias Dahdouh, Sara González, Fernando Lázaro, Esther Viedma, Natalia Stella, Julio García, Juan Carlos Galán, Rafael Cantón, Mª Dolores Folgueira, Rafael Delgado, Jesús Mingorance

Elias Dahdouh, Sara González, Fernando Lázaro, Esther Viedma, Natalia Stella, Julio García, Juan Carlos Galán, Rafael Cantón, Mª Dolores Folgueira, Rafael Delgado, Jesús Mingorance

EPI_ISL_417986

EPI_ISL_417987

EPI_ISL_417988

EPI_ISL_417989

EPI_ISL_417990

EPI_ISL_417992

EPI_ISL_417993

EPI_ISL_417994

EPI_ISL_417995

EPI_ISL_417996

EPI_ISL_417997

EPI_ISL_417998

hCoV-

19/Portugal/PT0001b/2020

hCoV-

19/Portugal/PT0003/2020

hCoV-

19/Portugal/PT0004/2020

hCoV-

19/Portugal/PT0005/2020

hCoV-

19/Portugal/PT0006a/2020

hCoV-

19/Portugal/PT0007/2020

hCoV-

19/Portugal/PT0008/2020

hCoV-

19/Portugal/PT0009/2020

hCoV-

19/Portugal/PT0010/2020

hCoV-

19/Portugal/PT0011/2020

hCoV-

19/Portugal/PT0012/2020

hCoV-

19/Portugal/PT0013/2020

Europe /

Portugal

Europe /

Portugal

Europe /

Portugal

Europe /

Portugal

Europe /

Portugal

Europe /

Portugal

Europe /

Portugal

Europe /

Portugal

Europe /

Portugal

Europe /

Portugal

Europe /

Portugal

Europe /

Portugal

2020-03-03

2020-03-03

2020-03-05

2020-03-04

2020-03-06

2020-03-07

2020-03-08

2020-03-08

2020-03-08

2020-03-08

2020-03-07

2020-03-08

Centro


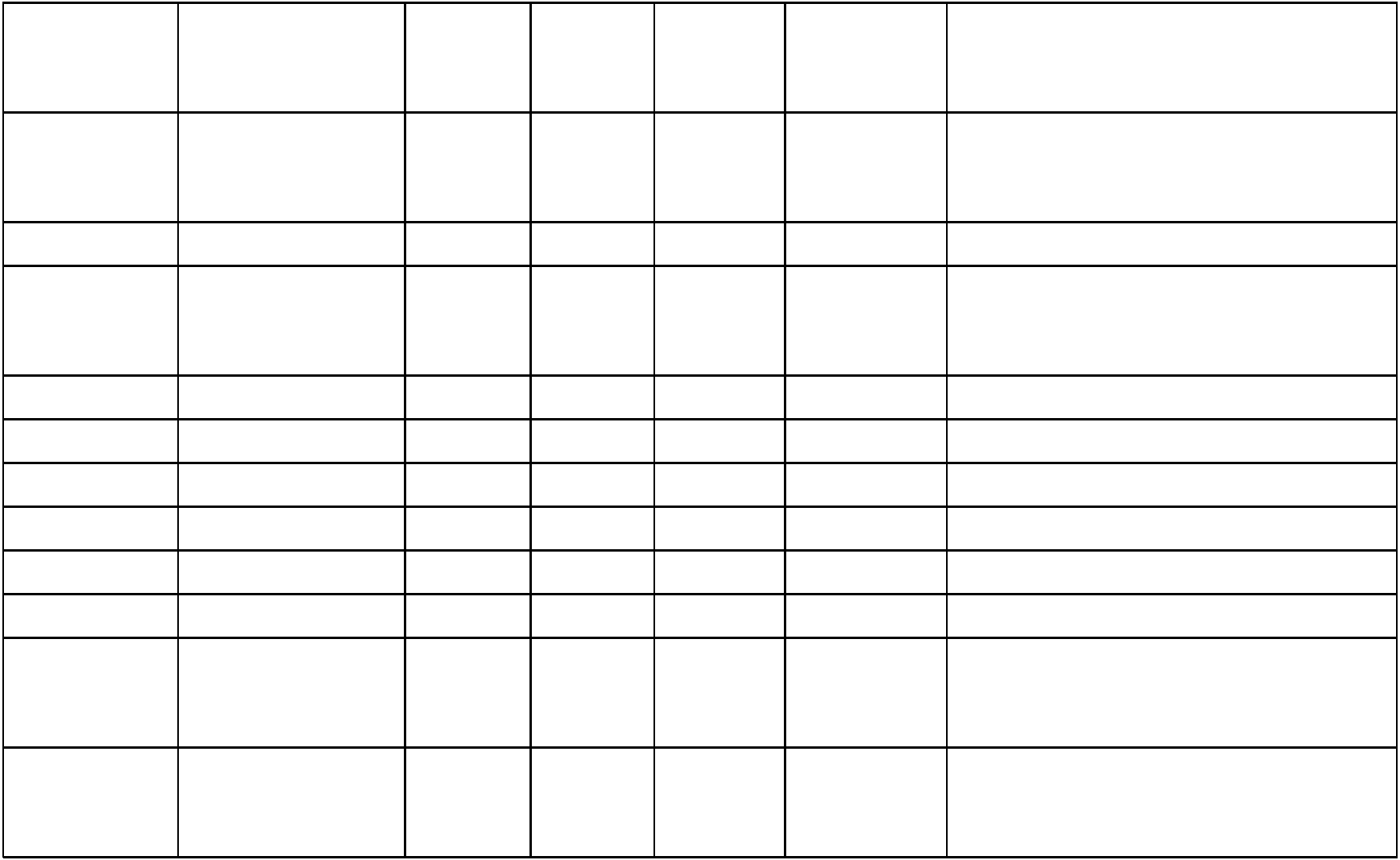


Hospitalar e

Universitario de Sao Joao, Porto

Centro

Hospitalar e

Universitario de Sao Joao, Porto

CHULC - H Curry Cabral

Centro

Hospitalar e

Universitario de Sao Joao, Porto

CHULC - H Curry Cabral

CHULC - H D

Estefania

CHULC - H D

Estefania

CHULC - H Curry Cabral

CHULC - H Curry Cabral

CHULC - H Curry Cabral

Centro Hospital do Porto, E.P.E.

- H. Geral de Santo Antonio

Centro Hospital do Porto, E.P.E.

- H. Geral de Santo Antonio

Instituto Nacional de

Saude (INSA)

Instituto Nacional de

Saude (INSA)

Instituto Nacional de

Saude (INSA)

Instituto Nacional de

Saude (INSA)

Instituto Nacional de

Saude (INSA)

Instituto Nacional de

Saude (INSA)

Instituto Nacional de

Saude (INSA)

Instituto Nacional de

Saude (INSA)

Instituto Nacional de

Saude (INSA)

Instituto Nacional de

Saude (INSA)

Instituto Nacional de

Saude (INSA)

Instituto Nacional de

Saude (INSA)

Guiomar et al

Guiomar et al

Guiomar et al

Guiomar et al

Guiomar et al

Guiomar et al

Guiomar et al

Guiomar et al

Guiomar et al

Guiomar et al

Guiomar et al

Guiomar et al

| EPI_ISL_417999 |  | hCoV- |  | Europe / | 2020-03-07 | |  |
| --- | --- | --- | --- | --- | --- | --- | --- |
|  |  | 19/Portugal/PT0014/2020 |  | Portugal |  |  |  |
|  |  |  |  |  |  |  |  |
|  |  |  |  |  |  |  |  |

EPI_ISL_418401 hCoV-19/Finland/14M12/2020 Europe / Finland 2020-03-14

EPI_ISL_418404 hCoV-19/Finland/14M16/2020 Europe / Finland 2020-03-14

EPI_ISL_418406 hCoV-19/Finland/14M26/2020 Europe / Finland 2020-03-14

Europe /

EPI_ISL_418408 hCoV-19/Finland/14M32/2020 Finland / 2020-03-14

Helsinki

Centro Hospital do Porto, E.P.E.


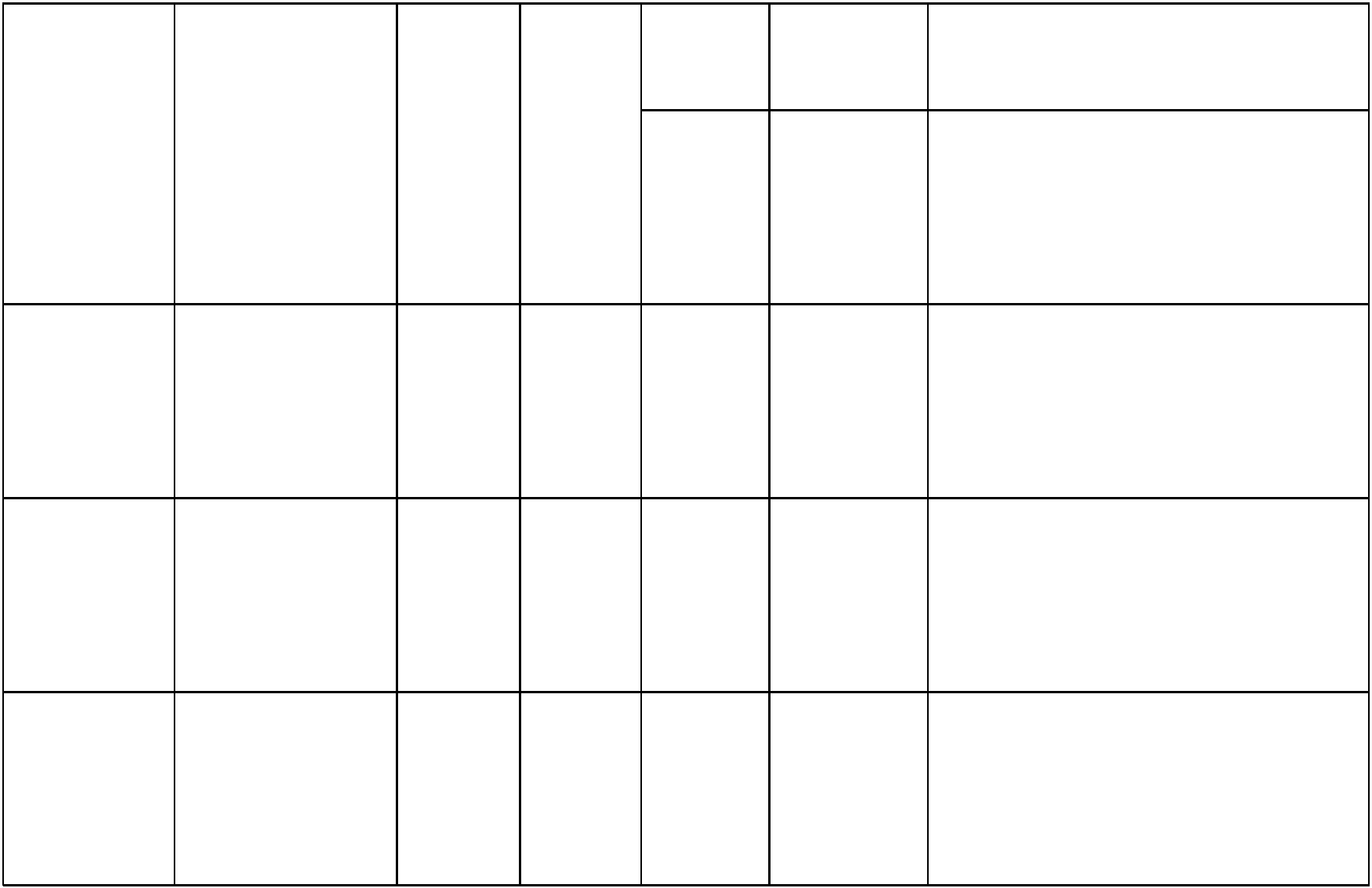


- H. Geral de Santo Antonio

Department of

Virology and

Immunology,

University of

Helsinki and

Helsinki

University

Hospital, Huslab

Finland

Department of

Virology and

Immunology,

University of

Helsinki and

Helsinki

University

Hospital, Huslab

Finland

Department of

Virology and

Immunology,

University of

Helsinki and

Helsinki

University

Hospital, Huslab

Finland

Department of

Virology and

Immunology,

University of

Helsinki and

Helsinki

University

Hospital, Huslab

Finland

Instituto Nacional de Saude (INSA)

Department of Virology, Faculty of Medicine, University of Helsinki, Helsinki, Finland

Department of Virology, Faculty of Medicine, University of Helsinki, Helsinki, Finland

Department of Virology, Faculty of Medicine, University of Helsinki, Helsinki, Finland

Department of Virology, Faculty of Medicine, University of Helsinki, Helsinki, Finland

Guiomar et al

Teemu Smura, Hannimari Kallio-Kokko, Olli Vapalahti

Teemu Smura, Hannimari Kallio-Kokko, Olli Vapalahti

Teemu Smura, Hannimari Kallio-Kokko, Olli Vapalahti

Teemu Smura, Hannimari Kallio-Kokko, Olli Vapalahti

EPI_ISL_418409

EPI_ISL_418410

EPI_ISL_418441

EPI_ISL_418442

EPI_ISL_418502

EPI_ISL_418503

hCoV-19/Finland/14M74/2020

hCoV-19/Finland/14M77/2020

hCoV-

19/Hangzhou/HZ48/2020

hCoV-

19/Hangzhou/HZ49/2020

hCoV-

19/Hangzhou/HZ60/2020

hCoV-

19/Hangzhou/HZ62/2020

Europe /

Finland /

Helsinki

Europe /

Finland /

Helsinki

Asia / China / Hangzhou

Asia / China / Hangzhou

Asia / China / Hangzhou

Asia / China / Hangzhou

|  | Department of |  |  |  |
| --- | --- | --- | --- | --- |
|  | Virology and |  |  |  |
|  | Immunology, |  | Department of |  |
|  | University of |  | Virology, Faculty of |  |
| 2020-03-14 | Helsinki and |  | Medicine, University |  |
|  | Helsinki |  | of Helsinki, Helsinki, |  |
|  | University |  | Finland |  |
|  | Hospital, Huslab |  |  |  |
|  | Finland |  |  |  |
|  |  |  |  |  |
|  | Department of |  |  |  |
|  | Virology and |  | Department of |  |
|  | Immunology, |  |  |  |
| 2020-03-14 | University of |  | Virology, Faculty of |  |
|  | Helsinki and |  | Medicine, University |  |
|  | Helsinki |  | of Helsinki, Helsinki, |  |
|  | University |  | Finland |  |
|  | Hospital, Huslab |  |  |  |
|  | Finland |  |  |  |
|  |  |  |  |  |
|  | Hangzhou |  | Inspection Center of |  |
| 2020-01-21 | Center for |  | Hangzhou Center for |  |
|  | Disease Control |  | Disease Control and |  |
|  |  |  |  |  |
|  | and Prevention |  | Prevention |  |
|  |  |  |  |  |
|  | Hangzhou |  | Inspection Center of |  |
| 2020-01-21 | Center for |  | Hangzhou Center for |  |
|  | Disease Control |  | Disease Control and |  |
|  |  |  |  |  |
|  | and Prevention |  | Prevention |  |
|  |  |  |  |  |
|  | Hangzhou |  | Inspection Center of |  |
| 2020-01-22 | Center for |  | Hangzhou Center for |  |
|  | Disease Control |  | Disease Control and |  |
|  |  |  |  |  |
|  | and Prevention |  | Prevention |  |
|  |  |  |  |  |
|  | Hangzhou |  | Inspection Center of |  |
| 2020-01-22 | Center for |  | Hangzhou Center for |  |
|  | Disease Control |  | Disease Control and |  |
|  |  |  |  |  |
|  | and Prevention |  | Prevention |  |
|  |  |  |  |  |


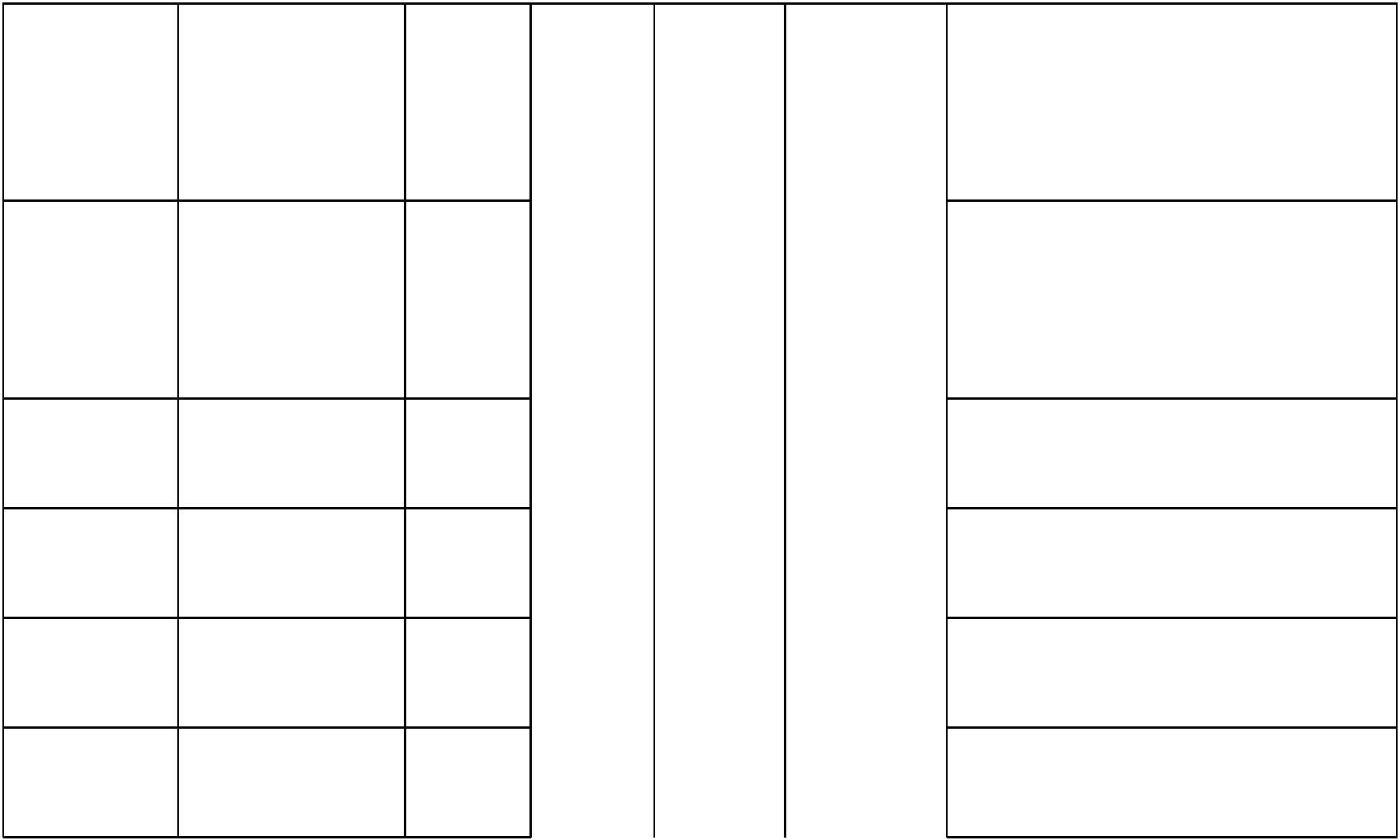


Teemu Smura, Hannimari Kallio-Kokko, Olli Vapalahti

Teemu Smura, Hannimari Kallio-Kokko, Olli Vapalahti

Yu hua, Wang haoqiu, Li jun, Yu xinfeng, Pan jingcao

Yu hua, Wang haoqiu, Li jun, Yu xinfeng, Pan jingcao

Yu hua, Wang haoqiu, Li jun, Yu xinfeng, Pan jingcao

Yu hua, Wang haoqiu, Li jun, Yu xinfeng, Pan jingcao

EPI_ISL_418504

EPI_ISL_418506

EPI_ISL_418507

EPI_ISL_418508

EPI_ISL_418509

EPI_ISL_418510

EPI_ISL_418511

EPI_ISL_418512

hCoV-

19/Hangzhou/HZ79/2020

hCoV-

19/Hangzhou/HZ90/2020

hCoV-

19/Hangzhou/HZ91/2020

hCoV-

19/Hangzhou/HZ162/2020

hCoV-

19/Hangzhou/HZ178/2020

hCoV-

19/Hangzhou/HZ185/2020

hCoV-

19/Hangzhou/HZ477/2020

hCoV-

19/Hangzhou/HZ481/2020

Asia / China / Hangzhou

Asia / China / Hangzhou

Asia/China/Zheji

ang/Hangzhou

Asia / China / Zhejiang / Hangzhou

Asia / China / Zhejiang / Hangzhou

Asia / China / Hangzhou

Asia / China / Zhejiang / Hangzhou

Asia / China / Zhejiang / Hangzhou

2020-01-21

2020-01-21

2020-01-21

2020-01-23

2020-01-23

2020-01-23

2020-01-24

2020-01-25

Hangzhou Center for Disease Control and Prevention


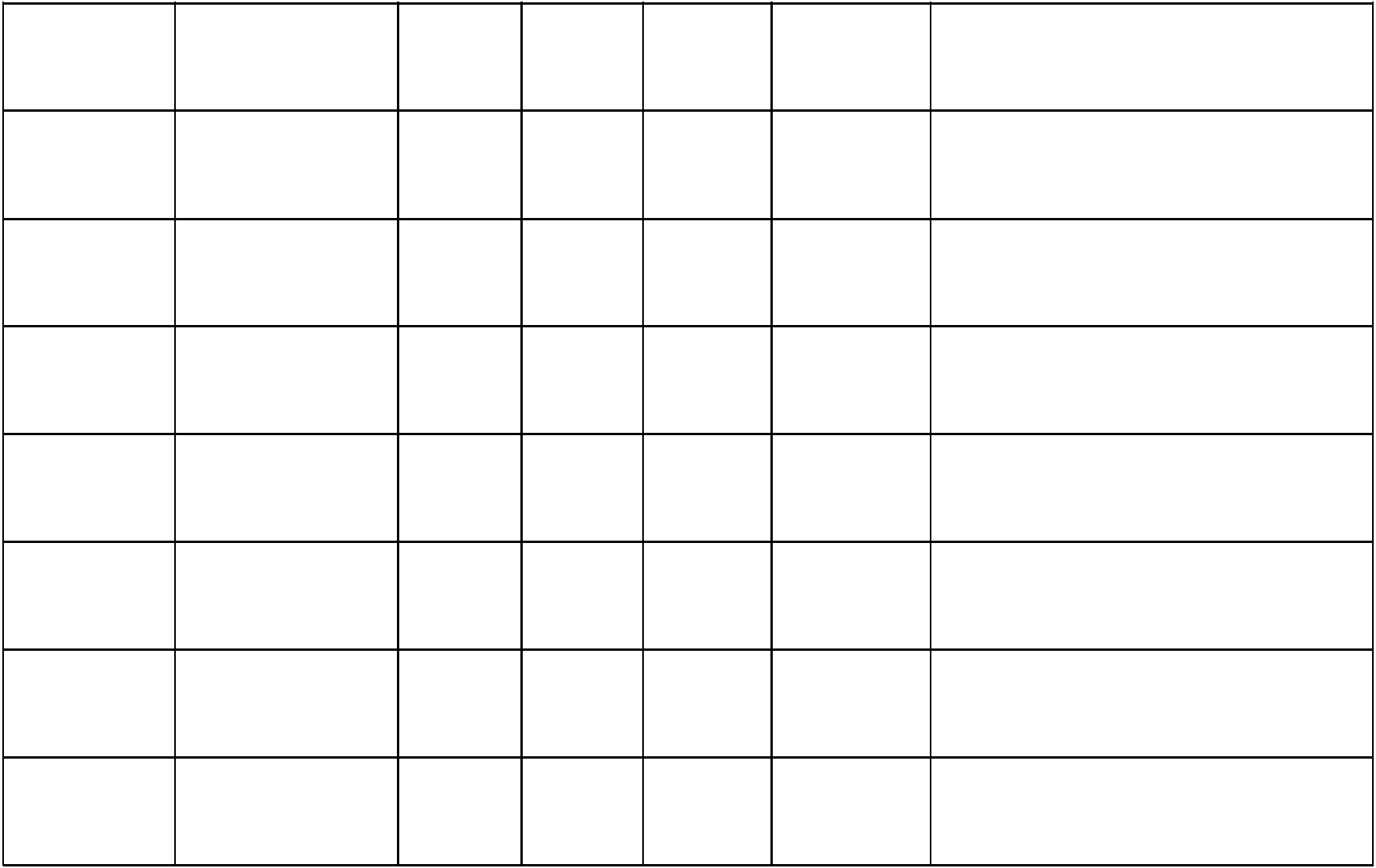


Hangzhou Center for Disease Control and Prevention

Hangzhou Center for Disease Control and Prevention

Hangzhou Center for Disease Control and Prevention

Hangzhou Center for Disease Control and Prevention

Hangzhou Center for Disease Control and Prevention

Hangzhou Center for Disease Control and Prevention

Hangzhou Center for Disease Control and Prevention

Inspection Center of Hangzhou Center for Disease Control and Prevention

Inspection Center of Hanghzou Center for Disease Control and Prevention

Inspection Center of Hangzhou Center for Disease Control and Prevention

Inspection Center of Hangzhou Center for Disease Control and Prevention

Inspection Center of Hangzhou Center for Disease Control and Prevention

Insepction Center of Hangzhou Center for Disease Control and Prevention

Inspection Center of Hangzhou Center for Disease Control and Prevention

Inspection Center of Hangzhou Center for Disease Control and Prevention

Yu hua, Wang haoqiu, Li jun, Yu xinfeng, Pan jingcao

Yu hua, Wang haoqiu, Li jun, Yu xinfeng, Pan jingcao

Yu hua, Wang haoqiu, Li jun, Yu xinfeng, Pan jingcao

Yu hua, Wang haoqiu, Li jun, Yu xinfeng, Pan jingcao

Yu hua, Wang haoqiu, Li jun, Yu xinfeng, Pan jingcao

Yu hua, Wang haoqiu, Li jun, Yu xinfeng, Pan jingcao

Yu hua, Wang haoqiu, Li jun, Yu xinfeng, Pan jingcao

Yu hua, Wang haoqiu, Li jun, Yu xinfeng, Pan jingcao

EPI_ISL_418513

EPI_ISL_418514

EPI_ISL_418515

EPI_ISL_418516

EPI_ISL_418548

EPI_ISL_418580

EPI_ISL_418581

EPI_ISL_418582

EPI_ISL_418583

hCoV-

19/Hangzhou/HZ551/2020

hCoV-

19/Hangzhou/HZ576/2020

hCoV-

19/Hangzhou/HZ638/2020

hCoV-19/Ireland/21023/2020

hCoV-19/Ireland/21145/2020

hCoV-19/Ireland/Dublin-

22361/2020

hCoV-19/Ireland/Dublin-

22428/2020

hCoV-19/Ireland/22901/2020

hCoV-19/Ireland/24042/2020

Asia / China / Hangzhou

Asia / China / Zhejiang / Hangzhou

Asia / China / Hangzhou

Europe / Ireland

/ Tipperary

Europe / Ireland

/ Dublin

Europe / Ireland

/ Cork

Europe / Ireland

/ Dublin

Europe / Ireland

/ Louth

Europe / Ireland

/ Dublin

2020-01-25

2020-01-25

2020-01-25

2020-03-06

2020-03-06

2020-03-08

2020-03-08

2020-03-10

2020-03-10

Hangzhou Center for Disease Control and Prevention


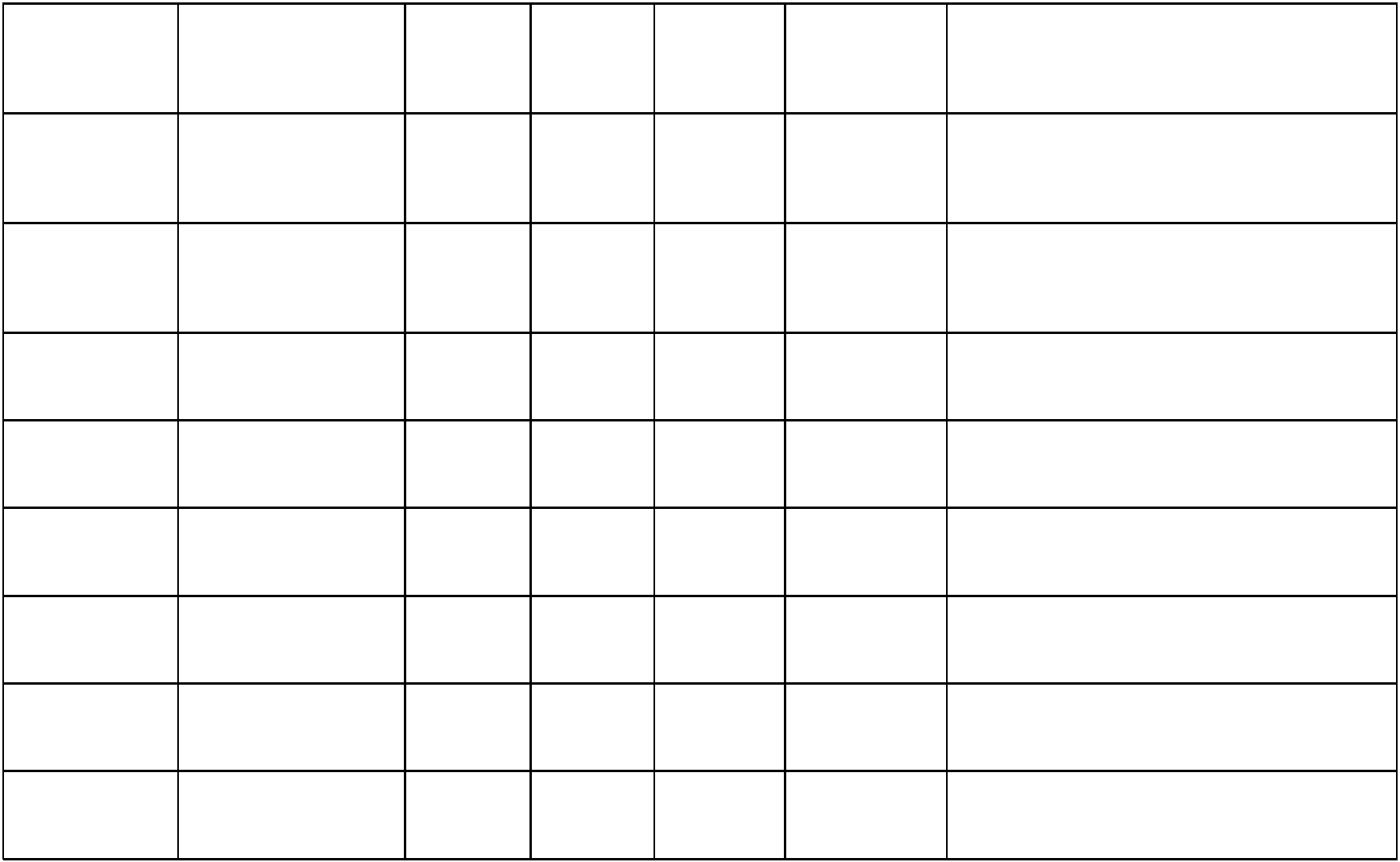


Hangzhou Center for Disease Control and Prevention

Hangzhou Center for Disease Control and Prevention

UCD National Virus Reference Laboratory

UCD National Virus Reference Laboratory

UCD National Virus Reference Laboratory

UCD National Virus Reference Laboratory

UCD National Virus Reference Laboratory

UCD National Virus Reference Laboratory

Inspection Center of Hangzhou Center for Disease Control and Prevention

Inspection Center of Hangzhou Center for Disease Control and Prevention

Inspection Center of Hangzhou Center for Disease Control and Prevention

UCD National Virus Reference Laboratory

UCD National Virus Reference Laboratory

UCD National Virus Reference Laboratory

UCD National Virus Reference Laboratory

UCD National Virus Reference Laboratory

UCD National Virus Reference Laboratory

Yu hua, Wang haoqiu, Li jun, Yu xinfeng, Pan jingcao

Yu hua, Wang haoqiu, Li jun, Yu xinfeng, Pan jingcao

Yu hua, Wang haoqiu, Li jun, Yu xinfeng, Pan jingcao

Michael Carr, Gabriel Gonzalez, Jonathan Dean, Suzie Coughlan, Alison Murphy, Kevin Byrne, Ken Wolfe, Jeff Connell, Brendan Loftus, Cillian F De Gascun

Michael Carr, Gabriel Gonzalez, Jonathan Dean, Suzie Coughlan, Alison Murphy, Kevin Byrne, Ken Wolfe, Jeff Connell, Brendan Loftus, Cillian F De Gascun

Michael Carr, Gabriel Gonzalez, Jonathan Dean, Suzie Coughlan, Alison Murphy, Kevin Byrne, Ken Wolfe, Jeff Connell, Brendan Loftus, Cillian F De Gascun

Michael Carr, Gabriel Gonzalez, Jonathan Dean, Suzie Coughlan, Alison Murphy, Kevin Byrne, Ken Wolfe, Jeff Connell, Brendan Loftus, Cillian F De Gascun

Michael Carr, Gabriel Gonzalez, Jonathan Dean, Suzie Coughlan, Alison Murphy, Kevin Byrne, Ken Wolfe, Jeff Connell, Brendan Loftus, Cillian F De Gascun

Michael Carr, Gabriel Gonzalez, Jonathan Dean, Suzie Coughlan, Alison Murphy, Kevin Byrne, Ken Wolfe, Jeff Connell, Brendan Loftus, Cillian F De Gascun

EPI_ISL_418584

EPI_ISL_419706

EPI_ISL_419708

EPI_ISL_419709

EPI_ISL_419710

EPI_ISL_419711

EPI_ISL_419712

EPI_ISL_419713

EPI_ISL_419733

hCoV-19/Ireland/24052/2020

hCoV-19/USA/VA-DCLS-

0012/2020

hCoV-19/USA/VA-DCLS-

0014/2020

hCoV-

19/Spain/PaisVasco201602/20

20

hCoV-19/USA/VA-DCLS-

0016/2020

hCoV-19/USA/VA-DCLS-

0017/2020

hCoV-19/USA/VA-DCLS-

0020/2020

hCoV-19/USA/VA-DCLS-

0021/2020

hCoV-19/Australia/VIC13/2020

Europe / Ireland

/ Wicklow

North America / USA / Virginia

North America / USA / Virginia

Europe / Spain

/

BasqueCountry

North America / USA / Virginia

North America /USA / Virginia

North America / USA / Virginia

North America / USA / Virginia

Oceania /

Australia /

Victoria

2020-03-10

2020-03-10

2020-03-12

2020-03-04

2020-03-12

2020-03-11

2020-03-10

2020-03-11

2020-01-31

| UCD National |  | UCD National Virus |  |
| --- | --- | --- | --- |
| Virus Reference |  |  |  |
|  |  | Reference Laboratory |  |
| Laboratory |  |  |  |
|  |  |  |  |
| Division of |  | Division of |  |
| Consolidated |  |  |  |
|  |  | Consolidated |  |
| Laboratory |  |  |  |
|  |  | Laboratory Services |  |
| Services |  |  |  |
|  |  |  |  |
|  |  |  |  |
| Division of |  | Division of |  |
| Consolidated |  |  |  |
|  |  | Consolidated |  |
| Laboratory |  |  |  |
|  |  | Laboratory Services |  |
| Services |  |  |  |
|  |  |  |  |
| HOSPITAL |  | Instituto de Salud |  |
| TXAGORRITXU |  | Carlos III |  |
|  |  |  |  |
| Division of |  | Division of |  |
| Consolidated |  |  |  |
|  |  | Consolidated |  |
| Laboratory |  |  |  |
|  |  | Laboratory Services |  |
| Services |  |  |  |
|  |  |  |  |
|  |  |  |  |
| Division of |  | Division of |  |
| Consolidated |  |  |  |
|  |  | Consolidated |  |
| Laboratory |  |  |  |
|  |  | Laboratory Services |  |
| Services |  |  |  |
|  |  |  |  |
| Division of |  | Division of |  |
| Consolidated |  |  |  |
|  |  | Consolidated |  |
| Laboratory |  |  |  |
|  |  | Laboratory Services |  |
| Services |  |  |  |
|  |  |  |  |
|  |  |  |  |
| Division of |  | Division of |  |
| Consolidated |  |  |  |
|  |  | Consolidated |  |
| Laboratory |  |  |  |
|  |  | Laboratory Services |  |
| Services |  |  |  |
|  |  |  |  |
|  |  |  |  |
| Victorian |  | Victorian Infectious |  |
|  |  | Diseases Reference |  |
| Infectious |  | Laboratory and |  |
| Diseases |  | Microbiological |  |
| Reference |  | Diagnostic Unit |  |
| Laboratory |  | Public Health |  |
| (VIDRL) |  | Laboratory, Doherty |  |
|  |  | Institute |  |
|  |  |  |  |


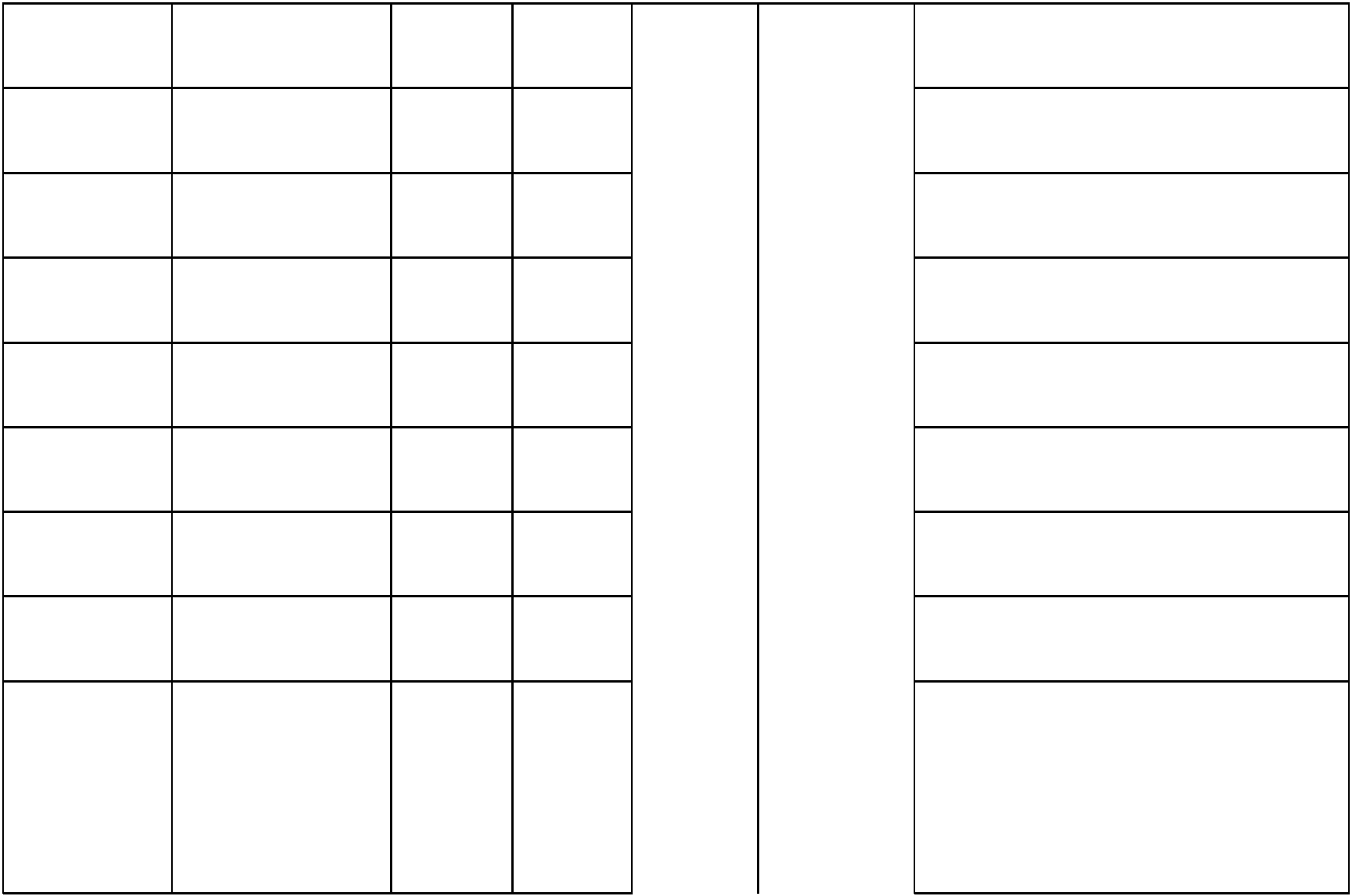


Michael Carr, Gabriel Gonzalez, Jonathan Dean, Suzie Coughlan, Alison Murphy, Kevin Byrne, Ken Wolfe, Jeff Connell, Brendan Loftus, Cillian F De Gascun

Division of Consolidated Laboratory Services

Division of Consolidated Laboratory Services

Iglesias-Caballero, M. Molinero Calamita, M. González-Esguevillas, M. Camarero S. Pozo F. Casas I. Jiménez, P. Jiménez, M. Zaballos, A. Monzón, S. Varona, S. Juliá, M. Cuesta, I. Gómez, C.

Division of Consolidated Laboratory Services

Division of Consolidated Laboratory Services

Division of Consolidated Laboratory Services

Division of Consolidated Laboratory Services

Caly L., Seemann T., Sait, M., Schultz M., Druce J., Sherry, N.

Oceania /

EPI_ISL_419737 hCoV-19/Australia/VIC18/2020 Australia / 2020-03-07

Victoria

Oceania /

EPI_ISL_419740 hCoV-19/Australia/VIC21/2020 Australia / 2020-03-09

Victoria

Oceania /

EPI_ISL_419745 hCoV-19/Australia/VIC26/2020 Australia / 2020-03-10

Victoria

Oceania /

EPI_ISL_419746 hCoV-19/Australia/VIC27/2020 Australia / 2020-03-10

Victoria

|  |  | Victorian Infectious |  |  |
| --- | --- | --- | --- | --- |
| Victorian |  | Diseases Reference |  |  |
| Infectious |  | Laboratory and |  |  |
| Diseases |  | Microbiological | Caly L., Seemann T., Sait, M., Schultz M., Druce J., Sherry, |  |
| Reference |  | Diagnostic Unit | N. |  |
| Laboratory |  | Public Health |  |  |
| (VIDRL) |  | Laboratory, Doherty |  |  |
|  |  | Institute |  |  |
|  |  |  |  |  |
|  |  | Victorian Infectious |  |  |
| Victorian |  | Diseases Reference |  |  |
| Infectious |  | Laboratory and |  |  |
| Diseases |  | Microbiological | Caly L., Seemann T., Sait, M., Schultz M., Druce J., Sherry, |  |
| Reference |  | Diagnostic Unit | N. |  |
| Laboratory |  | Public Health |  |  |
| (VIDRL) |  | Laboratory, Doherty |  |  |
|  |  | Institute |  |  |
|  |  |  |  |  |
|  |  | Victorian Infectious |  |  |
| Victorian |  | Diseases Reference |  |  |
| Infectious |  | Laboratory and |  |  |
| Diseases |  | Microbiological | Caly L., Seemann T., Sait, M., Schultz M., Druce J., Sherry, |  |
| Reference |  | Diagnostic Unit | N. |  |
| Laboratory |  | Public Health |  |  |
| (VIDRL) |  | Laboratory, Doherty |  |  |
|  |  | Institute |  |  |
|  |  |  |  |  |
| Victorian |  | Victorian Infectious |  |  |
|  |  | Diseases Reference |  |  |
| Infectious |  | Laboratory and |  |  |
| Diseases |  | Microbiological | Caly L., Seemann T., Sait, M., Schultz M., Druce J., Sherry, |  |
| Reference |  | Diagnostic Unit | N. |  |
| Laboratory |  | Public Health |  |  |
| (VIDRL) |  | Laboratory, Doherty |  |  |
|  |  | Institute |  |  |
|  |  |  |  |  |


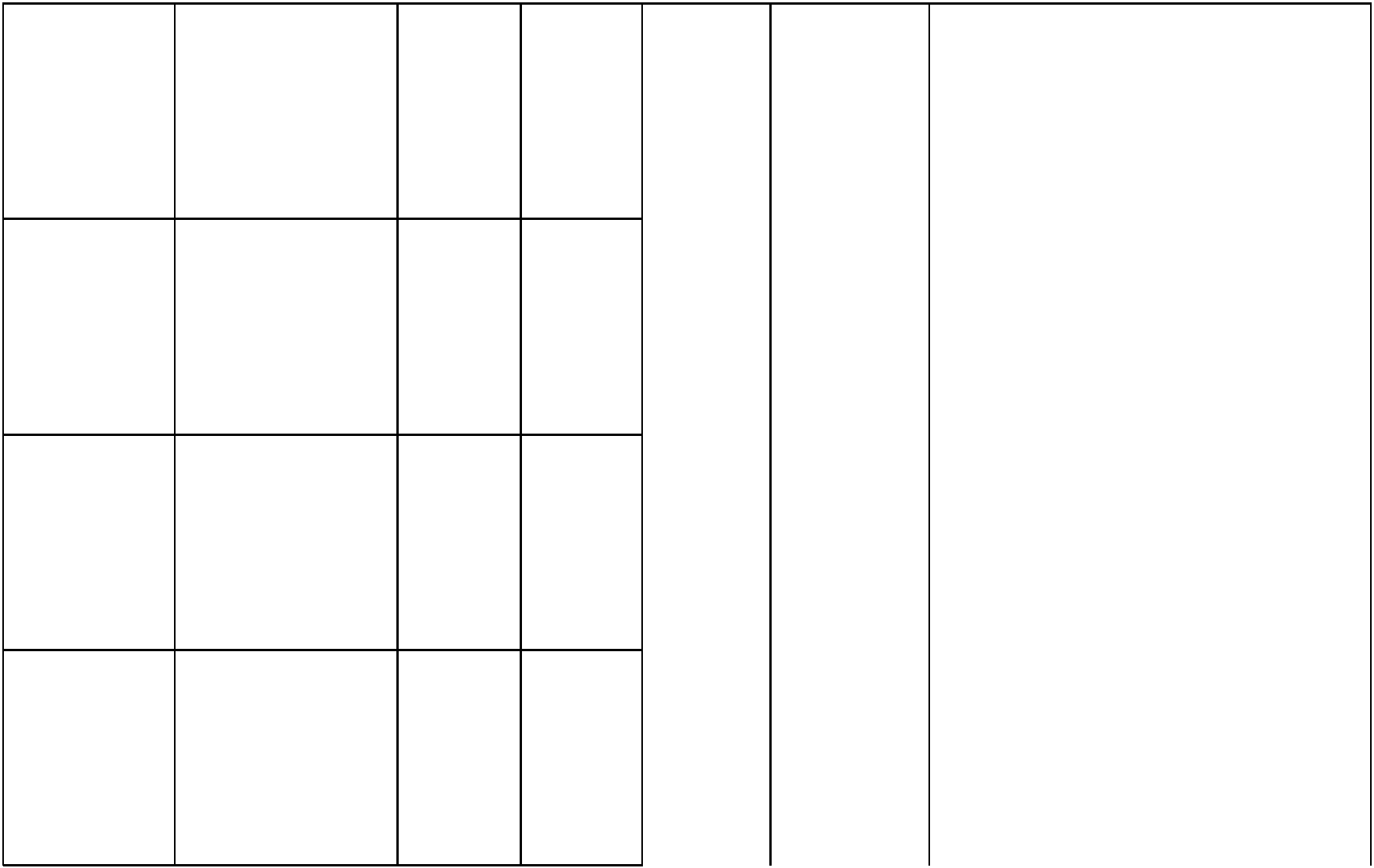


Oceania /

EPI_ISL_419749 hCoV-19/Australia/VIC30/2020 Australia / 2020-03-10

Victoria

Oceania /

EPI_ISL_419750 hCoV-19/Australia/VIC31/2020 Australia / 2020-03-10

Victoria

Oceania /

EPI_ISL_419751 hCoV-19/Australia/VIC32/2020 Australia / 2020-03-10

Victoria

Oceania /

EPI_ISL_419752 hCoV-19/Australia/VIC33/2020 Australia / 2020-03-10

Victoria

|  |  | Victorian Infectious |  |  |
| --- | --- | --- | --- | --- |
| Victorian |  | Diseases Reference |  |  |
| Infectious |  | Laboratory and |  |  |
| Diseases |  | Microbiological | Caly L., Seemann T., Sait, M., Schultz M., Druce J., Sherry, |  |
| Reference |  | Diagnostic Unit | N. |  |
| Laboratory |  | Public Health |  |  |
| (VIDRL) |  | Laboratory, Doherty |  |  |
|  |  | Institute |  |  |
|  |  |  |  |  |
|  |  | Victorian Infectious |  |  |
| Victorian |  | Diseases Reference |  |  |
| Infectious |  | Laboratory and |  |  |
| Diseases |  | Microbiological | Caly L., Seemann T., Sait, M., Schultz M., Druce J., Sherry, |  |
| Reference |  | Diagnostic Unit | N. |  |
| Laboratory |  | Public Health |  |  |
| (VIDRL) |  | Laboratory, Doherty |  |  |
|  |  | Institute |  |  |
|  |  |  |  |  |
|  |  | Victorian Infectious |  |  |
| Victorian |  | Diseases Reference |  |  |
| Infectious |  | Laboratory and |  |  |
| Diseases |  | Microbiological | Caly L., Seemann T., Sait, M., Schultz M., Druce J., Sherry, |  |
| Reference |  | Diagnostic Unit | N. |  |
| Laboratory |  | Public Health |  |  |
| (VIDRL) |  | Laboratory, Doherty |  |  |
|  |  | Institute |  |  |
|  |  |  |  |  |
| Victorian |  | Victorian Infectious |  |  |
|  |  | Diseases Reference |  |  |
| Infectious |  | Laboratory and |  |  |
| Diseases |  | Microbiological | Caly L., Seemann T., Sait, M., Schultz M., Druce J., Sherry, |  |
| Reference |  | Diagnostic Unit | N. |  |
| Laboratory |  | Public Health |  |  |
| (VIDRL) |  | Laboratory, Doherty |  |  |
|  |  | Institute |  |  |
|  |  |  |  |  |


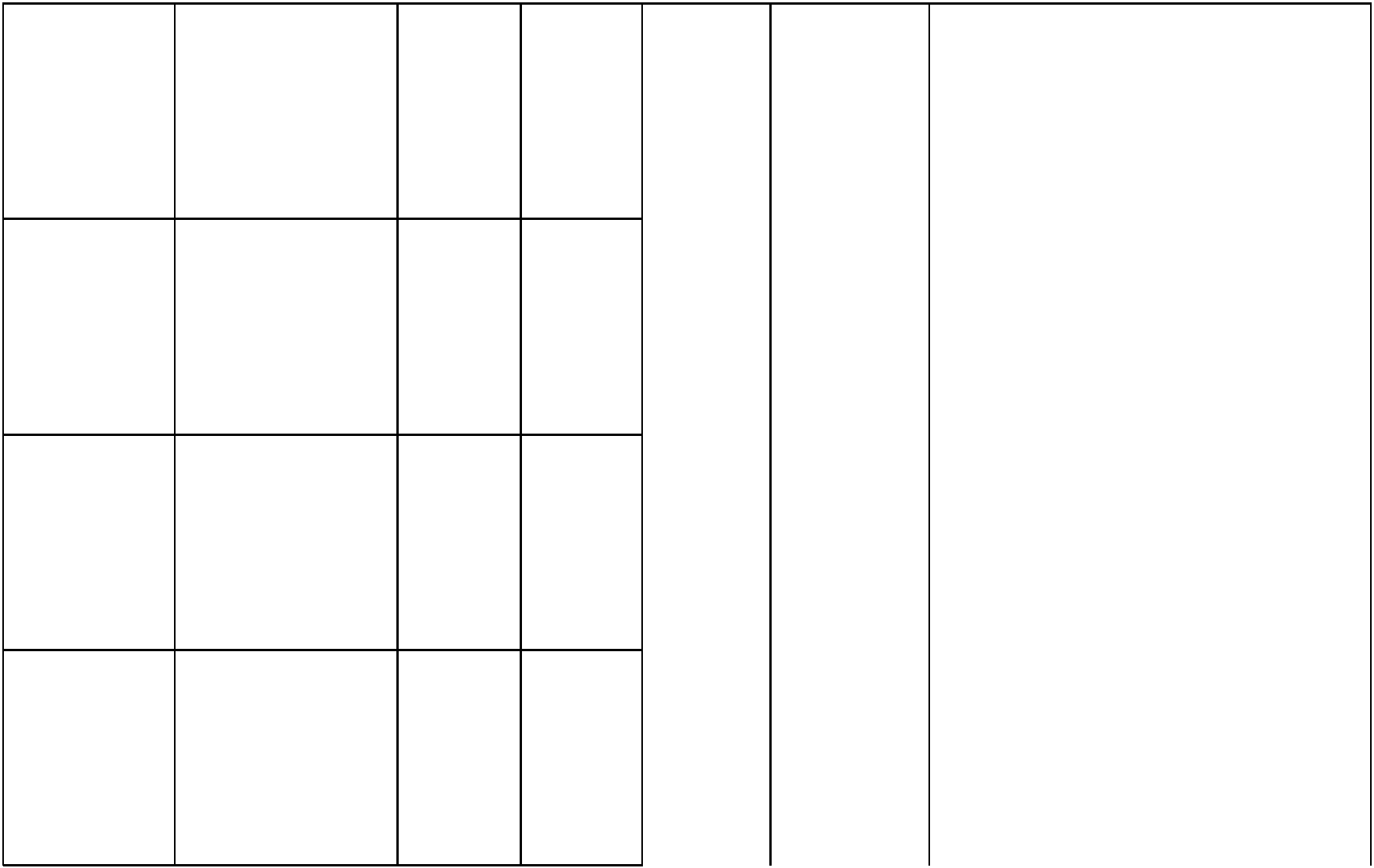


Oceania /

EPI_ISL_419754 hCoV-19/Australia/VIC37/2020 Australia / 2020-03-11

Victoria

Oceania /

EPI_ISL_419756 hCoV-19/Australia/VIC38/2020 Australia / 2020-03-11

Victoria

Oceania /

EPI_ISL_419765 hCoV-19/Australia/VIC36/2020 Australia / 2020-03-10

Victoria

Oceania /

EPI_ISL_419767 hCoV-19/Australia/VIC44/2020 Australia / 2020-03-11

Victoria

|  |  | Victorian Infectious |  |  |
| --- | --- | --- | --- | --- |
| Victorian |  | Diseases Reference |  |  |
| Infectious |  | Laboratory and |  |  |
| Diseases |  | Microbiological | Caly L., Seemann T., Sait, M., Schultz M., Druce J., Sherry, |  |
| Reference |  | Diagnostic Unit | N. |  |
| Laboratory |  | Public Health |  |  |
| (VIDRL) |  | Laboratory, Doherty |  |  |
|  |  | Institute |  |  |
|  |  |  |  |  |
|  |  | Victorian Infectious |  |  |
| Victorian |  | Diseases Reference |  |  |
| Infectious |  | Laboratory and |  |  |
| Diseases |  | Microbiological | Caly L., Seemann T., Sait, M., Schultz M., Druce J., Sherry, |  |
| Reference |  | Diagnostic Unit | N. |  |
| Laboratory |  | Public Health |  |  |
| (VIDRL) |  | Laboratory, Doherty |  |  |
|  |  | Institute |  |  |
|  |  |  |  |  |
|  |  | Victorian Infectious |  |  |
| Victorian |  | Diseases Reference |  |  |
| Infectious |  | Laboratory and |  |  |
| Diseases |  | Microbiological | Caly L., Seemann T., Sait, M., Schultz M., Druce J., Sherry, |  |
| Reference |  | Diagnostic Unit | N. |  |
| Laboratory |  | Public Health |  |  |
| (VIDRL) |  | Laboratory, Doherty |  |  |
|  |  | Institute |  |  |
|  |  |  |  |  |
| Victorian |  | Victorian Infectious |  |  |
|  |  | Diseases Reference |  |  |
| Infectious |  | Laboratory and |  |  |
| Diseases |  | Microbiological | Caly L., Seemann T., Sait, M., Schultz M., Druce J., Sherry, |  |
| Reference |  | Diagnostic Unit | N. |  |
| Laboratory |  | Public Health |  |  |
| (VIDRL) |  | Laboratory, Doherty |  |  |
|  |  | Institute |  |  |
|  |  |  |  |  |


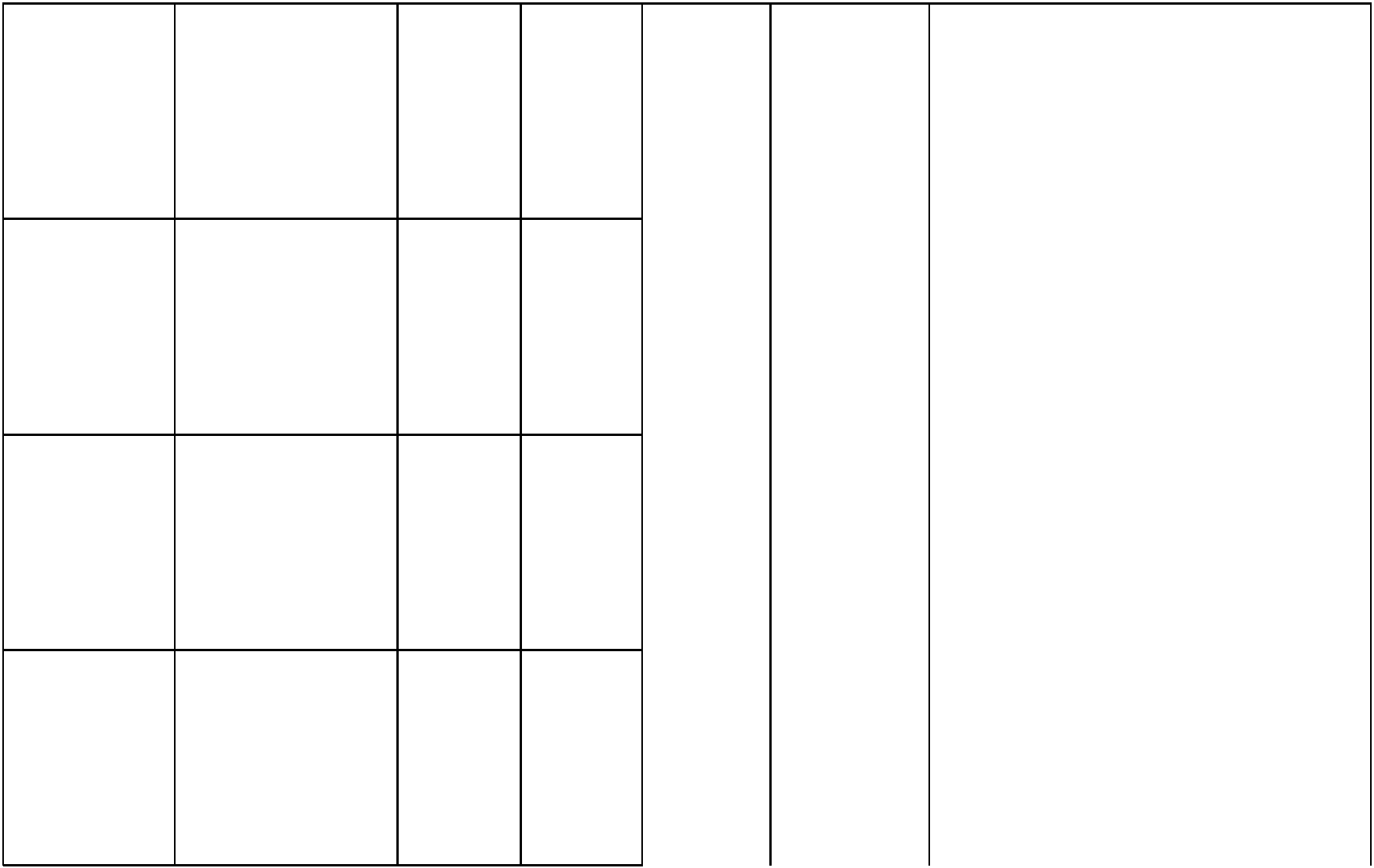


Oceania /

EPI_ISL_419768 hCoV-19/Australia/VIC52/2020 Australia / 2020-03-12

Victoria

Oceania /

EPI_ISL_419770 hCoV-19/Australia/VIC54/2020 Australia / 2020-03-12

Victoria

Oceania /

EPI_ISL_419774 hCoV-19/Australia/VIC62/2020 Australia / 2020-03-13

Victoria

Oceania /

EPI_ISL_419780 hCoV-19/Australia/VIC66/2020 Australia / 2020-03-13

Victoria

|  |  | Victorian Infectious |  |  |
| --- | --- | --- | --- | --- |
| Victorian |  | Diseases Reference |  |  |
| Infectious |  | Laboratory and |  |  |
| Diseases |  | Microbiological | Caly L., Seemann T., Sait, M., Schultz M., Druce J., Sherry, |  |
| Reference |  | Diagnostic Unit | N. |  |
| Laboratory |  | Public Health |  |  |
| (VIDRL) |  | Laboratory, Doherty |  |  |
|  |  | Institute |  |  |
|  |  |  |  |  |
|  |  | Victorian Infectious |  |  |
| Victorian |  | Diseases Reference |  |  |
| Infectious |  | Laboratory and |  |  |
| Diseases |  | Microbiological | Caly L., Seemann T., Sait, M., Schultz M., Druce J., Sherry, |  |
| Reference |  | Diagnostic Unit | N. |  |
| Laboratory |  | Public Health |  |  |
| (VIDRL) |  | Laboratory, Doherty |  |  |
|  |  | Institute |  |  |
|  |  |  |  |  |
|  |  | Victorian Infectious |  |  |
| Victorian |  | Diseases Reference |  |  |
| Infectious |  | Laboratory and |  |  |
| Diseases |  | Microbiological | Caly L., Seemann T., Sait, M., Schultz M., Druce J., Sherry, |  |
| Reference |  | Diagnostic Unit | N. |  |
| Laboratory |  | Public Health |  |  |
| (VIDRL) |  | Laboratory, Doherty |  |  |
|  |  | Institute |  |  |
|  |  |  |  |  |
| Victorian |  | Victorian Infectious |  |  |
|  |  | Diseases Reference |  |  |
| Infectious |  | Laboratory and |  |  |
| Diseases |  | Microbiological | Caly L., Seemann T., Sait, M., Schultz M., Druce J., Sherry, |  |
| Reference |  | Diagnostic Unit | N. |  |
| Laboratory |  | Public Health |  |  |
| (VIDRL) |  | Laboratory, Doherty |  |  |
|  |  | Institute |  |  |
|  |  |  |  |  |


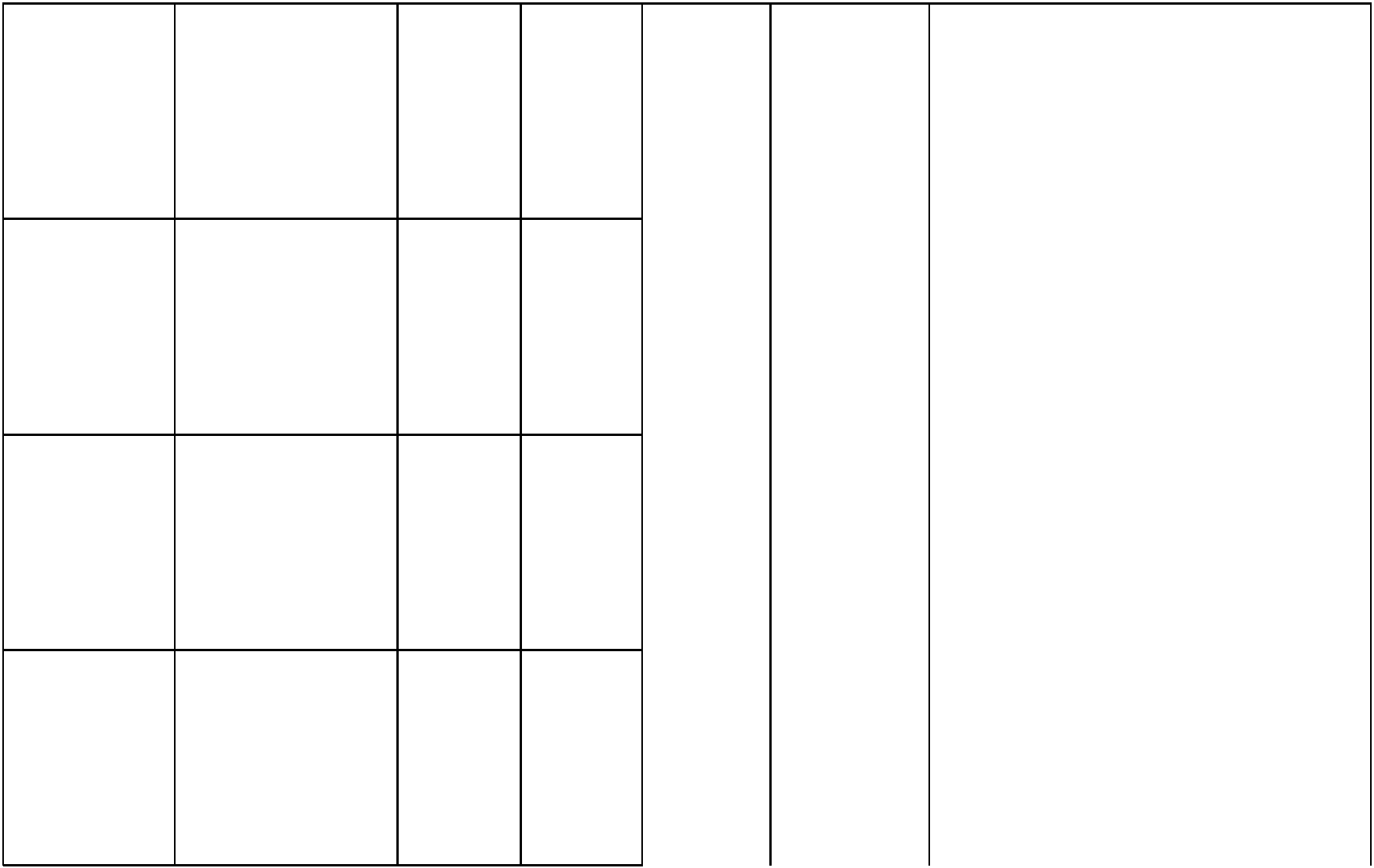


EPI_ISL_419781

EPI_ISL_419787

EPI_ISL_419794

EPI_ISL_419831

hCoV-19/Australia/VIC67/2020

hCoV-19/Australia/VIC75/2020

hCoV-19/Australia/VIC82/2020

hCoV-19/Australia/NT01/2020

Oceania /

Australia /

Victoria

Oceania /

Australia /

Victoria

Oceania /

Australia /

Victoria

Oceania /

Australia /

Northern

Territory

2020-03-13

2020-03-14

2020-03-14

2020-02-21

|  |  | Victorian Infectious |  |
| --- | --- | --- | --- |
| Victorian |  | Diseases Reference |  |
| Infectious |  | Laboratory and |  |
| Diseases |  | Microbiological | Caly L., Seemann T., Sait, M., Schultz M., Druce J., Sherry, |
| Reference |  | Diagnostic Unit | N. |
| Laboratory |  | Public Health |  |
| (VIDRL) |  | Laboratory, Doherty |  |
|  |  | Institute |  |
|  |  |  |  |
|  |  | Victorian Infectious |  |
| Victorian |  | Diseases Reference |  |
| Infectious |  | Laboratory and |  |
| Diseases |  | Microbiological | Caly L., Seemann T., Sait, M., Schultz M., Druce J., Sherry, |
| Reference |  | Diagnostic Unit | N. |
| Laboratory |  | Public Health |  |
| (VIDRL) |  | Laboratory, Doherty |  |
|  |  | Institute |  |
|  |  |  |  |
|  |  | Victorian Infectious |  |
| Victorian |  | Diseases Reference |  |
| Infectious |  | Laboratory and |  |
| Diseases |  | Microbiological | Caly L., Seemann T., Sait, M., Schultz M., Druce J., Sherry, |
| Reference |  | Diagnostic Unit | N. |
| Laboratory |  | Public Health |  |
| (VIDRL) |  | Laboratory, Doherty |  |
|  |  | Institute |  |
|  |  |  |  |
|  |  | Victorian Infectious |  |
|  |  | Diseases Reference |  |
|  |  | Laboratory and |  |
| Royal Darwin |  | Microbiological | Meumann, E., Seemann T., Sait, M., Schultz M., Caly L., |
| Hospital |  | Diagnostic Unit | Druce J. |
|  |  | Public Health |  |
|  |  | Laboratory, Doherty |  |
|  |  | Institute |  |
|  |  |  |  |


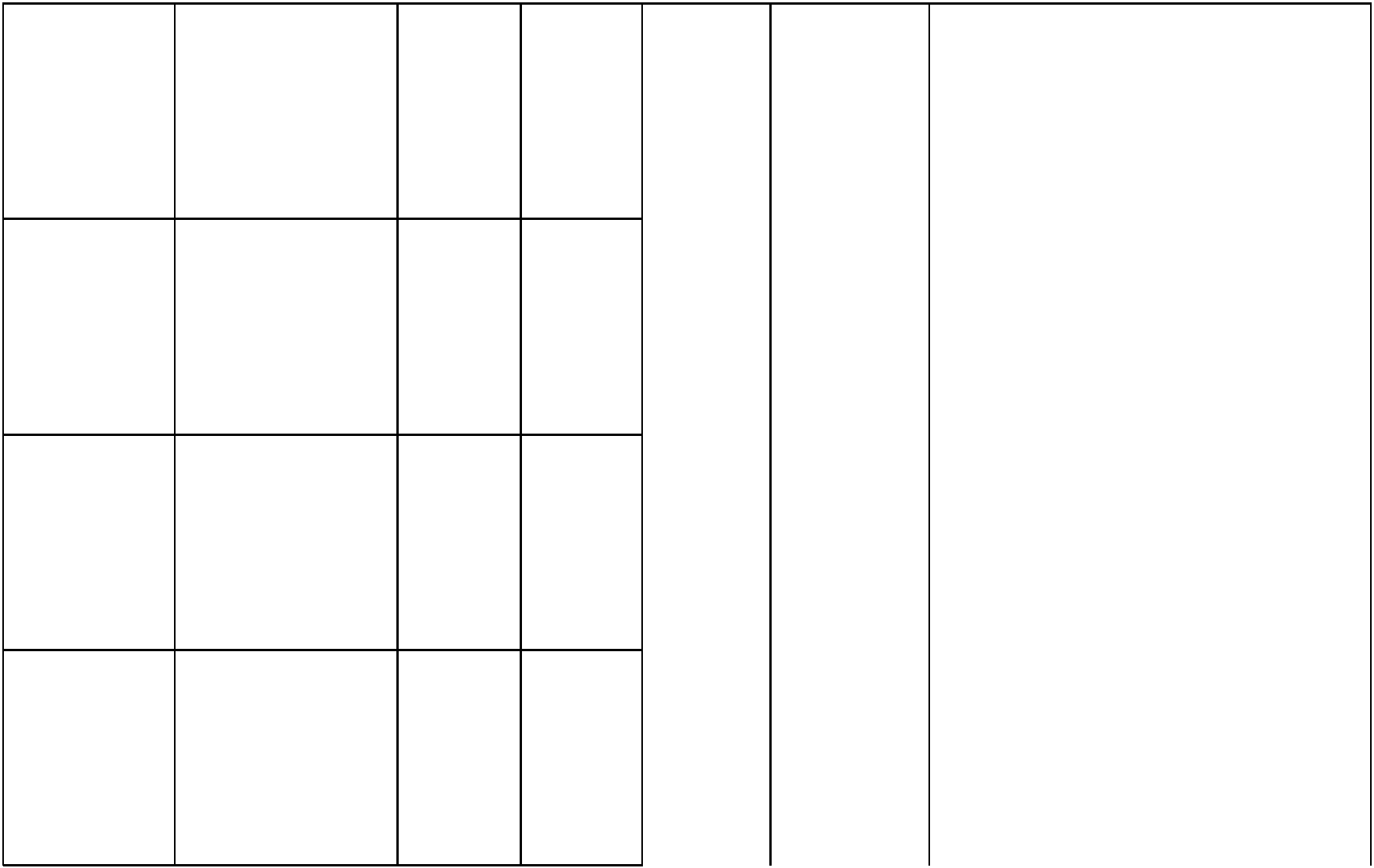


Oceania /

EPI_ISL_419832 hCoV-19/Australia/NT02/2020 ^Australia /^

Northern

Territory

|  |  | Victorian Infectious |  |  |
| --- | --- | --- | --- | --- |
|  |  | Diseases Reference |  |  |
|  |  | Laboratory and |  |  |
| 2020-02-21 | Royal Darwin | Microbiological | Meumann, E., Seemann T., Sait, M., Schultz M., Caly L., |  |
|  | Hospital | Diagnostic Unit | Druce J. |  |
|  |  |  |  |  |


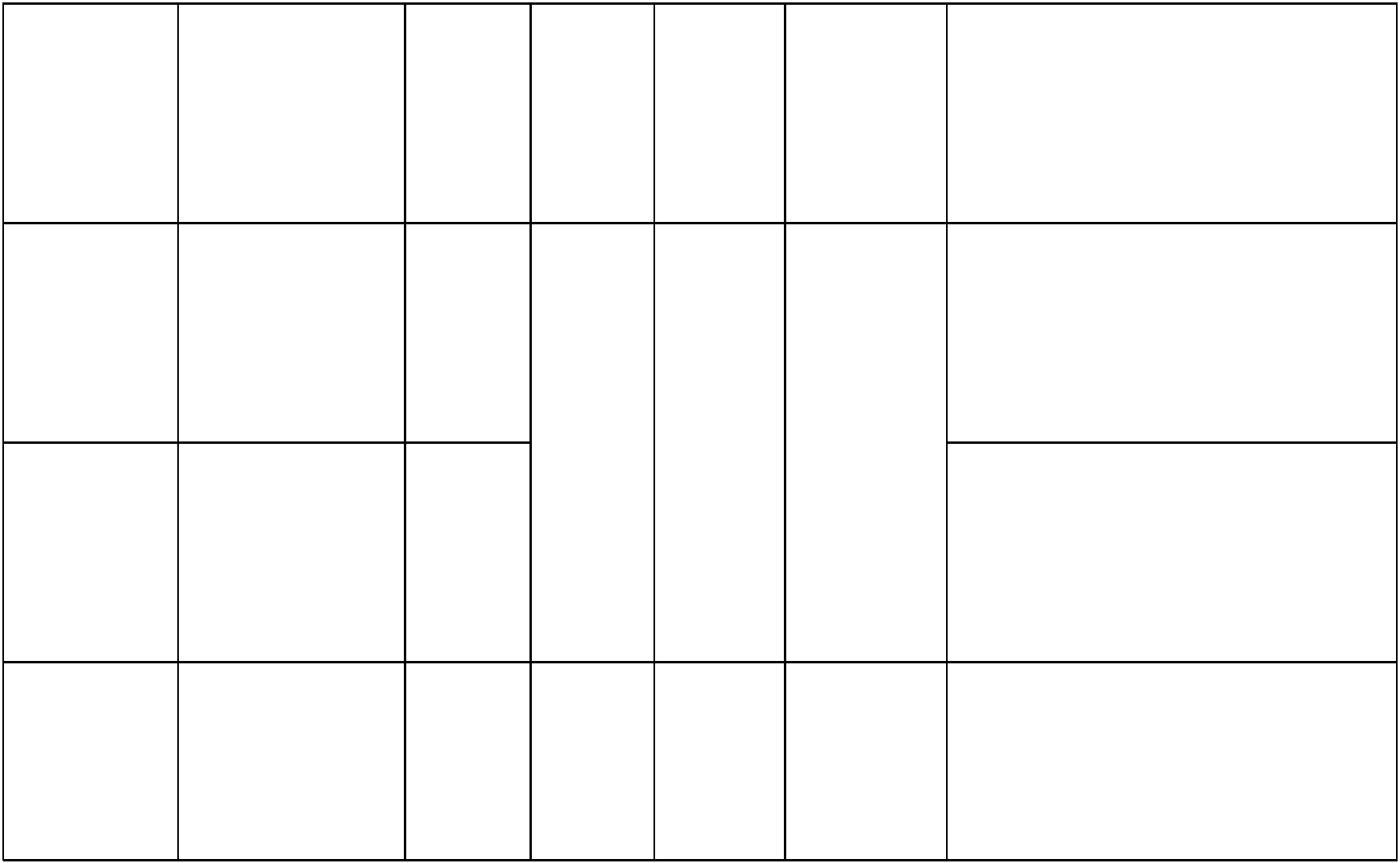


Public Health

Laboratory, Doherty

Institute

|  | hCoV- | Oceania / |  |
| --- | --- | --- | --- |
| EPI_ISL_419834 |  | Australia / |  |
|  | 19/Australia/VIC138/2020 |  |  |
|  |  | Victoria |  |
|  |  |  |  |

Oceania /

EPI_ISL_419835 hCoV-19/Australia/NT05/2020 ^Australia /^

Northern

Territory

|  |  |  | Victorian Infectious |  |
| --- | --- | --- | --- | --- |
|  | Victorian |  | Diseases Reference |  |
|  | Infectious |  | Laboratory and |  |
| 2020-02-23 | Diseases |  | Microbiological |  |
|  | Reference |  | Diagnostic Unit |  |
|  |  |  |  |  |
|  | Laboratory |  | Public Health |  |
|  | (VIDRL) |  | Laboratory, Doherty |  |
|  |  |  | Institute |  |
|  |  |  |  |  |
|  |  |  | Victorian Infectious |  |
|  |  |  | Diseases Reference |  |
|  |  |  | Laboratory and |  |
| 2020-02-24 | Royal Darwin |  | Microbiological |  |
|  | Hospital |  | Diagnostic Unit |  |
|  |  |  |  |  |

Public Health

Laboratory, Doherty

Institute

Caly L., Seemann T., Sait, M., Schultz M., Druce J., Sherry, N.

Meumann, E., Seemann T., Sait, M., Schultz M., Caly L.,

Druce J.

|  |  |  |  |  | Pathogen Discovery, |  |  |
| --- | --- | --- | --- | --- | --- | --- | --- |
|  |  |  |  |  | Respiratory Viruses | Krista Queen, Yan Li, Ying Tao, Jing Zhang, Anne Uehara, |  |
|  |  |  |  | AZ Department | Branch, Division of |  |  |
| EPI_ISL_420784 | hCoV-19/USA/AZ_4811/2020 | North America / | 2020-03-02 |  |  | Clinton R. Paden, Haibin Wang, Rachel Marine, Mary S. |  |
|  |  |  |  | of Health | Viral Diseases, |  |  |
|  |  | USA / Arizona |  | Services | Centers for Disease | Keckler, Alison S. Laufer Halpin, Jasmine Padilla, Justin Lee, |  |
|  |  |  |  |  |  | Christopher A. Elkins, Suxiang Tong |  |
|  |  |  |  |  | Control and |  |  |
|  |  |  |  |  |  |  |  |

Prevention

EPI_ISL_420785

EPI_ISL_420786

EPI_ISL_420787

EPI_ISL_420788

hCoV-19/USA/FL_6318/2020

hCoV-19/USA/GA_1320/2020

hCoV-19/USA/GA_1299/2020

hCoV-19/USA/GA_1445/2020

North America / USA / Florida

North America / USA / Georgia

North America / USA / Georgia

North America / USA / Georgia

2020-03-02

2020-03-03

2020-03-03

2020-03-04

FL Bureau of


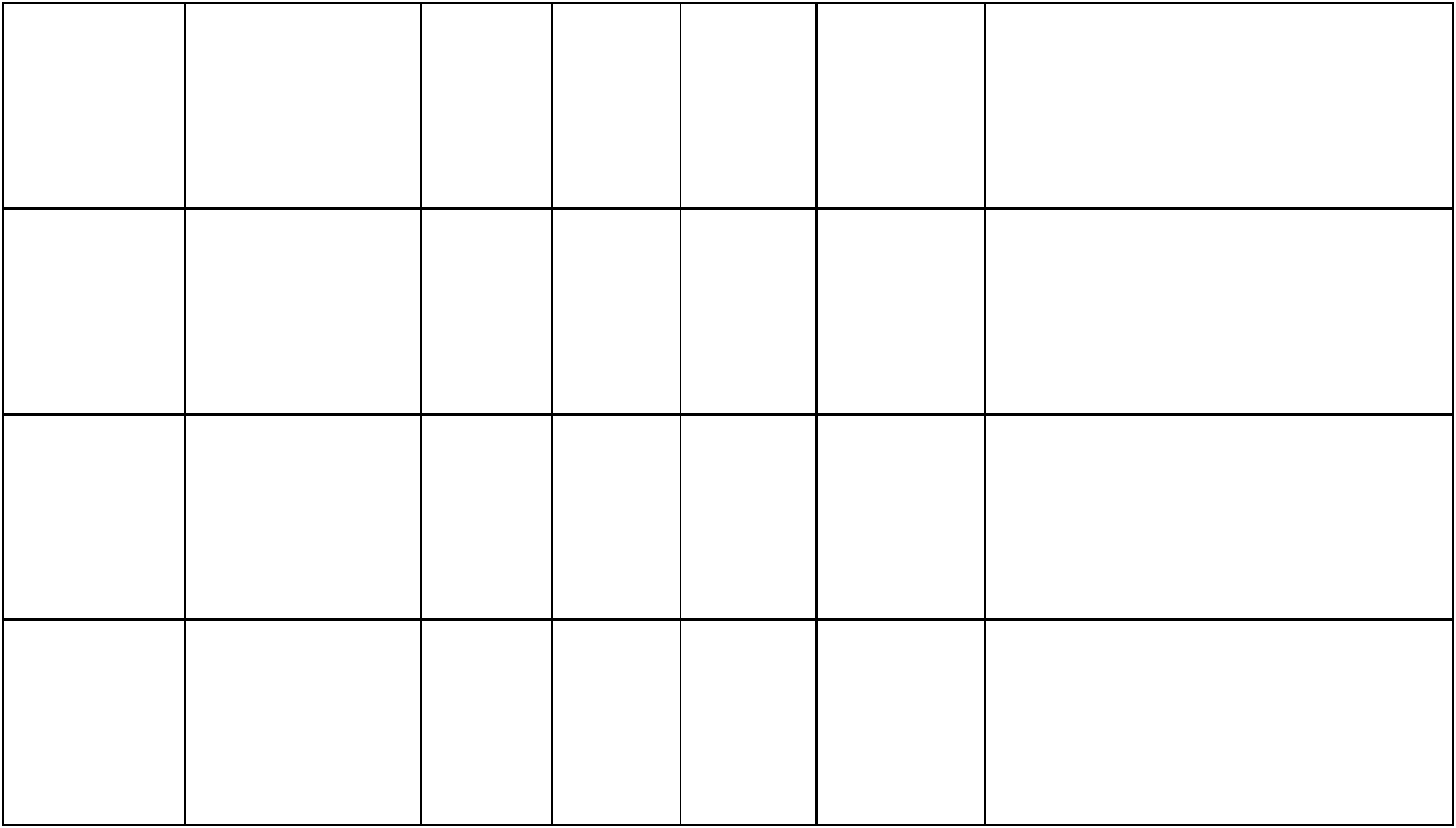


Health

Laboratories

Tampa

GA Department of Public Health

GA Department of Public Health

GA Department of Public Health

Pathogen Discovery, Respiratory Viruses Branch, Division of Viral Diseases, Centers for Disease Control and Prevention

Pathogen Discovery, Respiratory Viruses Branch, Division of Viral Diseases, Centers for Disease Control and Prevention

Pathogen Discovery, Respiratory Viruses Branch, Division of Viral Diseases, Centers for Disease Control and Prevention

Pathogen Discovery, Respiratory Viruses Branch, Division of Viral Diseases, Centers for Disease Control and Prevention

Krista Queen, Yan Li, Ying Tao, Jing Zhang, Anne Uehara, Clinton R. Paden, Haibin Wang, Rachel Marine, Mary S.

Keckler, Alison S. Laufer Halpin, Jasmine Padilla, Justin Lee, Christopher A. Elkins, Suxiang Tong

Krista Queen, Yan Li, Ying Tao, Jing Zhang, Anne Uehara, Clinton R. Paden, Haibin Wang, Rachel Marine, Mary S.

Keckler, Alison S. Laufer Halpin, Jasmine Padilla, Justin Lee, Christopher A. Elkins, Suxiang Tong

Krista Queen, Yan Li, Ying Tao, Jing Zhang, Anne Uehara, Clinton R. Paden, Haibin Wang, Rachel Marine, Mary S.

Keckler, Alison S. Laufer Halpin, Jasmine Padilla, Justin Lee, Christopher A. Elkins, Suxiang Tong

Krista Queen, Yan Li, Ying Tao, Jing Zhang, Anne Uehara, Clinton R. Paden, Haibin Wang, Rachel Marine, Mary S.

Keckler, Alison S. Laufer Halpin, Jasmine Padilla, Justin Lee, Christopher A. Elkins, Suxiang Tong

EPI_ISL_420789

EPI_ISL_420790

EPI_ISL_420791

EPI_ISL_420792

hCoV-19/USA/IL_1375/2020

hCoV-19/USA/IL_1293/2020

hCoV-19/USA/NH_0004/2020

hCoV-19/USA/NH_0008/2020

North America / USA / Illinois

North America / USA / Illinois

North America / USA / New Hampshire

North America / USA / New Hampshire

|  |  |  | Pathogen Discovery, |  |
| --- | --- | --- | --- | --- |
|  | Illinois |  | Respiratory Viruses |  |
|  | Department of |  | Branch, Division of |  |
| 2020-03-01 | Public Health |  | Viral Diseases, |  |
|  | Chicago |  | Centers for Disease |  |
|  | Laboratory |  | Control and |  |
|  |  |  | Prevention |  |
|  |  |  |  |  |
|  | Illinois |  | Pathogen Discovery, |  |
|  |  |  | Respiratory Viruses |  |
| 2020-03-01 | Department of |  | Branch, Division of |  |
|  | Public Health |  | Viral Diseases, |  |
|  | Chicago |  | Centers for Disease |  |
|  | Laboratory |  | Control and |  |
|  |  |  | Prevention |  |
|  |  |  |  |  |
|  |  |  | Pathogen Discovery, |  |
|  | NH Department |  | Respiratory Viruses |  |
|  | of Health and |  | Branch, Division of |  |
| 2020-02-29 | Human Services |  | Viral Diseases, |  |
|  | Public Health |  | Centers for Disease |  |
|  | Labs |  | Control and |  |
|  |  |  | Prevention |  |
|  |  |  |  |  |
|  | NH Department |  | Pathogen Discovery, |  |
|  |  |  | Respiratory Viruses |  |
| 2020-03-02 | of Health and |  | Branch, Division of |  |
|  | Human Services |  | Viral Diseases, |  |
|  | Public Health |  | Centers for Disease |  |
|  | Labs |  | Control and |  |
|  |  |  | Prevention |  |
|  |  |  |  |  |


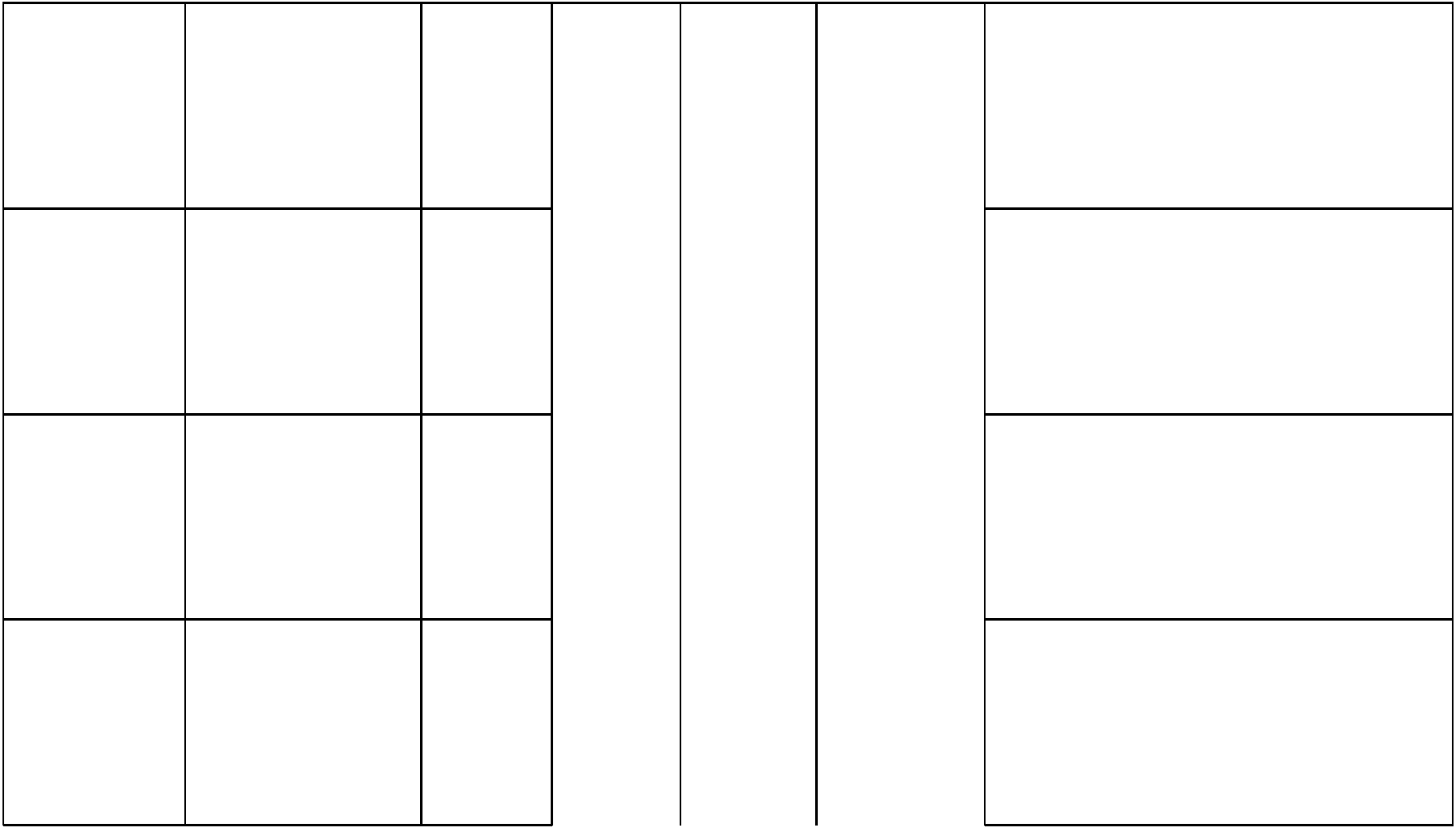


Krista Queen, Yan Li, Ying Tao, Jing Zhang, Anne Uehara, Clinton R. Paden, Haibin Wang, Rachel Marine, Mary S.

Keckler, Alison S. Laufer Halpin, Jasmine Padilla, Justin Lee, Christopher A. Elkins, Suxiang Tong

Krista Queen, Yan Li, Ying Tao, Jing Zhang, Anne Uehara, Clinton R. Paden, Haibin Wang, Rachel Marine, Mary S.

Keckler, Alison S. Laufer Halpin, Jasmine Padilla, Justin Lee, Christopher A. Elkins, Suxiang Tong

Krista Queen, Yan Li, Ying Tao, Jing Zhang, Anne Uehara, Clinton R. Paden, Haibin Wang, Rachel Marine, Mary S.

Keckler, Alison S. Laufer Halpin, Jasmine Padilla, Justin Lee, Christopher A. Elkins, Suxiang Tong

Krista Queen, Yan Li, Ying Tao, Jing Zhang, Anne Uehara, Clinton R. Paden, Haibin Wang, Rachel Marine, Mary S.

Keckler, Alison S. Laufer Halpin, Jasmine Padilla, Justin Lee, Christopher A. Elkins, Suxiang Tong


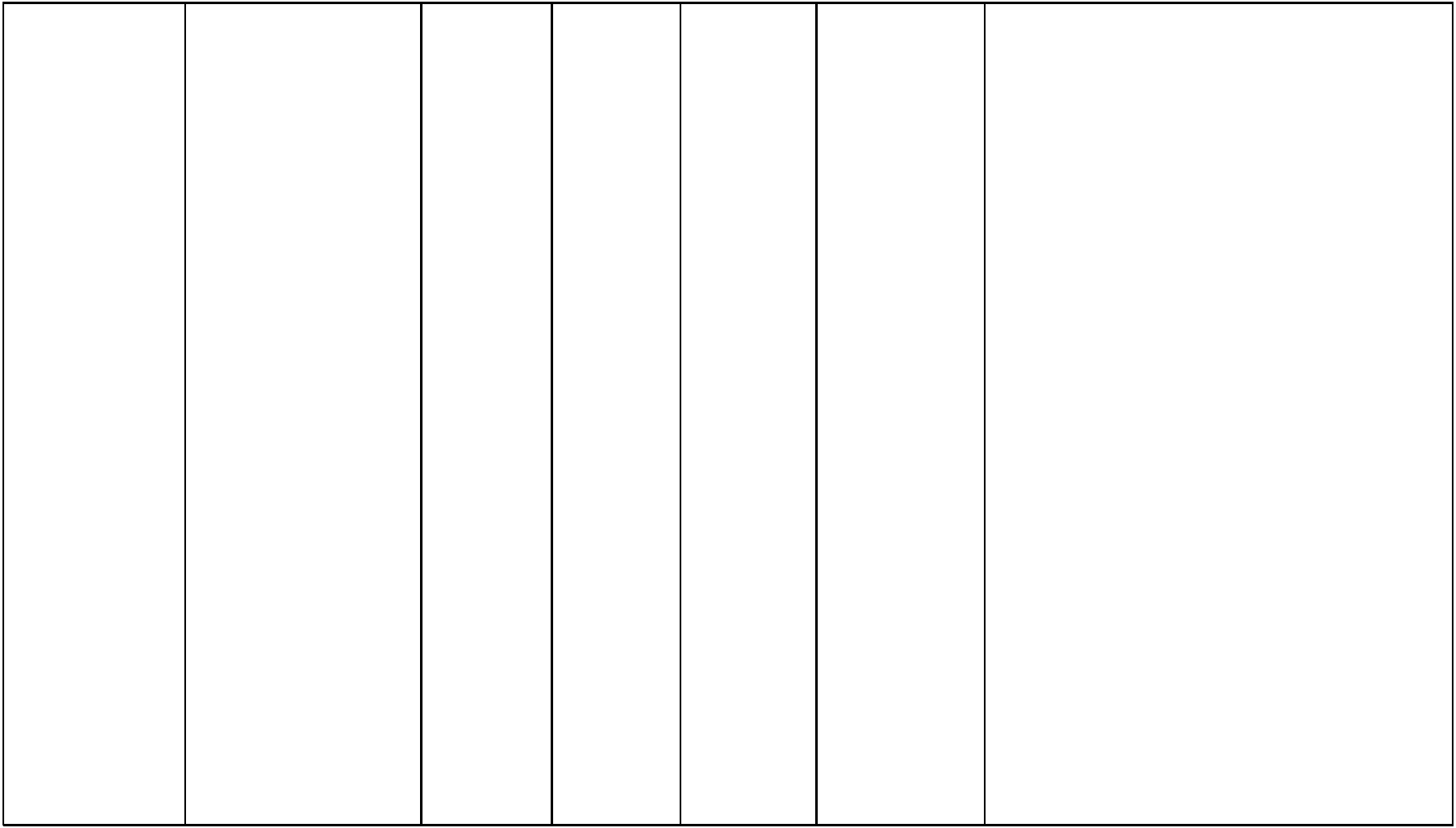


|  |  |  |  |  |  |  |  |  |  | Pathogen Discovery, |  |  |
| --- | --- | --- | --- | --- | --- | --- | --- | --- | --- | --- | --- | --- |
|  |  |  |  |  |  |  | Oregon | State |  | Respiratory Viruses | Krista Queen, Yan Li, Ying Tao, Jing Zhang, Anne Uehara, |  |
|  |  |  |  |  |  |  |  |  |  | Branch, Division of |  |  |
|  |  |  |  | North America / |  |  |  |  |  |  | Clinton R. Paden, Haibin Wang, Rachel Marine, Mary S. |  |
| EPI_ISL_420794 |  | hCoV-19/USA/OR_5430/2020 |  |  | 2020-03-01 | | Public | Health- |  | Viral Diseases, |  |  |
|  |  |  |  | USA / Oregon |  |  | Virology section | |  | Centers for Disease | Keckler, Alison S. Laufer Halpin, Jasmine Padilla, Justin Lee, |  |
|  |  |  |  |  |  |  |  |  |  |  | Christopher A. Elkins, Suxiang Tong |  |
|  |  |  |  |  |  |  |  |  |  | Control and |  |  |
|  |  |  |  |  |  |  |  |  |  |  |  |  |
|  |  |  |  |  |  |  |  |  |  | Prevention |  |  |
|  |  |  |  |  |  |  |  |  |  |  |  |  |
|  |  |  |  |  |  |  |  |  |  | Pathogen Discovery, |  |  |
|  |  |  |  |  |  |  |  |  |  | Respiratory Viruses | Krista Queen, Yan Li, Ying Tao, Jing Zhang, Anne Uehara, |  |
|  |  |  |  | North America / |  |  |  |  |  | Branch, Division of |  |  |
| EPI_ISL_420795 |  | hCoV-19/USA/RI_0556/2020 |  |  | 2020-03-01 | | RI State Health | |  |  | Clinton R. Paden, Haibin Wang, Rachel Marine, Mary S. |  |
|  |  |  |  | USA / Rhode |  |  |  |  |  | Viral Diseases, |  |  |
|  |  |  |  |  |  |  | Laboratory | |  |  | Keckler, Alison S. Laufer Halpin, Jasmine Padilla, Justin Lee, |  |
|  |  |  |  | Island |  |  |  |  |  | Centers for Disease |  |  |
|  |  |  |  |  |  |  |  |  |  |  | Christopher A. Elkins, Suxiang Tong |  |
|  |  |  |  |  |  |  |  |  |  | Control and |  |  |
|  |  |  |  |  |  |  |  |  |  |  |  |  |
|  |  |  |  |  |  |  |  |  |  | Prevention |  |  |
|  |  |  |  |  |  |  |  |  |  |  |  |  |
|  |  |  |  |  |  |  |  |  |  | Pathogen Discovery, |  |  |
|  |  |  |  |  |  |  |  |  |  | Respiratory Viruses | Krista Queen, Yan Li, Ying Tao, Jing Zhang, Anne Uehara, |  |
|  |  |  |  |  |  |  |  |  |  | Branch, Division of |  |  |
|  |  |  |  | North America / |  |  | Texas DSHS | |  |  | Clinton R. Paden, Haibin Wang, Rachel Marine, Mary S. |  |
| EPI_ISL_420796 |  | hCoV-19/USA/TX_2039/2020 |  |  | 2020-02-29 | |  |  |  | Viral Diseases, |  |  |
|  |  |  |  | USA / Texas |  |  | Lab Services | |  | Centers for Disease | Keckler, Alison S. Laufer Halpin, Jasmine Padilla, Justin Lee, |  |
|  |  |  |  |  |  |  |  |  |  |  | Christopher A. Elkins, Suxiang Tong |  |
|  |  |  |  |  |  |  |  |  |  | Control and |  |  |
|  |  |  |  |  |  |  |  |  |  |  |  |  |
|  |  |  |  |  |  |  |  |  |  | Prevention |  |  |
|  |  |  |  |  |  |  |  |  |  |  |  |  |
|  |  |  |  |  |  |  |  |  |  | Pathogen Discovery, |  |  |
|  |  |  |  |  |  |  |  |  |  | Respiratory Viruses | Krista Queen, Yan Li, Ying Tao, Jing Zhang, Anne Uehara, |  |
|  |  |  |  |  |  |  |  |  |  | Branch, Division of |  |  |
| EPI_ISL_420797 |  | hCoV-19/USA/TX_2817/2020 |  | North America / | 2020-03-01 | | Texas DSHS | |  |  | Clinton R. Paden, Haibin Wang, Rachel Marine, Mary S. |  |
|  |  |  |  |  |  |  |  |  |  | Viral Diseases, |  |  |
|  |  |  |  | USA / Texas |  |  | Lab Services | |  | Centers for Disease | Keckler, Alison S. Laufer Halpin, Jasmine Padilla, Justin Lee, |  |
|  |  |  |  |  |  |  |  |  |  |  | Christopher A. Elkins, Suxiang Tong |  |
|  |  |  |  |  |  |  |  |  |  | Control and |  |  |
|  |  |  |  |  |  |  |  |  |  |  |  |  |

Prevention

EPI_ISL_420798

EPI_ISL_420799

EPI_ISL_420889

EPI_ISL_420899

EPI_ISL_421171

EPI_ISL_421172

EPI_ISL_421173

hCoV-19/USA/TX_2967/2020

hCoV-19/Korea/BA-

ACH_2604/2020

hCoV-

19/Japan/Hu_DP_Kng_19-

031/2020

hCoV-19/Germany/BAV-

V2010492/2020

hCoV-

19/Spain/Madrid_H12_1301/2

020

hCoV-

19/Spain/Madrid_H12_1502/2

020

hCoV-

19/Spain/Madrid_H12_1703/2

020

North America / USA / Texas

Asia / Korea

Asia / Japan

Europe /

Germany /

Munich

Europe / Spain/

Madrid

Europe / Spain/

Madrid

Europe / Spain

/ Madrid

2020-03-01

2020-02-27

2020-02-14

2020-03-11

2020-03-05

2020-03-09

2020-03-08

Texas DSHS Lab Services


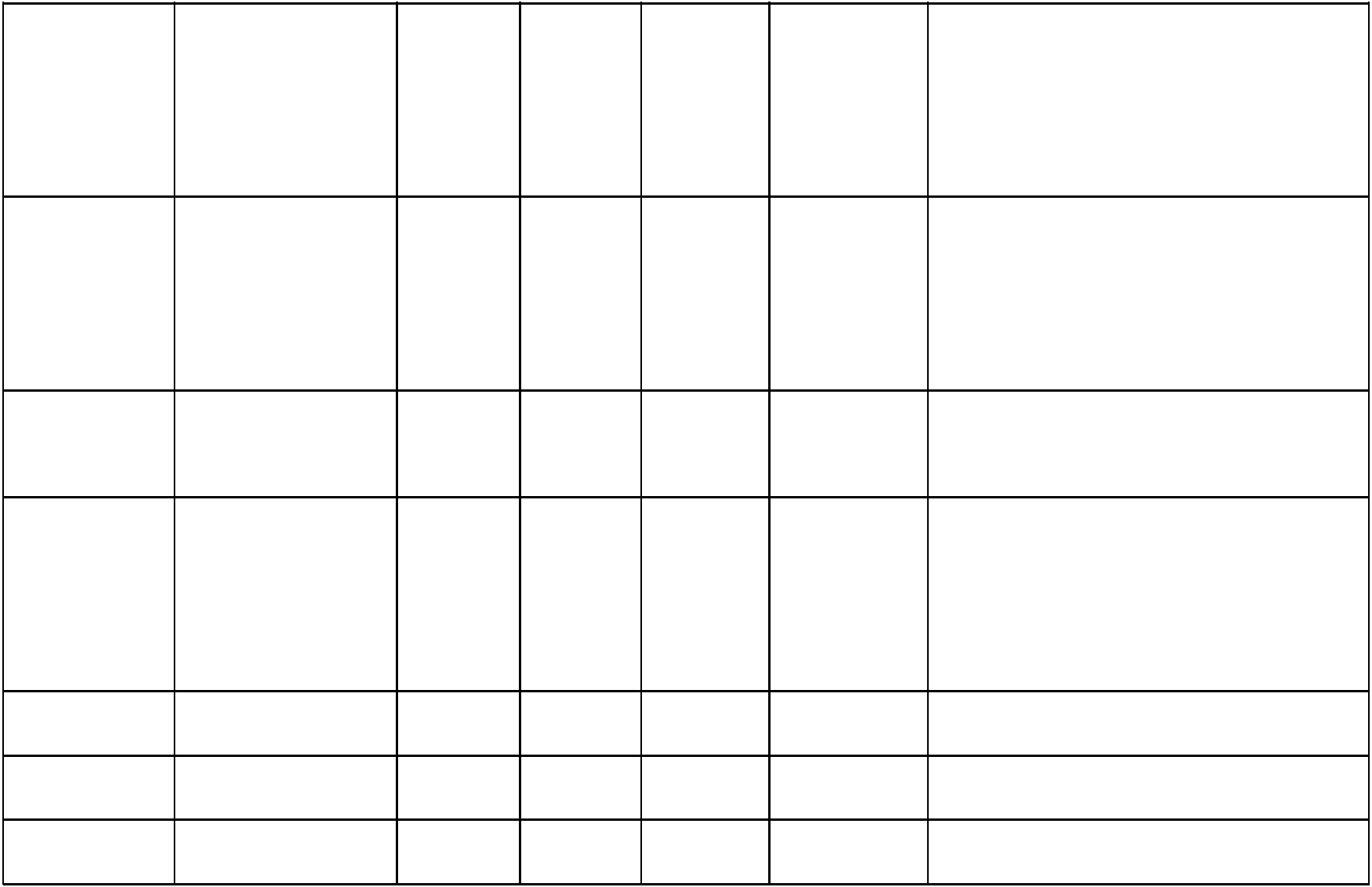


Brian D. Allgood

Army

Community

Hospital

unknown

Max von Pettenkofer Institute, Virology, National

Reference Center for Retroviruses, LMU Munich

Hospital Universitario 12 de Octubre

Hospital Universitario 12 de Octubre

Hospital Universitario 12 de Octubre

Pathogen Discovery, Respiratory Viruses Branch, Division of Viral Diseases, Centers for Disease Control and Prevention

Pathogen Discovery, Respiratory Viruses Branch, Division of Viral Diseases, Centers for Disease Control and Prevention

Takayuki Hishiki Kanagawa Prefectural Institute of Public Health

Laboratory for Functional Genome Analysis, Dept.

Genomics, Gene Center of the LMU Munich

Hospital

Universitario 12 de

Octubre

Hospital

Universitario 12 de

Octubre

Hospital

Universitario 12 de

Octubre

Krista Queen, Yan Li, Ying Tao, Jing Zhang, Anne Uehara, Clinton R. Paden, Haibin Wang, Rachel Marine, Mary S.

Keckler, Alison S. Laufer Halpin, Jasmine Padilla, Justin Lee, Christopher A. Elkins, Suxiang Tong

Krista Queen, Yan Li, Ying Tao, Jing Zhang, Anne Uehara, Clinton R. Paden, Haibin Wang, Rachel Marine, Mary S.

Keckler, Alison S. Laufer Halpin, Jasmine Padilla, Justin Lee, Christopher A. Elkins, Suxiang Tong

Hishiki,T., Suzuki,R., Sakuragi,J., Usui,K., Tanaka,Y., Kawai,J., Kogo,Y., Matsuki,Y., An,T., Hayashizaki,Y. and Takasaki,T.

Max Muenchhoff, Stefan Krebs, Alexander Graf, Ashok Varadharajan, Oliver Keppler, Helmut Blum

Esther Viedma, Sara González, Elias Dahdouh, Raúl Recio, Fernando Lázaro, Julio García, Mª Dolores Folgueira, Jesús Mingorance, Rafael Delgado

Esther Viedma, Sara González, Elias Dahdouh, Raúl Recio, Fernando Lázaro, Julio García, Mª Dolores Folgueira, Jesús Mingorance, Rafael Delgado

Esther Viedma, Sara González, Elias Dahdouh, Raúl Recio, Fernando Lázaro, Julio García, Mª Dolores Folgueira, Jesús Mingorance, Rafael Delgado

| EPI_ISL_421222 |  | hCoV- |  | Asia / China / |  |
| --- | --- | --- | --- | --- | --- |
|  |  | 19/Hangzhou/HZCDC6706/20 |  |  |  |
|  |  |  |  | Hangzhou |  |
|  | 20 | |  |  |  |
|  |  |  |  |  |  |
|  |  |  |  |  |  |
|  |  | hCoV- |  | Asia / China / |  |
| EPI_ISL_421224 |  | 19/Hangzhou/HZCDC0162/20 |  |  |  |
|  |  |  |  | Hangzhou |  |
|  | 20 | |  |  |  |
|  |  |  |  |  |  |
|  |  |  |  |  |  |

| EPI_ISL_422642 | hCoV- | Europe / |  |
| --- | --- | --- | --- |
|  | 19/Netherlands/Gelderland_6/ | Netherlands / |  |
|  | 2020 | Gelderland |  |

hCoV- Europe /

EPI_ISL_422643 19/Netherlands/Gelderland_7/ Netherlands /

2020 Gelderland

|  |  | hCoV- |  | Europe / |
| --- | --- | --- | --- | --- |
| EPI_ISL_422644 |  | 19/Netherlands/Gelderland_8/ |  | Netherlands / |
|  | 2020 | |  | Gelderland |
|  |  |  |  |  |

2020-03-14

2020-01-23

2020-03-09

2020-03-11

2020-03-13

Hangzhou Center for Diseases Control and Prevention


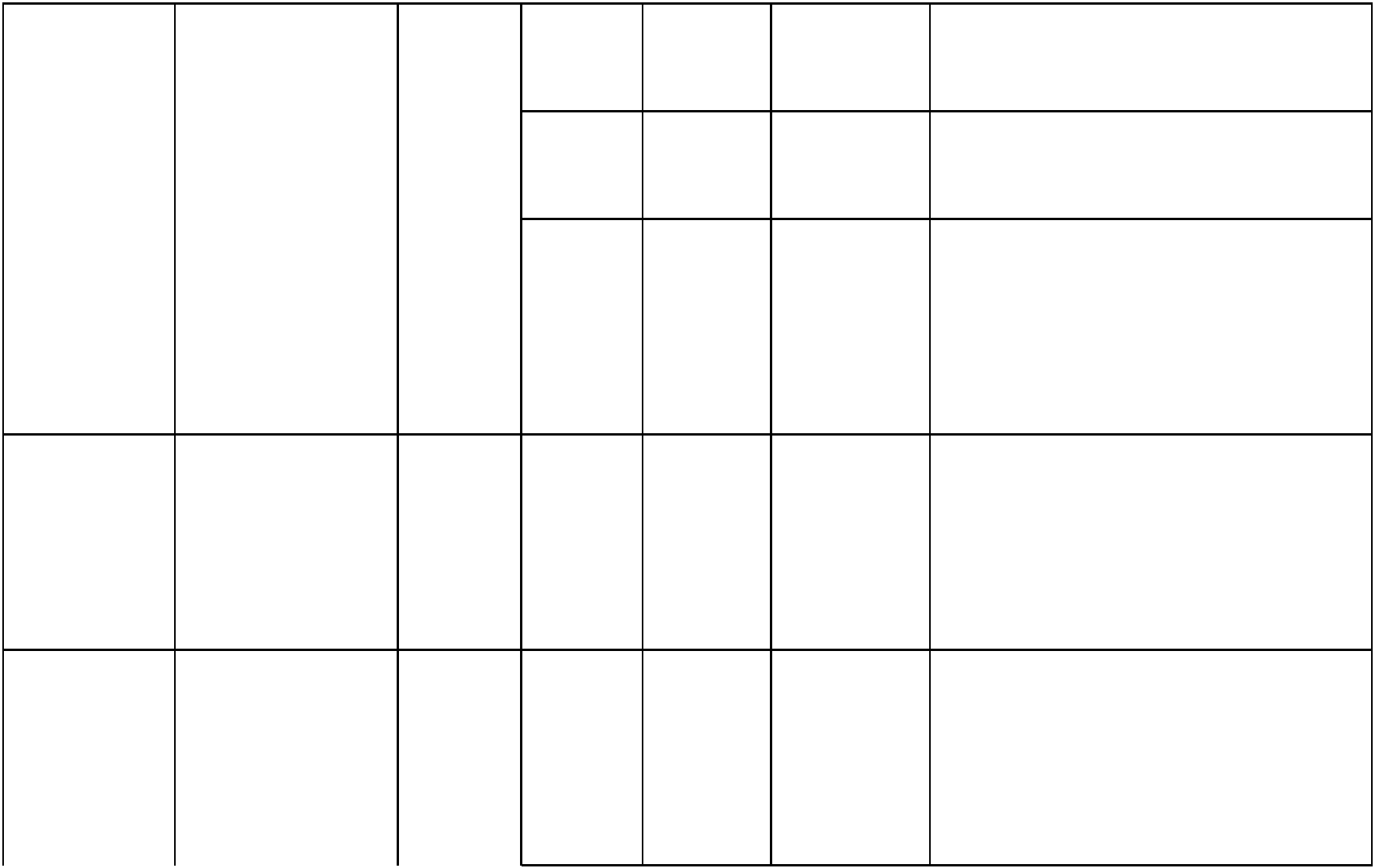


Hangzhou Center for Diseases Control and Prevention

Dutch COVID-

1. response team

Dutch COVID-

1. response team

Dutch COVID-

1. response team

Hangzhou Center for Diseases Control and Prevention

Hangzhou Center for Diseases Control and Prevention

Erasmus Medical

Center

Erasmus Medical

Center

Erasmus Medical

Center

Jun Li, Haoqiu Wang, Lingfeng Mao, Hua Yu, Xinfen Yu, Zhou Sun, Xin Qian, Shuchang Chen, Junfang Chen, Xuchu Wang

Jun Li, Haoqiu Wang, Lingfeng Mao, Hua Yu, Xinfen Yu, Zhou Sun, Xin Qian, Shuchang Chen, Junfang Chen, Xuchu Wang

Bas Oude Munnink, David Nieuwenhuijse, Reina Sikkema, Claudia Schapendonk, Irina Chestakova, Anne van der Linden, Theo Bestebroer, Stefan van Nieuwkoop, Mark Pronk, Pascal Lexmond, Corien Swaan, Manon Haverkate, Madelief Mollers, Mart Stein, Sandra Kengne Kamga Mobou, Jeroen van Kampen, Jolanda Voermans, Aura Timen, Corine GeurtsvanKessel, Annemiek van der Eijk, Richard Molenkamp, Marion Koopmans, on behalf of the Dutch national COVID-19 response team.

Bas Oude Munnink, David Nieuwenhuijse, Reina Sikkema, Claudia Schapendonk, Irina Chestakova, Anne van der Linden, Theo Bestebroer, Stefan van Nieuwkoop, Mark Pronk, Pascal Lexmond, Corien Swaan, Manon Haverkate, Madelief Mollers, Mart Stein, Sandra Kengne Kamga Mobou, Jeroen van Kampen, Jolanda Voermans, Aura Timen, Corine GeurtsvanKessel, Annemiek van der Eijk, Richard Molenkamp, Marion Koopmans, on behalf of the Dutch national COVID-19 response team.

Bas Oude Munnink, David Nieuwenhuijse, Reina Sikkema, Claudia Schapendonk, Irina Chestakova, Anne van der Linden, Theo Bestebroer, Stefan van Nieuwkoop, Mark Pronk, Pascal Lexmond, Corien Swaan, Manon Haverkate, Madelief Mollers, Mart Stein, Sandra Kengne Kamga Mobou, Jeroen van Kampen, Jolanda Voermans, Aura Timen, Corine GeurtsvanKessel, Annemiek van der Eijk, Richard Molenkamp, Marion Koopmans, on behalf of the Dutch national COVID-19 response team.


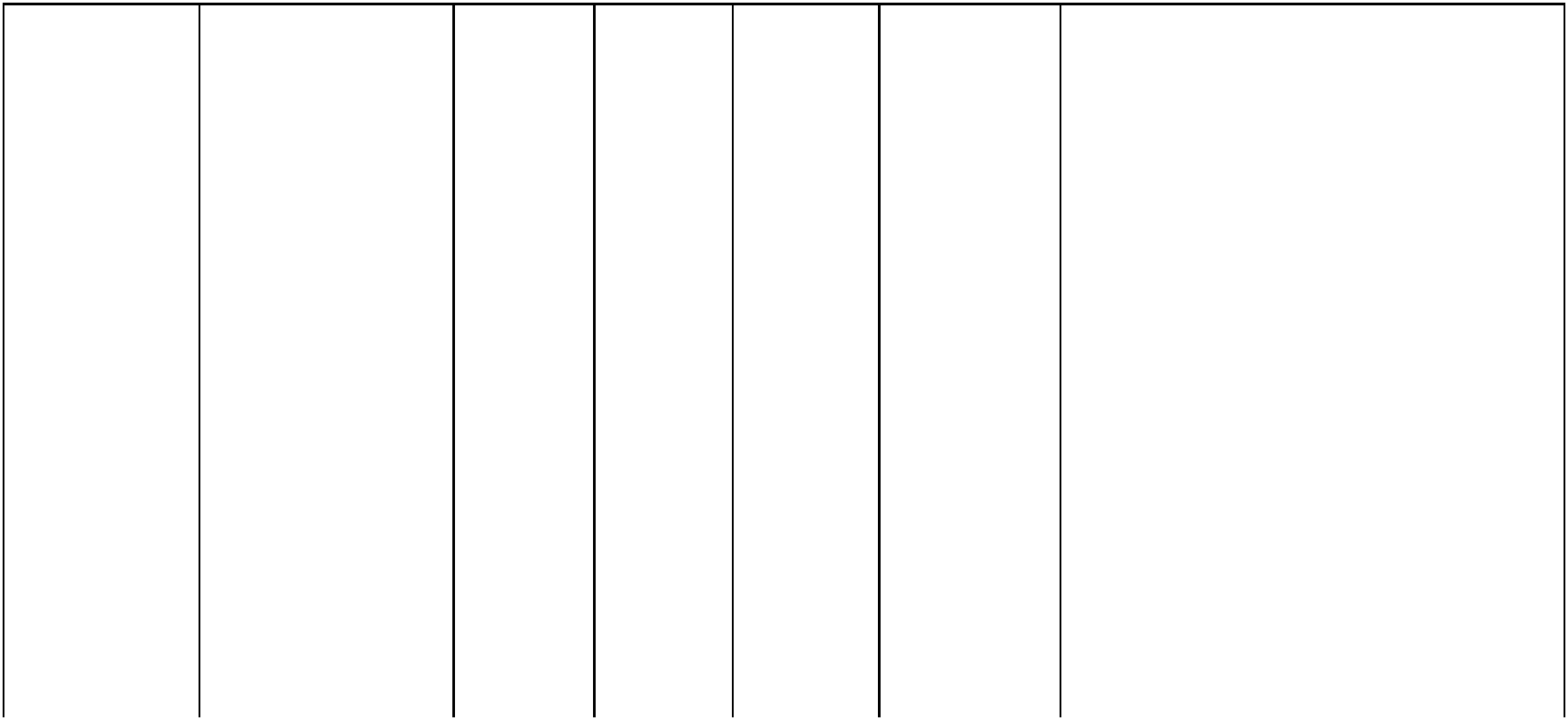


|  |  |  |  |  |  |  |  |  |  | Bas Oude Munnink, David Nieuwenhuijse, Reina Sikkema, |  |
| --- | --- | --- | --- | --- | --- | --- | --- | --- | --- | --- | --- |
|  |  |  |  |  |  |  |  |  |  | Claudia Schapendonk, Irina Chestakova, Anne van der |  |
|  |  |  |  |  |  |  |  |  |  | Linden, Theo Bestebroer, Stefan van Nieuwkoop, Mark |  |
| EPI_ISL_422662 |  | hCoV- |  | Europe / | 2020-03-10 | | Dutch COVID- |  | Erasmus Medical | Pronk, Pascal Lexmond, Corien Swaan, Manon Haverkate, |  |
|  |  |  |  |  |  |  | 19 response |  |  | Madelief Mollers, Mart Stein, Sandra Kengne Kamga Mobou, |  |
|  |  | 19/Netherlands/NA_114/2020 |  | Netherlands |  |  |  |  | Center |  |  |
|  |  |  |  |  |  |  | team |  |  | Jeroen van Kampen, Jolanda Voermans, Aura Timen, Corine |  |
|  |  |  |  |  |  |  |  |  |  |  |  |
|  |  |  |  |  |  |  |  |  |  | GeurtsvanKessel, Annemiek van der Eijk, Richard |  |
|  |  |  |  |  |  |  |  |  |  | Molenkamp, Marion Koopmans, on behalf of the Dutch |  |
|  |  |  |  |  |  |  |  |  |  | national COVID-19 response team. |  |
|  |  |  |  |  |  |  |  |  |  |  |  |
|  |  |  |  |  |  |  | Respiratory |  | Respiratory Virus |  |  |
|  |  |  |  |  |  |  | Virus Unit, |  |  | Monica Galiano, Shahjahan Miah, Angie Lackenby, Omolola |  |
|  |  | hCoV- |  | Europe / United |  |  |  |  | Unit, Microbiology |  |  |
|  |  |  |  |  |  |  | Microbiology |  |  | Akinbami, Tiina Talts, Leena Bhaw, Richard Myers, Steven |  |
| EPI_ISL_423802 |  | 19/England/20132079304/202 |  | Kingdom / | 2020-02-23 | |  |  | Services Colindale, |  |  |
|  |  |  |  |  |  |  | Services |  |  | Platt, Kirstin Edwards, Jonathan Hubb, Joanna Ellis, Maria |  |
|  | 0 | |  | England |  |  |  |  | Public Health |  |  |
|  |  |  |  |  |  |  | Colindale, Public |  |  | Zambon |  |
|  |  |  |  |  |  |  |  |  | England |  |  |
|  |  |  |  |  |  |  | Health England |  |  |  |  |
|  |  |  |  |  |  |  |  |  |  |  |  |
|  |  |  |  |  |  |  |  |  |  |  |  |
|  |  |  |  |  |  |  |  |  | Laboratory of | Concetta Castilletti, Barbara Bartolini, Martina Rueca, Cesare |  |
|  |  |  |  |  |  |  | INMI Lazzaro |  |  | Ernesto Maria Gruber, Francesco Messina, Fabrizio Carletti, |  |
|  |  |  |  | Europe / Italy / |  |  |  |  | Virology, INMI |  |  |
| EPI_ISL_424342 |  | hCoV-19/Italy/INMI8/2020 |  |  | 2020-03-07 | | Spallanzani |  |  | Eleonora Lalle, Licia Bordi, Giulia Matusali, Francesca |  |
|  |  |  |  | Rome |  |  |  |  | Lazzaro Spallanzani |  |  |
|  |  |  |  |  |  |  | IRCCS |  |  | Colavita, Maria Rosaria Capobianchi, Francesco Vairo, |  |
|  |  |  |  |  |  |  |  |  | IRCCS |  |  |
|  |  |  |  |  |  |  |  |  |  | Giuseppe Ippolito, Antonino Di Caro |  |
|  |  |  |  |  |  |  |  |  |  |  |  |
|  |  |  |  |  |  |  |  |  |  |  |  |
|  |  |  |  |  |  |  |  |  | Laboratory of | Eleonora Lalle, Barbara Bartolini, Martina Rueca, Cesare |  |
|  |  |  |  |  |  |  | INMI Lazzaro |  |  | Ernesto Maria Gruber, Francesco Messina, Fabrizio Carletti, |  |
|  |  |  |  | Europe / Italy / |  |  |  |  | Virology, INMI |  |  |
| EPI_ISL_424344 |  | hCoV-19/Italy/INMI10/2020 |  |  | 2020-03-04 | | Spallanzani |  |  | Licia Bordi, Giulia Matusali, Francesca Colavita, Maria |  |
|  |  |  |  | Rome |  |  |  |  | Lazzaro Spallanzani |  |  |
|  |  |  |  |  |  |  | IRCCS |  |  | Rosaria Capobianchi, Concetta Castilletti, Francesco Vairo, |  |
|  |  |  |  |  |  |  |  |  | IRCCS |  |  |
|  |  |  |  |  |  |  |  |  |  | Giuseppe Ippolito, Antonino Di Caro |  |
|  |  |  |  |  |  |  |  |  |  |  |  |
|  |  |  |  |  |  |  |  |  |  |  |  |


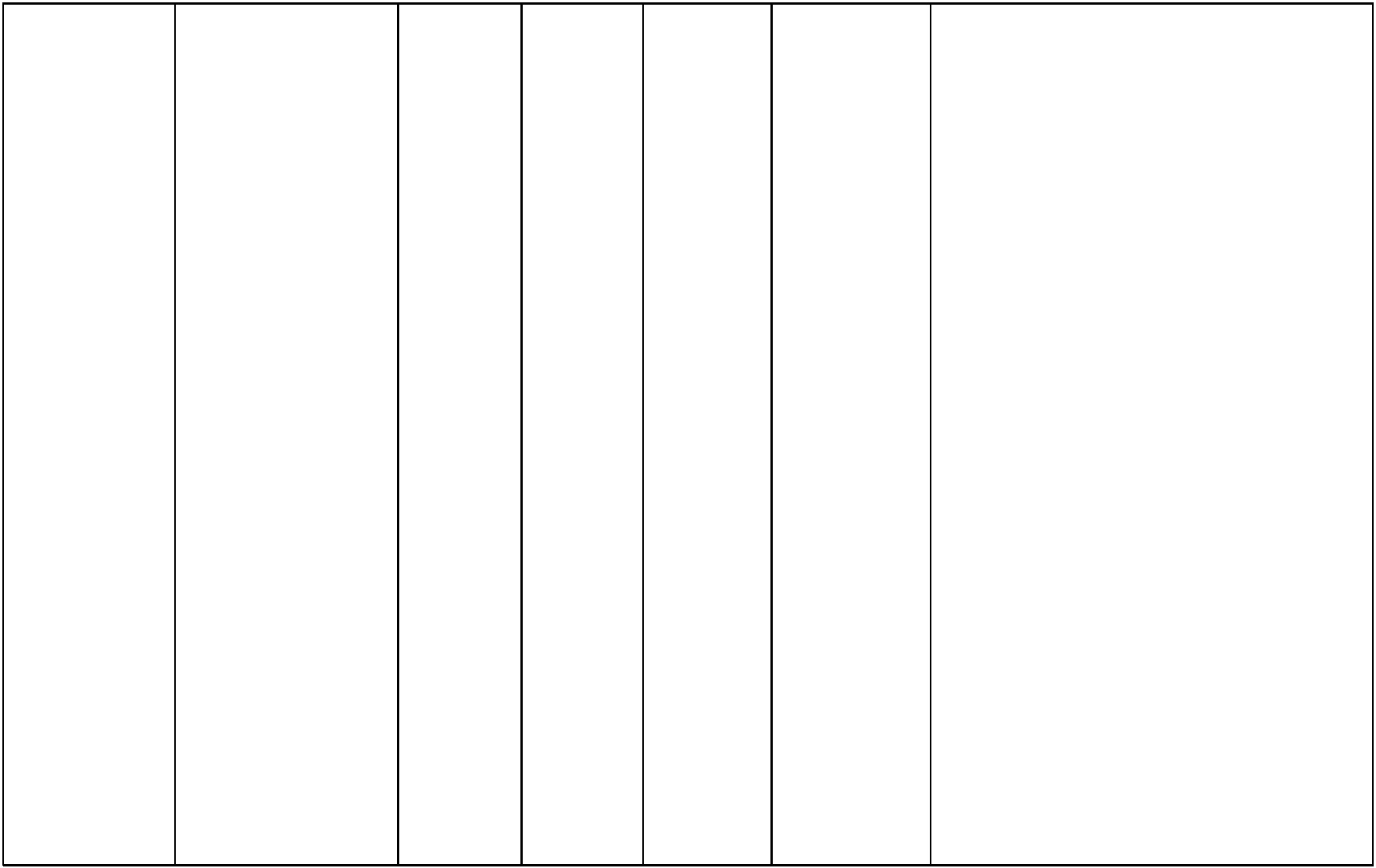


|  |  |  |  |  |  |  |  |  |  | Joel Armando Vázquez Pérez, Celia Boukadida, Santiago |  |
| --- | --- | --- | --- | --- | --- | --- | --- | --- | --- | --- | --- |
|  |  |  |  |  |  |  |  |  |  | Avila Ríos, Mario Mújica Sánchez, José Arturo Martínez |  |
|  |  |  |  |  |  |  |  |  |  | Orozco, Eduardo Becerril Vargas, Jorge Salas Hernández, |  |
|  |  |  |  |  |  |  |  |  |  | Irma López Martínez, Lucia Hernández Rivas, Gisela Barrera |  |
|  |  |  |  |  |  |  |  |  |  | Badillo, Edgar Mendieta Condado, Fabiola Garcés Ayala, |  |
|  |  |  |  | North America / |  |  | Instituto |  | Instituto Nacional de | Adnan Araiza Rodríguez, José Ernesto Ramírez González, |  |
| EPI_ISL_424345 |  | hCoV-19/Mexico/CDMX- |  |  | 2020-03-12 | | Nacional de |  |  | Victor Hugo Borja Aburto, Concepción Grajales Muñiz, Cesar |  |
|  |  |  |  | Mexico / |  |  |  |  | Enfermedades |  |  |
|  |  | INER_01/2020 |  |  |  |  | Enfermedades |  |  | Raúl González Bonilla, Carolina González Torres, Francisco |  |
|  |  |  |  | Mexico City |  |  |  |  | Respiratorias |  |  |
|  |  |  |  |  |  |  | Respiratorias |  |  | Javier Gaytán Cervantes, José Esteban Muñoz Medina, |  |
|  |  |  |  |  |  |  |  |  |  |  |  |
|  |  |  |  |  |  |  |  |  |  | Guillermo M. Ruiz-Palacios, Pilar Ramos Cervantes, Violeta |  |
|  |  |  |  |  |  |  |  |  |  | Ibarra Gonzalez, Fernando Ledesma Barrientos, Luis Alberto |  |
|  |  |  |  |  |  |  |  |  |  | García Andrade, Alfredo Ponce de León Garduño, Blanca |  |
|  |  |  |  |  |  |  |  |  |  | Taboada, Alejandro Sánchez, Pavel Isa, Ricardo Grande, |  |
|  |  |  |  |  |  |  |  |  |  | Gloria Vázquez, Francisco Pulido, Carlos F. Arias. |  |
|  |  |  |  |  |  |  |  |  |  |  |  |
| EPI_ISL_424352 |  | hCoV-19/Fuyang/FY002/2020 |  | Asia / China / | 2020-03-10 | | unknown |  | Clinical Laboratory | Ge,B. |  |
|  |  |  |  | Anhui / Fuyang |  |  |  |  |  |  |  |
|  |  |  |  |  |  |  |  |  |  |  |  |
|  |  |  |  |  |  |  |  |  |  |  |  |
|  |  | hCoV-19/Beijing/Wuhan_IME- |  | Asia / China / |  |  |  |  | Beijing Institute of | Fan,H., Qin,E., Wu,Y., Guo,Y., Zhang,X., Yong,Y., Hou,J., |  |
| EPI_ISL_424355 |  |  |  |  | 2020-01-24 | | unknown |  | Microbiology and | Xu,Z., Mu,J., Teng,Y., Mi,Z., Yang,R., Song,Y., Li,B. and |  |
|  |  | BJ01/2020 |  | Beijing |  |  |  |  |  |  |  |
|  |  |  |  |  |  |  |  |  | Epidemiology | Cui,Y. |  |
|  |  |  |  |  |  |  |  |  |  |  |  |
|  |  | hCoV-19/Beijing/Wuhan_IME- |  | Asia / China / |  |  |  |  | Beijing Institute of | Fan,H., Qin,E., Wu,Y., Guo,Y., Zhang,X., Yong,Y., Hou,J., |  |
| EPI_ISL_424356 |  |  |  |  | 2020-01-25 | | unknown |  | Microbiology and | Xu,Z., Mu,J., Teng,Y., Mi,Z., Yang,R., Song,Y., Li,B. and |  |
|  |  | BJ02/2020 |  | Beijing |  |  |  |  |  |  |  |
|  |  |  |  |  |  |  |  |  | Epidemiology | Cui,Y. |  |
|  |  |  |  |  |  |  |  |  |  |  |  |
|  |  | hCoV-19/Beijing/Wuhan_IME- |  | Asia / China / |  |  |  |  | Beijing Institute of | Fan,H., Qin,E., Wu,Y., Guo,Y., Zhang,X., Yong,Y., Hou,J., |  |
| EPI_ISL_424357 |  |  |  |  | 2020-01-28 | | unknown |  | Microbiology and | Xu,Z., Mu,J., Teng,Y., Mi,Z., Yang,R., Song,Y., Li,B. and |  |
|  |  | BJ03/2020 |  | Beijing |  |  |  |  |  |  |  |
|  |  |  |  |  |  |  |  |  | Epidemiology | Cui,Y. |  |
|  |  |  |  |  |  |  |  |  |  |  |  |
|  |  | hCoV-19/Beijing/Wuhan_IME- |  | Asia / China / |  |  |  |  | Beijing Institute of | Fan,H., Qin,E., Wu,Y., Guo,Y., Zhang,X., Yong,Y., Hou,J., |  |
| EPI_ISL_424358 |  |  |  |  | 2020-01-28 | | unknown |  | Microbiology and | Xu,Z., Mu,J., Teng,Y., Mi,Z., Yang,R., Song,Y., Li,B. and |  |
|  |  | BJ04/2020 |  | Beijing |  |  |  |  |  |  |  |
|  |  |  |  |  |  |  |  |  | Epidemiology | Cui,Y. |  |
|  |  |  |  |  |  |  |  |  |  |  |  |
|  |  | hCoV-19/Beijing/Wuhan_IME- |  | Asia / China / |  |  |  |  | Beijing Institute of | Fan,H., Qin,E., Wu,Y., Guo,Y., Zhang,X., Yong,Y., Hou,J., |  |
| EPI_ISL_424359 |  |  |  |  | 2020-01-27 | | unknown |  | Microbiology and | Xu,Z., Mu,J., Teng,Y., Mi,Z., Yang,R., Song,Y., Li,B. and |  |
|  |  | BJ05/2020 |  | Beijing |  |  |  |  |  |  |  |
|  |  |  |  |  |  |  |  |  | Epidemiology | Cui,Y. |  |
|  |  |  |  |  |  |  |  |  |  |  |  |
|  |  |  |  |  |  |  | Wadsworth |  |  |  |  |
|  |  | hCoV-19/USA/NY-Wadsworth- |  | North America / |  |  | Center, New |  | Wadsworth Center, | Kirsten St. George, Daryl M. Lamson, Sara Griesemer, |  |
| EPI_ISL_426025 |  |  |  |  | 2020-03-03 | | York State |  | New York State | Jonathan Plitnick, Navjot Singh, Matthew D. Shudt, Erica |  |
|  | 10496-01/2020 | |  | USA / New York |  |  |  |  |  |  |  |
|  |  |  |  |  |  |  | Department.of |  | Department.of Health | Lasek-Nesselquist |  |
|  |  |  |  |  |  |  |  |  |  |  |  |

Health

EPI_ISL_426031

EPI_ISL_426032

EPI_ISL_426035

EPI_ISL_426036

EPI_ISL_426039

EPI_ISL_426042

EPI_ISL_426045

EPI_ISL_426046

hCoV-19/USA/NY-Wadsworth-10704-01/2020

hCoV-19/USA/NY-Wadsworth-10707-01/2020

hCoV-19/USA/NY-Wadsworth-10957-02/2020

hCoV-19/USA/NY-Wadsworth-10958-01/2020

hCoV-19/USA/NY-Wadsworth-11003-01/2020

hCoV-19/USA/NY-Wadsworth-11202-01/2020

hCoV-19/USA/NY-Wadsworth-11344-01/2020

hCoV-19/USA/NY-Wadsworth-11353-01/2020

North America / USA / New York

North America / USA / New York

North America / USA / New York

North America / USA / New York

North America / USA / New York

North America / USA / New York

North America / USA / New York

North America / USA / New York

2020-03-04

2020-03-04

2020-03-04

2020-03-04

2020-03-05

2020-03-06

2020-03-06

2020-03-06

| Wadsworth |  |  |  |
| --- | --- | --- | --- |
| Center, New |  | Wadsworth Center, | Kirsten St. George, Daryl M. Lamson, Sara Griesemer, |
| York State |  | New York State | Jonathan Plitnick, Navjot Singh, Matthew D. Shudt, Erica |
| Department.of |  | Department.of Health | Lasek-Nesselquist |
| Health |  |  |  |
|  |  |  |  |
| Wadsworth |  |  |  |
| Center, New |  | Wadsworth Center, | Kirsten St. George, Daryl M. Lamson, Sara Griesemer, |
| York State |  | New York State | Jonathan Plitnick, Navjot Singh, Matthew D. Shudt, Erica |
| Department.of |  | Department.of Health | Lasek-Nesselquist |
| Health |  |  |  |
|  |  |  |  |
| Wadsworth |  |  |  |
| Center, New |  | Wadsworth Center, | Kirsten St. George, Daryl M. Lamson, Sara Griesemer, |
| York State |  | New York State | Jonathan Plitnick, Navjot Singh, Matthew D. Shudt, Erica |
| Department.of |  | Department.of Health | Lasek-Nesselquist |
| Health |  |  |  |
| Wadsworth |  |  |  |
| Center, New |  | Wadsworth Center, | Kirsten St. George, Daryl M. Lamson, Sara Griesemer, |
| York State |  | New York State | Jonathan Plitnick, Navjot Singh, Matthew D. Shudt, Erica |
| Department.of |  | Department.of Health | Lasek-Nesselquist |
| Health |  |  |  |
|  |  |  |  |
| Wadsworth |  |  |  |
| Center, New |  | Wadsworth Center, | Kirsten St. George, Daryl M. Lamson, Sara Griesemer, |
| York State |  | New York State | Jonathan Plitnick, Navjot Singh, Matthew D. Shudt, Erica |
| Department.of |  | Department.of Health | Lasek-Nesselquist |
| Health |  |  |  |
| Wadsworth |  |  |  |
| Center, New |  | Wadsworth Center, | Kirsten St. George, Daryl M. Lamson, Sara Griesemer, |
| York State |  | New York State | Jonathan Plitnick, Navjot Singh, Matthew D. Shudt, Erica |
| Department.of |  | Department.of Health | Lasek-Nesselquist |
| Health |  |  |  |
|  |  |  |  |
| Wadsworth |  |  |  |
| Center, New |  | Wadsworth Center, | Kirsten St. George, Daryl M. Lamson, Sara Griesemer, |
| York State |  | New York State | Jonathan Plitnick, Navjot Singh, Matthew D. Shudt, Erica |
| Department.of |  | Department.of Health | Lasek-Nesselquist |
| Health |  |  |  |
|  |  |  |  |
| Wadsworth |  |  |  |
| Center, New |  | Wadsworth Center, | Kirsten St. George, Daryl M. Lamson, Sara Griesemer, |
| York State |  | New York State | Jonathan Plitnick, Navjot Singh, Matthew D. Shudt, Erica |
| Department.of |  | Department.of Health | Lasek-Nesselquist |
| Health |  |  |  |
|  |  |  |  |


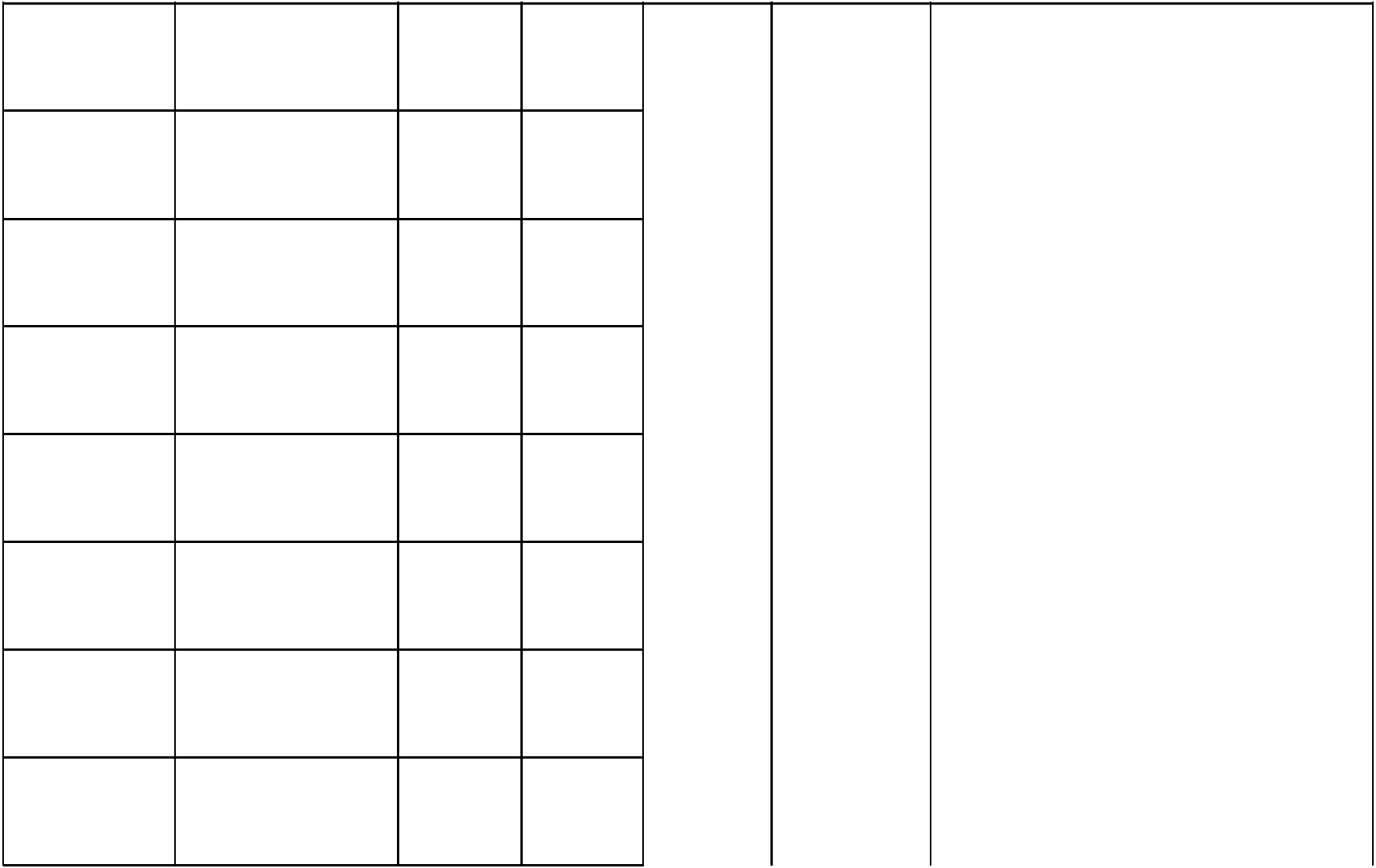


EPI_ISL_426047

EPI_ISL_426048

EPI_ISL_426163

EPI_ISL_426164

|  |  |  |  |  | Wadsworth |  |  |  |  |
| --- | --- | --- | --- | --- | --- | --- | --- | --- | --- |
| hCoV-19/USA/NY-Wadsworth- |  | North America / |  |  | Center, New |  | Wadsworth Center, | Kirsten St. George, Daryl M. Lamson, Sara Griesemer, |  |
|  |  |  | 2020-03-06 | | York State |  | New York State | Jonathan Plitnick, Navjot Singh, Matthew D. Shudt, Erica |  |
| 11354-01/2020 |  | USA / New York |  |  |  |  |  |  |  |
|  |  |  |  |  | Department.of |  | Department.of Health | Lasek-Nesselquist |  |
|  |  |  |  |  |  |  |  |  |  |
|  |  |  |  |  | Health |  |  |  |  |
|  |  |  |  |  |  |  |  |  |  |
|  |  |  |  |  | Wadsworth |  |  |  |  |
| hCoV-19/USA/NY-Wadsworth- |  | North America / |  |  | Center, New |  | Wadsworth Center, | Kirsten St. George, Daryl M. Lamson, Sara Griesemer, |  |
|  |  |  | 2020-03-06 | | York State |  | New York State | Jonathan Plitnick, Navjot Singh, Matthew D. Shudt, Erica |  |
| 11379-01/2020 |  | USA / New York |  |  |  |  |  |  |  |
|  |  |  |  |  | Department.of |  | Department.of Health | Lasek-Nesselquist |  |
|  |  |  |  |  |  |  |  |  |  |
|  |  |  |  |  | Health |  |  |  |  |
|  |  |  |  |  |  |  |  |  |  |
|  |  |  |  |  | Division of Viral |  |  |  |  |
|  |  |  |  |  | Diseases, |  |  |  |  |
|  |  |  |  |  | Center for |  | Division of Viral |  |  |
|  |  |  |  |  | Laboratory |  | Diseases, Center for |  |  |
| hCoV- |  | Asia / South | 2020-02-23 | | Control of |  | Laboratory Control of | Jeong-Min Kim, Yoon-Seok Chung, Namjoo Lee, Mi-Seon |  |
|  |  |  |  |  | Infectious |  | Infectious Diseases, | Kim, Sang Hee Woo, Hye-Jun Jo, Sehee Park, Heui Man |  |
| 19/Korea/KCDC2003/2020 |  | Korea |  |  |  |  |  |  |  |
|  |  |  |  |  | Diseases, Korea |  | Korea Centers for | Kim, Jun-Sub Kim, Junhyeong Jang, Myung Guk Han |  |
|  |  |  |  |  |  |  |  |  |  |
|  |  |  |  |  | Centers for |  | Diseases Control |  |  |
|  |  |  |  |  | Diseases |  | and Prevention |  |  |
|  |  |  |  |  | Control and |  |  |  |  |
|  |  |  |  |  | Prevention |  |  |  |  |
|  |  |  |  |  |  |  |  |  |  |
|  |  |  |  |  | Division of Viral |  |  |  |  |
|  |  |  |  |  | Diseases, |  |  |  |  |
|  |  |  |  |  | Center for |  | Division of Viral |  |  |
|  |  |  |  |  | Laboratory |  | Diseases, Center for | Jeong-Min Kim, Yoon-Seok Chung, Namjoo Lee, Mi-Seon |  |
|  |  |  |  |  | Control of |  | Laboratory Control of |  |  |
| hCoV- |  | Asia / South |  |  |  |  |  | Kim, Sang Hee Woo, Hye-Jun Jo, Sehee Park, Heui Man |  |
|  |  |  | 2020-02-02 | | Infectious |  | Infectious Diseases, |  |  |
| 19/Korea/KCDC2004/2020 |  | Korea |  |  |  |  |  | Kim, Jun-Sub Kim, Junhyeong Jang, Dong Hyun Song, |  |
|  |  |  |  |  | Diseases, Korea |  | Korea Centers for |  |  |
|  |  |  |  |  |  |  |  | Daesang Lee, Seong Tae Jeong, Myung Guk Han |  |
|  |  |  |  |  | Centers for |  | Diseases Control |  |  |
|  |  |  |  |  |  |  |  |  |  |
|  |  |  |  |  | Diseases |  | and Prevention |  |  |
|  |  |  |  |  | Control and |  |  |  |  |
|  |  |  |  |  | Prevention |  |  |  |  |
|  |  |  |  |  |  |  |  |  |  |


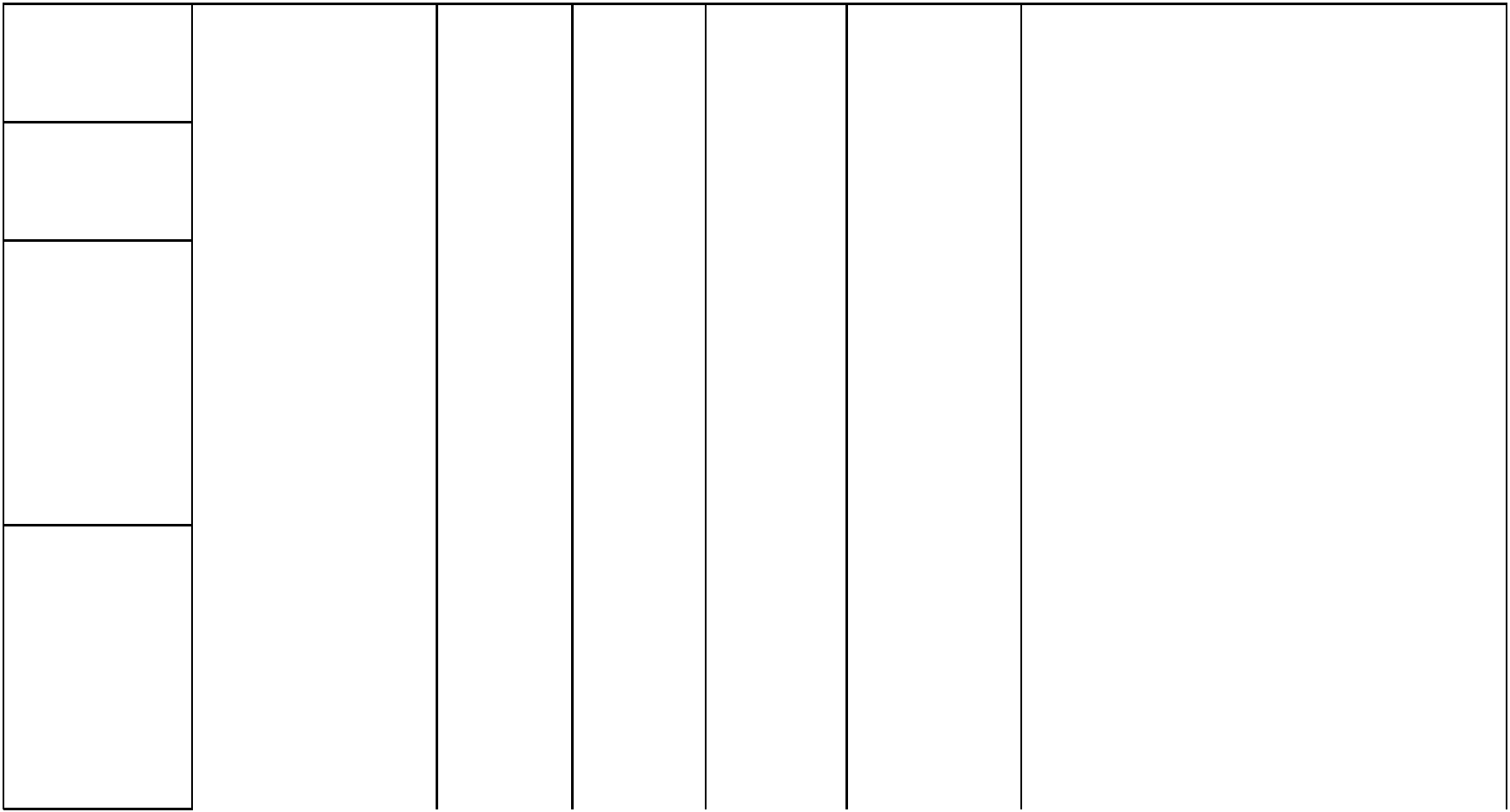


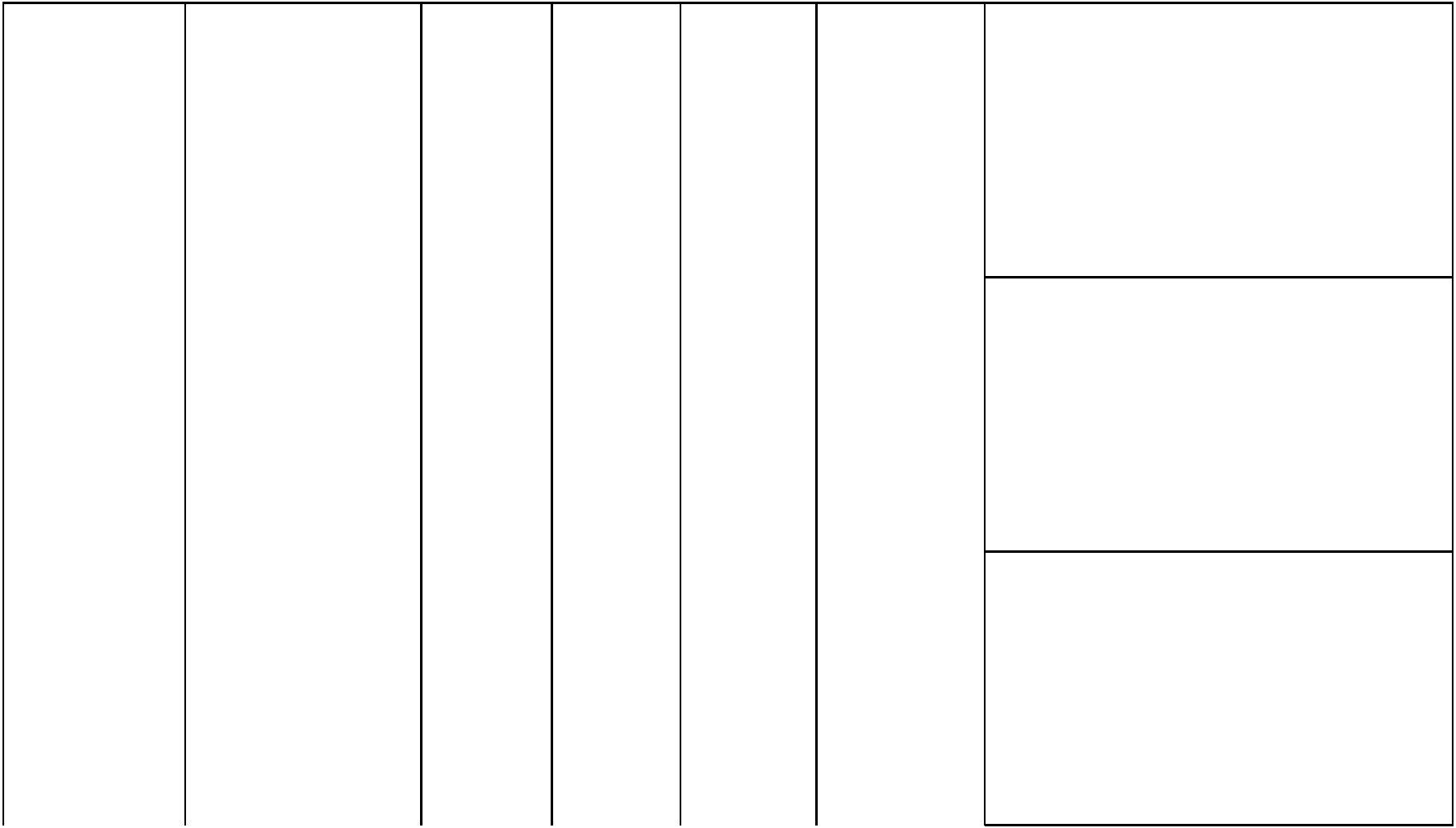


|  |  |  |  |  |  |  | Division of Viral |  |  |  |
| --- | --- | --- | --- | --- | --- | --- | --- | --- | --- | --- |
|  |  |  |  |  |  |  | Diseases, |  |  |  |
|  |  |  |  |  |  |  | Center for |  | Division of Viral |  |
|  |  |  |  |  |  |  | Laboratory |  | Diseases, Center for |  |
| EPI_ISL_426166 |  | hCoV- |  | Asia / South | 2020-02-04 | | Control of |  | Laboratory Control of |  |
|  |  |  |  |  |  |  | Infectious |  | Infectious Diseases, |  |
|  |  | 19/Korea/KCDC2005/2020 |  | Korea |  |  |  |  |  |  |
|  |  |  |  |  |  |  | Diseases, Korea |  | Korea Centers for |  |
|  |  |  |  |  |  |  |  |  |  |  |
|  |  |  |  |  |  |  | Centers for |  | Diseases Control |  |
|  |  |  |  |  |  |  | Diseases |  | and Prevention |  |
|  |  |  |  |  |  |  | Control and |  |  |  |
|  |  |  |  |  |  |  | Prevention |  |  |  |
|  |  |  |  |  |  |  |  |  |  |  |
|  |  |  |  |  |  |  | Division of Viral |  |  |  |
|  |  |  |  |  |  |  | Diseases, |  |  |  |
|  |  |  |  |  |  |  | Center for |  | Division of Viral |  |
|  |  |  |  |  |  |  | Laboratory |  | Diseases, Center for |  |
|  |  | hCoV- |  | Asia / South |  |  | Control of |  | Laboratory Control of |  |
| EPI_ISL_426168 |  |  |  |  | 2020-02-02 | | Infectious |  | Infectious Diseases, |  |
|  |  | 19/Korea/KCDC2006/2020 |  | Korea |  |  |  |  |  |  |
|  |  |  |  |  |  |  | Diseases, Korea |  | Korea Centers for |  |
|  |  |  |  |  |  |  |  |  |  |  |
|  |  |  |  |  |  |  | Centers for |  | Diseases Control |  |
|  |  |  |  |  |  |  | Diseases |  | and Prevention |  |
|  |  |  |  |  |  |  | Control and |  |  |  |
|  |  |  |  |  |  |  | Prevention |  |  |  |
|  |  |  |  |  |  |  |  |  |  |  |
|  |  |  |  |  |  |  | Division of Viral |  |  |  |
|  |  |  |  |  |  |  | Diseases, |  |  |  |
|  |  |  |  |  |  |  | Center for |  | Division of Viral |  |
|  |  |  |  |  |  |  | Laboratory |  | Diseases, Center for |  |
| EPI_ISL_426169 |  | hCoV- |  | Asia / South | 2020-02-05 | | Control of |  | Laboratory Control of |  |
|  |  |  |  |  |  |  | Infectious |  | Infectious Diseases, |  |
|  |  | 19/Korea/KCDC2007/2020 |  | Korea |  |  |  |  |  |  |
|  |  |  |  |  |  |  | Diseases, Korea |  | Korea Centers for |  |
|  |  |  |  |  |  |  |  |  |  |  |
|  |  |  |  |  |  |  | Centers for |  | Diseases Control |  |
|  |  |  |  |  |  |  | Diseases |  | and Prevention |  |
|  |  |  |  |  |  |  | Control and |  |  |  |
|  |  |  |  |  |  |  | Prevention |  |  |  |
|  |  |  |  |  |  |  |  |  |  |  |

Jeong-Min Kim, Yoon-Seok Chung, Namjoo Lee, Mi-Seon Kim, Sang Hee Woo, Hye-Jun Jo, Sehee Park, Heui Man Kim, Jun-Sub Kim, Junhyeong Jang, Myung Guk Han

Jeong-Min Kim, Yoon-Seok Chung, Namjoo Lee, Mi-Seon Kim, Sang Hee Woo, Hye-Jun Jo, Sehee Park, Heui Man Kim, Jun-Sub Kim, Junhyeong Jang, Myung Guk Han

Jeong-Min Kim, Yoon-Seok Chung, Namjoo Lee, Mi-Seon Kim, Sang Hee Woo, Hye-Jun Jo, Sehee Park, Heui Man Kim, Jun-Sub Kim, Junhyeong Jang, Dong Hyun Song, Daesang Lee, Seong Tae Jeong, Myung Guk Han


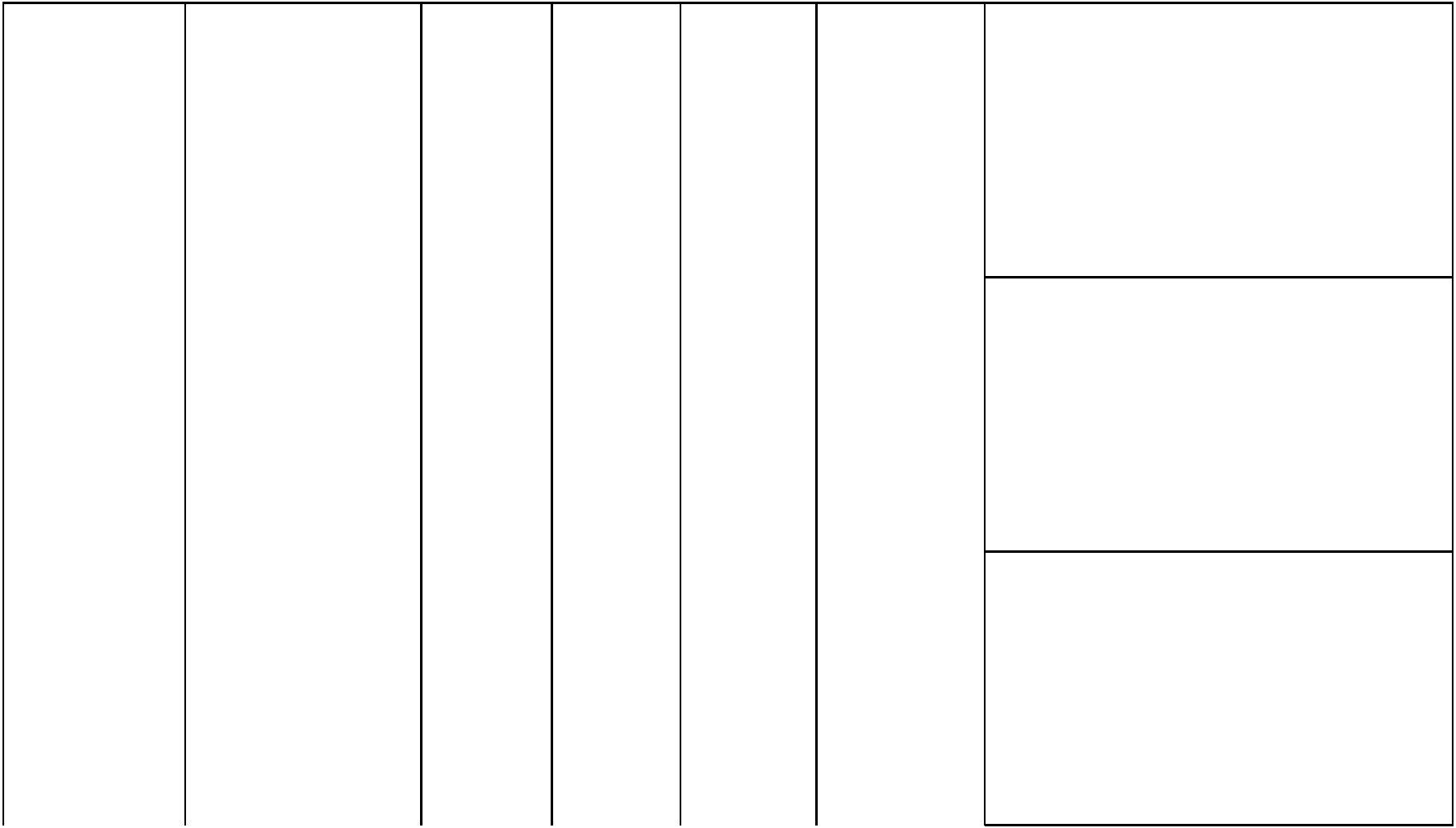


|  |  |  |  |  |  |  | Division of Viral |  |  |  |
| --- | --- | --- | --- | --- | --- | --- | --- | --- | --- | --- |
|  |  |  |  |  |  |  | Diseases, |  |  |  |
|  |  |  |  |  |  |  | Center for |  | Division of Viral |  |
|  |  |  |  |  |  |  | Laboratory |  | Diseases, Center for |  |
| EPI_ISL_426171 |  | hCoV- |  | Asia / South | 2020-02-05 | | Control of |  | Laboratory Control of |  |
|  |  |  |  |  |  |  | Infectious |  | Infectious Diseases, |  |
|  |  | 19/Korea/KCDC2008/2020 |  | Korea |  |  |  |  |  |  |
|  |  |  |  |  |  |  | Diseases, Korea |  | Korea Centers for |  |
|  |  |  |  |  |  |  |  |  |  |  |
|  |  |  |  |  |  |  | Centers for |  | Diseases Control |  |
|  |  |  |  |  |  |  | Diseases |  | and Prevention |  |
|  |  |  |  |  |  |  | Control and |  |  |  |
|  |  |  |  |  |  |  | Prevention |  |  |  |
|  |  |  |  |  |  |  |  |  |  |  |
|  |  |  |  |  |  |  | Division of Viral |  |  |  |
|  |  |  |  |  |  |  | Diseases, |  |  |  |
|  |  |  |  |  |  |  | Center for |  | Division of Viral |  |
|  |  |  |  |  |  |  | Laboratory |  | Diseases, Center for |  |
|  |  | hCoV- |  | Asia / South |  |  | Control of |  | Laboratory Control of |  |
| EPI_ISL_426173 |  |  |  |  | 2020-02-07 | | Infectious |  | Infectious Diseases, |  |
|  |  | 19/Korea/KCDC2009/2020 |  | Korea |  |  |  |  |  |  |
|  |  |  |  |  |  |  | Diseases, Korea |  | Korea Centers for |  |
|  |  |  |  |  |  |  |  |  |  |  |
|  |  |  |  |  |  |  | Centers for |  | Diseases Control |  |
|  |  |  |  |  |  |  | Diseases |  | and Prevention |  |
|  |  |  |  |  |  |  | Control and |  |  |  |
|  |  |  |  |  |  |  | Prevention |  |  |  |
|  |  |  |  |  |  |  |  |  |  |  |
|  |  |  |  |  |  |  | Division of Viral |  |  |  |
|  |  |  |  |  |  |  | Diseases, |  |  |  |
|  |  |  |  |  |  |  | Center for |  | Division of Viral |  |
|  |  |  |  |  |  |  | Laboratory |  | Diseases, Center for |  |
| EPI_ISL_426180 |  | hCoV- |  | Asia / South | 2020-02-05 | | Control of |  | Laboratory Control of |  |
|  |  |  |  |  |  |  | Infectious |  | Infectious Diseases, |  |
|  |  | 19/Korea/KCDC2010/2020 |  | Korea |  |  |  |  |  |  |
|  |  |  |  |  |  |  | Diseases, Korea |  | Korea Centers for |  |
|  |  |  |  |  |  |  |  |  |  |  |
|  |  |  |  |  |  |  | Centers for |  | Diseases Control |  |
|  |  |  |  |  |  |  | Diseases |  | and Prevention |  |
|  |  |  |  |  |  |  | Control and |  |  |  |
|  |  |  |  |  |  |  | Prevention |  |  |  |
|  |  |  |  |  |  |  |  |  |  |  |

Jeong-Min Kim, Yoon-Seok Chung, Namjoo Lee, Mi-Seon Kim, Sang Hee Woo, Hye-Jun Jo, Sehee Park, Heui Man Kim, Jun-Sub Kim, Junhyeong Jang, Dong Hyun Song, Daesang Lee, Seong Tae Jeong, Myung Guk Han

Jeong-Min Kim, Yoon-Seok Chung, Namjoo Lee, Mi-Seon Kim, Sang Hee Woo, Hye-Jun Jo, Sehee Park, Heui Man Kim, Jun-Sub Kim, Junhyeong Jang, Myung Guk Han

Jeong-Min Kim, Yoon-Seok Chung, Namjoo Lee, Mi-Seon Kim, Sang Hee Woo, Hye-Jun Jo, Sehee Park, Heui Man Kim, Jun-Sub Kim, Junhyeong Jang, Myung Guk Han


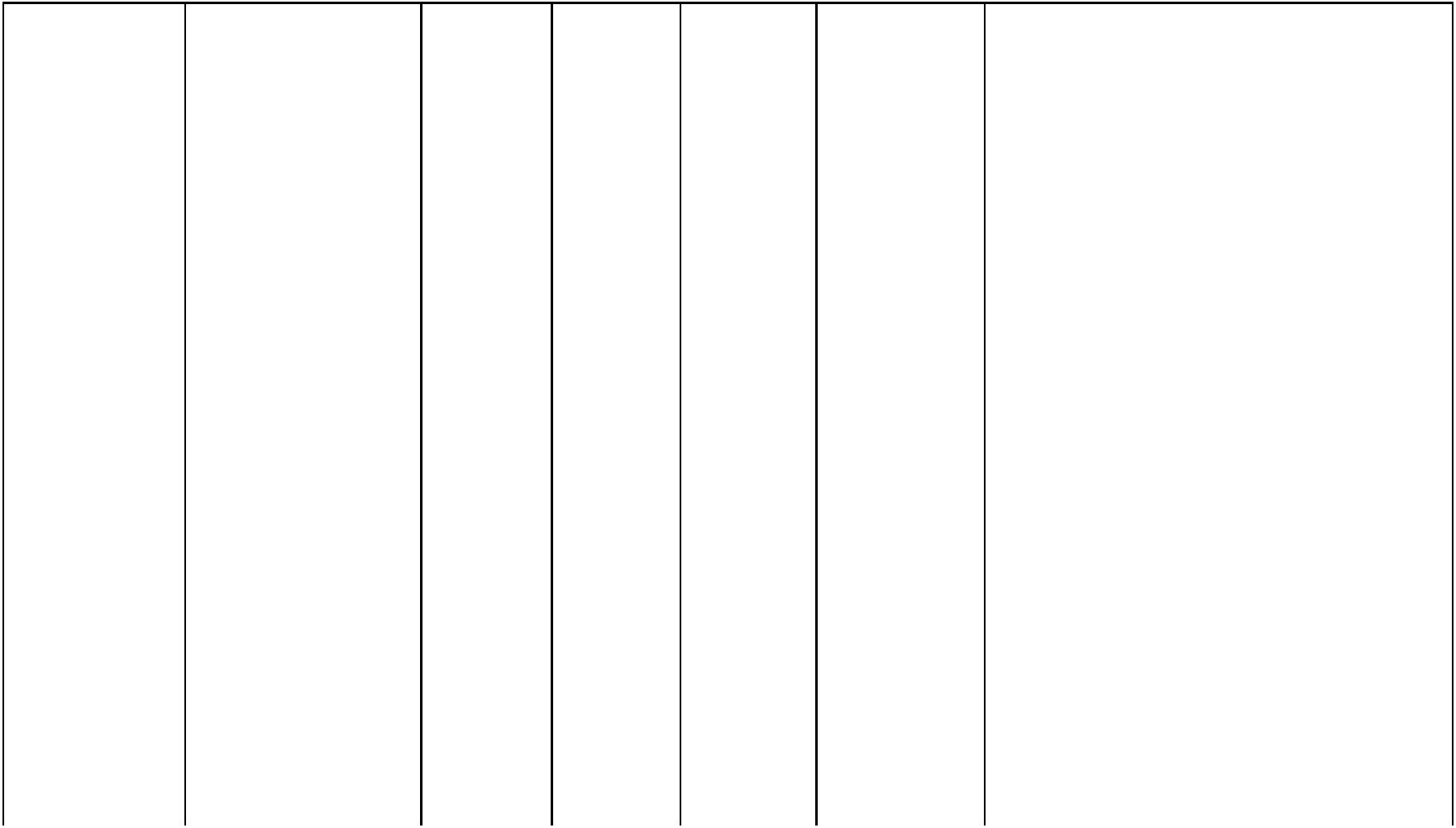


|  |  |  |  |  |  |  | Division of Viral |  |  |  |  |
| --- | --- | --- | --- | --- | --- | --- | --- | --- | --- | --- | --- |
|  |  |  |  |  |  |  | Diseases, |  |  |  |  |
|  |  |  |  |  |  |  | Center for |  | Division of Viral |  |  |
|  |  |  |  |  |  |  | Laboratory |  | Diseases, Center for |  |  |
| EPI_ISL_426181 |  | hCoV- |  | Asia / South | 2020-02-23 | | Control of |  | Laboratory Control of | Jeong-Min Kim, Yoon-Seok Chung, Namjoo Lee, Mi-Seon |  |
|  |  |  |  |  |  |  | Infectious |  | Infectious Diseases, | Kim, Sang Hee Woo, Hye-Jun Jo, Sehee Park, Heui Man |  |
|  |  | 19/Korea/KCDC2011/2020 |  | Korea |  |  |  |  |  |  |  |
|  |  |  |  |  |  |  | Diseases, Korea |  | Korea Centers for | Kim, Jun-Sub Kim, Junhyeong Jang, Myung Guk Han |  |
|  |  |  |  |  |  |  |  |  |  |  |  |
|  |  |  |  |  |  |  | Centers for |  | Diseases Control |  |  |
|  |  |  |  |  |  |  | Diseases |  | and Prevention |  |  |
|  |  |  |  |  |  |  | Control and |  |  |  |  |
|  |  |  |  |  |  |  | Prevention |  |  |  |  |
|  |  |  |  |  |  |  |  |  |  |  |  |
|  |  |  |  |  |  |  | Division of Viral |  |  |  |  |
|  |  |  |  |  |  |  | Diseases, |  |  |  |  |
|  |  |  |  |  |  |  | Center for |  | Division of Viral |  |  |
|  |  |  |  |  |  |  | Laboratory |  | Diseases, Center for |  |  |
|  |  | hCoV- |  | Asia / South |  |  | Control of |  | Laboratory Control of | Jeong-Min Kim, Yoon-Seok Chung, Namjoo Lee, Mi-Seon |  |
| EPI_ISL_426182 |  |  |  |  | 2020-02-09 | | Infectious |  | Infectious Diseases, | Kim, Sang Hee Woo, Hye-Jun Jo, Sehee Park, Heui Man |  |
|  |  | 19/Korea/KCDC2012/2020 |  | Korea |  |  |  |  |  |  |  |
|  |  |  |  |  |  |  | Diseases, Korea |  | Korea Centers for | Kim, Jun-Sub Kim, Junhyeong Jang, Myung Guk Han |  |
|  |  |  |  |  |  |  |  |  |  |  |  |
|  |  |  |  |  |  |  | Centers for |  | Diseases Control |  |  |
|  |  |  |  |  |  |  | Diseases |  | and Prevention |  |  |
|  |  |  |  |  |  |  | Control and |  |  |  |  |
|  |  |  |  |  |  |  | Prevention |  |  |  |  |
|  |  |  |  |  |  |  |  |  |  |  |  |
|  |  |  |  |  |  |  | Division of Viral |  |  |  |  |
|  |  |  |  |  |  |  | Diseases, |  |  |  |  |
|  |  |  |  |  |  |  | Center for |  | Division of Viral |  |  |
|  |  |  |  |  |  |  | Laboratory |  | Diseases, Center for |  |  |
| EPI_ISL_426183 |  | hCoV- |  | Asia / South | 2020-02-23 | | Control of |  | Laboratory Control of | Jeong-Min Kim, Yoon-Seok Chung, Namjoo Lee, Mi-Seon |  |
|  |  |  |  |  |  |  | Infectious |  | Infectious Diseases, | Kim, Sang Hee Woo, Hye-Jun Jo, Sehee Park, Heui Man |  |
|  |  | 19/Korea/KCDC2013/2020 |  | Korea |  |  |  |  |  |  |  |
|  |  |  |  |  |  |  | Diseases, Korea |  | Korea Centers for | Kim, Jun-Sub Kim, Junhyeong Jang, Myung Guk Han |  |
|  |  |  |  |  |  |  |  |  |  |  |  |
|  |  |  |  |  |  |  | Centers for |  | Diseases Control |  |  |
|  |  |  |  |  |  |  | Diseases |  | and Prevention |  |  |
|  |  |  |  |  |  |  | Control and |  |  |  |  |
|  |  |  |  |  |  |  | Prevention |  |  |  |  |
|  |  |  |  |  |  |  |  |  |  |  |  |

EPI_ISL_426187

hCoV-

19/Korea/KCDC2014/2020

Asia / South

Korea

|  | Division of Viral |  |  |  |
| --- | --- | --- | --- | --- |
|  | Diseases, |  |  |  |
|  | Center for | Division of Viral |  |  |
|  | Laboratory | Diseases, Center for |  |  |
| 2020-02-12 | Control of | Laboratory Control of | Jeong-Min Kim, Yoon-Seok Chung, Namjoo Lee, Mi-Seon |  |
|  | Infectious | Infectious Diseases, | Kim, Sang Hee Woo, Hye-Jun Jo, Sehee Park, Heui Man |  |
|  | Diseases, Korea | Korea Centers for | Kim, Jun-Sub Kim, Junhyeong Jang, Myung Guk Han |  |
|  | Centers for | Diseases Control |  |  |
|  | Diseases | and Prevention |  |  |
|  | Control and |  |  |  |
|  | Prevention |  |  |  |


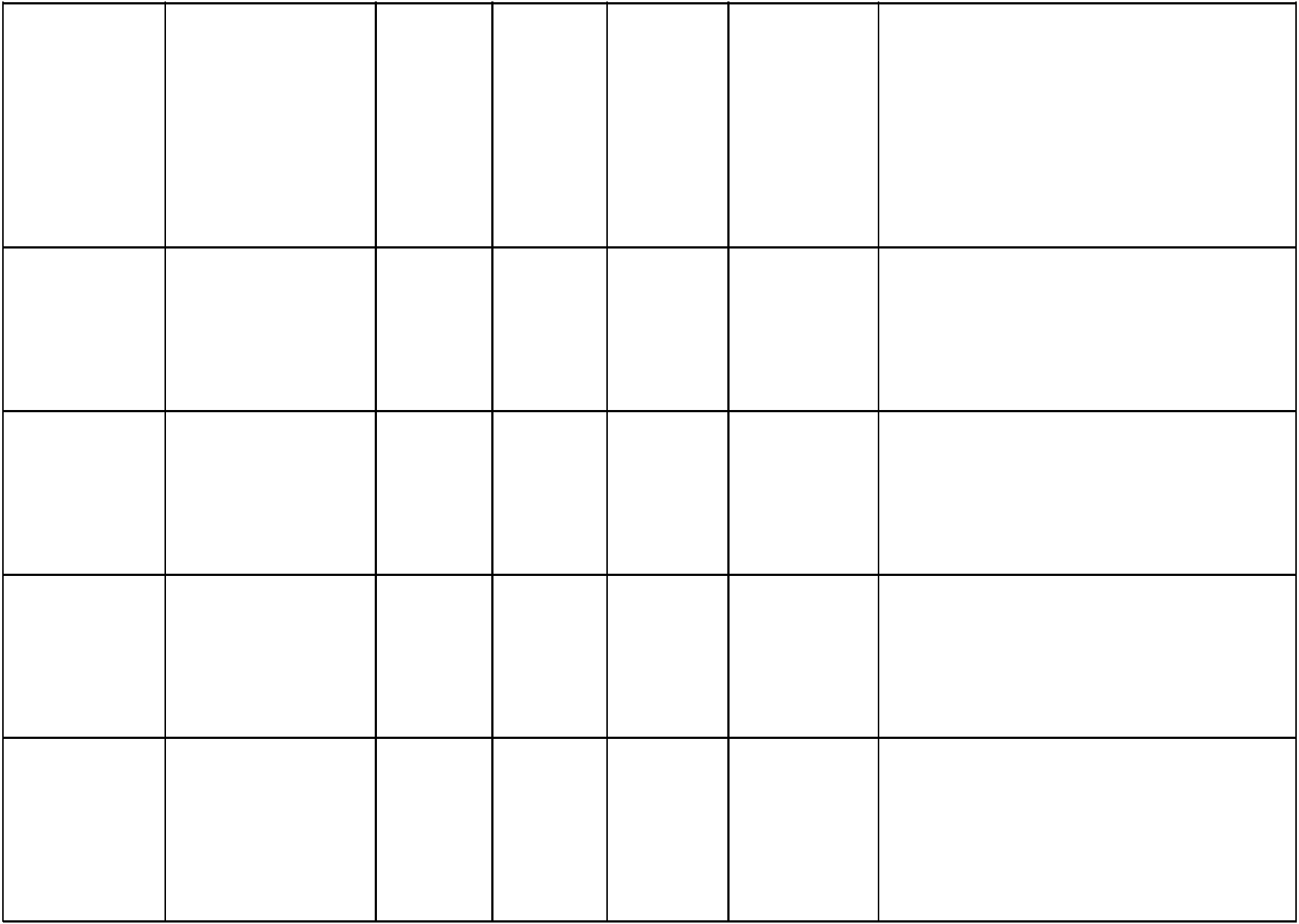


EPI_ISL_402119

EPI_ISL_402120

EPI_ISL_402121

EPI_ISL_402123

hCoV-19/Wuhan/IVDC-HB-

01/2019

hCoV-19/Wuhan/IVDC-HB-

04/2020

hCoV-19/Wuhan/IVDC-HB-

05/2019

hCoV-19/Wuhan/IPBCAMS-

WH-01/2019

Asia / China / Hubei / Wuhan

Asia / China / Hubei / Wuhan

Asia / China / Hubei / Wuhan

Asia / China / Hubei / Wuhan

2019-12-30

2020-01-01

2019-12-30

2019-12-24

National

Institute for Viral Disease Control and Prevention, China CDC

National

Institute for Viral Disease Control and Prevention, China CDC

National

Institute for Viral Disease Control and Prevention, China CDC

Institute of

Pathogen

Biology,

Chinese

Academy of

Medical

Sciences &

Peking Union

Medical College

National Institute for Viral Disease Control and Prevention, China CDC

National Institute for Viral Disease Control and Prevention, China CDC

National Institute for Viral Disease Control and Prevention, China CDC

Institute of Pathogen Biology, Chinese Academy of Medical Sciences & Peking Union Medical College

Wenjie Tan，Xiang Zhao，Wenling Wang，Xuejun Ma

，Yongzhong Jiang，Roujian Lu, Ji Wang, Weimin

Zhou，Peihua Niu，Peipei Liu，Faxian Zhan，Weifeng Shi，Baoying Huang，Jun Liu，Li Zhao，Yao Meng， Xiaozhou He，Fei Ye，Na Zhu，Yang Li，Jing Chen， Wenbo Xu，George F. Gao，Guizhen Wu

Wenjie Tan，Xiang Zhao，Wenling Wang，Xuejun Ma， Yongzhong Jiang，Roujian Lu，Ji Wang，Weimin Zhou， Peihua Niu，Peipei Liu，Faxian Zhan，Weifeng Shi， Baoying Huang，Jun Liu，Li Zhao，Yao Meng，Xiaozhou He，Fei Ye，Na Zhu，Yang Li，Jing Chen，Wenbo Xu， George F. Gao，Guizhen Wu

Wenjie Tan，Xuejun Ma，Xiang Zhao，Wenling Wang， Yongzhong Jiang，Roujian Lu，Ji Wang，Peihua Niu, Weimin Zhou, Faxian Zhan，Weifeng Shi，Baoying Huang，Jun Liu，Li Zhao，Yao Meng，Fei Ye，Na Zhu, Xiaozhou He，Peipei Liu, Yang Li，Jing Chen，Wenbo Xu，George F. Gao，Guizhen Wu

Lili Ren, Jianwei Wang, Qi Jin, Zichun Xiang, Zhiqiang Wu, Chao Wu, Yiwei Liu


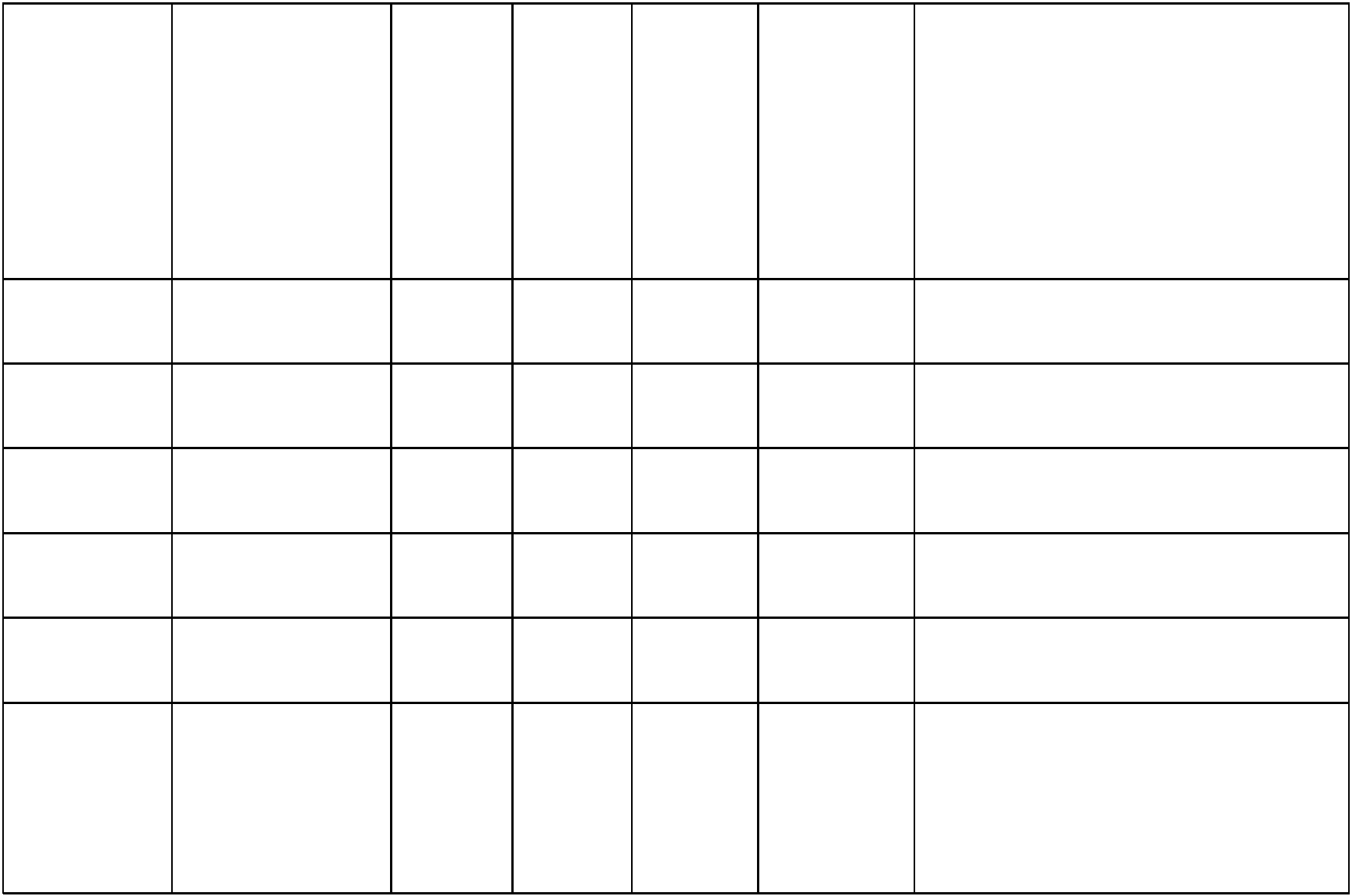


|  |  |  |  | Asia / China / |  |  | Wuhan Jinyintan |  | Wuhan Institute of | Peng Zhou, Xing-Lou Yang, Ding-Yu Zhang, Lei Zhang, Yan |  |
| --- | --- | --- | --- | --- | --- | --- | --- | --- | --- | --- | --- |
| EPI_ISL_402124 |  | hCoV-19/Wuhan/WIV04/2019 |  |  | 2019-12-30 | |  |  | Virology, Chinese |  |  |
|  |  |  |  | Hubei / Wuhan |  |  | Hospital |  |  | Zhu, Hao-Rui Si, Zhengli Shi |  |
|  |  |  |  |  |  |  |  |  | Academy of Sciences |  |  |
|  |  |  |  |  |  |  |  |  |  |  |  |
|  |  |  |  |  |  |  |  |  |  |  |  |
|  |  |  |  |  |  |  |  |  | National Institute for |  |  |
|  |  |  |  |  |  |  |  |  | Communicable | Zhang,Y.-Z., Wu,F., Chen,Y.-M., Pei,Y.-Y., Xu,L., Wang,W., |  |
|  |  |  |  |  |  |  |  |  | Disease Control and |  |  |
| EPI_ISL_402125 |  | hCoV-19/Wuhan-Hu-1/2019 |  | Asia / China | 2019-12-31 | | unknown |  | Prevention (ICDC) | Zhao,S., Yu,B., Hu,Y., Tao,Z.-W., Song,Z.-G., Tian,J.-H., |  |
|  |  |  |  |  |  |  |  |  | Chinese Center for | Zhang,Y.-L., Liu,Y., Zheng,J.-J., Dai,F.-H., Wang,Q.-M., |  |
|  |  |  |  |  |  |  |  |  |  |  |  |
|  |  |  |  |  |  |  |  |  | Disease Control and | She,J.-L. and Zhu,T.-Y. |  |
|  |  |  |  |  |  |  |  |  | Prevention (China |  |  |
|  |  |  |  |  |  |  |  |  | CDC) |  |  |

EPI_ISL_402127

EPI_ISL_402128

EPI_ISL_402129

EPI_ISL_402130

EPI_ISL_402132

EPI_ISL_403929

hCoV-19/Wuhan/WIV02/2019

hCoV-19/Wuhan/WIV05/2019

hCoV-19/Wuhan/WIV06/2019

hCoV-19/Wuhan/WIV07/2019

hCoV-19/Wuhan/HBCDC-HB-01/2019

hCoV-19/Wuhan/IPBCAMS-

WH-04/2019

Asia / China / Hubei / Wuhan

Asia / China / Hubei / Wuhan

Asia / China / Hubei / Wuhan

Asia / China / Hubei / Wuhan

Asia / China / Hubei / Wuhan

Asia / China / Hubei / Wuhan

2019-12-30

2019-12-30

2019-12-30

2019-12-30

2019-12-30

2019-12-30

Wuhan Jinyintan

Hospital

Wuhan Jinyintan

Hospital

Wuhan Jinyintan

Hospital

Wuhan Jinyintan

Hospital

Wuhan Jinyintan

Hospital

Institute of

Pathogen

Biology,

Chinese

Academy of

Medical

Sciences &

Peking Union

Medical College

Wuhan Institute of Virology, Chinese Academy of Sciences

Wuhan Institute of Virology, Chinese Academy of Sciences

Wuhan Institute of Virology, Chinese Academy of Sciences

Wuhan Institute of Virology, Chinese Academy of Sciences

Hubei Provincial Center for Disease Control and Prevention

Institute of Pathogen Biology, Chinese Academy of Medical Sciences & Peking Union Medical College

Peng Zhou, Xing-Lou Yang, Ding-Yu Zhang, Lei Zhang, Yan Zhu, Hao-Rui Si, Zhengli Shi

Peng Zhou, Xing-Lou Yang, Ding-Yu Zhang, Lei Zhang, Yan Zhu, Hao-Rui Si, Zhengli Shi

Peng Zhou, Xing-Lou Yang, Ding-Yu Zhang, Lei Zhang, Yan Zhu, Hao-Rui Si, Zhengli Shi

Peng Zhou, Xing-Lou Yang, Ding-Yu Zhang, Lei Zhang, Yan Zhu, Hao-Rui Si, Zhengli Shi

Bin Fang, Xiang Li, Xiao Yu, Linlin Liu, Bo Yang, Faxian Zhan, Guojun Ye, Xixiang Huo, Junqiang Xu, Bo Yu, Kun Cai, Jing Li, Yongzhong Jiang.

Lili Ren, Jianwei Wang, Qi Jin, Zichun Xiang, Zhiqiang Wu, Chao Wu, Yiwei Liu

EPI_ISL_403930

EPI_ISL_403931

EPI_ISL_403932

EPI_ISL_403933

hCoV-19/Wuhan/IPBCAMS-

WH-03/2019

hCoV-19/Wuhan/IPBCAMS-

WH-02/2019

hCoV-

19/Guangdong/20SF012/2020

hCoV-

19/Guangdong/20SF013/2020

Asia / China / Hubei / Wuhan

Asia / China / Hubei / Wuhan

Asia / China / Guandong / Shenzhen

Asia / China / Guandong / Shenzhen

2019-12-30

2019-12-30

2020-01-14

2020-01-15

Institute of


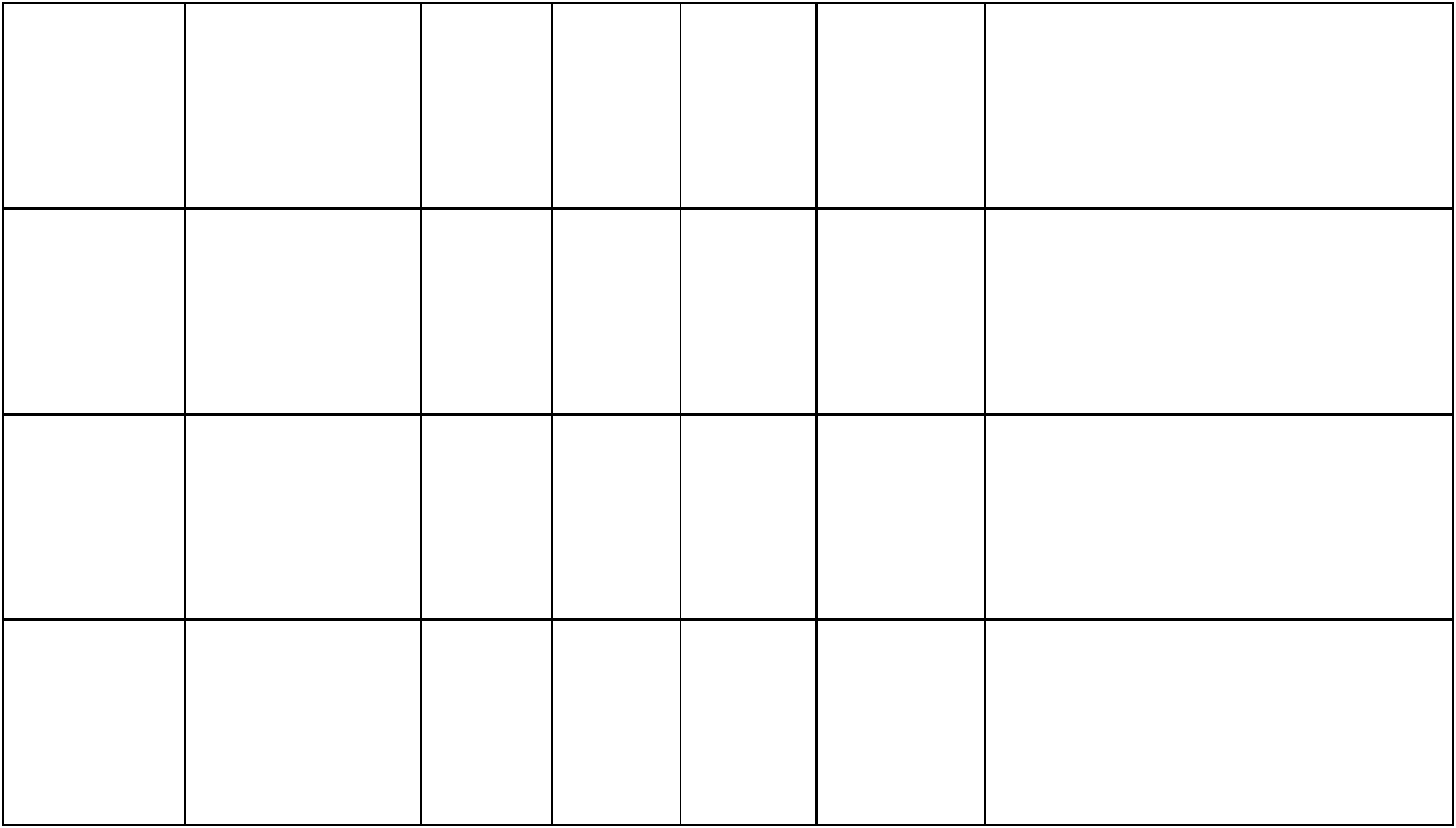


Pathogen

Biology,

Chinese

Academy of

Medical

Sciences &

Peking Union

Medical College

Institute of

Pathogen

Biology,

Chinese

Academy of

Medical

Sciences &

Peking Union

Medical College

Guangdong

Provincial Center for Diseases Control and Prevention; Guangdong Provincial Public Health

Guangdong

Provincial Center for Diseases Control and Prevention; Guangdong Provincial Public Health

Institute of Pathogen Biology, Chinese Academy of Medical Sciences & Peking Union Medical College

Institute of Pathogen Biology, Chinese Academy of Medical Sciences & Peking Union Medical College

Department of Microbiology, Guangdong Provincial Center for Diseases Control and Prevention

Department of Microbiology, Guangdong Provincial Center for Diseases Control and Prevention

Lili Ren, Jianwei Wang, Qi Jin, Zichun Xiang, Zhiqiang Wu, Chao Wu, Yiwei Liu

Lili Ren, Jianwei Wang, Qi Jin, Zichun Xiang, Zhiqiang Wu, Chao Wu, Yiwei Liu

Min Kang, Jie Wu, Jing Lu, Tao Liu, Baisheng Li, Shujiang Mei, Feng Ruan, Lifeng Lin, Changwen Ke, Haojie Zhong, Yingtao Zhang, Lirong Zou, Xuguang Chen, Qi Zhu, Jianpeng Xiao, Jianxiang Geng, Zhe Liu, Jianxiong Hu, Weilin Zeng, Xing Li, Yuhuang Liao, Xiujuan Tang, Songjian Xiao, Ying Wang, Yingchao Song, Xue Zhuang, Lijun Liang, Guanhao He, Huihong Deng, Tie Song, Jianfeng He, Wenjun Ma

Min Kang, Jie Wu, Jing Lu, Tao Liu, Baisheng Li, Shujiang Mei, Feng Ruan, Lifeng Lin, Changwen Ke, Haojie Zhong, Yingtao Zhang, Lirong Zou, Xuguang Chen, Qi Zhu, Jianpeng Xiao, Jianxiang Geng, Zhe Liu, Jianxiong Hu, Weilin Zeng, Xing Li, Yuhuang Liao, Xiujuan Tang, Songjian Xiao, Ying Wang, Yingchao Song, Xue Zhuang, Lijun Liang, Guanhao He, Huihong Deng, Tie Song, Jianfeng He, Wenjun Ma

EPI_ISL_403934

EPI_ISL_403935

EPI_ISL_403936

EPI_ISL_403937

hCoV-

19/Guangdong/20SF014/2020

hCoV-

19/Guangdong/20SF025/2020

hCoV-

19/Guangdong/20SF028/2020

hCoV-

19/Guangdong/20SF040/2020

|  |  |  | Guangdong |  |
| --- | --- | --- | --- | --- |
|  |  |  | Provincial |  |
| Asia / China / |  |  | Center for |  |
|  |  |  | Diseases |  |
| Guandong / | 2020-01-15 | | Control and |  |
| Shenzhen |  |  | Prevention; |  |
|  |  |  | Guangdong |  |
|  |  |  | Provincial Public |  |
|  |  |  | Health |  |
|  |  |  |  |  |
|  |  |  | Guangdong |  |
|  |  |  | Provincial |  |
|  |  |  | Center for |  |
| Asia / China / | 2020-01-15 | | Diseases |  |
| Guangdong / |  |  | Control and |  |
| Shenzhen |  |  | Prevention; |  |
|  |  |  | Guangdong |  |
|  |  |  | Provincial Public |  |
|  |  |  | Health |  |
|  |  |  |  |  |
|  |  |  | Guangdong |  |
|  |  |  | Provincial |  |
| Asia / China / |  |  | Center for |  |
|  |  |  | Diseases |  |
| Guangdong / | 2020-01-17 | | Control and |  |
| Zhuhai |  |  | Prevention; |  |
|  |  |  | Guangdong |  |
|  |  |  | Provincial Public |  |
|  |  |  | Health |  |
|  |  |  |  |  |
|  |  |  | Guangdong |  |
|  |  |  | Provincial |  |
|  |  |  | Center for |  |
| Asia / China / | 2020-01-18 | | Diseases |  |
| Guangdong / |  |  | Control and |  |
| Zhuhai |  |  | Prevention; |  |
|  |  |  | Guangdong |  |


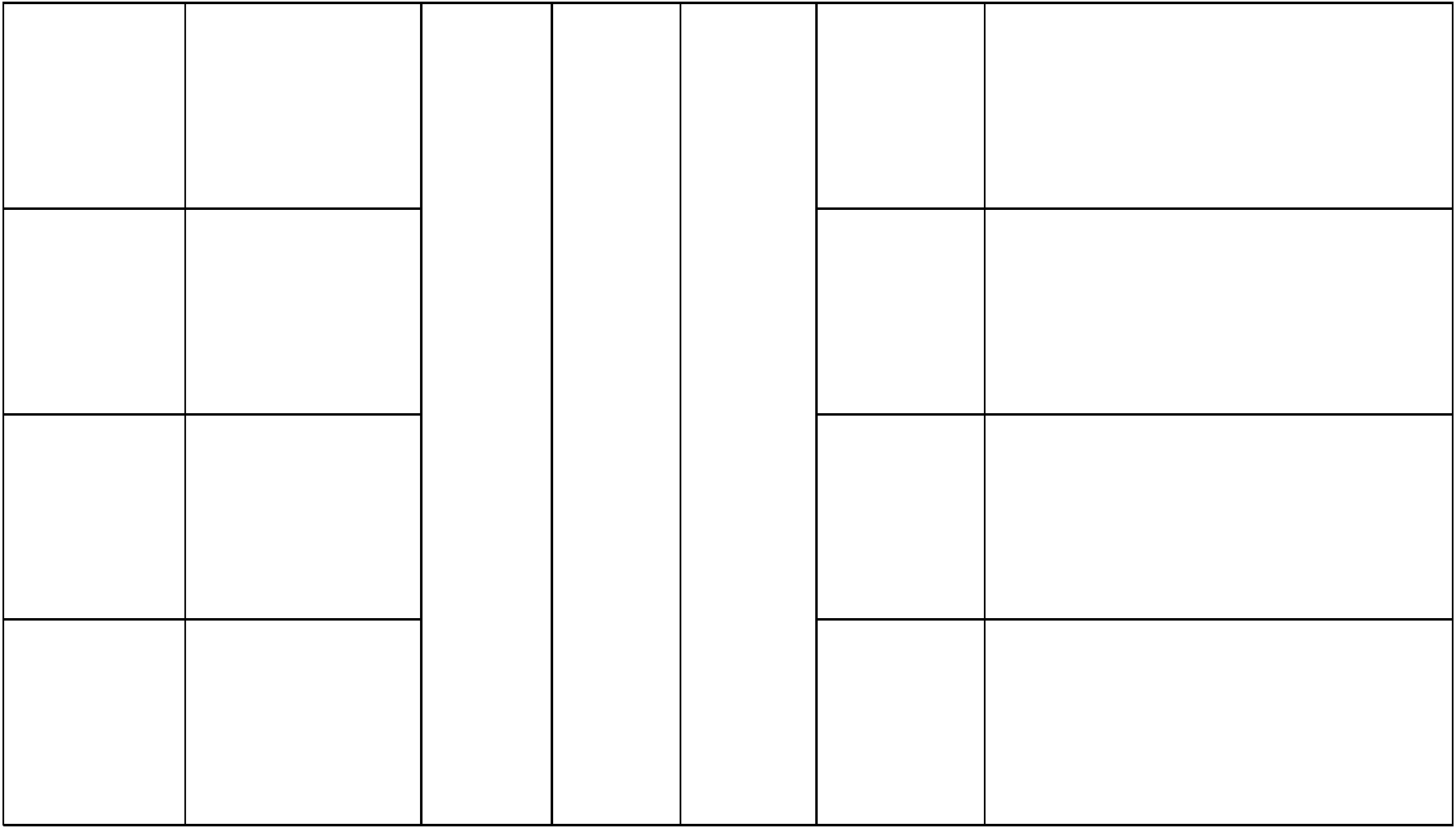


Provincial Public

Health

Department of Microbiology, Guangdong Provincial Center for Diseases Control and Prevention

Department of Microbiology, Guangdong Provincial Center for Diseases Control and Prevention

Department of Microbiology, Guangdong Provincial Center for Diseases Control and Prevention

Department of Microbiology, Guangdong Provincial Center for Diseases Control and Prevention

Min Kang, Jie Wu, Jing Lu, Tao Liu, Baisheng Li, Shujiang Mei, Feng Ruan, Lifeng Lin, Changwen Ke, Haojie Zhong, Yingtao Zhang, Lirong Zou, Xuguang Chen, Qi Zhu, Jianpeng Xiao, Jianxiang Geng, Zhe Liu, Jianxiong Hu, Weilin Zeng, Xing Li, Yuhuang Liao, Xiujuan Tang, Songjian Xiao, Ying Wang, Yingchao Song, Xue Zhuang, Lijun Liang, Guanhao He, Huihong Deng, Tie Song, Jianfeng He, Wenjun Ma

Min Kang, Jie Wu, Jing Lu, Tao Liu, Baisheng Li, Shujiang Mei, Feng Ruan, Lifeng Lin, Changwen Ke, Haojie Zhong, Yingtao Zhang, Lirong Zou, Xuguang Chen, Qi Zhu, Jianpeng Xiao, Jianxiang Geng, Zhe Liu, Jianxiong Hu, Weilin Zeng, Xing Li, Yuhuang Liao, Xiujuan Tang, Songjian Xiao, Ying Wang, Yingchao Song, Xue Zhuang, Lijun Liang, Guanhao He, Huihong Deng, Tie Song, Jianfeng He, Wenjun Ma

Min Kang, Jie Wu, Jing Lu, Tao Liu, Baisheng Li, Shujiang Mei, Feng Ruan, Lifeng Lin, Changwen Ke, Haojie Zhong, Yingtao Zhang, Lirong Zou, Xuguang Chen, Qi Zhu, Jianpeng Xiao, Jianxiang Geng, Zhe Liu, Jianxiong Hu, Weilin Zeng, Xing Li, Yuhuang Liao, Xiujuan Tang, Songjian Xiao, Ying Wang, Yingchao Song, Xue Zhuang, Lijun Liang, Guanhao He, Huihong Deng, Tie Song, Jianfeng He, Wenjun Ma

Min Kang, Jie Wu, Jing Lu, Tao Liu, Baisheng Li, Shujiang Mei, Feng Ruan, Lifeng Lin, Changwen Ke, Haojie Zhong, Yingtao Zhang, Lirong Zou, Xuguang Chen, Qi Zhu, Jianpeng Xiao, Jianxiang Geng, Zhe Liu, Jianxiong Hu, Weilin Zeng, Xing Li, Yuhuang Liao, Xiujuan Tang, Songjian Xiao, Ying Wang, Yingchao Song, Xue Zhuang, Lijun Liang, Guanhao He, Huihong Deng, Tie Song, Jianfeng He, Wenjun Ma


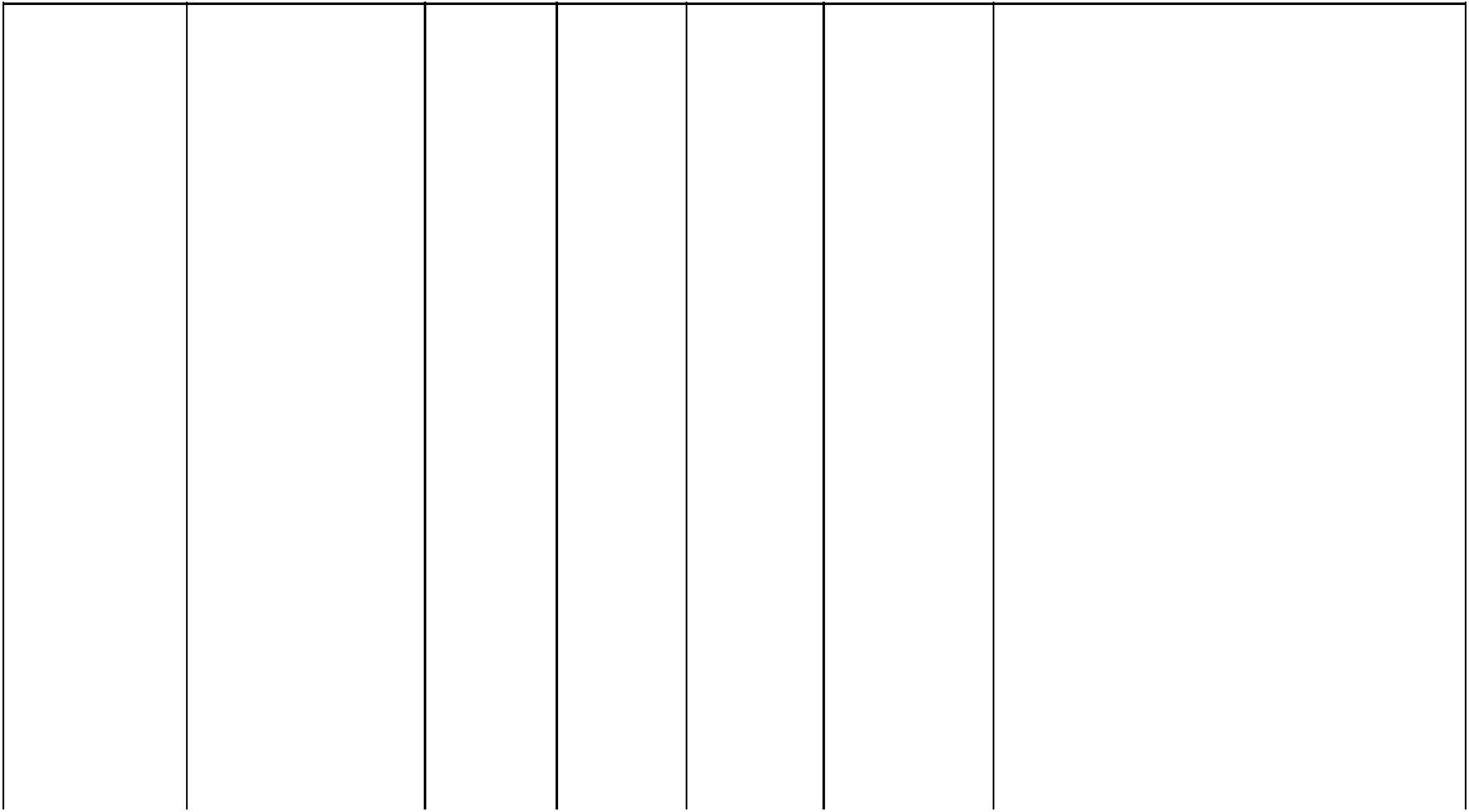


|  |  |  |  |  |  |  |  |  | 1. Department of |  |  |
| --- | --- | --- | --- | --- | --- | --- | --- | --- | --- | --- | --- |
|  |  |  |  |  |  |  |  |  | Medical Sciences, |  |  |
|  |  |  |  |  |  |  |  |  | Ministry of Public |  |  |
|  |  |  |  |  |  |  |  |  | Health, Thailand 2. | Pilailuk,Okada; Siripaporn,Phuygun; |  |
|  |  |  |  |  |  |  |  |  | Thai Red Cross |  |  |
|  |  |  |  |  |  |  |  |  |  | Thanutsapa,Thanadachakul; Supaporn,Wacharapluesadee; |  |
|  |  |  |  | Asia / Thailand |  |  | Bamrasnaradura |  | Emerging Infectious |  |  |
| EPI_ISL_403962 |  | hCoV-19/Thailand/61/2020 |  |  | 2020-01-08 | |  |  |  | Sittiporn,Parnmen; Warawan,Wongboot; |  |
|  |  |  |  | / Nonthaburi |  |  | Hospital |  | Diseases - Health |  |  |
|  |  |  |  |  |  |  |  |  |  | Sunthareeya,Waicharoen; Rome,Buathong; |  |
|  |  |  |  |  |  |  |  |  | Science Centre 3. |  |  |
|  |  |  |  |  |  |  |  |  |  | Malinee,Chittaganpitch; Nanthawan,Mekha |  |
|  |  |  |  |  |  |  |  |  | Department of |  |  |
|  |  |  |  |  |  |  |  |  |  |  |  |
|  |  |  |  |  |  |  |  |  | Disease Control, |  |  |
|  |  |  |  |  |  |  |  |  | Ministry of Public |  |  |
|  |  |  |  |  |  |  |  |  | Health, Thailand |  |  |
|  |  |  |  |  |  |  |  |  |  |  |  |
|  |  |  |  |  |  |  |  |  | 1. Department of |  |  |
|  |  |  |  |  |  |  |  |  | Medical Sciences, |  |  |
|  |  |  |  |  |  |  |  |  | Ministry of Public |  |  |
|  |  |  |  |  |  |  |  |  | Health, Thailand 2. | Pilailuk,Okada; Siripaporn,Phuygun; |  |
|  |  |  |  |  |  |  |  |  | Thai Red Cross |  |  |
|  |  |  |  | Asia / Thailand |  |  | Bamrasnaradura |  |  | Thanutsapa,Thanadachakul; Supaporn,Wacharapluesadee; |  |
|  |  |  |  |  |  |  |  |  | Emerging Infectious |  |  |
| EPI_ISL_403963 |  | hCoV-19/Thailand/74/2020 |  |  | 2020-01-13 | |  |  |  | Sittiporn,Parnmen; Warawan,Wongboot; |  |
|  |  |  |  | / Nonthaburi |  |  | Hospital |  | Diseases - Health |  |  |
|  |  |  |  |  |  |  |  |  |  | Sunthareeya,Waicharoen; Rome,Buathong; |  |
|  |  |  |  |  |  |  |  |  | Science Centre 3. |  |  |
|  |  |  |  |  |  |  |  |  |  | Malinee,Chittaganpitch; Nanthawan,Mekha |  |
|  |  |  |  |  |  |  |  |  | Department of |  |  |
|  |  |  |  |  |  |  |  |  |  |  |  |
|  |  |  |  |  |  |  |  |  | Disease Control, |  |  |
|  |  |  |  |  |  |  |  |  | Ministry of Public |  |  |
|  |  |  |  |  |  |  |  |  | Health, Thailand |  |  |
|  |  |  |  |  |  |  |  |  |  |  |  |
|  |  |  |  |  |  |  |  |  | Pathogen Discovery, |  |  |
|  |  |  |  |  |  |  |  |  | Respiratory Viruses | Ying Tao, Clinton R. Paden, Krista Queen, Anna Uehara, Yan |  |
|  |  |  |  | North America / |  |  | Arizona |  | Branch, Division of | Li, Jing Zhang, Xiaoyan Lu, Brian Lynch, Senthil Kumar K. |  |
| EPI_ISL_406223 |  | hCoV-19/USA/AZ1/2020 |  | USA / Arizona / | 2020-01-22 | | Department of |  | Viral Diseases, | Sakthivel, Brett L. Whitaker, Shifaq Kamili, Lijuan Wang, |  |
|  |  |  |  | Phoenix |  |  | Health Services |  | Centers for Disease | Janna' R. Murray, Susan I. Gerber, Stephen Lindstrom, |  |
|  |  |  |  |  |  |  |  |  | Control and | Suxiang Tong |  |
|  |  |  |  |  |  |  |  |  | Prevention |  |  |
|  |  |  |  |  |  |  |  |  |  |  |  |


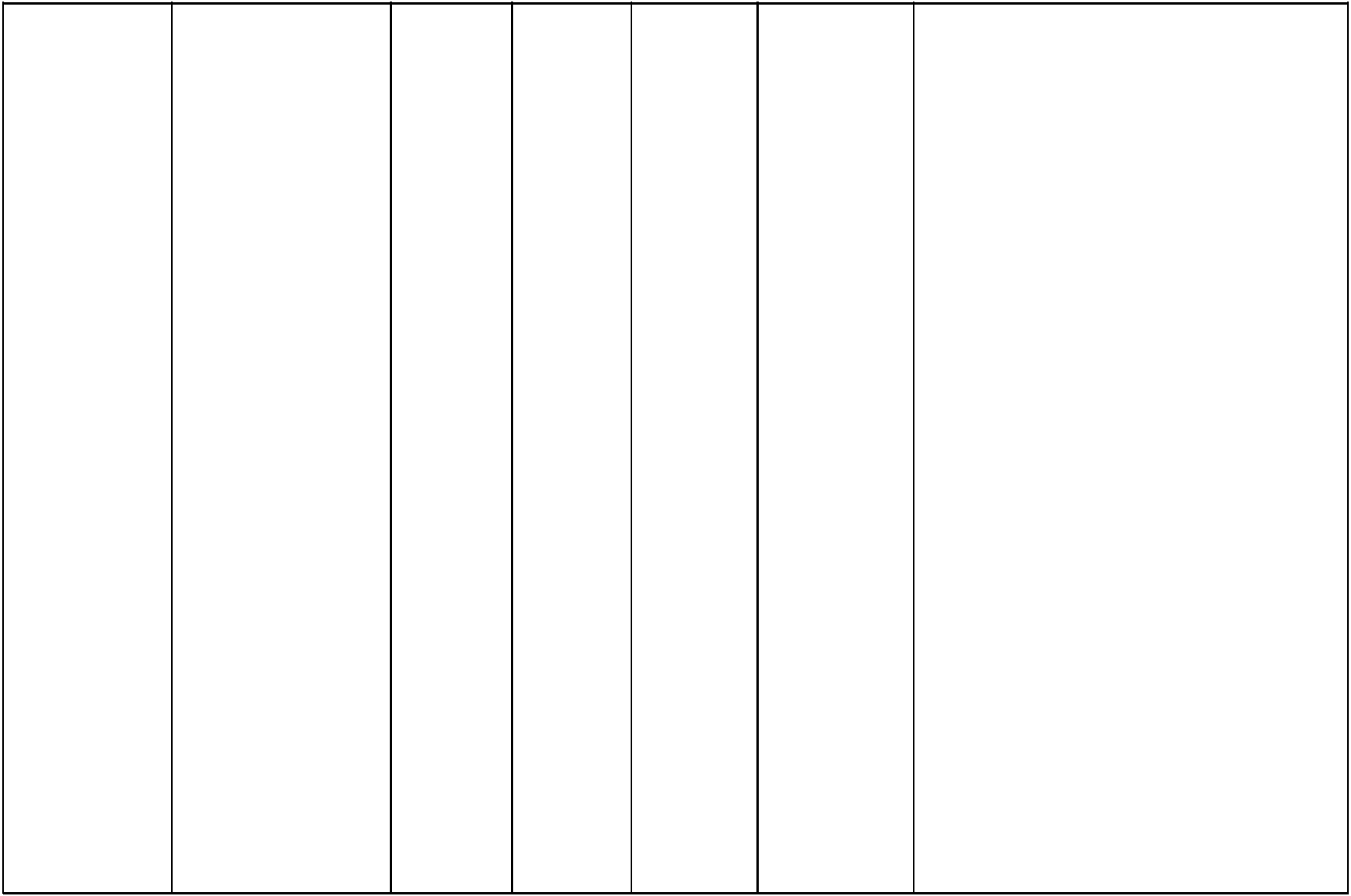


|  |  |  |  |  |  |  |  |  | Pathogen Discovery, |  |  |
| --- | --- | --- | --- | --- | --- | --- | --- | --- | --- | --- | --- |
|  |  |  |  |  |  |  | California |  | Respiratory Viruses | Krista Queen, Jing Zhang, Yan Li, Ying Tao, Anna Uehara, |  |
|  |  |  |  | North America / |  |  |  |  | Branch, Division of | Clinton Paden, Xiaoyan Lu, Brian Lynch, Senthil Kumar K. |  |
| EPI_ISL_408008 |  | hCoV-19/USA/CA3/2020 |  |  | 2020-01-29 | | Department of |  | Viral Diseases, | Sakthivel, Brett L. Whitaker, Shifaq Kamili, Lijuan Wang, |  |
|  |  |  |  | USA / California |  |  |  |  |  |  |  |
|  |  |  |  |  |  |  | Health |  | Centers for Disease | Janna' R. Murray, Susan I. Gerber, Stephen Lindstrom, |  |
|  |  |  |  |  |  |  |  |  |  |  |  |
|  |  |  |  |  |  |  |  |  | Control and | Suxiang Tong |  |
|  |  |  |  |  |  |  |  |  | Prevention |  |  |
|  |  |  |  |  |  |  |  |  |  |  |  |
|  |  |  |  |  |  |  |  |  | Pathogen Discovery, | Krista Queen, Jing Zhang, Yan Li, Ying Tao, Anna Uehara, |  |
|  |  |  |  |  |  |  |  |  | Respiratory Viruses |  |  |
| EPI_ISL_408009 |  | hCoV-19/USA/CA4/2020 |  | North America / | 2020-01-29 | | California |  | Branch, Division of | Clinton Paden, Xiaoyan Lu, Brian Lynch, Senthil Kumar K. |  |
|  |  |  |  |  |  |  | Department of |  | Viral Diseases, | Sakthivel, Brett L. Whitaker, Shifaq Kamili, Lijuan Wang, |  |
|  |  |  |  | USA / California |  |  |  |  |  |  |  |
|  |  |  |  |  |  |  | Health |  | Centers for Dieases | Janna' R. Murray, Susan I. Gerber, Stephen Lindstrom, |  |
|  |  |  |  |  |  |  |  |  |  |  |  |
|  |  |  |  |  |  |  |  |  | Control and | Suxiang Tong |  |
|  |  |  |  |  |  |  |  |  | Prevention |  |  |
|  |  |  |  |  |  |  |  |  |  |  |  |
|  |  |  |  |  |  |  |  |  | Pathogen Discovery, |  |  |
|  |  |  |  |  |  |  | California |  | Respiratory Viruses | Ying Tao, Krista Queen, Jing Zhang, Yan Li, Anna Uehara, |  |
|  |  |  |  | North America / |  |  |  |  | Branch, Division of | Clinton Paden, Xiaoyan Lu, Brian Lynch, Senthil Kumar K. |  |
| EPI_ISL_408010 |  | hCoV-19/USA/CA5/2020 |  |  | 2020-01-29 | | Department of |  | Viral Diseases, | Sakthivel, Brett L. Whitaker, Shifaq Kamili, Lijuan Wang, |  |
|  |  |  |  | USA / California |  |  |  |  |  |  |  |
|  |  |  |  |  |  |  | Health |  | Centers for Dieases | Janna' R. Murray, Susan I. Gerber, Stephen Lindstrom, |  |
|  |  |  |  |  |  |  |  |  |  |  |  |
|  |  |  |  |  |  |  |  |  | Control and | Suxiang Tong |  |
|  |  |  |  |  |  |  |  |  | Prevention |  |  |
|  |  |  |  |  |  |  |  |  |  |  |  |
|  |  |  |  |  |  |  | Department of |  | Microbial Genomics |  |  |
|  |  |  |  |  |  |  | Laboratory |  | Core Lab, National |  |  |
| EPI_ISL_410218 |  | hCoV-19/Taiwan/NTU02/2020 |  | Asia / Taiwan / | 2020-02-05 | | Medicine, |  | Taiwan University | Shiou-Hwei Yeh, You-Yu Lin, Ya-Yun Lai, Chiao-Ling Li, |  |
|  |  |  |  | Taipei |  |  | National Taiwan |  | Centers of Genomic | Shan-Chwen Chang, Pei-Jer Chen, Sui-Yuan Chang |  |
|  |  |  |  |  |  |  |  |  |  |  |  |
|  |  |  |  |  |  |  | University |  | and Precision |  |  |
|  |  |  |  |  |  |  | Hospital |  | Medicine |  |  |
|  |  |  |  |  |  |  |  |  |  |  |  |
|  |  |  |  |  |  |  | National |  |  |  |  |
|  |  |  |  |  |  |  | Influenza |  |  |  |  |
|  |  |  |  | Asia / Nepal / |  |  | Centre, National |  | The University of | Ranjit Sah , Runa Jha, Daniel Chu, Haogao Gu, Malik Peiris, |  |
| EPI_ISL_410301 |  | hCoV-19/Nepal/61/2020 |  |  | 2020-01-13 | | Public Health |  |  | Anup Bastola, Alfonso J. Rodriguez-Morales, Bibek Kumar |  |
|  |  |  |  | Kathmandu |  |  |  |  | Hong Kong |  |  |
|  |  |  |  |  |  |  | Laboratory, |  |  | Lal, Basu Dev Pandey, Leo Poon |  |
|  |  |  |  |  |  |  |  |  |  |  |  |

Kathmandu,

Nepal

EPI_ISL_411060

EPI_ISL_411066

EPI_ISL_412898

EPI_ISL_412899

EPI_ISL_412912

EPI_ISL_412966

EPI_ISL_412967

EPI_ISL_412968

EPI_ISL_412969

hCoV-19/Fujian/8/2020

hCoV-19/Fujian/13/2020

hCoV-19/Wuhan/HBCDC-HB-02/2019

hCoV-19/Wuhan/HBCDC-HB-03/2019

hCoV-19/Germany/Baden-

Wuerttemberg-1/2020

hCoV-19/China/IQTC01/2020

hCoV-19/China/IQTC02/2020

hCoV-

19/Japan/Hu_DP_Kng_19-

020/2020

hCoV-

19/Japan/Hu_DP_Kng_19-

027/2020

Asia / China /

Fujian

Asia / China /

Fujian

Asia / China / Hubei / Wuhan

Asia / China / Hubei / Wuhan

Europe /

Germany /

Baden-

Wuerttemberg

Asia / China /

Guangdong /

Guangzhou

Asia / China / Guangzhou

Asia / Japan

Asia / Japan

2020-01-21

2020-01-22

2019-12-30

2019-12-30

2020-02-25

2020-02-05

2020-01-29

2020-02-10

2020-02-10

Fujian Center for Disease Control and Prevention


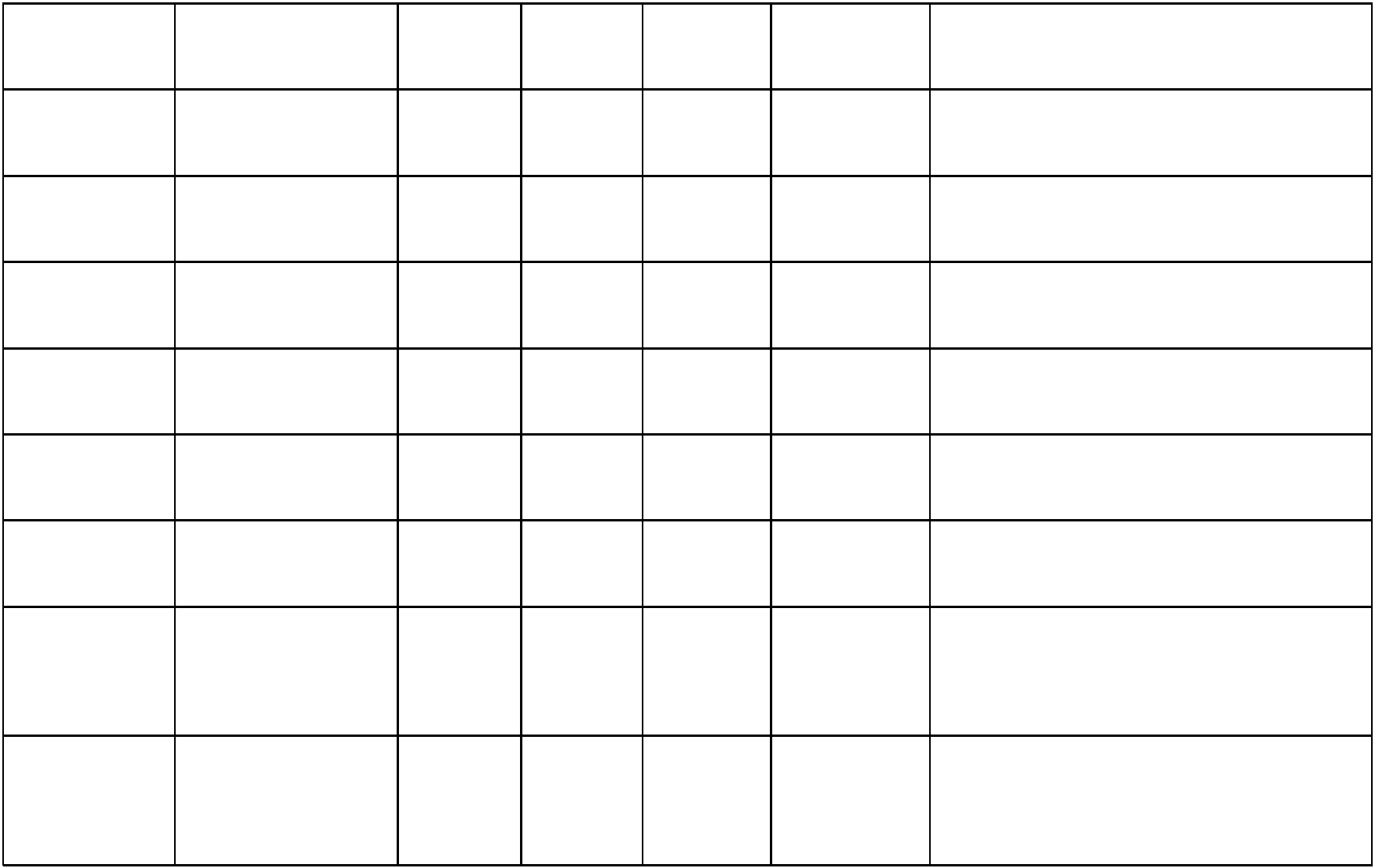


Fujian Center for Disease Control and Prevention

Wuhan Jinyintan

Hospital

Wuhan Jinyintan

Hospital

State Health Office Baden-Wuerttemberg

unknown

unknown

unknown

unknown

Fujian Center for Disease Control and Prevention

Fujian Center for Disease Control and Prevention

Hubei Provincial Center for Disease Control and Prevention

Hubei Provincial Center for Disease Control and Prevention

Charité

Universitätsmedizin

Berlin, Institute of

Virology

Technology Centre, Guangzhou Customs

Technology Centre, Guangzhou Customs

Takayuki Hishiki Kanagawa Prefectural Institute of Public Health, Department of Microbiology

Takayuki Hishiki Kanagawa Prefectural Institute of Public Health, Department of Microbiology

Chen Wei, Zhang Yanhua, He Wenxiang, Weng Yuwei

Chen Wei, Zhang Yanhua, He Wenxiang, Weng Yuwei

Bin Fang, Xiang Li, Xiao Yu, Linlin Liu, Bo Yang, Faxian Zhan, Guojun Ye, Xixiang Huo, Junqiang Xu, Bo Yu, Kun Cai, Jing Li, Yongzhong Jiang.

Bin Fang, Xiang Li, Xiao Yu, Linlin Liu, Bo Yang, Faxian Zhan, Guojun Ye, Xixiang Huo, Junqiang Xu, Bo Yu, Kun Cai, Jing Li, Yongzhong Jiang.

Victor M Corman, Julia Schneider, Barbara Mühlemann, Talitha Veith, Jörn Beheim-Schwarzbach, Terry Jones, Rainer Oehme, Silke Fischer, Christian Drosten

Shi,Y., Sun,J., Zheng,K., Huang,J. and Zhao,J.

Shi,Y., Zheng,K., Sun,J., Huang,J., Zhu,A., Zhuang,Z., Dai,J., Chen,Z., Sun,F., Zhang,Z., Li,X. and Wang,Y.

Hishiki,T., Suzuki,R., Sakuragi,J., Usui,K., Tanaka,Y., Kawai,J., Kogo,Y., Matsuki,Y., An,T., Hayashizaki,Y. and Takasaki,T.

Hishiki,T., Suzuki,R., Sakuragi,J., Usui,K., Tanaka,Y., Kawai,J., Kogo,Y., Matsuki,Y., An,T., Hayashizaki,Y. and Takasaki,T.

EPI_ISL_412970

EPI_ISL_412972

EPI_ISL_412973

EPI_ISL_412974

EPI_ISL_412975

hCoV-19/USA/WA2/2020

hCoV-19/Mexico/CDMX-

InDRE_01/2020

hCoV-19/Italy/CDG1/2020

hCoV-19/Italy/SPL1/2020

hCoV-

19/Australia/NSW05/2020

North America /

USA /

Washington /

Snohomish

County

North America / Mexico / Mexico City

Europe / Italy /

Lombardy

Europe / Italy /

Rome

Oceania / Australia / New South Wales / Sydney

2020-02-24

2020-02-27

2020-02-20

2020-01-29

2020-02-28

Washington


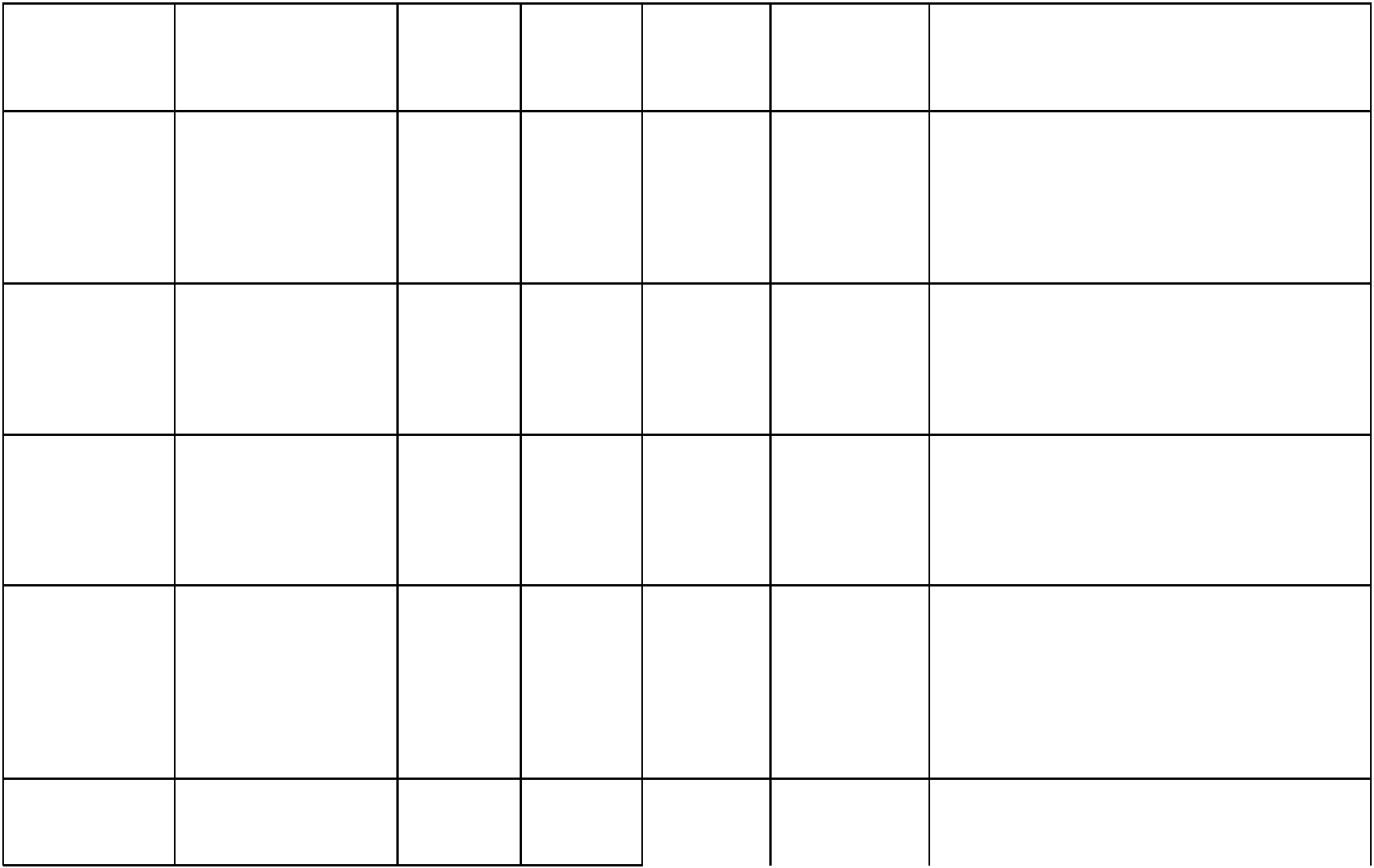


State

Department of

Health

Instituto

Nacional de

Enfermedades

Respiratorias

Department of

Infectious

Diseases,

Istituto

Superiore di

Sanità, Roma ,

Italy

Department of

Infectious

Diseases,

Istituto

Superiore di

Sanità, Rome,

Italy

Centre for Infectious Diseases and Microbiology Laboratory Services

Seattle Flu Study

Instituto de Diagnostico y Referencia Epidemiologicos (INDRE)

Virology Laboratory,

Scientific

Department, Army

Medical Center

Virology Laboratory,

Scientific

Department, Army

Medical Center

NSW Health

Pathology - Institute

of Clinical Pathology

and Medical

Research;

Westmead Hospital;

University of Sydney

Helen Chu, Michael Boeckh, Janet Englund, Michael Famulare, Barry Lutz, Deborah Nickerson, Mark Rieder, Lea Starita, Matthew Thompson, Jay Shendure, and Trevor Bedford

Ramirez-Gonzalez Ernesto, Garces-Ayala Fabiola, Araiza-Rodriguez Adnan, Mendieta-Condado Edgar, Rodriguez-Maldonado Abril, Wong-Arambula Claudia, Vazquez-Perez Joel, Martinez Arturo, Boukadida Celia, Munoz-Medina Esteban, Sanchez Alejandro, Isa Pavel, Taboada Blanca, Lopez Susana, Arias Carlos, Barrera-Badillo Gisela, Hernandez-Rivas Lucia, Lopez-Martinez Irma

Paola Stefanelli, Stefano Fiore, Antonella Marchi, Eleonora Benedetti, Concetta Fabiani, Giovanni Faggioni, Antonella Fortunato, Riccardo De Santis, Silvia Fillo, Anna Anselmo, Andrea Ciammaruconi, Stefano Palomba, Florigio Lista

Paola Stefanelli, Stefano Fiore, Antonella Marchi, Eleonora Benedetti, Concetta Fabiani, Giovanni Faggioni, Antonella Fortunato, Silvia Fillo, Riccardo De Santis, Andrea Ciammaruconi, Giancarlo Petralito, Filippo Molinari, Florigio Lista

Eden J-S, Carter I, Rahman H, Holmes EC, Rockett R, O’Sullivan MV, Sintchenko V, Chen SC, Maddocks S, Kok J and Dwyer DE for the 2019-nCoV Study Group

EPI_ISL_412978

hCoV-19/Wuhan/HBCDC-HB-02/2020

Asia / China / Hubei / Wuhan

2020-01-17

| The Central |  | Hubei Provincial | Bin Fang, Xiang Li, Xiao Yu, Linlin Liu, Bo Yang, Faxian |  |
| --- | --- | --- | --- | --- |
|  |  | Center for Disease |  |  |
| Hospital Of |  |  | Zhan, Guojun Ye, Xixiang Huo, Junqiang Xu, Bo Yu, Kun Cai, |  |
|  |  | Control and |  |  |
| Wuhan |  |  | Jing Li, Yongzhong Jiang. |  |
|  |  | Prevention |  |  |
|  |  |  |  |  |
|  |  |  |  |  |

EPI_ISL_412979

EPI_ISL_412980

EPI_ISL_412981

EPI_ISL_412982

EPI_ISL_412983

EPI_ISL_413602

hCoV-19/Wuhan/HBCDC-HB-03/2020

hCoV-19/Wuhan/HBCDC-HB-04/2020

hCoV-19/Wuhan/HBCDC-HB-05/2020

hCoV-19/Wuhan/HBCDC-HB-06/2020

hCoV-19/Tianmen/HBCDC-

HB-07/2020

hCoV-

19/Finland/FIN03032020A/202

0

Asia / China / Hubei / Wuhan

Asia / China / Hubei / Wuhan

Asia / China / Hubei / Wuhan

Asia / China / Hubei / Wuhan

Asia / China / Hubei / Tianmen

Europe /

Finland /

Helsinki

|  | Union Hospital |  |  |  |  |
| --- | --- | --- | --- | --- | --- |
|  | of Tongji |  | Hubei Provincial |  |  |
|  | Medical College, |  |  | Bin Fang, Xiang Li, Xiao Yu, Linlin Liu, Bo Yang, Faxian |  |
|  |  |  | Center for Disease |  |  |
| 2020-01-18 | Huazhong |  |  | Zhan, Guojun Ye, Xixiang Huo, Junqiang Xu, Bo Yu, Kun Cai, |  |
|  |  |  | Control and |  |  |
|  | University of |  |  | Jing Li, Yongzhong Jiang. |  |
|  |  |  | Prevention |  |  |
|  | Science and |  |  |  |  |
|  |  |  |  |  |  |
|  | Technology |  |  |  |  |
|  |  |  |  |  |  |
|  | Union Hospital |  |  |  |  |
|  | of Tongji |  | Hubei Provincial |  |  |
|  | Medical College, |  |  | Bin Fang, Xiang Li, Xiao Yu, Linlin Liu, Bo Yang, Faxian |  |
|  |  |  | Center for Disease |  |  |
| 2020-01-18 | Huazhong |  |  | Zhan, Guojun Ye, Xixiang Huo, Junqiang Xu, Bo Yu, Kun Cai, |  |
|  |  |  | Control and |  |  |
|  | University of |  |  | Jing Li, Yongzhong Jiang. |  |
|  |  |  | Prevention |  |  |
|  | Science and |  |  |  |  |
|  |  |  |  |  |  |
|  | Technology |  |  |  |  |
|  |  |  |  |  |  |
|  | CR&WISCO |  | Hubei Provincial | Bin Fang, Xiang Li, Xiao Yu, Linlin Liu, Bo Yang, Faxian |  |
|  |  |  | Center for Disease |  |  |
| 2020-01-18 | GENERAL |  |  | Zhan, Guojun Ye, Xixiang Huo, Junqiang Xu, Bo Yu, Kun Cai, |  |
|  |  |  | Control and |  |  |
|  | HOSPITAL |  |  | Jing Li, Yongzhong Jiang. |  |
|  |  |  | Prevention |  |  |
|  |  |  |  |  |  |
|  |  |  |  |  |  |
|  |  |  | Hubei Provincial | Bin Fang, Xiang Li, Xiao Yu, Linlin Liu, Bo Yang, Faxian |  |
| 2020-02-07 | Wuhan Lung |  | Center for Disease |  |  |
|  |  |  |  | Zhan, Guojun Ye, Xixiang Huo, Junqiang Xu, Bo Yu, Kun Cai, |  |
|  | Hospital |  | Control and |  |  |
|  |  |  |  | Jing Li, Yongzhong Jiang. |  |
|  |  |  | Prevention |  |  |
|  |  |  |  |  |  |
|  | Tianmen Center |  | Hubei Provincial | Bin Fang, Xiang Li, Xiao Yu, Linlin Liu, Bo Yang, Faxian |  |
|  | for Disease |  | Center for Disease |  |  |
| 2020-02-08 |  |  |  | Zhan, Guojun Ye, Xixiang Huo, Junqiang Xu, Bo Yu, Kun Cai, |  |
|  | Control and |  | Control and |  |  |
|  |  |  |  | Jing Li, YiFa Zhu, Yangyang Tao,Xierong Li,Yongzhong Jiang. |  |
|  | Prevention |  | Prevention |  |  |
|  |  |  |  |  |  |
|  |  |  |  |  |  |
|  | Department of |  |  |  |  |
|  | Virology and |  |  |  |  |
|  | Immunology, |  | Department of |  |  |
| 2020-03-03 | University of |  | Virology, Faculty of | Teemu Smura, Hannimari Kallio-Kokko, Olli Vapalahti |  |
|  | Helsinki and |  | Medicine, University |  |  |
|  | Helsinki |  | of Helsinki, Helsinki, |  |  |
|  | University |  | Finland |  |  |
|  | Hospital, Huslab |  |  |  |  |
|  | Finland |  |  |  |  |
|  |  |  |  |  |  |


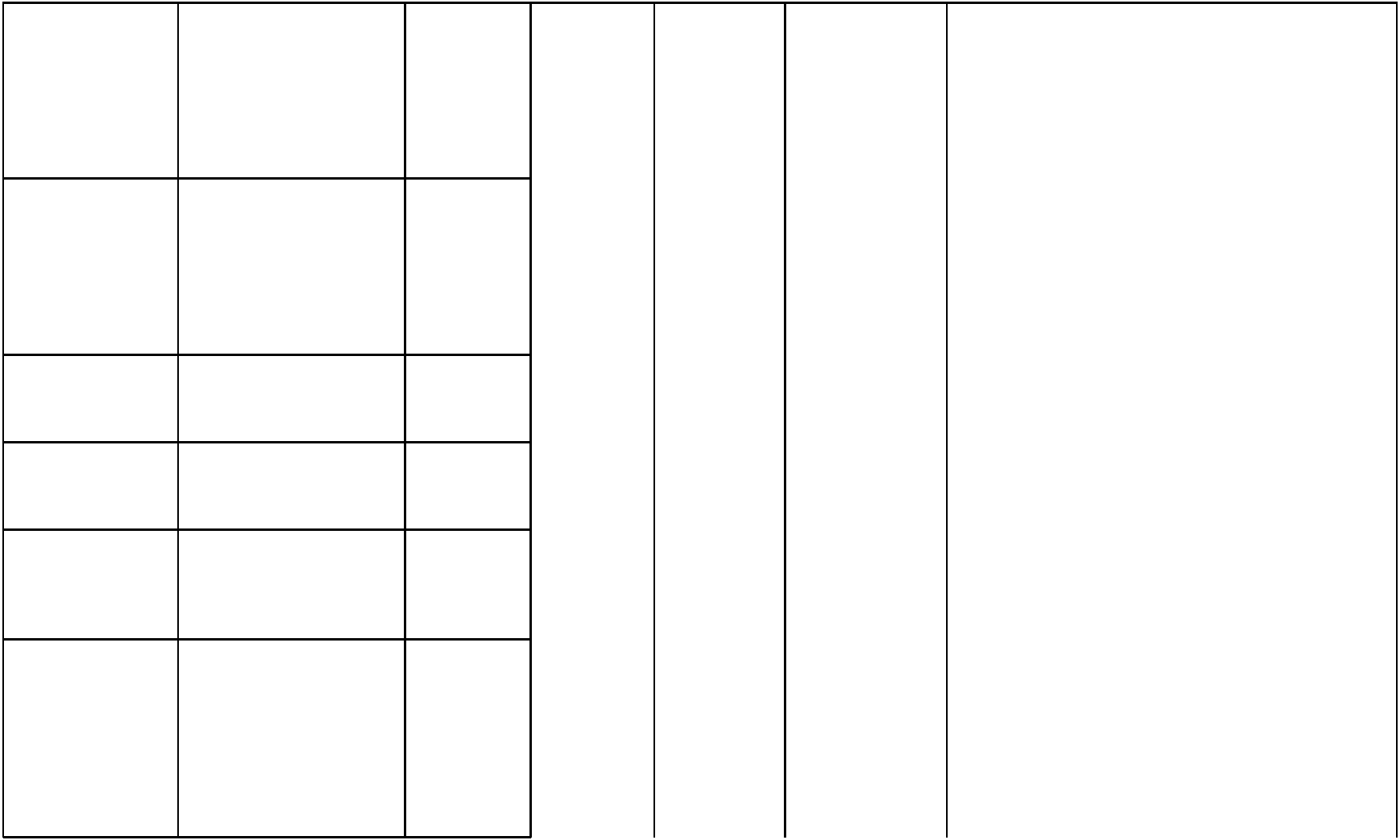


EPI_ISL_413603

EPI_ISL_413604

| hCoV- |  | Europe / |
| --- | --- | --- |
| 19/Finland/FIN03032020B/202 |  | Finland / |
| 0 |  | Helsinki |
|  |  |  |
| hCoV- |  | Europe / |
| 19/Finland/FIN03032020C/202 |  | Finland / |
| 0 |  | Helsinki |

|  | Department of |  |  |  |  |
| --- | --- | --- | --- | --- | --- |
|  | Virology and |  |  |  |  |
|  | Immunology, |  | Department of |  |  |
|  | University of |  | Virology, Faculty of |  |  |
| 2020-03-03 | Helsinki and |  | Medicine, University | Teemu Smura, Hannimari Kallio-Kokko, Olli Vapalahti |  |
|  | Helsinki |  | of Helsinki, Helsinki, |  |  |
|  | University |  | Finland |  |  |
|  | Hospital, Huslab |  |  |  |  |
|  | Finland |  |  |  |  |
|  |  |  |  |  |  |
|  | Department of |  |  |  |  |
|  | Virology and |  | Department of |  |  |
|  | Immunology, |  |  |  |  |
| 2020-03-03 | University of |  | Virology, Faculty of | Teemu Smura, Hannimari Kallio-Kokko, Olli Vapalahti |  |
|  | Helsinki and |  | Medicine, University |  |  |
|  | Helsinki |  | of Helsinki, Helsinki, |  |  |
|  | University |  | Finland |  |  |
|  | Hospital, Huslab |  |  |  |  |
|  | Finland |  |  |  |  |
|  |  |  |  |  |  |


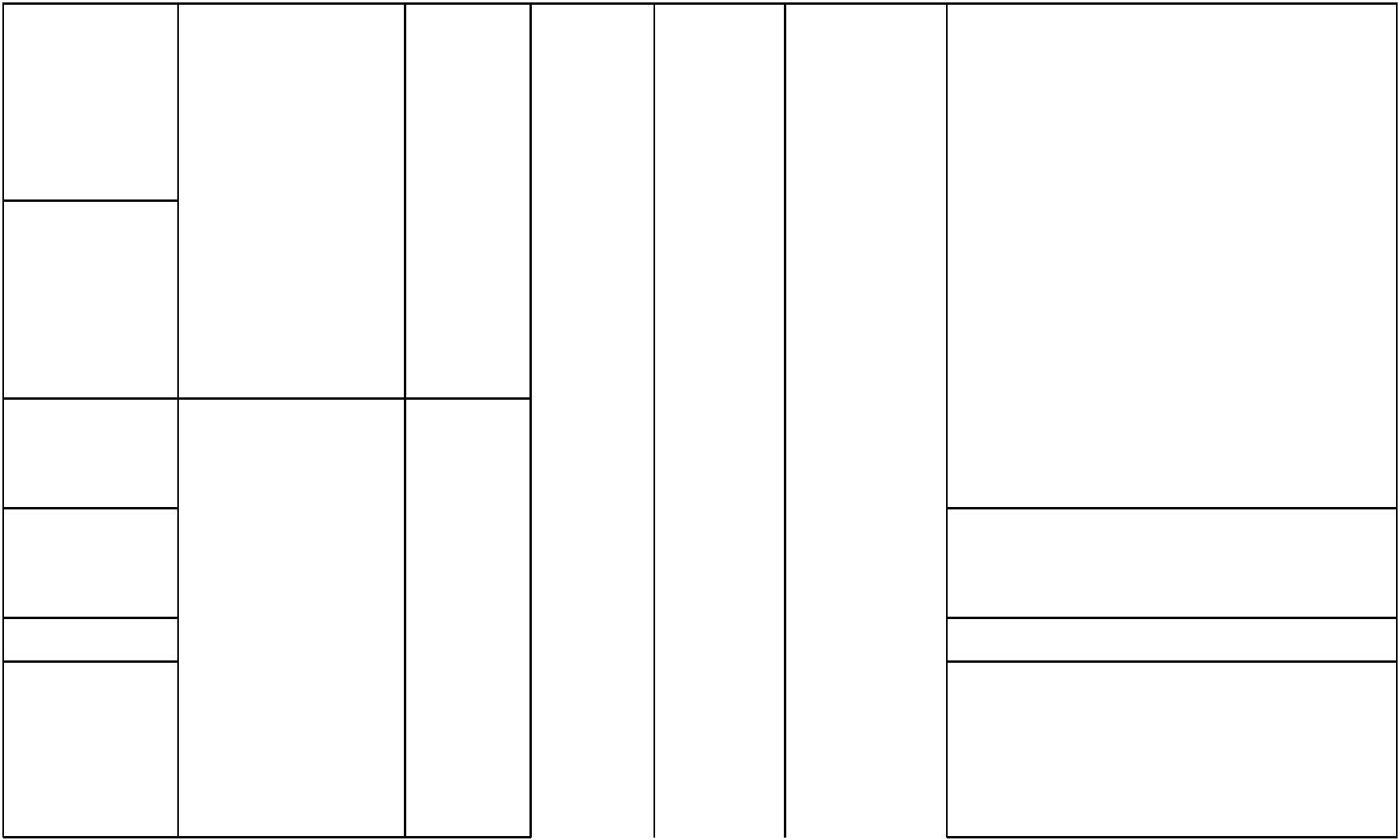


EPI_ISL_413647

EPI_ISL_413648

EPI_ISL_414619

EPI_ISL_414623

| hCoV-19/Portugal/CV62/2020 |  | Europe / |  |
| --- | --- | --- | --- |
|  |  | Portugal |  |
|  |  |  |  |
|  |  |  |  |
| hCoV-19/Portugal/CV63/2020 |  | Europe / |  |
|  |  | Portugal |  |
|  |  |  |  |
|  |  |  |  |
| hCoV-19/USA/WA- |  | North America / |  |
| UW32/2020 |  | USA |  |

Europe /

hCoV-19/France/GE1583/2020 France / Grand-

Est / Strasbourg

|  | Centro Hospital |  |  |  |
| --- | --- | --- | --- | --- |
| 2020-03-01 | do Porto, E.P.E. Instituto Nacional de | | |  |
|  | - H. Geral de |  | Saude (INSA) |  |
|  |  |  |  |  |
|  | Santo Antonio |  |  |  |
|  |  |  |  |  |
|  | Centro |  |  |  |
| 2020-03-01 | Hospitalar e |  | Instituto Nacional de |  |
|  | Universitário de |  | Saude (INSA) |  |
|  |  |  |  |  |
|  | Sao Joao, Porto |  |  |  |
|  |  |  |  |  |
| 2020-03-07 | UW Virology Lab |  | UW Virology Lab |  |
|  |  |  |  |  |
|  | Laboratoire de |  |  |  |
|  | Virologie Institut |  | National Reference |  |
|  | de Virologie -Center for Viruses of | | |  |
| 2020-02-25 | INSERM U 1109 |  | Respiratory |  |
|  | Hôpitaux |  | Infections, Institut |  |
|  | Universitaires |  | Pasteur, Paris |  |
|  | de Strasbourg |  |  |  |
|  |  |  |  |  |

Raquel Guiomar, Inês Costa, Pedro Pechirra, Joana Mendonça, Luís Vieira, Helena Ramos, Joana Isidro, Vítor Borges, João Paulo Gomes

Raquel Guiomar, Inês Costa, Pedro Pechirra, Joana Mendonça, Luís Vieira, João Tiago Guimarães, Joana Isidro, Vítor Borges, João Paulo Gomes

Pavitra Roychoudhury, Hong Xie, Keith Jerome, Alexander Greninger

Mélnie Albert, Marion Barbet, Sylvie Behillil, Méline Bizard,

Angela Brisebarre, Flora Donati Vincent Enouf, Maud Vanpeene, Sylvie van der Werf, Samira Fafi-Kremer

EPI_ISL_414624

EPI_ISL_414625

EPI_ISL_414626

hCoV-19/France/N1620/2020

hCoV-19/France/PL1643/2020

hCoV-19/France/HF1684/2020

Europe / France / Normandie / Rouen

Europe / France / Pays de la Loire / Nantes

Europe / France / Hauts

de France / Crépy en Valois

|  | Centre Hositalier |  | National Reference |  |  |
| --- | --- | --- | --- | --- | --- |
|  | Universitaire de |  | Center for Viruses of | Mélnie Albert, Marion Barbet, Sylvie Behillil, Méline Bizard, |  |
| 2020-02-26 | Rouen |  | Respiratory | Angela Brisebarre, Flora Donati Vincent Enouf, Maud |  |
|  | Laboratoire de |  | Infections, Institut | Vanpeene, Sylvie van der Werf, Jean-Christophe Plantier |  |
|  | Virologie |  | Pasteur, Paris |  |  |
|  |  |  |  |  |  |
|  | Centre |  |  |  |  |
|  | Hospitalier |  | National Reference |  |  |
| 2020-02-26 | Régional |  | Center for Viruses of | Mélnie Albert, Marion Barbet, Sylvie Behillil, Méline Bizard, |  |
|  | Universitaire de |  | Respiratory | Angela Brisebarre, Flora Donati Vincent Enouf, Maud |  |
|  | Nantes |  | Infections, Institut | Vanpeene, Sylvie van der Werf, Marianne Coste-Burel |  |
|  | Laboratoire de |  | Pasteur, Paris |  |  |
|  | Virologie |  |  |  |  |
|  |  |  |  |  |  |
|  |  |  | National Reference |  |  |
|  |  |  | Center for Viruses of | Mélnie Albert, Marion Barbet, Sylvie Behillil, Méline Bizard, |  |
| 2020-02-29 | unknown |  | Respiratory | Angela Brisebarre, Flora Donati Vincent Enouf, Maud |  |
|  |  |  | Infections, Institut | Vanpeene, Sylvie van der Werf |  |
|  |  |  | Pasteur, Paris |  |  |


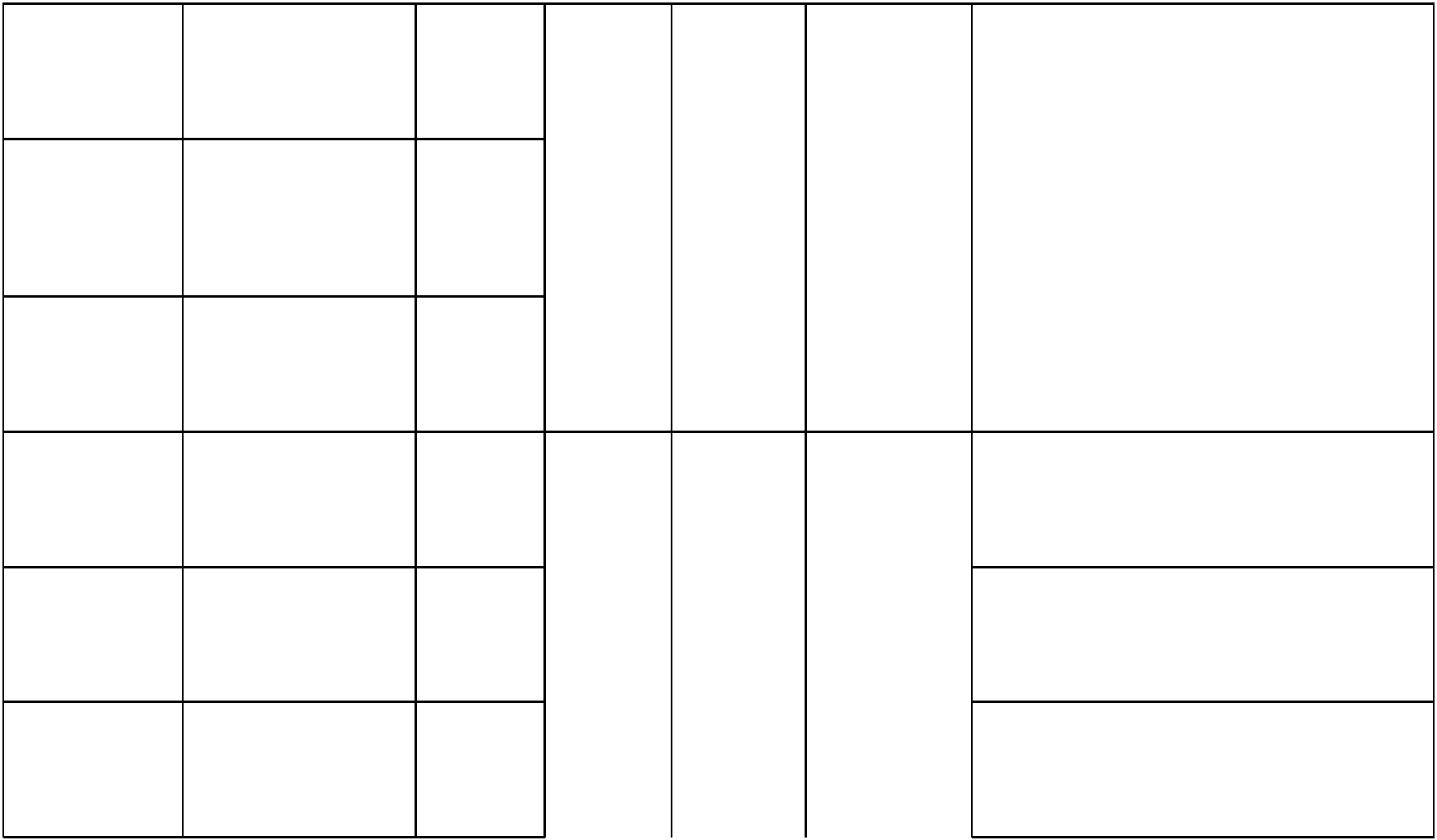


EPI_ISL_414627

EPI_ISL_414630

EPI_ISL_414631

hCoV-19/France/HF1795/2020

hCoV-19/France/HF1871/2020

hCoV-19/France/GE1973/2020

Europe / France / Hauts de France / Compiègne

Europe / France / Hauts de France / Compiègne

Europe /

France / Grand-

Est / Reims

|  | Centre |  | National Reference |  |
| --- | --- | --- | --- | --- |
|  | Hospitalier |  | Center for Viruses of |  |
| 2020-03-02 | Compiègne |  | Respiratory |  |
|  | Laboratoire de |  | Infections, Institut |  |
|  | Biologie |  | Pasteur, Paris |  |
|  |  |  |  |  |
|  | Centre |  | National Reference |  |
|  | Hospitalier |  | Center for Viruses of |  |
| 2020-03-03 | Compiègne |  | Respiratory |  |
|  | Laboratoire de |  | Infections, Institut |  |
|  | Biologie |  | Pasteur, Paris |  |
|  |  |  |  |  |
|  | Hôpital Robert |  | National Reference |  |
|  |  |  | Center for Viruses of |  |
|  | Debré |  |  |  |
| 2020-03-04 |  |  | Respiratory |  |
|  | Laboratoire de |  |  |  |
|  |  |  | Infections, Institut |  |
|  | Virologie |  |  |  |
|  |  |  | Pasteur, Paris |  |
|  |  |  |  |  |
|  |  |  |  |  |

Mélnie Albert, Marion Barbet, Sylvie Behillil, Méline Bizard, Angela Brisebarre, Flora Donati Vincent Enouf, Maud Vanpeene, Sylvie van der Werf, Raulin Olivia

Mélnie Albert, Marion Barbet, Sylvie Behillil, Méline Bizard, Angela Brisebarre, Flora Donati Vincent Enouf, Maud Vanpeene, Sylvie van der Werf, Raulin Olivia

Mélnie Albert, Marion Barbet, Sylvie Behillil, Méline Bizard,

Angela Brisebarre, Flora Donati Vincent Enouf, Maud

Vanpeene, Sylvie van der Werf, Laurent Andreoletti

EPI_ISL_414632

EPI_ISL_414635

EPI_ISL_414637

EPI_ISL_414638

EPI_ISL_414641

EPI_ISL_414642

|  |  |  |  |  | Hôpital Robert |  | National Reference |  |  |
| --- | --- | --- | --- | --- | --- | --- | --- | --- | --- |
|  |  | Europe / |  |  |  |  | Center for Viruses of | Mélnie Albert, Marion Barbet, Sylvie Behillil, Méline Bizard, |  |
|  |  |  |  |  | Debré |  |  |  |  |
| hCoV-19/France/GE1977/2020 |  | France / Grand- | 2020-03-04 | |  |  | Respiratory | Angela Brisebarre, Flora Donati Vincent Enouf, Maud |  |
|  |  |  |  |  | Laboratoire de |  |  |  |  |
|  |  | Est / Reims |  |  |  |  | Infections, Institut | Vanpeene, Sylvie van der Werf, Laurent Andreoletti |  |
|  |  |  |  |  | Virologie |  |  |  |  |
|  |  |  |  |  |  |  | Pasteur, Paris |  |  |
|  |  |  |  |  |  |  |  |  |  |
|  |  |  |  |  |  |  |  |  |  |
|  |  | Europe / |  |  | Centre |  | National Reference |  |  |
|  |  |  |  |  | Hospitalier |  | Center for Viruses of | Mélnie Albert, Marion Barbet, Sylvie Behillil, Méline Bizard, |  |
|  |  | France / Hauts |  |  |  |  |  |  |  |
| hCoV-19/France/HF1988/2020 |  |  | 2020-03-04 | | Compiègne |  | Respiratory | Angela Brisebarre, Flora Donati Vincent Enouf, Maud |  |
|  |  | de France / |  |  | Laboratoire de |  | Infections, Institut | Vanpeene, Sylvie van der Werf, Raulin Olivia |  |
|  |  | Compiègne |  |  |  |  |  |  |  |
|  |  |  |  |  | Biologie |  | Pasteur, Paris |  |  |
|  |  |  |  |  |  |  |  |  |  |
|  |  |  |  |  |  |  |  |  |  |
|  |  | Europe / |  |  | Centre |  | National Reference | Mélnie Albert, Marion Barbet, Sylvie Behillil, Méline Bizard, |  |
|  |  |  |  |  | Hospitalier |  | Center for Viruses of |  |  |
|  |  | France / Hauts |  |  |  |  |  |  |  |
| hCoV-19/France/HF1993/2020 |  |  | 2020-03-04 | | Compiègne |  | Respiratory | Angela Brisebarre, Flora Donati Vincent Enouf, Maud |  |
|  |  | de France / |  |  | Laboratoire de |  | Infections, Institut | Vanpeene, Sylvie van der Werf, Raulin Olivia |  |
|  |  | Compiègne |  |  |  |  |  |  |  |
|  |  |  |  |  | Biologie |  | Pasteur, Paris |  |  |
|  |  |  |  |  |  |  |  |  |  |
|  |  |  |  |  |  |  |  |  |  |
|  |  | Europe / |  |  | Centre |  | National Reference | Mélnie Albert, Marion Barbet, Sylvie Behillil, Méline Bizard, |  |
|  |  |  |  |  | Hospitalier |  | Center for Viruses of |  |  |
|  |  | France / Hauts |  |  |  |  |  |  |  |
| hCoV-19/France/HF1995/2020 |  |  | 2020-03-04 | | Compiègne |  | Respiratory | Angela Brisebarre, Flora Donati Vincent Enouf, Maud |  |
|  |  | de France / |  |  | Laboratoire de |  | Infections, Institut | Vanpeene, Sylvie van der Werf, Raulin Olivia |  |
|  |  | Compiègne |  |  |  |  |  |  |  |
|  |  |  |  |  | Biologie |  | Pasteur, Paris |  |  |
|  |  |  |  |  |  |  |  |  |  |
|  |  |  |  |  |  |  |  |  |  |
|  |  |  |  |  | Department of |  |  |  |  |
|  |  |  |  |  | Virology and |  |  |  |  |
|  |  |  |  |  | Immunology, |  | Department of |  |  |
| hCoV-19/Finland/FIN- |  |  |  |  | University of |  | Virology, Faculty of |  |  |
|  |  | Europe / Finland | 2020-03-05 | | Helsinki and |  | Medicine, University | Teemu Smura, Hannimari Kallio-Kokko, Olli Vapalahti |  |
| 313/2020 |  |  |  |  |  |  |  |  |  |
|  |  |  |  |  | Helsinki |  | of Helsinki, Helsinki, |  |  |
|  |  |  |  |  |  |  |  |  |  |
|  |  |  |  |  | University |  | Finland |  |  |
|  |  |  |  |  | Hospital, Huslab |  |  |  |  |
|  |  |  |  |  | Finland |  |  |  |  |
|  |  |  |  |  |  |  |  |  |  |
|  |  |  |  |  | Department of |  |  |  |  |
|  |  |  |  |  | Virology and |  |  |  |  |
|  |  |  |  |  | Immunology, |  | Department of |  |  |
| hCoV-19/Finland/FIN- |  |  |  |  | University of |  | Virology, Faculty of |  |  |
|  |  | Europe / Finland | 2020-03-08 | | Helsinki and |  | Medicine, University | Teemu Smura, Hannimari Kallio-Kokko, Olli Vapalahti |  |
| 455/2020 |  |  |  |  |  |  |  |  |  |
|  |  |  |  |  | Helsinki |  | of Helsinki, Helsinki, |  |  |
|  |  |  |  |  |  |  |  |  |  |
|  |  |  |  |  | University |  | Finland |  |  |
|  |  |  |  |  | Hospital, Huslab |  |  |  |  |
|  |  |  |  |  | Finland |  |  |  |  |
|  |  |  |  |  |  |  |  |  |  |


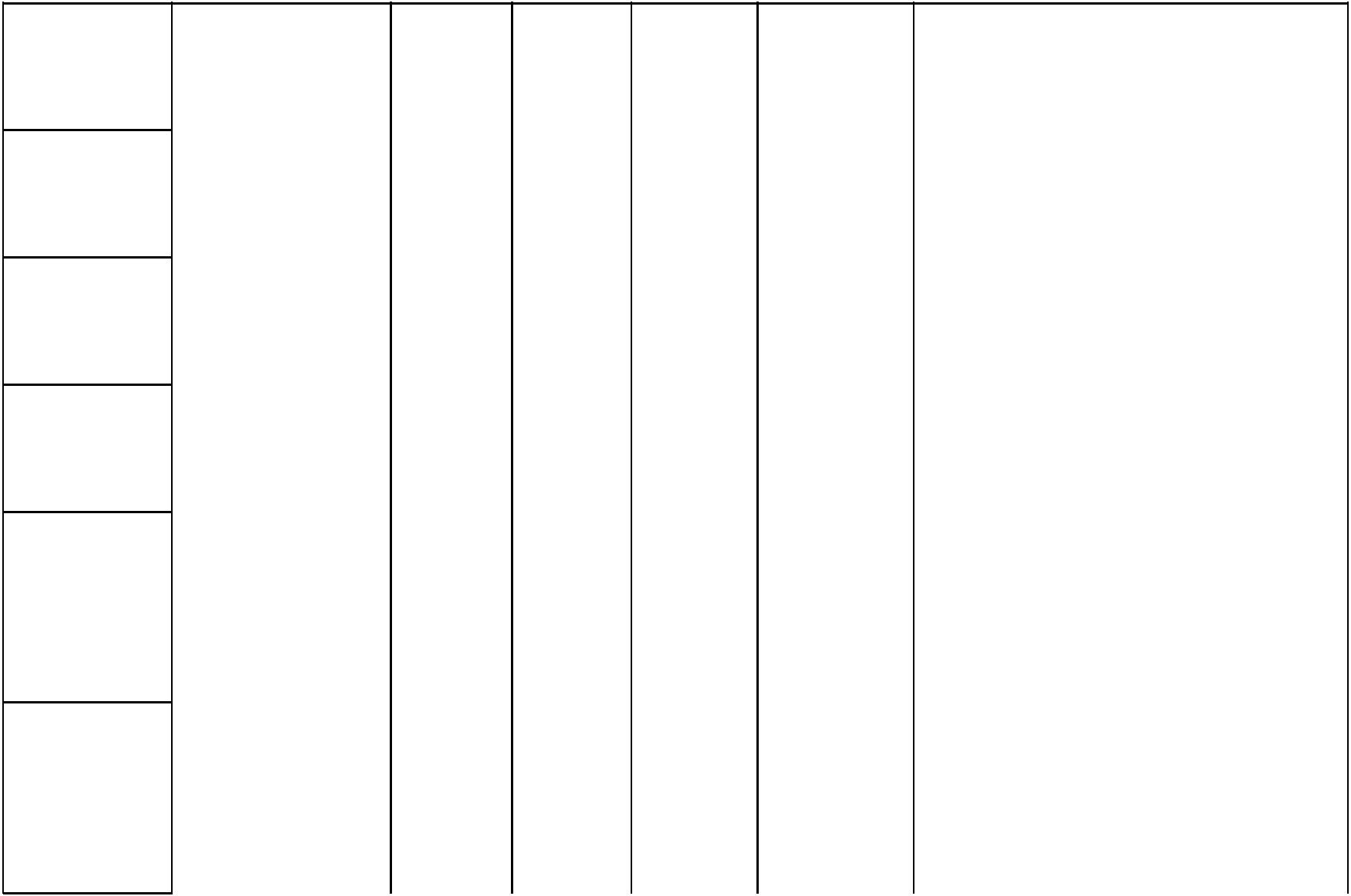


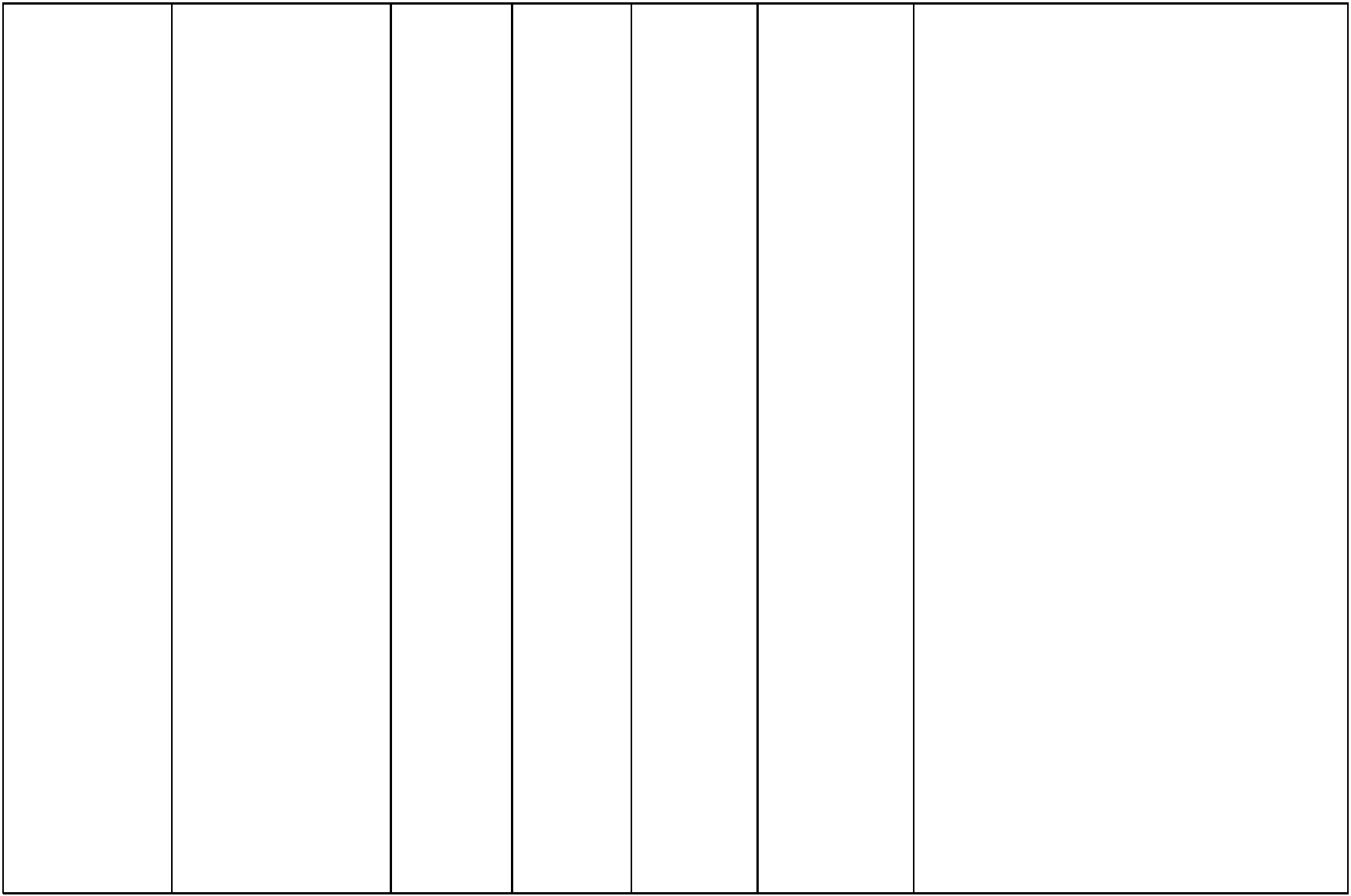


|  |  |  |  |  |  |  | Department of |  |  |  |  |
| --- | --- | --- | --- | --- | --- | --- | --- | --- | --- | --- | --- |
|  |  |  |  |  |  |  | Virology and |  |  |  |  |
|  |  |  |  |  |  |  | Immunology, |  | Department of |  |  |
|  |  | hCoV-19/Finland/FIN- |  |  |  |  | University of |  | Virology, Faculty of |  |  |
| EPI_ISL_414643 |  |  |  | Europe / Finland | 2020-03-07 | | Helsinki and |  | Medicine, University | Teemu Smura, Hannimari Kallio-Kokko, Olli Vapalahti |  |
|  | 508/2020 | |  |  |  |  |  |  |  |  |  |
|  |  |  |  |  |  |  | Helsinki |  | of Helsinki, Helsinki, |  |  |
|  |  |  |  |  |  |  |  |  |  |  |  |
|  |  |  |  |  |  |  | University |  | Finland |  |  |
|  |  |  |  |  |  |  | Hospital, Huslab |  |  |  |  |
|  |  |  |  |  |  |  | Finland |  |  |  |  |
|  |  |  |  |  |  |  |  |  |  |  |  |
|  |  |  |  |  |  |  | Department of |  |  |  |  |
|  |  |  |  |  |  |  | Virology and |  | Department of |  |  |
|  |  |  |  |  |  |  | Immunology, |  |  |  |  |
| EPI_ISL_414646 |  | hCoV-19/Finland/FIN- |  | Europe / Finland | 2020-03-04 | | University of |  | Virology, Faculty of | Teemu Smura, Hannimari Kallio-Kokko, Olli Vapalahti |  |
|  |  |  |  |  |  |  | Helsinki and |  | Medicine, University |  |  |
|  | 266/2020 | |  |  |  |  |  |  |  |  |  |
|  |  |  |  |  |  |  | Helsinki |  | of Helsinki, Helsinki, |  |  |
|  |  |  |  |  |  |  |  |  |  |  |  |
|  |  |  |  |  |  |  | University |  | Finland |  |  |
|  |  |  |  |  |  |  | Hospital, Huslab |  |  |  |  |
|  |  |  |  |  |  |  | Finland |  |  |  |  |
|  |  |  |  |  |  |  |  |  |  |  |  |
|  |  |  |  | North America / |  |  | Andersen Lab, |  | Andersen Lab, The | Mark Zeller, Catie Anderson, Emily Spender, Sarah Topol, |  |
|  |  | hCoV-19/USA/CA- |  | USA / California |  |  | The Scripps |  |  |  |  |
| EPI_ISL_414648 |  |  |  |  | 2020-03-11 | |  |  | Scripps Research | Raphaelle Klitting, Refugio Robles-Sikisaka, Karthik |  |
|  |  | PC101P/2020 |  | / San Diego |  |  | Research |  |  |  |  |
|  |  |  |  |  |  |  |  |  | Institute | Gangavarapu, Laura Nicholson, Kristian Andersen |  |
|  |  |  |  | County |  |  | Institute |  |  |  |  |
|  |  |  |  |  |  |  |  |  |  |  |  |
|  |  |  |  |  |  |  |  |  |  |  |  |
|  |  |  |  |  |  |  | State Key |  |  |  |  |
|  |  |  |  |  |  |  | Laboratory of |  |  |  |  |
|  |  |  |  |  |  |  | Respiratory |  |  |  |  |
|  |  |  |  |  |  |  | Disease, |  |  |  |  |
|  |  |  |  |  |  |  | National Clinical |  |  |  |  |
|  |  |  |  |  |  |  | Research |  |  |  |  |
|  |  |  |  |  |  |  | Center for |  | The First Affiliated |  |  |
|  |  | hCoV- |  | Asia / China / |  |  | Respiratory |  |  |  |  |
|  |  |  |  |  |  |  |  |  | Hospital of |  |  |
|  |  |  |  |  |  |  | Disease, |  |  |  |  |
| EPI_ISL_414686 |  | 19/Guangzhou/GZMU0030/20 |  | Guangdong / | 2020-02-27 | |  |  | Guangzhou Medical | Zhao et al |  |
|  |  |  |  |  |  |  | Guangzhou |  |  |  |  |
|  | 20 | |  | Guangzhou |  |  |  |  | University & BGI- |  |  |
|  |  |  |  |  |  |  | Institute of |  |  |  |  |
|  |  |  |  |  |  |  |  |  | Shenzhen |  |  |
|  |  |  |  |  |  |  | Respiratory |  |  |  |  |
|  |  |  |  |  |  |  |  |  |  |  |  |

Health, the First

Affiliated

Hospital of

Guangzhou

Medical

University

|  | hCoV- | Asia / China / |  |
| --- | --- | --- | --- |
| EPI_ISL_414689 | 19/Guangzhou/GZMU0044/20 | Guangdong / | 2020-02-25 |
|  | 20 | Guangzhou |  |

|  |  | hCoV- |  | Asia / China / |  |  |
| --- | --- | --- | --- | --- | --- | --- |
| EPI_ISL_414691 |  | 19/Guangzhou/GZMU0048/20 |  | Guangdong / | 2020-02-25 | |
|  | 20 | |  | Guangzhou |  |  |
|  |  |  |  |  |  |  |

State Key Laboratory of Respiratory Disease, National Clinical


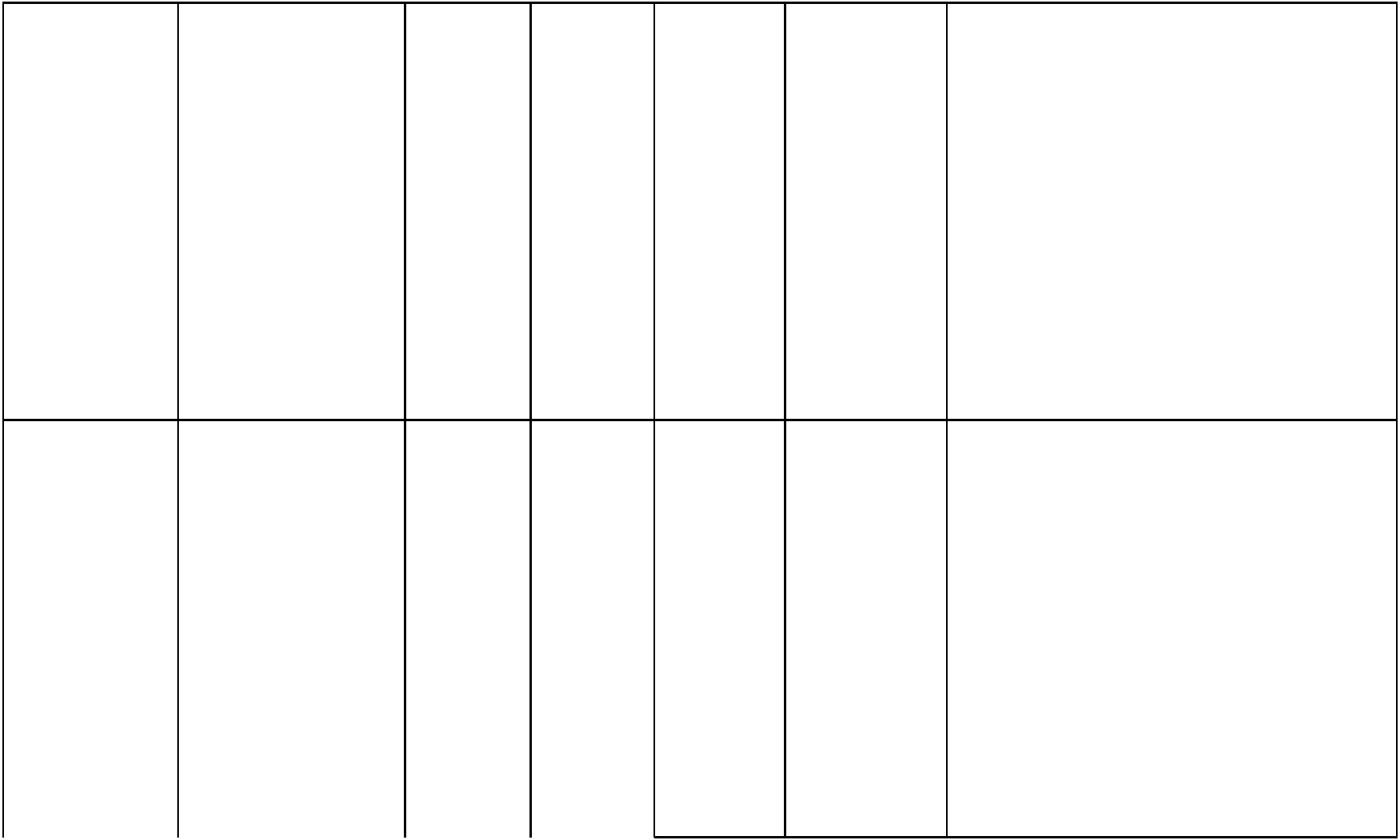


Research

Center for

Respiratory

Disease,

Guangzhou

Institute of

Respiratory

Health, the First

Affiliated

Hospital of

Guangzhou

Medical

University

State Key Laboratory of Respiratory Disease, National Clinical

Research

Center for

Respiratory

Disease,

Guangzhou

Institute of

Respiratory

Health, the First

Affiliated

Hospital of

Guangzhou

Medical

University

The First Affiliated

Hospital of

Guangzhou Medical

University & BGI-

Shenzhen

The First Affiliated

Hospital of

Guangzhou Medical

University & BGI-

Shenzhen

Zhao et al

Zhao et al


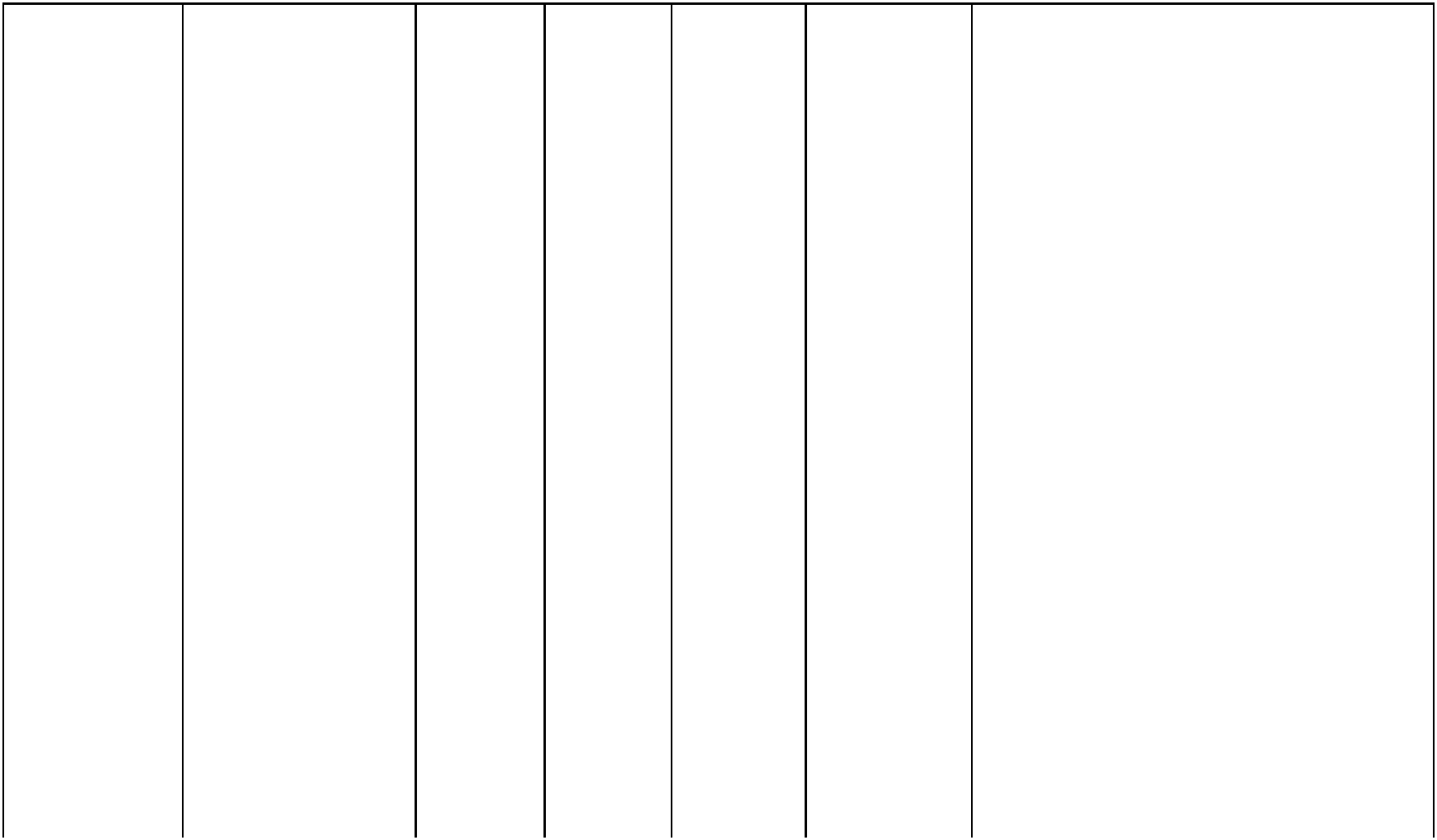


|  |  |  |  |  |  |  | State Key |  |  |  |  |
| --- | --- | --- | --- | --- | --- | --- | --- | --- | --- | --- | --- |
|  |  |  |  |  |  |  | Laboratory of |  |  |  |  |
|  |  |  |  |  |  |  | Respiratory |  |  |  |  |
|  |  |  |  |  |  |  | Disease, |  |  |  |  |
|  |  |  |  |  |  |  | National Clinical |  |  |  |  |
|  |  |  |  |  |  |  | Research |  |  |  |  |
|  |  |  |  |  |  |  | Center for |  | The First Affiliated |  |  |
|  |  |  |  |  |  |  | Respiratory |  |  |  |  |
|  |  | hCoV- |  | Asia / China / |  |  |  |  | Hospital of |  |  |
|  |  |  |  |  |  |  | Disease, |  |  |  |  |
| EPI_ISL_414692 |  | 19/Guangzhou/GZMU0014/20 |  | Guangdong / | 2020-02-25 | |  |  | Guangzhou Medical | Zhao et al |  |
|  |  |  |  |  |  |  | Guangzhou |  |  |  |  |
|  | 20 | |  | Guangzhou |  |  |  |  | University & BGI- |  |  |
|  |  |  |  |  |  |  | Institute of |  |  |  |  |
|  |  |  |  |  |  |  |  |  | Shenzhen |  |  |
|  |  |  |  |  |  |  | Respiratory |  |  |  |  |
|  |  |  |  |  |  |  |  |  |  |  |  |
|  |  |  |  |  |  |  | Health, the First |  |  |  |  |
|  |  |  |  |  |  |  | Affiliated |  |  |  |  |
|  |  |  |  |  |  |  | Hospital of |  |  |  |  |
|  |  |  |  |  |  |  | Guangzhou |  |  |  |  |
|  |  |  |  |  |  |  | Medical |  |  |  |  |
|  |  |  |  |  |  |  | University |  |  |  |  |
|  |  |  |  |  |  |  |  |  |  |  |  |
|  |  |  |  |  |  |  | Shandong |  |  | Xiao-Lin Jiang, Xiao-Li Zhang, Xiang-Na Zhao, Cun-Bao Li, |  |
|  |  | hCoV- |  | Asia / China / |  |  | Provincial |  | Beijing Institute of | Jie Lei, Zeng-Qiang Kou, Wen-Kui Sun, Yang Hang, Feng |  |
| EPI_ISL_414936 |  |  |  |  | 2020-01-23 | | Center for |  | Microbiology and | Gao, Sheng-Xiang Ji, Can-Fang Lin, Bo Pang, Ming-Xiao |  |
|  |  | 19/Shandong/LY003/2020 |  | Shandong |  |  |  |  |  |  |  |
|  |  |  |  |  |  |  | Disease Control |  | Epidemiology | Yao, Guo-Lin Wang, Lin Yao, Li-Jun Duan, Xiao Wei, Dian- |  |
|  |  |  |  |  |  |  |  |  |  |  |  |
|  |  |  |  |  |  |  | and Prevention |  |  | Ming Kang, Mai-Juan Ma |  |
|  |  |  |  |  |  |  |  |  |  |  |  |
|  |  |  |  |  |  |  | Shandong |  |  | Xiao-Lin Jiang, Xiao-Li Zhang, Xiang-Na Zhao, Cun-Bao Li, |  |
|  |  | hCoV- |  | Asia / China / |  |  | Provincial |  | Beijing Institute of | Jie Lei, Zeng-Qiang Kou, Wen-Kui Sun, Yang Hang, Feng |  |
| EPI_ISL_414937 |  |  |  |  | 2020-01-26 | | Center for |  | Microbiology and | Gao, Sheng-Xiang Ji, Can-Fang Lin, Bo Pang, Ming-Xiao |  |
|  |  | 19/Shandong/LY004/2020 |  | Shandong |  |  |  |  |  |  |  |
|  |  |  |  |  |  |  | Disease Control |  | Epidemiology | Yao, Guo-Lin Wang, Lin Yao, Li-Jun Duan, Xiao Wei, Dian- |  |
|  |  |  |  |  |  |  |  |  |  |  |  |
|  |  |  |  |  |  |  | and Prevention |  |  | Ming Kang, Mai-Juan Ma |  |
|  |  |  |  |  |  |  |  |  |  |  |  |
|  |  |  |  |  |  |  | Shandong |  | Beijing Institute of | Xiao-Lin Jiang, Xiao-Li Zhang, Xiang-Na Zhao, Cun-Bao Li, |  |
|  |  | hCoV- |  | Asia / China / |  |  | Provincial |  |  | Jie Lei, Zeng-Qiang Kou, Wen-Kui Sun, Yang Hang, Feng |  |
| EPI_ISL_414938 |  |  |  |  | 2020-01-24 | | Center for |  | Microbiology and | Gao, Sheng-Xiang Ji, Can-Fang Lin, Bo Pang, Ming-Xiao |  |
|  |  | 19/Shandong/LY005/2020 |  | Shandong |  |  |  |  |  |  |  |
|  |  |  |  |  |  |  | Disease Control |  | Epidemiology | Yao, Guo-Lin Wang, Lin Yao, Li-Jun Duan, Xiao Wei, Dian- |  |
|  |  |  |  |  |  |  |  |  |  |  |  |
|  |  |  |  |  |  |  | and Prevention |  |  | Ming Kang, Mai-Juan Ma |  |
|  |  |  |  |  |  |  |  |  |  |  |  |

EPI_ISL_414941

EPI_ISL_415129

EPI_ISL_415134

EPI_ISL_415136

EPI_ISL_415141

EPI_ISL_415152

EPI_ISL_415153

hCoV-

19/Shandong/LY008/2020

hCoV-

19/England/20099038206/202

0

hCoV-

19/England/20100004806/202

0

hCoV-

19/England/20100022706/202

0

hCoV-

19/England/20100121007/202

0

hCoV-

19/Panama/328677/2020

hCoV-19/Belgium/VLM-

03011/2020

Asia / China / Shandong

Europe / United Kingdom / England

Europe / United Kingdom / England

Europe / United Kingdom / England

Europe / United Kingdom / England

Central

America /

Panama /

Panama City

Europe /

Belgium /

Huldenberg

2020-01-30

2020-02-29

2020-02-29

2020-02-29

2020-02-29

2020-03-06

2020-03-03

Shandong


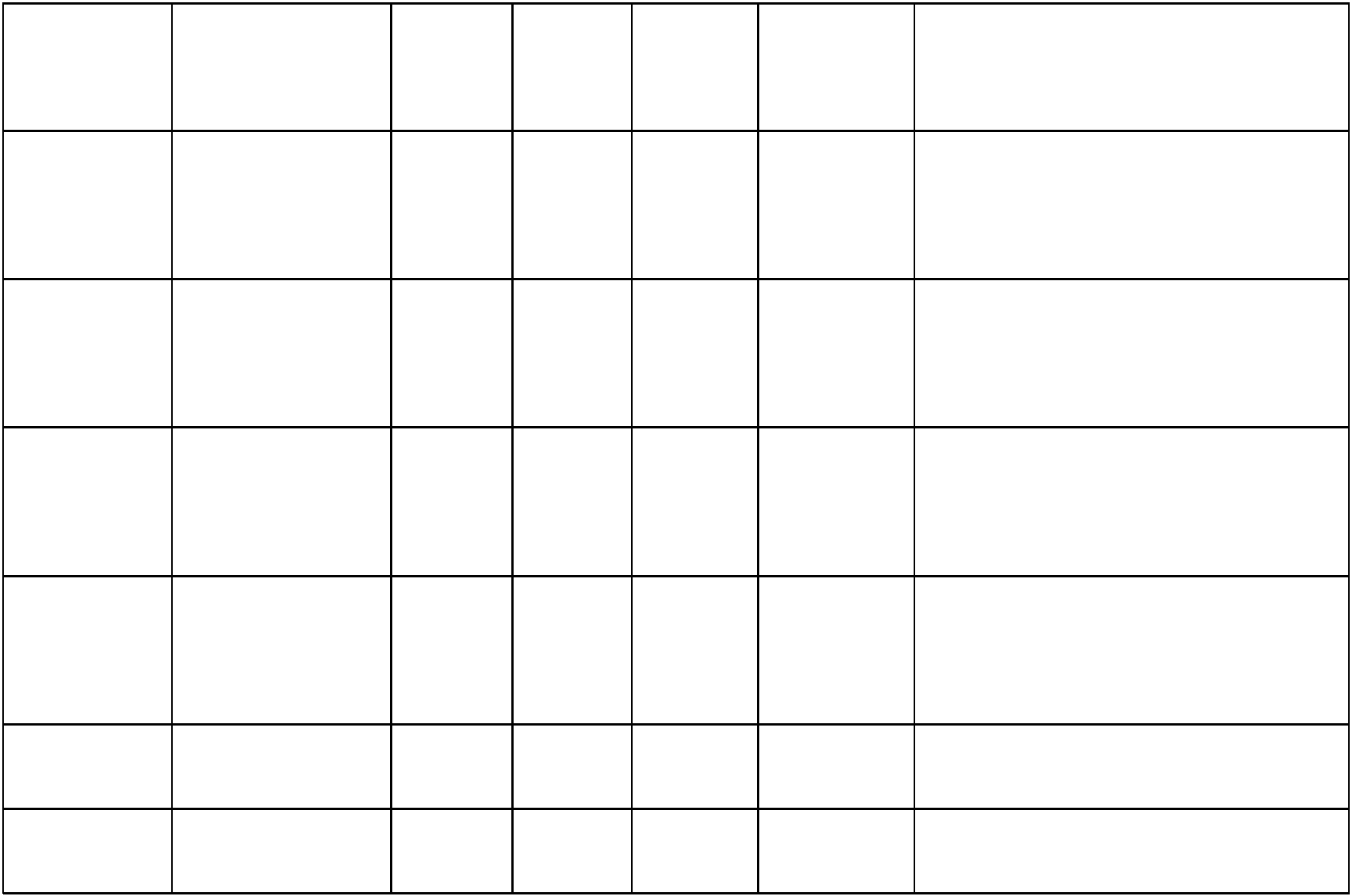


Provincial Center for Disease Control and Prevention

Respiratory Virus Unit, Microbiology Services Colindale, Public Health England

Respiratory Virus Unit, Microbiology Services Colindale, Public Health England

Respiratory Virus Unit, Microbiology Services Colindale, Public Health England

Respiratory Virus Unit, Microbiology Services Colindale, Public Health England

Gorgas

Memorial

Institute for Health Studies

KU Leuven, Clinical and Epidemiological Virology

Beijing Institute of Microbiology and Epidemiology

Respiratory Virus Unit, Microbiology Services Colindale, Public Health England

Respiratory Virus Unit, Microbiology Services Colindale, Public Health England

Respiratory Virus Unit, Microbiology Services Colindale, Public Health England

Respiratory Virus Unit, Microbiology Services Colindale, Public Health England

Gorgas Memorial Institute for Health Studies

KU Leuven, Clinical and Epidemiological Virology

Xiao-Lin Jiang, Xiao-Li Zhang, Xiang-Na Zhao, Cun-Bao Li, Jie Lei, Zeng-Qiang Kou, Wen-Kui Sun, Yang Hang, Feng Gao, Sheng-Xiang Ji, Can-Fang Lin, Bo Pang, Ming-Xiao Yao, Guo-Lin Wang, Lin Yao, Li-Jun Duan, Xiao Wei, Dian-Ming Kang, Mai-Juan Ma

Monica Galiano, Shahjahan Miah, Angie Lackenby, Omolola Akinbami, Tiina Talts, Leena Bhaw, Richard Myers, Steven Platt, Kirstin Edwards, Jonathan Hubb, Joanna Ellis, Maria Zambon

Monica Galiano, Shahjahan Miah, Angie Lackenby, Omolola Akinbami, Tiina Talts, Leena Bhaw, Richard Myers, Steven Platt, Kirstin Edwards, Jonathan Hubb, Joanna Ellis, Maria Zambon

Monica Galiano, Shahjahan Miah, Angie Lackenby, Omolola Akinbami, Tiina Talts, Leena Bhaw, Richard Myers, Steven Platt, Kirstin Edwards, Jonathan Hubb, Joanna Ellis, Maria Zambon

Monica Galiano, Shahjahan Miah, Angie Lackenby, Omolola Akinbami, Tiina Talts, Leena Bhaw, Richard Myers, Steven Platt, Kirstin Edwards, Jonathan Hubb, Joanna Ellis, Maria Zambon

Danilo Franco, Sandra Lopez-Verges, Elimelec Valdespino, Claudia Gonzalez, Oris Chavarria, Ambar Moreno, Yamilka Diaz, Leyda Abrego, Juan M. Pascale, Alexander A. Martinez.

Bert Vanmechelen, Joan Marti-Carreras, Tony Wawina, Marc Van Ranst, Piet Maes

EPI_ISL_415154

EPI_ISL_415155

EPI_ISL_415156

EPI_ISL_415157

EPI_ISL_415158

EPI_ISL_415159

EPI_ISL_415454

EPI_ISL_415455

EPI_ISL_415456

hCoV-19/Belgium/BM-

03012/2020

hCoV-19/Belgium/VAG-

03013/2020

hCoV-19/Belgium/SH-

03014/2020

hCoV-19/Belgium/BC-

03016/2020

hCoV-19/Belgium/QKJ-

03015/2020

hCoV-19/Belgium/BA-

02291/2020

hCoV-

19/Switzerland/GE1422/2020

hCoV-

19/Switzerland/GE0199/2020

hCoV-

19/Switzerland/BE6651/2020

Europe /

Belgium /

Kraainem

Europe /

Belgium /

Huldenberg

Europe /

Belgium /

Huldenberg

Europe /

Belgium / Sint-

Niklaas

Europe /

Belgium /

Brussels

Europe /

Belgium /

Leuven

Europe /

Switzerland

Europe /

Switzerland

Europe /

Switzerland

2020-03-01

2020-03-01

2020-03-01

2020-03-01

2020-03-01

2020-02-29

2020-02-28

2020-02-28

2020-02-29

KU Leuven, Clincal and Epidemiological Virology


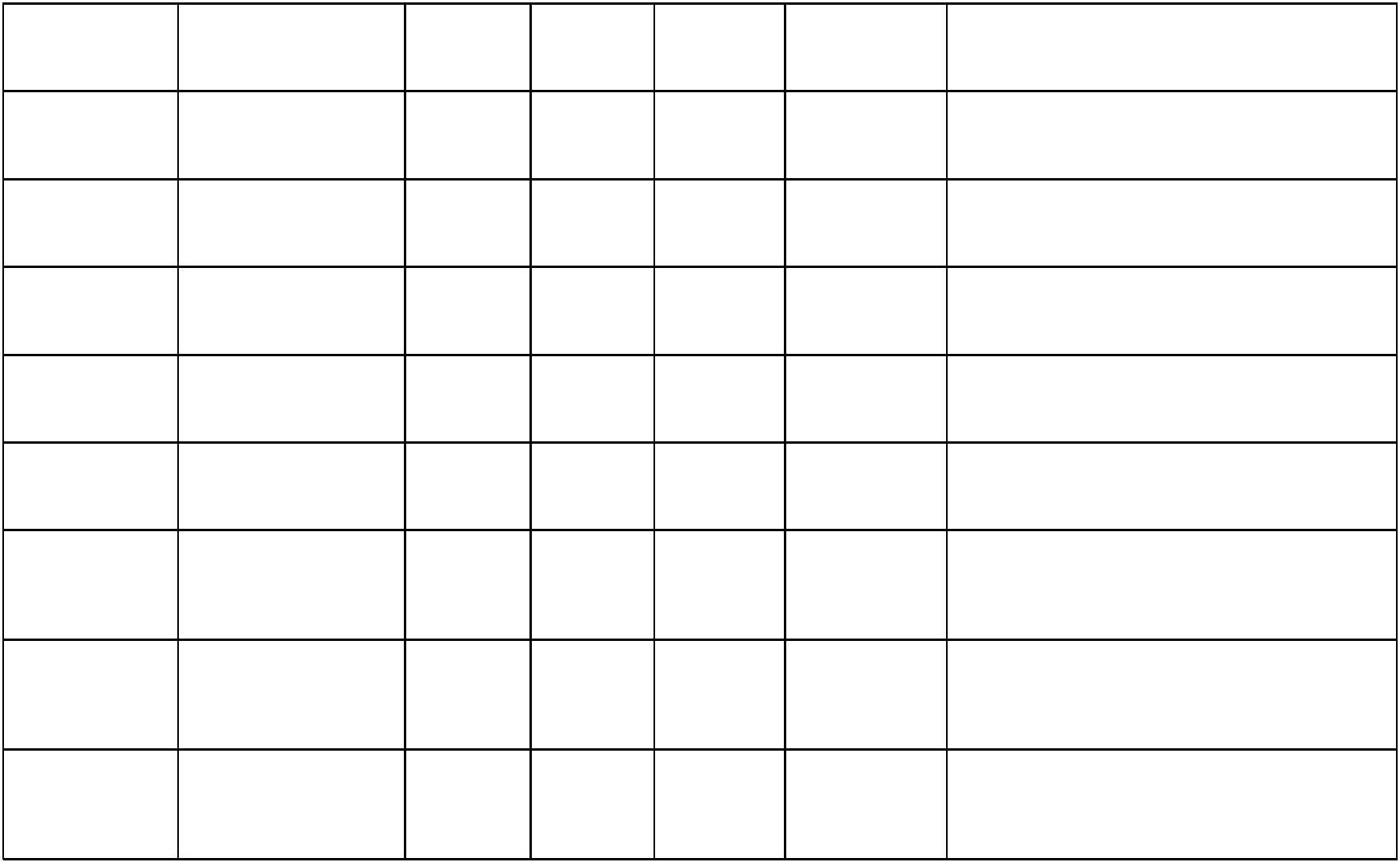


KU Leuven, Clinical and Epidemiological Virology

KU Leuven,

Clinical and

Epidemiological

Virology

KU Leuven, Clinical and Epidemiological Virology

KU Leuven, Clinical and Epidemiological Virology

KU Leuven,

Clinical and

Epidemiological

Virology

Hôpitaux

universitaires de

Genève

Laboratoire de

Virologie

Hôpitaux

universitaires de

Genève

Laboratoire de

Virologie

Hôpitaux universitaires de Genève

Laboratoire de Virologie

KU Leuven, Clincal and Epidemiological Virology

KU Leuven, Clinical and Epidemiological Virology

KU Leuven, Clinical and Epidemiological Virology

KU Leuven, Clinical and Epidemiological Virology

KU Leuven, Clinical and Epidemiological Virology

KU Leuven, Clinical and Epidemiological Virology

Hôpitaux

universitaires de

Genève Laboratoire

de Virologie

Hôpitaux

universitaires de

Genève Laboratoire

de Virologie

Hôpitaux

universitaires de

Genève Laboratoire

de Virologie

Bert Vanmechelen, Joan Marti-Careras, Tony Wawina, Marc Van Ranst, Piet Maes.

Bert Vanmechelen, Joan Marti-Carreras, Tony Wawina, Marc Van Ranst, Piet Maes

Bert Vanmechelen, Joan Marti-Carreras, Tony Wawina, Piet Maes

Bert Vanmechelen, Joan Marti-Carreras, Tony Wawina, Piet Maes

Bert Vanmechelen, Joan Marti-Carreras, Tony Wawina, Piet Maes

Bert Vanmechelen, Joan Marti-Carreras, Tony Wawina, Piet Maes

Laubscher F.

Laubscher F.

Laubscher F.

EPI_ISL_415457

EPI_ISL_415458

EPI_ISL_415459

EPI_ISL_415460

EPI_ISL_415462

EPI_ISL_415463

hCoV-

19/Switzerland/AG7120/2020

hCoV-

19/Switzerland/GE8102/2020

hCoV-

19/Switzerland/VD0503/2020

hCoV-

19/Netherlands/Flevoland_1/2

020

hCoV-

19/Netherlands/Gelderland_2/

2020

hCoV-

19/Netherlands/Gelderland_3/

2020

Europe /

Switzerland

Europe /

Switzerland

Europe / Switzerland / Genève

Europe / Netherlands / Flevoland

Europe / Netherlands / Gelderland

Europe / Netherlands / Gelderland

2020-02-29

2020-03-01

2020-02-29

2020-03-09

2020-03-09

2020-03-09

Hôpitaux


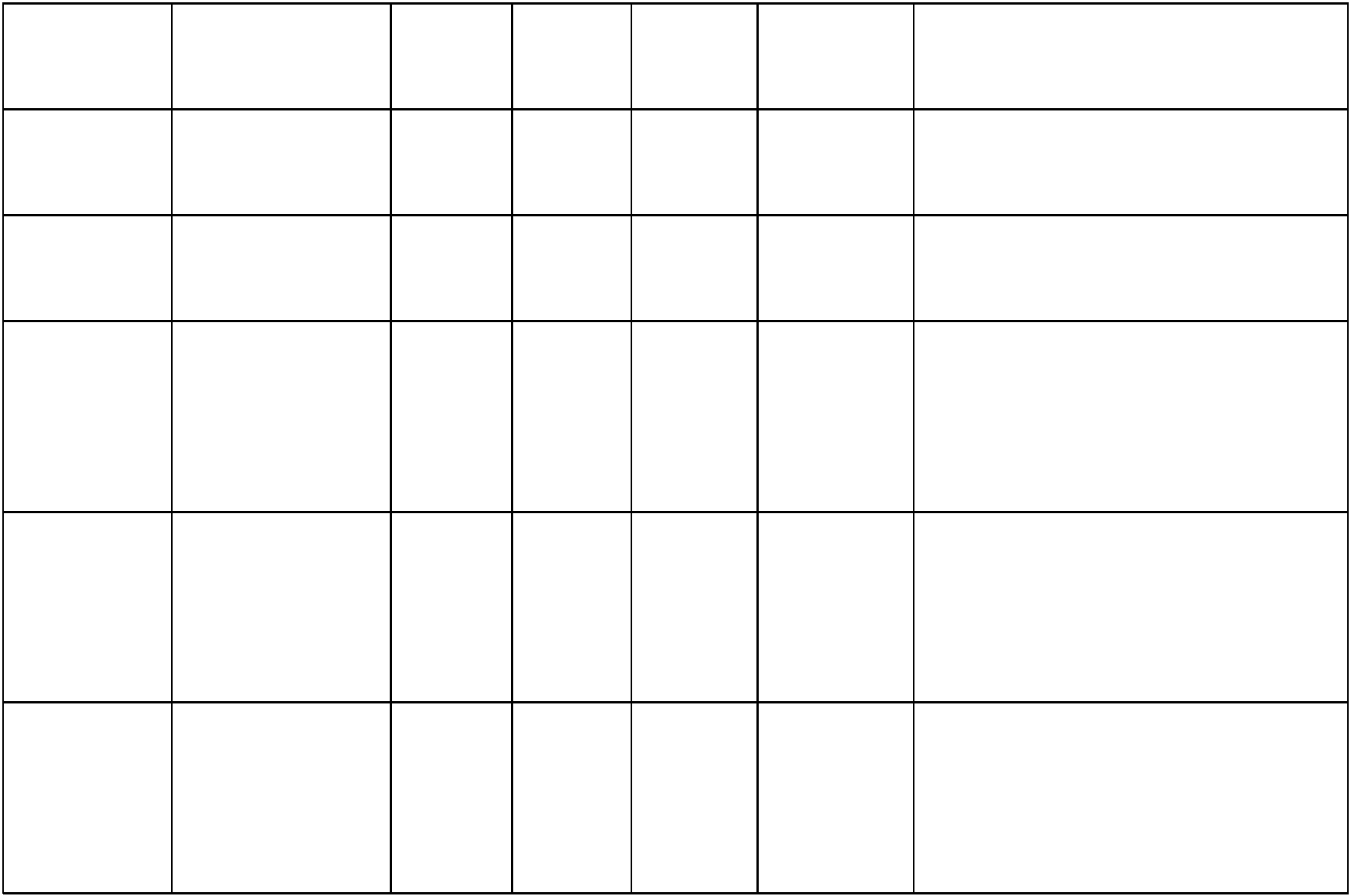


universitaires de

Genève

Laboratoire de

Virologie

Hôpitaux

universitaires de

Genève

Laboratoire de

Virologie

Hôpitaux universitaires de Genève

Laboratoire de Virologie

Dutch COVID-

1. response team

Dutch COVID-

1. response team

Dutch COVID-

1. response team

Hôpitaux

universitaires de

Genève Laboratoire

de Virologie

Hôpitaux

universitaires de

Genève Laboratoire

de Virologie

Hôpitaux

universitaires de

Genève Laboratoire

de Virologie

Erasmus Medical

Center

Erasmus Medical

Center

Erasmus Medical

Center

Laubscher F.

Laubscher F.

Laubscher F.

David Nieuwenhuijse, Bas Oude Munnink, Reina Sikkema, Claudia Schapendonk, Irina Chestakova, Anne van der Linden, Mark Pronk, Pascal Lexmond, Corien Swaan, Manon Haverkate, Madelief Mollers, Mart Stein, Sandra Kengne Kamga Mobou, Jeroen van Kampen, Jolanda Voermans, Aura Timen, Corine GeurtsvanKessel, Annemiek van der Eijk, Richard Molenkamp, Marion Koopmans, on behalf of the Dutch national COVID-19 response team.

David Nieuwenhuijse, Bas Oude Munnink, Reina Sikkema, Claudia Schapendonk, Irina Chestakova, Anne van der Linden, Mark Pronk, Pascal Lexmond, Corien Swaan, Manon Haverkate, Madelief Mollers, Mart Stein, Sandra Kengne Kamga Mobou, Jeroen van Kampen, Jolanda Voermans, Aura Timen, Corine GeurtsvanKessel, Annemiek van der Eijk, Richard Molenkamp, Marion Koopmans, on behalf of the Dutch national COVID-19 response team.

David Nieuwenhuijse, Bas Oude Munnink, Reina Sikkema, Claudia Schapendonk, Irina Chestakova, Anne van der Linden, Mark Pronk, Pascal Lexmond, Corien Swaan, Manon Haverkate, Madelief Mollers, Mart Stein, Sandra Kengne Kamga Mobou, Jeroen van Kampen, Jolanda Voermans, Aura Timen, Corine GeurtsvanKessel, Annemiek van der Eijk, Richard Molenkamp, Marion Koopmans, on behalf of the Dutch national COVID-19 response team.

EPI_ISL_415465

EPI_ISL_415466

EPI_ISL_415467

EPI_ISL_415468

hCoV-

19/Netherlands/NA_1/2020

hCoV-

19/Netherlands/NA_10/2020

hCoV-

19/Netherlands/NA_11/2020

hCoV-

19/Netherlands/NA_12/2020

Europe /

Netherlands

Europe /

Netherlands

Europe /

Netherlands

Europe /

Netherlands

2020-03-10

2020-03-09

2020-03-10

2020-03-10

Dutch COVID-


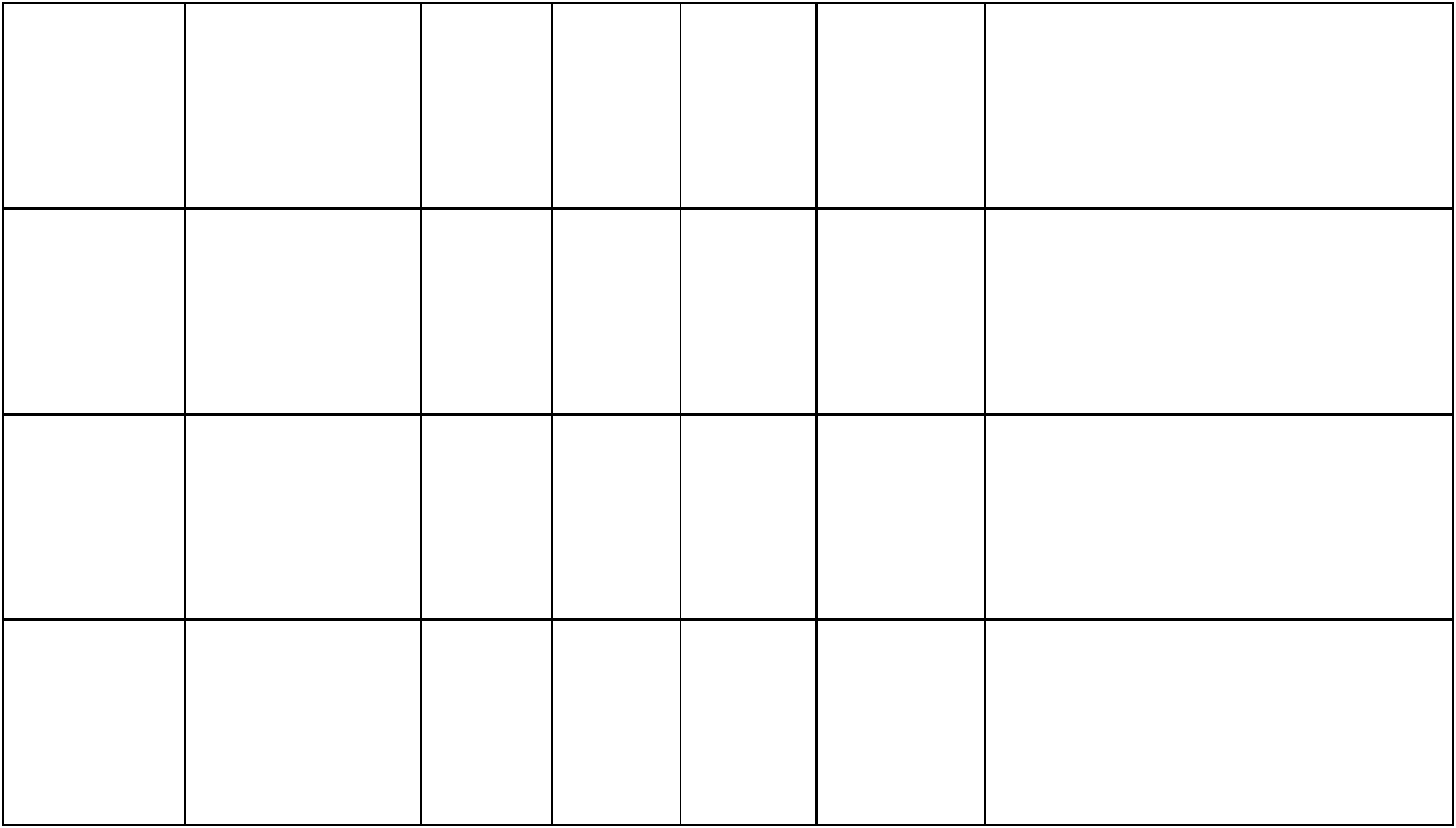


1. response team

Dutch COVID-

1. response team

Dutch COVID-

1. response team

Dutch COVID-

1. response team

Erasmus Medical

Center

Erasmus Medical

Center

Erasmus Medical

Center

Erasmus Medical

Center

David Nieuwenhuijse, Bas Oude Munnink, Reina Sikkema, Claudia Schapendonk, Irina Chestakova, Anne van der Linden, Mark Pronk, Pascal Lexmond, Corien Swaan, Manon Haverkate, Madelief Mollers, Mart Stein, Sandra Kengne Kamga Mobou, Jeroen van Kampen, Jolanda Voermans, Aura Timen, Corine GeurtsvanKessel, Annemiek van der Eijk, Richard Molenkamp, Marion Koopmans, on behalf of the Dutch national COVID-19 response team.

David Nieuwenhuijse, Bas Oude Munnink, Reina Sikkema, Claudia Schapendonk, Irina Chestakova, Anne van der Linden, Mark Pronk, Pascal Lexmond, Corien Swaan, Manon Haverkate, Madelief Mollers, Mart Stein, Sandra Kengne Kamga Mobou, Jeroen van Kampen, Jolanda Voermans, Aura Timen, Corine GeurtsvanKessel, Annemiek van der Eijk, Richard Molenkamp, Marion Koopmans, on behalf of the Dutch national COVID-19 response team.

David Nieuwenhuijse, Bas Oude Munnink, Reina Sikkema, Claudia Schapendonk, Irina Chestakova, Anne van der Linden, Mark Pronk, Pascal Lexmond, Corien Swaan, Manon Haverkate, Madelief Mollers, Mart Stein, Sandra Kengne Kamga Mobou, Jeroen van Kampen, Jolanda Voermans, Aura Timen, Corine GeurtsvanKessel, Annemiek van der Eijk, Richard Molenkamp, Marion Koopmans, on behalf of the Dutch national COVID-19 response team.

David Nieuwenhuijse, Bas Oude Munnink, Reina Sikkema, Claudia Schapendonk, Irina Chestakova, Anne van der Linden, Mark Pronk, Pascal Lexmond, Corien Swaan, Manon Haverkate, Madelief Mollers, Mart Stein, Sandra Kengne Kamga Mobou, Jeroen van Kampen, Jolanda Voermans, Aura Timen, Corine GeurtsvanKessel, Annemiek van der Eijk, Richard Molenkamp, Marion Koopmans, on behalf of the Dutch national COVID-19 response team.

| EPI_ISL_415469 |  | hCoV- |  | Europe / | 2020-03-10 | |  |
| --- | --- | --- | --- | --- | --- | --- | --- |
|  |  | 19/Netherlands/NA_13/2020 |  | Netherlands |  |  |  |
|  |  |  |  |  |  |  |  |
|  |  |  |  |  |  |  |  |

| EPI_ISL_416400 | hCoV- | Asia / China / | 2020-02-02 |  |
| --- | --- | --- | --- | --- |
|  | 19/Shanghai/SH0112/2020 | Shanghai |  |  |
|  |  |  |  |  |

| EPI_ISL_416401 |  | hCoV- |  | Asia / China / | 2020-02-02 | |  |
| --- | --- | --- | --- | --- | --- | --- | --- |
|  |  | 19/Shanghai/SH0114/2020 |  | Shanghai |  |  |  |
|  |  |  |  |  |  |  |  |
|  |  |  |  |  |  |  |  |

Dutch COVID-


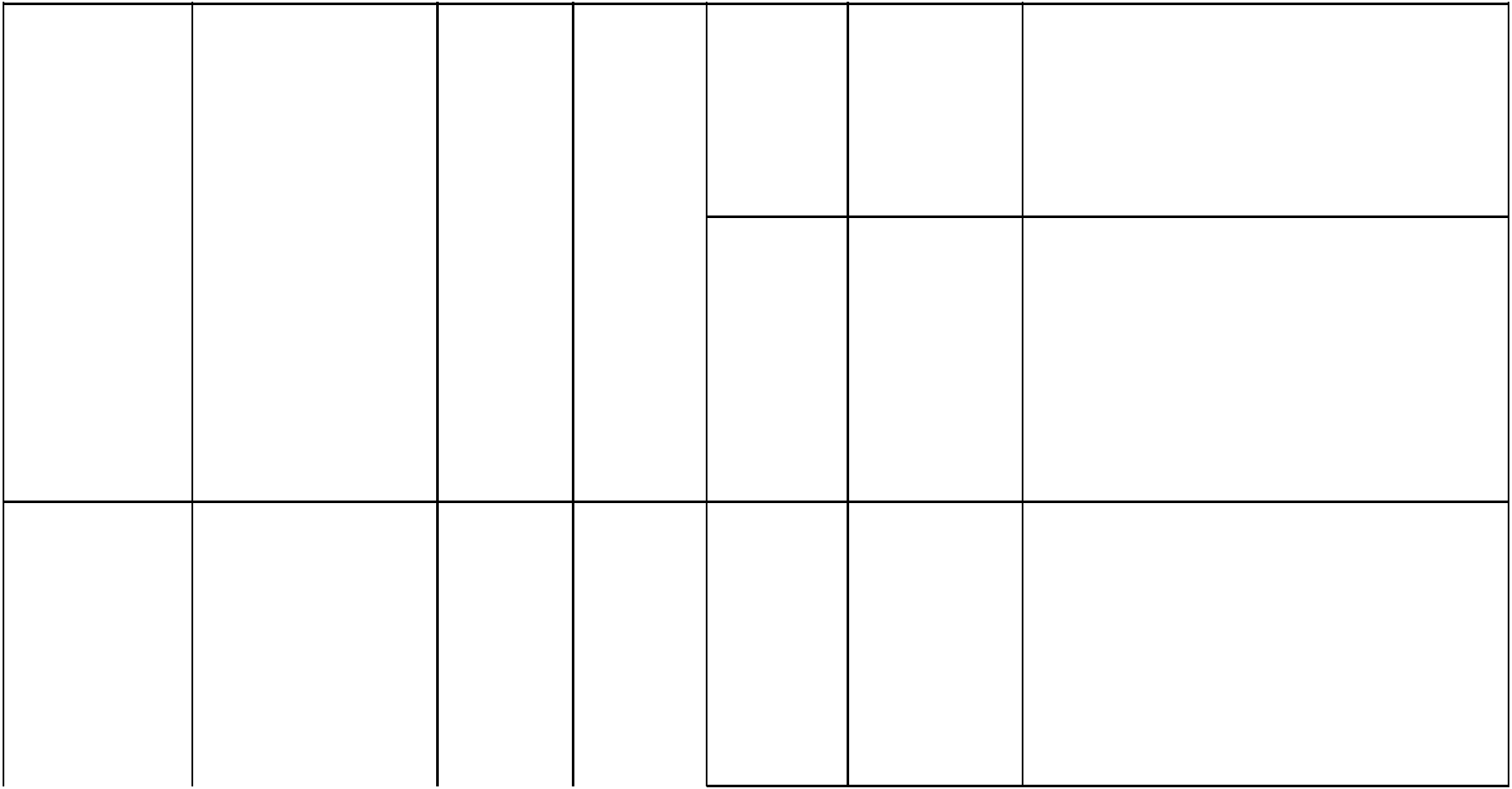


1. response team

Shanghai Public

Health Clinical

Center,

Shanghai

Medical College,

Fudan University

Shanghai Public

Health Clinical

Center,

Shanghai

Medical College,

Fudan University

Erasmus Medical

Center

National Research Center for Translational Medicine

(Shanghai), Ruijin Hospital affiliated to Shanghai Jiao Tong University School of Medicine & Shanghai Public Health Clinical Center

National Research Center for Translational Medicine

(Shanghai), Ruijin Hospital affiliated to Shanghai Jiao Tong University School of Medicine & Shanghai Public Health Clinical Center

David Nieuwenhuijse, Bas Oude Munnink, Reina Sikkema, Claudia Schapendonk, Irina Chestakova, Anne van der Linden, Mark Pronk, Pascal Lexmond, Corien Swaan, Manon Haverkate, Madelief Mollers, Mart Stein, Sandra Kengne Kamga Mobou, Jeroen van Kampen, Jolanda Voermans, Aura Timen, Corine GeurtsvanKessel, Annemiek van der Eijk, Richard Molenkamp, Marion Koopmans, on behalf of the Dutch national COVID-19 response team.

Shengyue Wang, Xiaonan Zhang, Gang Lu, Yun Tan, Yun Ling, Hongzhou Lu, Saijuan Chen

Shengyue Wang, Xiaonan Zhang, Gang Lu, Yun Tan, Yun Ling, Hongzhou Lu, Saijuan Chen

| EPI_ISL_416402 | hCoV- | Asia / China / | 2020-02-11 |  |
| --- | --- | --- | --- | --- |
|  | 19/Shanghai/SH0115/2020 | Shanghai |  |  |
|  |  |  |  |  |

| EPI_ISL_416403 | hCoV- | Asia / China / | 2020-02-02 |  |
| --- | --- | --- | --- | --- |
|  | 19/Shanghai/SH0117/2020 | Shanghai |  |  |
|  |  |  |  |  |

| EPI_ISL_416404 |  | hCoV- |  | Asia / China / | 2020-02-09 | |  |
| --- | --- | --- | --- | --- | --- | --- | --- |
|  |  | 19/Shanghai/SH0119/2020 |  | Shanghai |  |  |  |
|  |  |  |  |  |  |  |  |
|  |  |  |  |  |  |  |  |

Shanghai Public


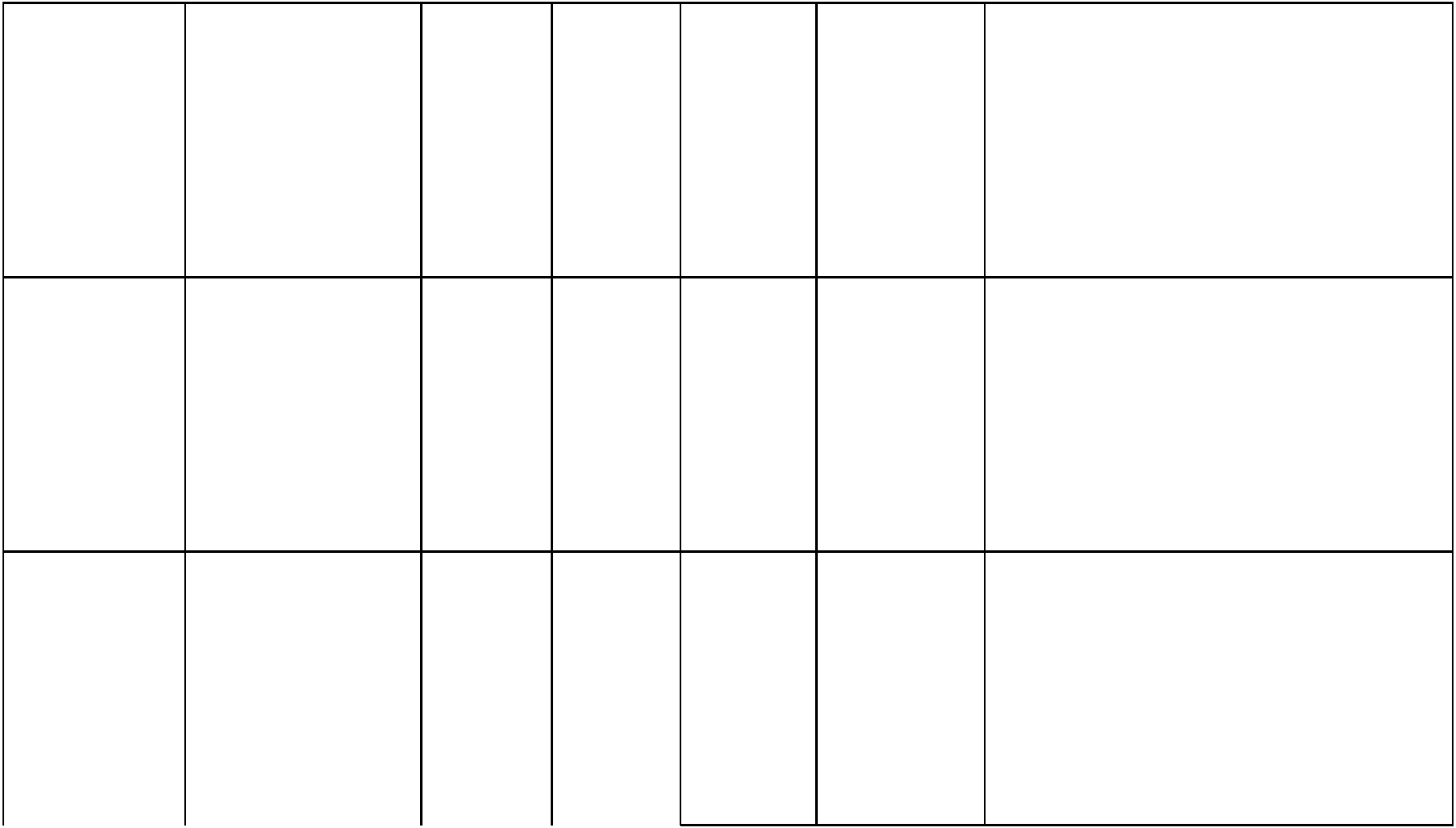


Health Clinical

Center,

Shanghai

Medical College,

Fudan University

Shanghai Public

Health Clinical

Center,

Shanghai

Medical College,

Fudan University

Shanghai Public

Health Clinical

Center,

Shanghai

Medical College,

Fudan University

National Research Center for Translational Medicine

(Shanghai), Ruijin Hospital affiliated to Shanghai Jiao Tong University School of Medicine & Shanghai Public Health Clinical Center

National Research Center for Translational Medicine

(Shanghai), Ruijin Hospital affiliated to Shanghai Jiao Tong University School of Medicine & Shanghai Public Health Clinical Center

National Research Center for Translational Medicine

(Shanghai), Ruijin Hospital affiliated to Shanghai Jiao Tong University School of Medicine & Shanghai Public Health Clinical Center

Shengyue Wang, Xiaonan Zhang, Gang Lu, Yun Tan, Yun Ling, Hongzhou Lu, Saijuan Chen

Shengyue Wang, Xiaonan Zhang, Gang Lu, Yun Tan, Yun Ling, Hongzhou Lu, Saijuan Chen

Shengyue Wang, Xiaonan Zhang, Gang Lu, Yun Tan, Yun Ling, Hongzhou Lu, Saijuan Chen

| EPI_ISL_416405 | hCoV- | Asia / China / | 2020-02-02 |  |
| --- | --- | --- | --- | --- |
|  | 19/Shanghai/SH0121/2020 | Shanghai |  |  |
|  |  |  |  |  |

| EPI_ISL_416406 | hCoV- | Asia / China / | 2020-02-15 |  |
| --- | --- | --- | --- | --- |
|  | 19/Shanghai/SH0125/2020 | Shanghai |  |  |
|  |  |  |  |  |

| EPI_ISL_416407 |  | hCoV- |  | Asia / China / | 2020-02-15 | |  |
| --- | --- | --- | --- | --- | --- | --- | --- |
|  |  | 19/Shanghai/SH0126/2020 |  | Shanghai |  |  |  |
|  |  |  |  |  |  |  |  |
|  |  |  |  |  |  |  |  |

Shanghai Public


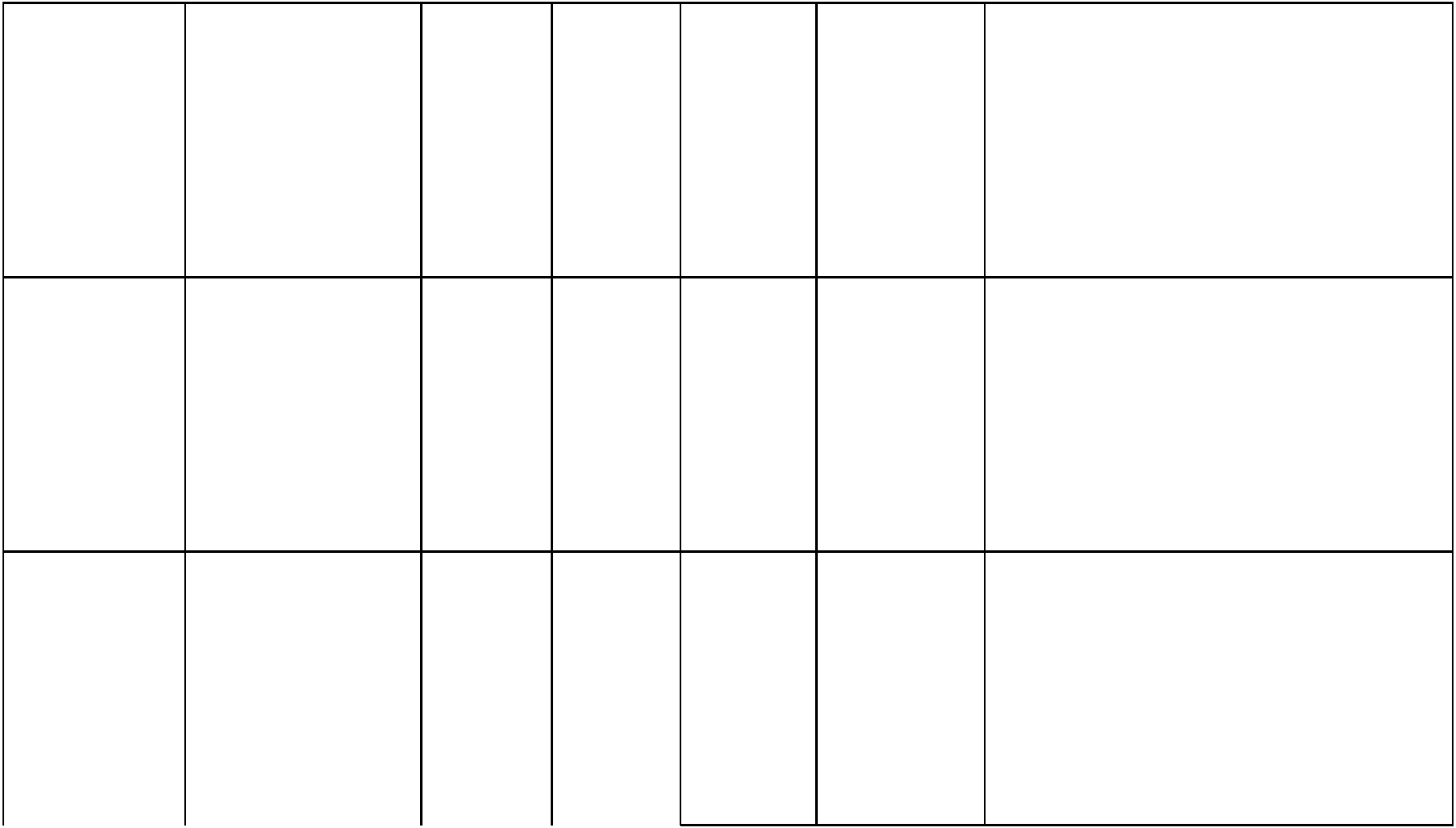


Health Clinical

Center,

Shanghai

Medical College,

Fudan University

Shanghai Public

Health Clinical

Center,

Shanghai

Medical College,

Fudan University

Shanghai Public

Health Clinical

Center,

Shanghai

Medical College,

Fudan University

National Research Center for Translational Medicine

(Shanghai), Ruijin Hospital affiliated to Shanghai Jiao Tong University School of Medicine & Shanghai Public Health Clinical Center

National Research Center for Translational Medicine

(Shanghai), Ruijin Hospital affiliated to Shanghai Jiao Tong University School of Medicine & Shanghai Public Health Clinical Center

National Research Center for Translational Medicine

(Shanghai), Ruijin Hospital affiliated to Shanghai Jiao Tong University School of Medicine & Shanghai Public Health Clinical Center

Shengyue Wang, Xiaonan Zhang, Gang Lu, Yun Tan, Yun Ling, Hongzhou Lu, Saijuan Chen

Shengyue Wang, Xiaonan Zhang, Gang Lu, Yun Tan, Yun Ling, Hongzhou Lu, Saijuan Chen

Shengyue Wang, Xiaonan Zhang, Gang Lu, Yun Tan, Yun Ling, Hongzhou Lu, Saijuan Chen

EPI_ISL_416409

EPI_ISL_416410

EPI_ISL_416411

EPI_ISL_416413

hCoV-

19/Shanghai/SH0128/2020

hCoV-19/Australia/VIC02/2020

hCoV-19/Australia/VIC03/2020

hCoV-19/Australia/VIC05/2020

Asia / China /

Shanghai

Oceania /

Australia /

Victoria /

Melbourne

Oceania /

Australia /

Victoria /

Melbourne

Oceania /

Australia /

Victoria /

Melbourne

2020-02-02

2020-01-24

2020-01-25

2020-03-05

|  |  | National Research |  |
| --- | --- | --- | --- |
|  |  | Center for |  |
| Shanghai Public |  | Translational |  |
|  |  | Medicine |  |
| Health Clinical |  |  |  |
|  |  | (Shanghai), Ruijin |  |
| Center, |  |  |  |
|  |  | Hospital affiliated to |  |
| Shanghai |  |  |  |
|  |  | Shanghai Jiao Tong |  |
| Medical College, |  |  |  |
|  |  | University School of |  |
| Fudan University |  |  |  |
|  |  | Medicine & |  |
|  |  |  |  |
|  |  | Shanghai Public |  |
|  |  | Health Clinical Center |  |
|  |  |  |  |
|  |  | Victorian Infectious |  |
| Victorian |  | Diseases Reference |  |
| Infectious |  | Laboratory and |  |
| Diseases |  | Microbiological |  |
| Reference |  | Diagnostic Unit |  |
| Laboratory |  | Public Health |  |
| (VIDRL) |  | Laboratory, Doherty |  |
|  |  | Institute |  |
|  |  |  |  |
|  |  | Victorian Infectious |  |
| Victorian |  | Diseases Reference |  |
| Infectious |  | Laboratory and |  |
| Diseases |  | Microbiological |  |
| Reference |  | Diagnostic Unit |  |
| Laboratory |  | Public Health |  |
| (VIDRL) |  | Laboratory, Doherty |  |
|  |  | Institute |  |
|  |  |  |  |
|  |  | Victorian Infectious |  |
| Victorian |  | Diseases Reference |  |
| Infectious |  | Laboratory and |  |
| Diseases |  | Microbiological |  |
| Reference |  | Diagnostic Unit |  |
| Laboratory |  | Public Health |  |
| (VIDRL) |  | Laboratory, Doherty |  |
|  |  | Institute |  |
|  |  |  |  |


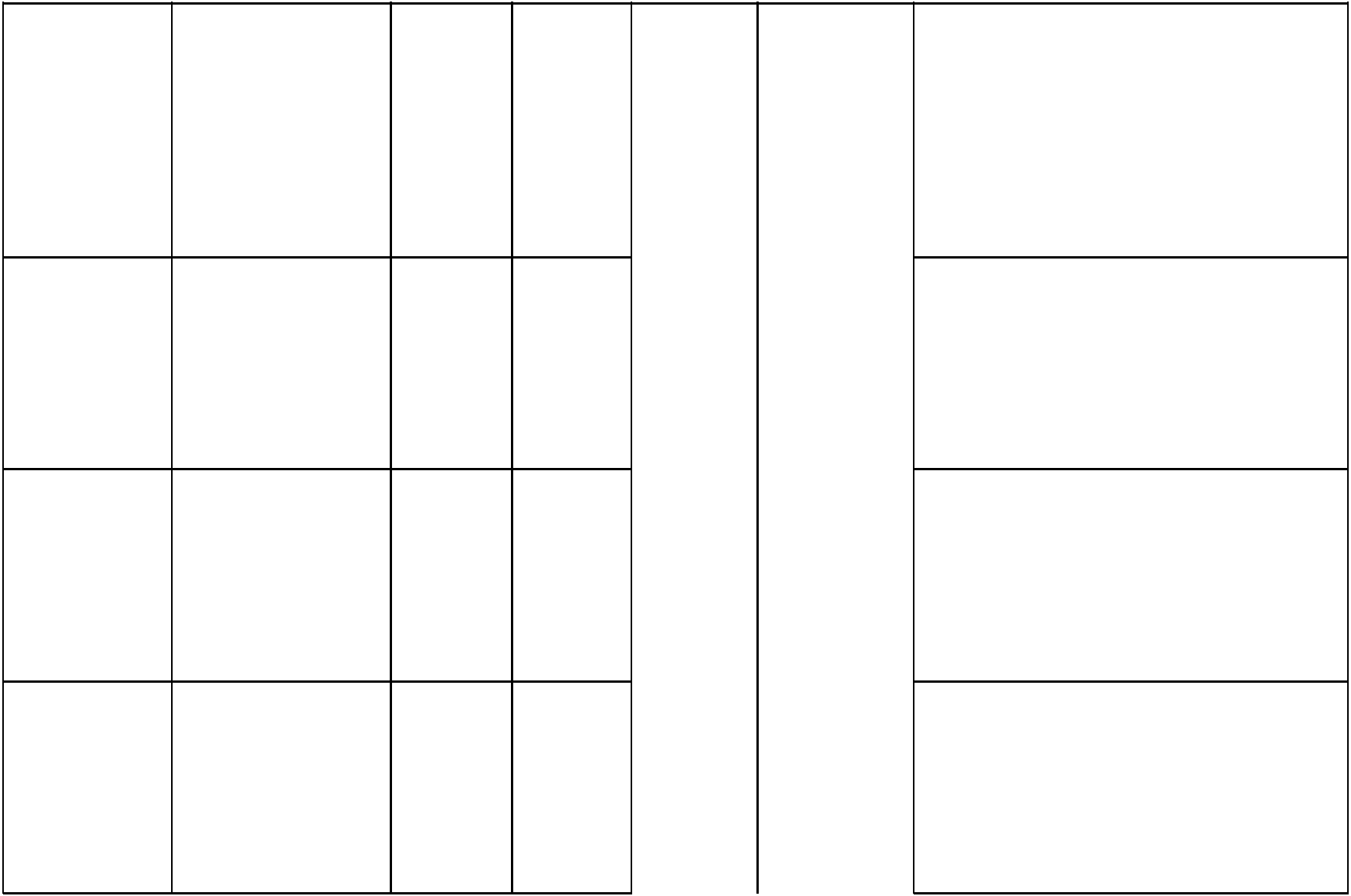


Shengyue Wang, Xiaonan Zhang, Gang Lu, Yun Tan, Yun Ling, Hongzhou Lu, Saijuan Chen

Caly L., Seemann T., Schultz M., Druce J., Taiaroa, G.

Caly L., Seemann T., Schultz M., Druce J., Taiaroa, G.

Caly L., Seemann T., Schultz M., Druce J., Taiaroa, G.

EPI_ISL_416415

EPI_ISL_416425

hCoV-19/Australia/VIC07/2020

hCoV-19/Hangzhou/ZJU-

07/2020

Oceania /

Australia /

Victoria /

Melbourne

Asia / China / Hangzhou

2020-02-08

2020-02-03

Victorian


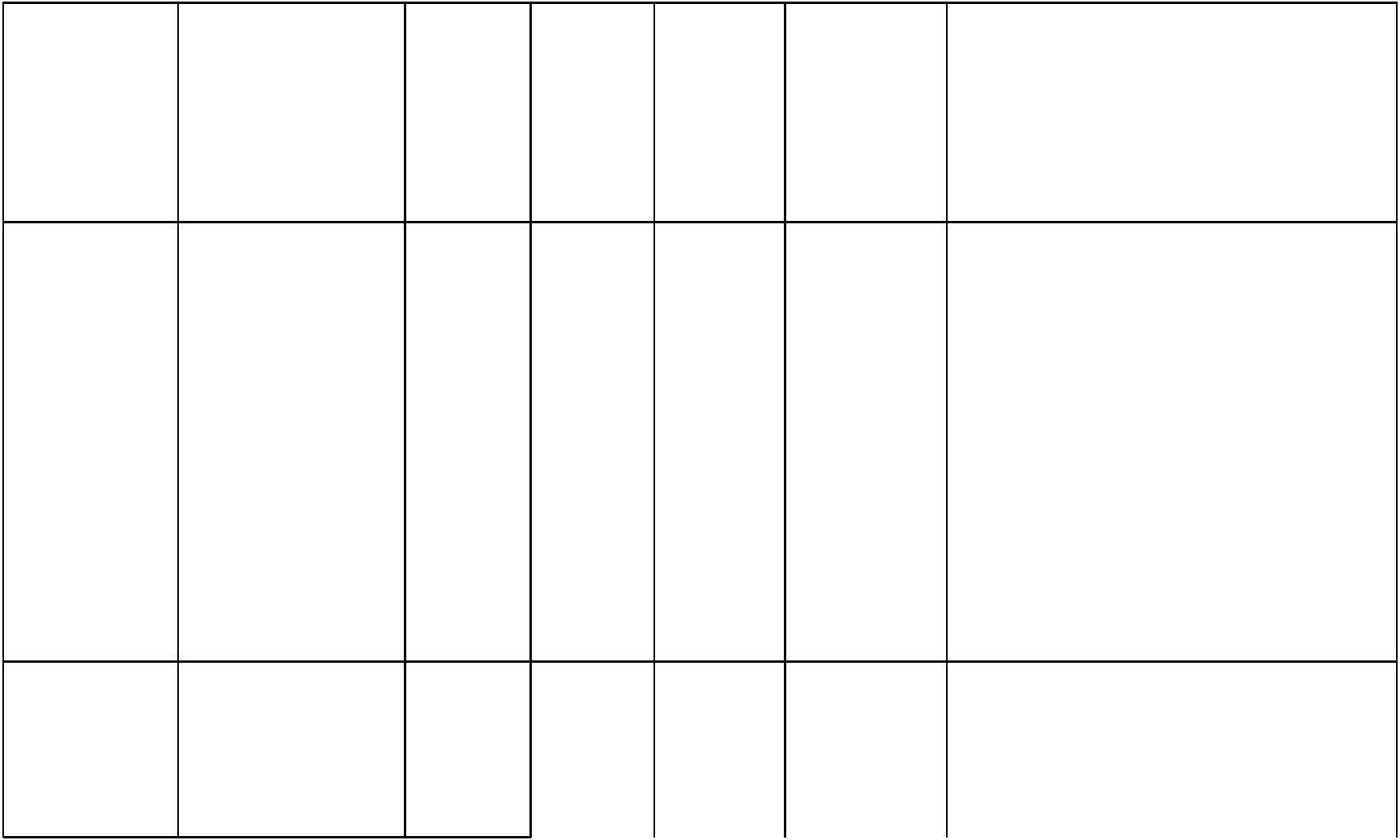


Infectious

Diseases

Reference

Laboratory

(VIDRL)

State Key

Laboratory for Diagnosis and Treatment of Infectious Diseases, National Clinical Research Center for Infectious Diseases, First Affiliated Hospital, Zhejiang University School of Medicine, Hangzhou, China 310003

Victorian Infectious Diseases Reference Laboratory and Microbiological Diagnostic Unit Public Health Laboratory, Doherty Institute

State Key Laboratory for Diagnosis and Treatment of Infectious Diseases, National Clinical

Research Center for Infectious Diseases, First Affiliated Hospital, Zhejiang University School of Medicine, Hangzhou, China 310003

Caly L., Seemann T., Schultz M., Druce J., Taiaroa, G.

Hangping Yao, Nanping Wu, Chao Jiang, Xiangyun Lu, Linfang Cheng, Fumin Liu, Zhigang Wu, Haibo Wu, Changzhong Jin, Min Zheng, Lanjuan Li

EPI_ISL_416428

hCoV-19/Vietnam/39607/2020

Asia / Vietnam /

Quangning

|  | National |  |  | Le Quynh Mai, Taichiro Takemura, Meng Ling Moi, Takeshi |  |
| --- | --- | --- | --- | --- | --- |
|  | Influenza |  | National Influenza |  |  |
|  |  |  |  | Nabeshima, Nguyen Le Khanh Hang, Hoang Vu Mai Phuong, |  |
|  | Center, National |  | Center, National |  |  |
|  |  |  |  | Ung Thi Hong Trang, Le Thi Thanh, Nguyen Vu Son, Vuong |  |
| 2020-03-07 | Institute of |  | Institute of Hygiene |  |  |
|  |  |  |  | Duc Cuong, Pham Thi Hien, Tran Thu Huong, Nguyen |  |
|  | Hygiene and |  | and Epidemiology |  |  |
|  |  |  |  | Phuong Anh, Pham Hong Quynh Anh, Kouichi Morita, |  |
|  | Epidemiology |  | (NIHE) |  |  |
|  |  |  |  | Futoshi Hasebe, Dang Duc Anh |  |
|  | (NIHE) |  |  |  |  |
|  |  |  |  |  |  |
|  |  |  |  |  |  |

EPI_ISL_416429

EPI_ISL_416430

EPI_ISL_416431

hCoV-19/Vietnam/CM99/2020

hCoV-

19/Vietnam/CM295/2020

hCoV-

19/Vietnam/CM296/2020

Asia / Vietnam /

Vinhphuc

Asia / Vietnam /

Hanoi

Asia / Vietnam /

Hanoi

|  | National |  |  | Le Quynh Mai, Taichiro Takemura, Meng Ling Moi, Takeshi |  |
| --- | --- | --- | --- | --- | --- |
|  | Influenza |  | National Influenza |  |  |
|  |  |  |  | Nabeshima, Nguyen Le Khanh Hang, Hoang Vu Mai Phuong, |  |
|  | Center, National |  | Center, National |  |  |
|  |  |  |  | Ung Thi Hong Trang, Le Thi Thanh, Nguyen Vu Son, Vuong |  |
| 2020-02-11 | Institute of |  | Institute of Hygiene |  |  |
|  |  |  |  | Duc Cuong, Pham Thi Hien, Tran Thu Huong, Nguyen |  |
|  | Hygiene and |  | and Epidemiology |  |  |
|  |  |  |  | Phuong Anh, Pham Hong Quynh Anh, Kouichi Morita, |  |
|  | Epidemiology |  | (NIHE) |  |  |
|  |  |  |  | Futoshi Hasebe, Dang Duc Anh |  |
|  | (NIHE) |  |  |  |  |
|  |  |  |  |  |  |
|  |  |  |  |  |  |
|  | National |  |  | Le Quynh Mai, Taichiro Takemura, Meng Ling Moi, Takeshi |  |
|  | Influenza |  | National Influenza |  |  |
|  |  |  |  | Nabeshima, Nguyen Le Khanh Hang, Hoang Vu Mai Phuong, |  |
|  | Center, National |  | Center, National |  |  |
|  |  |  |  | Ung Thi Hong Trang, Le Thi Thanh, Nguyen Vu Son, Vuong |  |
| 2020-03-06 | Institute of |  | Institute of Hygiene |  |  |
|  |  |  |  | Duc Cuong, Pham Thi Hien, Tran Thu Huong, Nguyen |  |
|  | Hygiene and |  | and Epidemiology |  |  |
|  |  |  |  | Phuong Anh, Pham Hong Quynh Anh, Kouichi Morita, |  |
|  | Epidemiology |  | (NIHE) |  |  |
|  |  |  |  | Futoshi Hasebe, Dang Duc Anh |  |
|  | (NIHE) |  |  |  |  |
|  |  |  |  |  |  |
|  |  |  |  |  |  |
|  | National |  |  | Le Quynh Mai, Taichiro Takemura, Meng Ling Moi, Takeshi |  |
|  | Influenza |  | National Influenza |  |  |
|  |  |  |  | Nabeshima, Nguyen Le Khanh Hang, Hoang Vu Mai Phuong, |  |
|  | Center, National |  | Center, National |  |  |
| 2020-03-06 |  |  |  | Ung Thi Hong Trang, Le Thi Thanh, Nguyen Vu Son, Vuong |  |
|  | Institute of |  | Institute of Hygiene |  |  |
|  |  |  |  | Duc Cuong, Pham Thi Hien, Tran Thu Huong, Nguyen |  |
|  | Hygiene and |  | and Epidemiology |  |  |
|  |  |  |  | Phuong Anh, Pham Hong Quynh Anh, Kouichi Morita, |  |
|  | Epidemiology |  | (NIHE) |  |  |
|  |  |  |  | Futoshi Hasebe, Dang Duc Anh |  |
|  | (NIHE) |  |  |  |  |
|  |  |  |  |  |  |


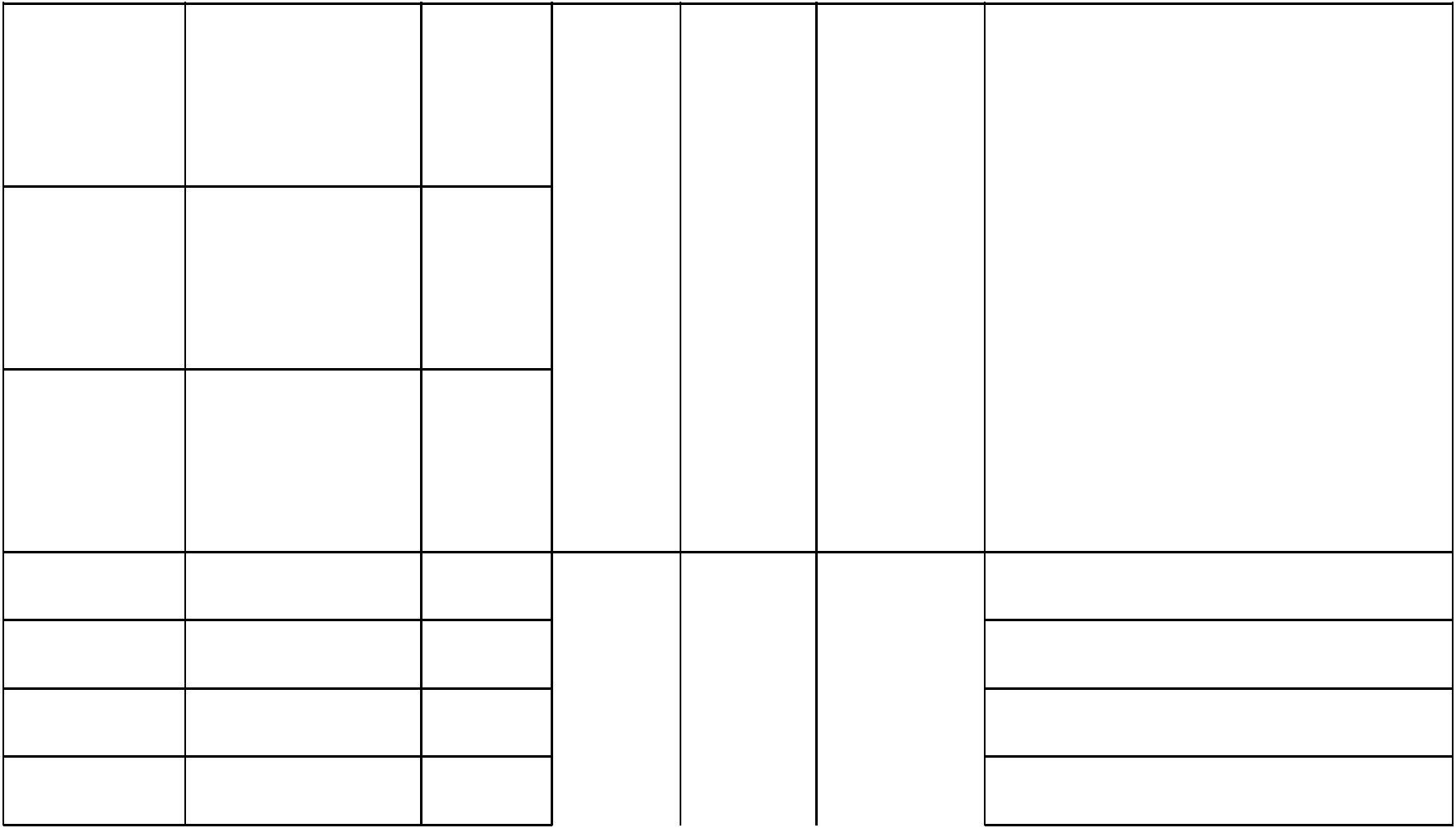


EPI_ISL_416443

EPI_ISL_416445

EPI_ISL_416447

EPI_ISL_416454

hCoV-19/USA/WA-

UW87/2020

hCoV-19/USA/WA-

UW89/2020

hCoV-19/USA/WA-

UW91/2020

hCoV-19/USA/WA-

UW37/2020

North America /

USA /

Washington

North America /

USA /

Washington

North America /

USA /

Washington

North America /

USA /

Washington

| 2020-03-10 | UW Virology Lab |  | UW Virology Lab |
| --- | --- | --- | --- |
|  |  |  |  |
| 2020-03-10 | UW Virology Lab |  | UW Virology Lab |
|  |  |  |  |
| 2020-03-10 | UW Virology Lab |  | UW Virology Lab |
|  |  |  |  |
| 2020-03-06 | UW Virology Lab |  | UW Virology Lab |
|  |  |  |  |

Pavitra Roychoudhury, Hong Xie, Keith Jerome, Alexander Greninger

Pavitra Roychoudhury, Hong Xie, Keith Jerome, Alexander Greninger

Pavitra Roychoudhury, Hong Xie, Keith Jerome, Alexander Greninger

Pavitra Roychoudhury, Hong Xie, Keith Jerome, Alexander Greninger

EPI_ISL_416458

hCoV-19/Kuwait/KU12/2020

Asia / Kuwait /

Hawali

North America /

|  | Virology |  |  |  |
| --- | --- | --- | --- | --- |
|  | laboratory |  |  |  |
|  | Ministry of |  |  |  |
| 2020-03-02 | Health Kuwait | Dasman Diabetes | Fahd Al-Mulla, Sumi John, Sara Alqabandi, Rasheeba iqbal, |  |
|  | sequenced at | Institute | Motasem Melhem, Ebaa alOzairi, Qais Al-Duwairi |  |
|  |  |  |  |  |


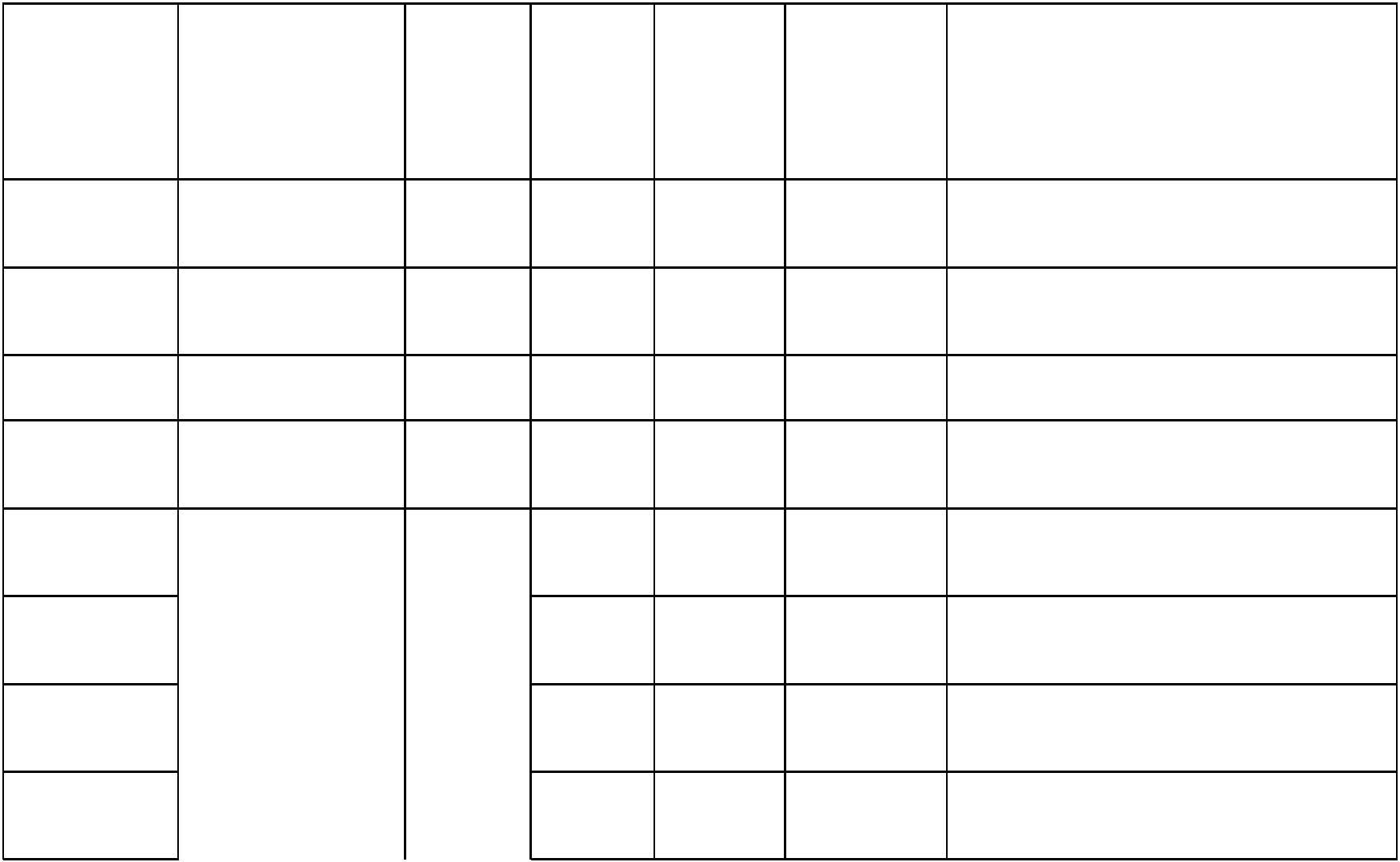


Dasman

Diabetes

Institute

EPI_ISL_416460

EPI_ISL_416461

EPI_ISL_416462

EPI_ISL_416465

hCoV-19/USA/WA-S5/2020

hCoV-19/USA/WA-S6/2020

hCoV-19/USA/WA-S7/2020

hCoV-19/USA/WA-S10/2020

USA /

Washington /

King County

North America /

USA /

Washington /

King County

North America /

USA /

Washington

North America /

USA /

Washington /

King County

2020-02-29

2020-02-29

2020-02-24

2020-02-29

Seattle Flu Study

Seattle Flu Study

Seattle Flu Study

Seattle Flu Study

Seattle Flu Study

Seattle Flu Study

Seattle Flu Study

Seattle Flu Study

Chu et al

Chu et al

Chu et al

Chu et al

EPI_ISL_416467

EPI_ISL_416468

EPI_ISL_416469

EPI_ISL_416470

| hCoV-19/Belgium/MTR- |  | Europe | / |  |
| --- | --- | --- | --- | --- |
|  |  | Belgium / | |  |
| 03021/2020 |  |  |  |  |
|  |  | Holsbeek | |  |
|  |  |  |  |  |
|  |  |  |  |  |
| hCoV-19/Belgium/GMH- |  | Europe | / |  |
|  |  | Belgium / | |  |
| 03022/2020 |  |  |  |  |
|  |  | Holsbeek | |  |
|  |  |  |  |  |
|  |  |  |  |  |
| hCoV-19/Belgium/SN- |  | Europe | / |  |
|  |  | Belgium | / |  |
| 03031/2020 |  |  |  |  |
|  |  | Kessel-Lo | |  |
|  |  |  |  |  |
|  |  |  |  |  |
| hCoV-19/Belgium/DB- |  | Europe | / |  |
|  |  | Belgium / | |  |
| 03023/2020 |  |  |  |  |
|  |  | Couthuin |  |  |
|  |  |  |  |  |
|  |  |  |  |  |

2020-03-02

2020-03-02

2020-03-03

2020-03-02

KU Leuven, Clinical and Epidemiological Virology

KU Leuven, Clinical and Epidemiological Virology

KU Leuven, Clinical and Epidemiological Virology

KU Leuven, Clinical and Epidemiological Virology

KU Leuven, Clinical and Epidemiological Virology

KU Leuven, Clinical and Epidemiological Virology

KU Leuven, Clinical and Epidemiological Virology

KU Leuven, Clinical and Epidemiological Virology

Bert Vanmechelen, Tony Wawina, Joan Marti-Carreras, Piet Maes

Bert Vanmechelen, Tony Wawina, Joan Marti-Carreras, Piet Maes

Bert Vanmechelen, Tony Wawina, Joan Marti-Carreras, Piet Maes

Bert Vanmechelen, Tony Wawina, Joan Marti-Carreras, Piet Maes

|  |  | hCoV-19/Belgium/DBD- |  | Europe | / |  |
| --- | --- | --- | --- | --- | --- | --- |
| EPI_ISL_416471 |  |  |  | Belgium | / |  |
|  | 03024/2020 | |  |  |  |  |
|  |  |  |  | Kessel-Lo | |  |
|  |  |  |  |  |  |  |
|  |  |  |  |  |  |  |
| EPI_ISL_416472 |  | hCoV-19/Belgium/UMF- |  | Europe | / |  |
|  |  |  |  | Belgium | / |  |
|  | 03025/2020 | |  |  |  |  |
|  |  |  |  | Kessel-Lo | |  |
|  |  |  |  |  |  |  |
|  |  |  |  |  |  |  |

| EPI_ISL_416473 |  | hCoV-19/Hangzhou/ZJU- |  | Asia / China / |  |
| --- | --- | --- | --- | --- | --- |
|  | 08/2020 | |  | Hangzhou |  |
|  |  |  |  |  |  |
|  |  |  |  |  |  |

2020-03-02

2020-03-02

2020-01-26

KU Leuven, Clinical and Epidemiological Virology


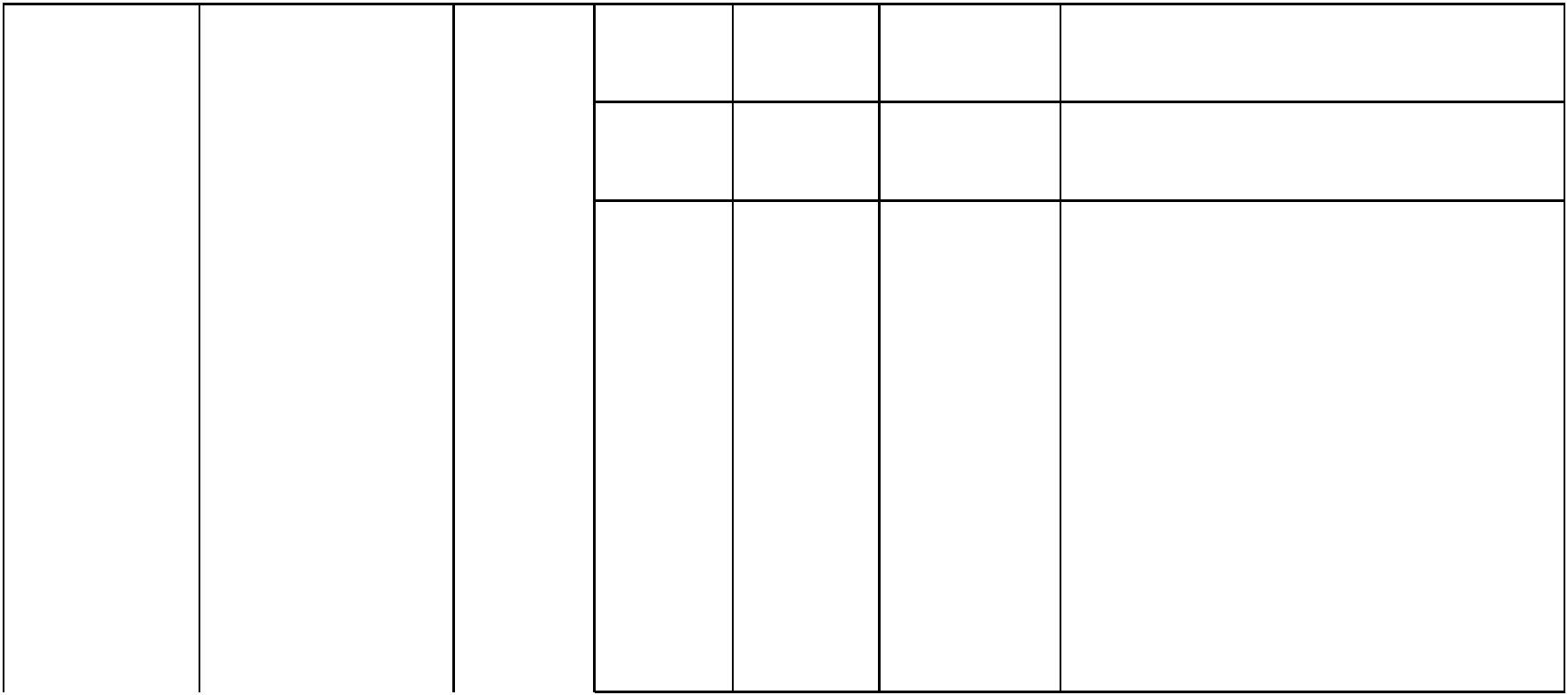


KU Leuven, Clinical and Epidemiological Virology

State Key

Laboratory for Diagnosis and Treatment of Infectious Diseases, National Clinical Research Center for Infectious Diseases, First Affiliated Hospital, Zhejiang University School of Medicine, Hangzhou, China 310003

KU Leuven, Clinical and Epidemiological Virology

KU Leuven, Clinical and Epidemiological Virology

State Key Laboratory for Diagnosis and Treatment of Infectious Diseases, National Clinical

Research Center for Infectious Diseases, First Affiliated Hospital, Zhejiang University School of Medicine, Hangzhou, China 310003

Bert Vanmechelen, Tony Wawina, Joan Marti-Carreras, Piet Maes

Bert Vanmechelen, Tony Wawina, Joan Marti-Carreras, Piet Maes

Hangping Yao, Nanping Wu, Chao Jiang, Xiangyun Lu, Linfang Cheng, Fumin Liu, Zhigang Wu, Haibo Wu, Changzhong Jin, Min Zheng, Lanjuan Li

EPI_ISL_416474

EPI_ISL_416475

EPI_ISL_416476

EPI_ISL_416477

hCoV-19/Hangzhou/ZJU-

09/2020

hCoV-19/Belgium/DBA-

03032/2020

hCoV-19/Belgium/MTR-

03026/2020

hCoV-19/Georgia/Tb-390/2020

Asia / China / Hangzhou

Europe /

Belgium /

Leuven

Europe /

Belgium /

Holsbeek

Asia / Georgia /

Tbilisi

2020-01-28

2020-03-03

2020-03-02

2020-03-08

| State Key |  |  |  |  |
| --- | --- | --- | --- | --- |
| Laboratory for |  |  |  |  |
| Diagnosis and |  |  |  |  |
| Treatment of |  | State Key Laboratory |  |  |
| Infectious |  |  |  |  |
|  |  | for Diagnosis and |  |  |
| Diseases, |  |  |  |  |
|  |  | Treatment of |  |  |
| National Clinical |  |  |  |  |
|  |  | Infectious Diseases, |  |  |
| Research |  |  |  |  |
|  |  | National Clinical |  |  |
| Center for |  |  | Hangping Yao, Nanping Wu, Chao Jiang, Xiangyun Lu, |  |
|  |  | Research Center for |  |  |
| Infectious |  |  | Linfang Cheng, Fumin Liu, Zhigang Wu, Haibo Wu, |  |
|  |  | Infectious Diseases, |  |  |
| Diseases, First |  |  | Changzhong Jin, Min Zheng, Lanjuan Li |  |
|  |  | First Affiliated |  |  |
| Affiliated |  |  |  |  |
|  |  | Hospital, Zhejiang |  |  |
| Hospital, |  |  |  |  |
|  |  | University School of |  |  |
| Zhejiang |  |  |  |  |
|  |  | Medicine, Hangzhou, |  |  |
| University |  |  |  |  |
|  |  | China 310003 |  |  |
| School of |  |  |  |  |
|  |  |  |  |  |
| Medicine, |  |  |  |  |
| Hangzhou, |  |  |  |  |
| China 310003 |  |  |  |  |
|  |  |  |  |  |
| KU Leuven, |  | KU Leuven, Clinical |  |  |
| Clinical and |  |  | Bert Vanmechelen, Tony Wawina, Joan Marti-Carreras, Piet |  |
|  |  | and Epidemiological |  |  |
| Epidemiological |  |  | Maes |  |
|  |  | Virology |  |  |
| Virology |  |  |  |  |
|  |  |  |  |  |
| KU Leuven, |  | KU Leuven, Clinical |  |  |
| Clinical and |  |  | Bert Vanmechelen, Tony Wawina, Joan Marti-Carreras, Piet |  |
|  |  | and Epidemiological |  |  |
| Epidemiological |  |  | Maes |  |
|  |  | Virology |  |  |
| Virology |  |  |  |  |
|  |  |  |  |  |
|  |  |  |  |  |
| R. G. Lugar |  |  | Marine Murtskhvaladze, Nato Kotaria, Ann Machablishvili, |  |
|  |  |  | Lela Sabadze, Mari Gavashelidze, Ana Papkiauri, Meri |  |
| Center for |  |  |  |  |
|  |  | R. G. Lugar Center | Pantsulaia, Gvantsa Brachveli, Tata Imnadze, Tamar |  |
| Public Health |  |  |  |  |
|  |  | for Public Health | Jashiashvili, Tea Tevdoradze, Ketevan Sidamonidze, |  |
| Research, |  |  |  |  |
|  |  | Research, National | Ekaterine Khmaladze, Ekaterine Zhghenti, Roena |  |
| National Center |  |  |  |  |
|  |  | Center for Disease | Sukhiashvili, Mariam Zakalashvili, Lela Urushadze, Magda |  |
| for Disease |  |  |  |  |
|  |  | Control and Public | Dgebuadze, Giorgi Tomashvili, Davit Tsaguria, Ekaterine |  |
| Control and |  |  |  |  |
|  |  | Health (NCDC) of | Zangaladze, Nino Berishvili, Gvantsa Chanturia, Adam |  |
| Public Health |  |  |  |  |
|  |  | Georgia. | Kotorashvili, Maia Alkhazashvili, Irma Burjanadze, Anna |  |
| (NCDC) of |  |  |  |  |
|  |  |  | Kasradze, Khatuna Zakhashvili, Paata Imnadze, Amiran |  |
| Georgia. |  |  |  |  |
|  |  |  | Gamkrelidze. |  |
|  |  |  |  |  |
|  |  |  |  |  |


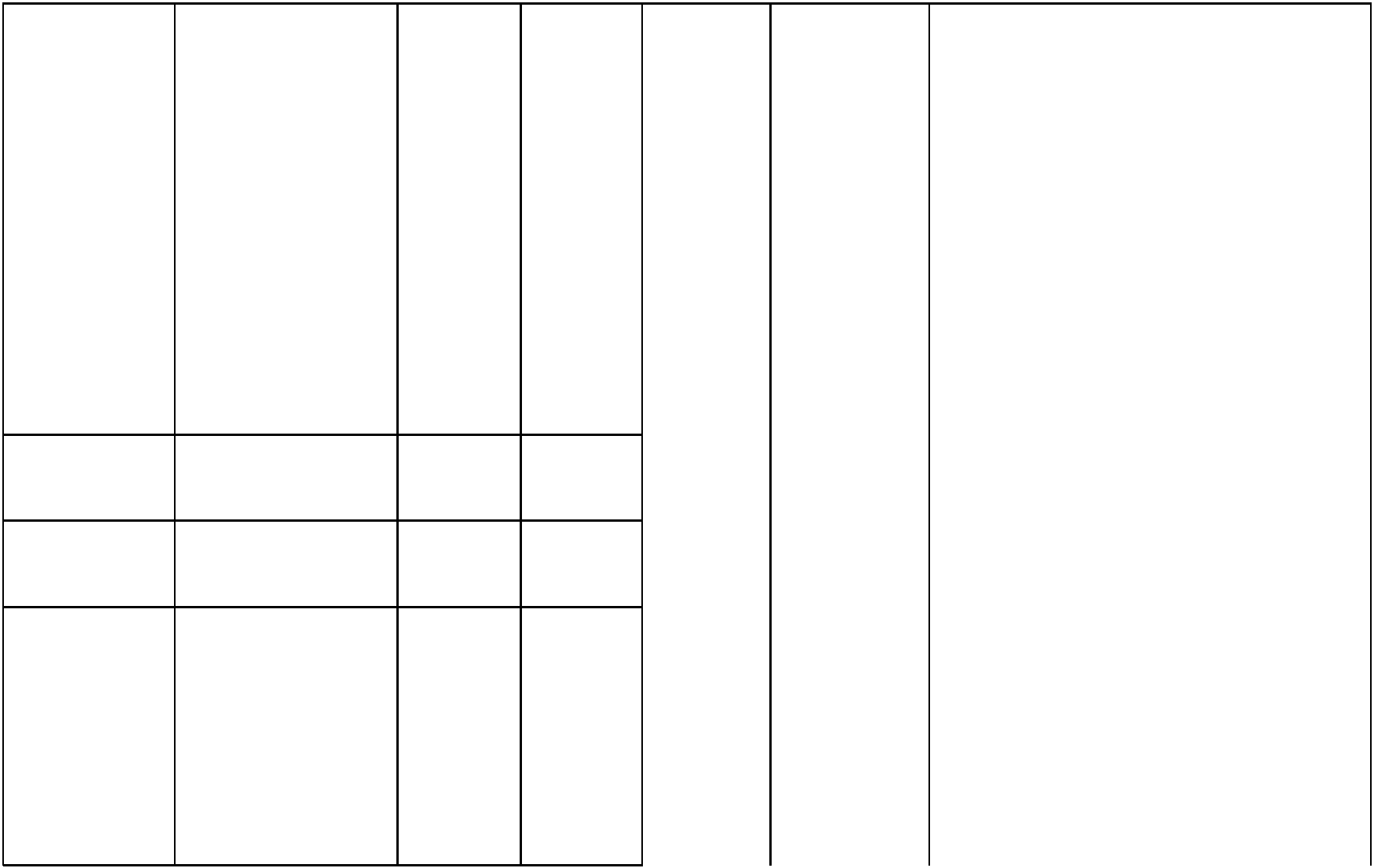


| EPI_ISL_416478 | hCoV-19/Georgia/Tb-673/2020 | Asia / Georgia / | 2020-03-14 |
| --- | --- | --- | --- |
|  |  | Tbilisi |  |

| EPI_ISL_416479 | hCoV-19/Georgia/Tb-273/2020 | Asia / Georgia / | 2020-03-05 |
| --- | --- | --- | --- |
|  |  | Tbilisi |  |

| EPI_ISL_416480 |  | hCoV-19/Georgia/Tb-537/2020 |  | Asia / Georgia / | 2020-03-11 | |
| --- | --- | --- | --- | --- | --- | --- |
|  |  |  |  | Tbilisi |  |  |
|  |  |  |  |  |  |  |

R. G. Lugar


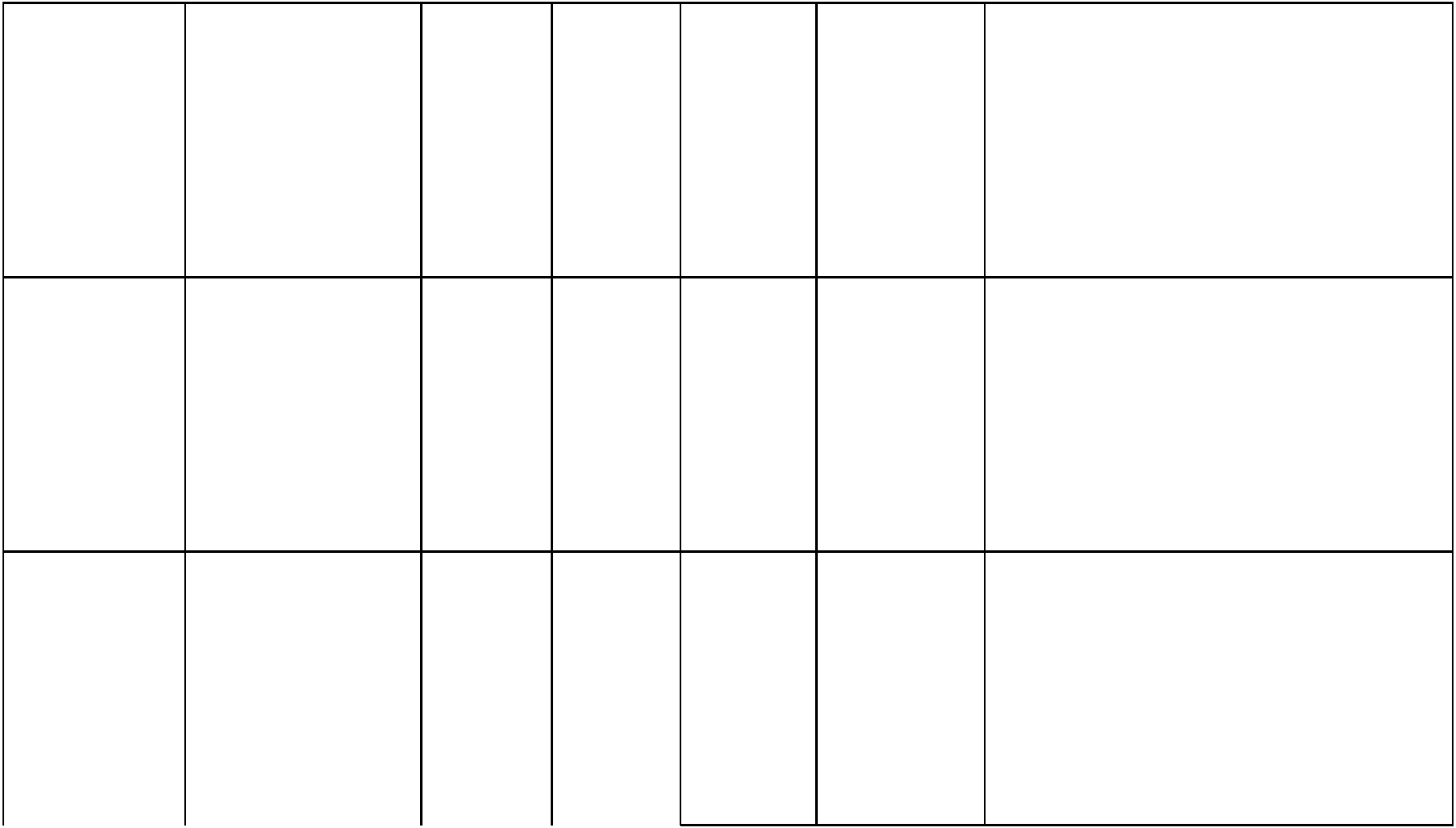


Center for

Public Health

Research,

National Center

for Disease

Control and

Public Health

(NCDC) of

Georgia.

R. G. Lugar

Center for

Public Health

Research,

National Center

for Disease

Control and

Public Health

(NCDC) of

Georgia.

R. G. Lugar

Center for

Public Health

Research,

National Center

for Disease

Control and

Public Health

(NCDC) of

Georgia.

R. G. Lugar Center

for Public Health

Research, National

Center for Disease

Control and Public

Health (NCDC) of

Georgia.

R. G. Lugar Center

for Public Health

Research, National

Center for Disease

Control and Public

Health (NCDC) of

Georgia.

R. G. Lugar Center

for Public Health

Research, National

Center for Disease

Control and Public

Health (NCDC) of

Georgia.

Marine Murtskhvaladze, Nato Kotaria, Ann Machablishvili, Lela Sabadze, Mari Gavashelidze, Ana Papkiauri, Meri Pantsulaia, Gvantsa Brachveli, Tata Imnadze, Tamar Jashiashvili, Tea Tevdoradze, Ketevan Sidamonidze, Ekaterine Khmaladze, Ekaterine Zhghenti, Roena Sukhiashvili, Mariam Zakalashvili, Lela Urushadze, Magda Dgebuadze, Giorgi Tomashvili, Davit Tsaguria, Ekaterine Zangaladze, Nino Berishvili, Gvantsa Chanturia, Adam Kotorashvili, Maia Alkhazashvili, Irma Burjanadze, Anna Kasradze, Khatuna Zakhashvili, Paata Imnadze, Amiran Gamkrelidze.

Marine Murtskhvaladze, Nato Kotaria, Ann Machablishvili, Lela Sabadze, Mari Gavashelidze, Ana Papkiauri, Meri Pantsulaia, Gvantsa Brachveli, Tata Imnadze, Tamar Jashiashvili, Tea Tevdoradze, Ketevan Sidamonidze, Ekaterine Khmaladze, Ekaterine Zhghenti, Roena Sukhiashvili, Mariam Zakalashvili, Lela Urushadze, Magda Dgebuadze, Giorgi Tomashvili, Davit Tsaguria, Ekaterine Zangaladze, Nino Berishvili, Gvantsa Chanturia, Adam Kotorashvili, Maia Alkhazashvili, Irma Burjanadze, Anna Kasradze, Khatuna Zakhashvili, Paata Imnadze, Amiran Gamkrelidze.

Ann Machablishvili, Nato Kotaria, Marine Murtskhvaladze, Lela Sabadze, Mari Gavashelidze, Ana Papkiauri, Meri Pantsulaia, Gvantsa Brachveli, Tata Imnadze, Tamar Jashiashvili, Tea Tevdoradze, Ketevan Sidamonidze, Ekaterine Khmaladze, Ekaterine Zhghenti, Roena Sukhiashvili, Mariam Zakalashvili, Lela Urushadze, Magda Dgebuadze, Giorgi Tomashvili, Davit Tsaguria, Ekaterine Zangaladze, Nino Berishvili, Gvantsa Chanturia, Adam Kotorashvili, Maia Alkhazashvili, Irma Burjanadze, Anna Kasradze, Khatuna Zakhashvili, Paata Imnadze, Amiran Gamkrelidze.

EPI_ISL_416482

EPI_ISL_416484

EPI_ISL_416485

EPI_ISL_416486

hCoV-19/Georgia/Tb/2020

hCoV-

19/Spain/Valencia5/2020

hCoV-

19/Spain/Valencia6/2020

hCoV-

19/Spain/Valencia7/2020

Asia / Georgia /

Tbilisi

Europe / Spain

- Comunitat Valenciana / Valencia

Europe / Spain

- Comunitat Valenciana / Valencia

Europe / Spain

- Comunitat Valenciana / Valencia

2020-03-13

2020-02-27

2020-02-27

2020-03-02

R. G. Lugar


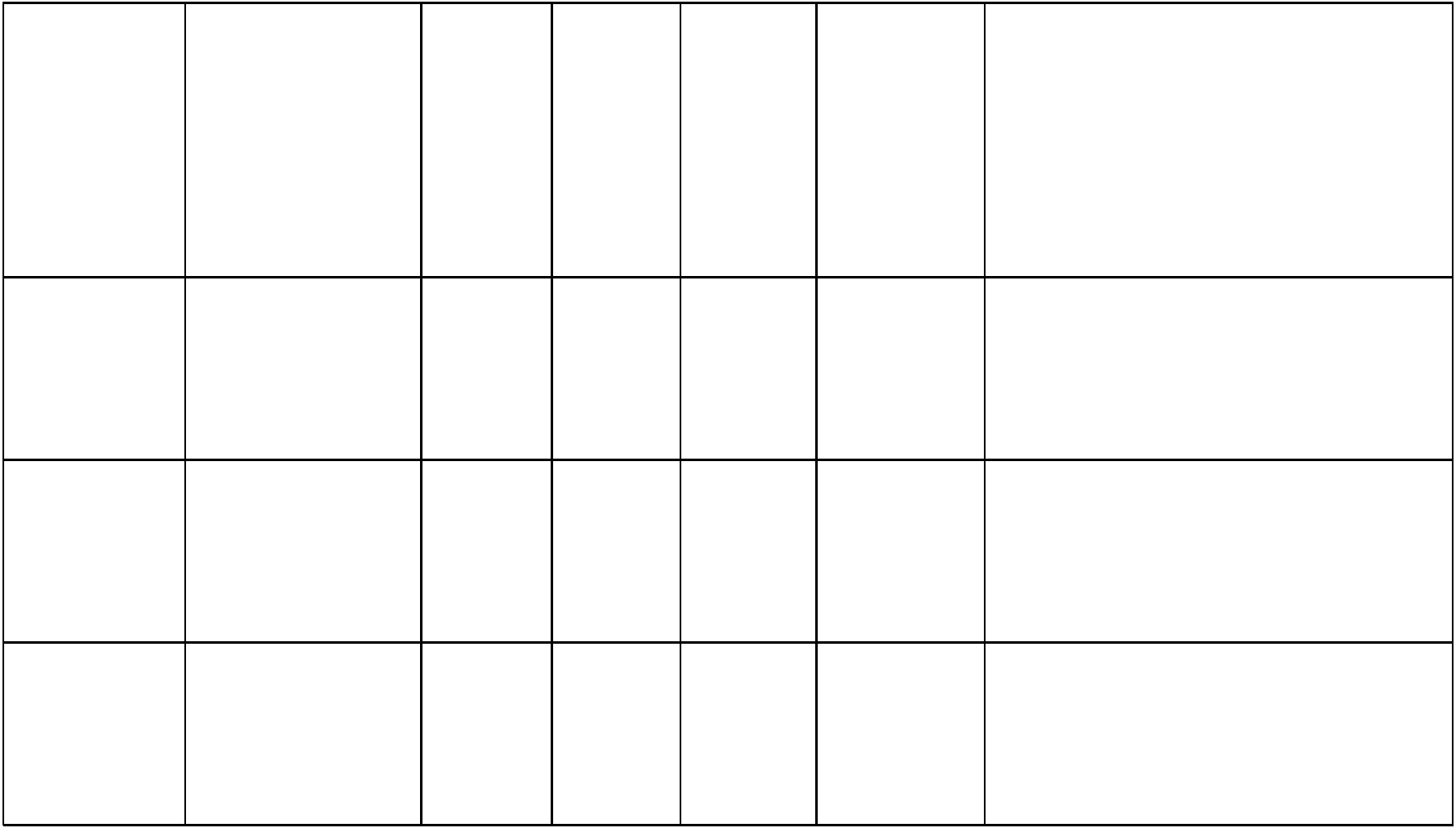


Center for

Public Health

Research,

National Center

for Disease

Control and

Public Health

(NCDC) of

Georgia.

Servicio de Microbiología. Consorcio

Hospital General Universitario de Valencia

Servicio de Microbiologia. Consorcio

Hospital General Universitario de Valencia

Servicio de Microbiología. Consorcio

Hospital General Universitario de Valencia

R. G. Lugar Center

for Public Health

Research, National

Center for Disease

Control and Public

Health (NCDC) of

Georgia.

Sequencing and Bioinformatics Service and Molecular Epidemiology Research Group. FISABIO-Public Health

Sequencing and Bioinformatics Service and Molecular Epidemiology Research Group. FISABIO-Public Health

Sequencing and Bioinformatics Service and Molecular Epidemiology Research Group. FISABIO-Public Health

Adam Kotorashvili, Marine Murtskhvaladze, Nato Kotaria, Ann Machablishvili, Lela Sabadze, Mari Gavashelidze, Ana Papkiauri, Meri Pantsulaia, Gvantsa Brachveli, Tata Imnadze, Tamar Jashiashvili, Tea Tevdoradze, Ketevan Sidamonidze, Ekaterine Khmaladze, Ekaterine Zhghenti, Roena Sukhiashvili, Mariam Zakalashvili, Lela Urushadze, Magda Dgebuadze, Giorgi Tomashvili, Davit Tsaguria, Ekaterine Zangaladze, Nino Berishvili, Gvantsa Chanturia, Maia Alkhazashvili, Irma Burjanadze, Anna Kasradze, Khatuna Zakhashvili, Paata Imnadze, Amiran Gamkrelidze.

Maria Dolores Ocete, Concepcion Gimeno, Giuseppe D'Auria, Griselda De Marco, Neris Garcia-Gonzalez, Maria Alma Bracho, Fernando Gonzalez-Candelas

Griselda De Marco, Neris Garcia-Gonzalez, Maria Alma

Bracho, Maria Dolores Ocete, Concepcion Gimeno,

Giuseppe D'Auria, Fernando Gonzalez-Candelas

Neris Garcia-Gonzalez, Maria Alma Bracho, Maria Dolores Ocete, Concepcion Gimeno, Giuseppe D'Auria, Griselda De Marco, Fernando Gonzalez-Candelas


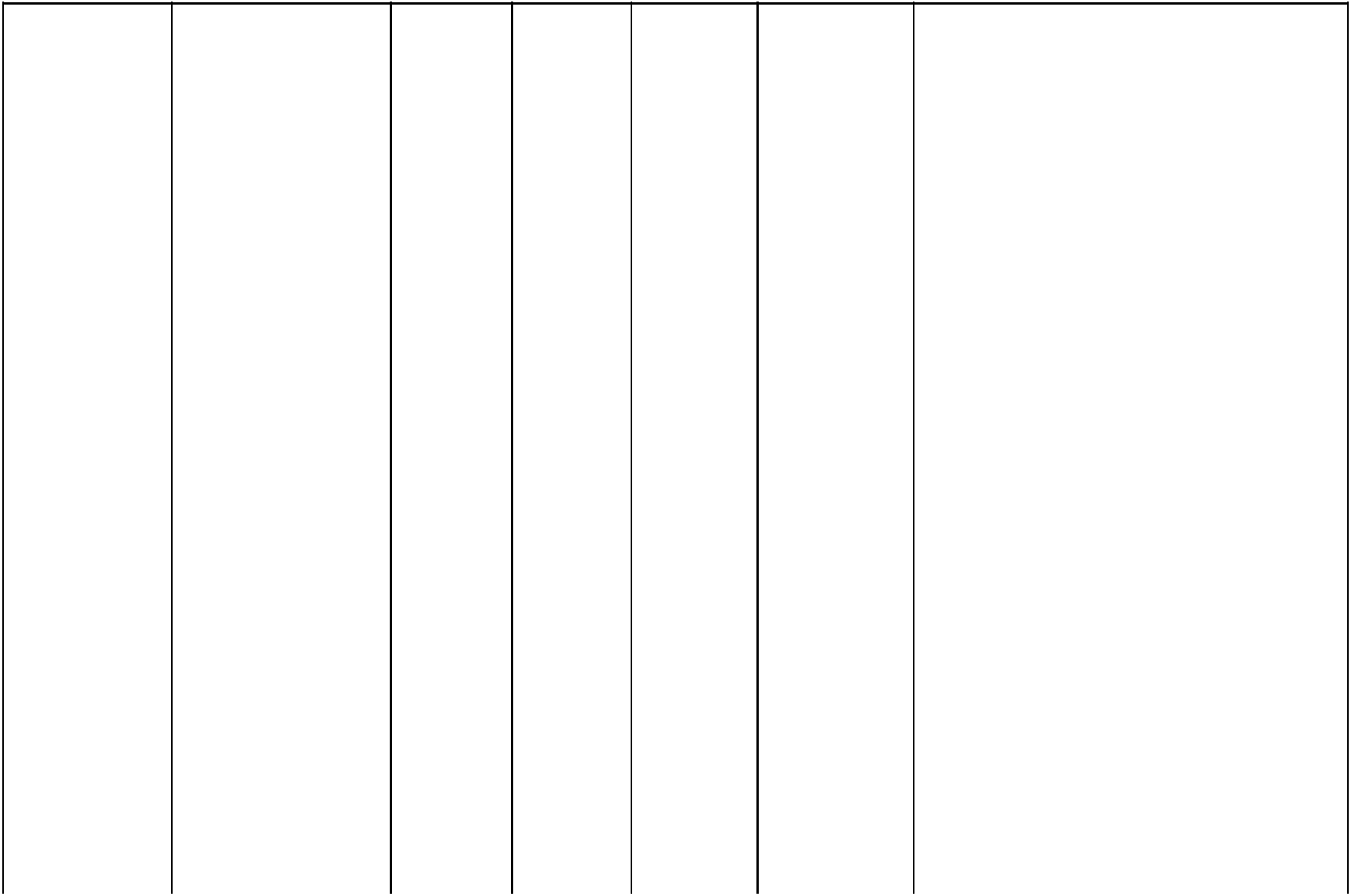


|  |  |  |  |  |  |  |  |  | Sequencing and |  |  |
| --- | --- | --- | --- | --- | --- | --- | --- | --- | --- | --- | --- |
|  |  |  |  | Europe / Spain |  |  | Servicio de |  | Bioinformatics |  |  |
|  |  |  |  |  |  |  | Microbiología. |  | Service and | Giuseppe D'Auria, Griselda De Marco, Neris Garcia- |  |
|  |  | hCoV- |  | / Comunitat |  |  | Consorcio |  | Molecular |  |  |
| EPI_ISL_416487 |  |  |  |  | 2020-03-04 | |  |  |  | Gonzalez, Maria Alma Bracho, Maria Dolores Ocete, |  |
|  |  | 19/Spain/Valencia8/2020 |  | Valenciana / |  |  | Hospital General |  | Epidemiology |  |  |
|  |  |  |  |  |  |  |  |  |  | Concepcion Gimeno, Fernando Gonzalez-Candelas |  |
|  |  |  |  | Valencia |  |  | Universitario de |  | Research Group. |  |  |
|  |  |  |  |  |  |  |  |  |  |  |  |
|  |  |  |  |  |  |  | Valencia |  | FISABIO-Public |  |  |
|  |  |  |  |  |  |  |  |  | Health |  |  |
|  |  |  |  |  |  |  |  |  |  |  |  |
|  |  |  |  |  |  |  | ViroGenetics - |  |  |  |  |
|  |  |  |  |  |  |  | BSL3 |  | ViroGenetics - BSL3 |  |  |
|  |  |  |  |  |  |  | Laboratory of |  |  |  |  |
|  |  |  |  |  |  |  |  |  | Laboratory of |  |  |
|  |  |  |  |  |  |  | Virology; Human |  |  |  |  |
|  |  |  |  |  |  |  |  |  | Virology; Human | Aleksandra Milewska, Ewelina Pośpiech, Agata Jarosz, |  |
|  |  |  |  |  |  |  | Genome |  |  |  |  |
|  |  |  |  |  |  |  |  |  | Genome Variation | Adrianna Klajmon, Kamila Marszałek, Katarzyna Pancer, |  |
|  |  |  |  | Europe / |  |  | Variation |  |  |  |  |
| EPI_ISL_416488 |  | hCoV-19/Poland/PL_P1/2020 |  |  | 2020-03-03 | |  |  | Research Group & | Magdalena Rzeczkowska, Tomasz Wołkowicz, Katarzyna |  |
|  |  |  |  | Poland / |  |  | Research Group |  |  |  |  |
|  |  |  |  |  |  |  |  |  | Genomics Centre | Zacharczuk, Agnieszka Kołakowska-Kulesza, Natalia |  |
|  |  |  |  | Zielonogorskie |  |  | & Genomics |  |  |  |  |
|  |  |  |  |  |  |  |  |  | MCB; Bioinformatics | Wolaniuk, Ewelina Hallman-Szelińska, Paweł P Łabaj, |  |
|  |  |  |  |  |  |  | Centre MCB; |  |  |  |  |
|  |  |  |  |  |  |  |  |  | Research Group | Wojciech Branicki, Krzysztof Pyrć |  |
|  |  |  |  |  |  |  | Bioinformatics |  |  |  |  |
|  |  |  |  |  |  |  |  |  | Department of |  |  |
|  |  |  |  |  |  |  | Research Group |  |  |  |  |
|  |  |  |  |  |  |  |  |  | Virology |  |  |
|  |  |  |  |  |  |  | Department of |  |  |  |  |
|  |  |  |  |  |  |  |  |  |  |  |  |
|  |  |  |  |  |  |  | Virology |  |  |  |  |
|  |  |  |  |  |  |  |  |  |  |  |  |
|  |  |  |  | Europe / |  |  | CH Jean de |  | National Reference |  |  |
|  |  |  |  |  |  |  |  |  | Center for Viruses of | Mélnie Albert, Marion Barbet, Sylvie Behillil, Méline Bizard, |  |
|  |  |  |  | France / Hauts |  |  | Navarre |  |  |  |  |
| EPI_ISL_416493 |  | hCoV-19/France/HF2196/2020 |  |  | 2020-03-08 | |  |  | Respiratory | Angela Brisebarre, Flora Donati, Etienne Simon-Lorière, |  |
|  |  |  |  | de France / |  |  | Laboratoire de |  | Infections, Institut | Vincent Enouf, Maud Vanpeene, Sylvie van der Werf |  |
|  |  |  |  | Château-Thierry |  |  | Biologie |  |  |  |  |
|  |  |  |  |  |  |  |  |  | Pasteur, Paris |  |  |
|  |  |  |  |  |  |  |  |  |  |  |  |
|  |  |  |  |  |  |  |  |  |  |  |  |
|  |  |  |  | Europe / |  |  | Centre Hositalier |  | National Reference | Mélnie Albert, Marion Barbet, Sylvie Behillil, Méline Bizard, |  |
|  |  |  |  |  |  |  | Universitaire de |  | Center for Viruses of |  |  |
|  |  |  |  | France / |  |  |  |  |  | Angela Brisebarre, Flora Donati, Etienne Simon-Lorière, |  |
| EPI_ISL_416494 |  | hCoV-19/France/N2223/2020 |  |  | 2020-03-04 | | Rouen |  | Respiratory |  |  |
|  |  |  |  | Normandie / |  |  |  |  |  | Vincent Enouf, Maud Vanpeene, Sylvie van der Werf, Jean- |  |
|  |  |  |  |  |  |  | Laboratoire de |  | Infections, Institut |  |  |
|  |  |  |  | Rouen |  |  |  |  |  | Christophe Plantier |  |
|  |  |  |  |  |  |  | Virologie |  | Pasteur, Paris |  |  |
|  |  |  |  |  |  |  |  |  |  |  |  |
|  |  |  |  |  |  |  |  |  |  |  |  |
|  |  |  |  | Europe / |  |  | Centre |  | National Reference | Mélnie Albert, Marion Barbet, Sylvie Behillil, Méline Bizard, |  |
|  |  |  |  |  |  |  | Hospitalier |  | Center for Viruses of |  |  |
|  |  |  |  | France / Hauts |  |  |  |  |  | Angela Brisebarre, Flora Donati, Etienne Simon-Lorière, |  |
| EPI_ISL_416495 |  | hCoV-19/France/HF2234/2020 |  |  | 2020-03-10 | | Compiègne |  | Respiratory |  |  |
|  |  |  |  | de France / |  |  | Laboratoire de |  | Infections, Institut | Vincent Enouf, Maud Vanpeene, Sylvie van der Werf, Raulin |  |
|  |  |  |  | Compiègne |  |  |  |  |  | Olivia |  |
|  |  |  |  |  |  |  | Biologie |  | Pasteur, Paris |  |  |
|  |  |  |  |  |  |  |  |  |  |  |  |
|  |  |  |  |  |  |  |  |  |  |  |  |

EPI_ISL_416496

EPI_ISL_416497

EPI_ISL_416498

EPI_ISL_416499

EPI_ISL_416706

EPI_ISL_416710

EPI_ISL_416745

EPI_ISL_416746

hCoV-19/France/HF2237/2020

hCoV-19/France/HF2239/2020

hCoV-

19/France/IDF2256/2020

hCoV-

19/France/IDF2278/2020

hCoV-19/USA/WA-

UW168/2020

hCoV-19/USA/WA-

UW172/2020

hCoV-

19/France/Pollionay_1733/202

0

hCoV-

19/France/Valence_425/2020

Europe / France / Hauts de France / Compiègne

Europe / France / Hauts de France / Compiègne

Europe / France / Ile de France / Garches

Europe / France / Ile de France / Longjumeau

North America /

USA /

Washington

North America /

USA /

Washington

Europe /

France / ARA

Europe /

France / ARA

2020-03-10

2020-03-10

2020-03-11

2020-03-11

2020-03-13

2020-03-13

2020-03-10

2020-03-03

Centre


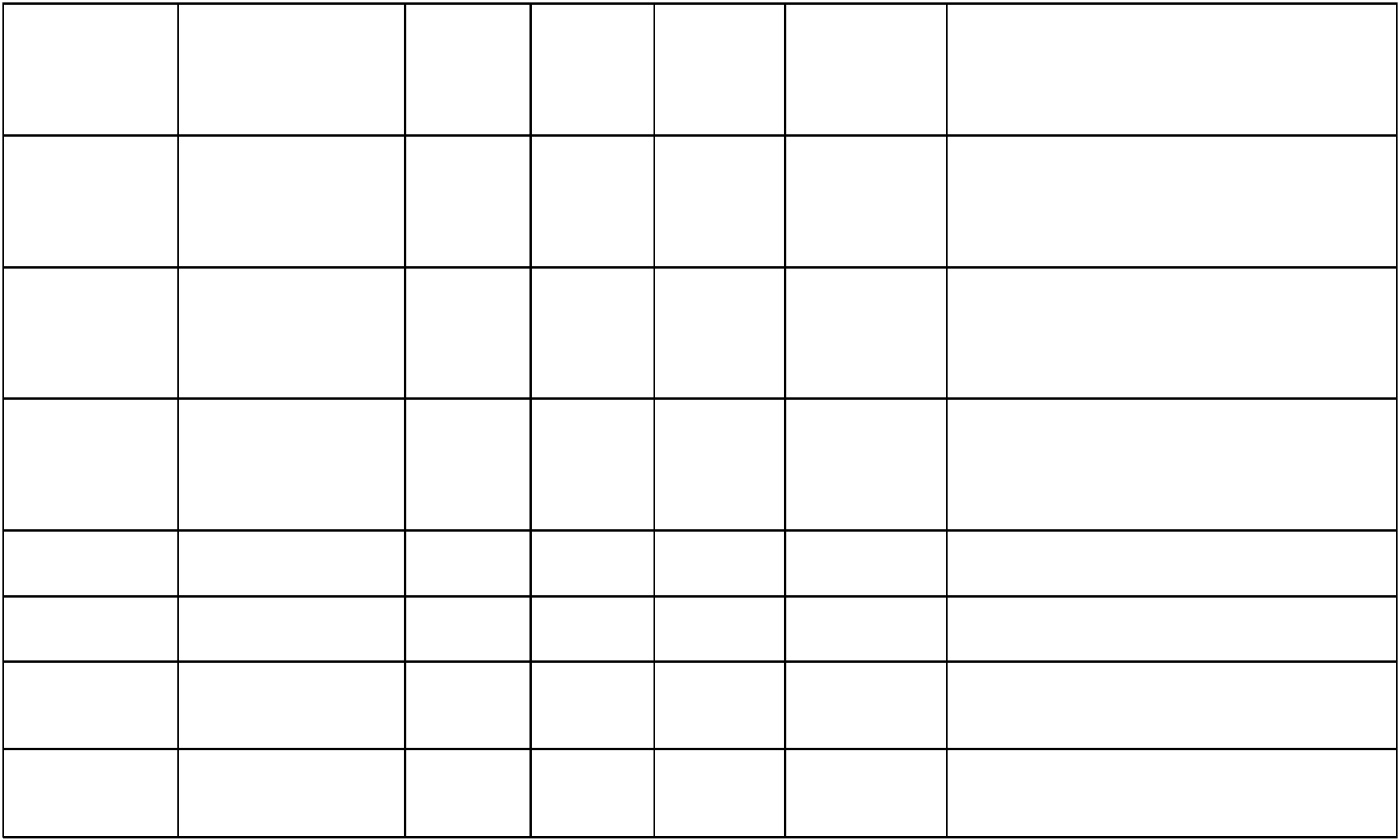


Hospitalier

Compiègne

Laboratoire de

Biologie

Centre

Hospitalier

Compiègne

Laboratoire de

Biologie

Institut Médico légal- Hop R. Poincaré

LABM GH nord Essonne

UW Virology Lab

UW Virology Lab

CNR Virus des Infections Respiratoires - France SUD

CNR Virus des

Infections

Respiratoires - France SUD

National Reference Center for Viruses of Respiratory Infections, Institut Pasteur, Paris

National Reference Center for Viruses of Respiratory Infections, Institut Pasteur, Paris

National Reference Center for Viruses of Respiratory Infections, Institut Pasteur, Paris

National Reference Center for Viruses of Respiratory Infections, Institut Pasteur, Paris

UW Virology Lab

UW Virology Lab

CNR Virus des Infections Respiratoires - France SUD

CNR Virus des

Infections

Respiratoires -

France SUD

Mélnie Albert, Marion Barbet, Sylvie Behillil, Méline Bizard, Angela Brisebarre, Flora Donati, Etienne Simon-Lorière, Vincent Enouf, Maud Vanpeene, Sylvie van der Werf, Raulin Olivia

Mélnie Albert, Marion Barbet, Sylvie Behillil, Méline Bizard, Angela Brisebarre, Flora Donati, Etienne Simon-Lorière, Vincent Enouf, Maud Vanpeene, Sylvie van der Werf, Raulin Olivia

Mélnie Albert, Marion Barbet, Sylvie Behillil, Méline Bizard, Angela Brisebarre, Flora Donati, Etienne Simon-Lorière, Vincent Enouf, Maud Vanpeene, Sylvie van der Werf

Mélnie Albert, Marion Barbet, Sylvie Behillil, Méline Bizard, Angela Brisebarre, Flora Donati, Etienne Simon-Lorière, Vincent Enouf, Maud Vanpeene, Sylvie van der Werf

Pavitra Roychoudhury, Hong Xie, Keith Jerome, Alexander Greninger

Pavitra Roychoudhury, Hong Xie, Keith Jerome, Alexander Greninger

Bal, Antonin; Destras, Gregory; Gaymard, Alexandre; Bouscambert-Duchamp, Maude; Cheynet, Valérie; Brengel-Pesce, Karen; Morfin-Sherpa, Florence; Valette, Martine; Josset, Laurence; Lina, Bruno.

Bal, Antonin; Destras, Gregory; Gaymard, Alexandre; Bouscambert-Duchamp, Maude; Cheynet, Valérie; Brengel-Pesce, Karen; Morfin-Sherpa, Florence; Valette, Martine; Josset, Laurence; Lina, Bruno.

EPI_ISL_416747

EPI_ISL_416748

EPI_ISL_416749

EPI_ISL_416751

EPI_ISL_416752

EPI_ISL_416753

EPI_ISL_416754

EPI_ISL_416756

EPI_ISL_416757

hCoV-

19/France/Lyon_487/2020

hCoV-

19/France/Lyon_508/2020

hCoV-

19/France/Valence_532/2020

hCoV-19/France/Clermont-

Ferrand_651/2020

hCoV-19/France/Clermont-

Ferrand_650/2020

hCoV-19/France/Lyon_06464

/2020

hCoV-

19/France/Lyon_06487/2020

hCoV-

19/France/Lyon_06531/2020

hCoV-19/France/Bourg-en-

Bresse_06678/2020

Europe /

France / ARA

Europe /

France / ARA

Europe /

France / ARA

Europe /

France / ARA

Europe /

France / ARA

Europe /

France / ARA

Europe /

France / ARA

Europe /

France / ARA

Europe /

France / ARA

2020-03-04

2020-03-04

2020-03-04

2020-03-05

2020-03-04

2020-03-06

2020-03-06

2020-03-06

2020-03-07

Institut des


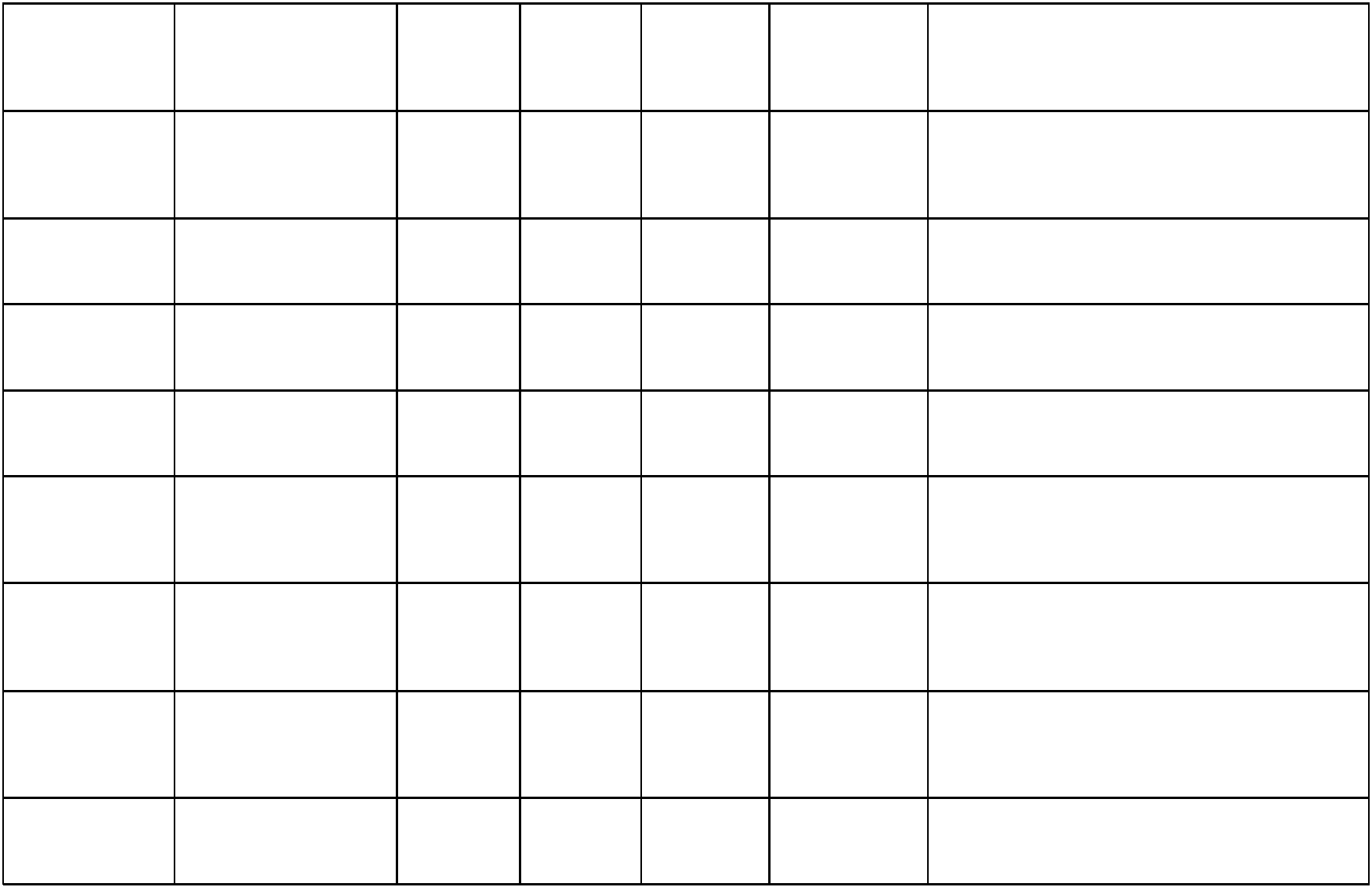


Agents

Infectieux (IAI) Hospices Civils de Lyon

Institut des

Agents

Infectieux (IAI) Hospices Civils de Lyon

Centre

Hospitalier de

Valence

CHU Gabriel

Montpied

CHU Gabriel

Montpied

Institut des

Agents

Infectieux (IAI) Hospices Civils de Lyon

Institut des

Agents

Infectieux (IAI) Hospices Civils de Lyon

Institut des

Agents

Infectieux (IAI) Hospices Civils de Lyon

Centre

Hospitalier de

Bourg en Bresse

CNR Virus des Infections Respiratoires - France SUD

CNR Virus des Infections Respiratoires - France SUD

CNR Virus des

Infections

Respiratoires - France SUD

CNR Virus des Infections Respiratoires - France SUD

CNR Virus des Infections Respiratoires - France SUD

CNR Virus des Infections Respiratoires - France SUD

CNR Virus des Infections Respiratoires - France SUD

CNR Virus des Infections Respiratoires - France SUD

CNR Virus des Infections Respiratoires - France SUD

Bal, Antonin; Destras, Gregory; Gaymard, Alexandre; Bouscambert-Duchamp, Maude; Cheynet, Valérie; Brengel-Pesce, Karen; Morfin-Sherpa, Florence; Valette, Martine; Josset, Laurence; Lina, Bruno.

Bal, Antonin; Destras, Gregory; Gaymard, Alexandre; Bouscambert-Duchamp, Maude; Cheynet, Valérie; Brengel-Pesce, Karen; Morfin-Sherpa, Florence; Valette, Martine; Josset, Laurence; Lina, Bruno.

Bal, Antonin; Destras, Gregory; Gaymard, Alexandre; Bouscambert-Duchamp, Maude; Cheynet, Valérie; Brengel-Pesce, Karen; Morfin-Sherpa, Florence; Valette, Martine; Josset, Laurence; Lina, Bruno.

Bal, Antonin; Destras, Gregory; Gaymard, Alexandre; Bouscambert-Duchamp, Maude; Cheynet, Valérie; Brengel-Pesce, Karen; Morfin-Sherpa, Florence; Valette, Martine; Josset, Laurence; Lina, Bruno.

Bal, Antonin; Destras, Gregory; Gaymard, Alexandre; Bouscambert-Duchamp, Maude; Cheynet, Valérie; Brengel-Pesce, Karen; Morfin-Sherpa, Florence; Valette, Martine; Josset, Laurence; Lina, Bruno.

Bal, Antonin; Destras, Gregory; Gaymard, Alexandre; Bouscambert-Duchamp, Maude; Cheynet, Valérie; Brengel-Pesce, Karen; Morfin-Sherpa, Florence; Valette, Martine; Josset, Laurence; Lina, Bruno.

Bal, Antonin; Destras, Gregory; Gaymard, Alexandre; Bouscambert-Duchamp, Maude; Cheynet, Valérie; Brengel-Pesce, Karen; Morfin-Sherpa, Florence; Valette, Martine; Josset, Laurence; Lina, Bruno.

Bal, Antonin; Destras, Gregory; Gaymard, Alexandre; Bouscambert-Duchamp, Maude; Cheynet, Valérie; Brengel-Pesce, Karen; Morfin-Sherpa, Florence; Valette, Martine; Josset, Laurence; Lina, Bruno.

Bal, Antonin; Destras, Gregory; Gaymard, Alexandre; Bouscambert-Duchamp, Maude; Cheynet, Valérie; Brengel-Pesce, Karen; Morfin-Sherpa, Florence; Valette, Martine; Josset, Laurence; Lina, Bruno.

EPI_ISL_416758

EPI_ISL_417010

EPI_ISL_417012

EPI_ISL_417013

EPI_ISL_417014

EPI_ISL_417015

EPI_ISL_417016

EPI_ISL_417017

EPI_ISL_417018

EPI_ISL_417019

EPI_ISL_417030

hCoV-

19/France/Lyon_0693/2020

hCoV-

19/Spain/Madrid201442/2020

hCoV-19/Belgium/ULG-

3843/2020

hCoV-19/Belgium/ULG-

4163/2020

hCoV-19/Belgium/ULG-

6216/2020

hCoV-19/Belgium/ULG-

6457/2020

hCoV-19/Belgium/ULG-

6503/2020

hCoV-19/Belgium/ULG-

6638/2020

hCoV-19/Belgium/ULG-

6670/2020

hCoV-19/Belgium/ULG-

6754/2020

hCoV-

19/Australia/NSW04/2020

Europe /

France / ARA

Europe / Spain

/ Madrid

Europe /

Belgium / Liège

Europe /

Belgium / Liège

Europe /

Belgium / Liège

Europe /

Belgium / Liège

Europe /

Belgium / Liège

Europe /

Belgium / Liège

Europe /

Belgium / Liège

Europe /

Belgium / Liège

Oceania / Australia / New South Wales / Sydney

2020-03-08

2020-03-04

2020-03-08

2020-03-09

2020-03-13

2020-03-13

2020-03-13

2020-03-14

2020-03-14

2020-03-14

2020-01-24

Institut des


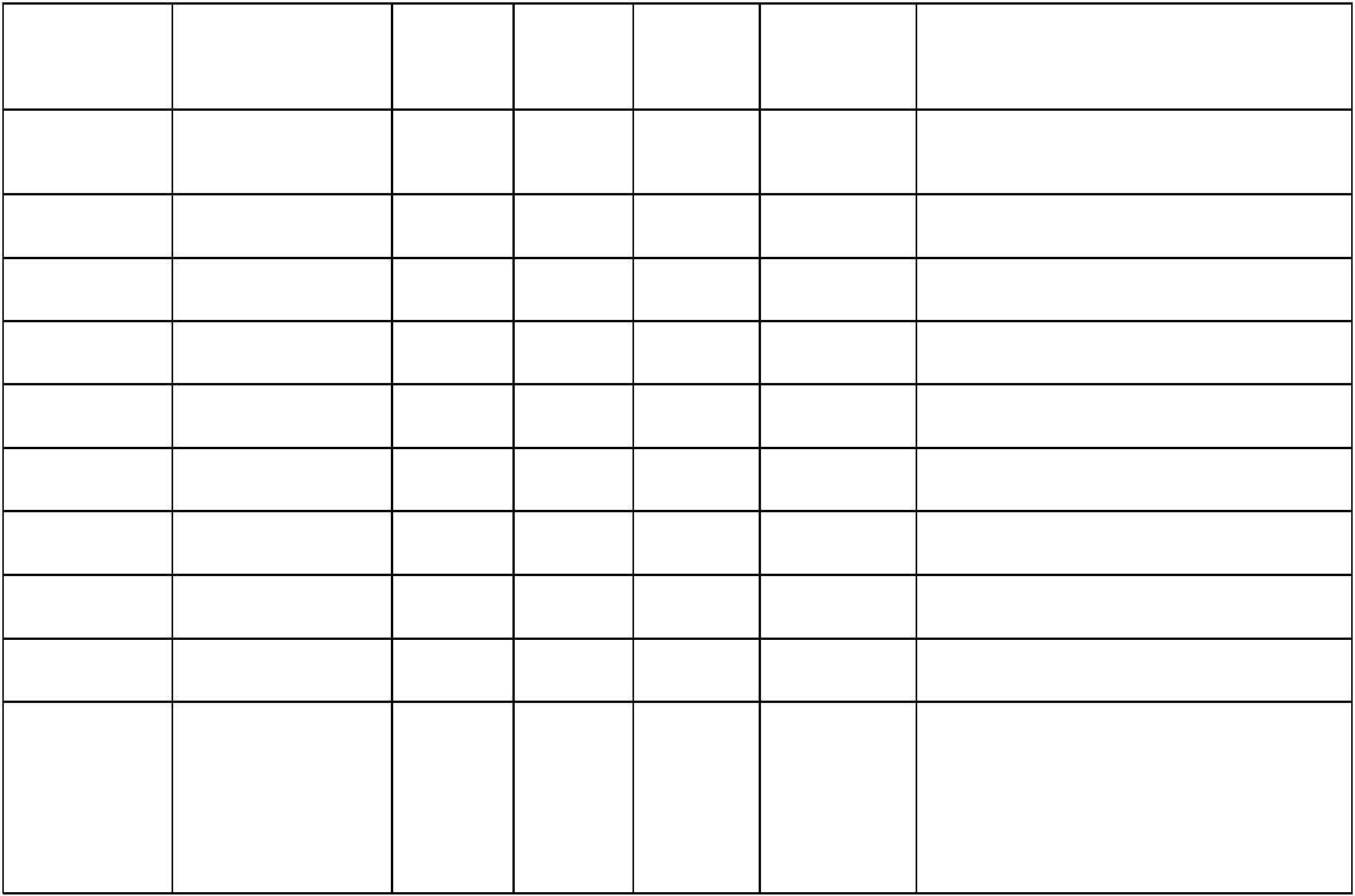


Agents

Infectieux (IAI) Hospices Civils de Lyon

FUNDACION JIMENEZ DIAZ

Department of

Clinical

Microbiology

Department of Clinical Microbiology

Department of Clinical Microbiology

Department of Clinical Microbiology

Department of Clinical Microbiology

Department of Clinical Microbiology

Department of Clinical Microbiology

Department of Clinical Microbiology

Centre for Infectious Diseases and Microbiology Laboratory Services

CNR Virus des Infections Respiratoires - France SUD

Instituto de Salud Carlos III

GIGA Medical

Genomics

GIGA Medical

Genomics

GIGA Medical

Genomics

GIGA Medical

Genomics

GIGA Medical

Genomics

GIGA Medical

Genomics

GIGA Medical

Genomics

GIGA Medical

Genomics

NSW Health

Pathology - Institute

of Clinical Pathology

and Medical

Research;

Westmead Hospital;

University of Sydney

Bal, Antonin; Destras, Gregory; Gaymard, Alexandre; Bouscambert-Duchamp, Maude; Cheynet, Valérie; Brengel-Pesce, Karen; Morfin-Sherpa, Florence; Valette, Martine; Josset, Laurence; Lina, Bruno.

Iglesias-Caballero, M. Molinero Calamita, M. González-Esguevillas, M. Camarero, S. Pozo, F. Casas, I. Jiménez, P. Jiménez, M. Zaballos, A. Monzón, S. Varona, S. Juliá, M. Cuesta, I. Fernández Roblas, R.

Durkin Keith, Artesi Maria, Bontems Sébastien, Boreux Raphaël, Meex Cécile, Melin Pierrette, Hayette Marie-Pierre, Bours Vincent.

Durkin Keith, Artesi Maria, Bontems Sébastien, Boreux Raphaël, Meex Cécile, Melin Pierrette, Hayette Marie-Pierre, Bours Vincent.

Durkin Keith, Artesi Maria, Bontems Sébastien, Boreux Raphaël, Meex Cécile, Melin Pierrette, Hayette Marie-Pierre, Bours Vincent.

Durkin Keith, Artesi Maria, Bontems Sébastien, Boreux Raphaël, Meex Cécile, Melin Pierrette, Hayette Marie-Pierre, Bours Vincent.

Durkin Keith, Artesi Maria, Bontems Sébastien, Boreux Raphaël, Meex Cécile, Melin Pierrette, Hayette Marie-Pierre, Bours Vincent.

Durkin Keith, Artesi Maria, Bontems Sébastien, Boreux Raphaël, Meex Cécile, Melin Pierrette, Hayette Marie-Pierre, Bours Vincent.

Durkin Keith, Artesi Maria, Bontems Sébastien, Boreux Raphaël, Meex Cécile, Melin Pierrette, Hayette Marie-Pierre, Bours Vincent.

Durkin Keith, Artesi Maria, Bontems Sébastien, Boreux Raphaël, Meex Cécile, Melin Pierrette, Hayette Marie-Pierre, Bours Vincent.

Eden J-S, Rockett R, Carter I, Rahman H, Holmes EC, O’Sullivan MV, Sintchenko V, Chen SC, Maddocks S, Kok J and Dwyer DE for the 2019-nCoV Study Group*

EPI_ISL_417031

EPI_ISL_417032

EPI_ISL_417033

EPI_ISL_417069

EPI_ISL_417072

EPI_ISL_417076

EPI_ISL_417085

hCoV-

19/Australia/QLDID919/2020

hCoV-

19/Australia/QLDID920/2020

hCoV-

19/Australia/QLDID921/2020

hCoV-19/USA/WA-S16/2020

hCoV-19/USA/WA-S19/2020

hCoV-19/USA/WA-S23/2020

hCoV-19/USA/WA-S32/2020

Oceania / Australia / Queensland / Gold Coast

Oceania / Australia / Queensland / Rockhampton

Oceania / Australia / Queensland / Brisbane

North America /

USA /

Washington

North America /

USA /

Washington /

King County

North America /

USA /

Washington /

Snohomish

County

North America /

USA /

Washington /

Snohomish

County

2020-03-11

2020-03-11

2020-03-11

2020-03-03

2020-03-02

2020-03-02

2020-03-02

Pathology Queensland


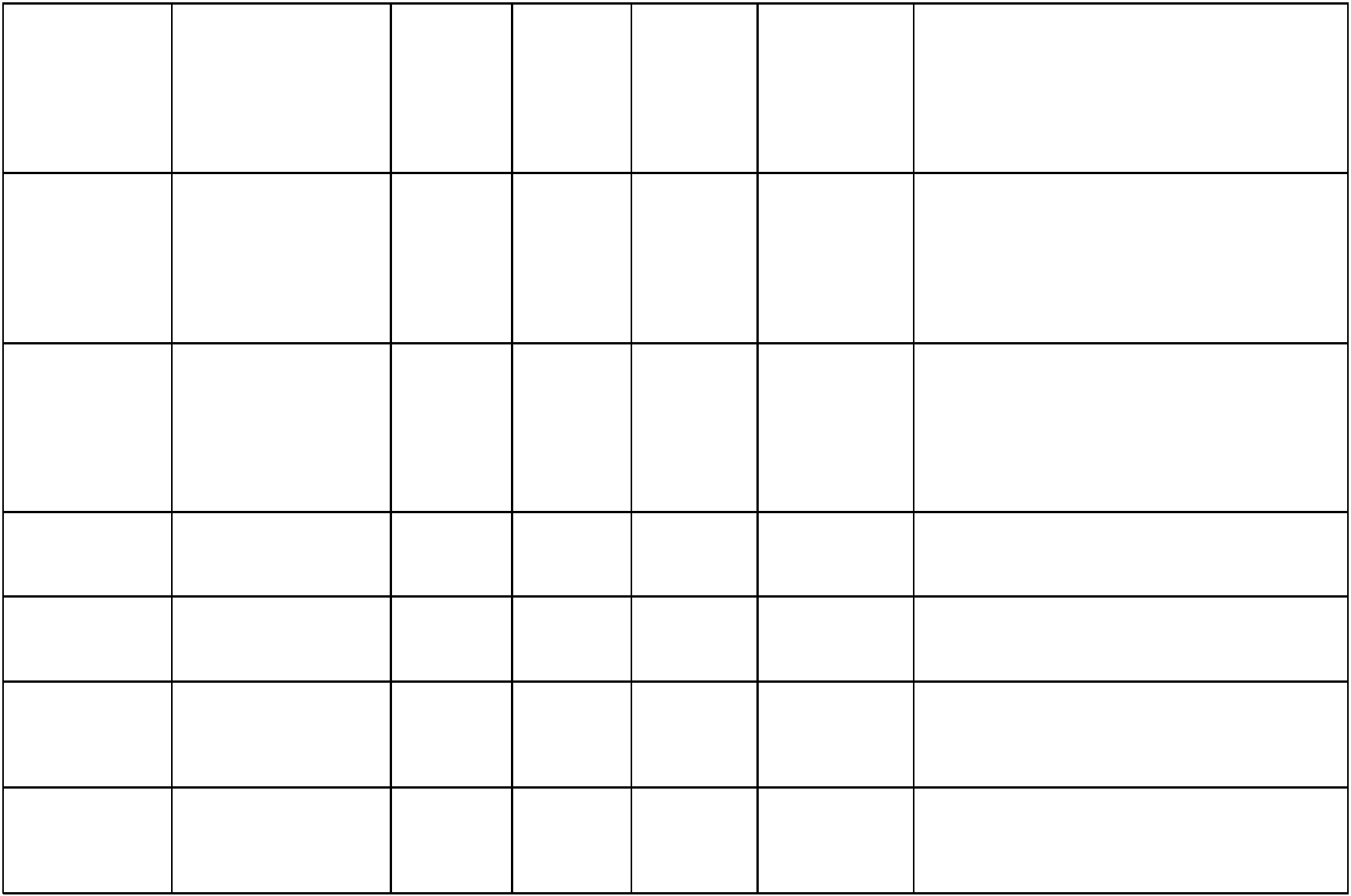


Rockhampton Base Hospital

Sullivan

Nicolaides

Pathology

Washington

State

Department of

Health

Washington

State

Department of

Health

Washington

State

Department of

Health

Washington

State

Department of

Health

Public Health

Virology Laboratory

Public Health

Virology Laboratory

Public Health

Virology Laboratory

Seattle Flu Study

Seattle Flu Study

Seattle Flu Study

Seattle Flu Study

Bixing Huang, Alyssa Pyke, Amanda De Jong, Andrew Van Den Hurk, Carmel Taylor, David Warrilow, Doris Genge, Elisabeth Gamez, Glen Hewitson, Ian Maxwell Mackay, Inga Sultana, Jamie McMahon, Jean Barcelon, Judy Northill, Mitchell Finger, Natalie Simpson, Neelima Nair, Peter Burtonclay, Peter Moore, Sarah Wheatley, Sean Moody, Sonja Hall-Mendelin, Timothy Gardam, and Frederick Moore

Bixing Huang, Alyssa Pyke, Amanda De Jong, Andrew Van Den Hurk, Carmel Taylor, David Warrilow, Doris Genge, Elisabeth Gamez, Glen Hewitson, Ian Maxwell Mackay, Inga Sultana, Jamie McMahon, Jean Barcelon, Judy Northill, Mitchell Finger, Natalie Simpson, Neelima Nair, Peter Burtonclay, Peter Moore, Sarah Wheatley, Sean Moody, Sonja Hall-Mendelin, Timothy Gardam, and Frederick Moore

Bixing Huang, Alyssa Pyke, Amanda De Jong, Andrew Van Den Hurk, Carmel Taylor, David Warrilow, Doris Genge, Elisabeth Gamez, Glen Hewitson, Ian Maxwell Mackay, Inga Sultana, Jamie McMahon, Jean Barcelon, Judy Northill, Mitchell Finger, Natalie Simpson, Neelima Nair, Peter Burtonclay, Peter Moore, Sarah Wheatley, Sean Moody, Sonja Hall-Mendelin, Timothy Gardam, and Frederick Moore

Chu etl al

Chu etl al

Chu etl al

Chu etl al


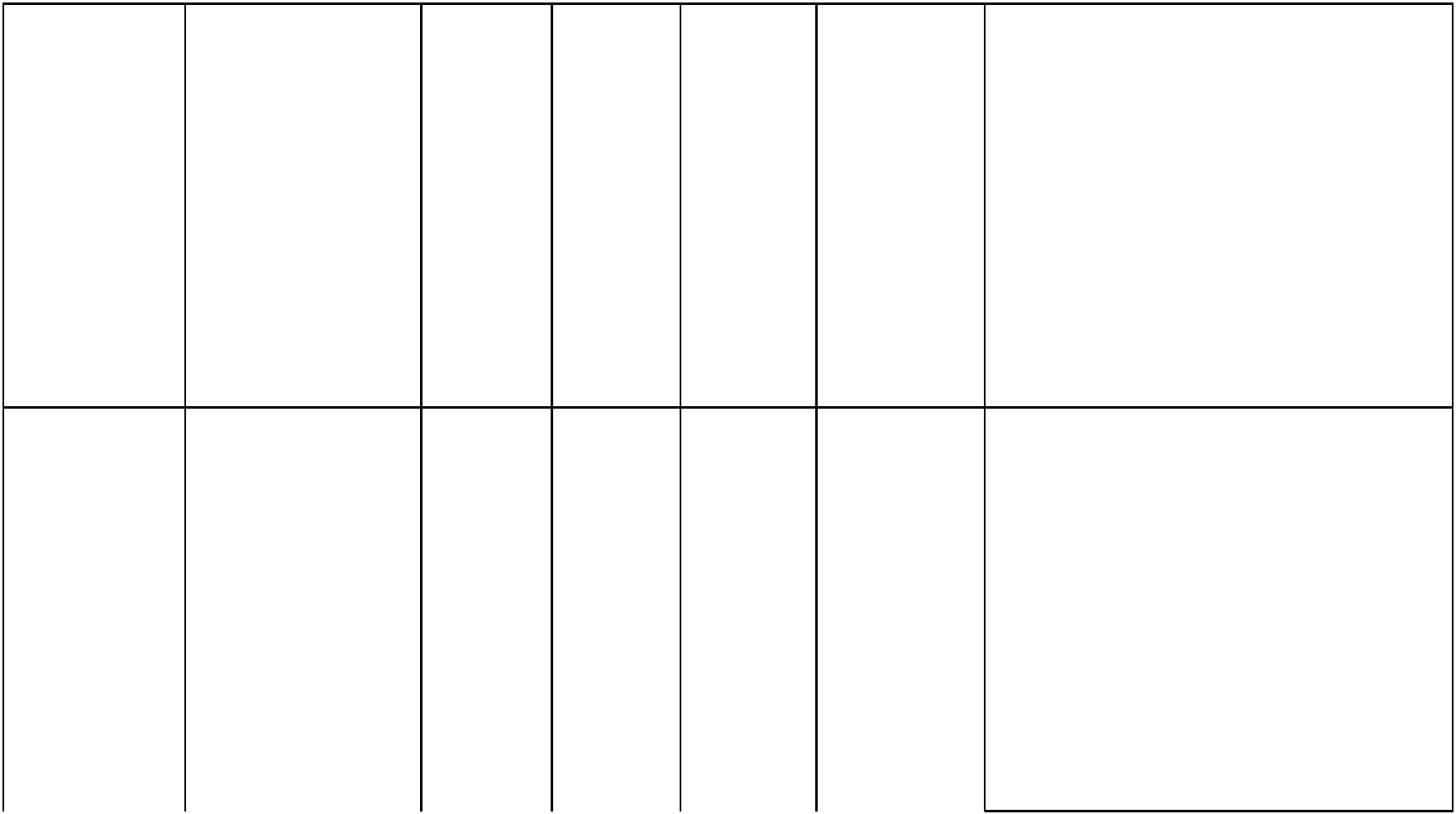


|  |  | Europe | / |  | The National |  |  |
| --- | --- | --- | --- | --- | --- | --- | --- |
|  |  |  |  |  | University |  |  |
| EPI_ISL_417721 | hCoV-19/Iceland/27/2020 | Iceland | / | 2020-03-03 |  | deCODE genetics |  |
|  |  |  |  |  | Hospital of |  |  |

| Reykjavik | Iceland |  |
| --- | --- | --- |
|  |  |

|  |  |  |  | Europe | / |  |  | The National |  |  |  |
| --- | --- | --- | --- | --- | --- | --- | --- | --- | --- | --- | --- |
|  |  |  |  |  |  |  |  | University |  |  |  |
| EPI_ISL_417741 |  | hCoV-19/Iceland/145/2020 |  | Iceland | / | 2020-03-13 | |  |  | deCODE genetics |  |
|  |  |  |  |  |  |  |  | Hospital of |  |  |  |
|  |  |  |  | Reykjavik | |  |  |  |  |  |  |
|  |  |  |  |  |  |  |  | Iceland |  |  |  |
|  |  |  |  |  |  |  |  |  |  |  |  |
|  |  |  |  |  |  |  |  |  |  |  |  |

Daniel F Gudbjartsson; Agnar Helgason; Hakon Jonsson; Olafur T Magnusson; Pall Melsted; Gudmundur L Norddahl; Jona Saemundsdottir; Asgeir Sigurdsson; Patrick Sulem; Arna B Agustsdottir; Berglind Eiriksdottir; Run Fridriksdottir; Elisabet E Gardarsdottir; Gudmundur Georgsson; Olafia S Gretarsdottir; Kjartan R Gudmundsson; Thora R Gunnarsdottir; Arnaldur Gylfason; Hilma Holm; Brynjar O Jensson; Aslaug Jonasdottir; Kamilla S Josefsdottir; Thordur Kristjansson; Droplaug N Magnusdottir; Louise le Roux; Gudrun Sigmundsdottir; Gardar Sveinbjornsson; Kristin E Sveinsdottir; Maney Sveinsdottir; Emil A Thorarensen; Bjarni Thorbjornsson; Gisli Masson; Ingileif Jonsdottir; Alma Moller; Thorolfur Gudnason; Karl G Kristinsson; Unnur Thorsteinsdottir; Kari Stefansson

Daniel F Gudbjartsson; Agnar Helgason; Hakon Jonsson; Olafur T Magnusson; Pall Melsted; Gudmundur L Norddahl; Jona Saemundsdottir; Asgeir Sigurdsson; Patrick Sulem; Arna B Agustsdottir; Berglind Eiriksdottir; Run Fridriksdottir; Elisabet E Gardarsdottir; Gudmundur Georgsson; Olafia S Gretarsdottir; Kjartan R Gudmundsson; Thora R Gunnarsdottir; Arnaldur Gylfason; Hilma Holm; Brynjar O Jensson; Aslaug Jonasdottir; Kamilla S Josefsdottir; Thordur Kristjansson; Droplaug N Magnusdottir; Louise le Roux; Gudrun Sigmundsdottir; Gardar Sveinbjornsson; Kristin E Sveinsdottir; Maney Sveinsdottir; Emil A Thorarensen; Bjarni Thorbjornsson; Gisli Masson; Ingileif Jonsdottir; Alma Moller; Thorolfur Gudnason; Karl G Kristinsson; Unnur Thorsteinsdottir; Kari Stefansson

|  |  | Europe | / |  | The National |  |  |
| --- | --- | --- | --- | --- | --- | --- | --- |
|  |  |  |  |  | University |  |  |
| EPI_ISL_417748 | hCoV-19/Iceland/109/2020 | Iceland | / | 2020-03-11 |  | deCODE genetics |  |
|  |  |  |  |  | Hospital of |  |  |

| Reykjavik | Iceland |  |
| --- | --- | --- |
|  |  |

|  |  |  |  | Europe | / |  |  | The National |  |  |  |
| --- | --- | --- | --- | --- | --- | --- | --- | --- | --- | --- | --- |
|  |  |  |  |  |  |  |  | University |  |  |  |
| EPI_ISL_417755 |  | hCoV-19/Iceland/169/2020 |  | Iceland | / | 2020-03-13 | |  |  | deCODE genetics |  |
|  |  |  |  |  |  |  |  | Hospital of |  |  |  |
|  |  |  |  | Reykjavik | |  |  |  |  |  |  |
|  |  |  |  |  |  |  |  | Iceland |  |  |  |
|  |  |  |  |  |  |  |  |  |  |  |  |
|  |  |  |  |  |  |  |  |  |  |  |  |

Daniel F Gudbjartsson; Agnar Helgason; Hakon Jonsson; Olafur T Magnusson; Pall Melsted; Gudmundur L Norddahl; Jona Saemundsdottir; Asgeir Sigurdsson; Patrick Sulem; Arna B Agustsdottir; Berglind Eiriksdottir; Run Fridriksdottir; Elisabet E Gardarsdottir; Gudmundur Georgsson; Olafia S Gretarsdottir; Kjartan R Gudmundsson; Thora R Gunnarsdottir; Arnaldur Gylfason; Hilma Holm; Brynjar O Jensson; Aslaug Jonasdottir; Kamilla S Josefsdottir; Thordur Kristjansson; Droplaug N Magnusdottir; Louise le Roux; Gudrun Sigmundsdottir; Gardar Sveinbjornsson; Kristin E Sveinsdottir; Maney Sveinsdottir; Emil A Thorarensen; Bjarni Thorbjornsson; Gisli Masson; Ingileif Jonsdottir; Alma Moller; Thorolfur Gudnason; Karl G Kristinsson; Unnur Thorsteinsdottir; Kari Stefansson

Daniel F Gudbjartsson; Agnar Helgason; Hakon Jonsson; Olafur T Magnusson; Pall Melsted; Gudmundur L Norddahl; Jona Saemundsdottir; Asgeir Sigurdsson; Patrick Sulem; Arna B Agustsdottir; Berglind Eiriksdottir; Run Fridriksdottir; Elisabet E Gardarsdottir; Gudmundur Georgsson; Olafia S Gretarsdottir; Kjartan R Gudmundsson; Thora R Gunnarsdottir; Arnaldur Gylfason; Hilma Holm; Brynjar O Jensson; Aslaug Jonasdottir; Kamilla S Josefsdottir; Thordur Kristjansson; Droplaug N Magnusdottir; Louise le Roux; Gudrun Sigmundsdottir; Gardar Sveinbjornsson; Kristin E Sveinsdottir; Maney Sveinsdottir; Emil A Thorarensen; Bjarni Thorbjornsson; Gisli Masson; Ingileif Jonsdottir; Alma Moller; Thorolfur Gudnason; Karl G Kristinsson; Unnur Thorsteinsdottir; Kari Stefansson

|  |  | Europe | / |  | The National |  |  |
| --- | --- | --- | --- | --- | --- | --- | --- |
|  |  |  |  |  | University |  |  |
| EPI_ISL_417770 | hCoV-19/Iceland/112/2020 | Iceland | / | 2020-03-11 |  | deCODE genetics |  |
|  |  |  |  |  | Hospital of |  |  |

| Reykjavik | Iceland |  |
| --- | --- | --- |
|  |  |

|  |  |  |  | Europe | / |  |  | The National |  |  |  |
| --- | --- | --- | --- | --- | --- | --- | --- | --- | --- | --- | --- |
|  |  |  |  |  |  |  |  | University |  |  |  |
| EPI_ISL_417785 |  | hCoV-19/Iceland/126/2020 |  | Iceland | / | 2020-03-12 | |  |  | deCODE genetics |  |
|  |  |  |  |  |  |  |  | Hospital of |  |  |  |
|  |  |  |  | Reykjavik | |  |  |  |  |  |  |
|  |  |  |  |  |  |  |  | Iceland |  |  |  |
|  |  |  |  |  |  |  |  |  |  |  |  |
|  |  |  |  |  |  |  |  |  |  |  |  |

Daniel F Gudbjartsson; Agnar Helgason; Hakon Jonsson; Olafur T Magnusson; Pall Melsted; Gudmundur L Norddahl; Jona Saemundsdottir; Asgeir Sigurdsson; Patrick Sulem; Arna B Agustsdottir; Berglind Eiriksdottir; Run Fridriksdottir; Elisabet E Gardarsdottir; Gudmundur Georgsson; Olafia S Gretarsdottir; Kjartan R Gudmundsson; Thora R Gunnarsdottir; Arnaldur Gylfason; Hilma Holm; Brynjar O Jensson; Aslaug Jonasdottir; Kamilla S Josefsdottir; Thordur Kristjansson; Droplaug N Magnusdottir; Louise le Roux; Gudrun Sigmundsdottir; Gardar Sveinbjornsson; Kristin E Sveinsdottir; Maney Sveinsdottir; Emil A Thorarensen; Bjarni Thorbjornsson; Gisli Masson; Ingileif Jonsdottir; Alma Moller; Thorolfur Gudnason; Karl G Kristinsson; Unnur Thorsteinsdottir; Kari Stefansson

Daniel F Gudbjartsson; Agnar Helgason; Hakon Jonsson; Olafur T Magnusson; Pall Melsted; Gudmundur L Norddahl; Jona Saemundsdottir; Asgeir Sigurdsson; Patrick Sulem; Arna B Agustsdottir; Berglind Eiriksdottir; Run Fridriksdottir; Elisabet E Gardarsdottir; Gudmundur Georgsson; Olafia S Gretarsdottir; Kjartan R Gudmundsson; Thora R Gunnarsdottir; Arnaldur Gylfason; Hilma Holm; Brynjar O Jensson; Aslaug Jonasdottir; Kamilla S Josefsdottir; Thordur Kristjansson; Droplaug N Magnusdottir; Louise le Roux; Gudrun Sigmundsdottir; Gardar Sveinbjornsson; Kristin E Sveinsdottir; Maney Sveinsdottir; Emil A Thorarensen; Bjarni Thorbjornsson; Gisli Masson; Ingileif Jonsdottir; Alma Moller; Thorolfur Gudnason; Karl G Kristinsson; Unnur Thorsteinsdottir; Kari Stefansson

|  |  | Europe | / |  | The National |  |  |
| --- | --- | --- | --- | --- | --- | --- | --- |
|  |  |  |  |  | University |  |  |
| EPI_ISL_417788 | hCoV-19/Iceland/133/2020 | Iceland | / | 2020-03-12 |  | deCODE genetics |  |
|  |  |  |  |  | Hospital of |  |  |

| Reykjavik | Iceland |  |
| --- | --- | --- |
|  |  |

|  |  |  |  | Europe | / |  |  | The National |  |  |  |
| --- | --- | --- | --- | --- | --- | --- | --- | --- | --- | --- | --- |
|  |  |  |  |  |  |  |  | University |  |  |  |
| EPI_ISL_417790 |  | hCoV-19/Iceland/139/2020 |  | Iceland | / | 2020-03-12 | |  |  | deCODE genetics |  |
|  |  |  |  |  |  |  |  | Hospital of |  |  |  |
|  |  |  |  | Reykjavik | |  |  |  |  |  |  |
|  |  |  |  |  |  |  |  | Iceland |  |  |  |
|  |  |  |  |  |  |  |  |  |  |  |  |
|  |  |  |  |  |  |  |  |  |  |  |  |

Daniel F Gudbjartsson; Agnar Helgason; Hakon Jonsson; Olafur T Magnusson; Pall Melsted; Gudmundur L Norddahl; Jona Saemundsdottir; Asgeir Sigurdsson; Patrick Sulem; Arna B Agustsdottir; Berglind Eiriksdottir; Run Fridriksdottir; Elisabet E Gardarsdottir; Gudmundur Georgsson; Olafia S Gretarsdottir; Kjartan R Gudmundsson; Thora R Gunnarsdottir; Arnaldur Gylfason; Hilma Holm; Brynjar O Jensson; Aslaug Jonasdottir; Kamilla S Josefsdottir; Thordur Kristjansson; Droplaug N Magnusdottir; Louise le Roux; Gudrun Sigmundsdottir; Gardar Sveinbjornsson; Kristin E Sveinsdottir; Maney Sveinsdottir; Emil A Thorarensen; Bjarni Thorbjornsson; Gisli Masson; Ingileif Jonsdottir; Alma Moller; Thorolfur Gudnason; Karl G Kristinsson; Unnur Thorsteinsdottir; Kari Stefansson

Daniel F Gudbjartsson; Agnar Helgason; Hakon Jonsson; Olafur T Magnusson; Pall Melsted; Gudmundur L Norddahl; Jona Saemundsdottir; Asgeir Sigurdsson; Patrick Sulem; Arna B Agustsdottir; Berglind Eiriksdottir; Run Fridriksdottir; Elisabet E Gardarsdottir; Gudmundur Georgsson; Olafia S Gretarsdottir; Kjartan R Gudmundsson; Thora R Gunnarsdottir; Arnaldur Gylfason; Hilma Holm; Brynjar O Jensson; Aslaug Jonasdottir; Kamilla S Josefsdottir; Thordur Kristjansson; Droplaug N Magnusdottir; Louise le Roux; Gudrun Sigmundsdottir; Gardar Sveinbjornsson; Kristin E Sveinsdottir; Maney Sveinsdottir; Emil A Thorarensen; Bjarni Thorbjornsson; Gisli Masson; Ingileif Jonsdottir; Alma Moller; Thorolfur Gudnason; Karl G Kristinsson; Unnur Thorsteinsdottir; Kari Stefansson

|  |  | Europe | / |  | The National |  |  |
| --- | --- | --- | --- | --- | --- | --- | --- |
|  |  |  |  |  | University |  |  |
| EPI_ISL_417791 | hCoV-19/Iceland/140/2020 | Iceland | / | 2020-03-12 |  | deCODE genetics |  |
|  |  |  |  |  | Hospital of |  |  |

| Reykjavik | Iceland |  |
| --- | --- | --- |
|  |  |

|  |  | Europe | / |  | The National |  |  |
| --- | --- | --- | --- | --- | --- | --- | --- |
|  |  |  |  |  | University |  |  |
| EPI_ISL_417798 | hCoV-19/Iceland/148/2020 | Iceland | / | 2020-03-13 |  | deCODE genetics |  |
|  |  |  |  |  | Hospital of |  |  |
|  |  | Reykjavik | |  |  |  |  |
|  |  |  |  |  | Iceland |  |  |
|  |  |  |  |  |  |  |  |

Daniel F Gudbjartsson; Agnar Helgason; Hakon Jonsson; Olafur T Magnusson; Pall Melsted; Gudmundur L Norddahl; Jona Saemundsdottir; Asgeir Sigurdsson; Patrick Sulem; Arna B Agustsdottir; Berglind Eiriksdottir; Run Fridriksdottir; Elisabet E Gardarsdottir; Gudmundur Georgsson; Olafia S Gretarsdottir; Kjartan R Gudmundsson; Thora R Gunnarsdottir; Arnaldur Gylfason; Hilma Holm; Brynjar O Jensson; Aslaug Jonasdottir; Kamilla S Josefsdottir; Thordur Kristjansson; Droplaug N Magnusdottir; Louise le Roux; Gudrun Sigmundsdottir; Gardar Sveinbjornsson; Kristin E Sveinsdottir; Maney Sveinsdottir; Emil A Thorarensen; Bjarni Thorbjornsson; Gisli Masson; Ingileif Jonsdottir; Alma Moller; Thorolfur Gudnason; Karl G Kristinsson; Unnur Thorsteinsdottir; Kari Stefansson

Daniel F Gudbjartsson; Agnar Helgason; Hakon Jonsson; Olafur T Magnusson; Pall Melsted; Gudmundur L Norddahl; Jona Saemundsdottir; Asgeir Sigurdsson; Patrick Sulem; Arna B Agustsdottir; Berglind Eiriksdottir; Run Fridriksdottir; Elisabet E Gardarsdottir; Gudmundur Georgsson; Olafia S Gretarsdottir; Kjartan R Gudmundsson; Thora R Gunnarsdottir; Arnaldur Gylfason; Hilma Holm; Brynjar O Jensson; Aslaug Jonasdottir; Kamilla S Josefsdottir; Thordur Kristjansson; Droplaug N Magnusdottir; Louise le Roux; Gudrun Sigmundsdottir; Gardar Sveinbjornsson; Kristin E Sveinsdottir; Maney Sveinsdottir; Emil A Thorarensen; Bjarni Thorbjornsson; Gisli Masson; Ingileif Jonsdottir; Alma Moller; Thorolfur Gudnason; Karl G Kristinsson; Unnur Thorsteinsdottir; Kari Stefansson

|  |  |  |  |  |  |  | Hospital |  |  | Elias Dahdouh, Sara González, Fernando Lázaro, Esther |  |
| --- | --- | --- | --- | --- | --- | --- | --- | --- | --- | --- | --- |
|  |  | hCoV- |  | Europe / Spain |  |  |  |  | Hospital | Viedma, Natalia Stella, Julio García, Juan Carlos Galán, |  |
| EPI_ISL_418182 |  |  |  |  | 2020-03-12 | | Universitario 12 |  |  |  |  |
|  |  | 19/Spain/Madrid_H8_37/2020 |  | / Madrid |  |  |  |  | Universitario La Paz | Rafael Cantón, Mª Dolores Folgueira, Rafael Delgado, Jesús |  |
|  |  |  |  |  |  |  | de Octubre |  |  |  |  |
|  |  |  |  |  |  |  |  |  |  | Mingorance |  |
|  |  |  |  |  |  |  |  |  |  |  |  |
|  |  |  |  |  |  |  |  |  |  |  |  |
| EPI_ISL_418206 |  | hCoV-19/Senegal/003/2020 |  | Africa / Senegal | 2020-02-28 | | Institut Pasteur |  | Institut Pasteur de | Ndongo Dia, Ousmane Faye, Amadou Alpha Sall |  |
|  |  |  |  | / Dakar |  |  | Dakar |  | Dakar |  |  |
|  |  |  |  |  |  |  |  |  |  |  |  |
|  |  |  |  |  |  |  |  |  |  |  |  |

EPI_ISL_418207

EPI_ISL_418208

EPI_ISL_418209

EPI_ISL_418210

EPI_ISL_418211

EPI_ISL_418212

EPI_ISL_418213

EPI_ISL_418216

EPI_ISL_418217

EPI_ISL_418218

EPI_ISL_418219

hCoV-19/Senegal/016/2020

hCoV-19/Senegal/020/2020

hCoV-19/Senegal/026/2020

hCoV-19/Senegal/073/2020

hCoV-19/Senegal/082/2020

hCoV-19/Senegal/087/2020

hCoV-19/Senegal/094/2020

hCoV-19/Senegal/136/2020

hCoV-19/Senegal/139/2020

hCoV-19/France/HF1465/2020

hCoV-19/France/B1623/2020

Africa / Senegal

/ Dakar

Africa / Senegal

/ Dakar

Africa / Senegal

/ Dakar

Africa / Senegal

/ Touba

Africa / Senegal

/ Touba

Africa / Senegal

/ Touba

Africa / Senegal

/ Touba

Africa / Senegal

/ Dakar

Africa / Senegal

/ Mbour

Europe / France / Hauts de France / Compiègne

Europe /

France /

Bretagne / Brest

2020-03-02

2020-03-04

2020-03-03

2020-03-10

2020-03-11

2020-03-11

2020-03-12

2020-03-13

2020-03-13

2020-02-21

2020-02-26

Institut Pasteur

Dakar

Institut Pasteur

Dakar

Institut Pasteur

Dakar

Institut Pasteur

Dakar

Institut Pasteur

Dakar

Institut Pasteur

Dakar

Institut Pasteur

Dakar

Institut Pasteur

Dakar

Institut Pasteur

Dakar

Centre

Hospitalier

Compiègne

Laboratoire de

Biologie

CHU - Hôpital

Cavale Blanche -

Labo. de

Virologie

Institut Pasteur de Dakar

Institut Pasteur de Dakar

Institut Pasteur de Dakar

Institut Pasteur de Dakar

Institut Pasteur de Dakar

Institut Pasteur de Dakar

Institut Pasteur de Dakar

Institut Pasteur de Dakar

Institut Pasteur de Dakar

National Reference Center for Viruses of Respiratory Infections, Institut Pasteur, Paris

National Reference Center for Viruses of Respiratory Infections, Institut Pasteur, Paris

Ndongo Dia, Ousmane Faye, Amadou Alpha Sall

Ndongo Dia, Ousmane Faye, Amadou Alpha Sall

Ndongo Dia, Ousmane Faye, Amadou Alpha Sall

Ndongo Dia, Ousmane Faye, Amadou Alpha Sall

Ndongo Dia, Ousmane Faye, Amadou Alpha Sall

Ndongo Dia, Ousmane Faye, Amadou Alpha sall

Ndongo Dia, Ousmane Faye, Amadou Alpha Sall

Ndongo Dia, Ousmane Faye, Amadou Alpha Sall

Ndongo Dia, Ousmane Faye, Amadou Alpha Sall

Mélanie Albert, Marion Barbet, Sylvie Behillil, Méline Bizard, Angela Brisebarre, Flora Donati, Fabiana Gambaro, Etienne Simon-Lorière, Vincent Enouf, Maud Vanpeene, Sylvie van der Werf, Raulin Olivia

Mélanie Albert, Marion Barbet, Sylvie Behillil, Méline Bizard, Angela Brisebarre, Flora Donati, Fabiana Gambaro, Etienne Simon-Lorière, Vincent Enouf, Maud Vanpeene, Sylvie van der Werf, Léa Pilorge

EPI_ISL_418220

EPI_ISL_418222

EPI_ISL_418223

EPI_ISL_418224

EPI_ISL_418225

EPI_ISL_418226

EPI_ISL_418227

hCoV-19/France/HF1645/2020

hCoV-

19/France/CVL2000/2020

hCoV-19/France/HF2060/2020

hCoV-19/France/HF2150/2020

hCoV-19/France/HF2155/2020

hCoV-19/France/HF2381/2020

hCoV-19/France/HF2393/2020

Europe / France / Hauts de France / Compiègne

Europe /

France / Centre-Val de Loire /

Tours

Europe / France / Hauts de France / Compiègne

Europe / France / Hauts de France / Compiègne

Europe / France / Hauts de France / Compiègne

Europe / France / Hauts

de France / Crouy en Thelle

Europe / France / Hauts de France / Compiègne

|  | Centre |  | National Reference |  |
| --- | --- | --- | --- | --- |
|  | Hospitalier |  | Center for Viruses of |  |
| 2020-02-28 | Compiègne |  | Respiratory |  |
|  | Laboratoire de |  | Infections, Institut |  |
|  | Biologie |  | Pasteur, Paris |  |
|  |  |  |  |  |
|  | CHRU |  | National Reference |  |
|  |  |  | Center for Viruses of |  |
|  | Bretonneau - |  |  |  |
| 2020-03-04 |  |  | Respiratory |  |
|  | Serv. Bacterio- |  |  |  |
|  |  |  | Infections, Institut |  |
|  | Virol. |  |  |  |
|  |  |  | Pasteur, Paris |  |
|  |  |  |  |  |
|  |  |  |  |  |
|  | Centre |  | National Reference |  |
|  | Hospitalier |  | Center for Viruses of |  |
| 2020-03-05 | Compiègne |  | Respiratory |  |
|  | Laboratoire de |  | Infections, Institut |  |
|  | Biologie |  | Pasteur, Paris |  |
|  |  |  |  |  |
|  | Centre |  | National Reference |  |
|  | Hospitalier |  | Center for Viruses of |  |
| 2020-03-08 | Compiègne |  | Respiratory |  |
|  | Laboratoire de |  | Infections, Institut |  |
|  | Biologie |  | Pasteur, Paris |  |
|  |  |  |  |  |
|  | Centre |  | National Reference |  |
|  | Hospitalier |  | Center for Viruses of |  |
| 2020-03-08 | Compiègne |  | Respiratory |  |
|  | Laboratoire de |  | Infections, Institut |  |
|  | Biologie |  | Pasteur, Paris |  |
|  |  |  |  |  |
|  | EHPAD - |  | National Reference |  |
|  |  |  | Center for Viruses of |  |
| 2020-03-09 | Résidences les |  | Respiratory |  |
|  | Cèdres |  | Infections, Institut |  |
|  |  |  | Pasteur, Paris |  |
|  |  |  |  |  |
|  | Centre |  | National Reference |  |
|  | Hospitalier |  | Center for Viruses of |  |
| 2020-03-12 | Compiègne |  | Respiratory |  |
|  | Laboratoire de |  | Infections, Institut |  |
|  | Biologie |  | Pasteur, Paris |  |
|  |  |  |  |  |

Mélanie Albert, Marion Barbet, Sylvie Behillil, Méline Bizard, Angela Brisebarre, Flora Donati, Fabiana Gambaro, Etienne Simon-Lorière, Vincent Enouf, Maud Vanpeene, Sylvie van der Werf, Raulin Olivia

Mélanie Albert, Marion Barbet, Sylvie Behillil, Méline Bizard, Angela Brisebarre, Flora Donati, Fabiana Gambaro, Etienne Simon-Lorière, Vincent Enouf, Maud Vanpeene, Sylvie van der Werf, Julien Marlet

Mélanie Albert, Marion Barbet, Sylvie Behillil, Méline Bizard, Angela Brisebarre, Flora Donati, Fabiana Gambaro, Etienne Simon-Lorière, Vincent Enouf, Maud Vanpeene, Sylvie van der Werf, Raulin Olivia

Mélanie Albert, Marion Barbet, Sylvie Behillil, Méline Bizard, Angela Brisebarre, Flora Donati, Fabiana Gambaro, Etienne Simon-Lorière, Vincent Enouf, Maud Vanpeene, Sylvie van der Werf, Raulin Olivia

Mélanie Albert, Marion Barbet, Sylvie Behillil, Méline Bizard, Angela Brisebarre, Flora Donati, Fabiana Gambaro, Etienne Simon-Lorière, Vincent Enouf, Maud Vanpeene, Sylvie van der Werf, Raulin Olivia

Mélanie Albert, Marion Barbet, Sylvie Behillil, Méline Bizard, Angela Brisebarre, Flora Donati, Etienne Simon-Lorière, Vincent Enouf, Maud Vanpeene, Sylvie van der Werf

Mélanie Albert, Marion Barbet, Sylvie Behillil, Méline Bizard, Angela Brisebarre, Flora Donati, Etienne Simon-Lorière, Vincent Enouf, Maud Vanpeene, Sylvie van der Werf, Raulin Olivia

EPI_ISL_418228

EPI_ISL_418229

EPI_ISL_418230

EPI_ISL_418234

EPI_ISL_418241

EPI_ISL_418242

EPI_ISL_418243

hCoV-19/France/HF2405/2020

hCoV-

19/France/IDF2410/2020

hCoV-

19/France/IDF2420/2020

hCoV-

19/France/IDF2534/2020

hCoV-

19/Algeria/G0638_2264/2020

hCoV-

19/Algeria/G0640_2265/2020

hCoV-

19/Spain/Andalucia201272/20

20

Europe / France / Hauts de France / Compiègne

Europe /

France / Ile de

France /

Levallois-Perret

Europe / France / Ile de France / Paris

Europe / France / Ile de France / Longjumeau

Africa / Algeria /

Boufarik

Africa / Algeria /

Blida

Europe / Spain

/ Andalusia

2020-03-12

2020-03-12

2020-03-13

2020-03-14

2020-03-02

2020-03-08

2020-02-28

Centre

Hospitalier

Compiègne

Laboratoire de

Biologie

Hopital franco britannique - Laboratoire

Clinique

AVERAY LA

BROUSTE,

Med. Polyvalente

LABM GH nord Essonne

NIC Viral

Respiratory Unit

- Institut Pasteur of Algeria

NIC Viral

Respiratory Unit

- Institut Pasteur of Algeria

HOSPITAL

UNIVERSITARI O VIRGEN DE LAS NIEVES

National Reference Center for Viruses of Respiratory Infections, Institut Pasteur, Paris

National Reference Center for Viruses of Respiratory Infections, Institut Pasteur, Paris

National Reference Center for Viruses of Respiratory Infections, Institut Pasteur, Paris

National Reference Center for Viruses of Respiratory Infections, Institut Pasteur, Paris

National Reference Center for Viruses of Respiratory Infections, Institut Pasteur, Paris

National Reference Center for Viruses of Respiratory Infections, Institut Pasteur, Paris

Instituto de Salud Carlos III

Mélanie Albert, Marion Barbet, Sylvie Behillil, Méline Bizard, Angela Brisebarre, Flora Donati, Etienne Simon-Lorière, Vincent Enouf, Maud Vanpeene, Sylvie van der Werf, Raulin Olivia

Mélanie Albert, Marion Barbet, Sylvie Behillil, Méline Bizard, Angela Brisebarre, Flora Donati, Etienne Simon-Lorière, Vincent Enouf, Maud Vanpeene, Sylvie van der Werf, Marianne Asso Bonnet

Mélanie Albert, Marion Barbet, Sylvie Behillil, Méline Bizard, Angela Brisebarre, Flora Donati, Etienne Simon-Lorière, Vincent Enouf, Maud Vanpeene, Sylvie van der Werf, Elsa Ngwem

Mélanie Albert, Marion Barbet, Sylvie Behillil, Méline Bizard, Angela Brisebarre, Flora Donati, Etienne Simon-Lorière, Vincent Enouf, Maud Vanpeene, Sylvie van der Werf, Christine Lambert

Mélanie Albert, Marion Barbet, Sylvie Behillil, Méline Bizard, Angela Brisebarre, Flora Donati, Etienne Simon-Lorière, Vincent Enouf, Maud Vanpeene, Sylvie van der Werf, Fawzi Derrar

Mélanie Albert, Marion Barbet, Sylvie Behillil, Méline Bizard, Angela Brisebarre, Flora Donati, Etienne Simon-Lorière, Vincent Enouf, Maud Vanpeene, Sylvie van der Werf, Fawzi Derrar

Iglesias-Caballero, M. Molinero Calamita, M. González-Esguevillas, M. Camarero, S. Pozo, F. Casas, I. Jiménez, P. Jiménez, M. Zaballos, A. Monzón, S. Varona, S. Juliá, M. Cuesta, I. Sanbonmatsu S.

EPI_ISL_418245

EPI_ISL_418246

EPI_ISL_418247

EPI_ISL_418248

EPI_ISL_418249

EPI_ISL_418251

EPI_ISL_418253

EPI_ISL_418255

EPI_ISL_418256

hCoV-

19/Spain/CastillaLaMancha20

1328/2020

hCoV-

19/Spain/CastillaLaMancha20

1329/2020

hCoV-

19/Spain/CastillayLeon201061

/2020

hCoV-

19/Spain/CastillayLeon201323

/2020

hCoV-

19/Spain/CastillayLeon201372

/2020

hCoV-

19/Spain/Madrid201105/2020

hCoV-

19/Spain/PaisVasco201382/20

20

hCoV-19/Italy/TE4925/2020

hCoV-19/Italy/TE4880/2020

Europe / Spain

- Castilla La Mancha

Europe / Spain

- Castilla-La Mancha

Europe / Spain / Castilla y Leon

Europe / Spain / Castilla y Leon

Europe / Spain / Castilla y Leon

Europe / Spain

/ Madrid

Europe / Spain

- Basque Country

Europe / Italy /

Abruzzo

Europe / Italy /

Abruzzo

2020-03-01

2020-03-01

2020-02-26

2020-03-01

2020-03-03

2020-02-25

2020-03-02

2020-03-14

2020-03-14

Hospital General

- Universitario de Guadalajara

Hospital General

- Universitario de Guadalajara

HOSPITAL GENERAL DE SEGOVIA

COMPLEJO ASISTENCIAL UNIVERSITARI O DE BURGOS

COMPLEJO ASISTENCIAL UNIVERSITARI O DE BURGOS

HOSPITAL

UNIVERSITARI

O LA PAZ

HOSPITAL

TXAGORRITXU

Presidio Ospedaliero "S. Spirito" - PESCARA

Ospedale “San Liberatore” di Atri

Instituto de Salud Carlos III

Instituto de Salud Carlos III

Instituto de Salud Carlos III

Instituto de Salud Carlos III

Instituto de Salud Carlos III

Instituto de Salud Carlos III

Instituto de Salud Carlos III

Istituto

Zooprofilattico

Sperimentale

dell'Abruzzo e Molise

"G. Caporale"

Istituto

Zooprofilattico

Sperimentale

dell'Abruzzo e Molise

"G. Caporale"

Iglesias-Caballero, M. Molinero Calamita, M. González-Esguevillas, M. Camarero, S. Pozo, F. Casas, I. Jiménez, P. Jiménez, M. Zaballos, A. Monzón, S. Varona, S. Juliá, M. Cuesta, I. Gonzalez-Praetorius A.

Iglesias-Caballero, M. Molinero Calamita, M. González-Esguevillas, M. Camarero, S. Pozo, F. Casas, I. Jiménez, P. Jiménez, M. Zaballos, A. Monzón, S. Varona, S. Juliá, M. Cuesta, I. Gonzalez-Praetorius A.

Iglesias-Caballero, M. Molinero Calamita, M. González-Esguevillas, M. Camarero, S. Pozo, F. Casas, I. Jiménez, P. Jiménez, M. Zaballos, A. Monzón, S. Varona, S. Juliá, M. Cuesta, I. Hernando-Real S.

Iglesias-Caballero, M. Molinero Calamita, M. González-Esguevillas, M. Camarero, S. Pozo, F. Casas, I. Jiménez, P. Jiménez, M. Zaballos, A. Monzón, S. Varona, S. Juliá, M. Cuesta, I. Megias-Lobon G.

Iglesias-Caballero, M. Molinero Calamita, M. González-Esguevillas, M. Camarero, S. Pozo, F. Casas, I. Jiménez, P. Jiménez, M. Zaballos, A. Monzón, S. Varona, S. Juliá, M. Cuesta, I. Megias-Lobon G.

Iglesias-Caballero, M. Molinero Calamita, M. González-Esguevillas, M. Camarero, S. Pozo, F. Casas, I. Jiménez, P. Jiménez, M. Zaballos, A. Monzón, S. Varona, S. Juliá, M. Cuesta, I. Romero P.

Iglesias-Caballero, M. Molinero Calamita, M. González-Esguevillas, M. Camarero, S. Pozo, F. Casas, I. Jiménez, P. Jiménez, M. Zaballos, A. Monzón, S. Varona, S. Juliá, M. Cuesta, I. Gomez-Gonzalez C.

Lorusso A, Marcacci M, Cammà C, Monaco F, Puglia I, Di Pasquale A, Rinaldi A, Mangone I, Savini G

Lorusso A, Marcacci M, Di Domenico M, Puglia I, Curini V, Ancora M, Di Pasquale A, Rinaldi A, Mangone I, Cammà C, Savini G.

EPI_ISL_418258

EPI_ISL_418259

EPI_ISL_418269

hCoV-19/Italy/TE4953/2020

hCoV-19/Italy/TE4959/2020

hCoV-19/Vietnam/19-

01S/2020

Europe / Italy /

Abruzzo

Europe / Italy /

Abruzzo

Asia / Vietnam / Ho Chi Minh City

2020-03-14

2020-03-14

2020-01-22

Presidio ospedaliero "Santo Spirito"

Presidio ospedaliero "Santo Spirito"

unknown

Istituto

Zooprofilattico

Sperimentale

dell'Abruzzo e Molise

"G. Caporale"

Istituto

Zooprofilattico

Sperimentale

dell'Abruzzo e Molise

"G. Caporale"

Microbiology and Immunology department

Lorusso A, Marcacci M, Di Domenico M, Puglia I, Curini V, Ancora M, Di Pasquale A, Rinaldi A, Mangone I, Cammà C, Savini G.

Lorusso A, Marcacci M, Di Domenico M, Puglia I, Curini V, Ancora M, Di Pasquale A, Rinaldi A, Mangone I, Cammà C, Savini G.

Cao,T.M., Nguyen,H.T., Pham,H.T.T., Vu,N.P.H., Dao,M.H., Huynh,L.T.K., Nguyen,L.T., Nguyen,N.T., Nguyen,T.T.N., Nguyen,A.H., Luong,Q.C., Nguyen,T.V., Tran,K.C., Pham,Q.D., Tran,T., Hoang,C.Q., Nguyen,T.T., Le,H.Q., Phung,T.M., Vo,T.N.A., Nguyen,S.N., Pham,D.T., Phan,L.T. and Nguyen,T.V.

EPI_ISL_418270

EPI_ISL_418340

EPI_ISL_418341

EPI_ISL_418342

EPI_ISL_418343

EPI_ISL_418345

EPI_ISL_418346

hCoV-19/Belgium/JL-

03044/2020

hCoV-

19/Canada/ON_PHL1083/202

0

hCoV-

19/Canada/ON_PHL0142/202

0

hCoV-

19/Canada/ON_PHL0178/202

0

hCoV-

19/Canada/ON_PHL6884/202

0

hCoV-

19/Canada/ON_PHL8751/202

0

hCoV-

19/Canada/ON_PHL0743/202

0

Europe /

Belgium /

Antwerp

North America / Canada / Ontario

North America / Canada / Ontario

North America / Canada / Ontario

North America / Canada / Ontario

North America / Canada / Ontario

North America / Canada / Ontario

2020-03-04

2020-03-09

2020-03-12

2020-03-11

2020-03-10

2020-02-29

2020-03-07

KU Leuven, Clinical and Epidemiological Virology

Public Health

Ontario

Laboratories

Public Health Ontario Laboratories

Public Health Ontario Laboratories

Public Health Ontario Laboratories

Public Health

Ontario

Laboratories

Public Health Ontario Laboratories

KU Leuven, Clinical and Epidemiological Virology

Public Health

Ontario Laboratories

Public Health

Ontario Laboratories

Public Health

Ontario Laboratories

Public Health

Ontario Laboratories

Public Health

Ontario Laboratories

Public Health

Ontario Laboratories

Tony Wawina, Joan Marti-Carreras, Bert Vanmechelen, Piet Maes

Alireza Eshaghi, Samir N Patel, Jonathan B Gubbay, Vanessa G Allen, Christine Frantz, Aimin Li, Sandeep Nagra

Alireza Eshaghi, Samir N Patel, Jonathan B Gubbay, Vanessa G Allen, Christine Frantz, Aimin Li, Sandeep Nagra

Alireza Eshaghi, Samir N Patel, Jonathan B Gubbay, Vanessa G Allen, Christine Frantz, Aimin Li, Sandeep Nagra

Alireza Eshaghi, Samir N Patel, Jonathan B Gubbay, Vanessa G Allen, Christine Frantz, Aimin Li, Sandeep Nagra

Alireza Eshaghi, Samir N Patel, Jonathan B Gubbay, Vanessa G Allen, Christine Frantz, Aimin Li, Sandeep Nagra

Alireza Eshaghi, Samir N Patel, Jonathan B Gubbay, Vanessa G Allen, Christine Frantz, Aimin Li, Sandeep Nagra

|  |  | hCoV- |  | North America / |  |  | Public Health |  |
| --- | --- | --- | --- | --- | --- | --- | --- | --- |
| EPI_ISL_418347 |  | 19/Canada/ON_PHL3741/202 |  | Canada / | 2020-03-11 | | Ontario |  |
|  | 0 | |  | Ontario |  |  | Laboratories |  |
|  |  | hCoV- |  | North America / |  |  | Public Health |  |
| EPI_ISL_418348 |  | 19/Canada/ON_PHL3680/202 |  | Canada / | 2020-03-08 | | Ontario |  |
|  | 0 | |  | Ontario |  |  | Laboratories |  |
|  |  | hCoV- |  | North America / |  |  | Public Health |  |
| EPI_ISL_418349 |  | 19/Canada/ON_PHL3650/202 |  | Canada / | 2020-03-07 | | Ontario |  |
|  | 0 | |  | Ontario |  |  | Laboratories |  |
|  |  | hCoV- |  | North America / |  |  | Public Health |  |
| EPI_ISL_418351 |  | 19/Canada/ON_PHL3575/202 |  | Canada / | 2020-03-12 | | Ontario |  |
|  | 0 | |  | Ontario |  |  | Laboratories |  |
|  |  | hCoV- |  | North America / |  |  | Public Health |  |
| EPI_ISL_418352 |  | 19/Canada/ON_PHL5672/202 |  | Canada / | 2020-03-11 | | Ontario |  |
|  | 0 | |  | Ontario |  |  | Laboratories |  |
|  |  | hCoV- |  | North America / |  |  | Public Health |  |
| EPI_ISL_418353 |  | 19/Canada/ON_PHL0539/202 |  | Canada / | 2020-03-09 | | Ontario |  |
|  | 0 | |  | Ontario |  |  | Laboratories |  |
|  |  | hCoV- |  | North America / |  |  | Public Health |  |
| EPI_ISL_418355 |  | 19/Canada/ON_PHLU8150/20 |  | Canada / | 2020-03-08 | | Ontario |  |
|  | 20 | |  | Ontario |  |  | Laboratories |  |
|  |  | hCoV- |  | North America / |  |  | Public Health |  |
| EPI_ISL_418356 |  | 19/Canada/ON_PHL6980/202 |  | Canada / | 2020-03-12 | | Ontario |  |
|  | 0 | |  | Ontario |  |  | Laboratories |  |
|  |  | hCoV- |  | North America / |  |  | Public Health |  |
| EPI_ISL_418357 |  | 19/Canada/ON_PHLH6415/20 |  | Canada / | 2020-03-12 | | Ontario |  |
|  | 20 | |  | Ontario |  |  | Laboratories |  |
|  |  | hCoV- |  | North America / |  |  | Public Health |  |
| EPI_ISL_418358 |  | 19/Canada/ON_PHL5756/202 |  | Canada / | 2020-03-12 | | Ontario |  |
|  | 0 | |  | Ontario |  |  | Laboratories |  |
|  |  | hCoV- |  | North America / |  |  | Public Health |  |
| EPI_ISL_418359 |  | 19/Canada/ON_PHL5757/202 |  | Canada / | 2020-03-12 | | Ontario |  |
|  |  | 0 |  | Ontario |  |  | Laboratories |  |
| EPI_ISL_418360 |  | hCoV- |  | North America / | 2020-03-11 | | Public Health |  |
|  |  | 19/Canada/ON_PHL3692/202 |  | Canada / |  |  | Ontario |  |
|  | 0 | |  | Ontario |  |  | Laboratories |  |
|  |  | hCoV- |  | North America / |  |  | Public Health |  |
| EPI_ISL_418361 |  | 19/Canada/ON_PHL1095/202 |  | Canada / | 2020-03-12 | | Ontario |  |
|  | 0 | |  | Ontario |  |  | Laboratories |  |
|  |  | hCoV- |  | North America / |  |  | Public Health |  |
| EPI_ISL_418362 |  | 19/Canada/ON_PHL3536/202 |  | Canada / | 2020-03-10 | | Ontario |  |
|  |  | 0 |  | Ontario |  |  | Laboratories |  |

Public Health

Ontario Laboratories

Public Health

Ontario Laboratories

Public Health

Ontario Laboratories

Public Health

Ontario Laboratories

Public Health

Ontario Laboratories

Public Health

Ontario Laboratories

Public Health

Ontario Laboratories

Public Health

Ontario Laboratories

Public Health

Ontario Laboratories

Public Health

Ontario Laboratories

Public Health

Ontario Laboratories

Public Health

Ontario Laboratories

Public Health

Ontario Laboratories

Public Health

Ontario Laboratories

Alireza Eshaghi, Samir N Patel, Jonathan B Gubbay, Vanessa G Allen, Christine Frantz, Aimin Li, Sandeep Nagra

Alireza Eshaghi, Samir N Patel, Jonathan B Gubbay, Vanessa G Allen, Christine Frantz, Aimin Li, Sandeep Nagra

Alireza Eshaghi, Samir N Patel, Jonathan B Gubbay, Vanessa G Allen, Christine Frantz, Aimin Li, Sandeep Nagra

Alireza Eshaghi, Samir N Patel, Jonathan B Gubbay, Vanessa G Allen, Christine Frantz, Aimin Li, Sandeep Nagra

Alireza Eshaghi, Samir N Patel, Jonathan B Gubbay, Vanessa G Allen, Christine Frantz, Aimin Li, Sandeep Nagra

Alireza Eshaghi, Samir N Patel, Jonathan B Gubbay, Vanessa G Allen, Christine Frantz, Aimin Li, Sandeep Nagra

Alireza Eshaghi, Samir N Patel, Jonathan B Gubbay, Vanessa G Allen, Christine Frantz, Aimin Li, Sandeep Nagra

Alireza Eshaghi, Samir N Patel, Jonathan B Gubbay, Vanessa G Allen, Christine Frantz, Aimin Li, Sandeep Nagra

Alireza Eshaghi, Samir N Patel, Jonathan B Gubbay, Vanessa G Allen, Christine Frantz, Aimin Li, Sandeep Nagra

Alireza Eshaghi, Samir N Patel, Jonathan B Gubbay, Vanessa G Allen, Christine Frantz, Aimin Li, Sandeep Nagra

Alireza Eshaghi, Samir N Patel, Jonathan B Gubbay, Vanessa G Allen, Christine Frantz, Aimin Li, Sandeep Nagra

Alireza Eshaghi, Samir N Patel, Jonathan B Gubbay, Vanessa G Allen, Christine Frantz, Aimin Li, Sandeep Nagra

Alireza Eshaghi, Samir N Patel, Jonathan B Gubbay, Vanessa G Allen, Christine Frantz, Aimin Li, Sandeep Nagra

Alireza Eshaghi, Samir N Patel, Jonathan B Gubbay, Vanessa G Allen, Christine Frantz, Aimin Li, Sandeep Nagra

|  |  | hCoV- |  | North America / |  |  | Public Health |  |
| --- | --- | --- | --- | --- | --- | --- | --- | --- |
| EPI_ISL_418363 |  | 19/Canada/ON_PHL3695/202 |  | Canada / | 2020-03-11 | | Ontario |  |
|  | 0 | |  | Ontario |  |  | Laboratories |  |
|  |  | hCoV- |  | North America / |  |  | Public Health |  |
| EPI_ISL_418364 |  | 19/Canada/ON_PHL6922/202 |  | Canada / | 2020-03-13 | | Ontario |  |
|  | 0 | |  | Ontario |  |  | Laboratories |  |
|  |  | hCoV- |  | North America / |  |  | Public Health |  |
| EPI_ISL_418365 |  | 19/Canada/ON_PHL0977/202 |  | Canada / | 2020-03-10 | | Ontario |  |
|  | 0 | |  | Ontario |  |  | Laboratories |  |
|  |  | hCoV- |  | North America / |  |  | Public Health |  |
| EPI_ISL_418366 |  | 19/Canada/ON_PHL3350/202 |  | Canada / | 2020-03-05 | | Ontario |  |
|  | 0 | |  | Ontario |  |  | Laboratories |  |
|  |  | hCoV- |  | North America / |  |  | Public Health |  |
| EPI_ISL_418367 |  | 19/Canada/ON_PHL7513/202 |  | Canada / | 2020-03-12 | | Ontario |  |
|  | 0 | |  | Ontario |  |  | Laboratories |  |
|  |  | hCoV- |  | North America / |  |  | Public Health |  |
| EPI_ISL_418368 |  | 19/Canada/ON_PHL3458/202 |  | Canada / | 2020-03-12 | | Ontario |  |
|  | 0 | |  | Ontario |  |  | Laboratories |  |
|  |  | hCoV- |  | North America / |  |  | Public Health |  |
| EPI_ISL_418369 |  | 19/Canada/ON_PHL8539/202 |  | Canada / | 2020-03-10 | | Ontario |  |
|  | 0 | |  | Ontario |  |  | Laboratories |  |
|  |  | hCoV- |  | North America / |  |  | Public Health |  |
| EPI_ISL_418370 |  | 19/Canada/ON_PHL0654/202 |  | Canada / | 2020-03-11 | | Ontario |  |
|  | 0 | |  | Ontario |  |  | Laboratories |  |
|  |  | hCoV- |  | North America / |  |  | Public Health |  |
| EPI_ISL_418371 |  | 19/Canada/ON_PHL2653/202 |  | Canada / | 2020-03-07 | | Ontario |  |
|  | 0 | |  | Ontario |  |  | Laboratories |  |
|  |  | hCoV- |  | North America / |  |  | Public Health |  |
| EPI_ISL_418372 |  | 19/Canada/ON_PHL8458/202 |  | Canada / | 2020-03-12 | | Ontario |  |
|  | 0 | |  | Ontario |  |  | Laboratories |  |
|  |  | hCoV- |  | North America / |  |  | Public Health |  |
| EPI_ISL_418373 |  | 19/Canada/ON_PHL7590/202 |  | Canada / | 2020-03-14 | | Ontario |  |
|  |  | 0 |  | Ontario |  |  | Laboratories |  |
| EPI_ISL_418374 |  | hCoV- |  | North America / | 2020-03-11 | | Public Health |  |
|  |  | 19/Canada/ON_PHL4232/202 |  | Canada / |  |  | Ontario |  |
|  | 0 | |  | Ontario |  |  | Laboratories |  |
|  |  | hCoV- |  | North America / |  |  | Public Health |  |
| EPI_ISL_418375 |  | 19/Canada/ON_PHL0141/202 |  | Canada / | 2020-03-12 | | Ontario |  |
|  | 0 | |  | Ontario |  |  | Laboratories |  |
|  |  | hCoV- |  | North America / |  |  | Public Health |  |
| EPI_ISL_418376 |  | 19/Canada/ON_PHL3459/202 |  | Canada / | 2020-03-12 | | Ontario |  |
|  |  | 0 |  | Ontario |  |  | Laboratories |  |

Public Health

Ontario Laboratories

Public Health

Ontario Laboratories

Public Health

Ontario Laboratories

Public Health

Ontario Laboratories

Public Health

Ontario Laboratories

Public Health

Ontario Laboratories

Public Health

Ontario Laboratories

Public Health

Ontario Laboratories

Public Health

Ontario Laboratories

Public Health

Ontario Laboratories

Public Health

Ontario Laboratories

Public Health

Ontario Laboratories

Public Health

Ontario Laboratories

Public Health

Ontario Laboratories

Alireza Eshaghi, Samir N Patel, Jonathan B Gubbay, Vanessa G Allen, Christine Frantz, Aimin Li, Sandeep Nagra

Alireza Eshaghi, Samir N Patel, Jonathan B Gubbay, Vanessa G Allen, Christine Frantz, Aimin Li, Sandeep Nagra

Alireza Eshaghi, Samir N Patel, Jonathan B Gubbay, Vanessa G Allen, Christine Frantz, Aimin Li, Sandeep Nagra

Alireza Eshaghi, Samir N Patel, Jonathan B Gubbay, Vanessa G Allen, Christine Frantz, Aimin Li, Sandeep Nagra

Alireza Eshaghi, Samir N Patel, Jonathan B Gubbay, Vanessa G Allen, Christine Frantz, Aimin Li, Sandeep Nagra

Alireza Eshaghi, Samir N Patel, Jonathan B Gubbay, Vanessa G Allen, Christine Frantz, Aimin Li, Sandeep Nagra

Alireza Eshaghi, Samir N Patel, Jonathan B Gubbay, Vanessa G Allen, Christine Frantz, Aimin Li, Sandeep Nagra

Alireza Eshaghi, Samir N Patel, Jonathan B Gubbay, Vanessa G Allen, Christine Frantz, Aimin Li, Sandeep Nagra

Alireza Eshaghi, Samir N Patel, Jonathan B Gubbay, Vanessa G Allen, Christine Frantz, Aimin Li, Sandeep Nagra

Alireza Eshaghi, Samir N Patel, Jonathan B Gubbay, Vanessa G Allen, Christine Frantz, Aimin Li, Sandeep Nagra

Alireza Eshaghi, Samir N Patel, Jonathan B Gubbay, Vanessa G Allen, Christine Frantz, Aimin Li, Sandeep Nagra

Alireza Eshaghi, Samir N Patel, Jonathan B Gubbay, Vanessa G Allen, Christine Frantz, Aimin Li, Sandeep Nagra

Alireza Eshaghi, Samir N Patel, Jonathan B Gubbay, Vanessa G Allen, Christine Frantz, Aimin Li, Sandeep Nagra

Alireza Eshaghi, Samir N Patel, Jonathan B Gubbay, Vanessa G Allen, Christine Frantz, Aimin Li, Sandeep Nagra

EPI_ISL_418378

EPI_ISL_419541

EPI_ISL_419553

EPI_ISL_419554

EPI_ISL_419555

hCoV-

19/Canada/ON_PHL0976/202

0

hCoV-19/Germany/NRW-

24/2020

hCoV-19/USA/RI_0520/2020

hCoV-19/USA/CA_2602/2020

hCoV-19/USA/WA_5030/2020

North America / Canada / Ontario

Europe /

Germany /

Duesseldorf

North America / USA / Rhode Island

North America / USA / California

North America /

USA /

Washington

2020-03-13

2020-03-14

2020-02-28

2020-02-26

2020-02-27

Public Health Ontario Laboratories

Center of Medical Microbiology, Virology, and Hospital Hygiene, University of Duesseldorf

RI State Health Laboratories

California Department of Public Health

WA State Department of Health

Public Health

Ontario Laboratories

Center of Medical Microbiology, Virology, and Hospital Hygiene, University of Duesseldorf

Pathogen Discovery, Respiratory Viruses Branch, Division of Viral Diseases, Centers for Disease Control and Prevention

Pathogen Discovery, Respiratory Viruses Branch, Division of Viral Diseases, Centers for Disease Control and Prevention

Pathogen Discovery, Respiratory Viruses Branch, Division of Viral Diseases, Centers for Disease Control and Prevention

Alireza Eshaghi, Samir N Patel, Jonathan B Gubbay, Vanessa G Allen, Christine Frantz, Aimin Li, Sandeep Nagra

Ortwin Adams, Marcel Andree, Alexander Dilthey, Torsten Feldt, Sandra Hauka, Torsten Houwaart, Björn-Erik Jensen, Detlef Kindgen-Milles, Malte Kohns Vasconcelos, Klaus Pfeffer, Tina Senff, Daniel Strelow, Jörg Timm, Andreas Walker, Tobias Wienemann

Ying Tao, Jing Zhang, Krista Queen, Anna Uehara, Clinton R. Paden, Yan Li, Haibin Wang, Jasmine Padilla, Justin Lee, Suxiang Tong

Ying Tao, Jing Zhang, Krista Queen, Anna Uehara, Clinton R. Paden, Yan Li, Haibin Wang, Jasmine Padilla, Justin Lee, Suxiang Tong

Ying Tao, Jing Zhang, Krista Queen, Anna Uehara, Clinton R. Paden, Yan Li, Haibin Wang, Jasmine Padilla, Justin Lee, Suxiang Tong

EPI_ISL_419556

EPI_ISL_419557

EPI_ISL_419558

EPI_ISL_419559

hCoV-19/USA/GA_2741/2020

hCoV-19/USA/GA_2742/2020

hCoV-19/USA/OR_2656/2020

hCoV-19/USA/FL_5125/2020

North America / USA / Georgia

North America / USA / Georgia

North America / USA / Oregon

North America / USA / Florida

2020-02-29

2020-02-29

2020-02-27

2020-02-28

GA Department of Public Health Laboratory

GA Department of Public Health Laboratory

OR State PHL-Virology/Immunol ogy Section

FL Bureau of Public Health Laboratories-Tampa

Pathogen Discovery, Respiratory Viruses Branch, Division of Viral Diseases, Centers for Disease Control and Prevention

Pathogen Discovery, Respiratory Viruses Branch, Division of Viral Diseases, Centers for Disease Control and Prevention

Pathogen Discovery, Respiratory Viruses Branch, Division of Viral Diseases, Centers for Disease Control and Prevention

Pathogen Discovery, Respiratory Viruses Branch, Division of Viral Diseases, Centers for Disease Control and Prevention

Ying Tao, Jing Zhang, Krista Queen, Anna Uehara, Clinton R. Paden, Yan Li, Haibin Wang, Jasmine Padilla, Justin Lee, Suxiang Tong

Ying Tao, Jing Zhang, Krista Queen, Anna Uehara, Clinton R. Paden, Yan Li, Haibin Wang, Jasmine Padilla, Justin Lee, Suxiang Tong

Ying Tao, Jing Zhang, Krista Queen, Anna Uehara, Clinton R. Paden, Yan Li, Haibin Wang, Jasmine Padilla, Justin Lee, Suxiang Tong

Anna Uehara, Ying Tao, Jing Zhang, Krista Queen, Clinton R. Paden, Yan Li, Haibin Wang, Jasmine Padilla, Justin Lee, Suxiang Tong

EPI_ISL_419560

EPI_ISL_419561

EPI_ISL_419562

EPI_ISL_419563

EPI_ISL_419566

EPI_ISL_419568

hCoV-19/USA/FL_5091/2020

hCoV-19/USA/TX_2020/2020

hCoV-

19/Luxembourg/LNS0000001/

2020

hCoV-

19/Luxembourg/LNS0156959/

2020

hCoV-

19/Luxembourg/LNS0641910/

2020

hCoV-

19/Luxembourg/LNS0756270/

2020

North America / USA / Florida

North America / USA / Texas

Europe /

Luxembourg

Europe /

Luxembourg

Europe /

Luxembourg

Europe /

Luxembourg

2020-02-28

2020-02-29

2020-02-29

2020-03-12

2020-03-05

2020-03-14

|  |  | Pathogen Discovery, |  |  |
| --- | --- | --- | --- | --- |
| FL Bureau of |  | Respiratory Viruses | Anna Uehara, Ying Tao, Jing Zhang, Krista Queen, Clinton R. |  |
|  |  | Branch, Division of |  |  |
| Public Health |  |  |  |  |
|  |  | Viral Diseases, | Paden, Yan Li, Haibin Wang, Jasmine Padilla, Justin Lee, |  |
| Laboratories- |  |  |  |  |
|  |  | Centers for Disease | Suxiang Tong |  |
| Tampa |  |  |  |  |
|  |  | Control and |  |  |
|  |  |  |  |  |
|  |  | Prevention |  |  |
|  |  |  |  |  |
| Texas |  | Pathogen Discovery, |  |  |
|  |  | Respiratory Viruses |  |  |
| Department of |  | Branch, Division of | Anna Uehara, Ying Tao, Jing Zhang, Krista Queen, Clinton R. |  |
| State Health |  | Viral Diseases, | Paden, Yan Li, Haibin Wang, Jasmine Padilla, Justin Lee, |  |
| Services Lab |  | Centers for Disease | Suxiang Tong |  |
| Services |  | Control and |  |  |
|  |  | Prevention |  |  |
|  |  |  |  |  |
| Laboratoire |  | Laboratoire National |  |  |
| National de |  | de Santé, | Anke Wienecke-Baldacchino, Ardashel Latsuzbaia, Jessica |  |
| Santé, |  | Microbiology, | Tapp, Catherine Ragimbeau, Guillaume Fournier, Tamir |  |
| Microbiology, |  | Epidemiology and | Abdelrahman, Trung Nguyen Nguyen, Joel Mossong |  |
| Virology |  | Microbial Genomics |  |  |
|  |  |  |  |  |
| Laboratoire |  | Laboratoire National |  |  |
| National de |  | de Santé, | Anke Wienecke-Baldacchino, Ardashel Latsuzbaia, Jessica |  |
| Santé, |  | Microbiology, | Tapp, Catherine Ragimbeau, Guillaume Fournier, Tamir |  |
| Microbiology, |  | Epidemiology and | Abdelrahman, Trung Nguyen Nguyen, Joel Mossong |  |
| Virology |  | Microbial Genomics |  |  |
|  |  |  |  |  |
| Laboratoire |  | Laboratoire National |  |  |
| National de |  | de Santé, | Anke Wienecke-Baldacchino, Ardashel Latsuzbaia, Jessica |  |
| Santé, |  | Microbiology, | Tapp, Catherine Ragimbeau, Guillaume Fournier, Tamir |  |
| Microbiology, |  | Epidemiology and | Abdelrahman, Trung Nguyen Nguyen, Joel Mossong |  |
| Virology |  | Microbial Genomics |  |  |
|  |  |  |  |  |
| Laboratoire |  | Laboratoire National |  |  |
| National de |  | de Santé, | Anke Wienecke-Baldacchino, Ardashel Latsuzbaia, Jessica |  |
| Santé, |  | Microbiology, | Tapp, Catherine Ragimbeau, Guillaume Fournier, Tamir |  |
| Microbiology, |  | Epidemiology and | Abdelrahman, Trung Nguyen Nguyen, Joel Mossong |  |
| Virology |  | Microbial Genomics |  |  |

EPI_ISL_419569

EPI_ISL_419570

EPI_ISL_419573

EPI_ISL_419578

EPI_ISL_419579

EPI_ISL_419580

EPI_ISL_419582

EPI_ISL_419584

hCoV-

19/Luxembourg/LNS0945359/

2020

hCoV-

19/Luxembourg/LNS1234709/

2020

hCoV-

19/Luxembourg/LNS1874423/

2020

hCoV-

19/Luxembourg/LNS2614631/

2020

hCoV-

19/Luxembourg/LNS2886370/

2020

hCoV-

19/Luxembourg/LNS2907333/

2020

hCoV-

19/Luxembourg/LNS3156434/

2020

hCoV-

19/Luxembourg/LNS3711853/

2020

Europe /

Luxembourg

Europe /

Luxembourg

Europe /

Luxembourg

Europe /

Luxembourg

Europe /

Luxembourg

Europe /

Luxembourg

Europe /

Luxembourg

Europe /

Luxembourg

2020-03-07

2020-03-12

2020-03-11

2020-03-08

2020-03-14

2020-03-12

2020-03-13

2020-03-13

| Laboratoire |  | Laboratoire National |
| --- | --- | --- |
| National de |  | de Santé, |
| Santé, |  | Microbiology, |
| Microbiology, |  | Epidemiology and |
| Virology |  | Microbial Genomics |
|  |  |  |
| Laboratoire |  | Laboratoire National |
| National de |  | de Santé, |
| Santé, |  | Microbiology, |
| Microbiology, |  | Epidemiology and |
| Virology |  | Microbial Genomics |
|  |  |  |
| Laboratoire |  | Laboratoire National |
| National de |  | de Santé, |
| Santé, |  | Microbiology, |
| Microbiology, |  | Epidemiology and |
| Virology |  | Microbial Genomics |
| Laboratoire |  | Laboratoire National |
| National de |  | de Santé, |
| Santé, |  | Microbiology, |
| Microbiology, |  | Epidemiology and |
| Virology |  | Microbial Genomics |
|  |  |  |
| Laboratoire |  | Laboratoire National |
| National de |  | de Santé, |
| Santé, |  | Microbiology, |
| Microbiology, |  | Epidemiology and |
| Virology |  | Microbial Genomics |
| Laboratoire |  | Laboratoire National |
| National de |  | de Santé, |
| Santé, |  | Microbiology, |
| Microbiology, |  | Epidemiology and |
| Virology |  | Microbial Genomics |
|  |  |  |
| Laboratoire |  | Laboratoire National |
| National de |  | de Santé, |
| Santé, |  | Microbiology, |
| Microbiology, |  | Epidemiology and |
| Virology |  | Microbial Genomics |
|  |  |  |
| Laboratoire |  | Laboratoire National |
| National de |  | de Santé, |
| Santé, |  | Microbiology, |
| Microbiology, |  | Epidemiology and |
| Virology |  | Microbial Genomics |
|  |  |  |

Anke Wienecke-Baldacchino, Ardashel Latsuzbaia, Jessica Tapp, Catherine Ragimbeau, Guillaume Fournier, Tamir Abdelrahman, Trung Nguyen Nguyen, Joel Mossong

Anke Wienecke-Baldacchino, Ardashel Latsuzbaia, Jessica Tapp, Catherine Ragimbeau, Guillaume Fournier, Tamir Abdelrahman, Trung Nguyen Nguyen, Joel Mossong

Anke Wienecke-Baldacchino, Ardashel Latsuzbaia, Jessica Tapp, Catherine Ragimbeau, Guillaume Fournier, Tamir Abdelrahman, Trung Nguyen Nguyen, Joel Mossong

Anke Wienecke-Baldacchino, Ardashel Latsuzbaia, Jessica Tapp, Catherine Ragimbeau, Guillaume Fournier, Tamir Abdelrahman, Trung Nguyen Nguyen, Joel Mossong

Anke Wienecke-Baldacchino, Ardashel Latsuzbaia, Jessica Tapp, Catherine Ragimbeau, Guillaume Fournier, Tamir Abdelrahman, Trung Nguyen Nguyen, Joel Mossong

Anke Wienecke-Baldacchino, Ardashel Latsuzbaia, Jessica Tapp, Catherine Ragimbeau, Guillaume Fournier, Tamir Abdelrahman, Trung Nguyen Nguyen, Joel Mossong

Anke Wienecke-Baldacchino, Ardashel Latsuzbaia, Jessica Tapp, Catherine Ragimbeau, Guillaume Fournier, Tamir Abdelrahman, Trung Nguyen Nguyen, Joel Mossong

Anke Wienecke-Baldacchino, Ardashel Latsuzbaia, Jessica Tapp, Catherine Ragimbeau, Guillaume Fournier, Tamir Abdelrahman, Trung Nguyen Nguyen, Joel Mossong

EPI_ISL_419585

EPI_ISL_419586

EPI_ISL_419587

EPI_ISL_419590

EPI_ISL_419591

EPI_ISL_419595

EPI_ISL_419596

EPI_ISL_419598

hCoV-

19/Luxembourg/LNS3879580/

2020

hCoV-

19/Luxembourg/LNS4134806/

2020

hCoV-

19/Luxembourg/LNS4691488/

2020

hCoV-

19/Luxembourg/LNS5032431/

2020

hCoV-

19/Luxembourg/LNS5228153/

2020

hCoV-

19/Luxembourg/LNS7753431/

2020

hCoV-

19/Luxembourg/LNS7866283/

2020

hCoV-

19/Luxembourg/LNS8188502/

2020

Europe /

Luxembourg

Europe /

Luxembourg

Europe /

Luxembourg

Europe /

Luxembourg

Europe /

Luxembourg

Europe /

Luxembourg

Europe /

Luxembourg

Europe /

Luxembourg

2020-03-14

2020-03-14

2020-03-11

2020-03-11

2020-03-14

2020-03-12

2020-03-12

2020-03-14

| Laboratoire |  | Laboratoire National |
| --- | --- | --- |
| National de |  | de Santé, |
| Santé, |  | Microbiology, |
| Microbiology, |  | Epidemiology and |
| Virology |  | Microbial Genomics |
|  |  |  |
| Laboratoire |  | Laboratoire National |
| National de |  | de Santé, |
| Santé, |  | Microbiology, |
| Microbiology, |  | Epidemiology and |
| Virology |  | Microbial Genomics |
|  |  |  |
| Laboratoire |  | Laboratoire National |
| National de |  | de Santé, |
| Santé, |  | Microbiology, |
| Microbiology, |  | Epidemiology and |
| Virology |  | Microbial Genomics |
| Laboratoire |  | Laboratoire National |
| National de |  | de Santé, |
| Santé, |  | Microbiology, |
| Microbiology, |  | Epidemiology and |
| Virology |  | Microbial Genomics |
|  |  |  |
| Laboratoire |  | Laboratoire National |
| National de |  | de Santé, |
| Santé, |  | Microbiology, |
| Microbiology, |  | Epidemiology and |
| Virology |  | Microbial Genomics |
| Laboratoire |  | Laboratoire National |
| National de |  | de Santé, |
| Santé, |  | Microbiology, |
| Microbiology, |  | Epidemiology and |
| Virology |  | Microbial Genomics |
|  |  |  |
| Laboratoire |  | Laboratoire National |
| National de |  | de Santé, |
| Santé, |  | Microbiology, |
| Microbiology, |  | Epidemiology and |
| Virology |  | Microbial Genomics |
|  |  |  |
| Laboratoire |  | Laboratoire National |
| National de |  | de Santé, |
| Santé, |  | Microbiology, |
| Microbiology, |  | Epidemiology and |
| Virology |  | Microbial Genomics |
|  |  |  |

Anke Wienecke-Baldacchino, Ardashel Latsuzbaia, Jessica Tapp, Catherine Ragimbeau, Guillaume Fournier, Tamir Abdelrahman, Trung Nguyen Nguyen, Joel Mossong

Anke Wienecke-Baldacchino, Ardashel Latsuzbaia, Jessica Tapp, Catherine Ragimbeau, Guillaume Fournier, Tamir Abdelrahman, Trung Nguyen Nguyen, Joel Mossong

Anke Wienecke-Baldacchino, Ardashel Latsuzbaia, Jessica Tapp, Catherine Ragimbeau, Guillaume Fournier, Tamir Abdelrahman, Trung Nguyen Nguyen, Joel Mossong

Anke Wienecke-Baldacchino, Ardashel Latsuzbaia, Jessica Tapp, Catherine Ragimbeau, Guillaume Fournier, Tamir Abdelrahman, Trung Nguyen Nguyen, Joel Mossong

Anke Wienecke-Baldacchino, Ardashel Latsuzbaia, Jessica Tapp, Catherine Ragimbeau, Guillaume Fournier, Tamir Abdelrahman, Trung Nguyen Nguyen, Joel Mossong

Anke Wienecke-Baldacchino, Ardashel Latsuzbaia, Jessica Tapp, Catherine Ragimbeau, Guillaume Fournier, Tamir Abdelrahman, Trung Nguyen Nguyen, Joel Mossong

Anke Wienecke-Baldacchino, Ardashel Latsuzbaia, Jessica Tapp, Catherine Ragimbeau, Guillaume Fournier, Tamir Abdelrahman, Trung Nguyen Nguyen, Joel Mossong

Anke Wienecke-Baldacchino, Ardashel Latsuzbaia, Jessica Tapp, Catherine Ragimbeau, Guillaume Fournier, Tamir Abdelrahman, Trung Nguyen Nguyen, Joel Mossong

EPI_ISL_419600

EPI_ISL_419602

EPI_ISL_419606

EPI_ISL_419607

EPI_ISL_419676

EPI_ISL_419677

hCoV-

19/Luxembourg/LNS9080444/

2020

hCoV-

19/Luxembourg/LNS9324837/

2020

hCoV-

19/Luxembourg/LNS9862689/

2020

hCoV-

19/Luxembourg/LNS9982497/

2020

hCoV-

19/Spain/Valencia12/2020

hCoV-

19/Spain/Valencia13/2020

Europe /

Luxembourg

Europe /

Luxembourg

Europe /

Luxembourg

Europe /

Luxembourg

Europe / Spain

- Comunitat Valenciana / Valencia

Europe / Spain

- Comunitat Valenciana / Valencia

2020-03-13

2020-03-12

2020-03-11

2020-03-14

2020-03-09

2020-03-09

Laboratoire

National de

Santé,

Microbiology,

Virology

Laboratoire

National de

Santé,

Microbiology,

Virology

Laboratoire

National de

Santé,

Microbiology,

Virology

Laboratoire

National de

Santé,

Microbiology,

Virology

Servicio de Microbiología. Consorcio

Hospital General Universitario de Valencia

Servicio de Microbiología. Consorcio

Hospital General Universitario de Valencia

Laboratoire National

de Santé,

Microbiology,

Epidemiology and

Microbial Genomics

Laboratoire National

de Santé,

Microbiology,

Epidemiology and

Microbial Genomics

Laboratoire National

de Santé,

Microbiology,

Epidemiology and

Microbial Genomics

Laboratoire National

de Santé,

Microbiology,

Epidemiology and

Microbial Genomics

Sequencing and Bioinformatics Service and Molecular Epidemiology Research Group. FISABIO-Public Health

Sequencing and Bioinformatics Service and Molecular Epidemiology Research Group. FISABIO-Public Health

Anke Wienecke-Baldacchino, Ardashel Latsuzbaia, Jessica Tapp, Catherine Ragimbeau, Guillaume Fournier, Tamir Abdelrahman, Trung Nguyen Nguyen, Joel Mossong

Anke Wienecke-Baldacchino, Ardashel Latsuzbaia, Jessica Tapp, Catherine Ragimbeau, Guillaume Fournier, Tamir Abdelrahman, Trung Nguyen Nguyen, Joel Mossong

Anke Wienecke-Baldacchino, Ardashel Latsuzbaia, Jessica Tapp, Catherine Ragimbeau, Guillaume Fournier, Tamir Abdelrahman, Trung Nguyen Nguyen, Joel Mossong

Anke Wienecke-Baldacchino, Ardashel Latsuzbaia, Jessica Tapp, Catherine Ragimbeau, Guillaume Fournier, Tamir Abdelrahman, Trung Nguyen Nguyen, Joel Mossong

Maria Dolores Ocete, Giuseppe D'Auria, Griselda De Marco, Neris Garcia-Gonzalez, Maria Alma Bracho, Concepcion Gimeno, Fernando Gonzalez-Candelas

Giuseppe D'Auria, Griselda De Marco, Neris Garcia-Gonzalez, Maria Alma Bracho, Maria Dolores Ocete, Concepcion Gimeno, Fernando Gonzalez-Candelas

EPI_ISL_419678

EPI_ISL_419679

EPI_ISL_419680

EPI_ISL_419681

EPI_ISL_419682

hCoV-

19/Spain/Valencia14/2020

hCoV-

19/Spain/Valencia15/2020

hCoV-

19/Spain/Valencia16/2020

hCoV-

19/Spain/Valencia17/2020

hCoV-

19/Spain/Valencia18/2020

Europe / Spain

- Comunitat Valenciana / Valencia

Europe / Spain

- Comunitat Valenciana / Valencia

Europe / Spain

- Comunitat Valenciana / Valencia

Europe / Spain

- Comunitat Valenciana / Valencia

Europe / Spain

- Comunitat Valenciana / Valencia

2020-03-09

2020-03-02

2020-03-10

2020-03-10

2020-03-10

Servicio de Microbiología. Consorcio

Hospital General Universitario de Valencia

Servicio de Microbiología. Consorcio

Hospital General Universitario de Valencia

Servicio de Microbiología. Consorcio

Hospital General Universitario de Valencia

Servicio de Microbiología. Consorcio

Hospital General Universitario de Valencia

Servicio de Microbiología. Consorcio

Hospital General Universitario de Valencia

Sequencing and Bioinformatics Service and Molecular Epidemiology Research Group. FISABIO-Public Health

Sequencing and Bioinformatics Service and Molecular Epidemiology Research Group. FISABIO-Public Health

Sequencing and Bioinformatics Service and Molecular Epidemiology Research Group. FISABIO-Public Health

Sequencing and Bioinformatics Service and Molecular Epidemiology Research Group. FISABIO-Public Health

Sequencing and Bioinformatics Service and Molecular Epidemiology Research Group. FISABIO-Public Health

Griselda De Marco, Neris Garcia-Gonzalez, Maria Alma Bracho, Maria Dolores Ocete, Giuseppe D'Auria, Concepcion Gimeno, Fernando Gonzalez-Candelas

Neris Garcia-Gonzalez, Maria Alma Bracho, Maria Dolores Ocete, Giuseppe D'Auria, Griselda De Marco, Concepcion Gimeno, Fernando Gonzalez-Candelas

Maria Alma Bracho, Maria Dolores Ocete, Giuseppe D'Auria, Griselda De Marco, Neris Garcia-Gonzalez, Concepcion Gimeno, Fernando Gonzalez-Candelas

Maria Dolores Ocete, Giuseppe D'Auria, Griselda De Marco, Neris Garcia-Gonzalez, Maria Alma Bracho, Concepcion Gimeno, Fernando Gonzalez-Candelas

Giuseppe D'Auria, Griselda De Marco, Neris Garcia-Gonzalez, Maria Alma Bracho, Maria Dolores Ocete, Concepcion Gimeno, Fernando Gonzalez-Candelas

|  |  |  |  |  |  |  |  |  |  | Sequencing and |  |  |
| --- | --- | --- | --- | --- | --- | --- | --- | --- | --- | --- | --- | --- |
|  |  |  |  | Europe / Spain |  |  | Servicio de |  |  | Bioinformatics |  |  |
|  |  |  |  |  |  |  | Microbiología. | |  | Service and | Griselda De Marco, Neris Garcia-Gonzalez, Maria Alma |  |
|  |  | hCoV- |  | / Comunitat |  |  | Consorcio |  |  | Molecular |  |  |
| EPI_ISL_419683 |  |  |  |  | 2020-03-08 | |  |  |  |  | Bracho, Maria Dolores Ocete, Giuseppe D'Auria, Concepcion |  |
|  |  | 19/Spain/Valencia19/2020 |  | Valenciana / |  |  | Hospital General | |  | Epidemiology |  |  |
|  |  |  |  |  |  |  |  |  |  |  | Gimeno, Fernando Gonzalez-Candelas |  |
|  |  |  |  | Valencia |  |  | Universitario de | |  | Research Group. |  |  |
|  |  |  |  |  |  |  |  |  |  |  |  |  |
|  |  |  |  |  |  |  | Valencia |  |  | FISABIO-Public |  |  |
|  |  |  |  |  |  |  |  |  |  | Health |  |  |
|  |  |  |  |  |  |  |  |  |  |  |  |  |
|  |  |  |  |  |  |  | Victorian |  |  | Victorian Infectious |  |  |
|  |  |  |  |  |  |  |  |  |  | Diseases Reference |  |  |
|  |  |  |  | Oceania / |  |  | Infectious |  |  | Laboratory and |  |  |
|  |  |  |  |  |  |  | Diseases |  |  | Microbiological | Caly L., Seemann T., Sait, M., Schultz M., Druce J., Sherry, |  |
| EPI_ISL_419826 |  | hCoV-19/Australia/VIC51/2020 |  | Australia / | 2020-03-12 | |  |  |  |  |  |  |
|  |  |  |  |  |  |  | Reference |  |  | Diagnostic Unit | N. |  |
|  |  |  |  | Victoria |  |  |  |  |  |  |  |  |
|  |  |  |  |  |  |  | Laboratory |  |  | Public Health |  |  |
|  |  |  |  |  |  |  |  |  |  |  |  |  |
|  |  |  |  |  |  |  | (VIDRL) |  |  | Laboratory, Doherty |  |  |
|  |  |  |  |  |  |  |  |  |  | Institute |  |  |
|  |  |  |  |  |  |  |  |  |  |  |  |  |
|  |  |  |  | Oceania / |  |  | Department | of |  | Department of |  |  |
| EPI_ISL_420532 |  | hCoV-19/Australia/WA02/2020 |  | Australia / | 2020-03-13 | | Microbiology, |  |  | Microbiology, | Chisha Sikazwe, Jurissa Lang, Avram Levy, David Speers |  |
|  |  |  |  | Western |  |  | PathWest QEII | |  | PathWest QEII | and David Smith |  |
|  |  |  |  |  |  |  |  |  |  |  |  |  |
|  |  |  |  | Australia / Perth |  |  | Medical Centre | |  | Medical Centre |  |  |
|  |  |  |  |  |  |  |  |  |  |  |  |  |
|  |  |  |  | Oceania / |  |  | Department | of |  | Department of |  |  |
| EPI_ISL_420533 |  | hCoV-19/Australia/WA03/2020 |  | Australia / | 2020-03-14 | | Microbiology, |  |  | Microbiology, | Chisha Sikazwe, Jurissa Lang, Avram Levy, David Speers |  |
|  |  |  |  | Western |  |  | PathWest QEII | |  | PathWest QEII | and David Smith |  |
|  |  |  |  |  |  |  |  |  |  |  |  |  |
|  |  |  |  | Australia / Perth |  |  | Medical Centre | |  | Medical Centre |  |  |
|  |  |  |  |  |  |  |  |  |  |  |  |  |
|  |  |  |  | Oceania / |  |  | Department | of |  | Department of |  |  |
| EPI_ISL_420534 |  | hCoV-19/Australia/WA06/2020 |  | Australia / | 2020-03-04 | | Microbiology, |  |  | Microbiology, | Chisha Sikazwe, Jurissa Lang, Avram Levy, David Speers |  |
|  |  |  |  | Western |  |  | PathWest QEII | |  | PathWest QEII | and David Smith |  |
|  |  |  |  |  |  |  |  |  |  |  |  |  |
|  |  |  |  | Australia / Perth |  |  | Medical Centre | |  | Medical Centre |  |  |
|  |  |  |  |  |  |  |  |  |  |  |  |  |
|  |  |  |  | Oceania / |  |  | Department | of |  | Department of |  |  |
| EPI_ISL_420537 |  | hCoV-19/Australia/WA07/2020 |  | Australia / | 2020-03-13 | | Microbiology, |  |  | Microbiology, | Chisha Sikazwe, Jurissa Lang, Avram Levy, David Speers |  |
|  |  |  |  | Western |  |  | PathWest QEII | |  | PathWest QEII | and David Smith |  |
|  |  |  |  |  |  |  |  |  |  |  |  |  |
|  |  |  |  | Australia / Perth |  |  | Medical Centre | |  | Medical Centre |  |  |
|  |  |  |  |  |  |  |  |  |  |  |  |  |

EPI_ISL_420538

EPI_ISL_420539

EPI_ISL_420800

EPI_ISL_420801

EPI_ISL_421225

EPI_ISL_421226

hCoV-19/Australia/WA09/2020

hCoV-19/Australia/WA10/2020

hCoV-19/Korea/BA-

ACH_2718/2020

hCoV-19/Korea/BA-

ACH_2719/2020

hCoV-

19/Hangzhou/HZCDC0135/20

20

hCoV-

19/Hangzhou/HZCDC0119/20

20

Oceania / Australia / Western Australia / Perth

Oceania / Australia / Western Australia / Perth

Asia / Korea

Asia / Korea

Asia / China / Hangzhou

Asia / China / Hangzhou

2020-03-14

2020-03-14

2020-02-29

2020-02-29

2020-01-22

2020-01-22

Department of Microbiology, PathWest QEII Medical Centre

Department of Microbiology, PathWest QEII Medical Centre

Brian D. Allgood

Army

Community

Hospital

Brian D. Allgood

Army

Community

Hospital

Hangzhou Center for Diseases Control and Prevention

Hangzhou

Center for

Diseases

Control and

Prevention

Department of Microbiology, PathWest QEII Medical Centre

Department of Microbiology, PathWest QEII Medical Centre

Pathogen Discovery, Respiratory Viruses Branch, Division of Viral Diseases, Centers for Disease Control and Prevention

Pathogen Discovery, Respiratory Viruses Branch, Division of Viral Diseases, Centers for Disease Control and Prevention

Hangzhou Center for Diseases Control and Prevention

Hangzhou Center for Diseases Control and Prevention

Chisha Sikazwe, Jurissa Lang, Avram Levy, David Speers and David Smith

Chisha Sikazwe, Jurissa Lang, Avram Levy, David Speers and David Smith

Krista Queen, Yan Li, Ying Tao, Jing Zhang, Anne Uehara, Clinton R. Paden, Haibin Wang, Rachel Marine, Mary S.

Keckler, Alison S. Laufer Halpin, Jasmine Padilla, Justin Lee, Christopher A. Elkins, Suxiang Tong

Krista Queen, Yan Li, Ying Tao, Jing Zhang, Anne Uehara, Clinton R. Paden, Haibin Wang, Rachel Marine, Mary S.

Keckler, Alison S. Laufer Halpin, Jasmine Padilla, Justin Lee, Christopher A. Elkins, Suxiang Tong

Jun Li, Haoqiu Wang, Lingfeng Mao, Hua Yu, Xinfen Yu, Zhou Sun, Xin Qian, Shuchang Chen, Junfang Chen, Xuchu Wang

Jun Li, Haoqiu Wang, Lingfeng Mao, Hua Yu, Xinfen Yu, Zhou Sun, Xin Qian, Shuchang Chen, Junfang Chen, Xuchu Wang

EPI_ISL_421227

EPI_ISL_421228

EPI_ISL_421229

EPI_ISL_421230

EPI_ISL_421231

EPI_ISL_421232

EPI_ISL_421233

EPI_ISL_421234

hCoV-

19/Hangzhou/HZCDC0091L/2

020

hCoV-

19/Hangzhou/HZCDC0091/20

20

hCoV-

19/Hangzhou/HZCDC0090L/2

020

hCoV-

19/Hangzhou/HZCDC0090/20

20

hCoV-

19/Hangzhou/HZCDC0049L/2

020

hCoV-

19/Hangzhou/HZCDC0048L/2

020

hCoV-

19/Hangzhou/HZCDC0048/20

20

hCoV-

19/Hangzhou/HZCDC0025/20

20

Asia / China / Hangzhou

Asia / China / Hangzhou

Asia / China / Hangzhou

Asia / China / Hangzhou

Asia / China / Hangzhou

Asia / China / Hangzhou

Asia / China / Hangzhou

Asia / China / Hangzhou

2020-01-21

2020-01-21

2020-01-21

2020-01-21

2020-01-21

2020-01-21

2020-01-21

2020-01-21

Hangzhou Center for Diseases Control and Prevention

Hangzhou Center for Diseases Control and Prevention

Hangzhou

Center for Diseases Control and Prevention

Hangzhou Center for Diseases Control and Prevention

Hangzhou

Center for Diseases Control and Prevention

Hangzhou Center for Diseases Control and Prevention

Hangzhou Center for Diseases Control and Prevention

Hangzhou Center for Diseases Control and Prevention

Hangzhou Center for Diseases Control and Prevention

Hangzhou Center for Diseases Control and Prevention

Hangzhou Center for Diseases Control and Prevention

Hangzhou Center for Diseases Control and Prevention

Hangzhou Center for Diseases Control and Prevention

Hangzhou Center for Diseases Control and Prevention

Hangzhou Center for Diseases Control and Prevention

Hangzhou Center for Diseases Control and Prevention

Jun Li, Haoqiu Wang, Lingfeng Mao, Hua Yu, Xinfen Yu, Zhou Sun, Xin Qian, Shuchang Chen, Junfang Chen, Xuchu Wang

Jun Li, Haoqiu Wang, Lingfeng Mao, Hua Yu, Xinfen Yu, Zhou Sun, Xin Qian, Shuchang Chen, Junfang Chen, Xuchu Wang

Jun Li, Haoqiu Wang, Lingfeng Mao, Hua Yu, Xinfen Yu, Zhou Sun, Xin Qian, Shuchang Chen, Junfang Chen, Xuchu Wang

Jun Li, Haoqiu Wang, Lingfeng Mao, Hua Yu, Xinfen Yu, Zhou Sun, Xin Qian, Shuchang Chen, Junfang Chen, Xuchu Wang

Jun Li, Haoqiu Wang, Lingfeng Mao, Hua Yu, Xinfen Yu, Zhou Sun, Xin Qian, Shuchang Chen, Junfang Chen, Xuchu Wang

Jun Li, Haoqiu Wang, Lingfeng Mao, Hua Yu, Xinfen Yu, Zhou Sun, Xin Qian, Shuchang Chen, Junfang Chen, Xuchu Wang

Jun Li, Haoqiu Wang, Lingfeng Mao, Hua Yu, Xinfen Yu, Zhou Sun, Xin Qian, Shuchang Chen, Junfang Chen, Xuchu Wang

Jun Li, Haoqiu Wang, Lingfeng Mao, Hua Yu, Xinfen Yu, Zhou Sun, Xin Qian, Shuchang Chen, Junfang Chen, Xuchu Wang

EPI_ISL_421235

EPI_ISL_421236

EPI_ISL_421237

EPI_ISL_421238

EPI_ISL_421239

EPI_ISL_421240

EPI_ISL_421241

hCoV-

19/Hangzhou/HZCDC0013/20

20

hCoV-

19/Hangzhou/HZCDC0012/20

20

hCoV-19/Jiujiang/JX22/2020

hCoV-

19/Nanchang/JX174/2020

hCoV-

19/Shangrao/JX105/2020

hCoV-

19/Shangrao/JX1948/2020

hCoV-

19/Nanchang/JX177/2020

Asia / China / Hangzhou

Asia / China / Hangzhou

Asia / China / Jiangxi / Jiujiang

Asia / China / Jiangxi / Nanchang

Asia / China /

Jiangxi /

Shangrao

Asia / China /

Jiangxi /

Shangrao

Asia / China / Jiangxi / Nanchang

2020-01-20

2020-01-20

2020-01-22

2020-01-29

2020-01-26

2020-02-05

2020-01-30

Hangzhou Center for Diseases Control and Prevention

Hangzhou Center for Diseases Control and Prevention

Jiangxi Province Center for Disease Control and Prevention

Jiangxi Province Center for Disease Control and Prevention

Jiangxi Province Center for Disease Control and Prevention

Jiangxi Province Center for Disease Control and Prevention

Jiangxi Province Center for Disease Control and Prevention

Hangzhou Center for Diseases Control and Prevention

Hangzhou Center for Diseases Control and Prevention

Jiangxi Province Center for Disease Control and Prevention

Jiangxi Province Center for Disease Control and Prevention

Jiangxi Province Center for Disease Control and Prevention

Jiangxi Province Center for Disease Control and Prevention

Jiangxi Province Center for Disease Control and Prevention

Jun Li, Haoqiu Wang, Lingfeng Mao, Hua Yu, Xinfen Yu, Zhou Sun, Xin Qian, Shuchang Chen, Junfang Chen, Xuchu Wang

Jun Li, Haoqiu Wang, Lingfeng Mao, Hua Yu, Xinfen Yu, Zhou Sun, Xin Qian, Shuchang Chen, Junfang Chen, Xuchu Wang

JianXiong Li,Ying Xiong,Tian Gong,Yong Shi,Jun Zhou,Fang Xiao,ShiWen Liu,XiaoQing Liu,Gang Xu,DaJin Xiao,Xin Ran,YanNi Zhang

JianXiong Li,Ying Xiong,Tian Gong,Yong Shi,Jun Zhou,Fang Xiao,ShiWen Liu,XiaoQing Liu,Gang Xu,DaJin Xiao,Xin Ran,YanNi Zhang

JianXiong Li,Ying Xiong,Tian Gong,Yong Shi,Jun Zhou,Fang Xiao,ShiWen Liu,XiaoQing Liu,Gang Xu,DaJin Xiao,Xin Ran,YanNi Zhang

JianXiong Li,Ying Xiong,Tian Gong,Yong Shi,Jun Zhou,Fang Xiao,ShiWen Liu,XiaoQing Liu,Gang Xu,DaJin Xiao,Xin Ran,YanNi Zhang

JianXiong Li,Ying Xiong,Tian Gong,Yong Shi,Jun Zhou,Fang Xiao,ShiWen Liu,XiaoQing Liu,Gang Xu,DaJin Xiao,Xin Ran,YanNi Zhang

EPI_ISL_421242

EPI_ISL_421243

EPI_ISL_421244

EPI_ISL_421245

EPI_ISL_421246

EPI_ISL_421247

EPI_ISL_421248

hCoV-19/Ganzhou/JX81/2020

hCoV-

19/Nanchang/JX14/2020

hCoV-19/Shangrao/JX29/2020

hCoV-

19/Nanchang/JX90/2020

hCoV-

19/Nanchang/JXN3T4/2020

hCoV-

19/Nanchang/JX149/2020

hCoV-

19/Nanchang/JX39/2020

Asia / China / Jiangxi / Ganzhou

Asia / China / Jiangxi / Nanchang

Asia / China /

Jiangxi /

Shangrao

Asia / China / Jiangxi / Nanchang

Asia / China / Jiangxi / Nanchang

Asia / China / Jiangxi / Nanchang

Asia / China / Jiangxi / Nanchang

2020-01-25

2020-01-21

2020-01-22

2020-01-25

2020-02-26

2020-01-27

2020-01-23

Jiangxi Province Center for Disease Control and Prevention

Jiangxi Province Center for Disease Control and Prevention

Jiangxi Province Center for Disease Control and Prevention

Jiangxi Province Center for Disease Control and Prevention

Jiangxi Province Center for Disease Control and Prevention

Jiangxi Province Center for Disease Control and Prevention

Jiangxi Province Center for Disease Control and Prevention

Jiangxi Province Center for Disease Control and Prevention

Jiangxi Province Center for Disease Control and Prevention

Jiangxi Province Center for Disease Control and Prevention

Jiangxi Province Center for Disease Control and Prevention

Jiangxi Province Center for Disease Control and Prevention

Jiangxi Province Center for Disease Control and Prevention

Jiangxi Province Center for Disease Control and Prevention

JianXiong Li,Ying Xiong,Tian Gong,Yong Shi,Jun Zhou,Fang Xiao,ShiWen Liu,XiaoQing Liu,Gang Xu,DaJin Xiao,Xin Ran,YanNi Zhang

JianXiong Li,Ying Xiong,Tian Gong,Yong Shi,Jun Zhou,Fang Xiao,ShiWen Liu,XiaoQing Liu,Gang Xu,DaJin Xiao,Xin Ran,YanNi Zhang

JianXiong Li,Ying Xiong,Tian Gong,Yong Shi,Jun Zhou,Fang Xiao,ShiWen Liu,XiaoQing Liu,Gang Xu,DaJin Xiao,Xin Ran,YanNi Zhang

JianXiong Li,Ying Xiong,Tian Gong,Yong Shi,Jun Zhou,Fang Xiao,ShiWen Liu,XiaoQing Liu,Gang Xu,DaJin Xiao,Xin Ran,YanNi Zhang

JianXiong Li,Ying Xiong,Tian Gong,Yong Shi,Jun Zhou,Fang Xiao,ShiWen Liu,XiaoQing Liu,Gang Xu,DaJin Xiao,Xin Ran,YanNi Zhang

JianXiong Li,Ying Xiong,Tian Gong,Yong Shi,Jun Zhou,Fang Xiao,ShiWen Liu,XiaoQing Liu,Gang Xu,DaJin Xiao,Xin Ran,YanNi Zhang

JianXiong Li,Ying Xiong,Tian Gong,Yong Shi,Jun Zhou,Fang Xiao,ShiWen Liu,XiaoQing Liu,Gang Xu,DaJin Xiao,Xin Ran,YanNi Zhang

EPI_ISL_421249

EPI_ISL_421250

EPI_ISL_421251

EPI_ISL_421252

EPI_ISL_421253

EPI_ISL_421254

EPI_ISL_421256

hCoV-19/Xinyu/JX122/2020

hCoV-

19/Shangrao/JX1177/2020

hCoV-

19/Shangrao/JX1176/2020

hCoV-19/Pingxiang/JX5/2020

hCoV-19/Jian/JX129/2020

hCoV-

19/Shangrao/JX1178/2020

hCoV-19/Jian/JX169/2020

Asia / China / Jiangxi / Xinyu

Asia / China /

Jiangxi /

Shangrao

Asia / China /

Jiangxi /

Shangrao

Asia / China /

Jiangxi /

Pingxiang

Asia / China / Jiangxi / Jian

Asia / China /

Jiangxi /

Shangrao

Asia / China / Jiangxi / Jian

2020-01-25

2020-02-03

2020-02-03

2020-01-11

2020-01-26

2020-02-03

2020-01-27

Jiangxi Province Center for Disease Control and Prevention

Jiangxi Province Center for Disease Control and Prevention

Jiangxi Province Center for Disease Control and Prevention

Jiangxi Province Center for Disease Control and Prevention

Jiangxi Province Center for Disease Control and Prevention

Jiangxi Province Center for Disease Control and Prevention

Jiangxi Province Center for Disease Control and Prevention

Jiangxi Province Center for Disease Control and Prevention

Jiangxi Province Center for Disease Control and Prevention

Jiangxi Province Center for Disease Control and Prevention

Jiangxi Province Center for Disease Control and Prevention

Jiangxi Province Center for Disease Control and Prevention

Jiangxi Province Center for Disease Control and Prevention

Jiangxi Province Center for Disease Control and Prevention

JianXiong Li,Ying Xiong,Tian Gong,Yong Shi,Jun Zhou,Fang Xiao,ShiWen Liu,XiaoQing Liu,Gang Xu,DaJin Xiao,Xin Ran,YanNi Zhang

JianXiong Li,Ying Xiong,Tian Gong,Yong Shi,Jun Zhou,Fang Xiao,ShiWen Liu,XiaoQing Liu,Gang Xu,DaJin Xiao,Xin Ran,YanNi Zhang

JianXiong Li,Ying Xiong,Tian Gong,Yong Shi,Jun Zhou,Fang Xiao,ShiWen Liu,XiaoQing Liu,Gang Xu,DaJin Xiao,Xin Ran,YanNi Zhang

JianXiong Li,Ying Xiong,Tian Gong,Yong Shi,Jun Zhou,Fang Xiao,ShiWen Liu,XiaoQing Liu,Gang Xu,DaJin Xiao,Xin Ran,YanNi Zhang

JianXiong Li,Ying Xiong,Tian Gong,Yong Shi,Jun Zhou,Fang Xiao,ShiWen Liu,XiaoQing Liu,Gang Xu,DaJin Xiao,Xin Ran,YanNi Zhang

JianXiong Li,Ying Xiong,Tian Gong,Yong Shi,Jun Zhou,Fang Xiao,ShiWen Liu,XiaoQing Liu,Gang Xu,DaJin Xiao,Xin Ran,YanNi Zhang

JianXiong Li,Ying Xiong,Tian Gong,Yong Shi,Jun Zhou,Fang Xiao,ShiWen Liu,XiaoQing Liu,Gang Xu,DaJin Xiao,Xin Ran,YanNi Zhang

EPI_ISL_421257

EPI_ISL_421258

EPI_ISL_421259

EPI_ISL_421260

EPI_ISL_421261

EPI_ISL_421262

EPI_ISL_421272

hCoV-

19/Shangrao/JX1215/2020

hCoV-

19/Shangrao/JX1974/2020

hCoV-

19/Pingxiang/JX151/2020

hCoV-19/Xinyu/JX124/2020

hCoV-

19/Nanchang/JX176/2020

hCoV-

19/Nanchang/JX155/2020

hCoV-19/USA/WY-

WYPHL001/2020

Asia / China /

Jiangxi /

Shangrao

Asia / China /

Jiangxi /

Shangrao

Asia / China /

Jiangxi /

Pingxiang

Asia / China / Jiangxi / Xinyu

Asia / China / Jiangxi / Nanchang

Asia / China / Jiangxi / Nanchang

North America / USA / Wyoming

2020-02-02

2020-02-08

2020-01-27

2020-01-26

2020-01-29

2020-01-29

2020-03-10

Jiangxi Province Center for Disease Control and Prevention

Jiangxi Province Center for Disease Control and Prevention

Jiangxi Province Center for Disease Control and Prevention

Jiangxi Province Center for Disease Control and Prevention

Jiangxi Province Center for Disease Control and Prevention

Jiangxi Province Center for Disease Control and Prevention

Wyoming Public

Health

Laboratory

Jiangxi Province Center for Disease Control and Prevention

Jiangxi Province Center for Disease Control and Prevention

Jiangxi Province Center for Disease Control and Prevention

Jiangxi Province Center for Disease Control and Prevention

Jiangxi Province Center for Disease Control and Prevention

Jiangxi Province Center for Disease Control and Prevention

Center for Global

Health, University of New Mexico Health Sciences Center

JianXiong Li,Ying Xiong,Tian Gong,Yong Shi,Jun Zhou,Fang Xiao,ShiWen Liu,XiaoQing Liu,Gang Xu,DaJin Xiao,Xin Ran,YanNi Zhang

JianXiong Li,Ying Xiong,Tian Gong,Yong Shi,Jun Zhou,Fang Xiao,ShiWen Liu,XiaoQing Liu,Gang Xu,DaJin Xiao,Xin Ran,YanNi Zhang

JianXiong Li,Ying Xiong,Tian Gong,Yong Shi,Jun Zhou,Fang Xiao,ShiWen Liu,XiaoQing Liu,Gang Xu,DaJin Xiao,Xin Ran,YanNi Zhang

JianXiong Li,Ying Xiong,Tian Gong,Yong Shi,Jun Zhou,Fang Xiao,ShiWen Liu,XiaoQing Liu,Gang Xu,DaJin Xiao,Xin Ran,YanNi Zhang

JianXiong Li,Ying Xiong,Tian Gong,Yong Shi,Jun Zhou,Fang Xiao,ShiWen Liu,XiaoQing Liu,Gang Xu,DaJin Xiao,Xin Ran,YanNi Zhang

JianXiong Li,Ying Xiong,Tian Gong,Yong Shi,Jun Zhou,Fang Xiao,ShiWen Liu,XiaoQing Liu,Gang Xu,DaJin Xiao,Xin Ran,YanNi Zhang

Daryl Domman, Kurt Schwalm, Rob Christensen, Wanda Manley, Cari Sloma, Noah Hull, Darrell Dinwiddie

EPI_ISL_422407

EPI_ISL_422408

EPI_ISL_422409

EPI_ISL_422411

EPI_ISL_422412

EPI_ISL_422428

hCoV-19/Taiwan/NTU04/2020

hCoV-19/Taiwan/NTU05/2020

hCoV-19/Taiwan/NTU06/2020

hCoV-19/Taiwan/NTU08/2020

hCoV-19/Taiwan/NTU09/2020

hCoV-19/Singapore/35/2020

Asia / Taiwan /

Taipei

Asia / Taiwan /

Taipei

Asia / Taiwan /

Taipei

Asia / Taiwan /

Taipei

Asia / Taiwan /

Taipei

Asia / Singapore

2020-03-04

2020-03-13

2020-03-13

2020-03-14

2020-03-14

2020-02-06

| Department of |  | Microbial Genomics |
| --- | --- | --- |
| Laboratory |  | Core Lab, National |
| Medicine, |  | Taiwan University |
| National Taiwan |  | Centers of Genomic |
| University |  | and Precision |
| Hospital |  | Medicine |
|  |  |  |
| Department of |  | Microbial Genomics |
| Laboratory |  | Core Lab, National |
| Medicine, |  | Taiwan University |
| National Taiwan |  | Centers of Genomic |
| University |  | and Precision |
| Hospital |  | Medicine |
|  |  |  |
| Department of |  | Microbial Genomics |
| Laboratory |  | Core Lab, National |
| Medicine, |  | Taiwan University |
| National Taiwan |  | Centers of Genomic |
| University |  | and Precision |
| Hospital |  | Medicine |
|  |  |  |
| Department of |  | Microbial Genomics |
| Laboratory |  | Core Lab, National |
| Medicine, |  | Taiwan University |
| National Taiwan |  | Centers of Genomic |
| University |  | and Precision |
| Hospital |  | Medicine |
|  |  |  |
| Department of |  | Microbial Genomics |
| Laboratory |  | Core Lab, National |
| Medicine, |  | Taiwan University |
| National Taiwan |  | Centers of Genomic |
| University |  | and Precision |
| Hospital |  | Medicine |
|  |  |  |
| National Public |  |  |
| Health |  | National Public |
| Laboratory, |  | Health Laboratory, |
| National Centre |  | National Centre for |
| for Infectious |  | Infectious Diseases |
| Diseases |  |  |
|  |  |  |

Shiou-Hwei Yeh, You-Yu Lin, Ya-Yun Lai, Chiao-Ling Li, Shan-Chwen Chang, Pei-Jer Chen, Sui-Yuan Chang

Shiou-Hwei Yeh, You-Yu Lin, Ya-Yun Lai, Chiao-Ling Li, Shan-Chwen Chang, Pei-Jer Chen, Sui-Yuan Chang

Shiou-Hwei Yeh, You-Yu Lin, Ya-Yun Lai, Chiao-Ling Li, Shan-Chwen Chang, Pei-Jer Chen, Sui-Yuan Chang

Shiou-Hwei Yeh, You-Yu Lin, Ya-Yun Lai, Chiao-Ling Li, Shan-Chwen Chang, Pei-Jer Chen, Sui-Yuan Chang

Shiou-Hwei Yeh, You-Yu Lin, Ya-Yun Lai, Chiao-Ling Li, Shan-Chwen Chang, Pei-Jer Chen, Sui-Yuan Chang

Mak TM, Octavia S, Cui L, Lin RTP

EPI_ISL_422429

EPI_ISL_424360

EPI_ISL_424855

EPI_ISL_424856

EPI_ISL_424857

hCoV-19/Singapore/36/2020

hCoV-19/Beijing/Wuhan_IME-BJ07/2020

hCoV-19/USA/FL_7169/2020

hCoV-19/USA/FL_9656/2020

hCoV-19/USA/FL_1177/2020

Asia / Singapore

Asia / China /

Beijing

North America / USA / Florida

North America / USA / Florida

North America / USA / Florida

2020-02-16

2020-01-29

2020-03-02

2020-03-06

2020-03-06

National Public

Health

Laboratory,

National Centre

for Infectious

Diseases

unknown

FL Bureau of Public Health Laboratories-Tampa

FL Bur. of Public

Health

Laboratories-

Jacksonville

FL Bur. of Public

Health

Laboratories-

Jacksonville

National Public Health Laboratory, National Centre for Infectious Diseases

Beijing Institute of Microbiology and Epidemiology

Pathogen Discovery, Respiratory Viruses Branch, Division of Viral Diseases, Centers for Disease Control and Prevention

Pathogen Discovery, Respiratory Viruses Branch, Division of Viral Diseases, Centers for Disease Control and Prevention

Pathogen Discovery, Respiratory Viruses Branch, Division of Viral Diseases, Centers for Disease Control and Prevention

Mak TM, Octavia S, Cui L, Lin RTP

Fan,H., Qin,E., Wu,Y., Guo,Y., Zhang,X., Yong,Y., Hou,J., Xu,Z., Mu,J., Teng,Y., Mi,Z., Yang,R., Song,Y., Li,B. and Cui,Y.

Yan Li, Krista Queen, Clinton R. Paden, Rachel Marine, Anna

Uehara, Ying Tao, Jing Zhang, Haibin Wang, Mary S. Keckler, Alison S. Laufer Halpin, Christopher A. Elkins, Suxiang Tong

Yan Li, Krista Queen, Clinton R. Paden, Rachel Marine, Anna

Uehara, Ying Tao, Jing Zhang, Haibin Wang, Mary S. Keckler, Alison S. Laufer Halpin, Christopher A. Elkins, Suxiang Tong

Yan Li, Krista Queen, Clinton R. Paden, Rachel Marine, Anna

Uehara, Ying Tao, Jing Zhang, Haibin Wang, Mary S. Keckler, Alison S. Laufer Halpin, Christopher A. Elkins, Suxiang Tong

EPI_ISL_424858

EPI_ISL_424859

EPI_ISL_424861

EPI_ISL_424864

hCoV-19/USA/GA_1621/2020

hCoV-19/USA/GA_1847/2020

hCoV-19/USA/GA_2118/2020

hCoV-19/USA/GA_2343/2020

North America / USA / Georgia

North America / USA / Georgia

North America / USA / Georgia

North America / USA / Georgia

|  |  |  | Pathogen Discovery, |  |
| --- | --- | --- | --- | --- |
|  | GA Department |  | Respiratory Viruses |  |
|  |  |  | Branch, Division of |  |
| 2020-03-04 | of Public Health |  | Viral Diseases, |  |
|  | Laboratory |  | Centers for Disease |  |
|  |  |  | Control and |  |
|  |  |  | Prevention |  |
|  |  |  |  |  |
|  |  |  | Pathogen Discovery, |  |
|  |  |  | Respiratory Viruses |  |
| 2020-03-05 | GA Department |  | Branch, Division of |  |
|  | of Public Health |  | Viral Diseases, |  |
|  | Laboratory |  | Centers for Disease |  |
|  |  |  | Control and |  |
|  |  |  | Prevention |  |
|  |  |  |  |  |
|  |  |  | Pathogen Discovery, |  |
|  | GA Department |  | Respiratory Viruses |  |
|  |  |  | Branch, Division of |  |
| 2020-03-08 | of Public Health |  | Viral Diseases, |  |
|  | Laboratory |  | Centers for Disease |  |
|  |  |  | Control and |  |
|  |  |  | Prevention |  |
|  |  |  |  |  |
|  |  |  | Pathogen Discovery, |  |
|  |  |  | Respiratory Viruses |  |
| 2020-03-10 | GA Department |  | Branch, Division of |  |
|  | of Public Health |  | Viral Diseases, |  |
|  | Laboratory |  | Centers for Disease |  |
|  |  |  | Control and |  |
|  |  |  | Prevention |  |
|  |  |  |  |  |

Yan Li, Krista Queen, Clinton R. Paden, Rachel Marine, Anna

Uehara, Ying Tao, Jing Zhang, Haibin Wang, Mary S. Keckler, Alison S. Laufer Halpin, Christopher A. Elkins, Suxiang Tong

Yan Li, Krista Queen, Clinton R. Paden, Rachel Marine, Anna

Uehara, Ying Tao, Jing Zhang, Haibin Wang, Mary S. Keckler, Alison S. Laufer Halpin, Christopher A. Elkins, Suxiang Tong

Yan Li, Krista Queen, Clinton R. Paden, Rachel Marine, Anna

Uehara, Ying Tao, Jing Zhang, Haibin Wang, Mary S. Keckler, Alison S. Laufer Halpin, Christopher A. Elkins, Suxiang Tong

Yan Li, Krista Queen, Clinton R. Paden, Rachel Marine, Anna

Uehara, Ying Tao, Jing Zhang, Haibin Wang, Mary S. Keckler, Alison S. Laufer Halpin, Christopher A. Elkins, Suxiang Tong

EPI_ISL_424865

EPI_ISL_424866

EPI_ISL_424867

EPI_ISL_424868

hCoV-19/USA/IA_6394/2020

hCoV-19/USA/IN_2001/2020

hCoV-19/USA/KS_4126/2020

hCoV-19/USA/LA_0842/2020

North America /

USA / Iowa

North America / USA / Indiana

North America / USA / Kansas

North America / USA / Louisiana

|  |  |  | Pathogen Discovery, |  |
| --- | --- | --- | --- | --- |
|  | IA State |  | Respiratory Viruses |  |
|  |  |  | Branch, Division of |  |
| 2020-03-08 | Hygienic |  | Viral Diseases, |  |
|  | Laboratory |  | Centers for Disease |  |
|  |  |  | Control and |  |
|  |  |  | Prevention |  |
|  |  |  |  |  |
|  | IN State |  | Pathogen Discovery, |  |
|  |  |  | Respiratory Viruses |  |
| 2020-03-09 | Department of |  | Branch, Division of |  |
|  | Health |  | Viral Diseases, |  |
|  | Laboratory |  | Centers for Disease |  |
|  | Services |  | Control and |  |
|  |  |  | Prevention |  |
|  |  |  |  |  |
|  |  |  | Pathogen Discovery, |  |
|  | KS Health and |  | Respiratory Viruses |  |
|  |  |  | Branch, Division of |  |
| 2020-03-06 | Environmental |  | Viral Diseases, |  |
|  | Laboratories |  | Centers for Disease |  |
|  |  |  | Control and |  |
|  |  |  | Prevention |  |
|  |  |  |  |  |
|  |  |  | Pathogen Discovery, |  |
|  |  |  | Respiratory Viruses |  |
| 2020-03-09 | LA Office of |  | Branch, Division of |  |
|  | Public Health |  | Viral Diseases, |  |
|  | Laboratories |  | Centers for Disease |  |
|  |  |  | Control and |  |
|  |  |  | Prevention |  |
|  |  |  |  |  |

Yan Li, Krista Queen, Clinton R. Paden, Rachel Marine, Anna

Uehara, Ying Tao, Jing Zhang, Haibin Wang, Mary S. Keckler, Alison S. Laufer Halpin, Christopher A. Elkins, Suxiang Tong

Yan Li, Krista Queen, Clinton R. Paden, Rachel Marine, Anna

Uehara, Ying Tao, Jing Zhang, Haibin Wang, Mary S. Keckler, Alison S. Laufer Halpin, Christopher A. Elkins, Suxiang Tong

Yan Li, Krista Queen, Clinton R. Paden, Rachel Marine, Anna

Uehara, Ying Tao, Jing Zhang, Haibin Wang, Mary S. Keckler, Alison S. Laufer Halpin, Christopher A. Elkins, Suxiang Tong

Yan Li, Krista Queen, Clinton R. Paden, Rachel Marine, Anna

Uehara, Ying Tao, Jing Zhang, Haibin Wang, Mary S. Keckler, Alison S. Laufer Halpin, Christopher A. Elkins, Suxiang Tong

EPI_ISL_424869

EPI_ISL_424870

EPI_ISL_424871

EPI_ISL_424872

hCoV-19/USA/MD_0026/2020

hCoV-19/USA/MO_4470/2020

hCoV-19/USA/NC_6999/2020

hCoV-19/USA/NC_0024/2020

North America / USA / Maryland

North America / USA / Missouri

North America / USA / North Carolina

North America / USA / North Carolina

|  |  |  | Pathogen Discovery, |  |
| --- | --- | --- | --- | --- |
|  | MD DOH |  | Respiratory Viruses |  |
|  |  |  | Branch, Division of |  |
| 2020-03-04 | Laboratories |  | Viral Diseases, |  |
|  | Administration |  | Centers for Disease |  |
|  |  |  | Control and |  |
|  |  |  | Prevention |  |
|  |  |  |  |  |
|  |  |  | Pathogen Discovery, |  |
|  |  |  | Respiratory Viruses |  |
| 2020-03-06 | MO State Public |  | Branch, Division of |  |
|  | Health |  | Viral Diseases, |  |
|  | Laboratory |  | Centers for Disease |  |
|  |  |  | Control and |  |
|  |  |  | Prevention |  |
|  |  |  |  |  |
|  |  |  | Pathogen Discovery, |  |
|  | NC State |  | Respiratory Viruses |  |
|  |  |  | Branch, Division of |  |
| 2020-03-06 | Laboratory of |  | Viral Diseases, |  |
|  | Public Health |  | Centers for Disease |  |
|  |  |  | Control and |  |
|  |  |  | Prevention |  |
|  |  |  |  |  |
|  |  |  | Pathogen Discovery, |  |
|  |  |  | Respiratory Viruses |  |
| 2020-03-08 | NC State |  | Branch, Division of |  |
|  | Laboratory of |  | Viral Diseases, |  |
|  | Public Health |  | Centers for Disease |  |
|  |  |  | Control and |  |
|  |  |  | Prevention |  |
|  |  |  |  |  |

Yan Li, Krista Queen, Clinton R. Paden, Rachel Marine, Anna

Uehara, Ying Tao, Jing Zhang, Haibin Wang, Mary S. Keckler, Alison S. Laufer Halpin, Christopher A. Elkins, Suxiang Tong

Yan Li, Krista Queen, Clinton R. Paden, Rachel Marine, Anna

Uehara, Ying Tao, Jing Zhang, Haibin Wang, Mary S. Keckler, Alison S. Laufer Halpin, Christopher A. Elkins, Suxiang Tong

Yan Li, Krista Queen, Clinton R. Paden, Rachel Marine, Anna

Uehara, Ying Tao, Jing Zhang, Haibin Wang, Mary S. Keckler, Alison S. Laufer Halpin, Christopher A. Elkins, Suxiang Tong

Yan Li, Krista Queen, Clinton R. Paden, Rachel Marine, Anna

Uehara, Ying Tao, Jing Zhang, Haibin Wang, Mary S. Keckler, Alison S. Laufer Halpin, Christopher A. Elkins, Suxiang Tong

EPI_ISL_424873

EPI_ISL_424874

EPI_ISL_424875

EPI_ISL_424876

hCoV-19/USA/NC_0031/2020

hCoV-19/USA/NE_6605/2020

hCoV-19/USA/NE_7025/2020

hCoV-19/USA/NH_0029/2020

North America / USA / North Carolina

North America / USA / Nebraska

North America / USA / Nebraska

North America / USA / New Hampshire

|  |  |  | Pathogen Discovery, |  |
| --- | --- | --- | --- | --- |
|  | NC State |  | Respiratory Viruses |  |
|  |  |  | Branch, Division of |  |
| 2020-03-07 | Laboratory of |  | Viral Diseases, |  |
|  | Public Health |  | Centers for Disease |  |
|  |  |  | Control and |  |
|  |  |  | Prevention |  |
|  |  |  |  |  |
|  |  |  | Pathogen Discovery, |  |
|  |  |  | Respiratory Viruses |  |
| 2020-03-05 | NE Public |  | Branch, Division of |  |
|  | Health |  | Viral Diseases, |  |
|  | Laboratory |  | Centers for Disease |  |
|  |  |  | Control and |  |
|  |  |  | Prevention |  |
|  |  |  |  |  |
|  |  |  | Pathogen Discovery, |  |
|  | NE Public |  | Respiratory Viruses |  |
|  |  |  | Branch, Division of |  |
| 2020-03-07 | Health |  | Viral Diseases, |  |
|  | Laboratory |  | Centers for Disease |  |
|  |  |  | Control and |  |
|  |  |  | Prevention |  |
|  |  |  |  |  |
|  | NH Dept. of |  | Pathogen Discovery, |  |
|  |  |  | Respiratory Viruses |  |
| 2020-03-06 | Health and |  | Branch, Division of |  |
|  | Human Services |  | Viral Diseases, |  |
|  | Public Health |  | Centers for Disease |  |
|  | Labs |  | Control and |  |
|  |  |  | Prevention |  |
|  |  |  |  |  |

Yan Li, Krista Queen, Clinton R. Paden, Rachel Marine, Anna

Uehara, Ying Tao, Jing Zhang, Haibin Wang, Mary S. Keckler, Alison S. Laufer Halpin, Christopher A. Elkins, Suxiang Tong

Yan Li, Krista Queen, Clinton R. Paden, Rachel Marine, Anna

Uehara, Ying Tao, Jing Zhang, Haibin Wang, Mary S. Keckler, Alison S. Laufer Halpin, Christopher A. Elkins, Suxiang Tong

Yan Li, Krista Queen, Clinton R. Paden, Rachel Marine, Anna

Uehara, Ying Tao, Jing Zhang, Haibin Wang, Mary S. Keckler, Alison S. Laufer Halpin, Christopher A. Elkins, Suxiang Tong

Yan Li, Krista Queen, Clinton R. Paden, Rachel Marine, Anna

Uehara, Ying Tao, Jing Zhang, Haibin Wang, Mary S. Keckler, Alison S. Laufer Halpin, Christopher A. Elkins, Suxiang Tong

EPI_ISL_424877

EPI_ISL_424878

EPI_ISL_424879

EPI_ISL_424880

hCoV-19/USA/NH_0033/2020

hCoV-19/USA/NJ_3592/2020

hCoV-19/USA/NV_0052/2020

hCoV-19/USA/OH_0023/2020

North America / USA / New Hampshire

North America / USA / New Jersey

North America / USA / Nevada

North America /

USA / Ohio

2020-03-06

2020-03-04

2020-03-09

2020-03-08

NH Dept. of Health and Human Services Public Health Labs

NJ Public Health

and

Environmental

Laboratories

NV-Southern

Nevada Public

Health

Laboratory

OH Department

of Health

Laboratory

Pathogen Discovery, Respiratory Viruses Branch, Division of Viral Diseases, Centers for Disease Control and Prevention

Pathogen Discovery, Respiratory Viruses Branch, Division of Viral Diseases, Centers for Disease Control and Prevention

Pathogen Discovery, Respiratory Viruses Branch, Division of Viral Diseases, Centers for Disease Control and Prevention

Pathogen Discovery, Respiratory Viruses Branch, Division of Viral Diseases, Centers for Disease Control and Prevention

Yan Li, Krista Queen, Clinton R. Paden, Rachel Marine, Anna

Uehara, Ying Tao, Jing Zhang, Haibin Wang, Mary S. Keckler, Alison S. Laufer Halpin, Christopher A. Elkins, Suxiang Tong

Yan Li, Krista Queen, Clinton R. Paden, Rachel Marine, Anna

Uehara, Ying Tao, Jing Zhang, Haibin Wang, Mary S. Keckler, Alison S. Laufer Halpin, Christopher A. Elkins, Suxiang Tong

Yan Li, Krista Queen, Clinton R. Paden, Rachel Marine, Anna

Uehara, Ying Tao, Jing Zhang, Haibin Wang, Mary S. Keckler, Alison S. Laufer Halpin, Christopher A. Elkins, Suxiang Tong

Yan Li, Krista Queen, Clinton R. Paden, Rachel Marine, Anna

Uehara, Ying Tao, Jing Zhang, Haibin Wang, Mary S. Keckler, Alison S. Laufer Halpin, Christopher A. Elkins, Suxiang Tong

North America /

EPI_ISL_424881 hCoV-19/USA/PA_1802/2020 USA / 2020-03-05

Pennsylvania

North America /

EPI_ISL_424882 hCoV-19/USA/PA_1881/2020 USA / 2020-03-05

Pennsylvania

North America /

EPI_ISL_424883 hCoV-19/USA/PA_2317/2020 USA / 2020-03-07

Pennsylvania

North America /

EPI_ISL_424884 hCoV-19/USA/PA_2937/2020 USA / 2020-03-08

Pennsylvania

PA Department of Health, Bureau of Laboratories

PA Department of Health, Bureau of Laboratories

PA Department of Health, Bureau of Laboratories

PA Department of Health, Bureau of Laboratories

Pathogen Discovery, Respiratory Viruses Branch, Division of Viral Diseases, Centers for Disease Control and Prevention

Pathogen Discovery, Respiratory Viruses Branch, Division of Viral Diseases, Centers for Disease Control and Prevention

Pathogen Discovery, Respiratory Viruses Branch, Division of Viral Diseases, Centers for Disease Control and Prevention

Pathogen Discovery, Respiratory Viruses Branch, Division of Viral Diseases, Centers for Disease Control and Prevention

Yan Li, Krista Queen, Clinton R. Paden, Rachel Marine, Anna

Uehara, Ying Tao, Jing Zhang, Haibin Wang, Mary S. Keckler, Alison S. Laufer Halpin, Christopher A. Elkins, Suxiang Tong

Yan Li, Krista Queen, Clinton R. Paden, Rachel Marine, Anna

Uehara, Ying Tao, Jing Zhang, Haibin Wang, Mary S. Keckler, Alison S. Laufer Halpin, Christopher A. Elkins, Suxiang Tong

Yan Li, Krista Queen, Clinton R. Paden, Rachel Marine, Anna

Uehara, Ying Tao, Jing Zhang, Haibin Wang, Mary S. Keckler, Alison S. Laufer Halpin, Christopher A. Elkins, Suxiang Tong

Yan Li, Krista Queen, Clinton R. Paden, Rachel Marine, Anna

Uehara, Ying Tao, Jing Zhang, Haibin Wang, Mary S. Keckler, Alison S. Laufer Halpin, Christopher A. Elkins, Suxiang Tong

North America /

EPI_ISL_424885 hCoV-19/USA/PA_4395/2020 USA / 2020-03-09

Pennsylvania

North America /

EPI_ISL_424886 hCoV-19/USA/PA_4405/2020 USA / 2020-03-06

Pennsylvania

North America /

EPI_ISL_424887 hCoV-19/USA/RI_0702/2020 USA / Rhode 2020-03-05

Island

North America /

EPI_ISL_424888 hCoV-19/USA/SC_6370/2020 USA / South 2020-03-08

Carolina

PA Department of Health, Bureau of Laboratories

PA Department of Health, Bureau of Laboratories

RI State Health Laboratories

SC Dept of Health and Env. Control-Bureau of Laboratories

Pathogen Discovery, Respiratory Viruses Branch, Division of Viral Diseases, Centers for Disease Control and Prevention

Pathogen Discovery, Respiratory Viruses Branch, Division of Viral Diseases, Centers for Disease Control and Prevention

Pathogen Discovery, Respiratory Viruses Branch, Division of Viral Diseases, Centers for Disease Control and Prevention

Pathogen Discovery, Respiratory Viruses Branch, Division of Viral Diseases, Centers for Disease Control and Prevention

Yan Li, Krista Queen, Clinton R. Paden, Rachel Marine, Anna

Uehara, Ying Tao, Jing Zhang, Haibin Wang, Mary S. Keckler, Alison S. Laufer Halpin, Christopher A. Elkins, Suxiang Tong

Yan Li, Krista Queen, Clinton R. Paden, Rachel Marine, Anna

Uehara, Ying Tao, Jing Zhang, Haibin Wang, Mary S. Keckler, Alison S. Laufer Halpin, Christopher A. Elkins, Suxiang Tong

Yan Li, Krista Queen, Clinton R. Paden, Rachel Marine, Anna

Uehara, Ying Tao, Jing Zhang, Haibin Wang, Mary S. Keckler, Alison S. Laufer Halpin, Christopher A. Elkins, Suxiang Tong

Yan Li, Krista Queen, Clinton R. Paden, Rachel Marine, Anna

Uehara, Ying Tao, Jing Zhang, Haibin Wang, Mary S. Keckler, Alison S. Laufer Halpin, Christopher A. Elkins, Suxiang Tong

EPI_ISL_424889

EPI_ISL_424890

EPI_ISL_424891

EPI_ISL_424892

hCoV-19/USA/UT_8199/2020

hCoV-19/USA/UT_8906/2020

hCoV-19/USA/VA_6352/2020

hCoV-19/USA/VA_6377/2020

North America /

USA / Utah

North America /

USA / Utah

North America / USA / Virginia

North America / USA / Virginia

2020-03-05

2020-03-07

2020-03-08

2020-03-08

UT-Unified State Labs: Public Health Utah DOH

UT-Unified State Labs: Public Health Utah DOH

VA-Division of Consolidated Laboratory Services

VA-Division of Consolidated Laboratory Services

Pathogen Discovery, Respiratory Viruses Branch, Division of Viral Diseases, Centers for Disease Control and Prevention

Pathogen Discovery, Respiratory Viruses Branch, Division of Viral Diseases, Centers for Disease Control and Prevention

Pathogen Discovery, Respiratory Viruses Branch, Division of Viral Diseases, Centers for Disease Control and Prevention

Pathogen Discovery, Respiratory Viruses Branch, Division of Viral Diseases, Centers for Disease Control and Prevention

Yan Li, Krista Queen, Clinton R. Paden, Rachel Marine, Anna

Uehara, Ying Tao, Jing Zhang, Haibin Wang, Mary S. Keckler, Alison S. Laufer Halpin, Christopher A. Elkins, Suxiang Tong

Yan Li, Krista Queen, Clinton R. Paden, Rachel Marine, Anna

Uehara, Ying Tao, Jing Zhang, Haibin Wang, Mary S. Keckler, Alison S. Laufer Halpin, Christopher A. Elkins, Suxiang Tong

Yan Li, Krista Queen, Clinton R. Paden, Rachel Marine, Anna

Uehara, Ying Tao, Jing Zhang, Haibin Wang, Mary S. Keckler, Alison S. Laufer Halpin, Christopher A. Elkins, Suxiang Tong

Yan Li, Krista Queen, Clinton R. Paden, Rachel Marine, Anna

Uehara, Ying Tao, Jing Zhang, Haibin Wang, Mary S. Keckler, Alison S. Laufer Halpin, Christopher A. Elkins, Suxiang Tong

EPI_ISL_424893

EPI_ISL_424894

EPI_ISL_424895

EPI_ISL_424896

hCoV-19/USA/VA_6382/2020

hCoV-19/USA/VA_6389/2020

hCoV-19/USA/IA_6396/2020

hCoV-19/USA/IA_6395/2020

North America / USA / Virginia

North America / USA / Virginia

North America /

USA / Iowa

North America /

USA / Iowa

2020-03-08

2020-03-08

2020-03-08

2020-03-08

VA-Division of Consolidated Laboratory Services

VA-Division of Consolidated Laboratory Services

IA State

Hygienic

Laboratory

IA State

Hygienic

Laboratory

Pathogen Discovery, Respiratory Viruses Branch, Division of Viral Diseases, Centers for Disease Control and Prevention

Pathogen Discovery, Respiratory Viruses Branch, Division of Viral Diseases, Centers for Disease Control and Prevention

Pathogen Discovery, Respiratory Viruses Branch, Division of Viral Diseases, Centers for Disease Control and Prevention

Pathogen Discovery, Respiratory Viruses Branch, Division of Viral Diseases, Centers for Disease Control and Prevention

Yan Li, Krista Queen, Clinton R. Paden, Rachel Marine, Anna

Uehara, Ying Tao, Jing Zhang, Haibin Wang, Mary S. Keckler, Alison S. Laufer Halpin, Christopher A. Elkins, Suxiang Tong

Yan Li, Krista Queen, Clinton R. Paden, Rachel Marine, Anna

Uehara, Ying Tao, Jing Zhang, Haibin Wang, Mary S. Keckler, Alison S. Laufer Halpin, Christopher A. Elkins, Suxiang Tong

Ying Tao, Clinton R. Paden, Jing Zhang, Krista Queen, Anna Uehara, Yan Li, Haibin Wang, Mary S. Keckler, Alison S. Laufer Halpin, Christopher A. Elkins, Suxiang Tong

Ying Tao, Clinton R. Paden, Jing Zhang, Krista Queen, Anna Uehara, Yan Li, Haibin Wang, Mary S. Keckler, Alison S. Laufer Halpin, Christopher A. Elkins, Suxiang Tong

EPI_ISL_424897

EPI_ISL_424898

EPI_ISL_424899

EPI_ISL_424900

hCoV-19/USA/IA_6401/2020

hCoV-19/USA/IA_6399/2020

hCoV-19/USA/IA_6391/2020

hCoV-19/USA/IA_6390/2020

North America /

USA / Iowa

North America /

USA / Iowa

North America /

USA / Iowa

North America /

USA / Iowa

|  |  |  | Pathogen Discovery, |  |  |
| --- | --- | --- | --- | --- | --- |
|  | IA State |  | Respiratory Viruses | Ying Tao, Clinton R. Paden, Jing Zhang, Krista Queen, Anna |  |
|  |  |  | Branch, Division of |  |  |
| 2020-03-08 | Hygienic |  | Viral Diseases, | Uehara, Yan Li, Haibin Wang, Mary S. Keckler, Alison S. |  |
|  | Laboratory |  | Centers for Disease | Laufer Halpin, Christopher A. Elkins, Suxiang Tong |  |
|  |  |  | Control and |  |  |
|  |  |  | Prevention |  |  |
|  |  |  |  |  |  |
|  |  |  | Pathogen Discovery, |  |  |
|  |  |  | Respiratory Viruses |  |  |
| 2020-03-08 | IA State |  | Branch, Division of | Ying Tao, Clinton R. Paden, Jing Zhang, Krista Queen, Anna |  |
|  | Hygienic |  | Viral Diseases, | Uehara, Yan Li, Haibin Wang, Mary S. Keckler, Alison S. |  |
|  | Laboratory |  | Centers for Disease | Laufer Halpin, Christopher A. Elkins, Suxiang Tong |  |
|  |  |  | Control and |  |  |
|  |  |  | Prevention |  |  |
|  |  |  |  |  |  |
|  |  |  | Pathogen Discovery, |  |  |
|  | IA State |  | Respiratory Viruses | Ying Tao, Clinton R. Paden, Jing Zhang, Krista Queen, Anna |  |
|  |  |  | Branch, Division of |  |  |
| 2020-03-07 | Hygienic |  | Viral Diseases, | Uehara, Yan Li, Haibin Wang, Mary S. Keckler, Alison S. |  |
|  | Laboratory |  | Centers for Disease | Laufer Halpin, Christopher A. Elkins, Suxiang Tong |  |
|  |  |  | Control and |  |  |
|  |  |  | Prevention |  |  |
|  |  |  |  |  |  |
|  |  |  | Pathogen Discovery, |  |  |
|  |  |  | Respiratory Viruses |  |  |
| 2020-03-07 | IA State |  | Branch, Division of | Ying Tao, Clinton R. Paden, Jing Zhang, Krista Queen, Anna |  |
|  | Hygienic |  | Viral Diseases, | Uehara, Yan Li, Haibin Wang, Mary S. Keckler, Alison S. |  |
|  | Laboratory |  | Centers for Disease | Laufer Halpin, Christopher A. Elkins, Suxiang Tong |  |
|  |  |  | Control and |  |  |
|  |  |  | Prevention |  |  |
|  |  |  |  |  |  |

North America /

EPI_ISL_424901 hCoV-19/USA/NJ_3201/2020 USA / New 2020-03-04

Jersey

North America /

EPI_ISL_424902 hCoV-19/USA/SC_3520/2020 USA / South 2020-03-05

Carolina

North America /

EPI_ISL_424903 hCoV-19/USA/SC_3570/2020 USA / South 2020-03-07

Carolina

North America /

EPI_ISL_424904 hCoV-19/USA/SC_3571/2020 USA / South 2020-03-07

Carolina

NJ Public Health

and

Environmental

Laboratories

SC Dept of Health and Env. Control-Bureau of Laboratories

SC Dept of Health and Env. Control-Bureau of Laboratories

SC Dept of Health and Env. Control-Bureau of Laboratories

Pathogen Discovery, Respiratory Viruses Branch, Division of Viral Diseases, Centers for Disease Control and Prevention

Pathogen Discovery, Respiratory Viruses Branch, Division of Viral Diseases, Centers for Disease Control and Prevention

Pathogen Discovery, Respiratory Viruses Branch, Division of Viral Diseases, Centers for Disease Control and Prevention

Pathogen Discovery, Respiratory Viruses Branch, Division of Viral Diseases, Centers for Disease Control and Prevention

Ying Tao, Clinton R. Paden, Jing Zhang, Krista Queen, Anna Uehara, Yan Li, Haibin Wang, Mary S. Keckler, Alison S. Laufer Halpin, Christopher A. Elkins, Suxiang Tong

Ying Tao, Clinton R. Paden, Jing Zhang, Krista Queen, Anna Uehara, Yan Li, Haibin Wang, Mary S. Keckler, Alison S. Laufer Halpin, Christopher A. Elkins, Suxiang Tong

Ying Tao, Clinton R. Paden, Jing Zhang, Krista Queen, Anna Uehara, Yan Li, Haibin Wang, Mary S. Keckler, Alison S. Laufer Halpin, Christopher A. Elkins, Suxiang Tong

Ying Tao, Clinton R. Paden, Jing Zhang, Krista Queen, Anna Uehara, Yan Li, Haibin Wang, Mary S. Keckler, Alison S. Laufer Halpin, Christopher A. Elkins, Suxiang Tong

EPI_ISL_424905

EPI_ISL_424906

EPI_ISL_424907

EPI_ISL_424908

hCoV-19/USA/SC_3572/2020

hCoV-19/USA/NY_1922/2020

hCoV-19/USA/VA_6171/2020

hCoV-19/USA/MA_8932/2020

North America / USA / South Carolina

North America / USA / New York

North America / USA / Virginia

North America /

USA /

Massachusetts

2020-03-05

2020-03-04

2020-03-06

2020-03-05

SC Dept of Health and Env. Control-Bureau of Laboratories

NYC

Department of

Health and

Mental Hygiene

VA-Division of Consolidated Laboratory Services

MA State Public

Health

Laboratory

Pathogen Discovery, Respiratory Viruses Branch, Division of Viral Diseases, Centers for Disease Control and Prevention

Pathogen Discovery, Respiratory Viruses Branch, Division of Viral Diseases, Centers for Disease Control and Prevention

Pathogen Discovery, Respiratory Viruses Branch, Division of Viral Diseases, Centers for Disease Control and Prevention

Pathogen Discovery, Respiratory Viruses Branch, Division of Viral Diseases, Centers for Disease Control and Prevention

Ying Tao, Clinton R. Paden, Jing Zhang, Krista Queen, Anna Uehara, Yan Li, Haibin Wang, Mary S. Keckler, Alison S. Laufer Halpin, Christopher A. Elkins, Suxiang Tong

Ying Tao, Clinton R. Paden, Jing Zhang, Krista Queen, Anna Uehara, Yan Li, Haibin Wang, Mary S. Keckler, Alison S. Laufer Halpin, Christopher A. Elkins, Suxiang Tong

Ying Tao, Clinton R. Paden, Jing Zhang, Krista Queen, Anna Uehara, Yan Li, Haibin Wang, Mary S. Keckler, Alison S. Laufer Halpin, Christopher A. Elkins, Suxiang Tong

Ying Tao, Clinton R. Paden, Jing Zhang, Krista Queen, Anna Uehara, Yan Li, Haibin Wang, Mary S. Keckler, Alison S. Laufer Halpin, Christopher A. Elkins, Suxiang Tong

|  |  |  |  |  |  |  |  |  | Pathogen Discovery, |  |  |
| --- | --- | --- | --- | --- | --- | --- | --- | --- | --- | --- | --- |
|  |  |  |  | North America / |  |  | MA State Public |  | Respiratory Viruses | Ying Tao, Clinton R. Paden, Jing Zhang, Krista Queen, Anna |  |
|  |  |  |  |  |  |  |  |  | Branch, Division of |  |  |
| EPI_ISL_424909 |  | hCoV-19/USA/MA_8933/2020 |  | USA / | 2020-03-05 | | Health |  | Viral Diseases, | Uehara, Yan Li, Haibin Wang, Mary S. Keckler, Alison S. |  |
|  |  |  |  | Massachusetts |  |  | Laboratory |  | Centers for Disease | Laufer Halpin, Christopher A. Elkins, Suxiang Tong |  |
|  |  |  |  |  |  |  |  |  | Control and |  |  |
|  |  |  |  |  |  |  |  |  | Prevention |  |  |
|  |  |  |  |  |  |  |  |  |  |  |  |
|  |  |  |  |  |  |  |  |  | Pathogen Discovery, |  |  |
|  |  |  |  |  |  |  |  |  | Respiratory Viruses |  |  |
| EPI_ISL_424910 |  | hCoV-19/USA/MA_9703/2020 |  | North America / | 2020-03-05 | | MA State Public |  | Branch, Division of | Ying Tao, Clinton R. Paden, Jing Zhang, Krista Queen, Anna |  |
|  |  |  |  | USA / |  |  | Health |  | Viral Diseases, | Uehara, Yan Li, Haibin Wang, Mary S. Keckler, Alison S. |  |
|  |  |  |  | Massachusetts |  |  | Laboratory |  | Centers for Disease | Laufer Halpin, Christopher A. Elkins, Suxiang Tong |  |
|  |  |  |  |  |  |  |  |  | Control and |  |  |
|  |  |  |  |  |  |  |  |  | Prevention |  |  |
|  |  |  |  |  |  |  |  |  |  |  |  |
|  |  |  |  |  |  |  |  |  | Pathogen Discovery, |  |  |
|  |  |  |  | North America / |  |  | MA State Public |  | Respiratory Viruses | Ying Tao, Clinton R. Paden, Jing Zhang, Krista Queen, Anna |  |
|  |  |  |  |  |  |  |  |  | Branch, Division of |  |  |
| EPI_ISL_424911 |  | hCoV-19/USA/MA_9704/2020 |  | USA / | 2020-03-04 | | Health |  | Viral Diseases, | Uehara, Yan Li, Haibin Wang, Mary S. Keckler, Alison S. |  |
|  |  |  |  | Massachusetts |  |  | Laboratory |  | Centers for Disease | Laufer Halpin, Christopher A. Elkins, Suxiang Tong |  |
|  |  |  |  |  |  |  |  |  | Control and |  |  |
|  |  |  |  |  |  |  |  |  | Prevention |  |  |
|  |  |  |  |  |  |  |  |  |  |  |  |
|  |  |  |  |  |  |  |  |  | Pathogen Discovery, |  |  |
|  |  |  |  |  |  |  |  |  | Respiratory Viruses |  |  |
| EPI_ISL_424912 |  | hCoV-19/USA/MA_9889/2020 |  | North America / | 2020-03-04 | | MA State Public |  | Branch, Division of | Ying Tao, Clinton R. Paden, Jing Zhang, Krista Queen, Anna |  |
|  |  |  |  | USA / |  |  | Health |  | Viral Diseases, | Uehara, Yan Li, Haibin Wang, Mary S. Keckler, Alison S. |  |
|  |  |  |  | Massachusetts |  |  | Laboratory |  | Centers for Disease | Laufer Halpin, Christopher A. Elkins, Suxiang Tong |  |
|  |  |  |  |  |  |  |  |  | Control and |  |  |
|  |  |  |  |  |  |  |  |  | Prevention |  |  |
|  |  |  |  |  |  |  |  |  |  |  |  |

|  |  |  |  |  |  |  |  |  | Pathogen Discovery, |  |  |
| --- | --- | --- | --- | --- | --- | --- | --- | --- | --- | --- | --- |
|  |  |  |  | North America / |  |  | MA State Public |  | Respiratory Viruses | Ying Tao, Clinton R. Paden, Jing Zhang, Krista Queen, Anna |  |
|  |  |  |  |  |  |  |  |  | Branch, Division of |  |  |
| EPI_ISL_424913 |  | hCoV-19/USA/MA_1355/2020 |  | USA / | 2020-03-05 | | Health |  | Viral Diseases, | Uehara, Yan Li, Haibin Wang, Mary S. Keckler, Alison S. |  |
|  |  |  |  | Massachusetts |  |  | Laboratory |  | Centers for Disease | Laufer Halpin, Christopher A. Elkins, Suxiang Tong |  |
|  |  |  |  |  |  |  |  |  | Control and |  |  |
|  |  |  |  |  |  |  |  |  | Prevention |  |  |
|  |  |  |  |  |  |  |  |  |  |  |  |
|  |  |  |  |  |  |  |  |  | Pathogen Discovery, |  |  |
|  |  |  |  |  |  |  |  |  | Respiratory Viruses |  |  |
| EPI_ISL_424914 |  | hCoV-19/USA/MA_2652/2020 |  | North America / | 2020-03-06 | | MA State Public |  | Branch, Division of | Ying Tao, Clinton R. Paden, Jing Zhang, Krista Queen, Anna |  |
|  |  |  |  | USA / |  |  | Health |  | Viral Diseases, | Uehara, Yan Li, Haibin Wang, Mary S. Keckler, Alison S. |  |
|  |  |  |  | Massachusetts |  |  | Laboratory |  | Centers for Disease | Laufer Halpin, Christopher A. Elkins, Suxiang Tong |  |
|  |  |  |  |  |  |  |  |  | Control and |  |  |
|  |  |  |  |  |  |  |  |  | Prevention |  |  |
|  |  |  |  |  |  |  |  |  |  |  |  |
|  |  |  |  |  |  |  |  |  | Pathogen Discovery, |  |  |
|  |  |  |  | North America / |  |  | MA State Public |  | Respiratory Viruses | Ying Tao, Clinton R. Paden, Jing Zhang, Krista Queen, Anna |  |
|  |  |  |  |  |  |  |  |  | Branch, Division of |  |  |
| EPI_ISL_424915 |  | hCoV-19/USA/MA_3614/2020 |  | USA / | 2020-03-06 | | Health |  | Viral Diseases, | Uehara, Yan Li, Haibin Wang, Mary S. Keckler, Alison S. |  |
|  |  |  |  | Massachusetts |  |  | Laboratory |  | Centers for Disease | Laufer Halpin, Christopher A. Elkins, Suxiang Tong |  |
|  |  |  |  |  |  |  |  |  | Control and |  |  |
|  |  |  |  |  |  |  |  |  | Prevention |  |  |
|  |  |  |  |  |  |  |  |  |  |  |  |
|  |  |  |  |  |  |  |  |  | Pathogen Discovery, |  |  |
|  |  |  |  |  |  |  |  |  | Respiratory Viruses |  |  |
| EPI_ISL_424916 |  | hCoV-19/USA/MA_3616/2020 |  | North America / | 2020-03-06 | | MA State Public |  | Branch, Division of | Ying Tao, Clinton R. Paden, Jing Zhang, Krista Queen, Anna |  |
|  |  |  |  | USA / |  |  | Health |  | Viral Diseases, | Uehara, Yan Li, Haibin Wang, Mary S. Keckler, Alison S. |  |
|  |  |  |  | Massachusetts |  |  | Laboratory |  | Centers for Disease | Laufer Halpin, Christopher A. Elkins, Suxiang Tong |  |
|  |  |  |  |  |  |  |  |  | Control and |  |  |
|  |  |  |  |  |  |  |  |  | Prevention |  |  |
|  |  |  |  |  |  |  |  |  |  |  |  |

|  |  |  |  |  |  |  |  |  | Pathogen Discovery, |  |  |
| --- | --- | --- | --- | --- | --- | --- | --- | --- | --- | --- | --- |
|  |  |  |  | North America / |  |  | MA State Public |  | Respiratory Viruses | Ying Tao, Clinton R. Paden, Jing Zhang, Krista Queen, Anna |  |
|  |  |  |  |  |  |  |  |  | Branch, Division of |  |  |
| EPI_ISL_424917 |  | hCoV-19/USA/MA_3623/2020 |  | USA / | 2020-03-06 | | Health |  | Viral Diseases, | Uehara, Yan Li, Haibin Wang, Mary S. Keckler, Alison S. |  |
|  |  |  |  | Massachusetts |  |  | Laboratory |  | Centers for Disease | Laufer Halpin, Christopher A. Elkins, Suxiang Tong |  |
|  |  |  |  |  |  |  |  |  | Control and |  |  |
|  |  |  |  |  |  |  |  |  | Prevention |  |  |
|  |  |  |  |  |  |  |  |  |  |  |  |
|  |  |  |  |  |  |  |  |  | Pathogen Discovery, |  |  |
|  |  |  |  |  |  |  |  |  | Respiratory Viruses |  |  |
| EPI_ISL_424918 |  | hCoV-19/USA/MA_3626/2020 |  | North America / | 2020-03-06 | | MA State Public |  | Branch, Division of | Ying Tao, Clinton R. Paden, Jing Zhang, Krista Queen, Anna |  |
|  |  |  |  | USA / |  |  | Health |  | Viral Diseases, | Uehara, Yan Li, Haibin Wang, Mary S. Keckler, Alison S. |  |
|  |  |  |  | Massachusetts |  |  | Laboratory |  | Centers for Disease | Laufer Halpin, Christopher A. Elkins, Suxiang Tong |  |
|  |  |  |  |  |  |  |  |  | Control and |  |  |
|  |  |  |  |  |  |  |  |  | Prevention |  |  |
|  |  |  |  |  |  |  |  |  |  |  |  |
|  |  |  |  |  |  |  |  |  | Pathogen Discovery, |  |  |
|  |  |  |  | North America / |  |  | MA State Public |  | Respiratory Viruses | Ying Tao, Clinton R. Paden, Jing Zhang, Krista Queen, Anna |  |
|  |  |  |  |  |  |  |  |  | Branch, Division of |  |  |
| EPI_ISL_424919 |  | hCoV-19/USA/MA_3642/2020 |  | USA / | 2020-03-07 | | Health |  | Viral Diseases, | Uehara, Yan Li, Haibin Wang, Mary S. Keckler, Alison S. |  |
|  |  |  |  | Massachusetts |  |  | Laboratory |  | Centers for Disease | Laufer Halpin, Christopher A. Elkins, Suxiang Tong |  |
|  |  |  |  |  |  |  |  |  | Control and |  |  |
|  |  |  |  |  |  |  |  |  | Prevention |  |  |
|  |  |  |  |  |  |  |  |  |  |  |  |
|  |  |  |  |  |  |  |  |  | Pathogen Discovery, |  |  |
|  |  |  |  |  |  |  |  |  | Respiratory Viruses |  |  |
| EPI_ISL_424920 |  | hCoV-19/USA/MA_3653/2020 |  | North America / | 2020-03-06 | | MA State Public |  | Branch, Division of | Ying Tao, Clinton R. Paden, Jing Zhang, Krista Queen, Anna |  |
|  |  |  |  | USA / |  |  | Health |  | Viral Diseases, | Uehara, Yan Li, Haibin Wang, Mary S. Keckler, Alison S. |  |
|  |  |  |  | Massachusetts |  |  | Laboratory |  | Centers for Disease | Laufer Halpin, Christopher A. Elkins, Suxiang Tong |  |
|  |  |  |  |  |  |  |  |  | Control and |  |  |
|  |  |  |  |  |  |  |  |  | Prevention |  |  |
|  |  |  |  |  |  |  |  |  |  |  |  |

EPI_ISL_425647

EPI_ISL_425653

EPI_ISL_425654

hCoV-

19/Scotland/CVR100/2020

hCoV-

19/Scotland/CVR107/2020

hCoV-

19/Scotland/CVR108/2020

|  |  | West of |  |  |
| --- | --- | --- | --- | --- |
|  |  | Scotland |  |  |
|  |  | Specialist |  |  |
| Europe / United | 2020-03-14 | Virology Centre, | COVID-19 Genomics |  |
| Kingdom / |  | NHSGGC / | UK (COG-UK) |  |
| Scotland |  | MRC-University | Consortium |  |
|  |  | of Glasgow |  |  |

Centre for Virus

Research

|  |  | West of |  |
| --- | --- | --- | --- |
|  |  | Scotland |  |
|  |  | Specialist |  |
| Europe / United |  | Virology Centre, | COVID-19 Genomics |
| Kingdom / | 2020-03-13 | NHSGGC / | UK (COG-UK) |
| Scotland |  | MRC-University | Consortium |
|  |  | of Glasgow |  |

Centre for Virus

Research

|  |  | West of |  |  |
| --- | --- | --- | --- | --- |
|  |  | Scotland |  |  |
|  |  | Specialist |  |  |
| Europe / United | 2020-03-13 | Virology Centre, | COVID-19 Genomics |  |
| Kingdom / |  | NHSGGC / | UK (COG-UK) |  |
| Scotland |  | MRC-University | Consortium |  |
|  |  | of Glasgow |  |  |

Centre for Virus

Research

Ana da Silva Filipe, Kathy Smollett, Stephen Carmichael, Natasha Johnson, Daniel Mair, Lily Tong, Jenna Nichols; Sarah McDonald; Richard Orton, Joseph Hughes, Sreenu Vattipally, David L Robertson; Kathy Li, Natasha Jesudason, Rajiv Shah, James Shepherd, Antonia Ho, Emma Thomson; Alasdair MacLean, Rory Gunson.

Ana da Silva Filipe, Kathy Smollett, Stephen Carmichael, Natasha Johnson, Daniel Mair, Lily Tong, Jenna Nichols; Sarah McDonald; Richard Orton, Joseph Hughes, Sreenu Vattipally, David L Robertson; Kathy Li, Natasha Jesudason, Rajiv Shah, James Shepherd, Antonia Ho, Emma Thomson; Alasdair MacLean, Rory Gunson.

Ana da Silva Filipe, Kathy Smollett, Stephen Carmichael, Natasha Johnson, Daniel Mair, Lily Tong, Jenna Nichols; Sarah McDonald; Richard Orton, Joseph Hughes, Sreenu Vattipally, David L Robertson; Kathy Li, Natasha Jesudason, Rajiv Shah, James Shepherd, Antonia Ho, Emma Thomson; Alasdair MacLean, Rory Gunson.

EPI_ISL_404227

EPI_ISL_404228

EPI_ISL_404253

EPI_ISL_405839

EPI_ISL_406030

EPI_ISL_406031

hCoV-19/Zhejiang/WZ-

01/2020

hCoV-19/Zhejiang/WZ-

02/2020

hCoV-19/USA/IL1/2020

hCoV-19/Shenzhen/HKU-SZ-005/2020

hCoV-19/Shenzhen/HKU-SZ-002/2020

hCoV-19/Taiwan/2/2020

Asia / China /

Zhejiang

Asia / China /

Zhejiang

North America / USA / Illinois / Chicago

Asia / China / Guangdong / Shenzhen

Asia / China / Guangdong / Shenzhen

Asia / Taiwan /

Kaohsiung

2020-01-16

2020-01-17

2020-01-21

2020-01-11

2020-01-10

2020-01-23

Zhejiang

Provincial Center for Disease Control and Prevention

Zhejiang

Provincial Center for Disease Control and Prevention

IL Department of Public Health Chicago Laboratory

The University of Hong Kong - Shenzhen Hospital

The University of Hong Kong - Shenzhen Hospital

Centers for

Disease Control, R.O.C. (Taiwan)

Department of Microbiology, Zhejiang Provincial Center for Disease Control and Prevention

Department of Microbiology, Zhejiang Provincial Center for Disease Control and Prevention

Pathogen Discovery, Respiratory Viruses Branch, Division of Viral Diseases, Centers for Dieases Control and Prevention

Li Ka Shing Faculty of Medicine, The University of Hong Kong

Li Ka Shing Faculty of Medicine, The University of Hong Kong

Centers for Disease Control, R.O.C. (Taiwan)

Yin Chen, Yanjun Zhang, Haiyan Mao, Junhang Pan, Xiuyu Lou, Yiyu Lu, Juying Yan, Hanping Zhu, Jian Gao, Yan Feng, Yi Sun, Hao Yan, Zhen Li, Yisheng Sun, Liming Gong, Qiong Ge, Wen Shi, Xinying Wang, Wenwu Yao, Zhangnv Yang, Fang Xu, Chen Chen, Enfu Chen, Zhen Wang, Zhiping Chen, Jianmin Jiang, Chonggao Hu

Yanjun Zhang, Yin Chen, Haiyan Mao, Junhang Pan, Xiuyu Lou, Yiyu Lu, Juying Yan, Hanping Zhu, Jian Gao, Yan Feng, Yi Sun, Hao Yan, Zhen Li, Yisheng Sun, Liming Gong, Qiong Ge, Wen Shi, Xinying Wang, Wenwu Yao, Zhangnv Yang, Fang Xu, Chen Chen, Enfu Chen, Zhen Wang, Zhiping Chen, Jianmin Jiang, Chonggao Hu

Ying Tao, Krista Queen, Clinton R. Paden, Jing Zhang, Yan Li, Anna Uehara, Xiaoyan Lu, Brian Lynch, Senthil Kumar K.

Sakthivel, Brett L. Whitaker, Shifaq Kamili, Lijuan Wang, Janna' R. Murray, Susan I. Gerber, Stephen Lindstrom, Suxiang Tong

Chan,J.F.-W., Yuan,S., Kok,K.H., To,K.K.-W., Chu,H., Yang,J., Xing,F., Liu,J., Yip,C.C.-Y., Poon,R.W.-S., Tsai,H.W., Lo,S.K.-F., Chan,K.H., Poon,V.K.-M., Chan,W.M., Ip,J.D., Cai,J.P., Cheng,V.C.-C., Chen,H., Hui,C.K.-M. and Yuen,K.Y.

Chan,J.F.-W., Yuan,S., Kok,K.H., To,K.K.-W., Chu,H., Yang,J., Xing,F., Liu,J., Yip,C.C.-Y., Poon,R.W.-S., Tsai,H.W., Lo,S.K.-F., Chan,K.H., Poon,V.K.-M., Chan,W.M., Ip,J.D., Cai,J.P., Cheng,V.C.-C., Chen,H., Hui,C.K.-M. and Yuen,K.Y.

Ji-Rong Yang, Yu-Chi Lin, Jung-Jung Mu, Ming-Tsan Liu,

Shu-Ying Li

|  |  |  |  |  |  |  |  |  | Pathogen Discovery, |  |  |
| --- | --- | --- | --- | --- | --- | --- | --- | --- | --- | --- | --- |
|  |  |  |  | North America / |  |  | California |  | Respiratory Viruses | Anna Uehara, Krista Queen, Ying Tao, Yan Li, Clinton R. |  |
|  |  |  |  |  |  |  |  |  | Branch, Division of | Paden, Jing Zhang, Xiaoyan Lu, Brian Lynch, Senthil Kumar |  |
| EPI_ISL_406034 |  | hCoV-19/USA/CA1/2020 |  | USA / California | 2020-01-23 | | Department of |  | Viral Diseases, | K. Sakthivel, Brett L. Whitaker, Shifaq Kamili, Lijuan Wang, |  |
|  |  |  |  | / Los Angeles |  |  | Public Health |  | Centers for Dieases | Janna' R. Murray, Susan I. Gerber, Stephen Lindstrom, |  |
|  |  |  |  |  |  |  |  |  | Control and | Suxiang Tong |  |
|  |  |  |  |  |  |  |  |  | Prevention |  |  |
|  |  |  |  |  |  |  |  |  |  |  |  |
|  |  |  |  |  |  |  |  |  | Pathogen Discovery, | Anna Uehara, Krista Queen, Ying Tao, Yan Li, Clinton R. |  |
|  |  |  |  |  |  |  |  |  | Respiratory Viruses |  |  |
| EPI_ISL_406036 |  | hCoV-19/USA/CA2/2020 |  | North America / | 2020-01-22 | | California |  | Branch, Division of | Paden, Jing Zhang, Xiaoyan Lu, Brian Lynch, Senthil Kumar |  |
|  |  |  |  | USA / California |  |  | Department of |  | Viral Diseases, | K. Sakthivel, Brett L. Whitaker, Shifaq Kamili, Lijuan Wang, |  |
|  |  |  |  | / Orange County |  |  | Public Health |  | Centers for Dieases | Janna' R. Murray, Susan I. Gerber, Stephen Lindstrom, |  |
|  |  |  |  |  |  |  |  |  | Control and | Suxiang Tong |  |
|  |  |  |  |  |  |  |  |  | Prevention |  |  |
|  |  |  |  |  |  |  |  |  |  |  |  |
|  |  |  |  |  |  |  |  |  | BGI & Institute of |  |  |
|  |  |  |  |  |  |  |  |  | Microbiology, |  |  |
|  |  |  |  |  |  |  |  |  | Chinese Academy of |  |  |
|  |  |  |  |  |  |  | General Hospital |  | Sciences & |  |  |
|  |  |  |  |  |  |  | of Central |  | Shandong First |  |  |
| EPI_ISL_406800 |  | hCoV-19/Wuhan/WH03/2020 |  | Asia / China / | 2020-01-01 | | Theater |  | Medical University & | Weijun Chen, Yuhai Bi, Weifeng Shi and Zhenhong Hu |  |
|  |  |  |  |  |  |  | Command of |  | Shandong Academy |  |  |
|  |  |  |  | Hubei / Wuhan |  |  |  |  |  |  |  |
|  |  |  |  |  |  |  | People's |  | of Medical Sciences |  |  |
|  |  |  |  |  |  |  |  |  |  |  |  |
|  |  |  |  |  |  |  | Liberation Army |  | & General Hospital |  |  |
|  |  |  |  |  |  |  | of China |  | of Central Theater |  |  |
|  |  |  |  |  |  |  |  |  | Command of |  |  |

People's Liberation

Army of China

|  |  |  |  |  |  |  |  |  | BGI & Institute of |  |  |
| --- | --- | --- | --- | --- | --- | --- | --- | --- | --- | --- | --- |
|  |  |  |  |  |  |  |  |  | Microbiology, |  |  |
|  |  |  |  |  |  |  |  |  | Chinese Academy of |  |  |
|  |  |  |  |  |  |  | General Hospital |  | Sciences & |  |  |
|  |  |  |  |  |  |  | of Central |  | Shandong First |  |  |
| EPI_ISL_406801 |  | hCoV-19/Wuhan/WH04/2020 |  | Asia / China / | 2020-01-05 | | Theater |  | Medical University & | Weijun Chen, Yuhai Bi, Weifeng Shi and Zhenhong Hu |  |
|  |  |  |  |  |  |  | Command of |  | Shandong Academy |  |  |
|  |  |  |  | Hubei / Wuhan |  |  |  |  |  |  |  |
|  |  |  |  |  |  |  | People's |  | of Medical Sciences |  |  |
|  |  |  |  |  |  |  |  |  |  |  |  |
|  |  |  |  |  |  |  | Liberation Army |  | & General Hospital |  |  |
|  |  |  |  |  |  |  | of China |  | of Central Theater |  |  |
|  |  |  |  |  |  |  |  |  | Command of |  |  |
|  |  |  |  |  |  |  |  |  | People's Liberation |  |  |
|  |  |  |  |  |  |  |  |  | Army of China |  |  |
|  |  |  |  |  |  |  |  |  |  |  |  |
|  |  |  |  |  |  |  | Charité |  |  |  |  |
|  |  |  |  |  |  |  | Universitätsmedi |  |  |  |  |
|  |  |  |  | Europe / |  |  | zin Berlin, |  | Charité |  |  |
|  |  |  |  |  |  |  | Institute of |  |  | Victor M Corman, Julia Schneider, Talitha Veith, Barbara |  |
| EPI_ISL_406862 |  | hCoV- |  | Germany / | 2020-01-28 | |  |  | Universitätsmedizin |  |  |
|  |  |  |  |  |  |  | Virology; Institut |  |  | Mühlemann, Markus Antwerpen, Christian Drosten, Roman |  |
|  |  | 19/Germany/BavPat1/2020 |  | Bavaria / |  |  |  |  | Berlin, Institute of |  |  |
|  |  |  |  |  |  |  | für Mikrobiologie |  |  | Wölfel |  |
|  |  |  |  | Munich |  |  |  |  | Virology |  |  |
|  |  |  |  |  |  |  | der |  |  |  |  |
|  |  |  |  |  |  |  |  |  |  |  |  |
|  |  |  |  |  |  |  | Bundeswehr, |  |  |  |  |
|  |  |  |  |  |  |  | Munich |  |  |  |  |
|  |  |  |  |  |  |  |  |  |  |  |  |
|  |  |  |  |  |  |  | Hangzhou |  |  |  |  |
| EPI_ISL_406970 |  | hCoV-19/Hangzhou/HZ-1/2020 |  | Asia / China / | 2020-01-20 | | Center for |  | Hangzhou Center for | Yu Hua, Wang Haoqiu, Li Jun, Yu Xinfeng |  |
|  |  |  |  | Zhejiang / |  |  | Disease and |  | Disease and Control |  |  |
|  |  |  |  | Hangzhou |  |  | Control |  | Microbiology Lab |  |  |
|  |  |  |  |  |  |  | Microbiology Lab |  |  |  |  |
|  |  |  |  |  |  |  |  |  |  |  |  |
| EPI_ISL_406973 |  | hCoV-19/Singapore/1/2020 |  | Asia / Singapore | 2020-01-23 | | Singapore |  | National Public | Mak, TM; Octavia S; Chavatte JM; Zhou, ZY; Cui, L; Lin, RTP |  |
|  |  |  |  |  |  |  | General Hospital |  | Health Laboratory |  |  |
|  |  |  |  |  |  |  |  |  |  |  |  |
|  |  |  |  |  |  |  |  |  |  |  |  |
|  |  | hCoV- |  | Asia / China / |  |  | Hangzhou |  | Hangzhou Center for | Jun Li, Haoqiu Wang, Hua Yu, Lingfeng Mao, Xinfen Yu, |  |
|  |  |  |  |  |  |  | Center for |  |  |  |  |
| EPI_ISL_407313 |  | 19/Hangzhou/HZCDC0001/20 |  | Zhejiang / | 2020-01-19 | |  |  | Disease Control and | Zhou Sun, Qingxin Kong, Xin Qian, Shuchang Chen, Xuchu |  |
|  |  |  |  |  |  |  | Disease Control |  |  |  |  |
|  | 20 | |  | Hangzhou |  |  |  |  | Prevention | Wang |  |
|  |  |  |  |  |  |  | and Prevention |  |  |  |  |
|  |  |  |  |  |  |  |  |  |  |  |  |
|  |  |  |  |  |  |  |  |  |  |  |  |

EPI_ISL_408665

EPI_ISL_408666

EPI_ISL_408667

EPI_ISL_408669

EPI_ISL_408976

hCoV-19/Japan/TY-WK-

012/2020

hCoV-19/Japan/TY-WK-

501/2020

hCoV-19/Japan/TY-WK-

521/2020

hCoV-19/Japan/KY-V-

029/2020

hCoV-

19/Australia/NSW02/2020

Asia / Japan /

Tokyo

Asia / Japan /

Tokyo

Asia / Japan /

Tokyo

Asia / Japan /

Kyoto

Oceania / Australia / New South Wales / Sydney

2020-01-29

2020-01-31

2020-01-31

2020-01-29

2020-01-22

Dept. of Virology III, National Institute of Infectious Diseases

Dept. of Virology III, National Institute of Infectious Diseases

Dept. of Virology III, National Institute of Infectious Diseases

Dept. of Virology III, National Institute of Infectious Diseases

Centre for Infectious Diseases and Microbiology Laboratory Services

Pathogen Genomics Center, National Institute of Infectious Diseases

Pathogen Genomics Center, National Institute of Infectious Diseases

Pathogen Genomics Center, National Institute of Infectious Diseases

Pathogen Genomics Center, National Institute of Infectious Diseases

NSW Health

Pathology - Institute

of Clinical Pathology

and Medical

Research;

Westmead Hospital;

University of Sydney

Tsuyoshi Sekizuka, Shutoku Matsuyama, Naganori Nao, Kazuya Shirato, Makoto Takeda, Makoto Kuroda

Tsuyoshi Sekizuka, Shutoku Matsuyama, Naganori Nao, Kazuya Shirato, Makoto Takeda, Makoto Kuroda

Tsuyoshi Sekizuka, Shutoku Matsuyama, Naganori Nao, Kazuya Shirato, Makoto Takeda, Makoto Kuroda

Tsuyoshi Sekizuka, Shutoku Matsuyama, Naganori Nao, Kazuya Shirato, Makoto Takeda, Makoto Kuroda

Rockett R, Sadsad R, Eden J-S, Carter I, Rahman H, Holmes EC, O’Sullivan MV, Sintchenko V, Chen SC, Maddocks S, Kok J and Dwyer DE for the 2019-nCoV Study Group*

EPI_ISL_408977

EPI_ISL_409067

EPI_ISL_410045

EPI_ISL_412116

hCoV-

19/Australia/NSW03/2020

hCoV-19/USA/MA1/2020

hCoV-19/USA/IL2/2020

hCoV-19/England/09c/2020

Oceania / Australia / New South Wales / Sydney

North America /

USA /

Massachusetts

North America / USA / Illinois

Europe / United Kingdom / England

2020-01-25

2020-01-29

2020-01-28

2020-02-09

Serology,

Virology and

OTDS

Laboratories

(SAViD), NSW

Health

Pathology

Randwick

Massachusetts

Department of

Public Health

IL Department of Public Health Chicago Laboratory

Respiratory Virus Unit, Microbiology Services Colindale, Public Health England

NSW Health

Pathology - Institute of Clinical Pathology and Medical Research; Centre for Infectious Diseases and Microbiology Laboratory Services; Westmead Hospital; University of Sydney

Pathogen Discovery, Respiratory Viruses Branch, Division of Viral Diseases, Centers for Dieases Control and Prevention

Pathogen Discovery, Respiratory Viruses Branch, Division of Viral Diseases, Centers for Dieases Control and Prevention

Respiratory Virus Unit, Microbiology Services Colindale, Public Health England

Eden J-S, Carter I, Rahman H, Rawlinson W, Holmes EC, Rockett R, O’Sullivan MV, Sintchenko V, Chen SC, Maddocks S, Kok J and Dwyer DE for the 2019-nCoV Study Group*

Clinton R. Paden, Jing Zhang, Krista Queen, Yan Li, Ying Tao, Anna Uehara, Xiaoyan Lu, Brian Lynch, Senthil Kumar K. Sakthivel, Brett L. Whitaker, Shifaq Kamili, Lijuan Wang, Janna' R. Murray, Susan I. Gerber, Stephen Lindstrom, Suxiang Tong

Yan Li, Jing Zhang, Krista Queen, Ying Tao, Anna Uehara, Clinton R. Paden, Xiaoyan Lu, Brian Lynch, Senthil Kumar K. Sakthivel, Brett L. Whitaker, Shifaq Kamili, Lijuan Wang, Janna' R. Murray, Susan I. Gerber, Stephen Lindstrom, Suxiang Tong

Monica Galiano, Shahjahan Miah, Angie Lackenby, Omolola Akinbami, Tiina Talts, Leena Bhaw, Richard Myers, Steven Platt, Kirstin Edwards, Jonathan Hubb, Joanna Ellis, Maria Zambon

EPI_ISL_412862

EPI_ISL_413213

EPI_ISL_413214

EPI_ISL_413455

EPI_ISL_413456

EPI_ISL_413457

EPI_ISL_413459

hCoV-19/USA/CA9/2020

hCoV-

19/Australia/NSW06/2020

hCoV-

19/Australia/NSW07/2020

hCoV-19/USA/WA4-

UW2/2020

hCoV-19/USA/WA-S2/2020

hCoV-19/USA/WA6-

UW3/2020

hCoV-19/Japan/TK-20-31-

3/2020

North America /

USA / California

/ Solano

Oceania / Australia / New South Wales / Sydney

Oceania / Australia / New South Wales / Sydney

North America /

USA /

Washington

North America /

USA /

Washington /

King County

North America /

USA /

Washington

Asia / Japan /

Tokyo

2020-02-23

2020-02-29

2020-02-29

2020-02-28

2020-02-20

2020-02-29

2020-02-20

|  |  | Pathogen Discovery, |  |  |
| --- | --- | --- | --- | --- |
| California |  | Respiratory Viruses | Krista Queen, Anna Uehara, Jing Zhang, Yan Li, Ying Tao, |  |
|  |  | Branch, Division of | Clinton R. Paden, Haibin Wang, Shifaq Kamili, Xiaoyan Lu, |  |
| Department of |  | Viral Diseases, | Brian Lynch, Senthil Kumar K. Sakthivel, Brett L. Whitaker, |  |
| Public Health |  | Centers for Disease | Lijuan Wang, Janna' R. Murray, Jasmine Padilla, Justin Lee, |  |
|  |  | Control and | Susan I. Gerber, Stephen Lindstrom, Suxiang Tong |  |
|  |  | Prevention |  |  |
|  |  |  |  |  |
| Centre for |  | NSW Health |  |  |
|  |  | Pathology - Institute |  |  |
| Infectious |  |  |  |  |
|  |  | of Clinical Pathology | Eden J-S, Carter I, Rahman H, Holmes EC, Rockett R, |  |
| Diseases and |  |  |  |  |
|  |  | and Medical | O’Sullivan MV, Sintchenko V, Chen SC, Maddocks S, Kok J |  |
| Microbiology |  |  |  |  |
|  |  | Research; | and Dwyer DE for the 2019-nCoV Study Group* |  |
| Laboratory |  |  |  |  |
|  |  | Westmead Hospital; |  |  |
| Services |  |  |  |  |
|  |  | University of Sydney |  |  |
|  |  |  |  |  |
|  |  |  |  |  |
| Centre for |  | NSW Health |  |  |
|  |  | Pathology - Institute |  |  |
| Infectious |  |  | Eden J-S, Carter I, Rahman H, Holmes EC, Rockett R, |  |
|  |  | of Clinical Pathology |  |  |
| Diseases and |  |  |  |  |
|  |  | and Medical | O’Sullivan MV, Sintchenko V, Chen SC, Maddocks S, Kok J |  |
| Microbiology |  |  |  |  |
|  |  | Research; | and Dwyer DE for the 2019-nCoV Study Group* |  |
| Laboratory |  |  |  |  |
|  |  | Westmead Hospital; |  |  |
| Services |  |  |  |  |
|  |  | University of Sydney |  |  |
|  |  |  |  |  |
|  |  |  |  |  |
| Washington |  | University of | Pavitra Roychoudhury, Arun Nalla, Hong Xie, Keith Jerome, |  |
| State Public |  | Washington Virology |  |  |
|  |  |  | Alexander Greninger |  |
| Health Lab |  | Lab |  |  |
|  |  |  |  |  |
| Seattle Flu Study |  | Seattle Flu Study | Chu et al |  |
|  |  |  |  |  |
| Washington |  |  | Pavitra Roychoudhury, Arun Nalla, Hong Xie, Keith Jerome, |  |
| State Public |  | UW Virology Lab |  |  |
|  |  |  | Alexander Greninger |  |
| Health Lab |  |  |  |  |
|  |  |  |  |  |
|  |  | Pathogen Genomics | Tsuyoshi Sekizuka, Kentaro Itokawa, Takuya Adachi, |  |
| Department of |  |  | Masahiro Sano, Jun Yamazaki, Ippei Miyamoto, Haruka |  |
|  |  | Center, National |  |  |
| Pathology, |  |  | Nishioka, Ja-Mun Chong, Noriko Nakajima, Yuko Sato, |  |
|  |  | Institute of Infectious |  |  |
| Toshima Hospital |  |  | Minoru Tobiume, Harutaka Katano, Tadaki Suzuki, Makoto |  |
|  |  | Diseases | Kuroda |  |
|  |  |  |  |  |
|  |  |  |  |  |

EPI_ISL_413488

EPI_ISL_413489

EPI_ISL_413996

EPI_ISL_413997

EPI_ISL_413999

EPI_ISL_414414

EPI_ISL_414423

hCoV-19/Germany/NRW-

01/2020

hCoV-19/Italy/UniSR1/2020

hCoV-

19/Switzerland/TI9486/2020

hCoV-

19/Switzerland/GE3895/2020

hCoV-

19/Switzerland/AG0361/2020

hCoV-

19/Australia/QLD09/2020

hCoV-

19/Netherlands/Gelderland_1/

2020

Europe / Germany / North Rhine Westphalia / Heinsberg District

Europe / Italy / Lombardy / Milan

Europe / Switzerland / Tessin

Europe / Switzerland / Geneva

Europe /

Switzerland /

Argovie

Oceania / Australia / Queensland / Gold Coast

Europe / Netherlands / Gelderland

2020-02-28

2020-03-03

2020-02-24

2020-02-26

2020-02-27

2020-02-29

2020-03-02

| Center of |  |  |  |  |
| --- | --- | --- | --- | --- |
| Medical |  | Center of Medical | Ortwin Adams, Marcel Andree, Alexander Dilthey, Torsten |  |
| Microbiology, |  | Microbiology, |  |  |
|  |  |  | Feldt, Sandra Hauka, Torsten Houwaart, Björn-Erik Jensen, |  |
| Virology, and |  | Virology, and |  |  |
|  |  |  | Detlef Kindgen-Milles, Malte Kohns Vasconcelos, Klaus |  |
| Hospital |  | Hospital Hygiene, |  |  |
|  |  |  | Pfeffer, Tina Senff, Daniel Strelow, Jörg Timm, Andreas |  |
| Hygiene, |  | University of |  |  |
|  |  |  | Walker, Tobias Wienemann |  |
| University of |  | Duesseldorf |  |  |
|  |  |  |  |  |
| Duesseldorf |  |  |  |  |
|  |  |  |  |  |
| Laboratorio di |  | Laboratorio di |  |  |
| Microbiologia e |  |  | R.A Diotti, E. Criscuolo, M. Castelli, V. Caputo, R. Ferrarese, |  |
|  |  | Microbiologia e |  |  |
| Virologia, |  |  |  |  |
|  |  | Virologia, Università | M. Sampaolo, E. Boeri, I. Negri, V. Amato, G. Lo Raso, C. Di |  |
| Università Vita- |  |  |  |  |
|  |  | Vita-Salute San | Resta, R. Burioni, M. Clementi, N. Mancini & N. Clementi |  |
| Salute San |  |  |  |  |
|  |  | Raffaele, Milano |  |  |
| Raffaele, Milano |  |  |  |  |
|  |  |  |  |  |
|  |  |  |  |  |
| Laboratoire de |  | Swiss National | LAUBSCHER Florian et al. |  |
|  |  | Reference Centre for |  |  |
| Virologie, HUG |  |  |  |  |
|  |  | Influenza |  |  |
|  |  |  |  |  |
| Laboratoire de |  | Swiss National | LAUBSCHER Florian et al. |  |
|  |  | Reference Centre for |  |  |
| Virologie, HUG |  |  |  |  |
|  |  | Influenza |  |  |
|  |  |  |  |  |
| Laboratoire de |  | Swiss National | LAUBSCHER Florian et al. |  |
|  |  | Reference Centre for |  |  |
| Virologie, HUG |  |  |  |  |
|  |  | Influenza |  |  |
|  |  |  |  |  |
|  |  |  | Bixing Huang, Alyssa Pyke, Amanda De Jong, Andrew Van |  |
|  |  |  | Den Hurk, Carmel Taylor, David Warrilow, Doris Genge, |  |
| Pathology |  | Public Health | Elisabeth Gamez, Glen Hewitson, Ian Maxwell Mackay, Inga |  |
|  |  |  | Sultana, Jamie McMahon, Jean Barcelon, Judy Northill, |  |
| Queensland |  | Virology Laboratory |  |  |
|  |  |  | Mitchell Finger, Natalie Simpson, Neelima Nair, Peter |  |
|  |  |  |  |  |
|  |  |  | Burtonclay, Peter Moore, Sarah Wheatley, Sean Moody, |  |
|  |  |  | Sonja Hall-Mendelin, Timothy Gardam, and Frederick Moore |  |
|  |  |  |  |  |
|  |  |  | David Nieuwenhuijse, Bas Oude Munnink, Reina Sikkema, |  |
|  |  |  | Claudia Schapendonk, Irina Chestakova, Anne van der |  |
| Dutch COVID- |  | Erasmus Medical | Linden, Mark Pronk, Pascal Lexmond, Corien Swaan, Manon |  |
|  |  |  | Haverkate, Madelief Mollers, Mart Stein, Sandra Kengne |  |
| 19 response |  |  |  |  |
|  |  | Center | Kamga Mobou, Jeroen van Kampen, Jolanda Voermans, |  |
| team |  |  |  |  |
|  |  |  | Aura Timen, Corine GeurtsvanKessel, Annemiek van der |  |
|  |  |  |  |  |
|  |  |  | Eijk, Richard Molenkamp, Marion Koopmans, on behalf of the |  |
|  |  |  | Dutch national COVID-19 response team. |  |
|  |  |  |  |  |

|  | hCoV- | Europe / |  | Dutch COVID- |
| --- | --- | --- | --- | --- |
| EPI_ISL_414424 | 19/Netherlands/Limburg_2/202 | Netherlands / | 2020-03-03 | 19 response |
|  | 0 | Limburg |  | team |

| EPI_ISL_414425 | hCoV- | Europe / | 2020-03-03 | Dutch COVID- |  |
| --- | --- | --- | --- | --- | --- |
|  | 19/Netherlands/Limburg_3/202 | Netherlands / |  | 19 response |  |
|  | 0 | Limburg |  | team |  |

|  | hCoV- | Europe / |  | Dutch COVID- |
| --- | --- | --- | --- | --- |
| EPI_ISL_414426 | 19/Netherlands/Limburg_4/202 | Netherlands / | 2020-03-03 | 19 response |
|  | 0 | Limburg |  | team |

| EPI_ISL_414428 |  | hCoV- |  | Europe / | 2020-03-02 | | Dutch COVID- |  |
| --- | --- | --- | --- | --- | --- | --- | --- | --- |
|  |  | 19/Netherlands/NoordBrabant |  | Netherlands / |  |  | 19 response |  |
|  | _1/2020 | |  | Noord Brabant |  |  | team |  |
|  |  |  |  |  |  |  |  |  |

Erasmus Medical

Center

Erasmus Medical

Center

Erasmus Medical

Center

Erasmus Medical

Center

David Nieuwenhuijse, Bas Oude Munnink, Reina Sikkema, Claudia Schapendonk, Irina Chestakova, Anne van der Linden, Mark Pronk, Pascal Lexmond, Corien Swaan, Manon Haverkate, Madelief Mollers, Mart Stein, Sandra Kengne Kamga Mobou, Jeroen van Kampen, Jolanda Voermans, Aura Timen, Corine GeurtsvanKessel, Annemiek van der Eijk, Richard Molenkamp, Marion Koopmans, on behalf of the Dutch national COVID-19 response team.

David Nieuwenhuijse, Bas Oude Munnink, Reina Sikkema, Claudia Schapendonk, Irina Chestakova, Anne van der Linden, Mark Pronk, Pascal Lexmond, Corien Swaan, Manon Haverkate, Madelief Mollers, Mart Stein, Sandra Kengne Kamga Mobou, Jeroen van Kampen, Jolanda Voermans, Aura Timen, Corine GeurtsvanKessel, Annemiek van der Eijk, Richard Molenkamp, Marion Koopmans, on behalf of the Dutch national COVID-19 response team.

David Nieuwenhuijse, Bas Oude Munnink, Reina Sikkema, Claudia Schapendonk, Irina Chestakova, Anne van der Linden, Mark Pronk, Pascal Lexmond, Corien Swaan, Manon Haverkate, Madelief Mollers, Mart Stein, Sandra Kengne Kamga Mobou, Jeroen van Kampen, Jolanda Voermans, Aura Timen, Corine GeurtsvanKessel, Annemiek van der Eijk, Richard Molenkamp, Marion Koopmans, on behalf of the Dutch national COVID-19 response team.

David Nieuwenhuijse, Bas Oude Munnink, Reina Sikkema, Claudia Schapendonk, Irina Chestakova, Anne van der Linden, Mark Pronk, Pascal Lexmond, Corien Swaan, Manon Haverkate, Madelief Mollers, Mart Stein, Sandra Kengne Kamga Mobou, Jeroen van Kampen, Jolanda Voermans, Aura Timen, Corine GeurtsvanKessel, Annemiek van der Eijk, Richard Molenkamp, Marion Koopmans, on behalf of the Dutch national COVID-19 response team.

|  | hCoV- | Europe / |  | Dutch COVID- |
| --- | --- | --- | --- | --- |
| EPI_ISL_414429 | 19/Netherlands/NoordBrabant | Netherlands / | 2020-03-02 | 19 response |
|  | _3/2020 | Noord Brabant |  | team |

| EPI_ISL_414433 | hCoV- | Europe / | 2020-03-03 | Dutch COVID- |  |
[truncated: 1,098,525 more chars]
