# Supplementary material for: The ongoing COVID-19 epidemic in Minas Gerais, Brazil: insights from epidemiological data and SARS-CoV-2 whole genome sequencing
Source: Emerg Microbes Infect. 2020 Aug 11;9(1):1824–34. doi: 10.1080/22221751.2020.1803146 (PMC7473129; doi:10.1080/22221751.2020.1803146)
Supplement: Supplementary_Material.docx [file TEMI_A_1803146_SM3075.docx]

**Supplementary Material**

## Projection of number of infections and observation rate from assumed infection fatality ratio

### Methods

Our data included reported cases and deaths for 3 Brazilian states: São Paulo (SP), Minas Gerais (MG) and Rio de Janeiro (RJ). From the mortality time series (MTS), we projected the local number of infections making two main simplifying assumptions: that the infection fatality ratio of SARS-CoV-2 would be similar in Brazil to that reported elsewhere, and that the number of deaths is well reported.

The infection fatality ratio (IFR) is calculated by the ratio of number of deaths and number of infections, in which the latter is generally unknown and likely to be a few to several times higher than reported cases. The IFR we consider is the one reported by Verity and colleagues (mean 0.66%, CI 95% 0.39-1.33%, [[1]](https://paperpile.com/c/V1tw35/o7Mh)) for its general use in modelling aimed at informing interventions, e.g. [[2]](https://paperpile.com/c/V1tw35/E89g). For each Brazilian state, using the mean and 95%CI of the IFR, we obtain a projected total number of cases in time $I\left( t \right)=D\left( t \right)/\left( {IFR}/{100} \right)$, where D(t) is the cumulative number of deaths. With a projection of the number of infections in time, we obtain the likely observation rate of cases from $\theta\left( t \right)=c\left( t \right)/I\left( t \right)$ where c(t) is the number of reported cases in time. We also looked at the case fatality ratio (CFR) in time, defined as the ratio between the reported deaths and reported cases.

### Results

The 3 Brazilian states presented the same general behaviour in terms of projected total number of infections and reported cases: the difference between the number of projected total infections (informed by the MTS) and the number of reported cases increased in time (Figure S1, top). As such, the projected observation rates declined with time (Figure S1, bottom), and all states appeared to converge to similar observation rates with time. By the last time point analysed, RJ and SP had similar observation rates at 7.6% (4.49-15.3) for RJ and 7.74% (4.57-15.6) for SP. MG, for which the epidemic has started later in time, the observation rate was 15.3% (9.05-30.8).

| 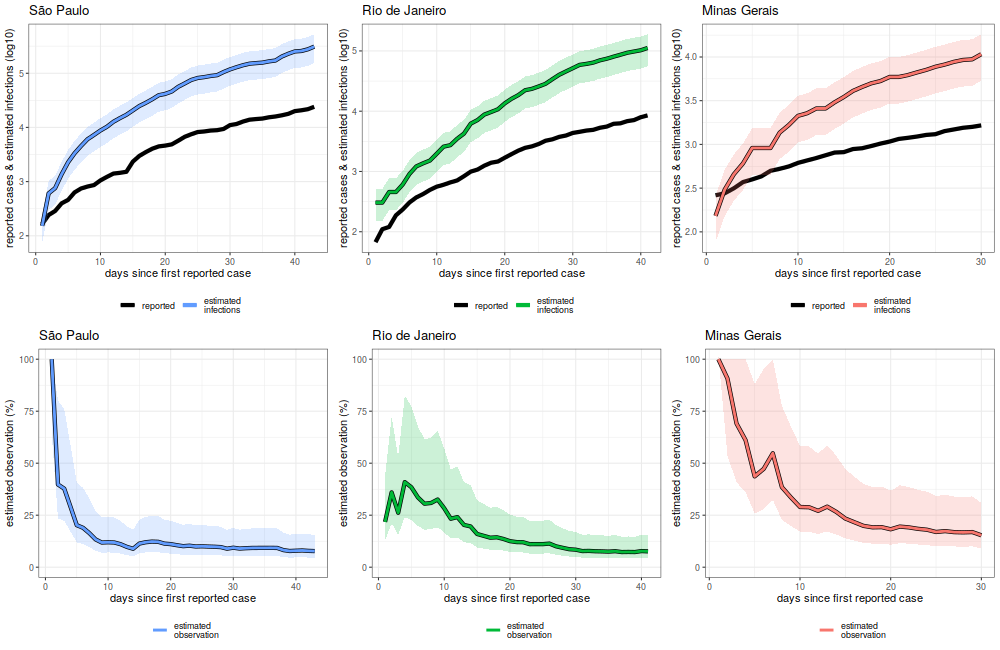 |
| --- |
| **Figure S1.** (top) Reported cumulative cases (black) and projected number of infections (colors) per state. (bottom) Projected observation rate per state. SP = São Paulo, MG = Minas Gerais, RJ = Rio de Janeiro. |

We next looked at the CFR within the states (Figure S2). Following the trend in decreasing observation rates, the CRF increased with time. RJ and SP presented similar CRF at the end of the time series data (which had similar lengths), while MG consistently presented lower CRF than the other two states. The CRFs for the entire period were: 2.67% (0.63-4.04) for MG, 5.39% (1.71-9.0) for RJ and 6.0% (1.66-8.4) for SP. For SP and RJ, these were consistently higher than reported elsewhere, e.g.: 2.6% (95% CI 0.89-6.7) for the Diamond Princess cruise ship [[3]](https://paperpile.com/c/V1tw35/yBWi), and 3.67% (95% CI 3.56-3.80) and 1.2% (95% CI 0.3-2.7) and 1.4% (95% CI 0.9-2.1) for Wuhan (China) [[1,3,4]](https://paperpile.com/c/V1tw35/WZfT+yBWi+o7Mh).

| 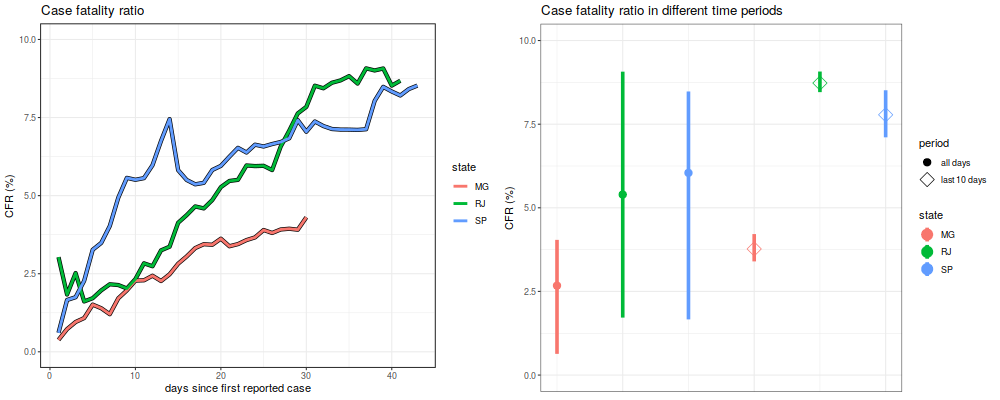 |
| --- |
| **Figure S2.** (A, left) Case fatality ratio in time for each state. (B, right) Case fatality ratio (mean, 95% range) for entire period of data (full circles) and for the last 10 days of each time series (open diamonds). SP = São Paulo, MG = Minas Gerais, RJ = Rio de Janeiro. |

## Estimation of reproduction numbers

The reproduction number R (basic R0, effective Re) of a pathogen can be estimated using mechanistic or statistical models applied to time series of confirmed cases. At early stages of an outbreak, methods aim at fitting the epidemic growth rate r, from which estimates of doubling times, R0 and Re can be obtained under certain assumptions.

For SARS-CoV-2 the confirmed (cases) time series (CTS) are subject to many undetermined factors. For example, test results depend on time since infection and sample type. At the start of the epidemic testing also tends to be reserved for contact tracing, and unless all cases are traced, it is thus a biased sample of ongoing transmission. The latter is a particularly problematic factor, since the vast majority of SARS-CoV-2 infections are known to be asymptomatic. Depending on local capacity and infrastructure, testing efforts may not be constant in time. In fact, as the epidemic progresses, testing capacity may be overwhelmed, or testing strategies may shift to target particular subgroups of the population.

For SARS-CoV-2, a second epidemic data source is the mortality time series. Here, we assume that death events (and thus MTS) are less prone to problems such as those that affect the representativeness of CTS versus the real but unknown infection time series (ITS). In particular, that death events should be more easily detected, since they only occur in symptomatic infections, they do not necessarily depend on the frailties of tests for current infection, and reporting of all death events in a community is generally mandatory and performed on already existing pathways in health systems. Deaths mostly occur among a proportion of the population at risk of severe disease [[5]](https://paperpile.com/c/V1tw35/Pi9s). A potentially long time period between infection and death has also been reported [[6]](https://paperpile.com/c/V1tw35/UMkHr). The MTS is expected to be both a lagged and undersampling of the ITS. Most importantly for the estimation of the epidemic growth rate, we here assume that the MTS should conserve the growth and shape of the unknown ITS.

In this supplementary material, we estimate the epidemic growth rate r of the ITS from the CTS and from the MTS independently. We use a maximum likelihood estimation approach together with a phenomenological model of exponential growth, and some well established theoretical formulations on how r relates to R. For the estimations based on MTS data, we include 3 Brazilian states - São Paulo (SP), Rio de Janeiro (RJ), Minas Gerais (MG) - and for comparison, also include MTS from the United Kingdom (UK), Italy (IT) and Spain (SN). For the estimations based on the CTS data, we include the 3 Brazilian states only.

### Methods

##### Phenomenological model

We used the model described in [[7]](https://paperpile.com/c/V1tw35/RuEA): $M\left( t \right)=$, where M(t) is the number of cumulative deaths in time t, m is a positive integer, $m=1/\left( 1-p \right)$, $A={M_{0}}^{1/m}$ with M0 the number of deaths at t=0, and p is the deceleration of growth parameter. The later exists in $0<p<1$, for which sub-exponential growth is obtained. For example, for $p=0.5$ growth is quadratic, and as $p\to1$ growth will tend to be purely exponential in the limit. We use this general formulation (instead of pure exponential formulation) to allow for further flexibility in future research. For the results presented, we assume growth is exponential, fixing p to 0.9999.

##### Maximum likelihood estimation of growth rate

Cumulative death counts are modelled according to the phenomenological model detailed above, and the negative log-likelihood of the data given the model is defined using a negative-binomial distribution. The function *mle2* from the R-package bbmle was used to estimate the growth rate r with default parameters and method set as ’Brent’ (for one dimensional MLE) [[8]](https://paperpile.com/c/V1tw35/RjrC)

##### Relationship of growth rate r with reproduction number R

The reproduction number R was estimated from the maximum likelihood estimated growth rate r in two different ways:

(i) The estimated growth rate r and an assumed serial interval distribution (SID) were used to calculate $R=$ with $a={m^{2}}/{s^{2}}$ and $b=m/{s^{2}}$, m being the SID mean and s the SID standard deviation. This approach is similar to that described in Imperial College London’s report 13 (ICL13) [[2]](https://paperpile.com/c/V1tw35/E89g). In this approach, no assumptions are made on the infectious, latent or incubation periods of SARS-CoV-2. The SID distribution used is the one estimated by Nishiura and colleagues [[9]](https://paperpile.com/c/V1tw35/QcQb), with m=4.7 and s=2.9 (also very similar to the ones used in [[2,10]](https://paperpile.com/c/V1tw35/E89g+Lxjk)). We term this approach the serial interval approach.

(ii) The estimated growth rate r and assumed prior distributions for the incubation and infectious periods of SARS-CoV-2 were used to calculate $R=\left( 1+r/\sigma\right)\left( 1+r/\delta\right)$, with $1/\sigma$ the infectious period (InfP) and $1/\delta$ the incubation period (IncP). This approach is the one described by Wallinga and Lipsitch [[11]](https://paperpile.com/c/V1tw35/aqaf), which is based on an SEIR modelling framework and expects both the InfP and IncP to be exponentially distributed. We assumed priors with exponential distributions with mean 5.1 days for IncP [[2,6,12–17]](https://paperpile.com/c/V1tw35/UMkHr+NO3Tc+d3zss+BxGkT+E89g+aosr+pfrD+uyQW) and 4 days for the InfP [[6,12,13,16]](https://paperpile.com/c/V1tw35/UMkHr+NO3Tc+d3zss+pfrD). We term this approach the SEIR estimation.

##### Doubling time, geographical distances and population size

Doubling time was calculated as $ln\left( 2 \right)/r$ with r the growth rate [[11]](https://paperpile.com/c/V1tw35/aqaf). Geographical distances (lat,lon) between death / case reports were calculated using the function *distVincentyEllipsoid* from the R-package geosphere R-package [[5,18]](https://paperpile.com/c/V1tw35/Pi9s+gGp3). We considered the approximate population sizes 40M for SP, 16.5M for RJ, 21M for MG, 66M for UK, 60M for IT and 47 for SN.

### Reproduction number results using the case time series (CTS)

Each of the 3 Brazilian states had CTS of different length (here starting on the date of the first reported case), and the growth rate appeared to vary in time within each state (Figure S3). Slowing down of the CTS has been described in many regions and is likely to be a consequence of changes in population behaviour and / or official social distancing interventions [[13,15,19]](https://paperpile.com/c/V1tw35/aosr+d3zss+Z75y).

| 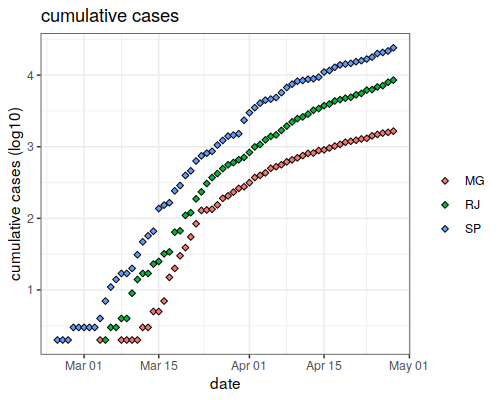 | **Figure S3.**  Log10 cumulative cases per Brazilian state and European country (coloured diamonds).  SP = São Paulo, MG = Minas Gerais, RJ = Rio de Janeiro. |
| --- | --- |

We divided the CTS of each state into several periods (according to total size) and performed the MLE of growth rate independently for those periods (MG: 4, RJ: 4, SP:5 equally spaced periods). A summary of the resulting fits is presented in Figure S4. The phenomenological model was able to approximate the CTS of each state (white points for data versus colored diamonds for model). As the CTS of all states slowed down with time, the mean estimated R also slowed down with time.

| 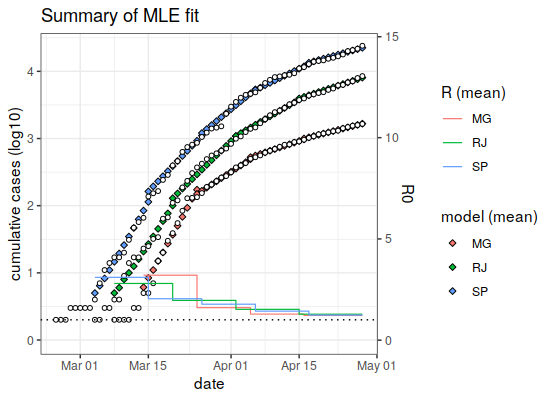 | **Figure S4.**  Log10 cumulative cases per Brazilian state (white points) versus mean model fit (colored diamonds).  Colored line segments are the estimated R mean for each of the considered time periods per state (colors).  Horizontal dotted line is R=1.  SP = São Paulo, MG = Minas Gerais, RJ = Rio de Janeiro. |
| --- | --- |

The posteriors for doubling times and R (from the SEIR approach) per time period are presented in Figure S5. Following the slow down of the CTS of each state, the doubling time increases and the R decreases with time. The doubling time posteriors present little variation, related only to the MLE estimated growth rate r. The R estimations present more variation, both from the MLE estimated growth rate r as well as from the sampling of the assumed priors for the incubation and infectious periods (described for the SEIR approach above).

| 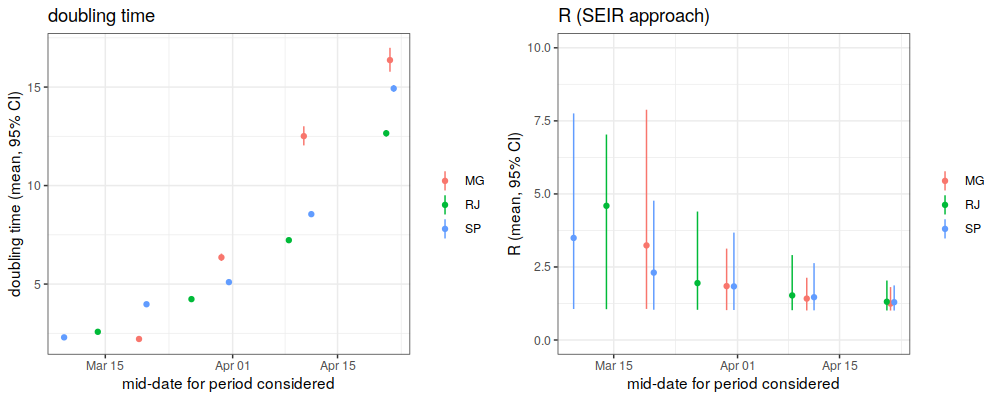 |
| --- |
| **Figure S5.** (A, left) Doubling times as estimated for each state / country per considered time period. (B, right) R as estimated for each state per considered time period (SEIR approach). SP = São Paulo, MG = Minas Gerais, RJ = Rio de Janeiro. |

We also looked at a posterior of R across the different fitted periods per region, by considering all of the posteriors from each period (Figure S6). The two methods of estimating R from the growth rate gave similar posteriors (albeit with different variation). For the SEIR approach, the estimates were: SP 2.07 (CI 95% 1.01-4.1); RJ 2.3 (CI 95% 1.02-4.0); and MG 1.9 (CI 95% 1.01-3.7). For the serial interval approach, the estimates were: SP 1.91 (CI 95% 1.2-3.1); RJ 1.88 (CI 95% 1.27-2.8); and MG 1.82 (CI 95% 1.2-3.25). These R ranges are similar between approaches and to others reported elsewhere after lockdown interventions [[12,16,20,21]](https://paperpile.com/c/V1tw35/pfrD+NO3Tc+gKXO+jp87). The first estimation for each state was higher, likely a representation of lower adherence or lack of lockdown guidelines initially, and importantly also in range with previously reported estimates on pre-lockdown [[13]](https://paperpile.com/c/V1tw35/d3zss)[[22]](https://paperpile.com/c/V1tw35/UZoq)[[23]](https://paperpile.com/c/V1tw35/8ir9) [[20]](https://paperpile.com/c/V1tw35/gKXO)[[24]](https://paperpile.com/c/V1tw35/agrk).

| 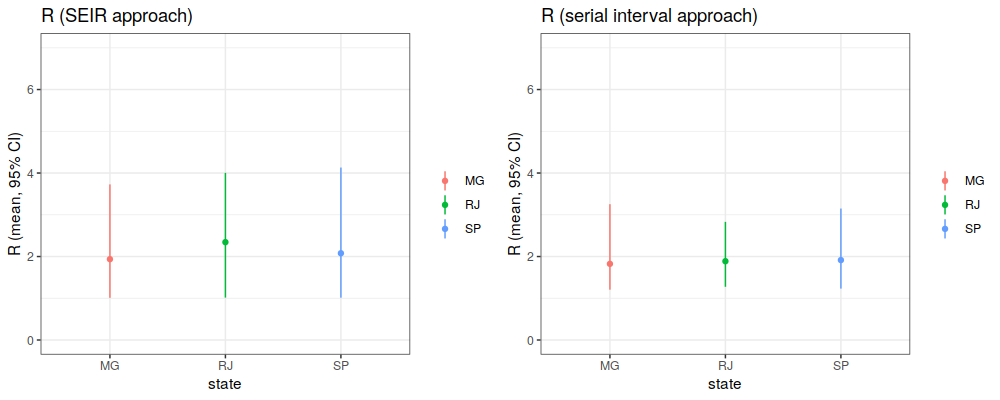 |
| --- |
| **Figure S6.** (A, left) R as estimated for each state / country for their entire CTS (SEIR approach). (B, right) R as estimated for each state / country for their entire CTS (serial interval approach). SP = São Paulo, MG = Minas Gerais, RJ = Rio de Janeiro. |

Incidence is typically calculated per 100K individuals (inc = 100K * cases / population size). However, the denominator in the normalisation (state population size) may not be representative of the total population affected by the virus in the Brazillian states. In other words, a more representative denominator could be the total population size of only the areas (within each state) that have had reported cases. We termed this population size the effective population size of each state - which was ~16.5M for RJ, ~40M for SP and ~13.6M for MG. In relation to the total population sizes of the states, this equated to ~100% of RJ, ~100% of SP and 64% of MG. When normalizing the CTS per 100K using the effective population sizes, the CTS for SP and RJ remained largely unchanged, but the CTS for MG was transformed to higher values (Figure S7). Thus, by 28/04/2020, using the effective population sizes, we calculate that the case incidence per 100K has been ~60 in SP, ~51 in RJ and ~7.85 in MG.

| 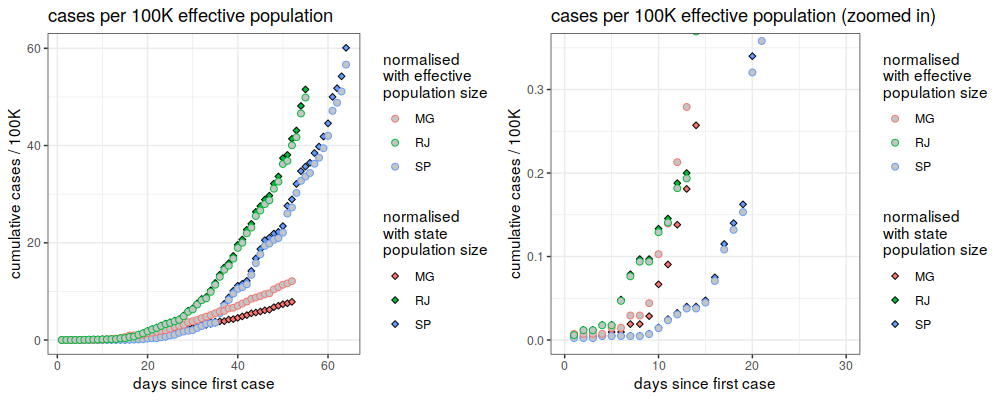 |
| --- |
| **Figure S7.** (A, left) Cumulative cases per effective 100K individuals per state / country with normalised time to day of first reported case and normalised to the maximum number of cases per CTS. See definition of effective population size in the text. (B, right) Same as A but zoomed in for the first 30 days. SP = São Paulo, MG = Minas Gerais, RJ = Rio de Janeiro. See main text for data sources. |

### Reproduction number results using the mortality time series (MTS)

Each of the MTS analysed had different lengths (here starting on the date of first death) (Figure S8). We thus divided the MTS into several periods and performed the MLE of growth rate independently for those periods (MG: 4, RJ: 4, SP:6, UK:7, SN:5 equally spaced periods). A summary of the resulting fits is presented in Figure S9. The phenomenological model is able to approximate the MTS of each state (white points for data versus colored diamonds for model). As the MTS of all states slowed down with time, the mean estimated R also slowed down.

| 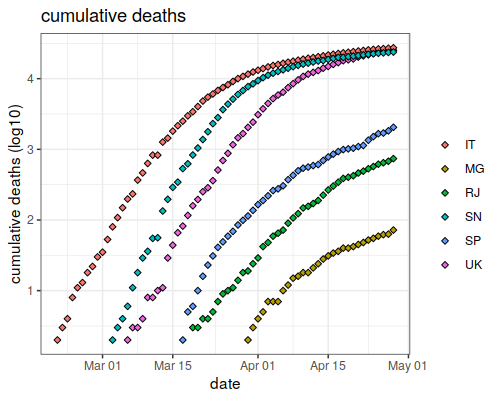 | **Figure S8.**  Log10 cumulative deaths per Brazilian state and European country (coloured diamonds).  SP = São Paulo, MG = Minas Gerais, RJ = Rio de Janeiro, IT = Italy, UK = United Kingdom and SN = Spain.  See main text for data sources. |
| --- | --- |

| 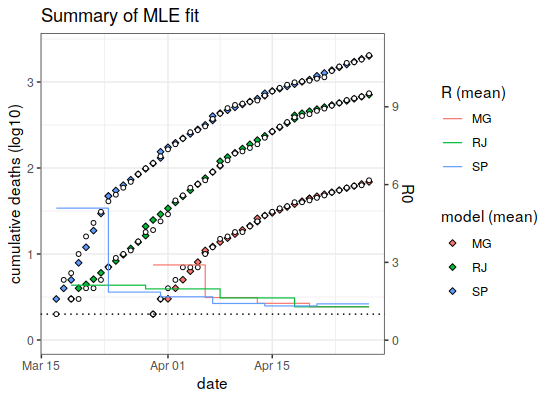 | **Figure S9.**  Log10 cumulative deaths per Brazilian state (white points) versus mean model fit (colored diamonds).  Colored line segments are the estimated R mean for each of the considered time periods per state (colors).  Horizontal dotted line is R=1.  SP = São Paulo, MG = Minas Gerais, RJ = Rio de Janeiro.  See main text for data sources. |
| --- | --- |

The posteriors for doubling times and R (from the SEIR approach) per time period are presented in Figure S10. Following the slow down of the MTS of each state, the doubling time increases and the R decreases with time. The doubling time posteriors present little variation, related only to the MLE estimated growth rate r.

| 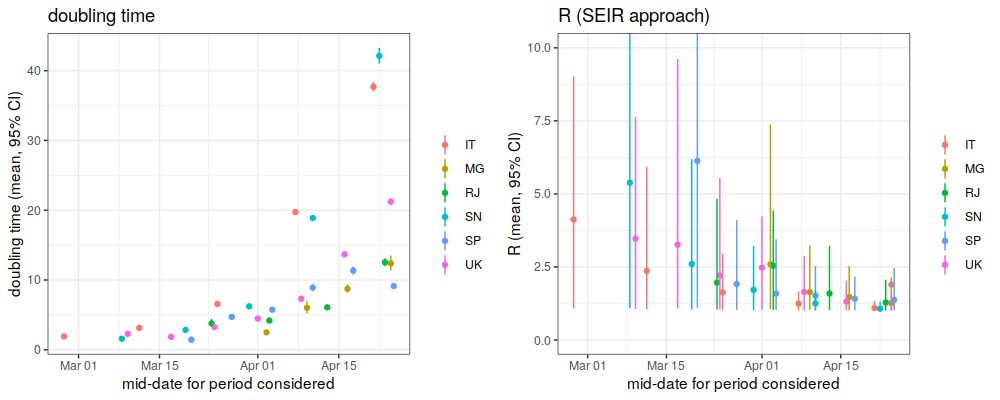 |
| --- |
| **Figure S10.** (A, left) Doubling times as estimated for each state / country per considered time period. (B, right) R as estimated for each state per considered time period (SEIR approach). SP = São Paulo, MG = Minas Gerais, RJ = Rio de Janeiro, IT = Italy, UK = United Kingdom and SN = Spain. |

The posterior of R across the different fitted periods per state are presented in Figure S11. The two methods of estimating R from the growth rate gave similar posteriors. For the SEIR approach, the estimates were: SP 2.32 (CI 95% 1.01-4.5); RJ 1.84 (CI 95% 1.01-3.66); and MG 1.74 (CI 95% 1.01-3.76). For the serial interval approach, the estimates were: SP 2.12 (CI 95% 1.3-5.2); RJ 1.75 (CI 95% 1.27-2.26); and MG 1.8 (CI 95% 1.26-3.03). These R ranges are similar to the ones obtained from the CTS and others reported elsewhere (see list of citations above in the section dedicated to R estimation from CTS).

| 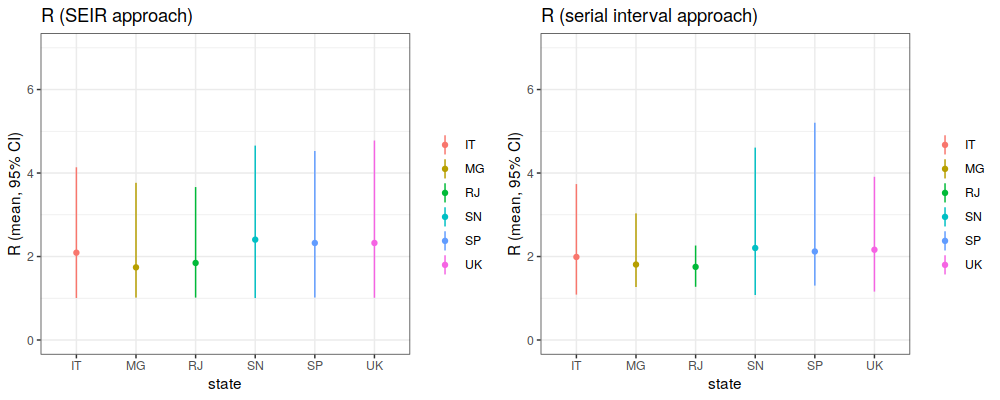 |
| --- |
| **Figure S11.** (A, left) R as estimated for each state / country for their entire MTS (SEIR approach). (B, right) R as estimated for each state / country for their entire MTS (serial interval approach). SP = São Paulo, MG = Minas Gerais, RJ = Rio de Janeiro, IT = Italy, UK = United Kingdom and SN = Spain. |

The effective population size of each state was 15.724.804 for RJ, 36.815.327 for SP and 7.486.968 for MG. In relation to the total population sizes of the states, this equated to ~95% of RJ, ~92% of SP and 35% of MG. When normalizing the MTS per 100K using the effective population sizes, the MTS for SP and RJ remained largely unchanged, but the MTS for MG was transformed to become similar to the other states (Figure S12). Thus, by 28/04/2020, using the effective population sizes, we calculate that the mortality incidence per 100K has been ~5.56 in SP, ~4.69 in RJ and ~0.94 in MG.

| 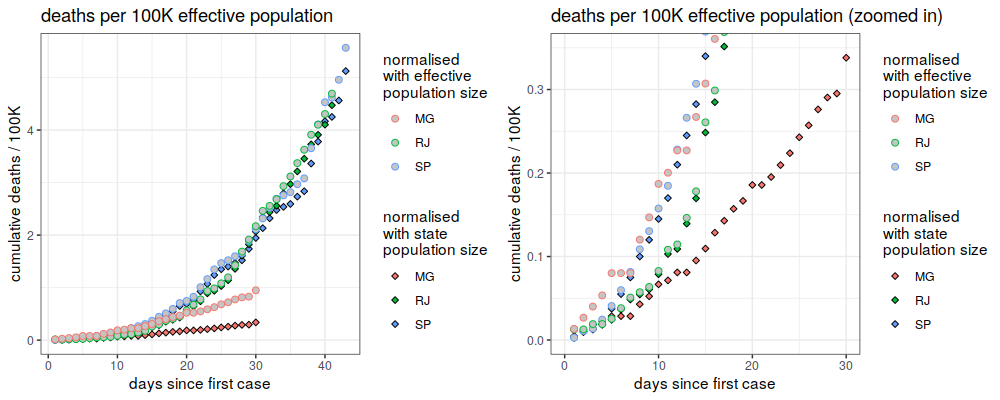 |
| --- |
| **Figure S12.** (A, left) Cumulative deaths per effective 100K individuals per state / country with normalised time to day of first reported death and normalised to the maximum number of deaths per MTS. See definition of effective population size in the text. (B, right) Same as A but zoomed in for the first 30 days. SP = São Paulo, MG = Minas Gerais, RJ = Rio de Janeiro. See main text for data sources. |

## Spatial results

##### Methods: geographical distances and population size

Geographical distances (lat,lon) between death / case reports were calculated using the function *distVincentyEllipsoid* from the R-package geosphere R-package [[5,18]](https://paperpile.com/c/V1tw35/Pi9s+gGp3). We considered the approximate population sizes 40M for SP, 16.5M for RJ, 21M for MG, 66M for UK, 60M for IT and 47 for SN.

### Results using the **cases** time series (CTS)

We mapped the reported cases within the Brazilian states (Figure S13). In contrast to SP and RJ, there appeared to be no clear signal around the capital city of MG (Belo Horizonte). This suggested that the cases in MG were more uniformly distributed in the state, compared to the other two states. The geo-location of each reported case was used to calculate the (pairwise) distance (km) distribution of all cases (Figure S14A). Cases in the MG were on average ~271 km away, ~104 in RJ and ~207 in SP. Differences between the distributions were significant with a Wilcox test. We also calculated the distribution of distances between the location of each case and the location of the capital city for each state (Figure S14B). In MG, reported deaths were on average ~103 km from the capital Belo Horizonte, while in SP they were ~0.05 km, and in RJ ~1.45 km away from their capital cities.

| 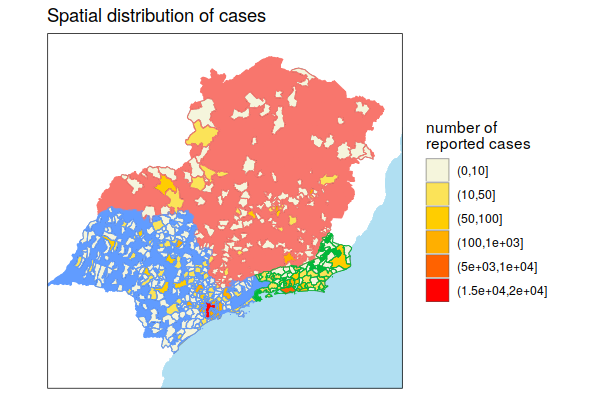 | **Figure S13.** Map with location (municipio) of cases, colored by total number of reports.  Different background colors highlight the boundaries of the 3 states: blue for SP = São Paulo, red for MG = Minas Gerais, green for RJ = Rio de Janeiro. |
| --- | --- |

| 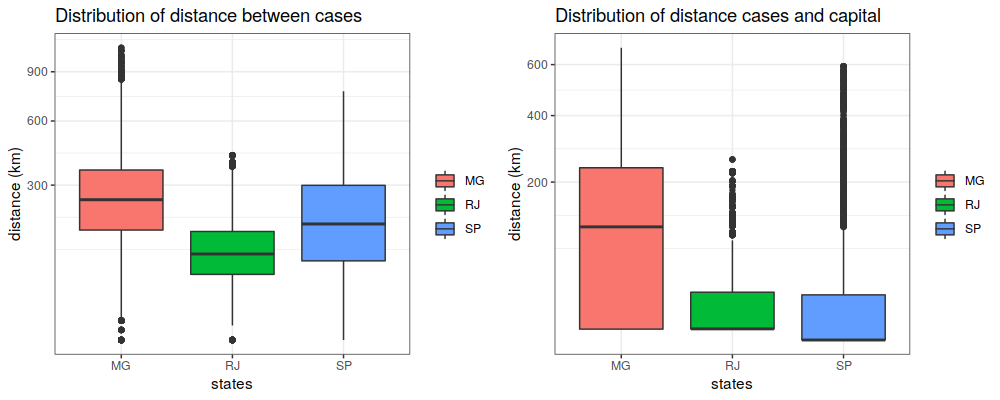 |
| --- |
| **Figure S14.** (A, left) Distribution of distances between each pair of reported cases by state. (B, right) Distribution of distances between each reported case and each state’s capital. 3 states: blue for SP = São Paulo, red for MG = Minas Gerais, green for RJ = Rio de Janeiro. |

### Results using the mortality time series (MTS)

The geographical distribution of reported deaths within the Brazilian states is presented in Figure S15. The distribution suggested that the cases in MG were more uniformly distributed in the state, compared to the other two states which appeared to have a higher number of cases close to their capital cities. The geo-location of each reported death was used to calculate the (pairwise) distance (km) distribution of all deaths (Figure S16A). Deaths in the MG were on average ~316 km away, ~86 in RJ and ~159 in SP. Differences between the distributions were significant with a Wilcox test. The distribution of distances between the location of each death event and the location of the capital city for each state (Figure S16B) also varied between states: in MG, reported deaths were on average ~229 km from the capital Belo Horizonte, while in SP they were ~28 km, and in RJ ~18 km away from their capital cities.

| 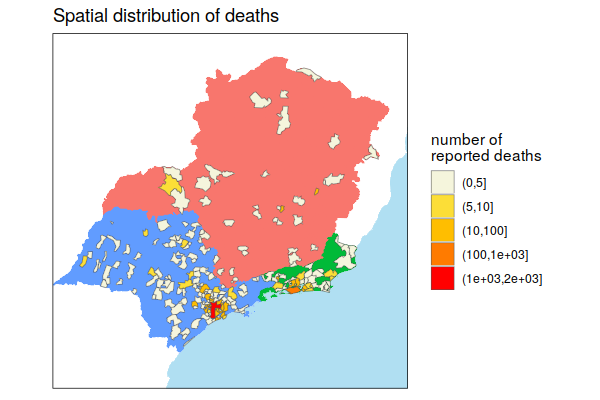 | **Figure S15.** Map with location (municipio) of death events, colored by total number of reports.  Different background colors highlight the boundaries of the 3 states: blue for SP = São Paulo, red for MG = Minas Gerais, green for RJ = Rio de Janeiro. |
| --- | --- |

| 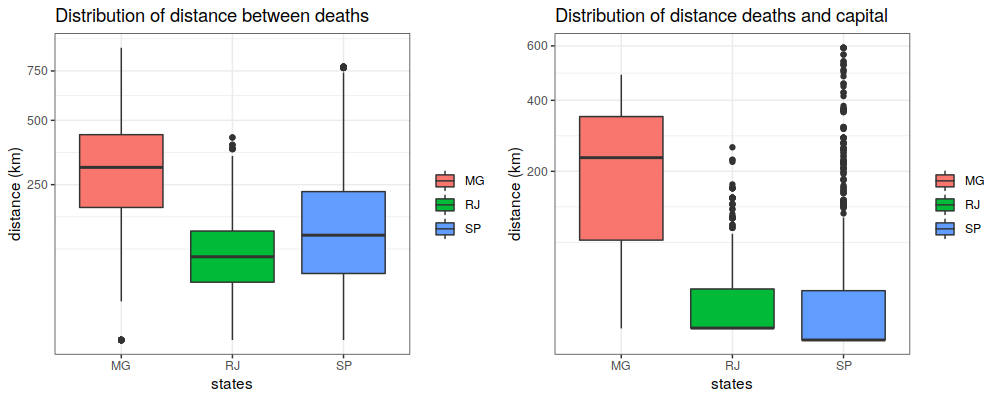 |
| --- |
| **Figure S16.** (A, left) Distribution of distances between each pair of reported deaths by state. (B, right) Distribution of distances between each reported death and each state’s capital. 3 states: blue for SP = São Paulo, red for MG = Minas Gerais, green for RJ = Rio de Janeiro. |


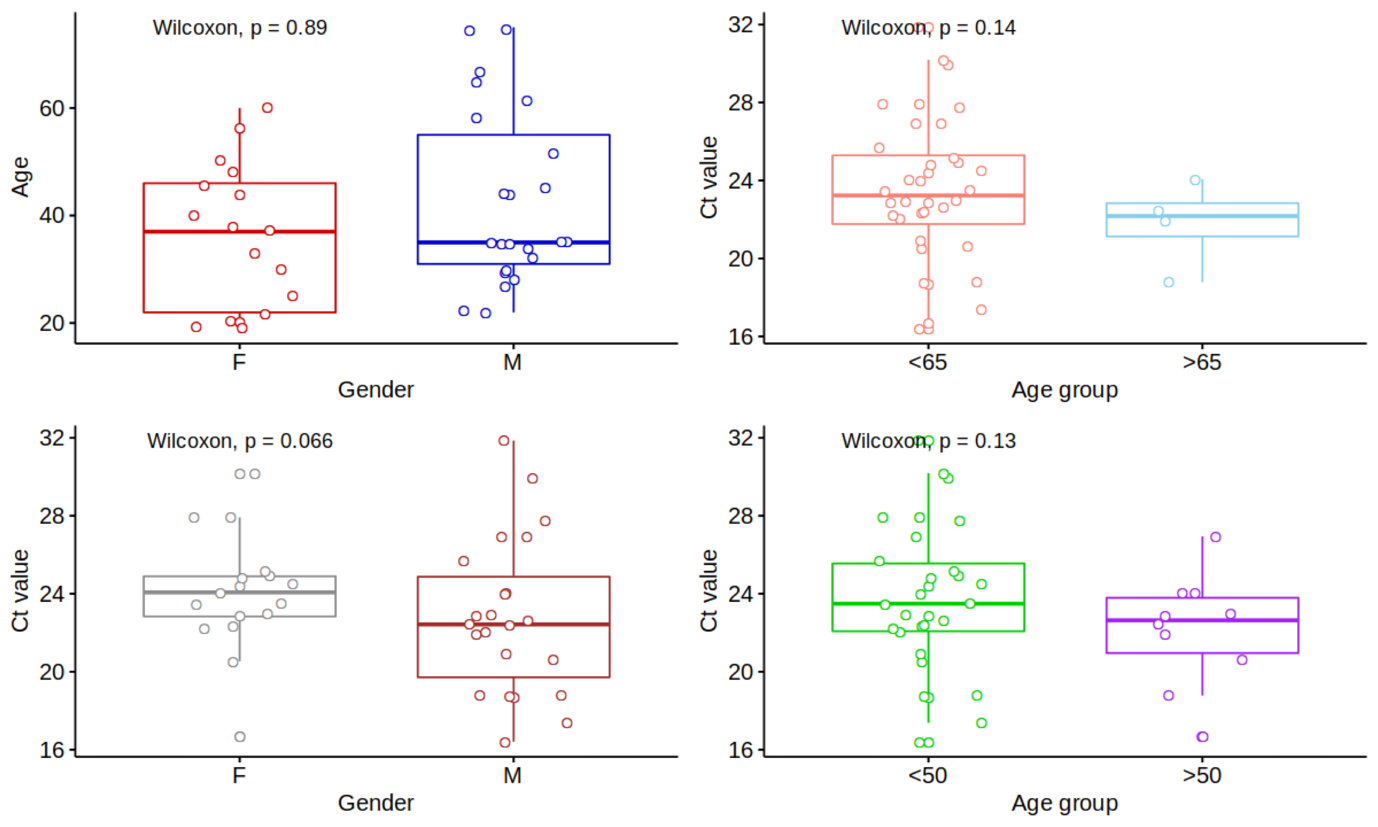


**S17 Fig. Comparisons between demographic variables and Ct values from sequenced samples.**  (top-left) Age of sample individuals versus gender. (top-right) Cycle threshold (Ct) for age groups <65 and >=65 years of age. (bottom-left) Ct for gender. (bottom- right) Ct for age groups <50 and >=50 years of age. Wilcoxon p-value is presented for every panel. None of the comparisons are statistically significant.

**Other materials**

**S1 Table.** Sequencing statistics for the 40 SARS-CoV-2 sequences generated.

| **Project-ID** | **Lab ID** | **Number Reads** | **Coverage (%)** | **Coverage depth** | **NT (%)** | **AA (%)** |
| --- | --- | --- | --- | --- | --- | --- |
| **CV1** | 47/20 | 38727 | 74.8 | 900.2 | 99.3 | 98.7 |
| **CV2** | 115/20 | 4355 | 93.3 | 91.5 | 97.3 | 94.9 |
| **CV3** | 135/20 | 709 | 85.2 | 32.2 | 97.7 | 95.6 |
| **CV4** | 242/20 | 701399 | 99.8 | 12199.7 | 99.9 | 99.8 |
| **CV5** | 252/20 | 51430 | 50.4 | 1779.2 | 99 | 97.7 |
| **CV6** | 298/20 | 886430 | 99.7 | 16407.7 | 99.8 | 99.6 |
| **CV7** | 352/20 | 128770 | 77.8 | 2851.1 | 99 | 98.3 |
| **CV8** | 399/20 | 394793 | 97.1 | 7225.1 | 99.5 | 99.1 |
| **CV9** | 428/20 | 25233 | 90 | 1352.2 | 98.4 | 96.9 |
| **CV11** | 607/20 | 151738 | 68.4 | 7436 | 98.8 | 98 |
| **CV12** | 615/20 | 250223 | 94.4 | 4613.1 | 99.3 | 99 |
| **CV13** | 660/20 | 269275 | 94 | 5721.7 | 99.3 | 98.9 |
| **CV16** | 791/20 | 714787 | 99.3 | 12517.7 | 99.8 | 99.7 |
| **CV17** | 809/20 | 253298 | 83.8 | 5328 | 99.2 | 98.6 |
| **CV18** | 833/20 | 94542 | 68.4 | 2383.6 | 98.8 | 97.7 |
| **CV19** | 836/20 | 297994 | 93.7 | 5630 | 99.1 | 98.6 |
| **CV20** | 838/20 | 175601 | 84.8 | 3603.1 | 98.8 | 97.8 |
| **CV21** | 842/20 | 759661 | 99.8 | 13968.4 | 99.9 | 99.8 |
| **CV22** | 895/20 | 120293 | 74.4 | 3051.8 | 98.9 | 97.9 |
| **CV24** | 1028/20 | 164278 | 77.2 | 3242.2 | 98.8 | 98 |
| **CV26** | 1078/20 | 543415 | 98.2 | 9570.9 | 99.5 | 99.1 |
| **CV27** | 1166/20 | 92936 | 81.1 | 1653.1 | 99.8 | 99.5 |
| **CV28** | 1142/20 | 108547 | 88.8 | 1841.2 | 99.4 | 98.8 |
| **CV31** | 1274/20 | 724502 | 99.8 | 11455 | 99.9 | 99.8 |
| **CV32** | 1290/20 | 840747 | 99.8 | 14924.3 | 99.9 | 99.8 |
| **CV33** | 1420/20 | 240018 | 95.8 | 4396 | 99.8 | 99.6 |
| **CV34** | 1467/20 | 2636 | 75 | 45.7 | 96.8 | 93.5 |
| **CV35** | 1500/20 | 4002 | 82.8 | 81.9 | 97.8 | 95.8 |
| **CV36** | 1504/20 | 19333 | 49.9 | 558.2 | 99.9 | 99.2 |
| **CV40** | 1834/20 | 43159 | 55.7 | 1266.7 | 99.8 | 99.2 |
| **CV41** | 1892/20 | 140516 | 70.1 | 2680 | 99.7 | 99.2 |
| **CV42** | 2119/20 | 819943 | 99.2 | 13993.2 | 99.8 | 99.7 |
| **CV43** | 2159/20 | 319317 | 91 | 5364 | 99.7 | 99.4 |
| **CV44** | 2196/20 | 36069 | 68.3 | 761.5 | 99.6 | 99.0 |
| **CV45** | 2241/20 | 87213 | 73 | 1916.8 | 99.8 | 99.3 |
| **CV46** | 2271/20 | 26075 | 58 | 770.7 | 99.9 | 99.5 |
| **CV47** | 2288/20 | 46863 | 71.4 | 970.3 | 99.7 | 99.1 |
| **CV48** | 2693/20 | 173017 | 90.6 | 3291.5 | 99.8 | 99.5 |
| **CV49** | 2801/20 | 425400 | 95.5 | 8121.3 | 99.8 | 99.6 |
| **CV50** | 5068/20 | 1127 | 75.6 | 21.8 | 97.9 | 96.1 |

Project-ID=sample identifier; Coverage (%) = percentage of genome coverage relative to the reference *NC_045512.3*; NT (%) = percentage of nucleotide identity; AA (%) = percentage of amino acid identity.

**S2 Table. Results from the lineage assessment.** The 40 new sequences from Minas Gerais were assesseed by pipeline named Phylogenetic Assignment of Named Global Outbreak LINeages available in github.

| **Taxon** | **Lineage** | **UFbootstrap** |
| --- | --- | --- |
| CV1_SARS-COV-2\|Brazil\|MinasGerais\|Ipatinga\|2020-03-04 | B.1.8 | 45 |
| CV2_BC02_SARS-COV-2\|Brazil\|MinasGerais\|SeteLagoas\|2020-03-08 | B.1 | 82 |
| CV3_BC03_SARS-COV-2\|MinasGerais\|BeloHorizonte\|2020-03-09 | B.1 | 95 |
| CV4_BC04_SARS-COV-2\|Brazil\|MinasGerais\|JuizdeFora\|2020-03-09 | B.1 | 94 |
| CV5_BC05_SARS-COV-2\|Brazil\|MinasGerais\|BeloHorizonte\|2020-03-12 | B | 72 |
| CV6_BC06_SARS-COV-2\|Brazil\|MinasGerais\|BeloHorizonte\|2020-03-13 | B.1 | 80 |
| CV7_BC07_SARS-COV-2\|Brazil\|MinasGerais\|BeloHorizonte\|2020-03-13 | A | 81 |
| CV8_BC08_SARS-COV-2\|Brazil\|MinasGerais\|BeloHorizonte\|2020-03-13 | B.1 | 76 |
| CV9_BC09_SARS-COV-2\|Brazil\|MinasGerais\|BeloHorizonte\|2020-03-13 | B | 81 |
| CV11_BC10_SARS-COV-2\|Brazil\|MinasGerais\|Mariana\|2020-03-16 | B | 89 |
| CV12_BC11_SARS-COV-2\|Brazil\|MinasGerais\|JuizdeFora\|2020-03-11 | B.1 | 74 |
| CV13_BC12_SARS-COV-2\|Brazil\|MinasGerais\|BeloHorizonte\|2020-03-15 | B.1 | 96 |
| CV16_BC13_SARS-COV-2\|Brazil\|MinasGerais\|BeloHorizonte\|2020-03-16 | B.1 | 76 |
| CV17_BC14_SARS-COV-2\|Brazil\|MinasGerais\|SeteLagoas\|2020-03-11 | B.1 | 83 |
| CV18_BC15_SARS-COV-2\|Brazil\|MinasGerais\|BeloHorizonte\|2020-03-16 | B.1 | 97 |
| CV19_BC16_SARS-COV-2\|Brazil\|MinasGerais\|BeloHorizonte\|2020-03-16 | B.1 | 69 |
| CV20_BC17_SARS-COV-2\|Brazil\|MinasGerais\|BeloHorizonte\|2020-03-16 | B.1 | 94 |
| CV21_BC18_SARS-COV-2\|Brazil\|MinasGerais\|BomDespacho\|2020-03-16 | B.1 | 82 |
| CV22_BC19_SARS-COV-2\|Brazil\|MinasGerais\|Mariana\|2020-03-16 | B.2 | 45 |
| CV24_BC20_SARS-COV-2\|Brazil\|MinasGerais\|Uberlandia\|2020-03-16 | B.1 | 98 |
| CV26_BC22_SARS-COV-2\|Brazil\|MinasGerais\|BeloHorizonte\|2020-03-17 | B.1 | 84 |
| CV27\|SARS-COV-2\|Brazil\|MinasGerais\|BoaEsperança\|2020-03-17 | B.1 | 100 |
| CV28_BC24_SARS-COV-2\|Brazil\|MinasGerais\|SaoJoaodelRei\|2020-03-17 | B.1 | 20 |
| CV31\|SARS-COV-2\|Brazil\|MinasGerais\|Betim\|2020-03-17 | B.1.5 | 75 |
| CV32\|SARS-COV-2\|Brazil\|MinasGerais\|Betim\|2020-03-17 | B.1 | 84 |
| CV33\|SARS-COV-2\|Brazil\|MinasGerais\|Sabara\|2020-03-17 | B.1 | 72 |
| CV34\|SARS-COV-2\|Brazil\|MinasGerais\|BeloHorizonte\|2020-03-16 | B.1 | 98 |
| CV35\|SARS-COV-2\|Brazil\|MinasGerais\|PocosDeCaldas\|2020-03-18 | B.1 | 99 |
| CV36\|SARS-COV-2\|Brazil\|MinasGerais\|Muriae\|2020-03-18 | B.2 | 46 |
| CV40\|SARS-COV-2\|Brazil\|MinasGerais\|BeloHorizonte\|2020-03-19 | B.1 | 99 |
| CV41\|SARS-COV-2\|Brazil\|MinasGerais\|SerraDoSalitre\|2020-03-18 | B.1 | 85 |
| CV42\|SARS-COV-2\|Brazil\|MinasGerais\|SaoJoaoDelRei\|2020-03-20 | B.1 | 97 |
| CV43\|SARS-COV-2\|Brazil\|MinasGerais\|Patrocinio\|2020-03-17 | B.1 | 73 |
| CV44\|SARS-COV-2\|Brazil\|MinasGerais\|Patrocinio\|2020-03-18 | B.1 | 75 |
| CV45\|SARS-COV-2\|Brazil\|MinasGerais\|Muriae\|2020-03-20 | B.1 | 99 |
| CV46\|SARS-COV-2\|Brazil\|MinasGerais\|BeloHorizonte\|2020-03-20 | B.1 | 100 |
| CV47\|SARS-COV-2\|Brazil\|MinasGerais\|BeloHorizonte\|2020-03-19 | B.1 | 99 |
| CV48\|SARS-COV-2\|Brazil\|MinasGerais\|Varginha\|2020-03-20 | B.1 | 84 |
| CV49\|SARS-COV-2\|Brazil\|MinasGerais\|BeloHorizonte\|2020-03-20 | B.1 | 66 |
| CV50\|SARS-COV-2\|Brazil\|MinasGerais\|Mariana\|2020-03-26 | B.1 | 95 |


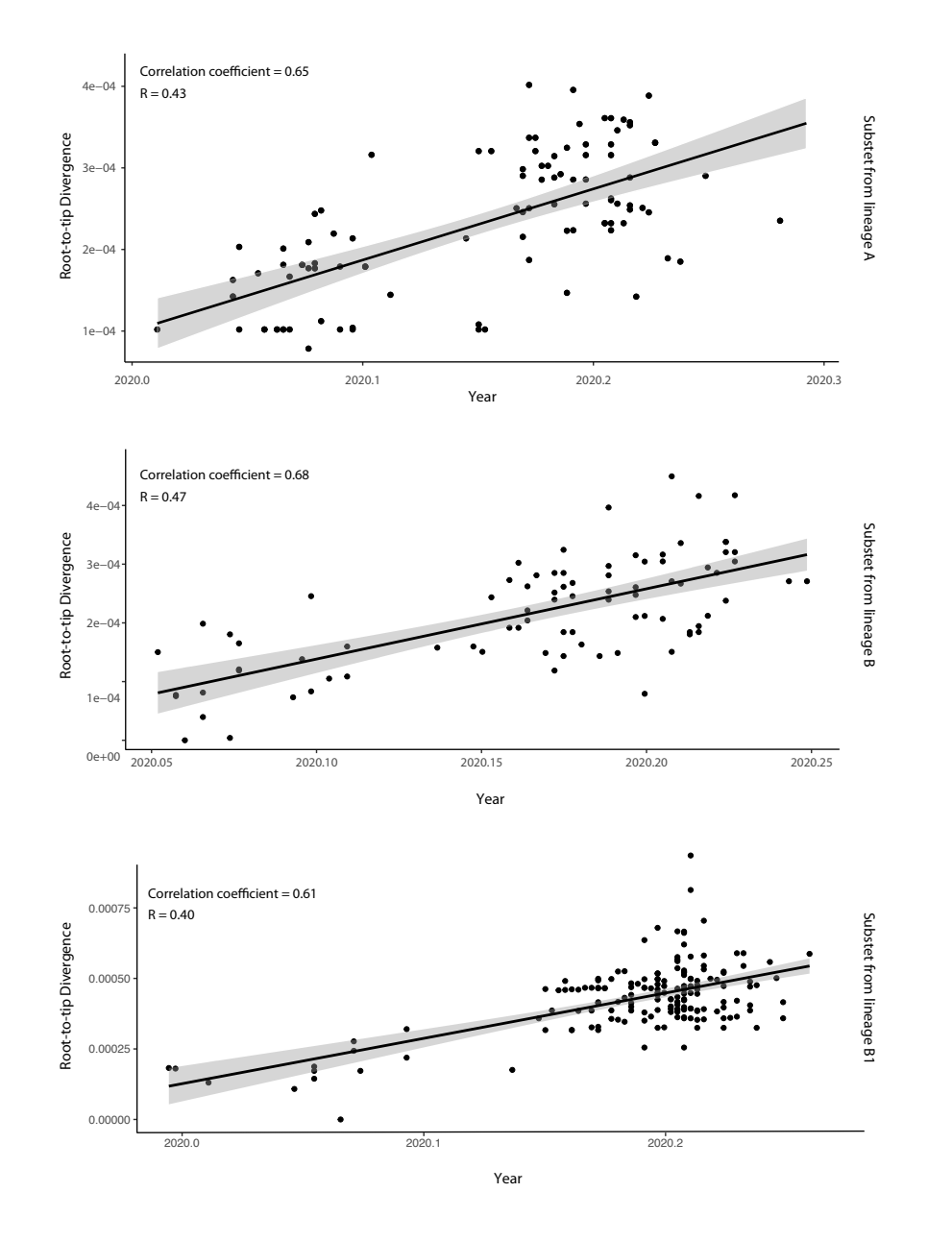


**Figure S18.** A regression of genetic divergence from root to tip against sampling dates using TemEst for each sub-dataset analysed in BEAST.

**References**

1. [Verity R, Okell LC, Dorigatti I, Winskill P, Whittaker C, Imai N, et al. Estimates of the severity of coronavirus disease 2019: a model-based analysis. Lancet Infect Dis. 2020. doi:](http://paperpile.com/b/V1tw35/o7Mh)[10.1016/S1473-3099(20)30243-7](http://dx.doi.org/10.1016/S1473-3099(20)30243-7)

2. [Flaxman S. Estimating the number of infections and the impact of non- pharmaceutical interventions on COVID-19 in 11 European countries. Imperial College COVID-19 Response Team. 2020. Available:](http://paperpile.com/b/V1tw35/E89g) <https://www.imperial.ac.uk/media/imperial-college/medicine/sph/ide/gida-fellowships/Imperial-College-COVID19-Europe-estimates-and-NPI-impact-30-03-2020.pdf>

3. [Russell TW, Hellewell J, Jarvis CI, van Zandvoort K, Abbott S, Ratnayake R, et al. Estimating the infection and case fatality ratio for coronavirus disease (COVID-19) using age-adjusted data from the outbreak on the Diamond Princess cruise ship, February 2020. Euro Surveill. 2020;25. doi:](http://paperpile.com/b/V1tw35/yBWi)[10.2807/1560-7917.ES.2020.25.12.2000256](http://dx.doi.org/10.2807/1560-7917.ES.2020.25.12.2000256)

4. [Wu JT, Leung K, Bushman M, Kishore N, Niehus R, de Salazar PM, et al. Estimating clinical severity of COVID-19 from the transmission dynamics in Wuhan, China. Nat Med. 2020;26: 506–510.](http://paperpile.com/b/V1tw35/WZfT)

5. [Zhou F, Yu T, Du R, Fan G, Liu Y, Liu Z, et al. Clinical course and risk factors for mortality of adult inpatients with COVID-19 in Wuhan, China: a retrospective cohort study. Lancet. 2020;395: 1054–1062.](http://paperpile.com/b/V1tw35/Pi9s)

6. [Linton NM, Kobayashi T, Yang Y, Hayashi K, Akhmetzhanov AR, Jung S-M, et al. Incubation Period and Other Epidemiological Characteristics of 2019 Novel Coronavirus Infections with Right Truncation: A Statistical Analysis of Publicly Available Case Data. J Clin Med Res. 2020;9. doi:](http://paperpile.com/b/V1tw35/UMkHr)[10.3390/jcm9020538](http://dx.doi.org/10.3390/jcm9020538)

7. [Chowell G, Sattenspiel L, Bansal S, Viboud C. Mathematical models to characterize early epidemic growth: A review. Phys Life Rev. 2016;18: 66–97.](http://paperpile.com/b/V1tw35/RuEA)

8. [bbmle R-package V1.0.23.1. Available:](http://paperpile.com/b/V1tw35/RjrC) <https://cran.r-project.org/web/packages/bbmle/index.html>

9. [Nishiura H, Linton NM, Akhmetzhanov AR. Serial interval of novel coronavirus (COVID-19) infections. Int J Infect Dis. 2020;93: 284–286.](http://paperpile.com/b/V1tw35/QcQb)

10. [He X, Lau EHY, Wu P, Deng X, Wang J, Hao X, et al. Temporal dynamics in viral shedding and transmissibility of COVID-19. Nat Med. 2020. doi:](http://paperpile.com/b/V1tw35/Lxjk)[10.1038/s41591-020-0869-5](http://dx.doi.org/10.1038/s41591-020-0869-5)

11. [Wallinga J, Lipsitch M. How generation intervals shape the relationship between growth rates and reproductive numbers. Proc Biol Sci. 2007;274: 599–604.](http://paperpile.com/b/V1tw35/aqaf)

12. [Kucharski AJ, Russell TW, Diamond C, Liu Y, Edmunds J, Funk S, et al. Early dynamics of transmission and control of COVID-19: a mathematical modelling study. Lancet Infect Dis. 2020. doi:](http://paperpile.com/b/V1tw35/NO3Tc)[10.1016/S1473-3099(20)30144-4](http://dx.doi.org/10.1016/S1473-3099(20)30144-4)

13. [Li Q, Guan X, Wu P, Wang X, Zhou L, Tong Y, et al. Early Transmission Dynamics in Wuhan, China, of Novel Coronavirus-Infected Pneumonia. N Engl J Med. 2020. doi:](http://paperpile.com/b/V1tw35/d3zss)[10.1056/NEJMoa2001316](http://dx.doi.org/10.1056/NEJMoa2001316)

14. [Woelfel R, Corman VM, Guggemos W, Seilmaier M, Zange S, Mueller MA, et al. Clinical presentation and virological assessment of hospitalized cases of coronavirus disease 2019 in a travel-associated transmission cluster. doi:](http://paperpile.com/b/V1tw35/BxGkT)[10.1101/2020.03.05.20030502](http://dx.doi.org/10.1101/2020.03.05.20030502)

15. [Salje H, Kiem CT, Lefrancq N, Courtejoie N, Bosetti P, Paireau J, et al. Estimating the burden of SARS-CoV-2 in France. doi:](http://paperpile.com/b/V1tw35/aosr)[10.1101/2020.04.20.20072413](http://dx.doi.org/10.1101/2020.04.20.20072413)

16. [Li R, Pei S, Chen B, Song Y, Zhang T, Yang W, et al. Substantial undocumented infection facilitates the rapid dissemination of novel coronavirus (SARS-CoV2). Science. 2020. doi:](http://paperpile.com/b/V1tw35/pfrD)[10.1126/science.abb3221](http://dx.doi.org/10.1126/science.abb3221)

17. [Davies NG, Klepac P, Liu Y, Prem K, Jit M, Eggo RM, et al. Age-dependent effects in the transmission and control of COVID-19 epidemics. doi:](http://paperpile.com/b/V1tw35/uyQW)[10.1101/2020.03.24.20043018](http://dx.doi.org/10.1101/2020.03.24.20043018)

18. [geosphere R-package 1.5-10. Available:](http://paperpile.com/b/V1tw35/gGp3) <https://cran.r-project.org/web/packages/geosphere/index.html>

19. [Tian H, Liu Y, Li Y, Wu C-H, Chen B, Kraemer MUG, et al. The impact of transmission control measures during the first 50 days of the COVID-19 epidemic in China. doi:](http://paperpile.com/b/V1tw35/Z75y)[10.1101/2020.01.30.20019844](http://dx.doi.org/10.1101/2020.01.30.20019844)

20. [Wu JT, Leung K, Leung GM. Nowcasting and forecasting the potential domestic and international spread of the 2019-nCoV outbreak originating in Wuhan, China: a modelling study. The Lancet. 2020. pp. 689–697. doi:](http://paperpile.com/b/V1tw35/gKXO)[10.1016/s0140-6736(20)30260-9](http://dx.doi.org/10.1016/s0140-6736(20)30260-9)

21. [Tindale L, Coombe M, Stockdale JE, Garlock E, Lau WYV, Saraswat M, et al. Transmission interval estimates suggest pre-symptomatic spread of COVID-19. doi:](http://paperpile.com/b/V1tw35/jp87)[10.1101/2020.03.03.20029983](http://dx.doi.org/10.1101/2020.03.03.20029983)

22. [Tang B, Wang X, Li Q, Bragazzi NL, Tang S, Xiao Y, et al. Estimation of the Transmission Risk of 2019-nCov and Its Implication for Public Health Interventions. SSRN Electronic Journal. doi:](http://paperpile.com/b/V1tw35/UZoq)[10.2139/ssrn.3525558](http://dx.doi.org/10.2139/ssrn.3525558)

23. [Cao Z, Zhang Q, Lu X, Pfeiffer D, Jia Z, Song H, et al. Estimating the effective reproduction number of the 2019-nCoV in China. doi:](http://paperpile.com/b/V1tw35/8ir9)[10.1101/2020.01.27.20018952](http://dx.doi.org/10.1101/2020.01.27.20018952)

24. [Zhao S, Lin Q, Ran J, Musa SS, Yang G, Wang W, et al. Preliminary estimation of the basic reproduction number of novel coronavirus (2019-nCoV) in China, from 2019 to 2020: A data-driven analysis in the early phase of the outbreak. Int J Infect Dis. 2020;92: 214–217.](http://paperpile.com/b/V1tw35/agrk)
